# Rational Chemical Design of Molecular Glue Degraders

**DOI:** 10.1021/acscentsci.2c01317

**Published:** 2023-04-11

**Authors:** Ethan S. Toriki, James W. Papatzimas, Kaila Nishikawa, Dustin Dovala, Andreas O. Frank, Matthew J. Hesse, Daniela Dankova, Jae-Geun Song, Megan Bruce-Smythe, Heidi Struble, Francisco J. Garcia, Scott M. Brittain, Andrew C. Kile, Lynn M. McGregor, Jeffrey M. McKenna, John A. Tallarico, Markus Schirle, Daniel K. Nomura

**Affiliations:** † Department of Chemistry, University of California, Berkeley, Berkeley, California 94720, United States; ‡ Novartis-Berkeley Translational Chemical Biology Institute, Berkeley, California 94720, United States; § Innovative Genomics Institute, Berkeley, California 94704, United States; ∥ Novartis Institutes for BioMedical Research, Emeryville, California 94608, United States; ⊥ Novartis Institutes for BioMedical Research, Cambridge, Massachusetts 02139, United States; # Department of Molecular and Cell Biology, University of California, Berkeley, Berkeley, California 94720, United States

## Abstract

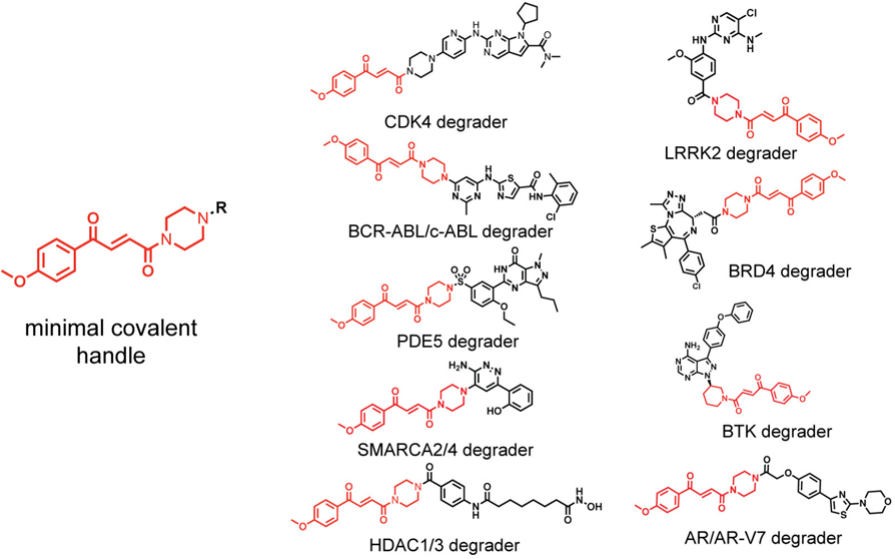

Targeted protein degradation with molecular glue degraders has arisen as a powerful therapeutic modality for eliminating classically undruggable disease-causing proteins through proteasome-mediated degradation. However, we currently lack rational chemical design principles for converting protein-targeting ligands into molecular glue degraders. To overcome this challenge, we sought to identify a transposable chemical handle that would convert protein-targeting ligands into molecular degraders of their corresponding targets. Using the CDK4/6 inhibitor ribociclib as a prototype, we identified a covalent handle that, when appended to the exit vector of ribociclib, induced the proteasome-mediated degradation of CDK4 in cancer cells. Further modification of our initial covalent scaffold led to an improved CDK4 degrader with the development of a but-2-ene-1,4-dione (“fumarate”) handle that showed improved interactions with RNF126. Subsequent chemoproteomic profiling revealed interactions of the CDK4 degrader and the optimized fumarate handle with RNF126 as well as additional RING-family E3 ligases. We then transplanted this covalent handle onto a diverse set of protein-targeting ligands to induce the degradation of BRD4, BCR-ABL and c-ABL, PDE5, AR and AR-V7, BTK, LRRK2, HDAC1/3, and SMARCA2/4. Our study undercovers a design strategy for converting protein-targeting ligands into covalent molecular glue degraders.

## Abstract 2

We identify a rational chemical design strategy for converting nondegradative protein-targeting ligands into molecular glue degraders of their target proteins.

## Introduction

Targeted protein degradation (TPD) has arisen as a powerful approach for destroying classically undruggable disease-causing proteins through ubiquitination and proteasome-mediated degradation. Two major strategic medicinal chemistry approaches exist for TPD: heterobifunctional proteolysis targeting chimeras (PROTACs) and monovalent molecular glue degraders. Both induce the proximity of an E3 ubiquitin ligase with a target protein leading to the ubiquitination and degradation of the protein in a proteasome-dependent manner.^1−4^ While PROTAC design is more modular wherein protein-targeting ligands can be connected via a linker to an E3 ligase recruiter, the discovery of novel molecular glue degraders has mostly been either fortuitous from phenotypic screens or via specific well-characterized E3 ligase-targeting recruiters (e.g., for cereblon).^5−11^ Given this landscape, a rational chemical design principle for converting protein-targeting ligands into molecular glue degraders is highly desired and would thereby facilitate a modular target-based design strategy of molecular glue degraders, which is currently lacking.

An example of a fortuitously discovered molecular glue degrader is thalidomide, which was originally developed as a morning sickness medicine but found to cause phocomelia birth defects. Immunomodulatory drug (IMiD) analogs of thalidomide have subsequently been developed as anticancer therapeutics. These IMiDs act through engaging in molecular glue interactions between the E3 ubiquitin ligase substrate receptor cereblon and various neosubstrate proteins such as SALL4 and Ikaros, leading to targeted ubiquitination and degradation of these proteins.^7,12−14^ Moving beyond fortuitous findings, cell-based phenotypic screening has also arisen as a powerful approach for discovering novel molecular glue degraders. Chemical screening for anticancer phenotypes coupled with counter-screening in hyponeddylation lines impaired in cullin E3 ligase activity with subsequent functional genomic or chemoproteomic mechanistic deconvolution has led to the discovery of novel molecular glue degraders for cyclin K and NFKB1.^15,16^

Several recent independent studies with specific protein targets and compounds have also revealed the possibility for converting protein-targeting ligands into molecular glue degraders through subtle chemical changes. These examples include: (1) CR8, a close analog of the nondegradative CDK12 inhibitor *(R)-*roscovitine, that engages in a ternary complex between CDK12-cyclin K and the CUL4 adaptor protein DDB1, leading to cyclin K ubiquitination and degradation;^17^ (2) BI-3802, a BCL6 inhibitor that was discovered to be a degrader of BCL6 through recognition of BI-3802-mediated polymerization filaments by the SIAH1 E3 ligase;^18^ (3) GNE-0011, MMH1, and MMH2, reversibly and irreversibly acting analogs of the nondegradative BRD4 inhibitor JQ1, that led to BRD4 degradation through ternary complex formation with the cullin E3 ligase substrate receptor DCAF16.^19−22^

There are thus many emerging innovative strategies for discovering novel molecular glue degraders, but their discoveries have largely been either through cell-based phenotypic screens or several independent one-off isolated examples of minor structural modifications to nondegradative inhibitors that have converted these compounds into degraders of their targets or associated protein complexes. The latter examples indicate that rational chemical design principles may exist to convert nondegradative protein-targeting ligands more systematically and modularly into molecular glue degraders of those targets, but such design principles are still poorly understood. Here, we have discovered a minimal covalent chemical moiety that can be appended onto various protein-targeting ligands to induce the degradation of their target proteins.

## Results

### Synthesis and Testing of Analogs with Chemical Handles Appended to the Exit Vector of the CDK4/6 Inhibitor Ribociclib

**Figure 1:**
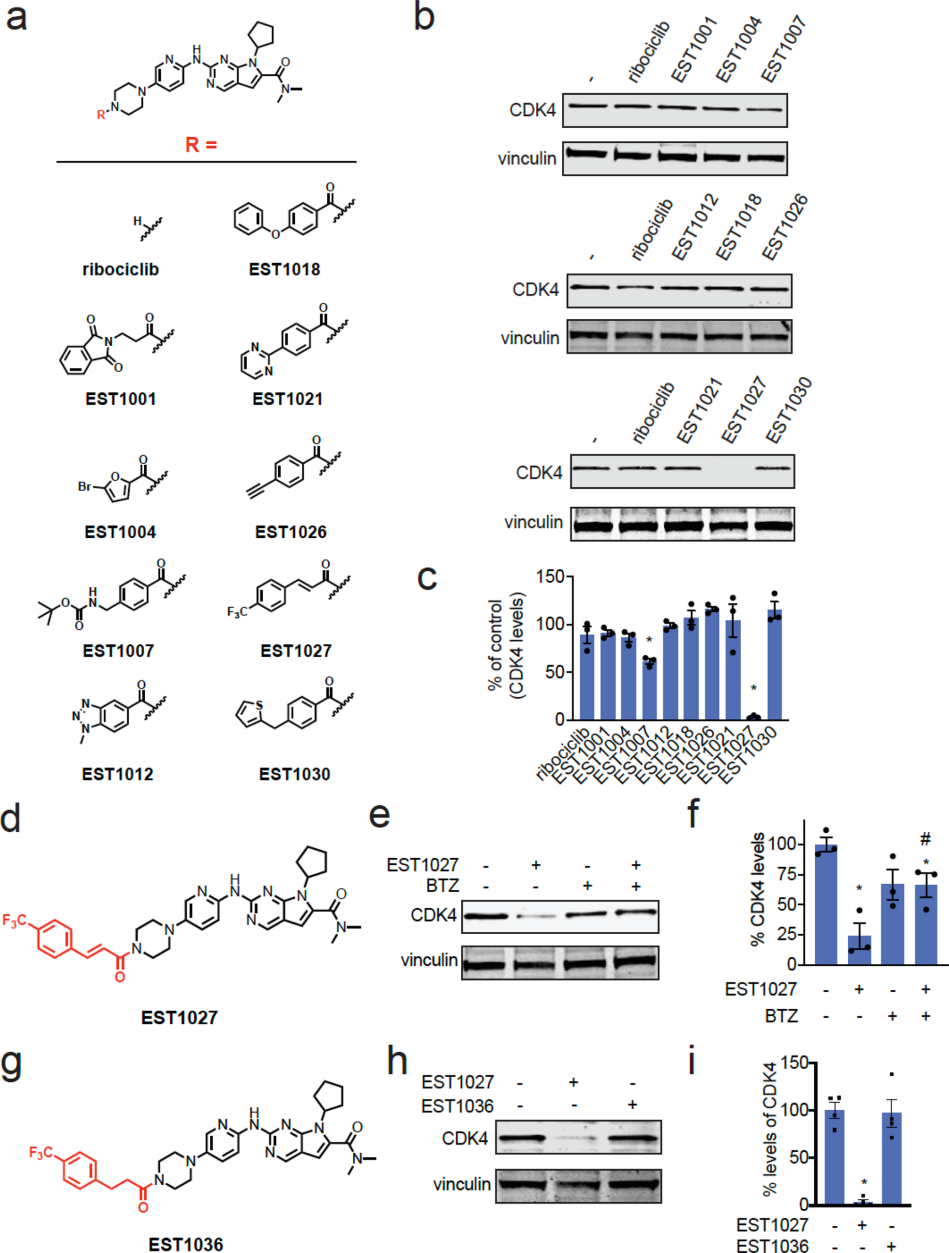
Synthesis and testing of analogs with chemical handles appended to the exit vector of the CDK4/6 inhibitor ribociclib. (a) Structures of ribociclib analogs wherein various chemical handles were appended onto the solvent-exposed end of ribociclib. (b) Testing ribociclib analogs in C33A cervical cancer cells to identify compounds that reduce CDK4 levels. C33A cells were treated with DMSO vehicle or compounds (3 μM) for 24 h. CDK4 and loading control vinculin levels were assessed by Western blotting. (c) Quantification of the data shown in (b). (d) Full structure of hit compound EST1027 that showed >50% loss of CDK4 in (b,c) with the appended chemical handle shown in red. (e) Proteasome-dependent degradation of CDK4 by EST1027. C33A cells were pretreated with DMSO vehicle or the proteasome inhibitor bortezomib (BTZ) (10 μM) 1 h prior to treatment of cells with DMSO vehicle or EST1027 (5 μM), and CDK4 and loading control vinculin levels were assessed by Western blotting. (f) Quantification of the experiment described in (e). (g) Structure of EST1036, a nonreactive derivative of EST1027. (h) EST1036 does not degrade CDK4. C33A cells were treated with DMSO vehicle or compounds (5 μM) for 24 h, and CDK4 and loading control vinculin levels were assessed by Western blotting. (i) Quantification of experiment in (h). Blots shown in (b,e,h) are representative of *n* = 3 biologically independent replicates/group. Bar graphs in (c,f,i) show individual replicate values and average ± sem. Statistical significance is calculated as *p < 0.05 compared to DMSO vehicle in (c,f,i) and #*p* < 0.05 compared to the EST1027-treated group in (f).

To identify potential chemical handles that could convert protein-targeting ligands into molecular glue degraders of their targets, we appended various moieties onto the solvent-exposed piperazine of the CDK4/6 inhibitor ribociclib (Figure 1a).^23^ Among the nine initial ribociclib analogs tested, we found one compound, EST1027, a trifluoromethylphenyl cinnamamide, that led to >50% reduction in CDK4, but not CDK6, levels in C33A cervical cancer cells treated for 24 h at 3 μM (Figures 1b–d and S1a**)**. This EST1027-mediated reduction in CDK4 occurred via proteasome-mediated degradation, since pretreatment of C33A cells with the proteasome inhibitor bortezomib attenuated EST1027-mediated CDK4 degradation (Figure 1e,f). Tandem mass tagging (TMT)-based quantitative proteomic analysis revealed a significant reduction in CDK4 levels in C33A cells after treatment with EST1027, alongside 100 other proteins that were significantly reduced out of >5000 total proteins quantified (Figure S1b and Table S1). The moderate selectivity observed may be due to downstream protein level changes resulting from CDK4 degradation or potential off-target effects of the cinnamamide handle. The cinnamamide motif is a possible covalent substrate that can undergo 1,4-addition with a cysteine thiolate anion. Confirming the necessity of this covalent functionality for the degradation of CDK4, a nonreactive trifluoromethylphenyl propionamide analog, EST1036, did not induce the degradation of CDK4 in C33A cells (Figure 1g–i).

### Structure–Activity Relationship of CDK4 Degrader

Encouraged by these data, we postulated that this trifluoromethylphenyl cinnamamide moiety could be appended onto other protein-targeting ligands to induce their degradation. Thus, we next appended this motif onto another structurally similar CDK4/6 inhibitor, palbociclib, to generate EST1090 (Figure S1c). Disappointingly, EST1090 did not degrade CDK4 in C33A cells (Figure S1d), indicating that this chemical motif was not generalizable and could not be transplanted onto other even very similar protein-targeting ligands to induce targeted degradation.

**Figure 2:**
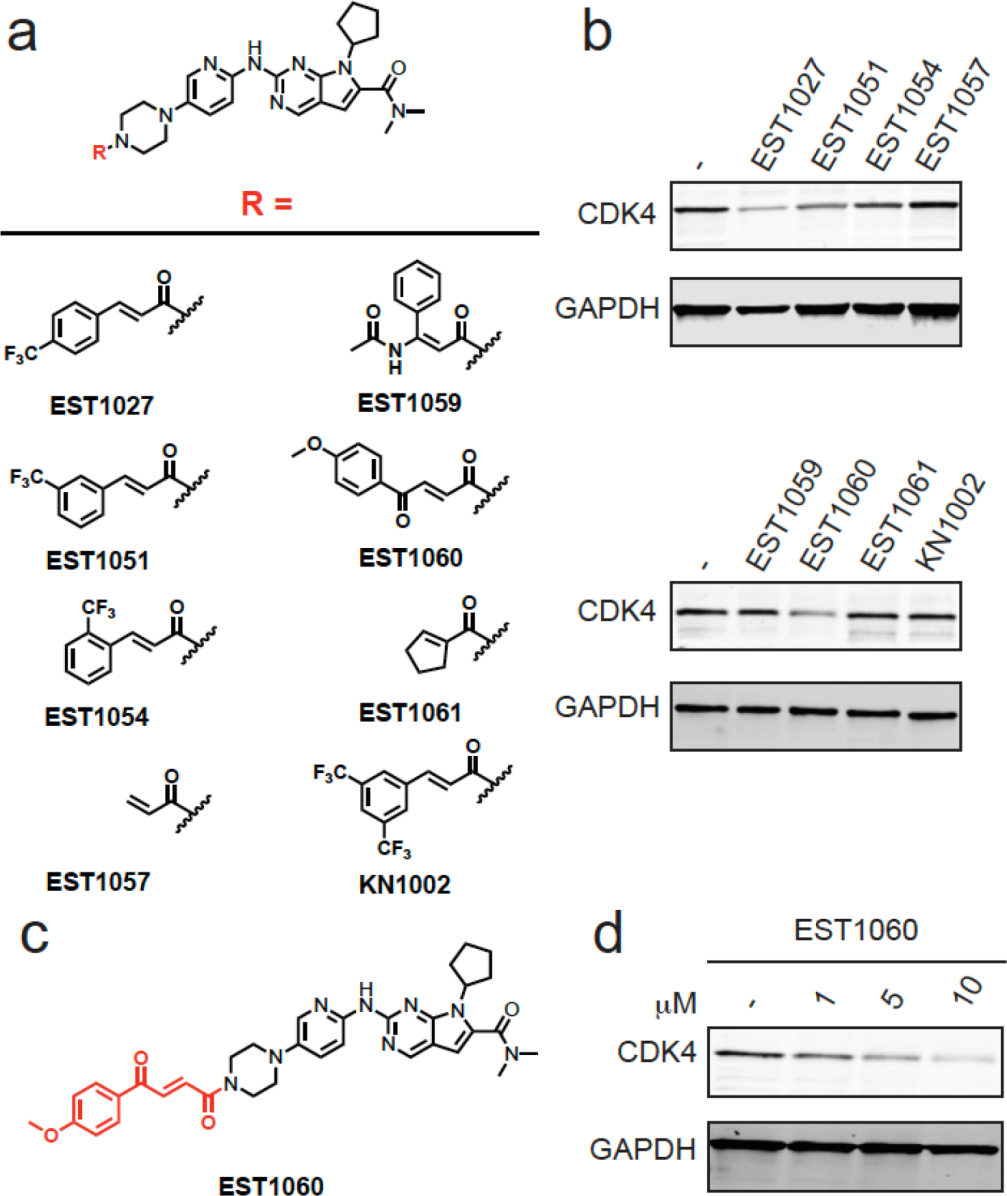
Structure–activity relationship of CDK4 degrader. (a) Structures of EST1027 analogs assessing structure–activity relationships. (b) Testing EST1027 analogs in C33A cervical cancer cells to identify compounds that reduce CDK4 levels. C33A cells were treated with DMSO vehicle or compounds (5 μM) for 24 h. CDK4 and loading control GAPDH levels were assessed by Western blotting. (c) Full structure of hit compound EST1060. (d) Dose–response of EST1060 CDK4 degradation. C33A cells were treated with DMSO vehicle or EST1060 for 24 h. CDK4 and loading control GAPDH levels were assessed by Western blotting. Gels and blots in (b,d) are representative images from *n* = 3 biologically independent replicates/group.

We therefore sought to explore structure–activity relationships of the cinnamamide motif for CDK4 degradation. Still using ribociclib as our testbed, we generated seven additional analogs to identify a better covalent chemical module (Figure 2a). Interestingly, moving the trifluoromethyl moiety from the *para*- to *ortho-* or *meta*- positions with EST1051, EST1054, and KN1002 abrogated CDK4 degradation in C33A cells, giving further support to specific interactions of these degrader compounds with an E3 ligase rather than nonspecific mechanisms (Figure 2a,b). We note that EST1027 caused a modest degree of C33A cytotoxicity that was also observed with close structural analogs EST1051 and EST1054, which did not degrade CDK4, indicating that general cytotoxicity was likely not mediating CDK4 loss (Figure S1e). Merely appending an acrylamide handle with EST1057 also did not cause CDK4 degradation (Figure 2a,b). Among additional derivatives tested, we observed improved dose-responsive CDK4 degradation with EST1060, containing a methoxyphenyl but-2-ene-1,4-dione (“fumarate derivative”), in C33A cells (Figure 2a–d). A nonreactive derivative of EST1060, JP-2-230, did not degrade CDK4 in C33A cells (Figure S2a,b). EST1060 also did not degrade CDK6 (Figure S2c). We next appended this fumarate module onto palbociclib to generate EST1089 (Figure S2d). EST1089 was now capable of degrading CDK4 (Figure S2e), suggesting that this moiety may be a more versatile chemical handle when compared to our original cinnamamide handle.

### Mapping the Proteome-Wide Targets of the Covalent Handle

**Figure 3:**
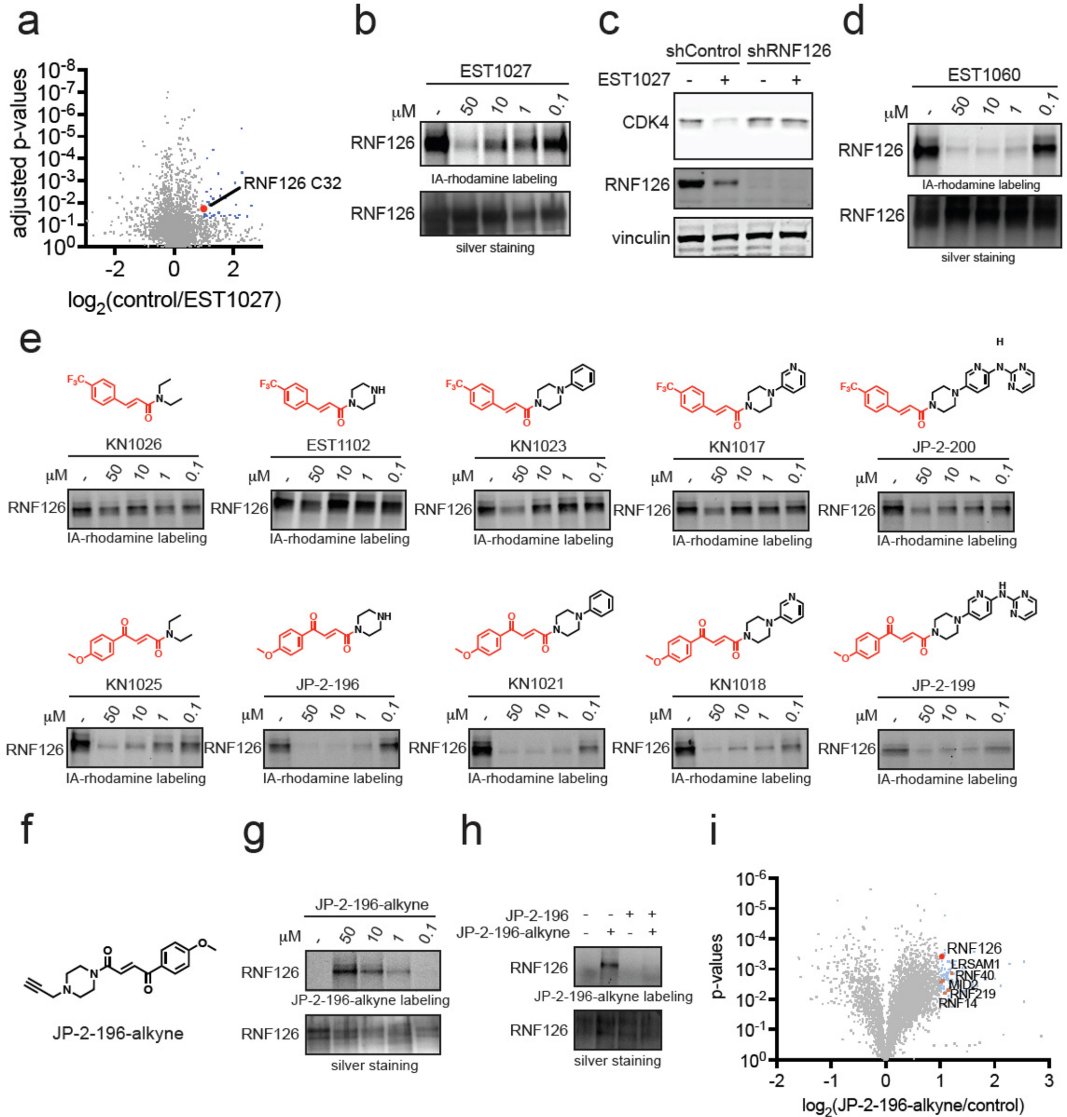
Mapping proteome-wide interactions of the fumarate handle. (a) Cysteine chemoproteomic profiling of EST1027 in C33A cervical cancer cells using isoDTB-ABPP. C33A cells were treated with DMSO vehicle or EST1027 (20 μM) for 2 h. Resulting lysates were labeled with an alkyne-functionalized iodoacetamide probe (IA-alkyne) (200 μM) for 1 h, after which isotopic desthiobiotin tags were appended by copper-catalyzed azide–alkyne cycloaddition (CuAAC) and taken through the isoDTB-ABPP procedure. Shown in blue and red are probe-modified cysteines that showed control/EST1027 ratios >2 with *p* < 0.05 from *n* = 3 biologically independent replicates. Shown in red is RNF126 C32. (b) Gel-based ABPP of EST1027 against RNF126. Recombinant RNF126 was preincubated with DMSO vehicle or EST1027 for 30 min prior to labeling of RNF126 with IA-rhodamine (250 nM) for 1 h. Gels were visualized by in-gel fluorescence, and protein loading was assessed by silver staining. (c) RNF126 knockdown attenuates EST1027-mediated CDK4 degradation. RNF126 was stably knocked down in C33A cells using short hairpin oligonucleotides (shRNF126) compared to nontargeting shControl oligonucleotides. C33A shControl and shRNF126 cells were treated with DMSO vehicle or EST1027 (5 μM) for 24 h. CDK4, RNF126, and loading control vinculin levels were assessed by Western blotting. (d) Gel-based ABPP of EST1060 against RNF126 performed as described in (b). (e) Gel-based ABPP of covalent chemical handles against RNF126 performed as described in (b). (f) Structure of JP-2-196-alkyne probe. (g) JP-2-196-alkyne labeling of pure RNF126 protein. RNF126 was labeled with DMSO vehicle or JP-2-196-alkyne for 30 min. Probe-modified RNF126 was subjected to CuAAC with a rhodamine-functionalized azide handle and visualized by SDS/PAGE and in-gel fluorescence. (h) Competition of JP-2-196-alkyne labeling of RNF126 by JP-2-196. RNF126 pure protein was preincubated with JP-2-196 (50 μM) for 30 min at 37 °C prior to JP-2-196 labeling (50 μM) for 30 min at room temperature. Probe-modified RNF126 was subjected to CuAAC with a rhodamine-functionalized azide handle and visualized by SDS/PAGE and in-gel fluorescence. (i) JP-2-196-alkyne pulldown proteomics showing significant and moderately selective engagement of RNF126 and five additional E3 ubiquitin ligases LRSAM1, RNF40, MID2, RNF219, and RNF14. HEK293T cells were treated with DMSO vehicle or JP-2-196-alkyne (10 μM) for 6 h. Subsequent lysates were subjected to CuAAC with an azide-functionalized biotin handle, after which probe-modified proteins were avidin-enriched, eluted, and digested, and analyzed by TMT-based quantitative proteomics. Data shown are ratios of JP-2-196-alkyne vs DMSO-control-enriched proteins and *p*-values from *n* = 3 biologically independent replicates/group. Gels and blots from (b–e,g,h) are representative of *n* = 3 biologically independent replicates/group.

Based on our data indicating that the covalent functionality on EST1027 and EST1060 was necessary to induce the degradation of CDK4 (Figures 1g–i and S2b), we postulated that this motif was leading to the covalent recognition of E3 ubiquitin ligases, leading to molecular glue interactions between E3 ligases and CDK4 and subsequent ubiquitination and proteasome-mediated degradation of CDK4. To identify E3 ligases covalently targeted by our original EST1027 CDK4 degrader, we performed isotopically labeled desthiobiotin azide-tag-based activity-based protein profiling (isoDTB-ABPP)^24^ in which we treated C33A cells *in situ* with vehicle or EST1027 and subsequently labeled resulting cell lysates with an alkyne-functionalized iodoacetamide probe to identify cysteines that were highly engaged by EST1027 across the proteome.^25−28^ Out of 3772 quantified probe-modified cysteines, we identified 49 targets that showed control/EST1027 ratios of >2 with a *p*-value of *p* < 0.05. Among these 49 targets, we identified a zinc-coordinating cysteine 32 (C32) on the RING-family E3 ubiquitin ligase RNF126 as a putative target of EST1027 that showed a control/EST1027 ratio of 2.0 (Figure 3a and Table S2).^29^ We posited that labeling by EST1027 of only one of the zinc-coordinating cysteines still allows functional zinc coordination with the other cysteines (C13, C16, and C29), maintaining RNF126 function. RNF126 was the only protein involved in the ubiquitin proteasome system among these targets, and as such, the other targets were not pursued for further characterization. RNF126 is an important E3 ligase involved in cellular protein quality control and is necessary to ubiquitinate and degrade mislocalized proteins in the cytosol or membrane proteins from the endoplasmic reticulum through its association with BAG6 and ubiquitination of BAG6-associated client proteins.^30,31^

We next confirmed the interaction of EST1027 with RNF126 by gel-based ABPP, showing dose-responsive competition of EST1027 against rhodamine-functionalized iodoacetamide cysteine labeling of recombinant RNF126 (Figure 3b). RNF126 knockdown completely attenuated EST1027-mediated CDK4 degradation in C33A cells, demonstrating that RNF126 was at least partially involved in the degradation of CDK4 with EST1027 (Figures 3c and S3a). Interestingly, we also observed significant reduction in RNF126 levels with EST1027 treatment, which was potentially achieved through the proteasomal degradation of the CDK4-EST027-RNF126 ternary complex (Figures 3c and S3b). EST1090, the palbociclib derivative with the cinnamamide handle which did not degrade CDK4, did not bind to RNF126 (Figure S3c).

Interestingly, we found that EST1060, with the fumarate handle, displaced cysteine-reactive probe labeling of pure RNF126 much more potently compared to EST1027 (Figure 3d). The nonreactive derivative of EST1060, JP-2-230, interestingly still showed binding to RNF126, albeit weaker than EST1060, indicating the fumarate motif may possess reversible binding affinity to RNF126 beyond its inherent reactivity (Figure S3d). We observed similar potent interactions with the palbociclib-based, fumarate CDK4 degrader EST1089 as EST1060 (Figure S3e). Mass spectrometry analysis of tryptic digests from RNF126 incubated with EST1027 or EST1060 further confirmed C32 as the site of modification (Figure S4a–d).

### Identifying the Minimal Covalent Chemical Handle Required for RNF126 Interactions

We next sought to understand the minimal chemical functionality and pharmacophore necessary to covalently interact with RNF126. To achieve this, we reverse engineered the EST1027 and EST1060 structures by taking their respective covalent handles and iteratively appending increasing portions of the ribociclib scaffold to assess their labeling of RNF126 by gel-based ABPP (Figure 3e). With trifluoromethylphenyl cinnamic acid appended to the minimal diethylamine moiety, KN1026, no RNF126 labeling was observed. In contrast, the methoxyphenyl fumarate handle linked to the diethylamine moiety, KN1025, showed labeling of RNF126, albeit weaker than EST1060 (Figure 3e). While appending a piperazine moiety onto trifluoromethylphenyl cinnamic acid to form EST1102 still did not confer labeling of RNF126, appending this substituent to the methoxyphenyl fumarate handle yielded JP-2-196, which showed significant potency comparable to that of EST1060 (Figure 3e). With the trifluoromethylphenyl cinnamamide handle, installing phenylpiperazine or pyridinylpiperazine substituents, KN1023 or KN1017, respectively, began to show binding to RNF126 with comparable potency to that observed with EST1027 (Figure 3e). However, this required linking a substantial portion of the ribociclib structure as exemplified by JP-2-200 (Figure 3e). In contrast, growing substituents on the methoxyphenyl fumarate handle with equivalent moieties, KN1021, KN1018, and JP-2-199, did not substantially improve potency against RNF126 beyond that observed with the piperazine substituent alone of JP-2-196 (Figure 3e). These data collectively showed that the covalent fumarate-derived motif is a better ligand for RNF126 and the *p*-methoxyphenylpiperazinyl fumarate JP-2-196 handle is the best minimal unit identified so far for covalently engaging RNF126.

To further map the targets of this optimized fumarate handle, we synthesized an alkyne-functionalized probe JP-2-196-alkyne (Figure 3f). We first demonstrated covalent and dose-responsive labeling of pure RNF126 protein with JP-2-196-alkyne by gel-based ABPP and that this labeling was attenuated upon pretreatment with JP-2-196 (Figure 3g,h). To assess the targets of this JP-2-196-alkyne probe, we next treated HEK293T cells with either the JP-2-196-alkyne probe or vehicle and subsequently appended an azide-functionalized biotin enrichment handle through copper-catalyzed “click-chemistry” followed by avidin enrichment of probe-modified peptides to assess probe-enriched proteins by quantitative proteomics (Figure 3i and Table S3). We identified 23 distinct protein targets that were significantly (*p* < 0.001) enriched by the JP-2-196-alkyne probe over vehicle control by >2-fold, of which RNF126 was the only E3 ligase among these 23 targets (Figure 3i). Using a less stringent filter, an additional 87 proteins were significantly (*p* < 0.01) enriched by the JP-2-196-alkyne probe by >2-fold, which included five additional E3 ligases—RNF40, MID2, RNF219, RNF14, and LRSAM1 (Figure 3i). Interestingly, all six of these belong to the RING family of E3 ligases. While all six of these RING E3 ligases are potentially involved in the mechanism underlying degradation, we sought to investigate the interactions of our fumarate handle with the most significantly enriched E3 ligase RNF126, which had already been a hit in the isoDTB-ABPP experiment.

To further characterize the binding of JP-2-196 to RNF126, we recorded the ^1^H-1D and ^1^H,^15^N-HMQC spectra of the uniformly ^15^N-enriched first 40 amino acids of RNF126 (RNF126(1–40)) in the absence and presence of JP-2-196. The apo spectrum of RNF126 showed the expected number of peaks. Signals were dispersed, with some peaks located downfield of 8.5 ppm, indicating the presence of a well-behaved and folded protein (Figure S5a; black peaks). While our spectrum was not entirely identical to a previously recorded spectrum due to the usage of different buffer conditions, we could transfer most of the published peak assignments with high confidence.^29^ Upon addition of JP-2-196 to apo-RNF126, we observed several residue-dependent chemical shift perturbations (CSPs), whereas the spectral dispersion and the overall signal intensities were unchanged (Figure S5a; red peaks). These results strongly suggested that our fumarate handle interacted with the protein without altering its fold or stability. Upon qualitatively clustering and mapping all spectral changes—CSPs and line broadening—onto a recently disclosed three-dimensional structure of RNF126(1–40),^29^ we found that the ligand specifically bound at or close to the zinc binding site and that the rest of the protein remained unaffected (Figure S5b).

### Transplanting a Fumarate-Based Covalent Chemical Handle onto Protein-Targeting Ligands That Already Possess Piperazines or Morpholines at the Exit Vector

**Figure 4:**
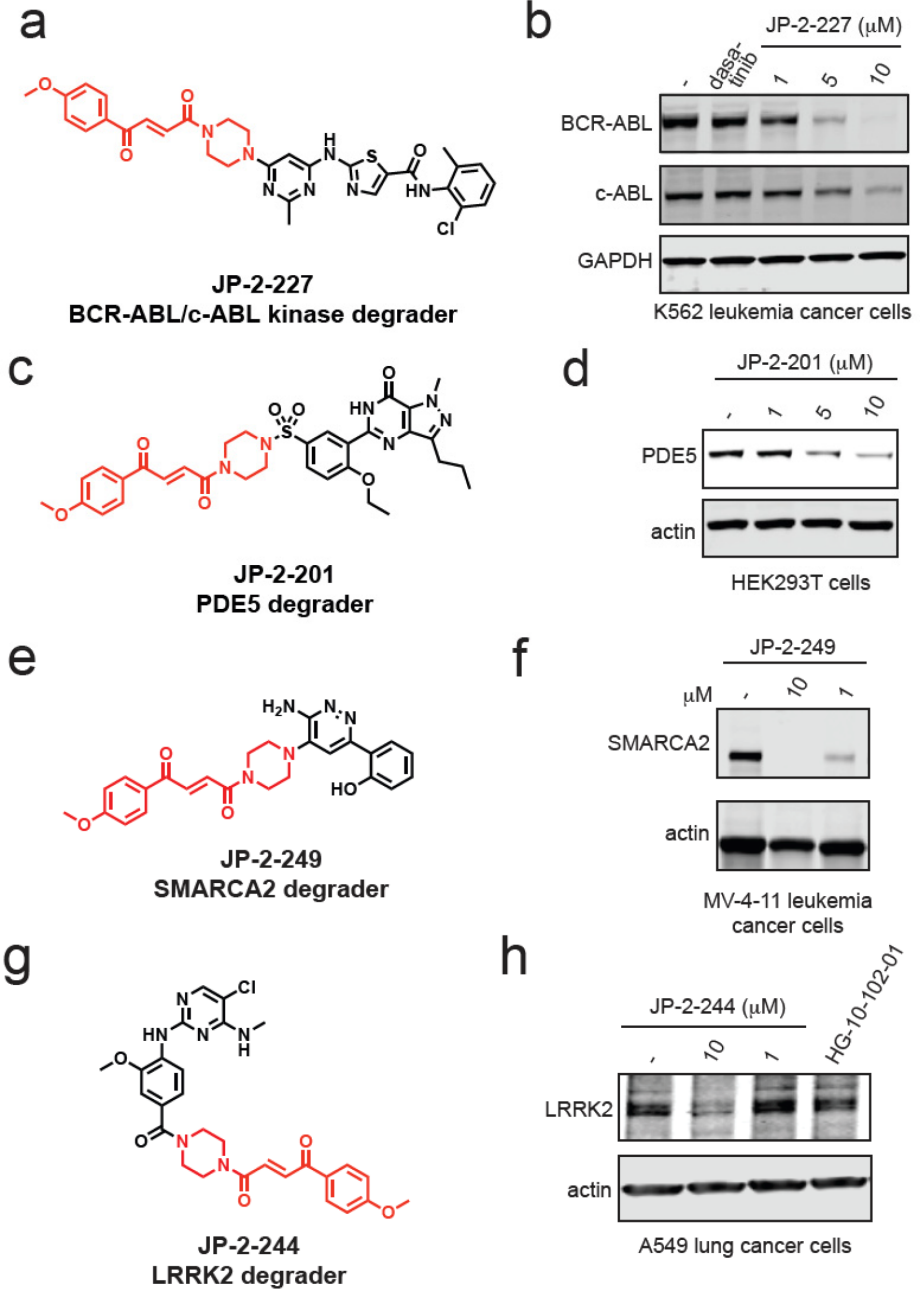
Transplanting a covalent chemical handle onto protein-targeting ligands that already possess piperazines at the exit vector. (a) Structure of JP-2-227 with the optimized covalent handle shown in red that was appended onto the BCR-ABL and c-ABL kinase inhibitor dasatinib. (b) JP-2-227 degrades BCR-ABL and c-ABL in K562 leukemia cancer cells. K562 cells were treated with DMSO vehicle or JP-2-227 for 24 h, and BCR-ABL, c-ABL, and loading control GAPDH levels were assessed by Western blotting. (c) Structure of JP-2-201 with the optimized covalent handle shown in red that was appended onto the PDE5 inhibitor sildenafil. (d) JP-2-201 degrades PDE5 in HEK293T cells. HEK293T cells were treated with DMSO vehicle or JP-2-201 for 24 h, and PDE5 and loading control actin levels were assessed by Western blotting. (e) Structure of SMARCA2 degrader JP-2-249 consisting of the optimized covalent handle incorporated into a previously reported protein-targeting ligand for SMARCA2. (f) MV-4-11 leukemia cancer cells were treated with DMSO vehicle or JP-2-249 for 24 h, and SMARCA2 and actin loading control levels were assessed by Western blotting. (g) Structure of LRRK2 degrader JP-2-244 consisting of the optimized covalent handle incorporated into a previously reported LRRK2 inhibitor. (h) A549 lung cancer cells were treated with DMSO vehicle or JP-2-244 for 24 h, and LRRK2 and actin loading control levels were assessed by Western blotting. Blots in (b,d,f,h) are representative of *n* = 3 biologically independent replicates/group.

Having identified a minimal motif required to potently engage RNF126, we next sought to transplant this handle onto other protein-targeting ligands to test if this module could be widely used to degrade the ligands’ respective protein targets. We first focused on ligands that, like ribociclib, already possessed piperazine moieties at the solvent-exposed exit vector. Incorporation of our fumarate motif onto the clinically approved BCR-ABL and c-ABL kinase inhibitor dasatinib, the phosphodiesterase 5 (PDE5) inhibitor sildenafil, the SMARCA2-bromodain ligand 1 used in a previously developed SMARCA2 PROTAC ABCI1 developed by the Ciulli group,^32^ and the LRRK2 inhibitor HG-10-102-01 that is currently under evaluation for Parkinson’s disease that bears a morpholine exit vector led to the generation of JP-2-227, JP-2-201, JP-2-249, and JP-2-244. All four of these derivatives showed potent labeling of RNF126 and led to the degradation of their targets BCR-ABL and c-ABL, PDE5, SMARCA2 and SMARCA4, and LRRK2, respectively (Figures 4a–h and S6a–j). Collectively, our data demonstrated that this fumarate chemical handle could be used to extend several protein-targeting ligands already bearing piperazine exit vectors to degrade their respective targets across several protein classes from kinases, phosphodiesterases, and transcriptional activators.

### Transplanting a Covalent Chemical Handle onto Protein-Targeting Ligands from Unrelated Chemical and Protein Classes

**Figure 5:**
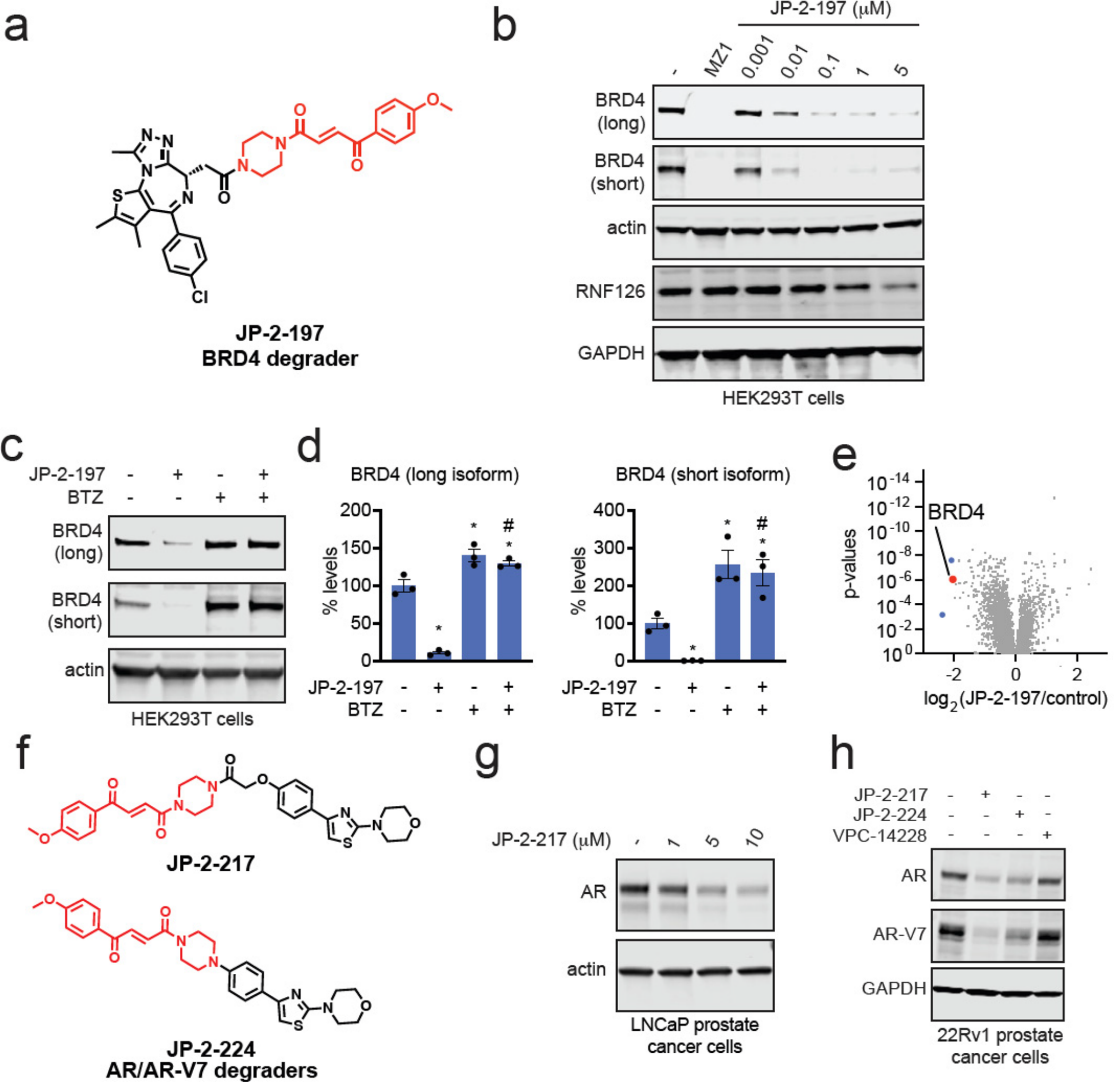
Transplanting a covalent chemical handle onto BET family inhibitor JQ1 and AR/AR-V7 targeting ligand to degrade BRD4 and AR/AR-V7. (a) Structure of JP-2-197 with the optimized covalent handle shown in red that was appended onto the BET family bromodomain inhibitor JQ1. (b) JP-2-197 degrades BRD4 in HEK293T cells. HEK293T cells were treated with DMSO vehicle, positive control BRD4 PROTAC MZ1 (1 μM), or JP-2-197 for 24 h. BRD4 long and short isoforms, RNF126, and loading controls actin and GAPDH levels were assessed by Western blotting. (c) Proteasome-dependent degradation of BRD4 by JP-2-197. HEK293T cells were pretreated with DMSO vehicle or the proteasome inhibitor BTZ (10 μM) for 1 h prior to treatment of cells with DMSO vehicle or JP-2-197 (1 μM), and BRD4 and loading control actin levels were assessed by Western blotting. (d) Quantification of the experiment described in (c). (e) TMT-based quantitative proteomic profiling of JP-2-197 in HEK293T cells. HEK293T cells were treated with DMSO vehicle or JP-2-197 (1 μM) for 24 h. Data are from *n* = 2 biological replicates per group. (f) Structures of two AR-V7 degraders consisting of the fumarate handle linked to an AR DNA-binding domain ligand VPC-14228–JP-2-217 and JP-2-224. (g) LNCaP prostate cancer cells were treated with JP-2-217 for 24 h, and AR and loading control actin levels were detected by Western blotting. (h) 22Rv1 prostate cancer cells were treated with DMSO vehicle, JP-2-217, JP-2-224, or VPC-14228 (10 μM) for 24 h, and AR, AR-V7, and loading control GAPDH levels were assessed by Western blotting. Blots and gels shown in (b,c,g,h) are representative images from *n* = 3 biologically independent replicates. Bar graphs in (d) show individual replicate values and average ± sem. Statistical significance is calculated as *p < 0.05 compared to DMSO vehicle and #*p* < 0.05 compared to cells treated with JP-2-197 alone.

We next sought to transplant this handle onto protein-targeting ligands that did not already possess a piperazine toward the exit vector of the compound. We first incorporated the JP-2-196 handle onto the BET family bromodomain inhibitor JQ1, forming JP-2-197 (Figure 5a). This compound still potently labeled pure RNF126 protein and led to highly potent midnanomolar degradation of both long and short isoforms of BRD4 in HEK293T cells in a dose-responsive, time-dependent, and proteasome-dependent manner (Figures 5b–d and S7a–c). JQ1 also inhibits other BET family proteins; however, we did not observe degradation of BRD2 or BRD3 (Figure S7d,e).^33^ Similar to our observation of RNF126 loss with EST1027, we also observed RNF126 loss at the highest concentration of JP-2-197 (Figure 5b). Pretreatment of cells with excess JQ1 completely attenuated JP-2-197-mediated BRD4 degradation (Figure S7f). Quantitative proteomic profiling of JP-2-197 also demonstrated selective degradation of BRD4 with two other targets also showing >4-fold reduced protein levels across >5000 proteins quantified (Figure 5e and Table S4). A nonreactive derivative of JP-2-197, JP-2-232, still showed binding to RNF126, albeit less potently compared to JP-2-197, but did not result in BRD4 degradation (Figure S7g–i). The chemical handle JP-2-196 itself does not alter BRD4 levels compared to the BRD4 degrader JP-2-197 (Figure S7i).

Next, we studied the interaction of JP-2-197 with RNF126(1–40) and BRD4(44–168) by proton-observed NMR. Our BRD4 construct showed two heavily upfield shifted methyl signals that were well-resolved (Figure S8a; black spectrum). Upon mixing BRD4(44–168) with JP-2-197, we observed strong signal perturbations of one of the methyl peaks (δ ≈ −0.19 ppm) and the appearance of new resonances (red spectrum) (Figure S8a). BRD4 remained soluble and folded when JP-2-197 was bound as indicated by an almost unchanged signal intensity of the unperturbed methyl resonance (δ ≈ −0.46 ppm) and the unaffected overall methyl group signal dispersion (Figure S8a). Combining RNF126(1–40), BRD4(44–168), and JP-2-197 together resulted in a ^1^H-1D spectrum with highly similar perturbations but with small additional chemical shift changes (e.g., for the peak at −0.38 ppm) and clear line broadening (green spectrum) (Figure S8a). We did not detect any comparable changes when the two proteins were combined without JP-2-197 (blue spectrum) or when treated with JQ1 or the fumarate handle JP-2-196 alone (magenta spectrum) (Figure S8a). Overall, these results support our premise that JP-2-197 induced a ternary complex formation between BRD4 and RNF126.

We generated a derivative of JP-2-197 that replaced its piperazine moiety with an ethyl diamine linker, JP-2-219 (Figure S8b). JP-2-219 was still able to degrade BRD4 but substantially less potently compared to JP-2-197, again demonstrating a tunable SAR for these covalent glue degraders (Figure S8c). We next assessed whether the maleic *Z*-isomer of our covalent handle was still capable of binding to RNF126 and whether this *cis*-isomer, when appended to JQ1, would affect BRD4 degradation. We found that both the *trans*- and *cis-*isomers of our covalent handle, both as *tert*-butyloxycarbonyl-protected handles—JP-2-190 and LE-21-PX17, respectively—were capable of binding to RNF126 (Figure S9a,b). We further demonstrated that the *cis*-isomer maleic handle appended onto JQ1 also potently bound to RNF126 and degraded BRD4 with equivalent potency compared to its *trans*-isomer JP-2-197 (Figure S9c–e).

BRD4 is a protein which is relatively easy to degrade and has been used as a test case for many different types of degraders.^27,34−36^ Thus, we next sought to expand the scope of ligand and target classes to understand the diversity of targets that our chemical handle could access for targeted protein degradation applications. First, we incorporated our fumarate handle into the pan-histone deacetylase (HDAC) inhibitor vorinostat to generate DD-1-073 (Figure S10a). We observed potent binding to RNF126 and degradation of HDAC1 and HDAC3 but not HDAC2 or HDAC6 in MDA-MB-231 cells (Figure S10b,c). Next, we incorporated our fumarate handle into the BTK inhibitor ibrutinib, replacing the BTK C481-targeting cysteine-reactive acrylamide warhead to generate JP-2-247 (Figure S10d). This molecule still potently labeled RNF126 and also showed BTK degradation in MINO lymphoma cancer cells (Figure S10e–g).

We next tackled a more challenging target, the truncated and constitutively active mutant of androgen receptor (AR), AR-V7, that drives the pathogenesis of androgen-resistant prostate cancers.^37^ AR-V7 is a relatively undruggable target, given that the ligand binding domain that is the target of most AR-targeting drugs is missing from AR-V7. We linked our fumarate derivative JP-2-196 onto a previously discovered DNA-binding domain ligand, VPC-14228, for the androgen receptor that had recently been used in several VHL-based PROTACs to degrade AR-V7, through two different types of linkages to yield JP-2-217 and JP-2-224 (Figure 5f).^38−40^ Both compounds showed potent binding to pure RNF126 protein (Figure S11a,b). Given that VPC-14228 binds to the DNA binding domain shared between wild-type full-length AR as well as its truncation mutants, we first tested this degrader for wild-type AR degradation in AR-sensitive LNCaP prostate cancer cells. JP-2-217 degraded AR in LNCaP cells in a dose-responsive manner (Figures 5g and S11c). We next tested both degraders in the androgen-resistant prostate cancer cell line 22Rv1 that expresses wild-type AR and AR-V7 and demonstrated that both JP-2-217 and JP-2-224, but not VPC-14228 or a previously reported VHL-based AR-V7 PROTAC (compound 6),^40^ degraded both wild-type AR and AR-V7 (Figures 5h and S11e–h). Overall, we demonstrated that the minimal covalent handle JP-2-196 could be used to convert protein-targeting ligands into degraders of several proteins from different protein classes.

## Discussion

While our chemical handle likely still requires significant optimization to improve potency, selectivity, and its versatility as a general motif for degrader design, we demonstrated proof-of-concept to convert protein-targeting ligands in a more rational manner into monovalent degraders of their targets without the need for long linkers and resultant high molecular weight PROTACs. We note that the molecular weights of all our degraders are lower than traditional PROTAC molecules, which may ease the burden of future medicinal chemistry efforts to generate compounds that show optimal pharmacokinetic parameters.

There are still many open questions that we hope to address in future studies. These questions include further understanding the mechanism underlying the versatile degradation observed with our chemical motif once appended to numerous ligands across so many different protein and ligand classes, the contribution of RNF126 and the role of BAG6 in RNF126-mediated responses, and the likely contribution of additional components of the ubiquitin-proteasome system beyond RNF126. We would also like to determine in future studies whether we are disrupting RNF126 endogenous function and whether this may pose any toxicity. While we were able to obtain a sufficient proteome to perform the described studies at the 24 h time points, many of the degraders reported here showed differing degrees of cytotoxicity at 24 h. We note though that many of the covalent derivatives that we generated in our original SAR showed similar cytotoxicity, but differential CDK4 degradation, supporting the premise that the degradation we observe is not through nonspecific toxicity-mediated mechanisms. Given that JP-2-196 targets a zinc-coordinating cysteine within RNF126 that is also found across many other RING E3 ligases, we conjecture that as we optimized for RNF126 binding, we likely also optimized binding toward similar motifs that may be conserved across other RING E3 ligases. This is supported by our chemoproteomic data demonstrating that our covalent handle also engages additional RING E3 ligases RNF40, MID2, RNF219, RNF14, and LRSAM1. We believe that these E3 ligases, alongside RNF126, may also play a role in the mechanism underlying target degradation observed in our study. Additional areas of investigation will be to further optimize the potency and selectivity while reducing the reactivity of our covalent handles so that this strategy can be more broadly applied in future drug discovery applications.

Overall, our study identifies a potential starting point for developing chemical rational design principles for converting protein-targeting ligands into monovalent molecular glue degraders through appending a minimal linker-less covalent handle that can recruit RNF126 and additional RING E3 ligases. We recognize that with the optimization of the RNF126-recognizing ligand, our resulting covalent handle linked to protein-targeting ligands could also be considered linker-less PROTACs, but we anticipate that as molecular glue and PROTAC design evolves, the definition between PROTACs and molecular glue degraders will likely begin to merge and that our study represents a step toward that direction.

## Supporting Information Available

Supporting methods, supporting references, Figures S1–S11, supporting table legends 1–4, and synthetic methods and characterization (PDF)
Table S1. TMT-based quantitative proteomic profiling of EST1027 in C33A cells (XLSX)
Table S2. Cysteine chemoproteomic profiling of EST1027 in C33A cells using isoDTB-ABPP (XLSX)
Table S3. Quantitative proteomics of JP-2-196-alkyne pulldown in HEK293T cells (XLSX)
Table S4. TMT-based quantitative proteomic profiling of JP-2-197 in HEK293T cells (XLSX)
