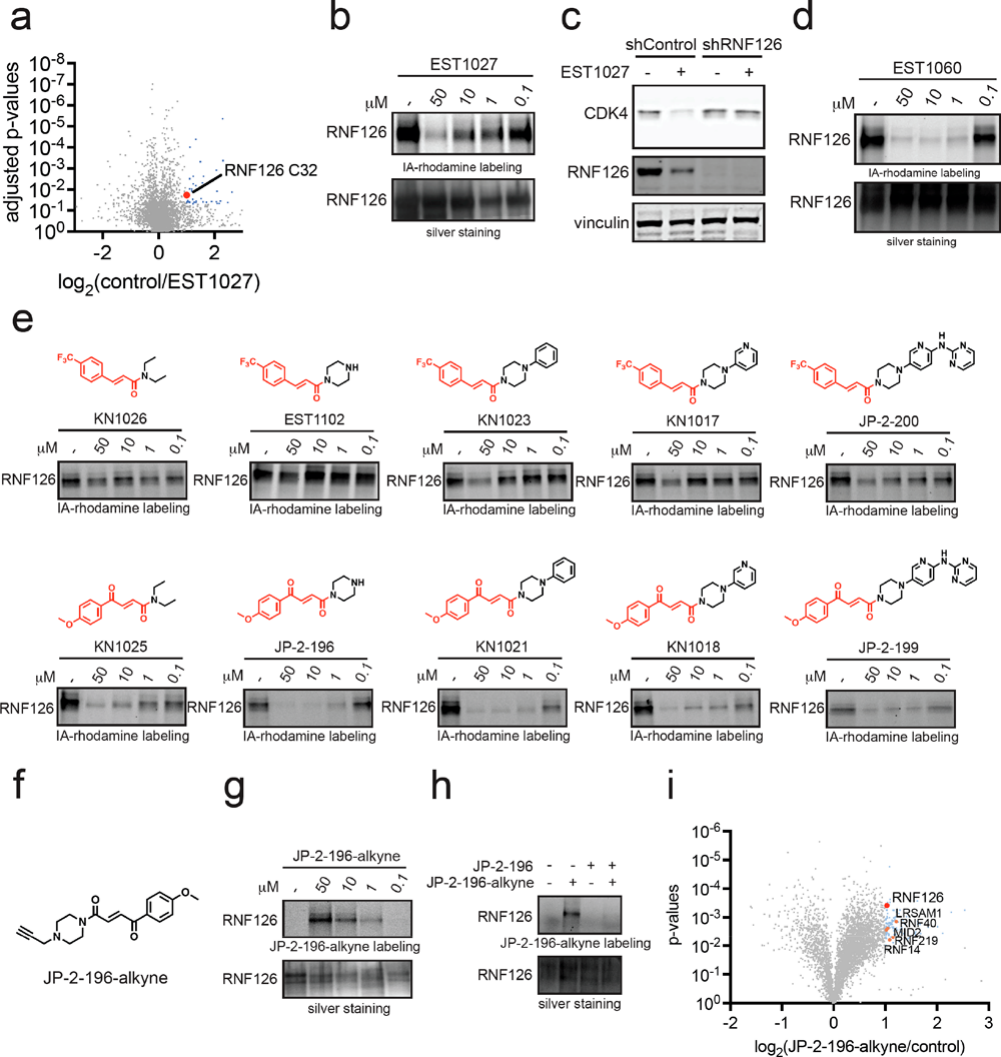# Correction to “Rational Chemical Design of Molecular Glue Degraders”

**DOI:** 10.1021/acscentsci.3c00844

**Published:** 2023-07-21

**Authors:** Ethan S. Toriki, James W. Papatzimas, Kaila Nishikawa, Dustin Dovala, Andreas O. Frank, Matthew J. Hesse, Daniela Dankova, Jae-Geun Song, Megan Bruce-Smythe, Heidi Struble, Francisco J. Garcia, Scott M. Brittain, Andrew C. Kile, Lynn M. McGregor, Jeffrey M. McKenna, John A. Tallarico, Markus Schirle, Daniel K. Nomura

We realized after publication that there was an error in Figure 3e in the structure of JP-2-200 where the hydrogen atom from the amine in JP-2-200 was far above the structure in the figure. We have corrected Figure 3 here.