# Supplementary material for: Rational Chemical Design of Molecular Glue Degraders
Source: ACS Cent Sci. 2023 Apr 11;9(5):915–26. doi: 10.1021/acscentsci.2c01317 (PMC10214506; doi:10.1021/acscentsci.2c01317)

Supporting Information

Rational Chemical Design of Molecular Glue Degraders

Ethan S. Toriki[1,2,3, #], James W. Papatzimas[1,2,3, #], Kaila Nishikawa[1,2,3], Dustin Dovala[2,4], Andreas O. Frank[2,4], Matthew J. Hesse[2,4], Daniela Dankova[1,2,3], Jae-Geun Song[2,4], Megan Bruce-Smythe[2,4], Heidi Struble[2,4], Francisco J. Garcia[2,5], Scott M. Brittain[2,5], Andrew C. Kile[2,4], Lynn M. McGregor[2,5], Jeffrey M. McKenna[2,5], John A. Tallarico[2,5], Markus Schirle[2,5], and Daniel K. Nomura[1,2,3,6] *

[1] Department of Chemistry, University of California, Berkeley, Berkeley, CA 94720 USA

[2] Novartis-Berkeley Translational Chemical Biology Institute

[3] Innovative Genomics Institute, Berkeley, CA 94704 USA.

[4] Novartis Institutes for BioMedical Research, Emeryville, CA 94608 USA

[5] Novartis Institutes for BioMedical Research, Cambridge, MA 02139 USA

[6] Department of Molecular and Cell Biology, University of California, Berkeley, Berkeley, CA 94720 USA

# co-first authorship

*correspondence to email: dnomura@berkeley.edu

Supporting Methods

Cell Culture

C33A cells were purchased from the American Type Culture Collection (ATCC) and were cultured in Dulbecco's Modified Eagle Medium (DMEM) containing 10% (v/v) fetal bovine serum (FBS) and maintained at 37 °C with 5% CO_2. 22RV1 cells were purchased from the ATCC and were cultured in RPMI-1640 Medium containing 10% (v/v) FBS and maintained at 37 °C with 5% CO_2**.** HEK293T cells were obtained from the UC Berkeley Cell Culture Facility and were cultured in DMEM containing 10% (v/v) FBS and maintained at 37 °C with 5% CO_2. K562 cells were obtained from the UC Berkeley Cell Culture Facility and were cultured in Iscove's Modified Dulbecco's Medium (IMDM) containing 10% (v/v) FBS and maintained at 37 °C with 5% CO_2. MV-4-11 cells were obtained from the ATCC and were cultured in IMDM containing 10% (v/v) FBS and maintained at 37 °C with 5% CO_2. A549 cells were obtained from the ATCC and were cultured in F-12K Medium containing 10% (v/v) FBS and maintained at 37 °C with 5% CO_2. Mino cells were obtained from the ATCC and were cultured in RPMI-1640 Medium containing 10% (v/v) FBS and maintained at 37 °C with 5% CO_2. LNCaP cells were obtained from the UC Berkeley Cell Culture Facility and were cultured in RPMI-1640 Medium containing 10% (v/v) FBS and maintained at 37 °C with 5% CO_2. Unless otherwise specified, all cell culture materials were purchased from Gibco. It is not known whether HEK293T cells are from male or female origin.

Expression and purification of recombinant RNF126 protein

RNF126 mammalian expression plasmid with a C-terminal FLAG tag was purchased from Origene (Origene Technologies Inc., RC204986). The plasmid was transformed into NEB 5-alpha Competent E. coli (DH5α) cells (NEB product no. C2987H). The following day, a single transformed colony was used to inoculate 50 ml of nutrient rich LB medium containing kanamycin (50 µg/mL) and was incubated at 37 °C overnight, with agitation (250 rpm). A Miniprep (Qiagen) kit was used to isolate the plasmid before sequence verification with appropriate primers.

HEK293T cells were grown to 30-50% confluency in DMEM supplemented with 10% FBS (Corning) and maintained at 37 °C with 5% CO_2. Immediately before transfection, media was replaced with DMEM

containing 10% FBS. Each plate was transfected with 24 µg of overexpression plasmid with 24 µL Lipofectamine 2000 (Invitrogen) in Opti-MEM. After 48 h cells were collected in PBS, lysed by sonication, and batch bound with anti-DYKDDDDK resin (GenScript, L00432) for 2 hours. Lysate and resin were washed with PBS and eluted with 133.33 µg/mL 3X FLAG peptide (ApexBio, A6001) in PBS. Five elutions were performed for 15 minutes each. Elutions were concentrated and the protein was stored in PBS. Concentration and purity was determined using the BCA assay and Western blotting.

Preparation of Cell Lysates

Cells were washed twice with cold PBS, scraped, and pelleted by centrifugation (700 g, 5 min, 4 °C). Pellets were resuspended in PBS, lysed by sonication or RIPA lysis buffer (Thermo Scientific), clarified by centrifugation (12,000 g, 10 min, 4 °C), and lysate was transferred to new low-adhesion microcentrifuge tubes. Proteome concentrations were determined using the BCA assay and lysate was diluted to appropriate working concentrations.

Western Blotting

Proteins were resolved by SDS/PAGE and transferred to nitrocellulose membranes using the Trans-Blot Turbo transfer system (Bio-Rad). Membranes were blocked with 5% BSA in Tris-buffered saline containing Tween 20 (TBS-T) solution for 1 hr at RT, washed in TBS-T, and probed with primary antibody diluted in recommended diluent per manufacturer overnight at 4 °C. After 3 washes with TBS-T, the membranes were incubated in the dark with IR680- or IR800-conjugated secondary antibodies at 1:10,000 dilution in 5 % BSA in TBS-T at RT for 1 h. After 3 additional washes with TBST, blots were visualized using an Odyssey Li-Cor fluorescent scanner. The membranes were stripped using ReBlot Plus Strong Antibody Stripping Solution (EMD Millipore, 2504) when additional primary antibody incubations were performed. Antibodies used in this study were CDK4 (Abcam ab108357), CDK6 (Cell Signaling Technology DCS83), Vinculin (Abcam ab129002), GAPDH (Cell Signaling Technology 14C10), RNF126 (Santa Cruz Biotechnology sc-376005), BRD4 (Abcam ab128874), BRD2 (Cell Signaling Technology D89B4), BRD3 (Abcam ab300106), Beta Actin (Cell Signaling Technology 13E5), PDE5 (Abcam ab259945), AR-V7 (Abcam ab273500), c-Abl (Santa Cruz Biotechnology sc-23), SMARCA2 (Abcam ab240648), BRG1 (SMARCA4) (Cell Signaling Technology D1Q7F), LRRK2 (Abcam

ab133474), BTK (Cell Signaling Technology D3H5), Androgen Receptor (Cell Signaling Technology D6F11), HDAC1 (Cell Signaling Technology 10E2), HDAC2 (Cell Signaling Technology 3F3), HDAC3 (Cell Signaling Technology 7G6C5), and HDAC6 (Cell Signaling Technology D2E5).

Knockdown studies

Short-hairpin oligonucleotides were used to knock down the expression of RNF126 in C33A cells. For lentivirus production, lentiviral plasmids and packaging plasmids (pMD2.G, Addgene catalog no. 12259 and psPAX2, Addgene catalog no. 12260) were transfected into HEK293T cells using Lipofectamine 2000 (Invitrogen). Lentivirus was collected from filtered cultured medium and used to infect the target cell line with 1:1000 dilution of polybrene. Target cells were selected over 3 d with 1 μg/mL of puromycin for C33A cells. The short-hairpin sequences which were used for generation of the knockdown lines were:

RNF126: TGCCATCATCACACAGCTCCT (Sigma RNF126 MISSION shRNA Bacterial Glycerol Stock, TRCN0000368954). MISSION TRC1.5 pLKO.1- or TRC2 pLKO.5-puro Non-Mammalian shRNA Control (Sigma) was used as a control shRNA.

IsoDTB-ABPP Cysteine Chemoproteomic Profiling of EST1027

C33A cells were treated with either EST1027 (20 μM) or DMSO for 2 h before cell collection and lysis. The proteome concentrations were determined using BCA assay and adjusted to 2 mg/mL. For each biological replicate, 2 aliquots of 1 mL of 2 mg/mL were used (i.e. 4 mg per condition). Each aliquot was treated with 20 μL of IA-alkyne (26.6 mg/mL in DMSO, 200 μM final concentration) for 1 h at RT. Two master mixes of the click reagents were prepared in the meanwhile, each containing 510 μL TBTA (0.9 mg/mL in 4:1 tBuOH/DMSO), 165 μL $CuSO_4$ (12.5 mg/mL in H_2O), 165 μL TCEP (14.0 mg/mL in H_2O) and 160 μL of either heavy or light isoDTB tags (4 mg in DMSO, Click Chemistry Tools, 1565). The samples were then treated with 120 μL of the heavy (DMSO treated) or light (compound treated) master mix for 1 h at RT. After incubation, one light and one heavy-labeled samples were combined and acetone-precipitated overnight at -20 °C. The samples were then centrifuged at 3,500 rpm for 10 min, acetone was removed, and the protein pellets resuspended in cold MeOH by sonication. The samples were centrifuged at 3,500 rpm for 10 min and MeOH was removed (repeated 3× in

total). The pellets were dissolved in 600 µL urea (8M in 0.1 M TEAB) by sonication and the urea concentration was then adjusted to 2M by adding 1800 µL of TEAB (0.1 M). Two tubes containing solubilized proteins were combined, further diluted with 2400 µL 0.2% NP40 in PBS, and bound to high-capacity streptavidin agarose beads (200 µL/sample, ThermoFisher, 20357) for 1 h at RT with mixing. The beads were then centrifuged for 1 min at 1,000 g, the supernatant was removed, and the beads were washed 3 times with 0.1% NP40 in PBS, 3 times with PBS and 3 times with H_2O. The samples were then resuspended in 8M urea (600 µL in 0.1 M TEAB) and treated with DTT (30 µL, 31 mg/mL in H_2O) for 45 min at 37 °C. They were then reacted with iodoacetamide (30 µL, 74 mg/mL in H_2O) for 30 min at RT, followed by DTT (30 µL, 31 mg/mL in H_2O) for 30 min at RT. The samples were diluted with 1800 µL TEAB (0.1 M), centrifuged for 1 min at 1,000 g, and the supernatant was removed. The beads were resuspended in 400 µL urea (2M in 0.1 M TEAB), and trypsin (8 µL, 0.5 mg/mL) was added and incubated for 20 h at 37 °C. The samples were then diluted with 800 µL 0.1% NP40 in PBS and the beads were washed 3 times with 0.1% NP40 in PBS, 3 times with PBS, and 3 times with H_2O. Peptides were then eluted with 0.1% formic acid in 50% acetonitrile (3 × 400 µL). The samples were then dried using a vacuum concentrator at 30 °C, resuspended in 300 µL 0.1% TFA in H_2O, and fractionated using high pH reversed-phase peptide fractionation kits (ThermoFisher, 84868) according to the manufacturer's protocol.

IsoDTB-ABPP Mass Spectrometry Analysis

Mass spectrometry analysis was performed on an Orbitrap Eclipse Tribrid Mass Spectrometer with a High Field Asymmetric Waveform Ion Mobility (FAIMS Pro) Interface (Thermo Scientific) with an UltiMate 3000 Nano Flow Rapid Separation LCnano System (Thermo Scientific). Off-line fractionated samples (5 µl aliquot of 15 µl sample) were injected via an autosampler (Thermo Scientific) onto a 5 µl sample loop which was subsequently eluted onto an Acclaim PepMap 100 C18 HPLC column (75 µm x 50 cm, nanoViper). Peptides were separated at a flow rate of 0.3 µl/min using the following gradient: 2 % buffer B (100 % acetonitrile with 0.1 % formic acid) in buffer A (95:5 water:acetonitrile, 0.1 % formic acid) for 5 min, followed by a gradient from 2 to 40 % buffer B from 5 to 159 min, 40 to 95 % buffer B from 159 to 160 minutes, holding at 95 % B from 160-179 min, 95 % to 2 % buffer B from 179 to 180 min, and then 2 % buffer B from 180 to 200 min. Voltage applied to the nano-LC electrospray ionization source was 2.1 kV. Data was acquired through an MS1 master scan (Orbitrap analysis, resolution 120,000, 400-1800 m/z, RF lens 30 %, heated capillary temperature 250 °C) with dynamic exclusion

enabled (repeat count 1, duration 60 s). Data-dependent data acquisition comprised a full MS1 scan followed by sequential MS2 scans based on 2 s cycle times. FAIMS compensation voltages (CV) of -35, -45, and -55 were applied. MS2 analysis consisted of: quadrupole isolation window of 0.7 m/z of precursor ion followed by higher energy collision dissociation (HCD) energy of 38 % with a orbitrap resolution of 50,000.

Data were extracted in the form of MS1 and MS2 files using Raw Converter (Scripps Research Institute) and searched against the Uniprot human database using ProLuCID search methodology in IP2 v.3-v.5 (Integrated Proteomics Applications, Inc.) [1]. Cysteine residues were searched with a static modification for carboxyaminomethylation (+57.02146) and up to two differential modifications for methionine oxidation and either the light or heavy isoDTB tags (+561.33872 or +567.34621, respectively). Peptides were required to be fully tryptic peptides. ProLUCID data were filtered through DTASelect to achieve a peptide false-positive rate below 5%. Only those probe-modified peptides that were evident across two out of three biological replicates were interpreted for their isotopic light to heavy ratios. Light versus heavy isotopic probe-modified peptide ratios are calculated by taking the mean of the ratios of each replicate paired light versus heavy precursor abundance for all peptide-spectral matches associated with a peptide. The paired abundances were also used to calculate a paired sample *t*-test *P* value in an effort to estimate constancy in paired abundances and significance in change between treatment and control. *P* values were corrected using the Benjamini–Hochberg method.

Gel-Based ABPP

Recombinant RNF126 (0.1μg/sample) was pre-treated with either DMSO vehicle or covalent ligand at 37 °C for 30 min in 25 μL of PBS, and subsequently treated with of IA-Rhodamine (concentrations designated in figure legends) (Setareh Biotech) at room temperature for 1 h in the dark. The reaction was stopped by addition of 4×reducing Laemmli SDS sample loading buffer (Alfa Aesar). After boiling at 95 °C for 5 min, the samples were separated on precast 4−20% Criterion TGX gels (Bio-Rad). Probe-labeled proteins were analyzed by in-gel fluorescence using a ChemiDoc MP (Bio-Rad).

NMR Spectroscopy

We recorded all NMR spectra on Bruker Avance III 600 MHz (using 3 mm tubes filled with 160 µL sample) and Bruker Neo 600 MHz (using 1.7 mm tubes filled with 40 µL of sample) spectrometers, equipped with either a 5 mm QCI-F cryo probe with z-gradient or a 1.7 mm TCI cryo probe, and kept the temperature constant at 298K during our experiments. Standard Bruker ^{1}H-1D (with excitation sculpting water suppression) and ^{1}H,^{15}N-SOFAST-HMQC experiments were conducted while using traditional or 50% non-uniform sampling[2]. Our samples contained 50 µM or 100 µM human wt-(U}-^{15}N-RNF126(1-40), unlabeled 50 µM BRD4(44-168) (for protein-protein binding experiments only), 5% (3 mm tubes) or 10% (1.7 mm tubes) D_2O, 25 mM Hepes pH 7.5, 150 mM NaCl, 11.1 µM DSS, 2% d_6-DMSO, 0-50 µM d-TCEP, and 100 µM or 200 µM of compounds (for samples with compound; DMSO-matched). All spectra were recorded at two time points (incubation times of 30 minutes and 24 hours) to detect time-dependent ligand binding effects.

We assigned most peaks by transferring the published resonance assignments onto our amide spectrum [3]. To localize the ligand-interaction site, we clustered compound-induced chemical shift perturbations and line broadening effects in groups with very strong, strong, medium, weak and no residue-specific spectral changes. Clusters were subsequently mapped onto the three-dimensional structure of RNF126(1-40) (PDB code: 2N9O) [3].

Protein Expression for NMR Studies

Plasmid construct 8xHis-Thioredoxin-TEV-RNF126(1-40) was transformed into BL21(DE3) competent cells and grown overnight on LB-agar plates. A single colony was used to inoculate a 3 mL LB + kanamycin starter culture, which was grown shaking at 37 °C for 4-6 hours. For each liter of culture, 50 mL of expression media (per liter: 50 mL 20X M9 salts dissolved in sterile water, 2 mM $MgSO_4$, 1 mL vitamin solution [Sigma R7256], 0.1 mL 1000X trace elements [made in-house], 1 g ^{15}N-NH_4Cl, 3 g glucose, 50 mg kanamycin, 0.05 mM $ZnCl_2$, 0.25 g ISOGRO-^{15}N [Sigma 606871], sterile water to volume) was inoculated with 0.5 mL of the starter culture and grown overnight shaking at 37 °C. The 50 mL culture was used to inoculate 950 mL of expression media until the OD_{600} reached 0.7-0.8, at which point the culture was induced with 0.5 mM IPTG and the temperature reduced to 18 °C. The culture was grown overnight (~18 hours) and harvested via centrifugation. Pellets were stored at -80 °C.

A pET vector containing 6xHis-TEV-BRD4(44-168) was used to transform BL21(DE3) *E. coli* using standard techniques. 1 L cultures were grown in Terrific Broth supplemented with 50 mM Sodium Phosphate pH 7.0 and 50 µg/mL kanamycin. Cells were induced when the OD600 reached 1.0-1.5 and were left to continue to grow overnight at 19 °C. Cells were harvested by centrifugation and lysed via cell homogenizer (Lysis Buffer: 50 mM Tris pH 8.0, 400 mM NaCl, 1 mM TCEP).

Protein Purification for NMR Studies

RNF126(1-40) cell pellets were thawed and resuspended in cold lysis buffer (20 mM HEPES, pH 8.0, 500 mM NaCl, 30 mM Imidazole) at a ratio of 1 g cells to 5 mL buffer. Cells were disrupted via sonication and separated from the supernatant via centrifugation. 1 mL of loose Ni Sepharose 6 Fast Flow resin per 50 mL of supernatant was washed three times in lysis buffer, then incubated with the supernatant for 2-3 hours, rotating at 4 °C. The slurry was washed with lysis buffer in a drip column until the flow-though no longer contained protein (measured via NanoDrop). The protein was eluted in 10 x 4 mL steps with the imidazole concentration increasing by 30 mM each step. Cleavage with TEV protease was performed via overnight dialysis in a 1000 MWCO dialysis cassette against dialysis buffer (20 mM HEPES, pH 8.0, 500 mM NaCl) spinning at 4 °C. The protein solution was run through a HisTrap FF 5 mL on AKTA to separate the tag. 0.05 mM $ZnCl_2$ was added to the eluate which was concentrated using an Amicon 1K centrifugal filter. The sample was run on a Superdex 30 size exclusion column with SEC buffer (20 mM HEPES, pH 7.5, 150 mM NaCl). Protein yield was estimated to be between 0.5 and ~1 mg per liter according to a NanoDrop readout.

BRD4 cell lysate was clarified via centrifugation (45,000 x g for 30 minutes) prior to IMAC purification using 5 mL Ni-NTA bulk resin pre-equilibrated with Lysis Buffer. The resin was then washed with 5 CV of Lysis Buffer followed by Lysis Buffer supplemented with increasing concentrations of imidazole (20 mM and 40 mM). The protein was eluted with 5 CV of Elution Buffer (50 mM Tris pH 8.0, 400 mM NaCl, 1 mM TCEP, 500 mM imidazole) and subsequently treated with TEV protease while dialyzing against 3 L of Lysis Buffer at 4 °C overnight. Cleaved protein was subject to reverse IMAC purification with 5 mL of Ni-NTA bulk resin pre-equilibrated with Lysis Buffer. The flow-through was collected, concentrated, and subjected to size exclusion chromatography using a Superdex 200 16/60 column attached to an AKTA FPLC. The column was pre-equilibrated with SEC Buffer (25 mM HEPES pH 7.5, 150 mM NaCl, 1 mM TCEP). Fractions containing protein

were pooled, concentrated, and aliquoted. Correct mass was confirmed by ESI-LC/MS and purity was confirmed by SDS-PAGE.

JP-2-196-Alkyne Pulldown Quantitative Proteomics

Cells were treated with either DMSO vehicle or compound (JP-2-196-alkyne, 10 µM) for 6 h. Cells were harvested and lysed by probe sonication in PBS. The proteome concentrations were determined using the BCA assay and adjusted to 5 mg/mL in a volume of 500 µL PBS. For each biological replicate, 4 aliquots were used (i.e. 10 mg per condition). Copper-catalyzed azide-alkyne cycloaddition (CuAAC) was performed by sequential addition of 10 µL TCEP (14.4 mg/mL in H_2O), 30 µL TBTA (0.9 mg/mL in 4:1 tBuOH/DMSO), 10 µL $CuSO_4$ (12.5 mg/mL in H_2O) and 10 µL biotin picolyl azide (10 mM in DMSO). After 1 h, proteomes were precipitated by centrifugation at 6,500 g, washed in ice-cold methanol, combined to attain 10 mg per sample, washed again, then denatured and resolubilized by heating in 1.2% SDS–PBS (1 mL) to 90 °C for 5 min. The soluble proteome was diluted with 4 mL of PBS and labeled proteins were bound to high-capacity streptavidin-agarose beads (170 µL/sample, Thermo Fisher Scientific, 20357) while rotating overnight at 4 °C. Bead-linked proteins were enriched by washing once with 0.2% SDS-PBS and 3 times each in PBS and water, then resuspended in 6 M urea/PBS (500 µL), reduced in DTT (25 µL, 30 mg/mL in H_2O), and alkylated with iodoacetamide (74 mg/mL in H_2O), before being washed and resuspended in 50 mM Triethylammonium bicarbonate (TEAB) (100 µL) and trypsinized overnight with sequencing grade trypsin (4 µL, 0.5 mg/mL). Tryptic peptides were eluted off through centrifugation. Individual samples were then labeled with isobaric tags using commercially available TMTsixplex (Thermo Fisher Scientific, P/N 90061) kits, in accordance with the manufacturer's protocols. Tagged samples (20 µg per sample) were combined, dried using a vacuum concentrator at 30 °C, resuspended with 300 µL 0.1% TFA in H_2O, and fractionated using high pH reversed-phase peptide fractionation kits (Thermo Fisher Scientific, P/N 84868) according to the manufacturer's protocol. Fractions were dried with using a vacuum concentrator at 30 °C, resuspended with 50 µL 0.1% FA in H_2O, and analyzed by LC-MS/MS as described below.

Quantitative TMT-based proteomic analysis was performed as previously described using a Thermo Eclipse with FAIMS LC-MS/MS [4]. Acquired MS data was processed using ProLuCID search methodology in IP2 v.3-v.5 (Integrated Proteomics Applications, Inc.) [1]. Trypsin cleavage specificity (cleavage at K, R except if followed by P) allowed for up to 2 missed cleavages. Carbamidomethylation of cysteine was set as a fixed modification, methionine oxidation, and TMT-modification of N-termini and lysine residues were set as variable modifications. Reporter ion ratio calculations were performed using summed abundances with most confident centroid selected from 20 ppm window. Only peptide-to-spectrum matches that are unique assignments to a given identified protein within the total dataset are considered for protein quantitation. High confidence protein identifications were reported with a <1% false discovery rate (FDR) cut-off. Differential abundance significance was estimated using ANOVA with Benjamini-Hochberg correction to determine p-values.

Quantitative TMT Proteomics Analysis

Cells were treated with either DMSO vehicle or compound (EST1027, 5 μM, or JP-2-197, 1 μM) for 24 h and lysate was prepared as described above. Briefly, 25-100 μg protein from each sample was reduced, alkylated and tryptically digested overnight. Individual samples were then labeled with isobaric tags using commercially available TMTsixplex (Thermo Fisher Scientific, P/N 90061) kits, in accordance with the the manufacturer's protocols. Tagged samples (20 μg per sample) were combined, dried using a vacuum concentrator at 30 °C, resuspended with 300 μL 0.1% TFA in H2O, and fractionated using high pH reversed-phase peptide fractionation kits (Thermo Fisher Scientific, P/N 84868) according to the manufacturer's protocol. Fractions were dried using a vacuum concentrator at 30 °C, resuspended with 50 μL 0.1% FA in H_2O, and analyzed by LC-MS/MS as described below.

Quantitative TMT-based proteomic analysis was performed as previously described using a Thermo Eclipse with FAIMS LC-MS/MS [4]. Acquired MS data was processed using ProLuCID search methodology in IP2 v.3-v.5 (Integrated Proteomics Applications, Inc.) [1]. Trypsin cleavage specificity (cleavage at K, R except if followed by P) allowed for up to 2 missed cleavages. Carbamidomethylation of cysteine was set as a fixed modification, methionine oxidation, and TMT-modification of N-termini and lysine residues were set as variable modifications.

Reporter ion ratio calculations were performed using summed abundances with most confident centroid selected from 20 ppm window. Only peptide-to-spectrum matches that are unique assignments to a given identified protein within the total dataset are considered for protein quantitation. High confidence protein identifications were reported with a <1% false discovery rate (FDR) cut-off. Differential abundance significance was estimated using ANOVA with Benjamini-Hochberg correction to determine p-values.

Data Availability Statement

The datasets generated during and/or analyzed during the current study are available from the corresponding author on reasonable request.

Code Availability Statement

Data processing and statistical analysis algorithms from our lab can be found on our lab's Github site: https://github.com/NomuraRG, and we can make any further code from this study available at reasonable request.

Safety Statement

No unexpected or unusually high safety hazards were encountered.

Supporting References

1. Xu, T. *et al.* ProLuCID: An improved SEQUEST-like algorithm with enhanced sensitivity and specificity. *J. Proteomics* **129**, 16–24 (2015).
2. Schanda, P., Kupce, E. & Brutscher, B. SOFAST-HMQC experiments for recording two-dimensional heteronuclear correlation spectra of proteins within a few seconds. *J. Biomol. NMR* **33**, 199–211 (2005).
3. Krysztofinska, E. M. *et al.* Structural and functional insights into the E3 ligase, RNF126. *Sci. Rep.* **6**, 26433 (2016).
4. King, E. A. *et al.* Chemoproteomics-Enabled Discovery of a Covalent Molecular Glue Degrader Targeting NF-κB. 2022.05.18.492542 Preprint at https://doi.org/10.1101/2022.05.18.492542 (2022).

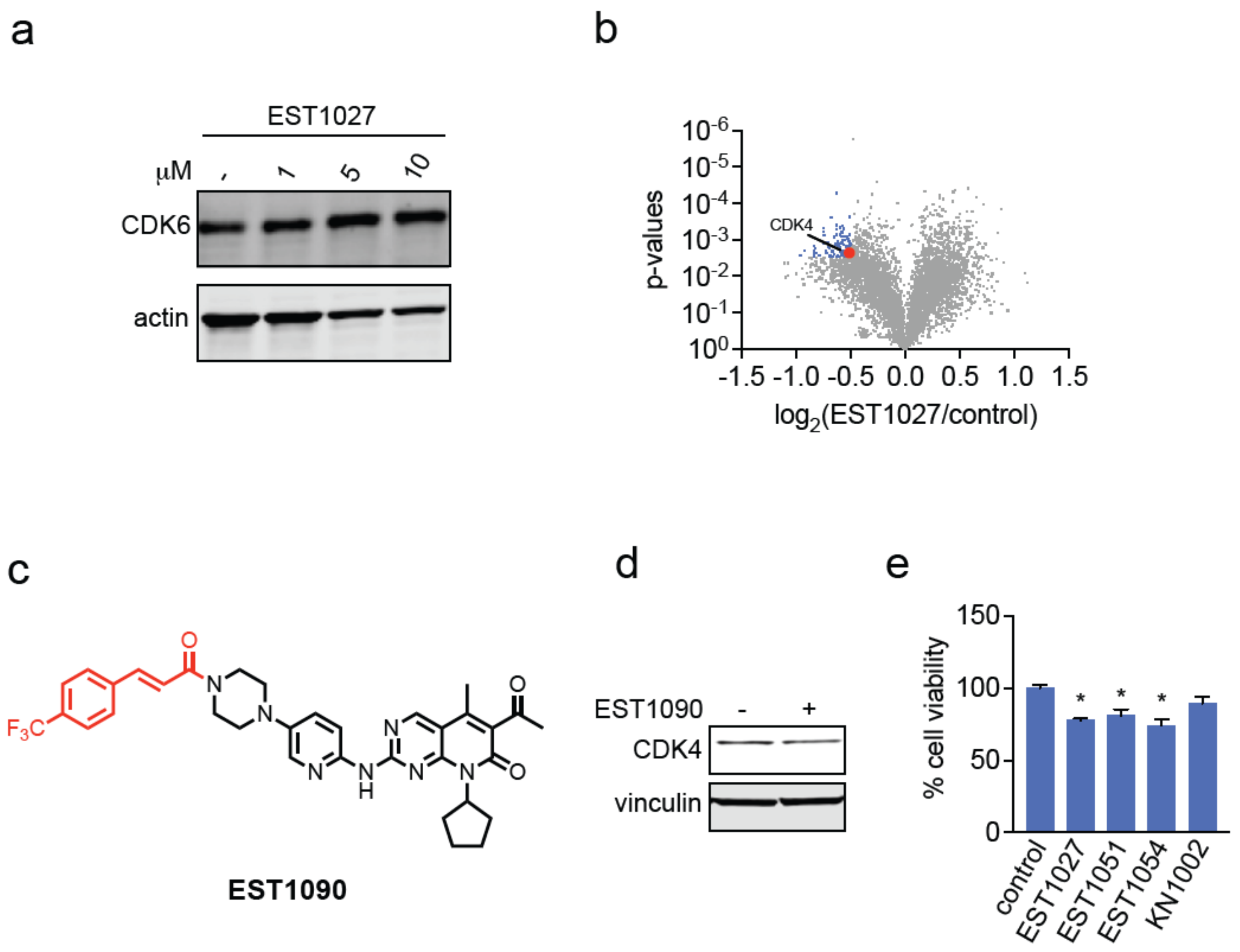

Figure S1. Palbociclib derivatives and their ability to degrade CDK4 and CDK6. **(a)** CDK6 levels in C33A cells. C33A cells were treated with DMSO vehicle or EST1027 for 24 h and CDK6 and loading control actin levels were detected by Western blotting. **(b)** TMT-based quantitative proteomic profiling of EST1027 in C33A cells. C33A cells were treated with DMSO vehicle or EST1027 (5 μM) for 24 h and cell lysates were subjected to TMT-based proteomic analyses. Shown are average EST1027/control TMT protein level ratios with points in blue or red noting proteins that showed ratios <0.7 and p<0.003 from n=3 biologically independent replicates/group. Shown in red is CDK4. **(c)** Structure of EST1090 with the trifluoromethyl propenamide handle linked to the CDK4/6 inhibitor Palbociclib. **(d)** CDK4 levels in C33A cells. C33A cells were treated with DMSO vehicle or EST1090 (10 μM) for 24 h and CDK4 and loading control vinculin levels were detected by Western blotting. **(e)** Cell viability assessed by Cell Titer Glo in C33A cells treated with DMSO vehicle or compounds (5 μM) for 24 h. Gels and blots in **(a, d)** are representative of n=3 biologically independent replicates/group. Bar graph in **(e)** show average ± sem. Statistical significance in **(e)** is calculated as *p<0.05 compared to DMSO vehicle.

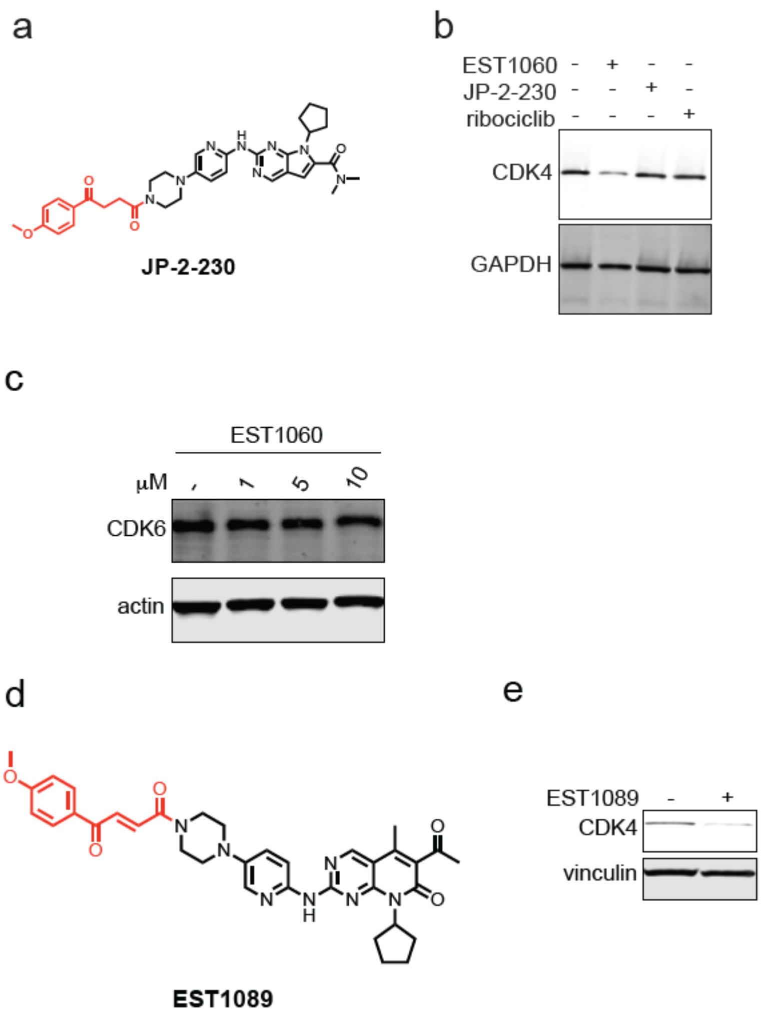

Figure S2. Ribociclib and Palbociclib derivatives and their ability to degrade CDK4 and CDK6. (a) Structure of JP-2-230 with non-reactive handle linked to the CDK4/6 inhibitor Ribociclib. **(b)** CDK4 levels in C33A cells. C33A cells were treated with DMSO vehicle, EST1060, or JP-2-230 (10 µM) for 24 h and CDK4 and loading control GAPDH levels were detected by Western blotting. **(c)** CDK6 levels in C33A cells. C33A cells were treated with DMSO vehicle or EST1060 for 24 h and CDK6 and loading control actin levels were assessed by Western blotting. **(d)** Structure of EST1089 with fumarate handle linked to the CDK4/6 inhibitor Palbociclib. **(e)** CDK4 levels in C33A cells. C33A cells were treated with DMSO vehicle or EST1089 (10 µM) for 24 h and CDK4 and loading control vinculin levels were detected by Western blotting. Gels and blots in **(b, c, e)** are representative of n=3 biologically independent replicates/group.

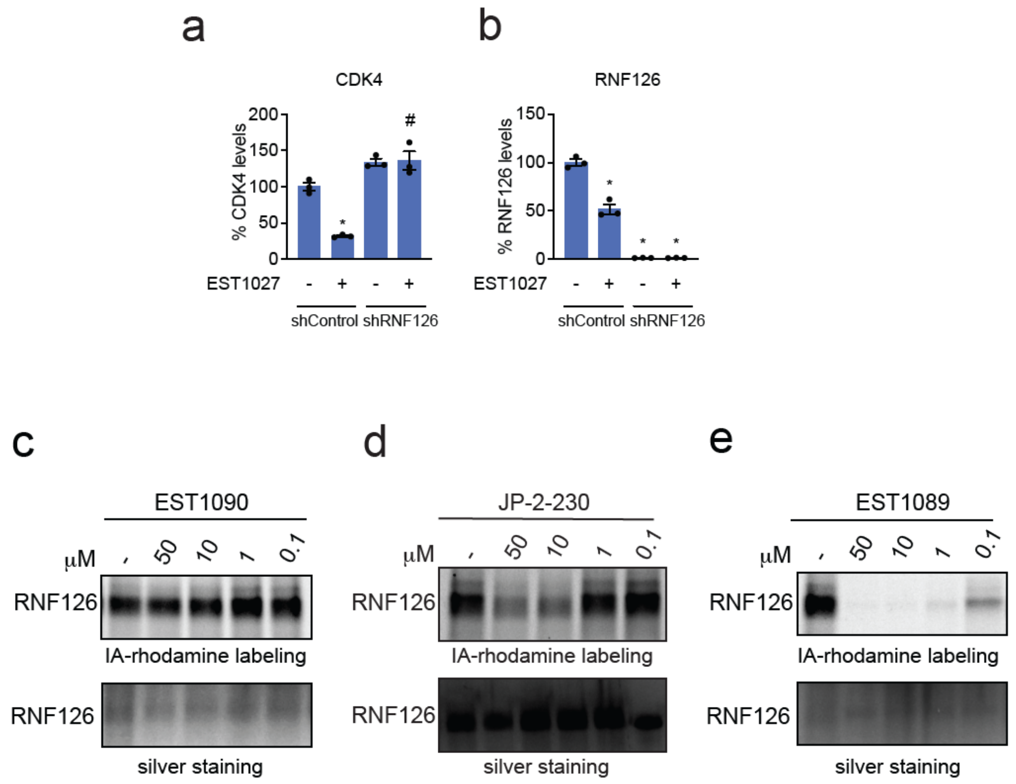

Figure S3. Characterization of CDK4 degraders. (a, b) Quantification of experiment described in **Figure 3c. (c, d, e)** Gel-based ABPP of EST1090, JP-2-230, EST1089 against RNF126. Recombinant RNF126 was pre-incubated with DMSO vehicle or compounds for 30 min prior to labeling of RNF126 with IA-rhodamine (250 nM) for 1 h. Gels were visualized by in-gel fluorescence and protein loading was assessed by silver staining. Shown are representative gels from n=3 biologically independent replicates/group. Bar graphs in **(a. b)** show individual replicate values and average ± sem. Statistical significance in **(a, b)** is calculated as *$p<0.05$ compared to DMSO vehicle and #$p<0.05$ compared to EST1027-treated group.

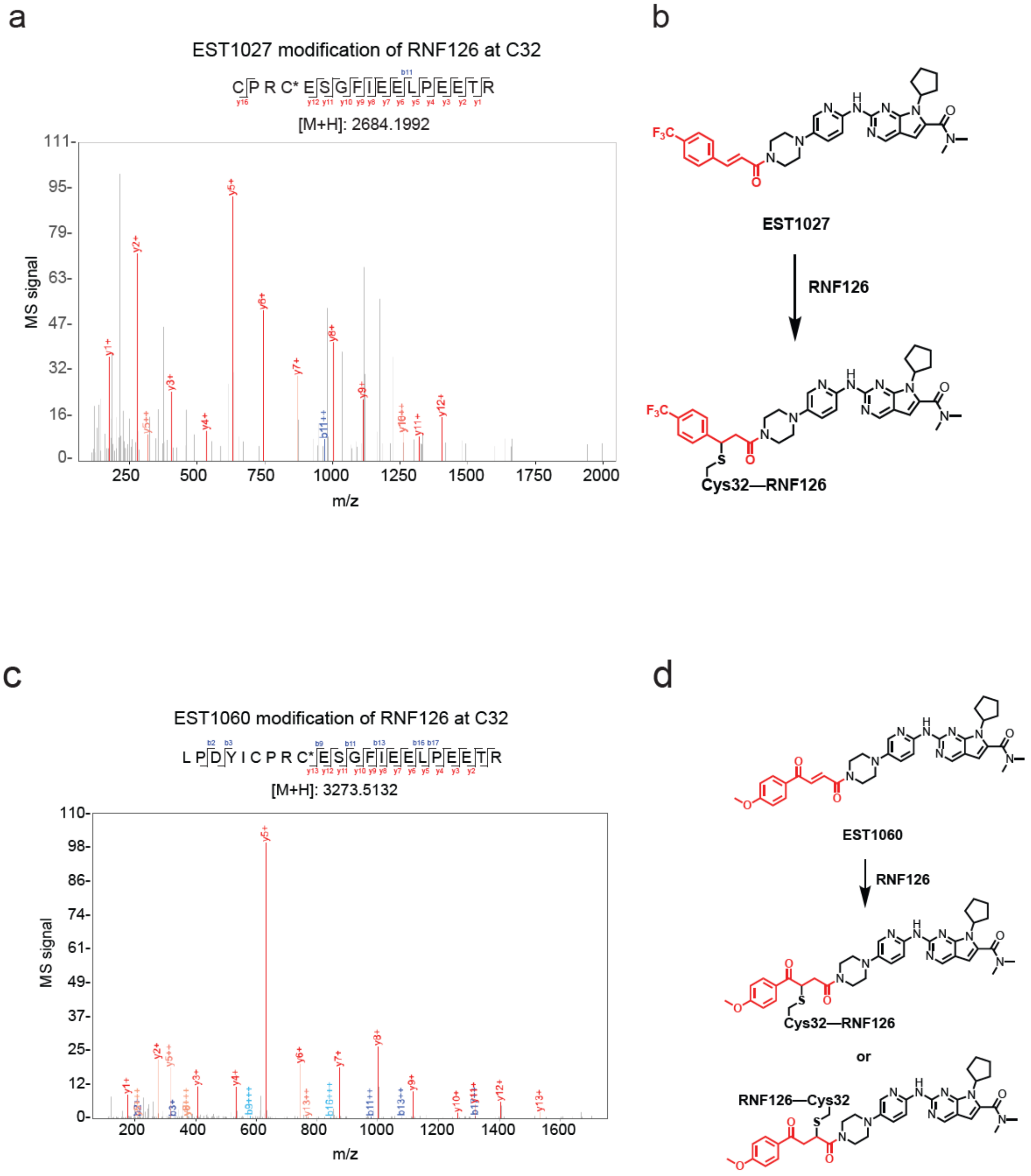

Figure S4. Site of modification analysis of EST1027 and EST1060 with pure RNF126 protein. RNF126 protein (24 μg) was incubated with EST1027 **(a, b)** or EST1060 **(c, d)** (50 μM) for 30 min and tryptic digests were analyzed by LC-MS/MS. **(b, d)** Structure of presumed adduct of EST1027 and EST1060 with C32 of RNF126.

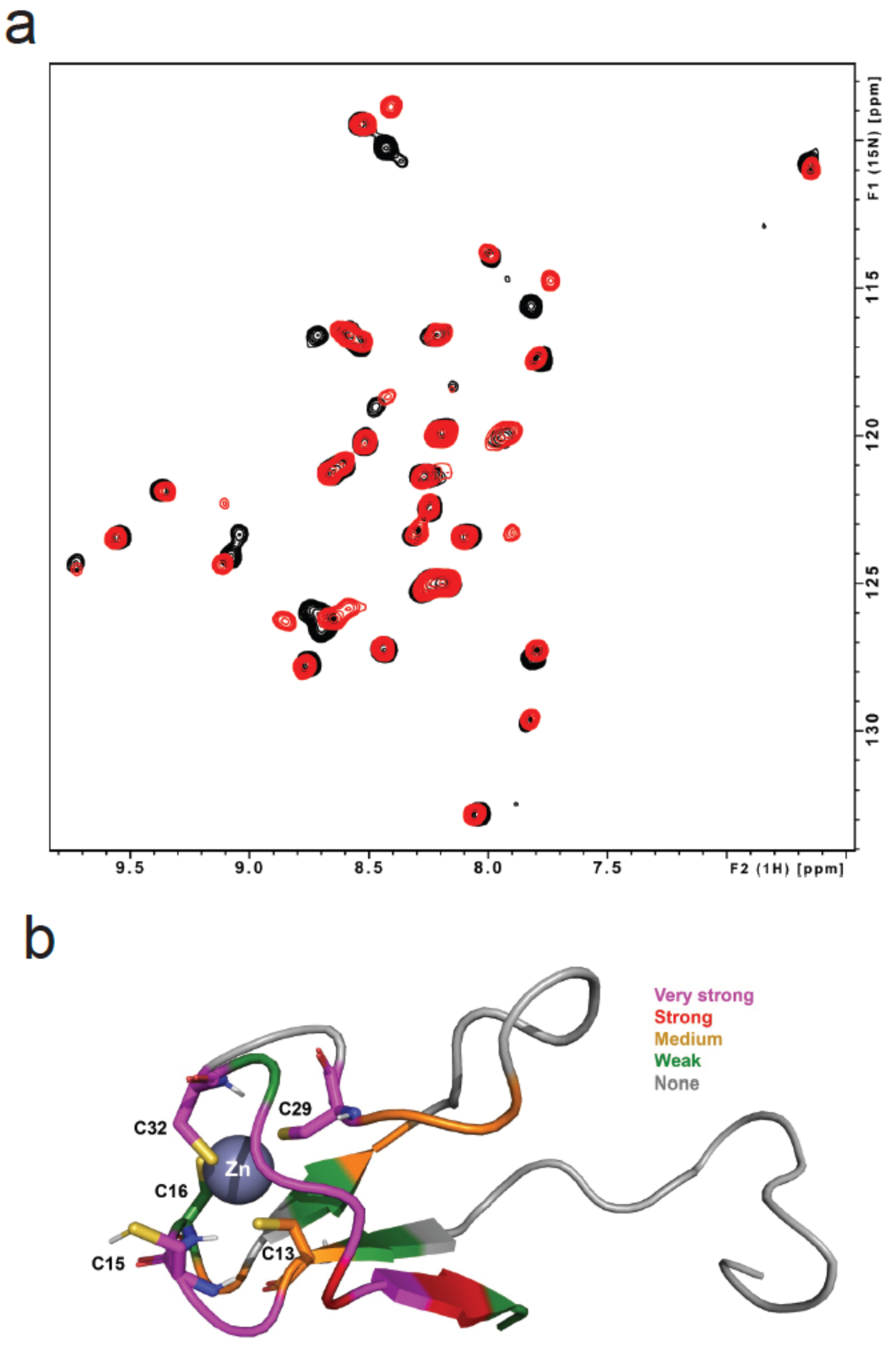

Figure S5. Fumarate binding to RNF126(1-40) is specific and does not lead to protein unfolding or aggregation. (a) Overlay of two $^{1}H,^{15}N$-HMQC spectra, with peaks shown in black corresponding to ~75 µM of apo-RNF126 and signals in red representing protein amide groups in the presence of 200 µM of JP-2-196. The compound-induced, peak-dependent chemical shift perturbations are proof of specific binding of the small molecule to RNF126. Overall protein signal intensities and the spectral dispersion are unchanged upon compound addition, indicating that the ligand interactions do not lead to protein unfolding or aggregation. **(b)** Three-dimensional structure of RNF126(1-40) with color-coded mapping of chemical shift perturbations and residue-specific line broadening. The ligand binds at or close to the zinc ("Zn") coordination site while the other areas of RNF126(1-40) remain unchanged.

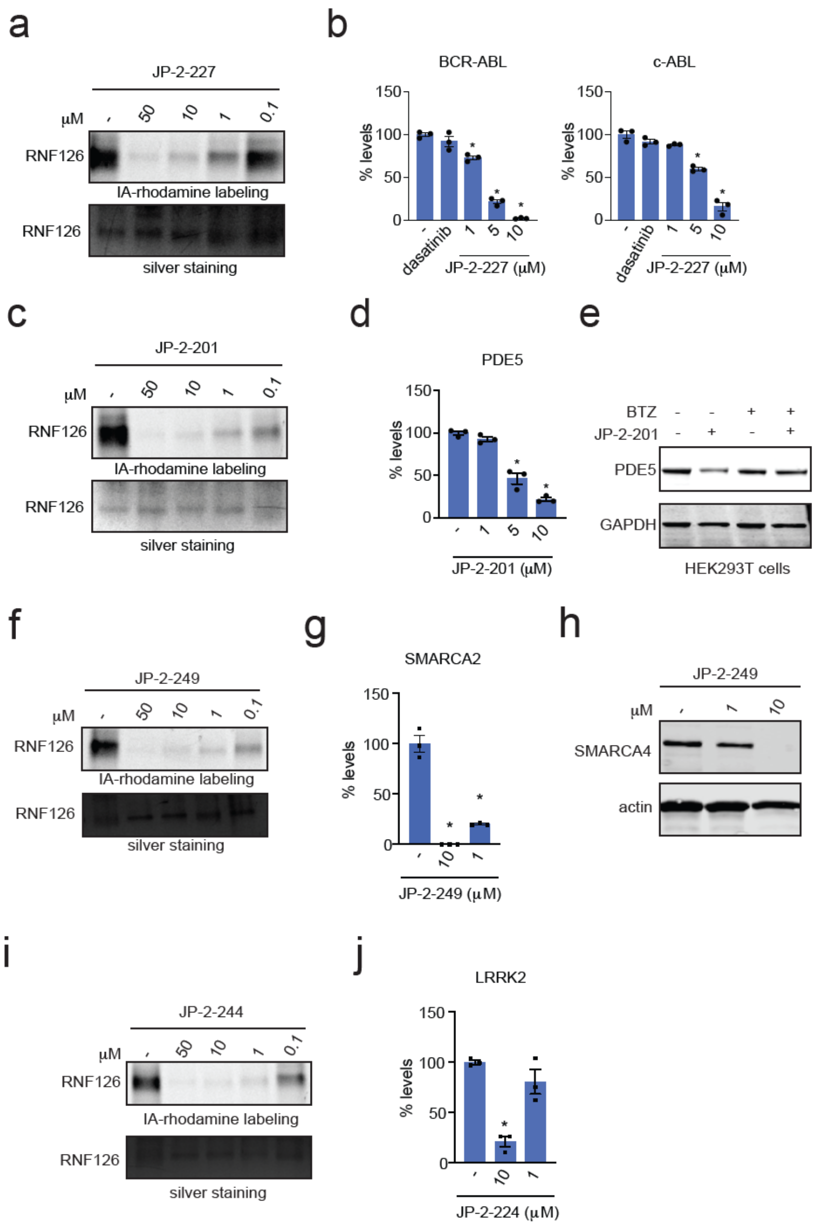

Figure S6. Characterizing Degraders Against Additional Targets. (a) Gel-based ABPP analysis of JP-2-227 against RNF126. Recombinant RNF126 was pre-incubated with DMSO vehicle or JP-2-227 for 30 min prior to labeling of RNF126 with IA-rhodamine (250 nM) for 1 h. Gels were visualized by in-gel fluorescence and protein loading was assessed by silver staining. **(b)** Quantitation of data in **Figure 4b**. **(c)** Gel-based ABPP analysis of JP-2-201 as described in **(a)**. **(d)** Quantitation of data in **Figure 4d. (e)** JP-2-201-mediated degradation of PDE5 is proteasome-dependent. HEK293T cells were pre-treated with DMSO vehicle or bortezomib (1 μM) for 1 h prior to treating cells with DMSO or JP-2-201 (10 μM). PDE5 and loading control GAPDH levels were assessed by Western blotting. **(f)** Gel-based ABPP analysis of JP-2-249 against RNF126 as described in **(a)**. **(g)** Quantitation of data in **Figure 4f. (h)** MV-4-11 cells were treated with DMSO vehicle or JP-2-249 for 24 h and SMARCA4 and loading control actin levels were assessed by Western blotting. **(i)** Gel-based ABPP of JP-2-244 against RNF126 as described in **(a)**. **(j)** Quantitation of data in **Figure 4h.** Gels and blots shown in **(a, c, e, f, h, i)** are representative images from n=3 biological replicates. Bar graphs in **(b, d, g, j)** show individual replicate values and average ± sem. Statistical significance is calculated as *p<0.05 compared to DMSO vehicle.

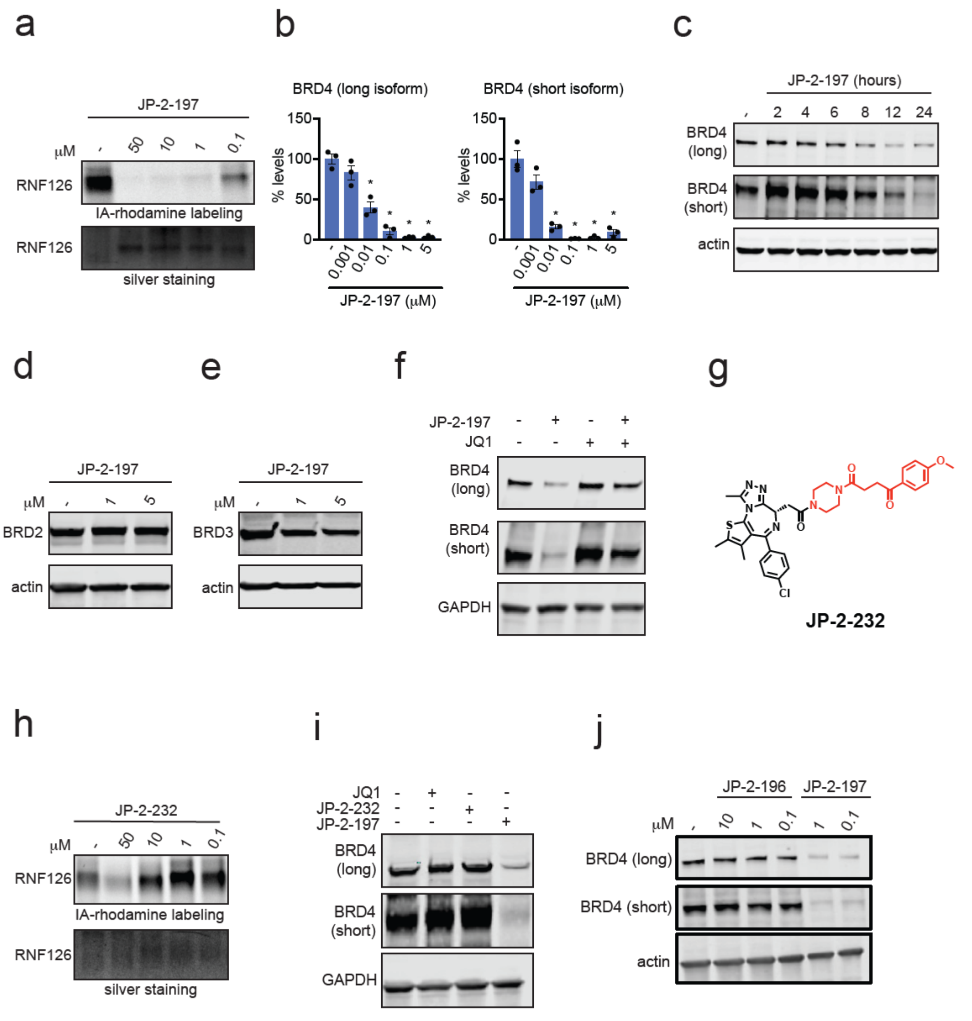

Figure S7. Characterization of BRD4 degraders and their derivatives. (a) Gel-based ABPP analysis of JP-2-197 against RNF126. Recombinant RNF126 was pre-incubated with DMSO vehicle or JP-2-197 for 30 min prior to labeling of RNF126 with IA-rhodamine (250 nM) for 1 h. Gels were visualized by in-gel fluorescence and protein loading was assessed by silver staining. **(b)** Quantitation of data in **Figure 5b. (c)** Time-course of BRD4 degradation with JP-2-197. HEK293T cells were treated with DMSO vehicle or JP-2-197 (1 μM) and BRD4 and loading control actin levels were assessed by Western blotting. **(d, e)** BRD2 **(d)** and BRD3 **(e)** levels in HEK293T cells. HEK293T cells were treated with DMSO vehicle or JP-2-197 for 24 h and BRD2, BRD3, and loading control actin levels were assessed by Western blotting. **(f)** HEK293T cells were pre-treated with either DMSO vehicle or JQ1 (1 μM) for 1 h prior to treating cells with DMSO or JP-2-197 (0.1 μM) for 24 h and BRD4 and loading control GAPDH levels were assessed by Western blotting. **(g)** Structure of non-reactive derivative of JP-2-197, JP-2-232. **(h)** Gel-based ABPP of JP-2-232 against RNF126. Recombinant RNF126 was pre-incubated with DMSO vehicle or JP-2-232 for 30 min prior to labeling of RNF126 with IA-rhodamine (250 nM) for 1 h. Gels were visualized by in-gel fluorescence and protein loading was assessed by silver staining. **(i)** BRD4 levels in HEK293T cells. HEK293T cells were treated with DMSO vehicle, JP-2-197, or JP-2-232 (1 μM) for 24 h and BRD4 and loading control GAPDH levels were detected by Western blotting. **(j)** HEK293T cells were treated with DMSO vehicle, JP-2-196, or JP-2-197 for 24 h and BRD4 and loading control actin levels were assessed by Western blotting. Gels and blots in **(a, c, d, e, f, h, i, j)** are representative of n=3 biologically independent replicates/group. Bar graphs in **(b)** show individual replicate values and average ± sem. Statistical significance is calculated as *p<0.05 compared to DMSO vehicle.

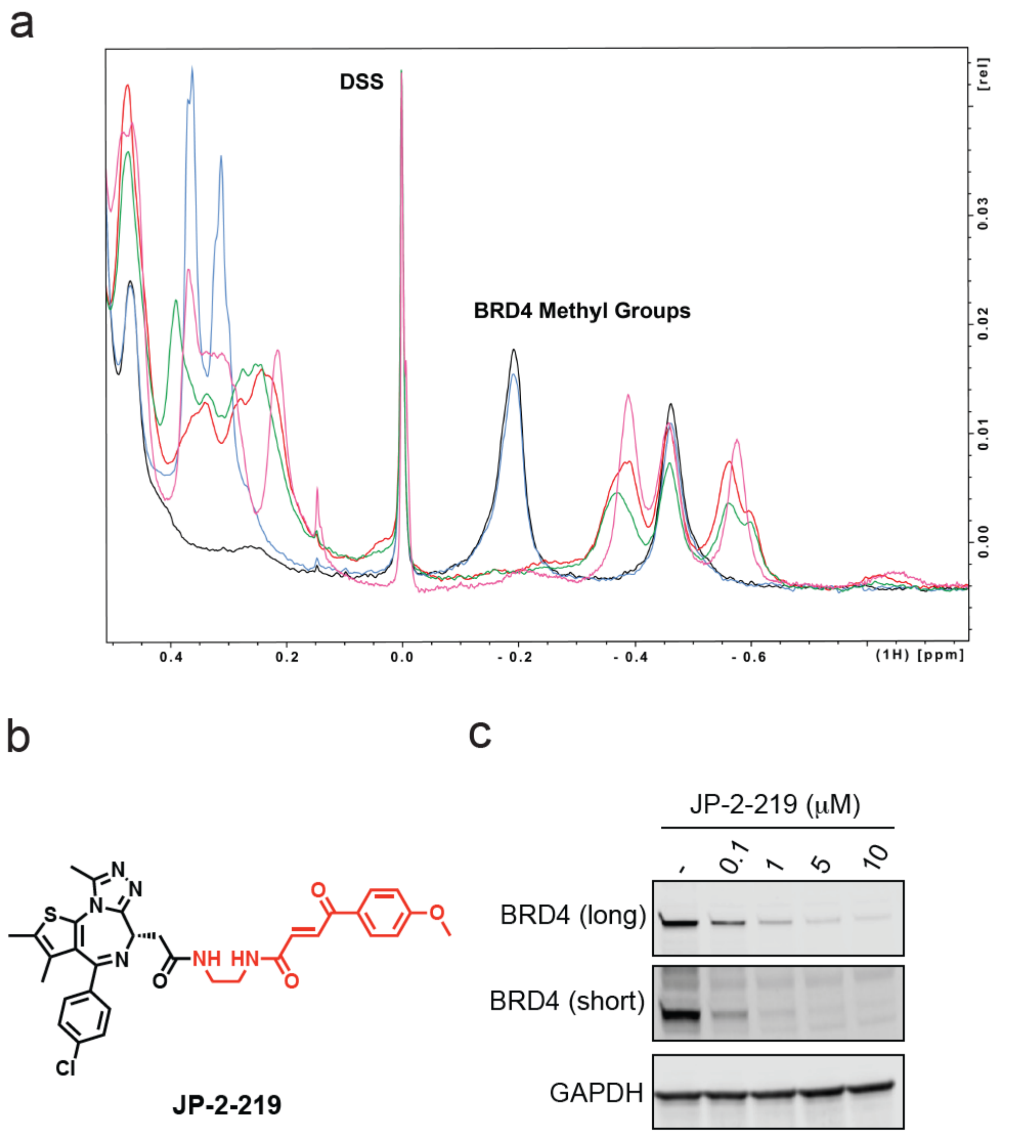

Figure S8. Characterization of BRD4 Degraders. (a) Overlay of ^{1}H-1D spectra of the following samples: 50 μM BRD4(44-168) (black); 50 μM BRD4(44-168) + 200 μM JP-2-197 (red); 50 μM BRD4(44-168) + RNF126(1-40) + JP-2-197 (green); 50 μM BRD4(44-168) + RNF126(1-40) (blue); 50 μM BRD4(44-168) + RNF126(1-40) + 100 μM JQ1 + 100 μM JP-2-196 (magenta). BRD4 (black spectrum) and JP-2-197 strongly interact with each other as indicated by the drastic perturbation of one of the upfield shifted BRD4 methyl signals (δ ≈ -0.19 ppm) and the appearance of additional signals (red spectrum). When RNF126(1-40) is present, the perturbations are further pronounced as small additional chemical shifts and line broadening are detected, suggesting slowed down protein tumbling due to the formation of a ternary protein-protein-glue complex (green spectrum). There is hardly any effect on the BRD4 methyl peaks in the absence of the glue molecule (blue spectrum). No line broadening is observed when the unlinked protein ligands JQ1 (BRD4 ligand) and JP-2-196 (RNF126 ligand) are added to the protein mixture (magenta). **(b)** Shown is a structure of JP-2-219, a BRD4 degrader with an alkyl linker rather than a piperazine linking the fumarate handle to the BRD4 inhibitor JQ1. **(c)** BRD4 levels in HEK293T cells. HEK293T cells were treated with DMSO vehicle or JP-2-219 (1 μM) for 24 h and BRD4 and loading control GAPDH levels were detected by Western blotting. Blot is a representative of n=3 biologically independent replicates/group.

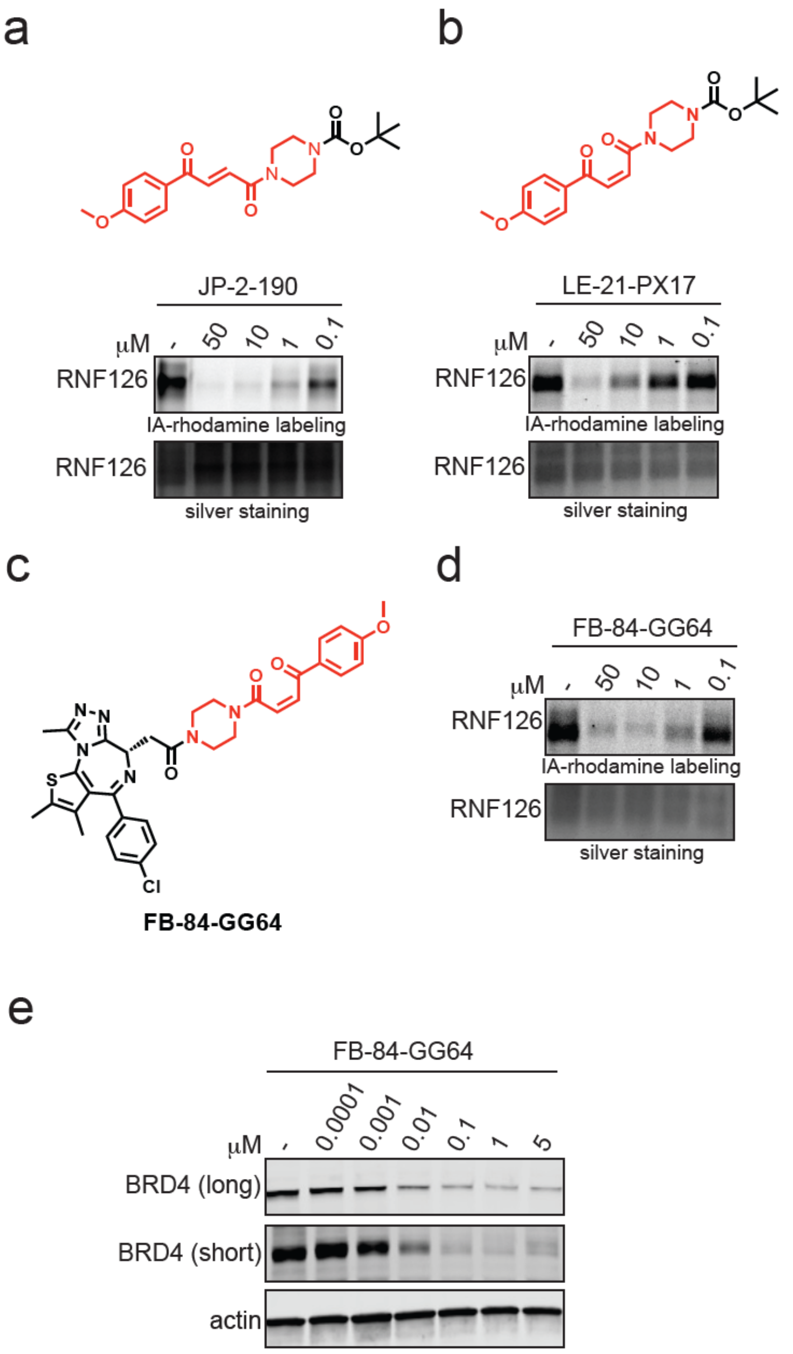

Figure S9. Characterizing the *cis*-isomer covalent handle and degrader. (a, b) Gel-based ABPP of JP-2-190 and LE-21-PX17 against RNF126. Recombinant RNF126 was pre-incubated with DMSO vehicle or compounds for 30 min prior to labeling of RNF126 with IA-rhodamine (250 nM) for 1 h. Gels were visualized by in-gel fluorescence and protein loading was assessed by silver staining. **(c)** Structure of the *cis* isoform of the JP-2-197 BRD4 degrader, FB-84-GG64. **(d)** Gel-based ABPP of FB-84-GG64 against RNF126. Recombinant RNF126 was pre-incubated with DMSO vehicle or FB-84-GG64 for 30 min prior to labeling of RNF126 with IA-rhodamine (250 nM) for 1 h. Gels were visualized by in-gel fluorescence and protein loading was assessed by silver staining. **(e)** BRD4 degradation by FB-84-GG64 in HEK293T cells. HEK293T cells were treated with DMSO vehicle or FB-84-GG64 for 24 h and BRD4 and loading control actin levels were assessed by Western blotting. Gels and blots shown in **(a, b, d, e)** are representative of n=3 biologically independent replicates/group.

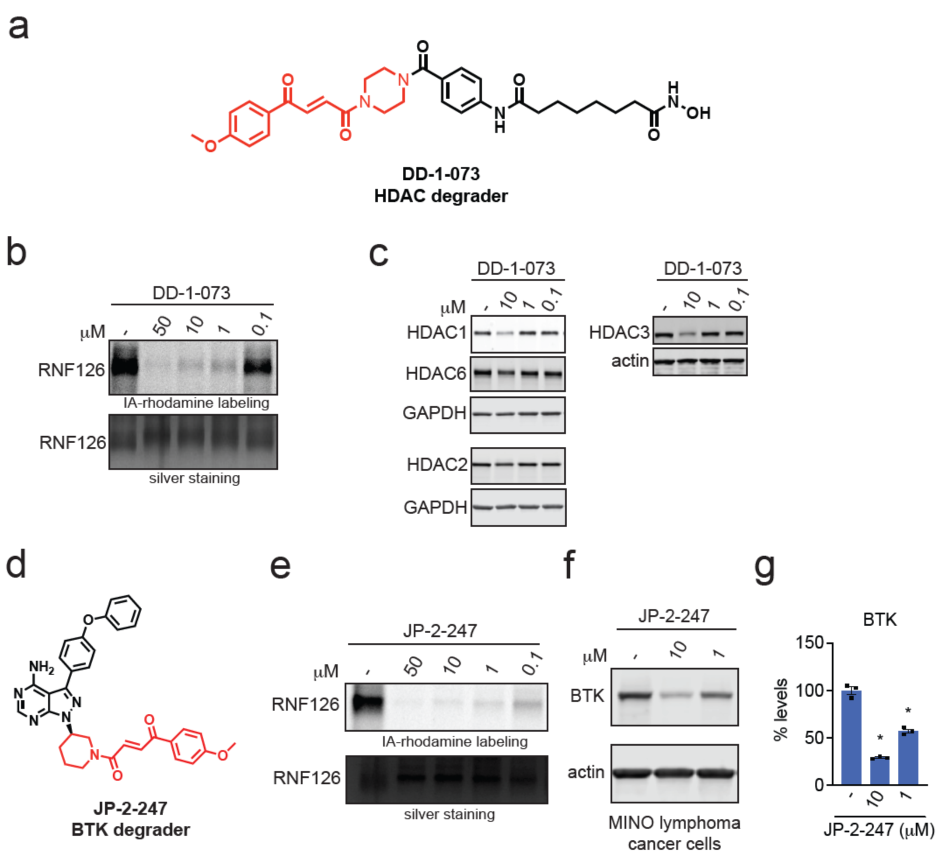

Figure S10. Characterization of HDAC and BTK Degraders. (a) Structure of DD-1-073 HDAC degrader linking our fumarate JP-2-196 handle to the HDAC inhibitor vorinostat. **(b)** Gel-based ABPP of DD-1-073 against RNF126. Recombinant RNF126 was pre-incubated with DMSO vehicle or DD-1-073 for 30 min prior to labeling of RNF126 with IA-rhodamine (250 nM) for 1 h. Gels were visualized by in-gel fluorescence and protein loading was assessed by silver staining. **(c)** HDAC1, HDAC2, HDAC3, and HDAC6 levels in HEK293T cells. HEK293T cells were treated with DMSO vehicle or DD-1-073 for 24 h and HDAC and loading control GAPDH and actin levels were detected by Western blotting. **(d)** Structure of a BTK degrader JP-2-247 consisting of the fumarate handle incorporated into the BTK inhibitor ibrutinib. **(e)** Gel-based ABPP analysis of JP-2-247 against RNF126. Recombinant RNF126 was pre-incubated with DMSO vehicle or JP-2-247 for 30 min prior to labeling of RNF126 with IA-rhodamine (250 nM) for 1 h. Gels were visualized by in-gel fluorescence and protein loading was assessed by silver staining. **(f)** MINO lymphoma cancer cells were treated with DMSO vehicle or JP-2-247 for 24 h and BTK and loading control actin levels were detected by Western blotting. **(g)** Quantitation of the data shown in **(f)**. Gels and blots shown in **(b, c, e, f)** are representative of n=3 biologically independent replicates/group. Bar graph in **(g)** shows individual replicate values and average ± sem. Statistical significance is calculated as *p<0.05 compared to DMSO vehicle.

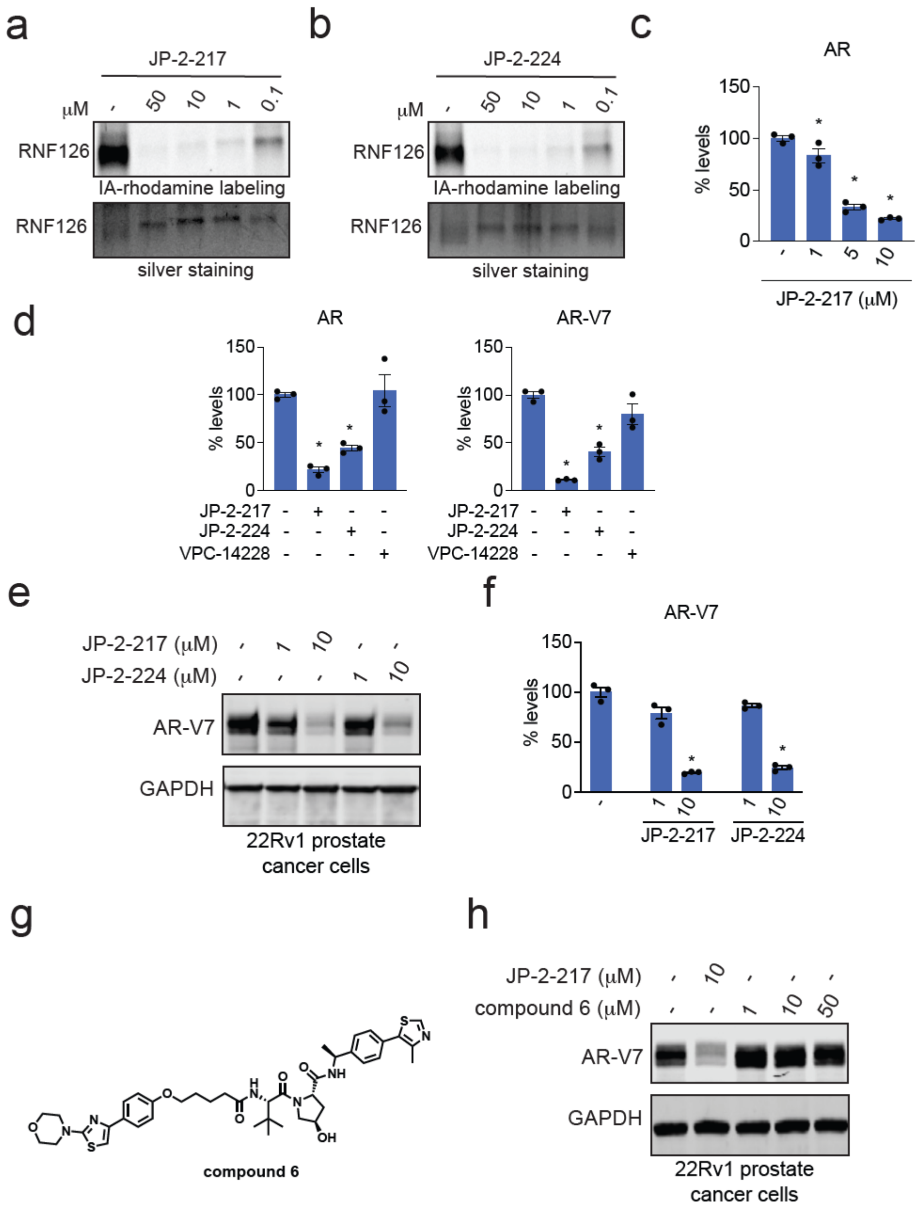

Figure S11. Characterization of AR-V7 Degraders. (a, b) Gel-based ABPP analysis of JP-2-217 **(a)** and JP-2-224 **(b)** against RNF126. Recombinant RNF126 was pre-incubated with DMSO vehicle or JP-2-217 or JP-2-224 for 30 min prior to labeling of RNF126 with IA-rhodamine (250 nM) for 1 h. Gels were visualized by in-gel fluorescence and protein loading was assessed by silver staining. **(c)** Quantitation of data in **Figure 5g**. **(d)** Quantitation of data in **Figure 5h. (e)** 22Rv1 cells were treated with DMSO vehicle, JP-2-217, or JP-2-224 for 24 h and AR-V7 and loading control GAPDH levels were detected by Western blotting. **(f)** Quantified AR-V7 levels from experiment described in **(e). (g)** Structure of a previously reported AR-V7 degrader linking the AR DNA binding domain ligand VPC-14228 to a VHL recruiter, compound 6. **(h)** AR-V7 levels in 22Rv1 cells. 22Rv1 cells were treated with DMSO vehicle, JP-2-217, or compound 6 for 24 h and AR-V7 and loading control GAPDH levels were detected by Western blotting. Gels and blots in **(a, b, e, h)** are representative of n=3 biologically independent replicates/group with individual replicates. Bar graphs in **(c, d, f)** show individual replicate values and average ± sem. Statistical significance is calculated as *p<0.05 compared to DMSO vehicle.

Supporting Table Legends

Table S1. TMT-based quantitative proteomic profiling of EST1027 in C33A cells. C33A cells were treated with DMSO vehicle or EST1027 (5 μM) for 24 h. Data are from n=3 biological replicates per group.

Table S2. Cysteine chemoproteomic profiling of EST1027 in C33A cells using isoDTB-ABPP. C33A cells were treated with DMSO vehicle or EST1027 (20 μM) for 2 h**.** Resulting lysates were labeled with an alkyne-functionalized iodoacetamide probe (IA-alkyne) (200 μM) for 1 h, after which isotopic desthiobiotin tags were appended by CuAAC and taken through the isoDTB-ABPP procedure. Experiment was from n=3 biologically-independent replicates.

Table S3. Quantitative Proteomics of JP-2-196-Alkyne Pulldown in HEK293T cells. JP-2-196 pulldown proteomics showing significant and moderately selective engagement of RNF126 with less significant engagement of 5 additional E3 ubiquitin ligases LRSAM1, RNF40, MID2, RNF219, and RNF14. HEK293T cells were treated with DMSO vehicle or JP-2-196-alkyne (10 μM) for 6 h. Subsequent lysates were subjected to copper-catalyzed azide-alkyne cycloaddition (CuAAC) with an azide-functionalized biotin handle, after which probe-modified proteins were avidin-enriched, eluted, and digested, and analyzed by TMT-based quantitative proteomics. Data shown are ratios of JP-2-196-alkyne vs DMSO control enriched proteins and p-values from n=3 biologically independent replicates/group.

Table S4. TMT-based quantitative proteomic profiling of JP-2-197 in HEK293T cells. HEK293T cells were treated with DMSO vehicle, JP-2-196 (1 μM), or JP-2-197 (1 μM) for 24 h. Data are from n=2 biological replicates per group.

Synthetic Methods and Characterization

All chemical reactions were carried out under a nitrogen atmosphere with dry solvents under anhydrous conditions, unless otherwise noted. Reagents were purchased at the highest commercial quality and used without further purification, unless otherwise stated. The key building block, *trans*-3-(4-methoxybenzoyl)acrylic acid, was acquired from Sigma-Aldrich (Catalog Number: 650765) at a reported purity of 99%, however upon further investigation this purity was found to vary between batches, consisting of anywhere from 0-10% of the *cis*-isomer. Reactions were stirred magnetically and monitored by thin layer chromatography (TLC) carried out on Merck glass silica gel plates (60 F254) using UV light as a visualizing agent and iodine and/or phosphomolybdic acid stain as developing agents. Solvents were removed *in vacuo* using either a Buchi R-300 Rotavapor (equipped with an I-300 Pro Interface, B-300 Base Heating Bath, Welch 2037B-01 DryFast pump, and VWR AD15R-40-V11B Circulating Bath). Solvents for silica gel chromatography were used as supplied by Sigma-Aldrich. Automated flash chromatography was performed on a Biotage Isolera instrument, equipped with a UV detector. Chromatograms were recorded at 254 and 280 nm. Analytical ultra-performance liquid chromatography (UPLC) was performed on a Waters Acquity UPLC-MS(SQD) with a Phenomenex Kinetix EVO-C18 2.6 µm, 100 Å, 2.1 × 50 mm column. Solvents (water and acetonitrile) containing 0.1% trifluoroacetic acid (TFA) were used. A gradient of 2-88% acetonitrile in water, 0−10 min was used at ambient temperature. Purification was run with a flow rate of 1.2 mL/min with detection at 220 nm. Certain fumarate analogs were sensitive to acidic aqueous purification conditions, and a small fraction was found to isomerize between the *E* and *Z*-isomers therefore a neutral, SFC analytical method was also employed using an Amino Phenyl SFC 5 µm, 120 Å, 30 mm × 25 cm column. Solvents (CO_2 and MeOH) were used at a flow rate of 2.5 mL/min with detection at 220 nm. The purity of final compounds was evaluated using the analytical systems described above, characterized by MS and nuclear magnetic resonance (NMR) and ratios of *E/Z*-isomers were reported as detected in UPLC analysis. Isomerization rates were analyzed for key final compounds by preparing small amounts of material in a 1:1 mixture of water:acetonitrile, filtering through a 0.2 µm syringe filter for UPLC analysis at days 1 and 6. Low-resolution mass spectra were obtained using Agilent 6460 Triple Quad LC/MS instrument. High-resolution mass spectra (HRMS) were obtained at the Catalysis Center at the College of Chemistry, University of California, Berkeley. ^{1}H and ^{13}C nuclear magnetic resonance (NMR) spectra were recorded on BRUKER AV (600 MHz and 700 MHz), AVB (400 MHz), AVQ (400 MHz) and NEO (500 MHz)

spectrometers. Measurements were carried out at ambient temperature. Chemical shifts (δ) are reported in ppm with the residual solvent signal as internal standard (chloroform at 7.26 and 77.00 ppm for ^{1}H NMR and ^{13}C NMR spectroscopy, respectively). The data is reported as (s = singlet, d = doublet, t = triplet, q = quartet, p = quintet, m = multiplet or unresolved, br = broad signal, coupling constant(s) in Hz, integration). 13C NMR spectra were recorded with broadband 1H decoupling.

General Procedures

Amide Couplings

General Procedure A

A mixture of the corresponding carboxylic acid (1.1 equiv.) and HATU (1.2 equiv.) was purged with N_2 for 5 minutes. The mixture was dissolved in *N,N*-dimethylformamide (DMF) (0.1 M), *N,N*-diisopropylethylamine (DIPEA) (3 equiv.) was added and the reaction mixture was allowed to stir at ambient temperature for 30 minutes. The corresponding amine (1 equiv.) was dissolved in DMF (0.1 M) then added dropwise and the reaction mixture was stirred at ambient temperature overnight. The reaction was quenched with 5 times the reaction volume of 5% $LiCl_{(aq)}$ and extracted 3 times with dichloromethane (DCM) or ethyl acetate (EtOAc). The organic extracts were dried over Na_2SO_4, vacuum filtered, and concentrated *in vacuo*. The resultant residue was purified by silica gel flash chromatography to afford the title compound.

General Procedure B

The corresponding carboxylic acid (1.1 equiv.) was added to a vessel and purged with N_2 for 5 minutes. The acid was then dissolved in DCM (0.1 M) and cooled to 0 °C in an ice bath. Oxalyl chloride (1.2 equiv.) was added dropwise at 0 °C. A few drops of DMF were added and the reaction mixture was allowed to stir and come to ambient temperature over 2 hours. The volatiles were removed *in vacuo* and the resultant residue was redissolved in DCM (0.1 M) and cooled to 0 °C in an ice bath. The corresponding amine (1.1 equiv.) was dissolved in DCM (0.1M). DIPEA (3 equiv.) was added, and the mixture was stirred for 5 minutes at ambient temperature before being added to the acyl chloride dropwise at 0 °C. The reaction mixture was allowed to stir and come to ambient temperature overnight. The reaction was quenched with water and extracted 3 times with

EtOAc. The organic extracts were dried over Na_2SO_4, vacuum filtered, and concentrated *in vacuo*. The resultant residue was purified by silica gel flash chromatography to afford the title compound.

General Procedure C

The corresponding carboxylic acid (1.1 equiv.) was added to a vessel and purged with N_2 for 5 minutes. The acid was dissolved in acetonitrile (0.1 M) and DIPEA (2 equiv.) was added. Pentafluoropyridine (1.1 equiv.) was added dropwise, and the reaction mixture was stirred at ambient temperature for 30 minutes. The corresponding amine was dissolved in acetonitrile (0.1 M) and added to acyl fluoride. The reaction mixture was stirred at 100 °C overnight. The volatiles were removed *in vacuo*, and the resultant residue was purified by silica gel flash chromatography to afford the title compound.

General Procedure D

The corresponding carboxylic acid (1.0 equiv.) was added to a vessel and purged with N_2 for 5 minutes. The acid was dissolved in DMF (0.1 M) and DIPEA (3 equiv.) was added. A >50% wt. solution of propylphosphonic anhydride (T3P) in EtOAc (1.5 equiv.) was added dropwise, and the reaction mixture was stirred at ambient temperature for 30 minutes. The corresponding amine (1.2 equiv.) was dissolved in DMF (0.1 M) then added dropwise and the reaction mixture was stirred at ambient temperature overnight. The reaction was quenched with 5 times the reaction volume of 5% $LiCl_{(aq)}$ and extracted 3 times with EtOAc. The organic extracts were washed once with 5% $LiCl_{(aq)}$, dried over Na_2SO_4, vacuum filtered, and concentrated *in vacuo*. The resultant residue was purified by silica gel flash chromatography to afford the title compound.

Tert-butyloxycarbonyl Deprotection

General Procedure E

The corresponding tert-butyloxycarbonyl protected amine (1 equiv.) was dissolved in DCM (0.1 M). Trifluoroacetic acid (32 equiv.) was added, and the reaction mixture was stirred at ambient temperature for 30 minutes to overnight. The volatiles were removed *in vacuo* and the crude residue was used without further purification, unless otherwise noted.

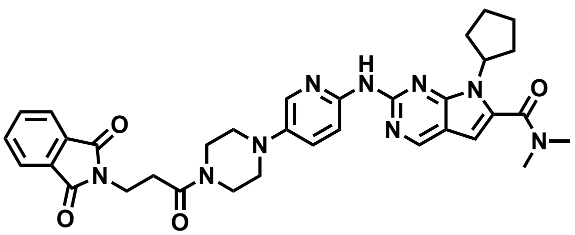

EST1001

General Procedure A was followed with 3-phthalimidopropionic acid (21.6 mg, 0.06 mmol), HATU (43.8 mg, 0.12 mmol), DIPEA (0.04 mL, 0.23 mmol), and ribociclib (25.0 mg, 0.06 mmol). The crude residue was purified by silica gel chromatography (0-10% MeOH in DCM) to afford 14.9 mg (41%) of the title compound as a yellow film.

^{1}H NMR (500 MHz, $CDCl_3$) δ 8.70 (s, 1H), 8.37 (d, *J* = 9.0 Hz, 1H), 8.03 – 7.95 (m, 2H), 7.89 – 7.81 (m, 2H), 7.75 – 7.68 (m, 2H), 7.32 (dd, *J* = 9.1, 3.0 Hz, 1H), 6.44 (s, 1H), 4.79 (p, *J* = 9.0 Hz, 1H), 4.09 – 4.03 (m, 2H), 3.80 (t, *J* = 5.2 Hz, 2H), 3.64 (t, *J* = 5.1 Hz, 2H), 3.15 (s, 6H), 3.14 – 3.08 (m, 4H), 2.85 – 2.78 (m, 2H), 2.65 – 2.52 (m, 2H), 2.06 (m 4H), 1.77 – 1.70 (m, 2H).

^{13}C NMR (151 MHz, $CDCl_3$) δ 168.4, 168.2, 164.1, 154.5, 151.9, 151.8, 147.6, 142.1, 137.6, 134.0, 132.1, 132.1, 127.4, 123.3, 112.7, 112.4, 101.0, 57.9, 50.6, 50.3, 45.4, 41.5, 34.3, 31.6, 30.2, 24.7.

LRMS (ESI) *m/z* calcd for $[C_{34}H_{37}N_9O_4 + H]^+$ = 636.3, found 636.4.

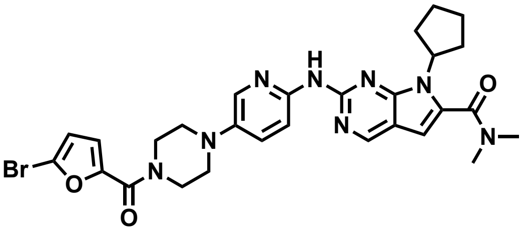

EST1004

General Procedure A was followed with 5-Bromo-2-furoic acid (11.0 mg, 0.06 mmol), HATU (43.8 mg, 0.12 mmol), DIPEA (0.04 mL, 0.23 mmol), and ribociclib (25.0mg, 0.06 mmol). The crude residue was purified by silica gel chromatography (0-15% MeOH in DCM) to afford 23.9 mg (63%) of the title compound as a yellow film.

^{1}H NMR (500 MHz, $CDCl_3$) δ 8.72 (s, 1H), 8.39 (d, *J* = 9.0 Hz, 1H), 8.25 (s, 1H), 8.06 (d, *J* = 3.0 Hz, 1H), 7.34 (dd, *J* = 9.1, 3.0 Hz, 1H), 7.03 (d, *J* = 3.5 Hz, 1H), 6.48 – 6.42 (m, 2H), 4.79 (p, *J* = 8.9 Hz, 1H), 3.98 (s, 4H), 3.24 – 3.18 (m, 4H), 3.15 (s, 6H), 2.58 (m, 2H), 2.11 – 2.01 (m, 4H), 1.76 – 1.68 (m, 2H).

^{13}C NMR (151 MHz, $CDCl_3$) δ 164.1, 157.8, 154.6, 151.9, 151.9, 149.5, 147.7, 142.0, 137.5, 132.0, 127.3, 124.4, 119.3, 113.5, 112.6, 112.5, 101.0, 57.9, 50.6, 30.2, 24.7.

LRMS (ESI) *m/z* calcd for $[C_{28}H_{31}BrN_8O_3 + H]^+$ = 607.3, found 607.2.

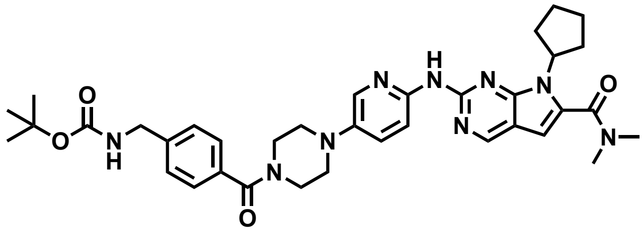

EST1007

General Procedure A was followed with 4-(Boc-aminomethyl)benzoic acid (14.5 mg, 0.06 mmol), HATU (43.8 mg, 0.12 mmol), DIPEA (0.04 mL, 0.23 mmol), and ribociclib (25.0 mg, 0.06 mmol). The crude residue was purified by silica gel chromatography (0-15% MeOH in DCM) to afford 29.5 mg (77%) of the title compound as a yellow film.

^{1}H NMR (500 MHz, $CDCl_3$) δ 8.72 (s, 1H), 8.37 (d, *J* = 9.1 Hz, 1H), 8.26 (s, 1H), 8.04 (d, *J* = 2.9 Hz, 1H), 7.41 (d, *J* = 7.9 Hz, 2H), 7.36 – 7.30 (m, 3H), 6.44 (s, 1H), 4.99 (d, *J* = 6.5 Hz, 1H), 4.79 (p, *J* = 9.0 Hz, 1H), 4.35 (d, *J* = 6.1 Hz, 2H), 3.94 (s, 2H), 3.62 (s, 2H), 3.20 (br s, 2H), 3.15 (s, 6H), 3.09 (br s, 2H), 2.58 (m, 2H), 2.11 – 2.00 (m, 4H), 1.76 – 1.67 (m, 2H), 1.46 (s, 9H).

^{13}C NMR (151 MHz, $CDCl_3$) δ 170.2, 164.1, 156.0, 154.6, 151.9, 151.9, 147.7, 142.0, 141.1, 137.6, 134.4, 132.0, 127.5, 127.4, 112.7, 112.5, 101.0, 79.7, 57.9, 50.6, 44.3, 39.4, 35.2, 30.2, 28.4, 24.7.

LRMS (ESI) *m/z* calcd for $[C_{36}H_{45}N_9O_4 + H]^+$ += 668.4, found 668.5.

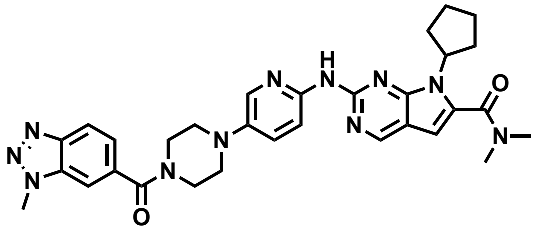

EST1012

General Procedure A was followed with 1-Methyl-1h-1,2,3-benzotriazole-6-carboxylic acid (10.2 mg, 0.06 mmol), HATU (43.8 mg, 0.12 mmol), DIPEA (0.04 mL, 0.23 mmol), and ribociclib (25.0 mg, 0.06 mmol). The crude residue was purified by silica gel chromatography (0-15% MeOH in DCM) to afford 23.7 mg (69%) of the title compound as a yellow film.

^{1}**H NMR** (500 MHz, $CDCl_3$) δ 8.72 (s, 1H), 8.34 (s, 1H), 8.21 (s, 1H), 8.15 (d, *J* = 1.1 Hz, 1H), 8.04 (d, *J* = 2.9 Hz, 1H), 7.68 – 7.58 (m, 2H), 7.35 (dd, *J* = 9.0, 2.9 Hz, 1H), 6.44 (s, 1H), 4.79 (p, *J* = 8.9 Hz, 1H), 4.34 (s, 3H), 3.94 (br s, 2H), 3.70 (br s, 2H), 3.15 (m, 10H), 2.57 (m, 2H), 2.12 – 1.99 (m, 4H), 1.71 (d, *J* = 4.1 Hz, 2H).

^{13}**C NMR** (126 MHz, $CDCl_3$) δ 169.7, 164.0, 151.9, 145.3, 142.0, 134.1, 131.3, 127.1, 119.2, 112.9, 112.6, 109.9, 101.0, 57.9, 50.6, 34.5, 30.2, 24.7.

LRMS (ESI) *m/z* calcd for $[C_{31}H_{35}N_{11}O_2 + H]^+$ = 594.3, found 594.3 .

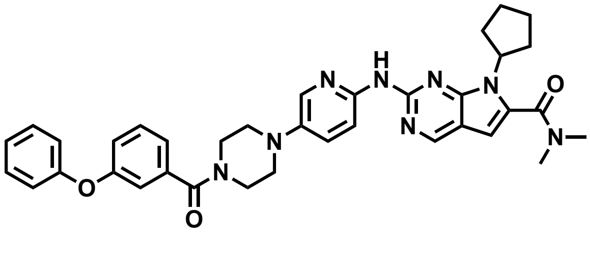

EST1018

General Procedure A was followed with 3-phenoxybenzoic acid (12.3 mg, 0.06 mmol), HATU (43.8 mg, 0.12 mmol), DIPEA (0.04 mL, 0.23 mmol), and ribociclib (25.0 mg, 0.06 mmol). The crude residue was purified by silica gel chromatography (0-15% MeOH in DCM) to afford 30.1 mg (83%) of the title compound as a yellow film.

^{1}**H NMR** (500 MHz, $CDCl_3$) δ 8.72 (s, 1H), 8.39 (d, *J* = 9.0 Hz, 1H), 8.30 (s, 1H), 8.05 (d, *J* = 2.9 Hz, 1H), 7.47 – 7.42 (m, 2H), 7.42 – 7.31 (m, 3H), 7.19 – 7.12 (m, 1H), 7.04 (ddd, *J* = 13.1, 7.6, 1.6 Hz, 4H), 6.44 (s, 1H), 4.79 (p, *J* = 8.9 Hz, 1H), 3.96 – 3.62 (m, 4H), 3.15 (m, 10H), 2.64 – 2.53 (m, 2H), 2.11 – 2.00 (m, 4H), 1.76 – 1.65 (m, 2H).

^{13}**C NMR** (126 MHz, $CDCl_3$) δ 170.1, 164.1, 159.1, 156.2, 154.5, 151.9, 151.8, 147.6, 142.1, 137.3, 132.1, 130.0, 129.8, 129.3, 127.5, 124.1, 119.7, 118.1, 112.7, 112.5, 101.0, 57.9, 50.7, 39.5, 35.2, 30.2, 24.7. (Mixture of rotamers)

LRMS (ESI) *m/z* calcd for $[C_{36}H_{38}N_8O_3 + H]^+$ = 631.3, found 631.3.

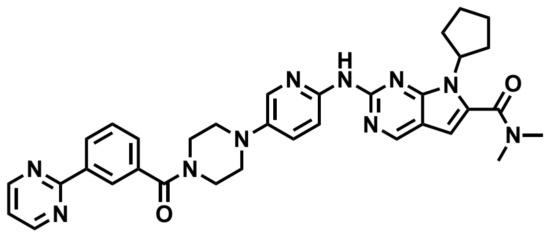

EST1021

General Procedure A was followed with 3-(2-pyrimidinyl)benzoic acid (11.5 mg, 0.06 mmol), HATU (43.8 mg, 0.12 mmol), DIPEA (0.04 mL, 0.23 mmol), and ribociclib (25.0 mg, 0.06 mmol). The crude residue was purified by silica gel chromatography (0-15% MeOH in DCM) to afford 12.2 mg (34%) of the title compound as a yellow film.

^{1}H NMR (500 MHz, $CDCl_3$) δ 8.83 (d, *J* = 4.8 Hz, 2H), 8.69 (s, 1H), 8.54 (dt, *J* = 8.9, 1.7 Hz, 2H), 8.38 (d, *J* = 9.1 Hz, 1H), 8.01 (d, *J* = 2.9 Hz, 1H), 7.92 (s, 1H), 7.63 – 7.54 (m, 2H), 7.35 (dd, *J* = 9.1, 2.9 Hz, 1H), 7.23 (t, *J* = 4.8 Hz, 1H), 6.44 (s, 1H), 4.79 (p, *J* = 8.9 Hz, 1H), 4.01 (br s, 2H), 3.69 (br s, 2H), 3.25 (br s, 2H), 3.15 (s, 6H), 3.13 (br s, 2H), 2.64 – 2.52 (m, 2H), 2.12 – 1.99 (m, 4H), 1.77 – 1.69 (m, 2H).

^{13}C NMR (151 MHz, $CDCl_3$) δ 170.1, 164.1, 163.9, 157.4, 154.4, 152.0, 151.8, 147.5, 142.2, 137.9, 135.9, 132.2, 129.5, 129.5, 129.1, 127.6, 126.8, 119.5, 112.8, 112.6, 101.0, 57.9, 42.1, 39.5, 35.2, 30.2, 24.7.

LRMS (ESI) *m/z* calcd for $[C_{34}H_{36}N_{10}O_2 + H]^+$ = 617.3, found 617.3.

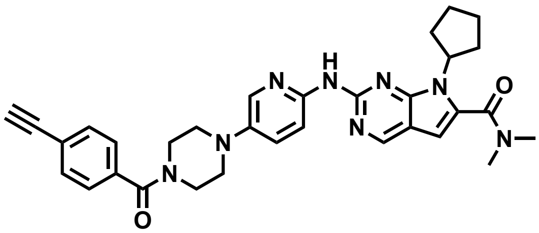

EST1026

General Procedure A was followed with 4-Ethynylbenzoic acid (8.4 mg, 0.06 mmol), HATU (43.8 mg, 0.12 mmol), DIPEA (0.04 mL, 0.23 mmol), and ribociclib (25.0 mg, 0.06 mmol). The crude residue was purified by silica gel chromatography (0-15% MeOH in DCM) to afford 13.6 mg (42%) of the title compound as a yellow film.

^{1}H NMR (500 MHz, CDCl3) δ 8.70 (s, 1H), 8.35 (d, J = 9.1 Hz, 1H), 8.16 (s, 1H), 7.99 (d, J = 2.9 Hz, 1H), 7.58 - 7.53 (m, 2H), 7.44 - 7.39 (m, 2H), 7.35 (dd, J = 9.1, 3.0 Hz, 1H), 6.45 (s, 1H), 4.79 (p, J = 9.0, 9.0, 9.0, 9.0 Hz, 1H), 3.93 (s, 2H), 3.61 (s, 2H), 3.16 (d, J = 3.9 Hz, 10H), 2.61 (s, 1H), 2.56 (dt, J = 16.4, 8.3, 8.3 Hz, 2H), 2.11 - 2.01 (m, 4H), 1.74 - 1.68 (m, 2H).

^{13}C NMR (151 MHz, $CDCl_3$) δ 169.7, 164.0, 154.2, 151.9, 151.6, 147.4, 142.1, 135.6, 132.3, 128.9, 127.2, 123.9, 112.9, 112.7, 101.0, 82.8, 78.8, 57.9, 47.6, 39.4, 35.2, 31.1, 30.2, 29.7, 24.8.

LRMS (ESI) *m/z* calcd for $[C_{32}H_{34}N_8O_2 + H]^+$ = 563.3, found 563.3.

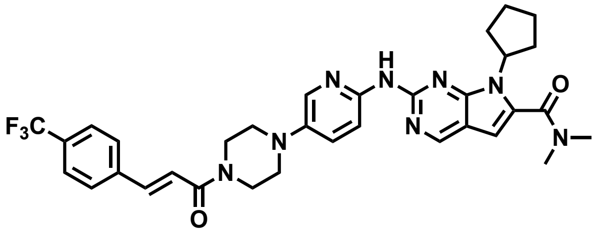

EST1027

General Procedure A was followed with *trans*-4-(trifluoromethyl)cinnamic acid (12.4 mg, 0.06 mmol), HATU (43.8 mg, 0.12 mmol), DIPEA (0.04 mL, 0.23 mmol), and ribociclib (25.0 mg, 0.06 mmol). The crude residue was purified by silica gel chromatography (0-15% MeOH in DCM) to afford 24.7 mg (68%) of the title compound as a yellow film.

^{1}H NMR (500 MHz, $CDCl_3$) δ 8.73 (s, 1H), 8.17 (s, 1H), 8.00 (d, *J* = 2.9 Hz, 1H), 7.71 (d, *J* = 15.4 Hz, 1H), 7.64 (s, 4H), 7.44 – 7.37 (m, 1H), 7.01 (d, *J* = 15.5 Hz, 1H), 6.46 (s, 1H), 4.77 (q, *J* = 8.9 Hz, 1H), 3.99 – 3.78 (m, 4H), 3.19 (br s, 4H), 3.15 (s, 6H), 2.53 (m, 2H), 2.10 – 1.97 (m, 4H), 1.69 (m, 2H).

^{13}C NMR (126 MHz, $CDCl_3$) δ 164.9, 164.1, 154.8, 152.1, 152.0, 148.0, 141.9, 141.3, 138.6, 137.7, 131.9, 131.2 (q, $^4J_{CF}$ = 32.5 Hz), 127.9, 127.3, 125.8 (q, $^3J_{CF}$ = 3.7 Hz), 125.0, 122.8 (q, $^1J_{CF}$ = 272.2 Hz), 119.4, 112.5, 101.1, 57.9, 50.9, 50.3, 45.9, 42.2, 39.4, 35.2, 30.1, 24.7.

HRMS (ESI) *m/z* calcd for $[C_{33}H_{35}F_3N_8O_2 + H]^+$ = 633.2835, found 633.2906.

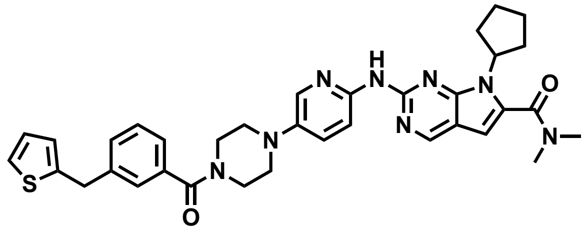

EST1030

General Procedure A was followed with 4-(2-thienylmethyl)benzoic acid (12.6 mg, 0.06 mmol), HATU (43.8 mg, 0.12 mmol), DIPEA (0.04 mL, 0.23 mmol), and ribociclib (25.0 mg, 0.06 mmol). The crude residue was purified by silica gel chromatography (0-15% MeOH in DCM) to afford 32.3 mg (88%) of the title compound as a yellow film.

^{1}H NMR (500 MHz, $CDCl_3$) δ 8.65 (s, 1H), 8.35 (d, *J* = 9.1 Hz, 1H), 8.30 (s, 1H), 7.89 (d, *J* = 2.9 Hz, 1H), 7.35 – 7.28 (m, 3H), 7.27 – 7.21 (m, 2H), 7.10 (dd, *J* = 5.2, 1.2 Hz, 1H), 6.87 (dd, *J* = 5.2, 3.4 Hz, 1H), 6.75 (dd, *J* = 3.3,

1.2 Hz, 1H), 6.38 (s, 1H), 4.73 (p, J = 8.8 Hz, 1H), 4.12 (s, 2H), 3.94 – 3.50 (m, 4H), 3.08 (s, 10H), 2.54 – 2.43 (m, 2H), 2.06 – 1.91 (m, 4H), 1.70 – 1.60 (m, 2H).

^{13}C NMR (126 MHz, $CDCl_3$) δ 170.4, 164.0, 154.4, 151.9, 151.7, 147.5, 143.0, 142.5, 142.1, 133.6, 132.3, 128.8, 127.8, 127.6, 126.9, 125.5, 124.2, 112.8, 112.7, 101.0, 57.9, 50.6, 39.5, 35.8, 30.2, 24.7.

LRMS (ESI) *m/z* calcd for $[C_{35}H_{38}N_8O_2S + H]^+$ = 635.3, found 635.4.

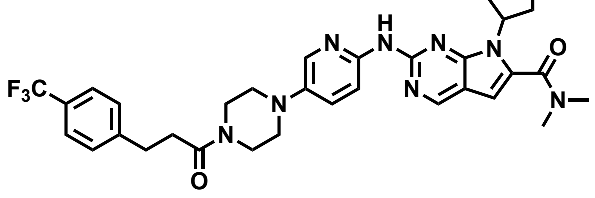

EST1036

General Procedure A was followed with 4-(trifluoromethyl)hydrocinnamic acid (99,5 mg, 0.46 mmol), HATU (350.2 mg, 0.92 mmol), DIPEA (0.32 mL, 1.4 mmol), and ribociclib (200.1 mg, 0.46 mmol). The crude residue was purified by reverse phase silica gel chromatography (5-95% MeCN in H_2O) to afford 133.0 mg (46%) of the title compound as a yellow film.

^{1}H NMR (500 MHz, $CDCl_3$) δ 9.93 (s, 1H), 8.76 (s, 1H), 7.79 (s, 1H), 7.54 (d, J = 8.2 Hz, 2H), 7.47 (d, J = 7.9 Hz, 2H), 7.32 (d, J = 7.9 Hz, 2H), 6.48 (s, 1H), 4.73 (p, J = 8.8 Hz, 1H), 3.74 (t, J = 5.0 Hz, 2H), 3.58 (t, J = 4.6 Hz, 2H), 3.12 (d, J = 13.6 Hz, 6H), 3.08 – 3.00 (m, 6H), 2.71 (t, J = 7.5 Hz, 2H), 2.38 (m, 2H), 2.05 – 1.91 (m, 4H), 1.64 – 1.56 (m, 2H).

^{13}C NMR (126 MHz, $CDCl_3$) δ 170.1, 164.1, 154.7, 152.0, 151.9, 147.9, 145.4, 141.9, 137.4, 132.0, 128.9, 128.4 (q, $^2J_{CF}$ = 32.2 Hz), 127.5, 127.4, 125.4 (q, $^3J_{CF}$ = 3.7 Hz), 124.3 (q, $^1J_{CF}$ = 271.8 Hz), 112.5, 101.1, 57.9, 50.6, 50.3, 45.4, 41.6, 39.4, 35.2, 34.4, 31.0, 30.1, 24.7.

HRMS (ESI) *m/z* calcd for $[C_{33}H_{37}F_3N_8O_2 + H]^+$ = 635.2992, found 635.3061.

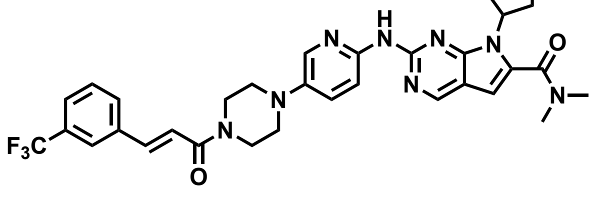

EST1051

General Procedure A was followed with 3-(trifluoromethyl)cinnamic acid (12.4 mg, 0.06 mmol), HATU (43.8 mg, 0.12 mmol), DIPEA (0.04 mL, 0.23 mmol), and ribociclib (25.0 mg, 0.06 mmol). The crude residue was purified by silica gel chromatography (0-10% MeOH in DCM) to afford 30.3 mg (83%) of the title compound as a yellow film.

^{1}H NMR (500 MHz, CDCl3) δ 8.65 (s, 1H), 8.32 (d, J = 9.0 Hz, 1H), 8.31 (s, 1H), 8.12 (s, 1H), 7.97 (d, J = 2.9 Hz, 1H), 7.72 (d, J = 2.1 Hz, 1H), 7.66 (d, J = 15.4 Hz, 1H), 7.62 (d, J = 7.7 Hz, 1H), 7.55 (d, J = 7.8 Hz, 1H), 7.45 (t, J = 7.8, 7.8 Hz, 1H), 7.29 (dd, J = 9.1, 3.0 Hz, 1H), 6.92 (d, J = 15.4 Hz, 1H), 6.38 (s, 1H), 4.73 (p, J = 9.0, 9.0, 8.9, 8.9 Hz, 1H), 3.91 - 3.76 (m, 4H), 3.13 (t, J = 5.0, 5.0 Hz, 4H), 3.09 (s, 6H), 2.57 - 2.46 (m, 2H), 2.05 - 1.95 (m, 4H), 1.69 - 1.62 (m, 2H).

^{13}C NMR (126 MHz, $CDCl_3$) δ 164.9, 164.0, 154.2, 151.9, 151.6, 147.3, 142.0, 141.6, 136.0, 132.5, 131.4 (q, $^2J_{CF}$ = 31.7 Hz), 131.3, 129.5, 126.2 (q, $^3J_{CF}$ = 3.7 Hz), 123.9 (q, $^3J_{CF}$ = 3.8 Hz), 122.8 (q, $^1J_{CF}$ = 272.6 Hz), 118.7, 113.0, 113.0, 101.0, 57.9, 50.3, 45.9, 42.2, 39.5, 35.2, 30.3, 24.8.

HRMS (ESI) *m/z* calcd for $[C_{33}H_{35}F_3N_8O_2 + H]^+$ = 633.2835, found 633.2906.

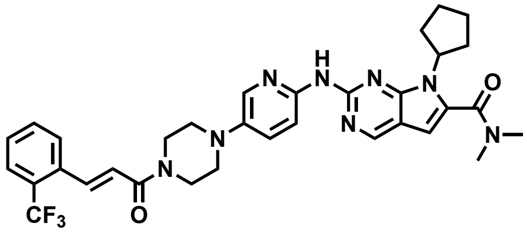

EST1054

General Procedure A was followed with *trans*-2-(trifluoromethyl)cinnamic acid (12.4 mg, 0.06 mmol), HATU (43.8 mg, 0.12 mmol), DIPEA (0.04 mL, 0.23 mmol), and ribociclib (25.0 mg, 0.06 mmol). The crude residue was purified by silica gel chromatography (0-10% MeOH in DCM) to afford 26.1 mg (72%) of the title compound as a yellow film.

^{1}H NMR (500 MHz, $CDCl_3$) δ 8.65 (s, 1H), 8.36 (d, *J* = 9.2 Hz, 1H), 8.30 (s, 1H), 7.96 – 7.87 (m, 2H), 7.66 – 7.60 (m, 2H), 7.50 (t, *J* = 7.6 Hz, 1H), 7.43 – 7.32 (m, 2H), 6.76 (d, *J* = 15.3 Hz, 1H), 6.39 (s, 1H), 4.73 (p, *J* = 8.7 Hz, 1H), 3.92 – 3.71 (m, 4H), 3.12 (t, *J* = 5.1 Hz, 4H), 3.08 (s, 6H), 2.48 (m, 2H), 2.06 – 1.95 (m, 4H), 1.71 – 1.59 (m, 2H).

^{13}**C NMR** (126 MHz, $CDCl_3$) δ 165.0, 164.0, 154.2, 151.9, 151.6, 147.4, 142.0, 138.4, 134.4, 132.4, 132.0, 129.1, 128.5 (q, $^{2}J_{CF}$ = 30.3 Hz), 128.0, 126.2 (q, $^{3}J_{CF}$ = 5.5 Hz), 122.9 (d, $^{1}J_{CF}$ = 274.0 Hz), 121.9, 112.9, 112.9, 101.0, 57.9, 50.2, 46.0, 42.1, 39.5, 35.2, 30.3, 24.8.

HRMS (ESI) *m/z* calcd for $[C_{33}H_{35}F_3N_8O_2 + H]^+$ = 633.2835, found 633.2906.

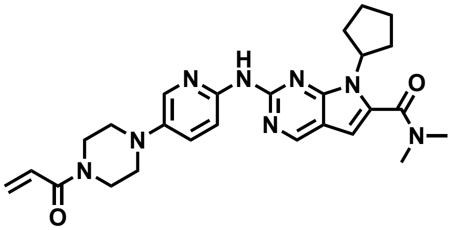

EST1057

General Procedure A was followed with acrylic acid (0.004 mL, 0.06 mmol), HATU (43.8 mg, 0.12 mmol), DIPEA (0.04 mL, 0.23 mmol), and ribociclib (25.0 mg, 0.06 mmol). The crude residue was purified by silica gel chromatography (0-10% MeOH in DCM) to afford 15.2 mg (54%) of the title compound as a yellow film.

^{1}**H NMR** (500 MHz, $CDCl_3$) δ 8.7 (s, 1H), 7.9 (br s, 2H), 7.6 – 7.4 (m, 1H), 6.6 (dd, *J* = 16.8, 10.6 Hz, 1H), 6.5 (s, 1H), 6.2 (d, *J* = 16.8 Hz, 1H), 5.7 (d, *J* = 10.6 Hz, 1H), 4.7 (p, *J* = 9.0 Hz, 1H), 3.8 (t, *J* = 5.2 Hz, 2H), 3.7 (q, *J* = 9.4 Hz, 2H), 3.1 – 3.1 (m, 8H), 3.0 – 2.9 (m, 2H), 2.4 (p, *J* = 8.3 Hz, 2H), 2.0 – 2.0 (m, 2H), 2.0 (q, *J* = 7.7 Hz, 2H), 1.6 (h, *J* = 8.0 Hz, 2H).

^{13}**C NMR** (126 MHz, $CDCl_3$) δ 165.4, 164.0, 154.4, 151.9, 151.8, 147.5, 142.1, 132.2, 128.3, 127.8, 127.3, 112.8, 112.7, 101.0, 57.9, 50.8, 50.3, 45.8, 41.9, 39.5, 30.2, 24.8.

LRMS (ESI) *m/z* calcd for $[C_{26}H_{32}N_8O_2 + H]^+$ = 489.3, found 489.4.

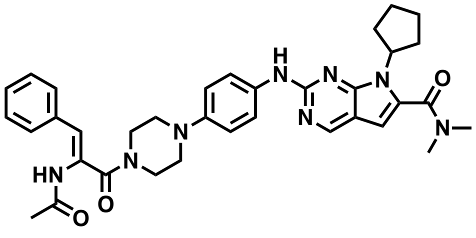

EST1059

General Procedure A was followed with 2-(acetylamino)-3-phenyl-2-propenoic acid (12.7 mg, 0.06 mmol), HATU (43.8 mg, 0.12 mmol), DIPEA (0.04 mL, 0.23 mmol), and ribociclib (25.0 mg, 0.06 mmol). The crude residue was purified by silica gel chromatography (0-10% MeOH in DCM) to afford 34.2 mg (96%) of the title compound as a yellow film.

^{1}H NMR (700 MHz, DMSO) δ 10.84 (s, 1H), 10.11 (s, 1H), 8.93 (s, 1H), 7.95 (d, *J* = 2.6 Hz, 2H), 7.75 (d, *J* = 8.9 Hz, 1H), 7.68 (d, *J* = 8.4 Hz, 2H), 7.63 (d, *J* = 8.3 Hz, 2H), 7.49 (d, *J* = 15.2 Hz, 1H), 7.23 (d, *J* = 15.3 Hz, 1H), 6.76 (s, 1H), 4.80 (p, *J* = 8.9 Hz, 1H), 3.91 (s, 2H), 3.77 (s, 2H), 3.22 (s, 4H), 3.06 (s, 6H), 2.40 – 2.31 (m, 2H), 2.07 (s, 3H), 2.05 – 2.00 (m, 2H), 1.99 – 1.93 (m, 2H), 1.66 (m, 2H).

^{13}C NMR (151 MHz, DMSO) δ 169.0, 165.2, 162.9, 153.0, 152.0, 149.7, 145.1, 142.4, 142.1, 141.1, 134.9, 130.2, 129.3, 119.3, 116.5, 115.0, 113.9, 101.4, 79.6, 57.5, 49.5, 49.1, 48.8, 45.1, 41.7, 39.2, 35.0, 30.5, 24.7, 24.5.

LRMS (ESI) *m/z* calcd for $[C_{35}H_{40}N_8O_3 + H]^+$ = 621.8, found 622.3.

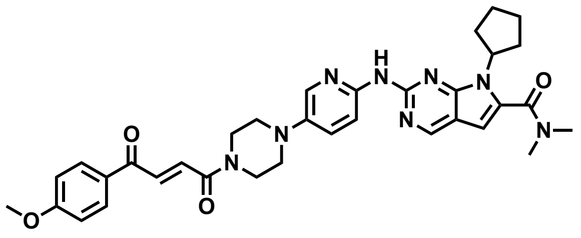

EST1060

General Procedure D was followed with *trans*-3-(4-methoxybenzoyl)acrylic acid (99.6 mg, 0.48 mmol), T3P (0.46 mL, 0.72 mmol), DIPEA (0.25 mL, 1.5 mmol), and ribociclib (216.0 mg, 0.57 mmol). The crude residue was purified by silica gel chromatography (0-10% MeOH in DCM). The resultant dark yellow oil was triturated in diethyl ether to afford 199.3 mg (83% - *E:Z* = >99:<1) of the title compound as an orange powder.

^{1}H NMR (500 MHz, $CDCl_3$) δ 8.75 (s, 2H), 8.28 (d, *J* = 8.1 Hz, 1H), 8.06 – 8.01 (m, 3H), 7.98 (d, *J* = 14.9 Hz, 1H), 7.50 (d, *J* = 15.0 Hz, 1H), 7.36 (dd, *J* = 9.1, 2.9 Hz, 1H), 7.00 – 6.93 (m, 2H), 6.45 (s, 1H), 4.78 (p, *J* = 8.9 Hz, 1H), 3.92 (t, *J* = 5.2 Hz, 2H), 3.88 (s, 3H), 3.82 (t, *J* = 5.1 Hz, 2H), 3.17 (t, *J* = 5.2 Hz, 4H), 3.14 (s, 6H), 2.60 – 2.49 (m, 2H), 2.11 – 1.97 (m, 4H), 1.73 – 1.63 (m, 2H).

^{13}C NMR (126 MHz, $CDCl_3$) δ 187.7, 164.2, 164.1, 164.1, 154.5, 151.9, 151.8, 147.7, 141.9, 134.7, 132.1, 131.3, 131.3, 130.0, 127.6, 114.1, 112.7, 112.5, 101.0, 57.9, 55.6, 50.9, 50.3, 46.0, 42.2, 39.5, 35.2 30.2, 24.7.

HRMS (ESI) *m/z* calcd for $[C_{34}H_{38}N_8O_4 + H]^+$ = 623.3016, found 623.3086.

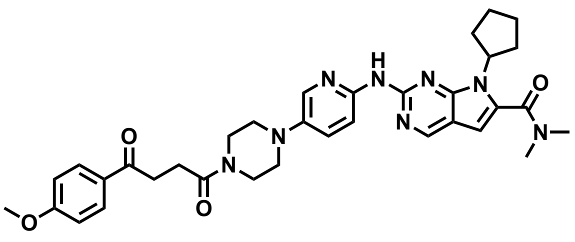

JP-2-230

A modified **General Procedure A** was followed with 3-(4-Methoxybenzoyl)propionic acid (15.9 mg, 0.08 mmol), HATU (25.5 mg, 0.07 mmol), DIPEA (0.04 mL, 0.23 mmol), and ribociclib (27.6 mg, 0.06 mmol). A precipitate had formed during the reaction which was vacuum filtered and washed with hexanes. The yellow precipitate was dissolved in DCM and dry loaded onto silica gel and was purified by silica gel chromatography (0-7% MeOH in DCM) to afford 28.9 mg (73%) of the title compound as a yellow-white powder.

^{1}H NMR (700 MHz, $CDCl_3$) δ 8.74 (s, 1H), 8.43 (s, 1H), 8.38 (d, *J* = 9.0 Hz, 1H), 8.07 (d, *J* = 2.9 Hz, 1H), 8.02 – 7.98 (m, 2H), 7.33 (dd, *J* = 9.1, 2.9 Hz, 1H), 6.95 – 6.91 (m, 2H), 6.44 (s, 1H), 4.79 (p, *J* = 8.9 Hz, 1H), 3.86 (s, 3H), 3.81 (t, *J* = 5.1 Hz, 2H), 3.76 (t, *J* = 5.1 Hz, 2H), 3.34 (t, *J* = 6.6 Hz, 2H), 3.18 (t, *J* = 5.1 Hz, 2H), 3.15 (s, 6H), 3.11 (t, *J* = 5.2 Hz, 2H), 2.82 (t, *J* = 6.6 Hz, 2H), 2.62 – 2.54 (m, 2H), 2.11 – 2.00 (m, 4H), 1.75 – 1.67 (m, 2H).

^{13}C NMR (151 MHz, $CDCl_3$) δ 197.6, 170.4, 164.1, 163.5, 154.6, 152.0, 151.9, 147.6, 142.1, 137.5, 132.0, 130.4, 129.9, 127.3, 113.7, 112.6, 112.4, 101.0, 57.9, 55.5, 50.6, 50.3, 45.4, 41.7, 39.5, 35.2, 33.2, 30.2, 27.1, 24.7.

HRMS (ESI) *m/z* calcd for $[C_{34}H_{40}N_8O_4 + H]^+$ = 625.3173, found 625.3242.

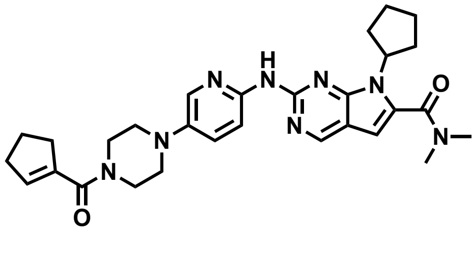

EST1061

General Procedure A was followed with 1-cyclopentenecarboxylic acid (6.5 mg, 0.06 mmol), HATU (43.8 mg, 0.12 mmol), DIPEA (0.04 mL, 0.23 mmol), and ribociclib (25.0 mg, 0.06 mmol). The crude residue was purified by silica gel chromatography (0-10% MeOH in DCM) to afford 23.3 mg (77%) of the title compound as a yellow powder.

^{1}H NMR (500 MHz, $CDCl_3$) δ 8.75 (s, 1H), 8.02 (s, 1H), 7.94 (d, *J* = 2.9 Hz, 1H), 7.50 – 7.42 (m, 1H), 6.47 (s, 1H), 5.93 (p, *J* = 2.2 Hz, 1H), 4.77 (p, *J* = 8.9 Hz, 1H), 3.78 (s, 4H), 3.21 – 3.08 (m, 10H), 2.63 (tq, *J* = 7.5, 2.4 Hz, 2H), 2.54 – 2.42 (m, 4H), 2.12 – 1.99 (m, 4H), 1.96 (p, *J* = 7.6 Hz, 2H), 1.67 (h, *J* = 8.4 Hz, 2H).

^{13}C NMR (151 MHz, $CDCl_3$) δ 168.6, 163.9, 154.1, 152.0, 151.4, 147.1, 142.2, 138.1, 133.1, 132.7, 113.0, 101.0, 58.0, 53.8, 50.5, 39.5, 35.2, 34.5, 33.3, 30.3, 24.8, 22.9, 18.7, 17.5.

LRMS (ESI) *m/z* calcd for $[C_{29}H_{36}N_8O_2 + H]^+$ = 529.3, found 529.4.

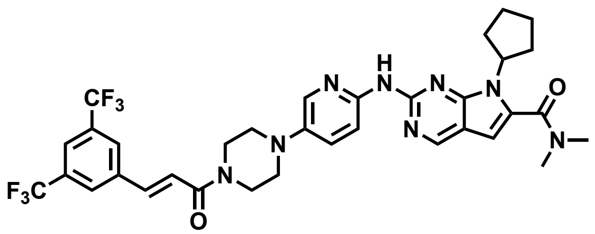

KN1002

General Procedure A was followed with *trans*-3,5-bis(trifluoromethyl)cinnamic acid (14.6 mg, 0.06 mmol), HATU (43.8 mg, 0.12 mmol), DIPEA (0.04 mL, 0.23 mmol), and ribociclib (25.0 mg, 0.06 mmol). The crude residue was purified by silica gel chromatography (0-10% MeOH in DCM) to afford 14.5 mg (40%) of the title compound as a yellow film.

^{1}H NMR (500 MHz, $CDCl_3$) δ 8.65 (s, 1H), 8.28 (s, 1H), 7.97 (d, *J* = 3.0 Hz, 2H), 7.87 (s, 2H), 7.79 (s, 1H), 7.68 (d, *J* = 15.4 Hz, 1H), 7.30 (dd, *J* = 9.2, 2.9 Hz, 1H), 6.99 (d, *J* = 15.4 Hz, 1H), 6.38 (s, 1H), 4.73 (t, *J* = 8.9 Hz, 1H), 3.88 (s, 2H), 3.82 (d, *J* = 13.7 Hz, 2H), 3.13 (d, *J* = 8.6 Hz, 4H), 3.09 (s, 6H), 2.50 (dq, *J* = 15.6, 7.3 Hz, 2H), 2.06 – 1.92 (m, 4H), 1.65 (dt, *J* = 12.6, 5.1 Hz, 2H).

^{13}C NMR (126 MHz, $CDCl_3$) δ 164.4, 164.2, 154.4, 151.9, 151.8, 147.6, 142.0, 139.9, 137.5, 137.2, 132.4 (q, $^{2}J_{CF}$ = 33.4 Hz), 132.1, 127.5, 126.3, 124.1, 122.9 (q, $^{1}J_{CF}$ = 273.0 Hz), 122.0, 120.7, 119.8, 112.7, 112.6, 112.6, 101.1, 57.9, 50.8, 50.3, 49.9, 49.8, 49.6, 49.4, 49.3, 45.9, 42.3, 39.5, 35.2, 30.1, 24.7.

LRMS (ESI) *m/z* calcd for $[C_{34}H_{34}F_6N_8O_2 + H]^+$ = 701.3, found 701.4.

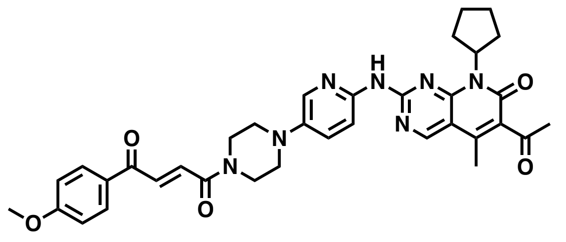

EST1089

General Procedure A was followed with 4-methoxycinnamic acid (13.8 mg, 0.06 mmol), HATU (42.5 mg, 0.11 mmol), DIPEA (0.04 mL, 0.23 mmol), and palbociclib (25.0 mg, 0.06 mmol). The crude residue was purified by silica gel chromatography (0-10% MeOH in DCM) to afford 26.1 mg (74% - *E:Z* = >99:<1) of the title compound as a yellow film.

^{1}H NMR (500 MHz, $CDCl_3$) δ 8.86 (s, 1H), 8.22 (d, *J* = 9.0 Hz, 1H), 8.09 (d, *J* = 2.9 Hz, 1H), 8.08 – 8.02 (m, 2H), 8.00 (d, *J* = 14.9 Hz, 1H), 7.52 (d, *J* = 14.8 Hz, 1H), 7.35 (dd, *J* = 9.1, 3.0 Hz, 1H), 7.01 – 6.95 (m, 2H), 5.88 (p, *J* = 8.9 Hz, 1H), 3.94 (t, *J* = 5.2 Hz, 2H), 3.89 (s, 3H), 3.85 (t, *J* = 5.1 Hz, 2H), 3.22 (t, *J* = 5.2 Hz, 4H), 2.54 (s, 3H), 2.38 (s, 3H), 2.36 – 2.30 (m, 1H), 2.12 – 2.01 (m, 2H), 1.89 (dddd, *J* = 15.4, 13.0, 7.6, 4.7 Hz, 2H), 1.75 – 1.64 (m, 3H).

^{13}C NMR (151 MHz, $CDCl_3$) δ 202.6, 187.6, 164.3, 164.1, 161.4, 158.0, 157.2, 155.5, 145.8, 143.0, 141.7, 137.3, 134.9, 131.3, 131.1, 130.9, 129.9, 126.9, 114.2, 113.6, 107.9, 55.6, 54.1, 53.4, 50.4, 49.8, 45.8, 42.1, 31.5, 28.1, 25.8, 22.7, 14.0.

HRMS (ESI) *m/z* calcd for $[C_{35}H_{37}N_7O_5 + H]^+$ = 636.2929, found 636.2935.

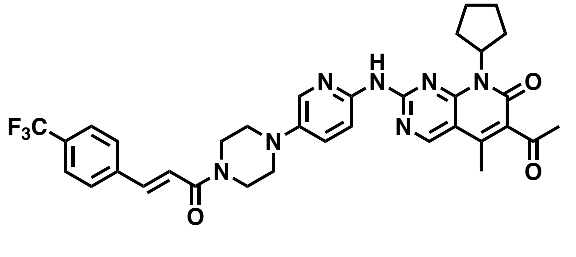

EST1090

General Procedure A was followed with *trans*-4-(trifluoromethyl)cinnamic acid (14.5 mg, 0.07 mmol), HATU (42.5 mg, 0.11 mmol), DIPEA (0.04 mL, 0.23 mmol), and palbociclib (0.25, 0.06 mmol). The crude residue was purified by silica gel chromatography (0-100% EtOAc in Hexanes) to afford 22.8 mg (63%) of the title compound as a yellow film.

^{1}H NMR (500 MHz, $CDCl_3$) δ 8.87 (s, 1H), 8.74 (s, 1H), 8.22 (d, *J* = 9.1 Hz, 1H), 8.09 (d, *J* = 2.9 Hz, 1H), 7.72 (d, *J* = 15.4 Hz, 1H), 7.64 (s, 4H), 7.37 (dd, *J* = 9.1, 3.0 Hz, 1H), 7.00 (d, *J* = 15.5 Hz, 1H), 5.88 (p, *J* = 8.9 Hz, 1H), 4.01 – 3.81 (m, 4H), 3.23 (t, *J* = 5.1 Hz, 4H), 2.54 (s, 3H), 2.38 (s, 3H), 2.37 – 2.31 (m, 2H), 2.07 (tdd, *J* = 11.9, 9.9, 5.2 Hz, 2H), 1.92 – 1.84 (m, 2H), 1.69 (tdd, *J* = 10.9, 6.6, 4.0 Hz, 2H).

^{13}C NMR (126 MHz, $CDCl_3$) δ 202.6, 164.9, 161.4, 158.0, 157.2, 155.6, 145.8, 143.1, 141.8, 141.6, 138.5 (d, $^5J_{CF}$ = 1.4 Hz) , 137.1, 131.4 (q, $^2J_{CF}$ = 32.7 Hz), 130.9, 128.0, 126.9, 125.8 (q, $^3J_{CF}$ = 3.8 Hz), 123.9 (d, $^1J_{CF}$ = 272.1 Hz), 119.2, 113.7, 107.9, 54.1, 50.3, 49.8, 45.7, 42.1, 31.5, 28.1, 25.8, 14.0.

HRMS (ESI) *m/z* calcd for $[C_{34}H_{34}F_3N_7O_3 + H]^+$ = 646.2748, found 646.2741.

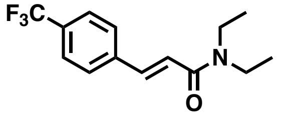

KN1026

General Procedure A was followed with *trans*-4-(trifluoromethyl)cinnamic acid (100.0 mg, 0.06 mmol), HATU (43.8 mg, 0.12 mmol), DIPEA (0.04 mL, 0.23 mmol), and *N,N*-diethylamine (0.05, 0.48 mmol). The crude residue was purified by silica gel chromatography (0-60% EtOAC in Hexanes) to afford 66.0 mg (50%) of the title compound as a yellow film.

^{1}H NMR (500 MHz, $CDCl_3$) δ 7.63 (d, *J* = 15.4 Hz, 1H), 7.54 (s, 4H), 6.83 (d, *J* = 15.4 Hz, 1H), 3.42 (dq, *J* = 9.8, 7.1 Hz, 4H), 1.19 (t, *J* = 7.2 Hz, 3H), 1.12 (t, *J* = 7.1 Hz, 3H).

^{13}C NMR (126 MHz, $CDCl_3$) δ 165.1, 140.5, 138.9 (d, $^5J_{CF}$ = 1.4 Hz), 131.0 (q, $^2J_{CF}$ = 32.5 Hz), 127.9, 125.7 (q, $^3J_{CF}$ = 3.8 Hz), 123.9 (q, $^1J_{CF}$ = 272.1 Hz), 120.3, 42.4, 41.2, 15.1, 13.1.

LRMS (ESI) *m/z* calcd for $[C_{14}H_{16}F_3NO + H]^+$ = 272.1, found 272.2.

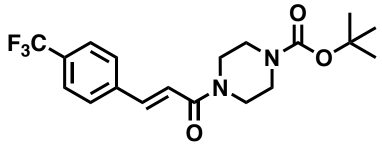

EST1096

General Procedure A was followed with *trans*-4-(trifluoromethyl)cinnamic acid (100.0 mg, 0.46 mmol), HATU (43.8 mg, 0.12 mmol), DIPEA (0.04 mL, 0.23 mmol), and 1-boc-piperazine (94.7 mg, 0.51 mmol). The crude residue was purified by silica gel chromatography (0-70% EtOAc in Hexanes) to afford 72.5 mg (41%) of the title compound as a white powder.

^{1}H NMR (500 MHz, $CDCl_3$) δ 7.62 (d, J = 15.4 Hz, 1H), 7.55 (s, 4H), 6.87 (d, J = 15.4 Hz, 1H), 3.60 (s, 4H), 3.42 (dd, J = 6.6, 3.9 Hz, 4H), 1.41 (s, 9H).

^{13}C NMR (151 MHz, $CDCl_3$) δ 165.0, 154.5, 141.4, 138.5, 131.3 (q, $^2J_{CF}$ = 32.6 Hz), 127.9, 125.8 (q, $^3J_{CF}$ = 3.7 Hz), 123.9 (q, $^1J_{CF}$ = 272.3 Hz), 119.3, 80.4, 45.7, 42.0, 28.4.

LRMS (ESI) *m/z* calcd for $[C_{19}H_{23}F_3N_2O_3 + Na]^+$ = 407.2, found 407.2.

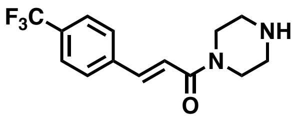

EST1102

General Procedure E was followed with **EST1096** (47.3 mg, 0.12 mmol), and TFA (0.3 mL, 3.94 mmol) for 2 hours. The crude residue was purified by silica gel chromatography (0-17% MeOH in DCM) using a Biotage® Sfär KP-Amino D cartridge to afford 14.5 mg (41%) of the title compound as a white film.

^{1}H NMR (500 MHz, $CDCl_3$) δ 7.64 (d, J = 15.5 Hz, 1H), 7.59 (s, 4H), 6.93 (d, J = 15.4 Hz, 1H), 3.72 – 3.66 (m, 2H), 3.64 – 3.58 (m, 2H), 2.89 (t, J = 5.0, 5.0 Hz, 4H), 1.80 (s, 1H).

^{13}C NMR (126 MHz, $CDCl_3$) δ 164.9, 141.3, 138.6, 131.2 (q, $^2J_{CF}$ = 32.6 Hz), 127.9, 125.8 (q, $^3J_{CF}$ = 3.8 Hz), 125.0, 122.8 (q, $^1J_{CF}$ = 272.0 Hz), 119.4, 46.5, 46.1, 45.5, 42.7.

LRMS (ESI) *m/z* calcd for $[C_{14}H_{15}F_3N_2O + H]+$ = 285.1, found 285.2.

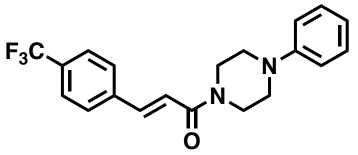

KN1023

General Procedure A was followed with *trans*-4-(trifluoromethyl)cinnamic acid (200.0 mg, 0.93 mmol), HATU (703.6 mg, 1.85 mmol), DIPEA (0.64 mL, 3.7 mmol), and 1-phenylpiperazine (150.1 mg, 0.93 mmol). The crude residue was purified by silica gel chromatography (15-55% EtOAc in Hexanes) to afford 125.0 mg (37%) of the title compound as a yellow powder.

^{1}H NMR (500 MHz, $CDCl_3$) δ 7.62 (d, J = 15.4 Hz, 1H), 7.53 (s, 4H), 7.23 – 7.16 (m, 2H), 6.91 (d, J = 15.4 Hz, 1H), 6.83 (dd, J = 16.5, 7.9 Hz, 3H), 3.80 (br s, 2H), 3.72 (br s, 2H), 3.13 (m, 4H).

^{13}C NMR (126 MHz, $CDCl_3$) δ 164.9, 150.9, 141.3, 138.7, 131.2 (q, $^2J_{CF}$ = 32.5 Hz), 129.3, 128.0, 125.8 (q, $^3J_{CF}$ = 3.8 Hz), 124.0 (q, $^1J_{CF}$ = 272.2 Hz), 120.6, 119.6, 116.7, 49.9, 49.4, 45.9, 42.2.

LRMS (ESI) *m/z* calcd for $[C_{20}H_{19}F_3N_2O + H]^+$ = 361.1, found 361.2.

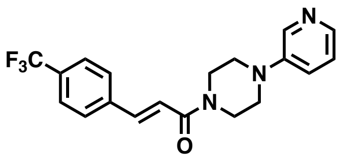

KN1017

General Procedure A was followed with *trans*-4-(trifluoromethyl)cinnamic acid (200.0 mg, 0.93 mmol), HATU (703.6 mg, 1.85 mmol), DIPEA (0.64 mL, 3.7 mmol), and 1-(3-pyridinyl)piperazine (151.1 mg, 0.93 mmol). The crude residue was purified by silica gel chromatography (0-10% MeOH in DCM) to afford 53.1 mg (16%) of the title compound as a yellow powder.

^{1}H NMR (500 MHz, $CDCl_3$) δ 8.34 – 8.29 (m, 1H), 8.14 (dd, *J* = 3.9, 2.1 Hz, 1H), 7.70 (d, *J* = 15.4 Hz, 1H), 7.61 (s, 4H), 7.23 – 7.14 (m, 2H), 6.98 (d, *J* = 15.5 Hz, 1H), 3.90 (br s, 2H), 3.83 (br s, 2H), 3.25 (m, 4H).

^{13}C NMR (126 MHz, $CDCl_3$) δ 164.9, 146.5, 141.7, 141.5, 139.1, 138.5, 131.3 (q, $^2J_{CF}$ = 32.6 Hz), 128.0, 125.8 (q, $^3J_{CF}$ = 3.8 Hz), 123.9 (q, $^1J_{CF}$ = 272.2 Hz), 123.6, 123.1, 119.2, 49.2, 48.7, 45.6, 41.9.

LRMS (ESI) *m/z* calcd for $[C_{19}H_{18}F_3N_3O + H]^+$ = 362.1, found 362.1.

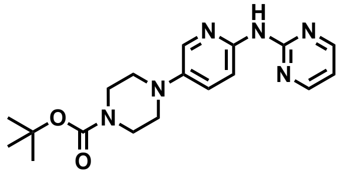

JP-2-195

A combination of 2-chloropyrimidine (100.0 mg, 0.95 mmol), tert-butyl 4-(6-aminopyridin-3-yl)piperazine-1-carboxylate (265.2 mg, 0.95 mmol), palladium (II) acetate (21.4 mg, 0.10 mmol), caesium carbonate (465.6 mg, 1.4 mmol), and Xantphos (82.7 mg, 0.14 mmol) was suspended in dioxane (10 mL) and heated to 120 °C overnight. The volatiles were removed *in vacuo*, and the crude residue was purified by silica gel chromatography (0-5% MeOH in DCM) to afford 282.1 mg (91%) of the title compound as a beige solid.

^{1}H NMR (700 MHz, $CDCl_3$) δ 8.50 (d, *J* = 4.8 Hz, 2H), 8.30 (d, *J* = 9.0 Hz, 1H), 8.19 (s, 1H), 8.07 (d, *J* = 2.9 Hz, 1H), 7.35 (dd, *J* = 9.1, 3.0 Hz, 1H), 6.78 (t, *J* = 4.8 Hz, 1H), 3.63 (t, *J* = 5.1 Hz, 4H), 3.11 (t, *J* = 5.2 Hz, 4H), 1.51 (s, 9H).

^{13}C NMR (151 MHz, $CDCl_3$) δ 159.3, 158.0, 154.7, 146.6, 143.0, 137.4, 127.2, 113.0, 112.9, 80.0, 50.1, 28.4.

LRMS (ESI) *m/z* calcd for $[C_{18}H_{24}N_6O_2 + H]^+$ = 375.2, found 375.3.

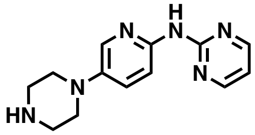

JP-2-198

General Procedure E was followed with **JP-2-195** (152.4 mg, 0.43 mmol), and TFA (1.1 mL, 13.7 mmol) for 2 hours. The crude material was used without further purification.

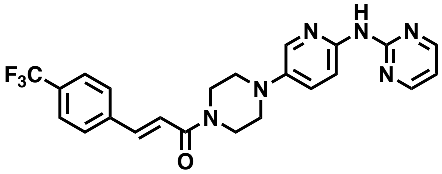

JP-2-200

General Procedure A was followed with *trans*-4-(trifluoromethyl)cinnamic acid (43.0 mg, 0.20 mmol), HATU (82.6 mg, 0.22 mmol), DIPEA (0.09 mL, 0.54 mmol), and **JP-2-198** (46.4 mg, 0.18 mmol). The crude residue was purified by silica gel chromatography (50-100% EtOAc in Hexanes followed by 0-5% MeOH in DCM) to afford 13.3 mg (16%, 2 steps) of the title compound as a yellow residue.

^{1}H NMR (700 MHz, $CDCl_3$) δ 8.48 (dd, J = 4.8, 1.6 Hz, 2H), 8.30 (d, J = 8.9 Hz, 1H), 8.14 (s, 1H), 8.05 (t, J = 2.2 Hz, 1H), 7.72 (dd, J = 15.5, 1.7 Hz, 1H), 7.64 (d, J = 1.7 Hz, 3H), 7.35 (dt, J = 9.1, 2.3 Hz, 1H), 7.00 (dd, J = 15.5, 1.6 Hz, 1H), 6.77 (td, J = 4.8, 1.7 Hz, 1H), 3.94 (br s, 2H), 3.85 (br s, 2H), 3.19 (m, 3H).

^{13}C NMR (126 MHz, $CDCl_3$) δ 164.9, 159.2, 158.0, 147.0, 142.5, 141.5, 138.6 (d, $^5J_{CF}$ = 1.3 Hz), 137.5, 131.3 (q, $^2J_{CF}$ = 32.8), 127.9, 127.4, 125.8 (q, $^3J_{CF}$ = 3.8 Hz), 123.9 (q, $^1J_{CF}$ = 272.2 Hz), 119.3, 113.1, 113.0, 50.7, 50.1, 45.8, 42.1, 29.7, 28.4.

LRMS (ESI) *m/z* calcd for $[C_{23}H_{21}F_3N_6O + H]^+$ = 455.2, found 455.2.

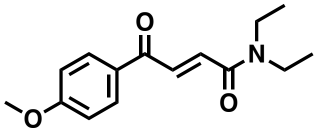

KN1025

General Procedure A was followed with *trans*-3-(4-methoxybenzoyl)acrylic acid (100.1 mg, 0.47 mmol), HATU (175.9 mg, 0.48 mmol), DIPEA (0.08 mL, 0.46 mmol), and *N,N*-diethylamine (25.0 mg, 0.06 mmol). The crude residue was purified by silica gel chromatography (0-55% EtOAc in Hexanes) to afford 16.3 mg (13%) of the title compound as a yellow film.

^{1}H NMR (700 MHz, $CDCl_3$) δ 8.06 – 8.04 (m, 2H), 7.99 (d, *J* = 14.8 Hz, 1H), 7.43 (d, *J* = 14.8 Hz, 1H), 6.99 – 6.95 (m, 2H), 3.89 (s, 3H), 3.52 – 3.45 (m, 4H), 1.24 (t, *J* = 7.2 Hz, 3H), 1.19 (t, *J* = 7.1 Hz, 3H).

^{13}C NMR (126 MHz, $CDCl_3$) δ 187.9, 164.5, 164.1, 134.0, 132.2, 131.3, 131.0, 130.1, 114.1, 55.6, 42.5, 41.2, 15.1, 13.0.

LRMS (ESI) *m/z* calcd for $[C_{15}H_{19}NO_3 + H]^+$ = 262.1, found 262.3.

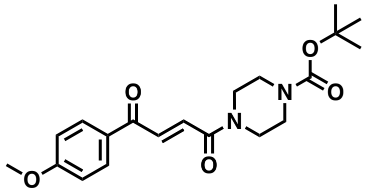

JP-2-190

General Procedure A was followed with *trans*-3-(4-methoxybenzoyl)acrylic acid (977 mg, 7.3 mmol), HATU (3.1 g, 8.1 mmol), DIPEA (3.8 mL, 21.8 mmol), and 1-boc-piperazine (1.5 g, 8.0 mmol). The crude residue was purified by silica gel chromatography (0-50% EtOAC in Hexanes) to afford 1.59 g (58%) of the title compound as a yellow solid.

^{1}H NMR (500 MHz, $CDCl_3$) δ 8.04 (d, *J* = 8.6 Hz, 2H), 7.96 (dd, *J* = 15.4, 3.9 Hz, 1H), 7.46 (d, *J* = 14.9 Hz, 1H), 6.98 (d, *J* = 8.7 Hz, 2H), 3.89 (s, 3H), 3.72 (t, *J* = 5.4, 5.4 Hz, 2H), 3.62 (t, *J* = 5.2, 5.2 Hz, 2H), 3.49 (t, *J* = 5.2, 5.2 Hz, 4H), 1.48 (s, 9H).

^{13}C NMR (126 MHz, $CDCl_3$) δ 187.6, 164.2, 154.5, 134.8, 131.3, 131.2, 131.1, 129.9, 114.1, 114.0, 80.5, 55.6, 45.9, 42.1, 28.4.

LRMS (ESI) *m/z* calcd for $[C_{20}H_{26}N_2O_5 + Na]^+$ = 397.2, found 397.2.

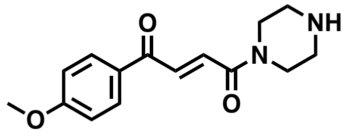

JP-2-196

General Procedure E was followed with **JP-2-190** (78.6 mg, 0.21 mmol), and TFA (0.51 mL, 6.7 mmol) for 1.5 hours. The reaction mixture was concentrated *in vacuo*, redissolved in DCM, and stirred with a saturated solution of $NaHCO_3$ (2 mL) for 30 min. The organic layer was separated, washed with brine, dried over Na_2SO_4, and concentrated *in vacuo*. The crude residue was purified by silica gel chromatography (0-12% MeOH in DCM) to afford 20.8 mg (81% - *E:Z* = 98.5:1.5) of the title compound as a white solid.

^{1}H NMR (700 MHz, $CDCl_3$) δ 7.99 (dd, *J* = 8.9, 1.9 Hz, 2H), 7.93 (dd, *J* = 14.9, 1.8 Hz, 1H), 7.37 (dd, *J* = 14.9, 1.8 Hz, 1H), 6.94 (dd, *J* = 9.0, 2.0 Hz, 2H), 3.97 (t, *J* = 5.4 Hz, 2H), 3.92 (t, *J* = 5.4 Hz, 2H), 3.85 (d, *J* = 1.9 Hz, 3H), 3.22 (t, *J* = 5.2 Hz, 4H), 2.73 (s, 1H).

^{13}C NMR (151 MHz, $CDCl_3$) δ 187.5, 164.5, 136.0, 131.5, 130.0, 129.7, 114.3, 55.7, 43.4, 43.1, 42.8, 38.9.

LRMS (ESI) *m/z* calcd for $[C_{15}H_{18}N_2O_3 + H]^+$ = 275.1, found 275.1.

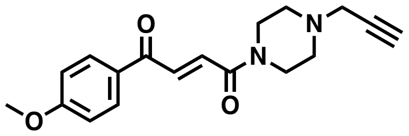

JP-2-253

A combination of **JP-2-196** (25.9 mg, 0.094 mmol) and potassium carbonate (15.7 mg, 0.11 mmol) was suspended in DMF (4 mL) and stirred at ambient temperature for 5 minutes. Propargyl bromide (0.01 mL, 0.13 mmol) was added, and the reaction mixture was heated to 90 °C overnight. The reaction mixture was concentrated *in vacuo*, and the crude residue was purified by silica gel chromatography (0-10% MeOH in DCM) to afford 11.7 mg (40% - *E:Z* = 96:4) of the title compound as a yellow oil.

^{1}H NMR (700 MHz, $CDCl_3$) δ 8.05 – 8.02 (m, 2H), 7.95 (d, *J* = 14.9 Hz, 1H), 7.47 (d, *J* = 14.9 Hz, 1H), 6.99 – 6.95 (m, 2H), 3.89 (s, 3H), 3.79 (t, *J* = 5.1 Hz, 2H), 3.68 (t, *J* = 5.1 Hz, 2H), 3.35 (d, *J* = 2.4 Hz, 2H), 2.60 (q, *J* = 4.6 Hz, 4H), 2.28 (d, *J* = 2.4 Hz, 1H).

^{13}C NMR (151 MHz, $CDCl_3$) δ 187.7, 164.2, 164.0, 134.4, 131.5, 131.3, 130.0, 114.1, 78.0, 73.8, 55.6, 51.9, 51.3, 46.8, 45.9, 42.1.

LRMS (ESI) *m/z* calcd for $[C_{18}H_{20}N_2O_3 + H]^+$ = 213.1, found 213.2.

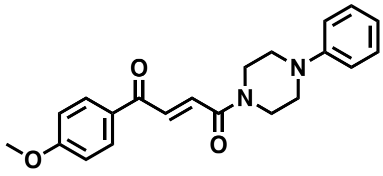

KN1021

General Procedure A was followed with *trans*-3-(4-methoxybenzoyl)acrylic acid (201.2 mg, 0.07 mmol), HATU (737.6 mg, 1.94 mmol), DIPEA (0.68 mL, 3.88 mmol), and 1-phenylpiperazine (25.0 mg, 0.06 mmol). The crude residue was purified by silica gel chromatography (15-55% EtOAc in Hexanes) to afford 255.6 mg (75%) of the title compound as an orange film.

^{1}H NMR (500 MHz, $CDCl_3$) δ 8.05 (d, *J* = 8.6 Hz, 2H), 7.99 (d, *J* = 14.9 Hz, 1H), 7.52 (d, *J* = 14.9 Hz, 1H), 7.30 (t, *J* = 7.7 Hz, 2H), 7.01 – 6.89 (m, 5H), 3.92 (m, 2H), 3.90 (s, 3H), 3.81 (t, *J* = 5.1 Hz, 2H), 3.23 (m, 4H).

^{13}C NMR (126 MHz, $CDCl_3$) δ 187.7, 164.2, 164.1, 150.8, 134.6, 131.4, 131.3, 130.0, 129.3, 120.8, 116.8, 114.1, 55.6, 50.0, 49.4, 46.0, 42.2.

LRMS (ESI) *m/z* calcd for $[C_{21}H_{22}N_2O_3 + H]^+$ = 351.2, found 351.1.

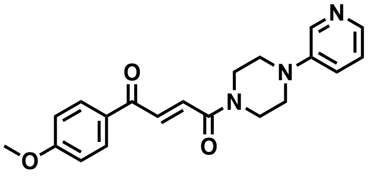

KN1018

General Procedure A was followed with *trans*-3-(4-methoxybenzoyl)acrylic acid (200.2 mg, 0.97 mmol), HATU (740.2 mg, 1.96 mmol), DIPEA (0.68 mL, 3.90 mmol), and 1-(3-pyridinyl)piperazine (158.3 mg, 0.97 mmol). The crude residue was purified by silica gel chromatography (0-8% MeOH in DCM) to afford 40.4 mg (12%) of the title compound as a yellow film.

^{1}H NMR (500 MHz, $CDCl_3$) δ 8.33 – 8.27 (m, 1H), 8.14 (dd, *J* = 3.8, 2.2 Hz, 1H), 8.06 – 8.00 (m, 2H), 7.97 (d, *J* = 14.9 Hz, 1H), 7.49 (d, *J* = 14.8 Hz, 1H), 7.22 – 7.14 (m, 2H), 6.99 – 6.92 (m, 2H), 3.90 (m, 2H), 3.86 (s, 3H), 3.80 (t, *J* = 5.2 Hz, 2H), 3.27 – 3.21 (m, 4H).

^{13}C NMR (126 MHz, $CDCl_3$) δ 187.6, 164.2, 164.1, 146.5, 141.7, 139.2, 134.8, 131.3, 131.1, 129.9, 123.6, 123.1, 114.1, 55.6, 49.2, 48.7, 45.7, 42.0.

LRMS (ESI) *m/z* calcd for $[C_{20}H_{21}N_3O_3 + H]^+$ = 352.2, found 352.1.

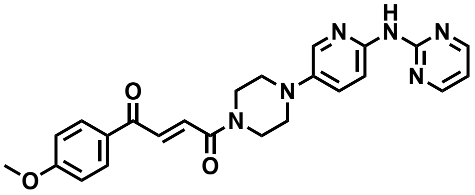

JP-2-199

General Procedure A was followed with *trans*-3-(4-methoxybenzoyl)acrylic acid (39.2 mg, 0.19 mmol), HATU (78.9 mg, 0.21 mmol), DIPEA (0.09 mL, 0.52 mmol), and **JP-2-198** (44.3 mg, 0.17 mmol). The crude residue was purified by silica gel chromatography (50-100% EtOAc in Hexanes followed by 0-5% MeOH in DCM) to afford 37.9 mg (49%) of the title compound as a yellow residue.

^{1}H NMR (700 MHz, $CDCl_3$) δ 8.77 (s, 1H), 8.50 (d, *J* = 4.8 Hz, 2H), 8.31 (d, *J* = 9.1 Hz, 1H), 8.10 (d, *J* = 2.9 Hz, 1H), 8.05 (d, *J* = 8.4 Hz, 2H), 7.99 (d, *J* = 14.8 Hz, 1H), 7.52 (d, *J* = 14.6 Hz, 1H), 7.34 (dd, *J* = 9.0, 2.9 Hz, 1H), 6.98 (d, *J* = 8.4 Hz, 2H), 6.76 (t, *J* = 4.9 Hz, 1H), 3.93 (t, *J* = 5.4 Hz, 2H), 3.89 (s, 3H), 3.83 (t, *J* = 5.1 Hz, 2H), 3.18 (q, *J* = 4.5 Hz, 4H).

^{13}C NMR (126 MHz, $CDCl_3$) δ 187.6, 164.2, 164.1, 159.3, 158.0, 147.2, 142.3, 137.5, 134.7, 131.3, 131.3, 130.0, 127.5, 114.1, 113.2, 112.9, 55.6, 50.7, 50.1, 45.9, 42.2, 29.7, 28.4.

LRMS (ESI) *m/z* calcd for $[C_{24}H_{24}N_6O_3 + H]^+$ = 445.2, found 445.2.

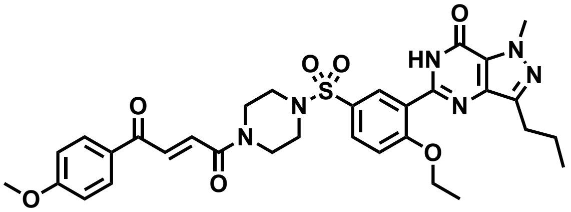

JP-2-201

The secondary amine **JP-2-196** (23.2 mg, 0.08 mmol) was dissolved in DCM (1 mL). DIPEA (0.1 mL, 0.5 mmol) was added, and the reaction mixture was stirred at ambient temperature for 5 minutes. A solution of 5-(5-Chlorosulfonyl-2-ethoxyphenyl)-1-methyl-3-propyl-1,6-dihydro-7H-pyrazolo[4,3-d]pyrimidin-7-one (34.7 mg, 0.08 mmol) in DCM (0.5 mL) was added dropwise to the reaction mixture and allowed to stir at ambient temperature for 2 hours. The volatiles were removed *in vacuo*, and the crude residue was purified by silica gel chromatography (25-100% EtOAc in Hexanes) to afford 49.5 mg (90% - *E:Z* = 90:10) of the title compound as a yellow oil.

^{1}H NMR (500 MHz, $CDCl_3$) δ 10.73 (s, 1H), 8.73 (d, *J* = 2.4 Hz, 1H), 7.92 – 7.89 (m, 2H), 7.82 (d, *J* = 14.9 Hz, 1H), 7.75 (dd, *J* = 8.7, 2.4 Hz, 1H), 7.27 (d, *J* = 14.8 Hz, 1H), 7.09 (d, *J* = 8.8 Hz, 1H), 6.89 – 6.84 (m, 2H), 4.30 (q, *J* = 7.0 Hz, 2H), 4.19 (s, 3H), 3.80 (s, 3H), 3.79 – 3.76 (m, 2H), 3.68 (t, *J* = 5.0 Hz, 2H), 3.05 (q, *J* = 5.4 Hz, 4H), 2.86 (t, *J* = 7.6 Hz, 2H), 1.79 (h, *J* = 7.4 Hz, 2H), 1.57 (t, *J* = 7.0 Hz, 3H), 0.96 (t, *J* = 7.4 Hz, 3H).

^{13}C NMR (126 MHz, $CDCl_3$) δ 187.3, 164.3, 164.0, 159.6, 153.6, 147.0, 146.2, 138.3, 135.2, 131.5, 131.3, 131.1, 130.5, 129.7, 128.5, 124.5, 121.4, 114.1, 113.3, 77.3, 66.2, 55.6, 46.4, 45.8, 45.4, 41.6, 38.2, 27.7, 22.3, 14.5, 14.1.

HRMS (ESI) *m/z* calcd for $[C_{32}H_{36}N_6O_7S + H]^+$ = 649.2366, found 649.2450.

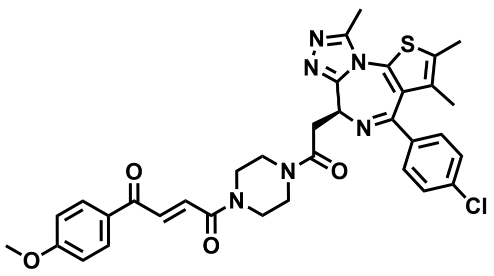

JP-2-197

General Procedure A was followed with (6S)-4-(4-chlorophenyl)-2,3,9-trimethyl-6H-thieno[3,2-f][1,2,4]triazolo[4,3-a][1,4]diazepine-6-acetic acid (JQ1-Acid) (92.5 mg, 0.23 mmol), HATU (93.0 mg, 0.24 mmol), DIPEA (0.1 mL, 0.6 mmol), and **JP-2-196** (55.0 mg, 0.2 mmol). The crude residue was purified by silica gel chromatography (0-5% MeOH in DCM) to afford 108.6 mg (83%, 2 steps - *E:Z* = 89:11) of the title compound as a yellow powder.

^{1}H NMR (700 MHz, $CDCl_3$) δ 8.08 (d, *J* = 8.6 Hz, 2H), 8.02 (dd, *J* = 14.9, 6.5 Hz, 1H), 7.51 (dd, *J* = 14.9, 6.7 Hz, 1H), 7.42 (d, *J* = 7.8 Hz, 2H), 7.39 – 7.34 (m, 2H), 7.01 (d, *J* = 8.5 Hz, 2H), 4.83 (td, *J* = 6.9, 2.9 Hz, 1H), 4.08 – 3.95 (m, 2H), 3.93 (s, 3H), 3.91 – 3.70 (m, 5H), 3.71 – 3.49 (m, 3H), 2.69 (s, 3H), 2.43 (s, 3H), 1.70 (s, 3H).

^{13}C NMR (151 MHz, $CDCl_3$) δ 187.8, 187.7, 169.3, 169.2, 164.5, 164.2, 164.2, 164.1, 155.6, 150.0, 136.7, 136.5, 134.9, 131.9, 131.3, 131.0, 131.0, 131.0, 130.9, 130.5, 129.7, 129.7, 128.6, 114.1, 114.0, 55.5, 54.2, 54.1, 45.8, 45.8, 45.6, 45.2, 42.1, 42.0, 41.8, 41.4, 35.1, 29.6, 14.2, 13.0, 11.6.

HRMS (ESI) *m/z* calcd for $[C_{34}H_{33}ClN_6O_4S + H]^+$ = 657.1973, found 657.2054.

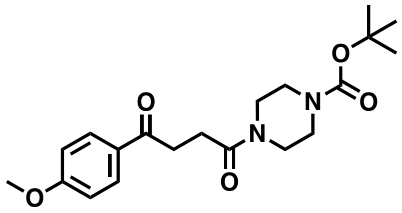

JP-2-229

General Procedure A was followed with 3-(4-methoxybenzoyl)propionic acid (100.7 mg, 0.48 mmol), HATU (206.8 mg, 0.54 mmol), DIPEA (0.25 mL, 1.45 mmol), and 1-boc-piperazine (104.7 mg, 0.56 mmol). The crude residue was purified by silica gel chromatography (0-100% EtOAc in Hexanes) to afford 132.8 mg (73%) of the title compound as a white powder.

^{1}**H NMR** (700 MHz, $CDCl_3$) δ 7.93 (d, *J* = 8.6 Hz, 2H), 6.87 (d, *J* = 8.5 Hz, 2H), 3.80 (s, 3H), 3.58 – 3.52 (m, 2H), 3.52 – 3.47 (m, 2H), 3.44 (d, *J* = 7.5 Hz, 2H), 3.35 (t, *J* = 5.5 Hz, 2H), 3.26 (t, *J* = 6.5 Hz, 2H), 2.71 (t, *J* = 6.6 Hz, 2H), 1.43 (s, 9H).

^{13}**C NMR** (151 MHz, $CDCl_3$) δ 197.5, 170.6, 163.5, 154.5, 130.3, 129.8, 113.7, 80.2, 55.4, 45.2, 41.6, 33.1, 29.7, 28.4, 27.1.

LRMS (ESI) *m/z* calcd for $[C_{20}H_{28}N_2O_5 + H]^+$ = 377.5, found 377.2.

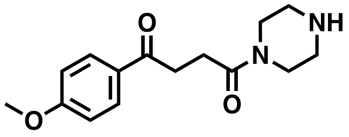

JP-2-231

General Procedure E was followed with **JP-2-229** (23.6 mg, 0.06 mmol), and TFA (0.13 mL, 1.7 mmol) for 2.5 hours. The crude material was used without further purification.

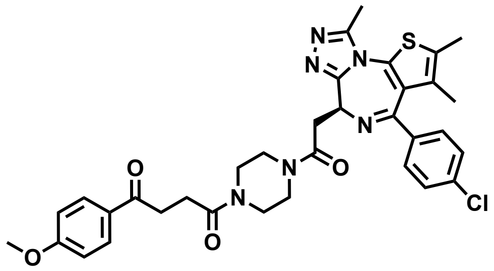

JP-2-232

General Procedure A was followed with (6S)-4-(4-chlorophenyl)-2,3,9-trimethyl-6H-thieno[3,2-f][1,2,4]triazolo[4,3-a][1,4]diazepine-6-acetic acid (JQ1-Acid) (26.1 mg, 0.07 mmol), HATU (27.3 mg, 0.07 mmol), DIPEA (0.4 mL, 0.19 mmol), and **JP-2-231** (17.3 mg, 0.06 mmol). The crude residue was purified by silica gel chromatography (0-7% MeOH in DCM) to afford 38.8 mg (94%, 2 steps) of the title compound as a yellow-white foam.

^{1}**H NMR** (700 MHz, $CDCl_3$) δ 7.99 (d, *J* = 8.8 Hz, 2H), 7.39 (d, *J* = 8.2 Hz, 2H), 7.32 (dd, *J* = 8.6, 4.2 Hz, 2H), 6.95 – 6.91 (m, 2H), 4.79 (q, *J* = 6.7 Hz, 1H), 4.01 – 3.94 (m, 1H), 3.86 (s, 3H), 3.85 – 3.80 (m, 2H), 3.79 – 3.69 (m, 4H), 3.69 – 3.63 (m, 1H), 3.60 – 3.55 (m, 1H), 3.54 – 3.48 (m, 1H), 3.42 – 3.35 (m, 1H), 3.32 – 3.26 (m, 1H), 2.84 (tt, *J* = 16.0, 6.5 Hz, 1H), 2.78 – 2.73 (m, 1H), 2.65 (d, *J* = 2.8 Hz, 3H), 2.39 (s, 3H), 1.67 (s, 3H).

^{13}C NMR (151 MHz, $CDCl_3$) δ 197.5, 170.7, 169.4, 169.2, 163.9, 163.8, 163.6, 155.8, 149.9, 149.9, 136.8, 136.7, 132.2, 130.9, 130.9, 130.7, 130.7, 130.5, 130.4, 129.9, 129.8, 128.7, 113.7, 55.5, 54.6, 54.4, 45.9, 45.6, 45.5, 45.2, 41.8, 41.7, 41.6, 35.4, 35.3, 33.2, 33.2, 31.9, 29.7, 27.2, 27.1, 22.7, 14.4, 14.1, 13.1, 11.8.

HRMS (ESI) *m/z* calcd for $[C_{34}H_{35}ClN_6O_4S + H]^+$ = 659.2129, found 659.2207.

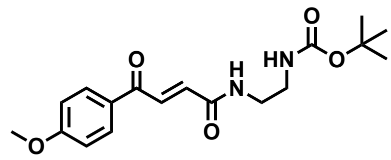

JP-2-216

General Procedure A was followed with *trans*-3-(4-methoxybenzoyl)acrylic acid (54.2 mg, 0.26 mmol), HATU (124.1 mg, 0.33 mmol), DIPEA (0.13 mL, 0.79 mmol), and *N*-boc-ethylenediamine (0.1 mL, 0.62 mmol). The crude residue was purified by silica gel chromatography (0-5% MeOH in DCM followed by 0-100% EtOAc in Hexanes) to afford 38.0 mg (42%) of the title compound as a brown residue, and 101.8 mg of a double addition byproduct. LRMS (ESI) of byproduct *m/z* calcd for $[C_{25}H_{40}N_4O_7 + H]^+$ = 509.3, found 509.3.

^{1}H NMR (500 MHz, $CDCl_3$) δ 8.1 – 8.0 (m, 2H), 7.9 (d, *J* = 14.96 Hz, 1H), 7.1 (s, 1H), 7.0 – 6.9 (m, 3H), 5.0 (d, *J* = 6.18 Hz, 1H), 3.9 (s, 3H), 3.5 (q, *J* = 5.48 Hz, 2H), 3.4 (q, *J* = 5.77 Hz, 2H), 1.4 (s, 9H).

^{13}C NMR (126 MHz, $CDCl_3$) δ 187.9, 164.9, 164.2, 157.1, 134.6, 133.2, 131.3, 130.0, 114.1, 80.0, 55.6, 41.4, 40.1, 28.4.

LRMS (ESI) *m/z* calcd for $[C_{18}H_{24}N_2O_5 + Na]^+$ = 371.2, found 371.1.

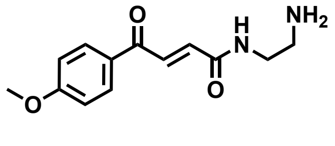

JP-2-218

General Procedure E was followed with **JP-2-216** (19.0 mg, 0.05 mmol), and TFA (0.13 mL, 1.8 mmol) for 40 minutes. The crude material was used without further purification.

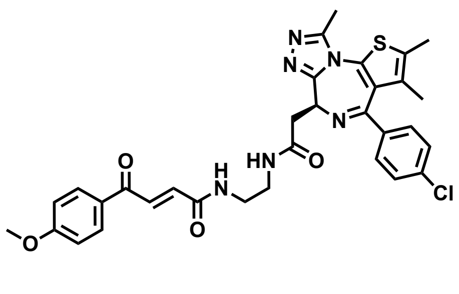

JP-2-219

General Procedure A was followed with (6S)-4-(4-chlorophenyl)-2,3,9-trimethyl-6H-thieno[3,2-f][1,2,4]triazolo[4,3-a][1,4]diazepine-6-acetic acid (JQ1-Acid) (24.6 mg, 0.06 mmol), HATU (27.0 mg, 0.07 mmol), DIPEA (0.04 mL, 0.23 mmol), and **JP-2-218** (13.5 mg, 0.05 mmol). The crude residue was purified by silica gel chromatography (0-6% MeOH in DCM) to afford 17.4 mg (51%, 2 steps - *E:Z* = 98:2) of the title compound as a yellow foam.

^{1}H NMR (600 MHz, $CDCl_3$) δ 8.02 – 7.98 (m, 2H), 7.95 (t, *J* = 5.6 Hz, 1H), 7.88 (s, 1H), 7.82 (q, *J* = 5.4 Hz, 1H), 7.39 – 7.36 (m, 2H), 7.29 (d, *J* = 7.4 Hz, 2H), 6.96 – 6.91 (m, 2H), 6.89 (d, *J* = 15.0 Hz, 1H), 4.71 (dd, *J* = 7.9, 6.2 Hz, 1H), 3.87 (s, 3H), 3.55 (qdt, *J* = 10.9, 6.8, 3.5 Hz, 5H), 3.46 (dd, *J* = 14.7, 6.2 Hz, 1H), 2.73 (s, 3H), 2.39 (s, 3H), 1.66 (s, 3H).

^{13}C NMR (151 MHz, $CDCl_3$) δ 188.0, 171.3, 164.8, 164.3, 164.0, 156.0, 150.4, 136.9, 136.4, 135.1, 132.6, 132.0, 131.2, 131.2, 131.0, 130.6, 130.2, 129.9, 128.7, 114.0, 55.5, 54.2, 40.3, 39.0, 38.9, 29.7, 14.4, 13.1, 11.8.

HRMS (ESI) *m/z* calcd for $[C_{32}H_{31}ClN_6O_4S + H]^+$ = 631.1816, found 631.1899.

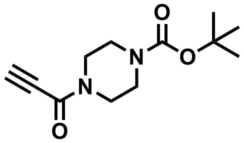

S1

To a solution of Boc-piperazine (7.2 g, 38.94 mmol) and DMAP (237 mg, 1.95 mmol) in DCM (45 mL) was added propiolic acid (3.0 g, 42.83 mmol) at ambient temperature. Next, DCC (5.6 g, 27.25 mmol) in DCM (5 mL) was added dropwise to the mixture at 0 °C, the mixture was stirred and warmed to ambient temperature over 1 hour. Once LCMS monitoring showed complete consumption of starting material, the reaction mixture was quenched with saturated $NaHCO_3$ aq. (20 mL) and extracted with EtOAc (20 mL x 3). The combined organic layers were washed by brine (50 mL), dried over Na_2SO_4, filtered and concentrated *in vacuo*. The crude residue was purified by silica gel chromatography (0-100% EtOAC in Petroleum Ether) to afford 7.2 g (78%) of the title compound as a yellow solid.

^{1}H NMR (400 MHz, $CDCl_3$) δ 3.75 (dd, *J* = 4.3, 6.2 Hz, 2H), 3.65 - 3.59 (m, 2H), 3.49 (dd, *J* = 4.3, 6.1 Hz, 2H), 3.45 - 3.40 (m, 2H), 3.16 (s, 1H), 1.48 (s, 9H).

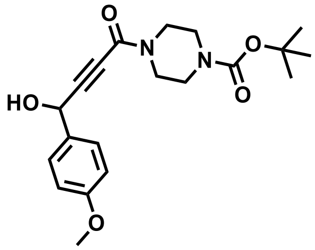

S2

To a solution of **S1** (2.0 g, 8.40 mmol) in THF (30 mL) was added dropwise n-BuLi (4.0 mL, 10.08 mmol) at -70 ^{o}C. The reaction was stirred at -70 ^{o}C for 30 mins. Then 4-methoxybenzaldehyde (914 mg, 6.72 mmol) and TMSCl (1.8 g, 16.80 mmol) was added dropwise to the mixture at -70 ^{o}C, the mixture was stirred and warmed to ambient temperature over 3 hours. The reaction mixture was quenched with ice water (200 mL), and extracted with EtOAc (200 mL x 2). The combined organic extracts were washed with brine (300 mL), dried over Na_2SO_4, filtered and concentrated *in vacuo*. The crude residue was purified by silica gel chromatography (0-100% EtOAc in Petroleum ether) to afford 1.3 g (41%) of the title compound as a white solid.

LCMS Rt = 0.454 min, M+23 (397.0), 71.7%.

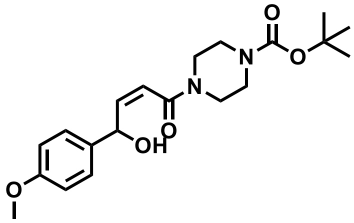

S3

A mixture of **S2** (300 mg, 0.80 mmol) and Lindlar's catalyst (30 mg) in THF (10 mL) was stirred at ambient temperature under a H_2 atmosphere (10 psi) for 2 hours. The reaction mixture was filtered and concentrated under reduced pressure to afford 300 mg of the title compound as a yellow solid, which was used without further purification.

^{1}H NMR (400 MHz, $CDCl_3$) δ 7.37 (d, J = 8.6 Hz, 2H), 6.90 - 6.86 (m, 2H), 6.28 (dd, J = 6.6, 11.9 Hz, 1H), 6.18 - 6.11 (m, 1H), 5.55 (br d, J = 5.1 Hz, 1H), 4.42 (br s, 1H), 3.81 - 3.79 (m, 3H), 3.68 - 3.61 (m, 2H), 3.49 - 3.41 (m, 6H), 1.48 (s, 9H).

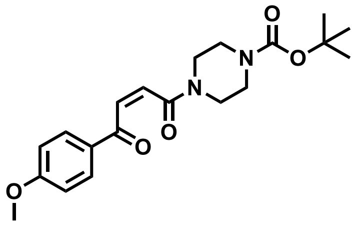

LE-21-PX17

To a mixture of **S3** (150 mg, 0.40 mmol) in DCM (5 mL) was added DMP (186 mg, 0.44 mmol) at 0 ºC. The mixture was stirred and warmed to ambient temperature over 2 hours. The reaction mixture was filtered and concentrated *in vacuo* The crude residue was purified by silica gel chromatography (0-100% EtOAc in Petroleum Ether) to afford 53 mg (35%- *E:Z* = >99:<1) of the title compound as a white solid.

^{1}H NMR (400 MHz,$CDCl_3$) δ 7.98 - 7.93 (m, 2H), 7.03 (d, J = 11.9 Hz, 1H), 6.98 - 6.92 (m, 2H), 6.56 (d, J = 12.0 Hz, 1H), 3.89 (s, 3H), 3.65 (br s, 2H), 3.52 - 3.44 (m, 6H), 1.49 - 1.47 (m, 9H).

^{13}C NMR (126 MHz, $CDCl_3$) δ 188.6, 166.7, 164.0, 154.5, 134.4, 131.1, 129.7, 128.7, 114.0, 80.2, 55.5, 46.1, 41.1, 28.4.

HRMS (ESI) *m/z* calcd for $[C_{20}H_{26}N_2O_5 + Na]^+$ = 379.1820, found 379.1735.

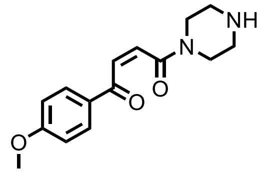

S4

General Procedure E was followed with **LE-21-PX17** (100.0 mg, 0.26 mmol), and TFA (0.4 mL) for 1 hour. The crude material was used without further purification.

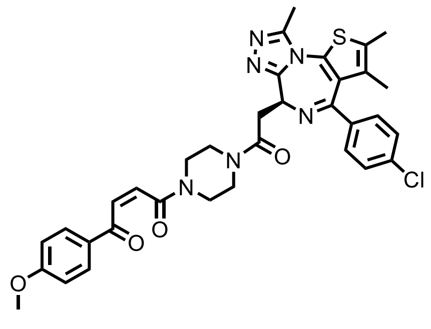

FB-84-GG64

General Procedure A was followed with (6S)-4-(4-chlorophenyl)-2,3,9-trimethyl-6H-thieno[3,2-f][1,2,4]triazolo[4,3-a][1,4]diazepine-6-acetic acid (JQ1-Acid) (144.0 mg, 0.36 mmol), HATU (152.1 mg, 0.40 mmol), DIPEA (0.16 mL, 0.94 mmol), and **S4** (100.0 mg, 0.36 mmol). The crude residue was purified by Prep-HPLC (NH_4HCO_3) to afford 31.2 mg (13%, 2 steps - *E:Z* = >99:<1) of the title compound as a white solid.

^{1}H NMR (500 MHz, $CDCl_3$) δ 7.89 (d, *J* = 8.5 Hz, 2H), 7.36 – 7.30 (m, 2H), 7.28 (d, *J* = 8.2 Hz, 2H), 6.99 (s, 1H), 6.93 – 6.85 (m, 2H), 6.52 (d, *J* = 11.8 Hz, 1H), 4.76 – 4.70 (m, 1H), 3.81 (s, 5H), 3.73 (s, 2H), 3.65 – 3.39 (m, 6H), 2.60 (s, 3H), 2.35 – 2.31 (m, 3H), 1.61 (d, *J* = 0.9 Hz, 3H).

^{13}C NMR (126 MHz, $CDCl_3$) δ 188.6, 169.3, 166.8, 164.0, 155.8, 149.9, 136.8, 136.7, 134.6, 134.5, 132.2, 131.1, 130.9, 130.7, 130.5, 129.8, 129.7, 128.7, 114.1, 55.6, 54.6, 54.4, 46.4, 45.9, 45.3, 41.7, 41.4, 41.2, 35.3, 14.4, 13.1, 11.9.

HRMS (ESI) *m/z* calcd for $[C_{34}H_{33}ClN_6O_4S + Na]^+$ = 679.2020, found 679.1867.

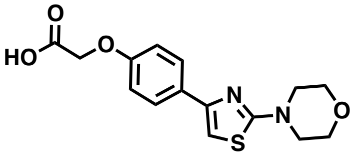

JP-2-215

A solution of *tert*-butyl 2-(4-(2-morpholinothiazol-4-yl)phenoxy)acetate (40.0 mg, 0.11 mmol) in 4 M hydrochloric acid in dioxane (0.7 mL) was stirred at ambient temperature overnight. The reaction mixture was diluted with toluene and the volatiles were removed *in vacuo* to yield a yellow-white solid which was used without further purification.

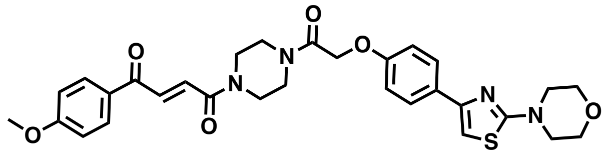

JP-2-217

General Procedure A was followed with 2-(4-(2-morpholinothiazol-4-yl)phenoxy)acetic acid (35.00 mg, 0.11 mmol), HATU (49.9 mg, 0.13 mmol), DIPEA (0.06 mL, 0.33 mmol), and **JP-2-196** (36.9 mg, 0.13 mmol). The crude residue was purified by silica gel chromatography (0-5% MeOH in DCM) to afford 34.6 mg (55%, 2 steps - *E:Z* = 93:7) of the title compound as a beige powder.

^{1}H NMR (700 MHz, $CDCl_3$) δ 8.03 (d, *J* = 8.6 Hz, 2H), 7.96 (dd, *J* = 14.9, 4.6 Hz, 1H), 7.79 – 7.76 (m, 2H), 7.43 (d, *J* = 14.7 Hz, 1H), 6.99 – 6.94 (m, 4H), 6.68 (s, 1H), 4.76 (d, *J* = 3.3 Hz, 2H), 3.89 (s, 3H), 3.85 – 3.82 (m, 4H), 3.73 (q, *J* = 6.3 Hz, 1H), 3.68 (p, *J* = 5.5 Hz, 5H), 3.64 – 3.59 (m, 2H), 3.54 – 3.50 (m, 4H).

^{13}C NMR (151 MHz, $CDCl_3$) δ 187.5, 171.2, 166.9, 164.4, 164.3, 157.1, 151.2, 135.1, 131.3, 130.8, 129.9, 129.3, 127.6, 114.5, 114.1, 100.5, 68.2, 68.0, 66.2, 55.6, 48.6, 46.1, 45.7, 45.2, 42.4, 42.0.

HRMS (ESI) *m/z* calcd for $[C_{30}H_{32}N_4O_6S + H]^+$ = 577.2043, found 577.2122.

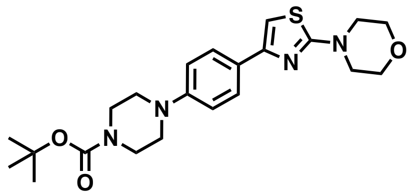

JP-2-221

A mixture of 4-(4-bromo-2-thiazolyl)morpholine (120.0 mg, 0.51 mmol), 4-(4-*tert*-butoxycarbonylpiperazinyl)phenylboronic acid pinacol ester (210.6, 0.54 mmol), caesium carbonate (485.9 mg, 1.5 mmol), and tetrakis(triphenylphosphine)palladium(0) (66.4 mg, 0.05 mmol) was suspended in DMF (12 mL) and stirred at 100 °C overnight. The reaction was quenched with 5% LiCl $_{(aq)}$ (60 mL) and extracted 3 times with DCM. The organic extracts were washed again with 5% LiCl $_{(aq)}$ and the organic extract was dried over Na_2SO_4, vacuum filtered, and the volatiles were removed *in vacuo*. The crude residue was purified by silica gel chromatography (0-50% EtOAc in Hexanes) to afford 28.8 mg (32%, BRSM) of the title compound as a white solid.

^{1}H NMR (700 MHz, $CDCl_3$) δ 7.75 – 7.72 (m, 2H), 6.93 – 6.90 (m, 2H), 6.65 (s, 1H), 3.85 – 3.81 (m, 4H), 3.58 (t, *J* = 5.2 Hz, 4H), 3.52 (dd, *J* = 5.9, 3.9 Hz, 4H), 3.16 (t, *J* = 5.1 Hz, 4H), 1.49 (s, 9H).

^{13}C NMR (126 MHz, $CDCl_3$) δ 171.1, 151.8, 150.7, 127.3, 127.2, 127.0, 116.8, 116.3, 99.7, 79.9, 66.3, 49.2, 48.6, 28.5.

LRMS (ESI) *m/z* calcd for $[C_{22}H_{30}N_4O_3S + H]^+$ = 431.6, found 431.2.

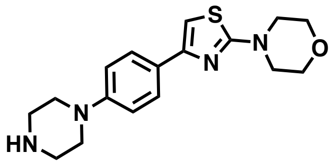

JP-2-223

General Procedure E was followed with **JP-2-221** (33.6 mg, 0.07 mmol), and TFA (0.18 mL, 2.4 mmol) for 30 minutes. The crude material was used without further purification.

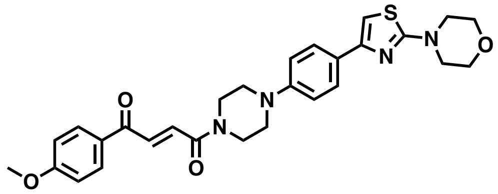

JP-2-224

General Procedure A was followed with *trans*-3-(4-methoxybenzoyl)acrylic acid (19.3 mg, 0.09 mmol), HATU (43.8 mg, 0.10 mmol), DIPEA (0.04 mL, 0.23 mmol), and **JP-2-223** (23.0 mg, 0.07 mmol). The crude residue was purified by silica gel chromatography (0-100% EtOAc in Hexanes) to afford 18.6 mg (51%, 2 steps - *E:Z* = 47:53) of the title compound as a light orange foam.

^{1}H NMR (700 MHz, $CDCl_3$) δ 8.07 – 8.03 (m, 2H), 7.99 (d, *J* = 14.8 Hz, 1H), 7.77 – 7.73 (m, 2H), 7.54 – 7.50 (m, 1H), 7.00 – 6.96 (m, 2H), 6.95 – 6.91 (m, 2H), 6.66 (s, 1H), 3.91 (t, *J* = 5.2 Hz, 2H), 3.89 (s, 3H), 3.83 (t, *J* = 4.9 Hz, 4H), 3.81 (d, *J* = 5.1 Hz, 2H), 3.52 (t, *J* = 4.9 Hz, 4H), 3.26 (q, *J* = 4.9 Hz, 4H).

^{13}C NMR (126 MHz, $CDCl_3$) δ 187.7, 171.2, 164.2, 164.1, 151.6, 150.1, 134.6, 131.3, 130.0, 127.7, 127.1, 116.4, 114.1, 99.9, 66.3, 55.6, 49.7, 49.1, 48.6, 45.9, 42.1.

HRMS (ESI) *m/z* calcd for $[C_{28}H_{30}N_4O_4S + H]^+$ = 519.1988, found 519.2067.

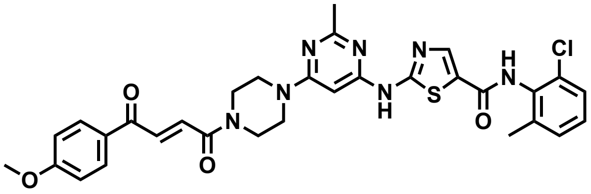

JP-2-227

General Procedure A was followed with *trans*-3-(4-methoxybenzoyl)acrylic acid (7.0 mg, 0.03 mmol), HATU (12.8 mg, 0.03 mmol), DIPEA (0.02 mL, 0.13 mmol), and *N*-deshydroxyethyl dasatinib (10.0 mg, 0.02 mmol). The crude residue was purified by silica gel chromatography (0-6% MeOH in DCM) to afford 12.8 mg (90% - *E:Z* = 93:7) of the title compound as a yellow solid.

^{1}H NMR (700 MHz, $CDCl_3$) δ 8.00 – 7.95 (m, 3H), 7.92 (d, *J* = 14.9 Hz, 1H), 7.42 (d, *J* = 14.9 Hz, 1H), 7.24 (d, *J* = 7.7 Hz, 1H), 7.15 – 7.07 (m, 2H), 6.95 – 6.91 (m, 2H), 5.87 (s, 1H), 3.83 (s, 3H), 3.77 (t, *J* = 5.3 Hz, 2H), 3.70 (p, *J* = 5.2 Hz, 4H), 3.62 (d, *J* = 5.5 Hz, 2H), 2.45 (s, 3H), 2.26 (s, 3H).

^{13}C NMR (126 MHz, $CDCl_3$) δ 188.0, 166.5, 164.6, 164.4, 162.8, 156.9, 140.4, 138.6, 135.0, 132.4, 131.4, 131.0, 129.7, 129.1, 128.1, 127.1, 125.6, 114.2, 83.1, 55.5, 45.5, 43.9, 43.6, 41.8, 29.6, 25.4, 18.7.

HRMS (ESI) *m/z* calcd for $[C_{31}H_{30}ClN_7O_4S + H]^+$ = 632.1769, found 632.1848.

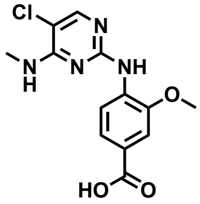

JP-2-241

A mixture of 2,5-dichloro-N-methyl pyrimidin-4-amine (106.2 mg, 0.60 mmol) and 4-amino-3-methoxybenzoic acid (111.8 mg, 0.67 mmol) was dissolved in a 1:1 mixture of Dioxane:H_2O (4 mL). A solution of 4 M HCl in dioxane (0.15 mL) was added and the reaction mixture was stirred at 100 °C overnight. A white precipitate formed upon cooling which was filtered and washed with H_2O to afford 78.7 mg (43%) of the title compound as a white powder which product was used without further purification.

^{1}H NMR (500 MHz, DMSO) δ 9.17 (s, 1H), 8.55 (d, *J* = 5.8 Hz, 1H), 8.29 (d, *J* = 8.5 Hz, 1H), 8.18 (s, 1H), 7.63 (d, *J* = 8.5 Hz, 1H), 7.55 (s, 1H), 3.93 (s, 3H), 2.98 (d, *J* = 4.6 Hz, 3H).

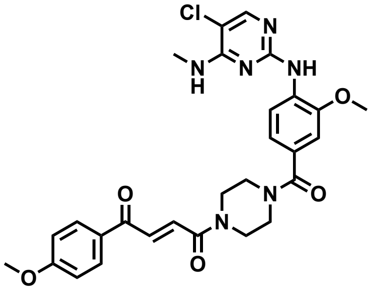

JP-2-244

General Procedure A was followed with **JP-2-241** (21.3 mg, 0.07 mmol), HATU (34.2 mg, 0.07 mmol), DIPEA (0.05 mL, 0.3 mmol), and **JP-2-196** (20.8 mg, 0.07 mmol). The crude residue was purified by silica gel chromatography (0-8% MeOH in DCM) to afford 23.3 mg (60%, 2 steps - *E:Z* = 91:9) of the title compound as a yellow film.

^{1}H NMR (700 MHz, $CDCl_3$) δ 8.58 (d, *J* = 8.2 Hz, 1H), 8.06 – 8.02 (m, 2H), 7.99 (d, *J* = 14.9 Hz, 1H), 7.93 (s, 1H), 7.67 (s, 1H), 7.47 (d, *J* = 14.9 Hz, 1H), 7.04 (d, *J* = 1.8 Hz, 1H), 7.02 (dd, *J* = 8.3, 1.8 Hz, 1H), 7.00 – 6.96 (m, 2H), 5.34 (q, *J* = 4.9 Hz, 1H), 3.93 (s, 3H), 3.89 (s, 3H), 3.81 – 3.59 (m, 8H), 3.11 (d, *J* = 4.9 Hz, 3H).

^{13}C NMR (126 MHz, $CDCl_3$) δ 187.5, 170.9, 164.3, 164.3, 158.6, 147.6, 135.1, 131.7, 131.4, 130.9, 129.9, 120.3, 116.9, 114.2, 109.7, 105.7, 56.0, 55.6, 46.1, 42.5, 28.2.

HRMS (ESI) *m/z* calcd for $[C_{28}H_{29}ClN_6O_5 + H]^+$ = 565.1888, found 565.1968.

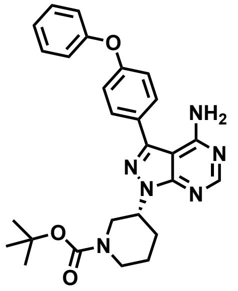

JP-2-242

A mixture of (4-phenoxyphenyl)boronic acid (60.6 mg, 0.28 mmol), *tert*-butyl (*R*)-3-(4-amino-3-iodo-1*H*-pyrazolo[3,4-*d*]pyrimidin-1-yl)piperidine-1-carboxylate (117.1 mg, 0.26 mmol), tetrakis(triphenylphosphine)palladium (28.4 mg, 0.03 mmol), and potassium carbonate (105.0 mg, 0.79 mmol) was suspended in a 1:1 mixture of Dioxane:H_2O (4 mL) and the reaction mixture was stirred at 90 °C for 5 hours. The solvent was removed *in vacuo*, and the crude residue was purified by silica gel chromatography (0-70% EtOAc in Hexanes) to afford 127.1 mg (99%) of the title compound as a yellow foam.

^{1}H NMR (500 MHz, $CDCl_3$) δ 8.37 (s, 1H), 7.65 (dd, *J* = 8.1, 6.1 Hz, 2H), 7.41 – 7.36 (m, 2H), 7.15 (dd, *J* = 8.4, 6.4 Hz, 3H), 7.08 (d, *J* = 7.9 Hz, 2H), 5.64 (s, 2H), 4.84 (tt, *J* = 10.9, 4.4 Hz, 1H), 4.47 – 4.03 (m, 2H), 2.85 (td, *J* = 13.0, 3.0 Hz, 1H), 2.32 – 2.14 (m, 2H), 1.88 (d, *J* = 7.9 Hz, 1H), 1.75 – 1.63 (m, 1H), 1.44 (s, 9H).

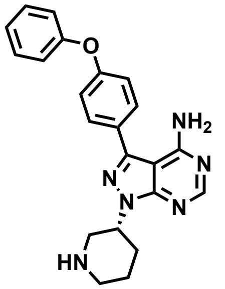

JP-2-246

General Procedure E was followed with **JP-2-242** (37.2 mg, 0.08 mmol), and TFA (0.19 mL, 2.5 mmol) for 30 minutes. The crude material was used without further purification.

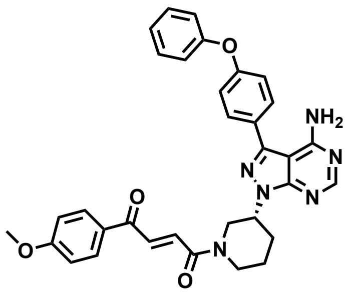

JP-2-247

General Procedure A was followed with *trans*-3-(4-methoxybenzoyl)acrylic acid (24.5 mg, 0.09 mmol), HATU (40.7 mg, 0.11 mmol), DIPEA (0.05 mL, 0.29 mmol), and **JP-2-246** (27.8 mg, 0.07 mmol). The crude residue was purified by silica gel chromatography (0-5% MeOH in DCM) and then by reverse phase silica gel chromatography (5-95% MeCN in H_2O) to afford 16.9 mg (41%, 2 steps - *E:Z* = 85:15) of the title compound as a white solid.

^{1}H NMR (700 MHz, $CDCl_3$) δ 8.38 – 8.31 (m, 1H), 8.07 – 8.03 (m, 1H), 8.00 – 7.97 (m, 1H), 7.97 – 7.85 (m, 1H), 7.67 – 7.62 (m, 2H), 7.55 – 7.41 (m, 1H), 7.40 – 7.36 (m, 2H), 7.17 (t, *J* = 7.3 Hz, 1H), 7.16 – 7.13 (m, 2H), 7.08 (d, *J* = 8.0 Hz, 2H), 7.00 – 6.96 (m, 1H), 6.96 – 6.92 (m, 1H), 5.64 (s, 2H), 4.96 – 4.87 (m, 2H), 4.55 – 4.49 (m, 1H), 4.22 (dd, *J* = 13.6, 4.1 Hz, 1H), 4.11 (d, *J* = 13.8 Hz, 1H), 3.92 (dd, *J* = 13.4, 9.9 Hz, 1H), 3.88 (d, *J* = 13.3 Hz, 3H), 3.48 (dd, *J* = 12.6, 10.6 Hz, 1H), 3.28 (td, *J* = 13.2, 2.9 Hz, 1H), 3.08 (ddd, *J* = 13.7, 11.1, 3.2 Hz, 1H), 2.45 – 2.33 (m, 1H), 2.28 (dd, *J* = 13.7, 4.1 Hz, 1H), 2.05 (ddt, *J* = 12.1, 7.5, 3.7 Hz, 1H), 1.81 – 1.71 (m, 1H).

^{13}C NMR (151 MHz, $CDCl_3$) δ 187.7, 187.6, 164.7, 164.4, 164.2, 164.1, 158.6, 158.6, 157.8, 156.4, 156.3, 155.8, 155.7, 154.3, 154.3, 144.1, 144.0, 134.4, 134.1, 132.0, 131.9, 131.3, 131.3, 130.0, 127.8, 127.6, 124.1, 119.6, 119.1, 114.1, 114.0, 98.6, 98.6, 55.6, 55.6, 53.4, 52.4, 50.1, 46.3, 46.2, 42.5, 30.3, 30.0, 25.3, 23.7.

HRMS (ESI) *m/z* calcd for $[C_{33}H_{30}N_6O_4 + H]^+$ = 575.2329, found 575.2406.

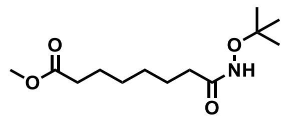

DD-1-42-P1

General Procedure D was followed with suberic acid monomethyl ester (500 mg, 2.66 mmol), T3P (1.66 mL, 2.79 mmol), DIPEA (1.38 mL, 7.98 mmol), and O-*tert*-Butylhydroxylamine hydrochloride (400 mg, 3.19 mmol). The reaction was quenched with 5% LiCl (aq) (60 mL) and extracted 3 times with EtOAc. The organic extracts were washed again with 5% LiCl (aq) and the organic extract was dried over Na_2SO_4, vacuum filtered, and the volatiles were removed *in vacuo*. The crude residue was purified by silica gel chromatography (0-100% EtOAc in Hexanes) to afford 573 mg (83%) of the title compound as a slightly yellow, thick oil.

^{1}H NMR (500 MHz, $CDCl_3$) δ 3.65 (s, 3H), 2.29 (t, J = 7.5 Hz, 2H), 2.14 – 2.01 (m, 1H), 1.62 (qd, J = 12.9, 8.0 Hz, 4H), 1.33 (p, J = 3.7 Hz, 4H), 1.25 (s, 9H).

^{13}C NMR (151 MHz, $CDCl_3$) δ 174.4, 172.2, 81.9, 51.6, 34.1, 33.4, 28.9, 26.4, 25.4, 24.8, 14.3.

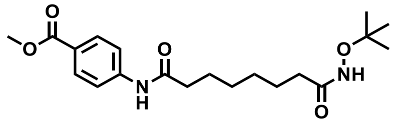

DD-1-47-P1

Methyl ester **DD-1-42-P1** (100 mg, 0.39 mmol) was dissolved in MeOH (2.0 mL) and 2M LiOH (aq, 0.77 mL) was added dropwise. The reaction mixture was stirred at ambient temperature overnight, and upon completion was quenched with 1M HCl (10 mL) and extracted 3 times with DCM. The organic extracts were washed again with brine and the organic extract was dried over Na_2SO_4, vacuum filtered, and the volatiles were removed *in vacuo*. The crude material was used without further purification.

A modified **General Procedure A** was followed with crude residue, HATU (147 mg, 0.39 mmol), DIPEA (0.2 mL, 1.16 mmol), and methyl 4-aminobenzoate (64 mg, 0.42 mmol). The reaction mixture was stirred at 100 °C for two days. The reaction was quenched with 5% LiCl (aq) (20 mL) and extracted 3 times with EtOAc. The organic extracts were washed again with 5% LiCl (aq) and the organic extract was dried over Na_2SO_4, vacuum filtered, and the volatiles were removed *in vacuo*. The crude residue was purified by silica gel chromatography (0-100% EtOAc in Hexanes) to afford 55 mg (38%) of the title compound as a beige solid.

^{1}H NMR (500 MHz, DMSO) δ 10.20 (s, 2H), 7.89 (d, J = 3.0 Hz, 2H), 7.72 (d, J = 3.0 Hz, 2H), 3.81 (s, 3H), 2.34 (t, J = 8.3 Hz, 2H), 1.99 (t, J = 7.9 Hz, 2H), 1.30 – 1.24 (m, 5H), 1.13 (s, 9H).

^{13}C NMR (126 MHz, DMSO) δ 171.9, 170.4, 165.8, 143.7, 130.2, 123.6, 118.3, 80.1, 51.8, 36.4, 32.3, 28.3, 26.4, 25.0, 24.9, 20.6.

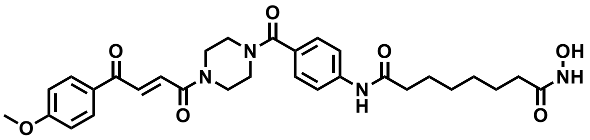

DD-1-073-P1

Methyl ester **DD-1-47-P1** (55 mg, 0.15 mmol) was dissolved in MeOH (6.0 mL) and 2M LiOH (aq, 0.90 mL) was added dropwise. The reaction mixture was stirred at ambient temperature for 7 days. The volatiles were removed *in vacuo*, and the off-white residue was dissolved in water and acidified with 1M HCl until a beige precipitate was formed that was vacuum filtered and dried under vacuum (40 mg) and the resultant material was used without further purification.

A modified **General Procedure A** was followed with crude residue, HATU (42 mg, 0.11 mmol), DIPEA (0.08 mL, 1.16 mmol), and **JP-2-196** (33 mg, 0.12 mmol) and stirred overnight. The reaction was quenched with 5% LiCl (aq) (20 mL) and extracted 3 times with EtOAc. The organic extracts were washed again with 5% LiCl (aq) and the organic extract was dried over Na_2SO_4, vacuum filtered, and the volatiles were removed *in vacuo*. The crude residue was purified by silica gel chromatography (0-10% MeOH in DCM) to afford 62 mg (66%) of the *tert*-butyl protected hydroxamic acid, which was dissolved in TFA (5.0 mL) and stirred at 35 °C overnight. The volatiles were removed *in vacuo* and the brown residue was dissolved in DCM, adsorbed onto silica gel and was purified by silica gel chromatography (0-10% MeOH in DCM, 0.1% AcOH) to afford 27mg (49%, 2 steps - *E:Z* = 97:7) of the title compound as a beige film.

^{1}H NMR (500 MHz, MeOD) δ 8.07 (dd, J = 8.9, 3.0 Hz, 2H), 7.92 (d, J = 15.1 Hz, 1H), 7.71 (d, J = 8.1 Hz, 2H), 7.52 (d, J = 15.2 Hz, 1H), 7.45 (d, J = 8.1 Hz, 2H), 7.11 – 7.06 (m, 2H), 3.91 (s, 3H), 3.85 – 3.45 (m, 8H), 2.41 (t, J = 7.4 Hz, 2H), 2.11 (t, J = 7.4 Hz, 2H), 1.69 (dp, J = 36.6, 7.0 Hz, 4H), 1.48 – 1.35 (m, 4H).

^{13}C NMR (126 MHz, MeOD) δ 189.4, 174.8, 172.6, 166.7, 166.0, 142.1, 135.9, 132.9, 132.4, 132.3, 131.3, 131.0, 129.3, 120.7, 115.3, 56.2, 47.1, 43.2, 37.9, 33.7, 29.9, 29.8, 26.6, 26.6.

HRMS (ESI) *m/z* calcd for $[C_{30}H_{36}N_4O_7 + Na]^+$ = 587.2476, found 587.2482.

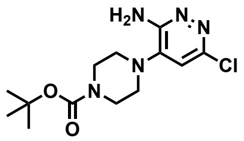

JP-2-238

A mixture of 3-amino-4-bromo-6-chloropyridazine (108.5 mg, 0.52 mmol) and 1-boc-piperazine (461.9 mg, 2.5 mmol) was dissolved in a THF (1 mL) and the reaction mixture was stirred at 80 °C for overnight. The solvent was removed *in vacuo*, and the crude residue was purified by silica gel chromatography (0-100% EtOAc in Hexanes) to afford 147.4 mg (90%) of the title compound as a yellow foam.

^{1}H NMR (500 MHz, $CDCl_3$) δ 6.73 (s, 1H), 5.12 (s, 2H), 3.60 – 3.55 (m, 4H), 3.00 (t, *J* = 5.0 Hz, 4H), 1.48 (s, 9H).

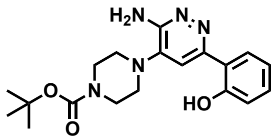

JP-2-243

A mixture of **JP-2-238** (122.3 mg, 0.39 mmol), 2-hydroxyphenyl boronic acid (138.4 mg, 1.00 mmol), [1,1′-Bis(diphenylphosphino)ferrocene]dichloropalladium(II) (34.5 mg, 0.05 mmol), and potassium carbonate (186.1 mg, 1.35 mmol) was suspended in a 1:1 mixture of MeCN:H_2O (2.5 mL) in a sealed tube, and the reaction mixture was stirred at 120 °C for 45 minutes. The solvent was removed *in vacuo*, and the crude residue was purified by silica gel chromatography (0-100% EtOAc in Hexanes) to afford 21.2 mg (15%) of the title compound as a white-yellow powder.

^{1}H NMR (500 MHz, $CDCl_3$) δ 7.57 (dd, *J* = 8.0, 1.6 Hz, 1H), 7.29 (ddd, *J* = 8.5, 7.2, 1.6 Hz, 1H), 7.05 (dd, *J* = 8.2, 1.2 Hz, 1H), 6.91 (td, *J* = 7.6, 1.3 Hz, 1H), 4.88 (s, 2H), 3.65 (t, *J* = 5.0 Hz, 4H), 3.10 (t, *J* = 5.0 Hz, 4H), 1.50 (s, 9H).

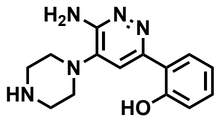

JP-2-248

General Procedure E was followed with **JP-2-243** (11.2 mg, 0.03 mmol), and TFA (0.09 mL, 0.96 mmol) for 1 hour. The solvent was removed *in vacuo*, and the crude residue was purified by silica gel chromatography (0-20% MeOH in DCM with 0.1% Et_3N) to afford 6.2 mg (76%) of the title compound as a yellow oil.

^{1}H NMR (600 MHz, DMSO) δ 7.92 (dd, *J* = 8.3, 1.6 Hz, 1H), 7.48 (s, 1H), 7.24 (ddd, *J* = 8.4, 7.3, 1.6 Hz, 1H), 6.89 (dtd, *J* = 8.2, 3.6, 1.2 Hz, 2H), 6.23 (d, *J* = 6.0 Hz, 1H), 4.01 (s, 1H), 3.04 (t, *J* = 5.0 Hz, 4H), 2.98 – 2.91 (m, 4H).

LRMS (ESI) *m/z* calcd for $[C_{14}H_{17}N_5O + H]^+$ = 272.3, found 272.1.

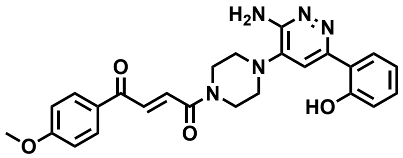

JP-2-249

General Procedure A was followed with *trans*-3-(4-methoxybenzoyl)acrylic acid (12.5 mg, 0.06 mmol), HATU (27.7 mg, 0.07 mmol), DIPEA (0.05 mL, 0.29 mmol), and **JP-2-248** (16.4 mg, 0.06 mmol). The crude residue was purified by silica gel chromatography (0-10% MeOH in DCM) to afford 25.5 mg (92%, 2 steps - *E:Z* = 92:8) of the title compound as a white-yellow powder.

^{1}H NMR (700 MHz, $CDCl_3$) δ 8.02 (d, *J* = 8.5 Hz, 2H), 7.97 (d, *J* = 14.9 Hz, 1H), 7.54 (d, *J* = 8.0 Hz, 1H), 7.48 (d, *J* = 14.9 Hz, 1H), 7.30 (s, 1H), 7.26 (t, *J* = 1.7 Hz, 1H), 7.00 (d, *J* = 8.1 Hz, 1H), 6.96 (d, *J* = 8.6 Hz, 2H), 6.89 (t, *J* = 7.5 Hz, 1H), 3.92 (t, *J* = 5.0 Hz, 2H), 3.87 (s, 3H), 3.86 – 3.83 (m, 2H), 3.16 (t, *J* = 5.2 Hz, 4H), 2.47 (br s, 2H).

^{13}C NMR (151 MHz, $CDCl_3$) δ 187.8, 164.6, 164.5, 158.7, 155.0, 154.1, 140.6, 135.3, 131.5, 131.1, 130.9, 129.8, 125.3, 119.1, 118.4, 117.5, 114.3, 111.6, 55.7, 49.6, 49.1, 49.1, 45.9, 42.1.

HRMS (ESI) *m/z* calcd for $[C_{25}H_{25}N_5O_4 + H]^+$ = 460.1979, found 460.1983.

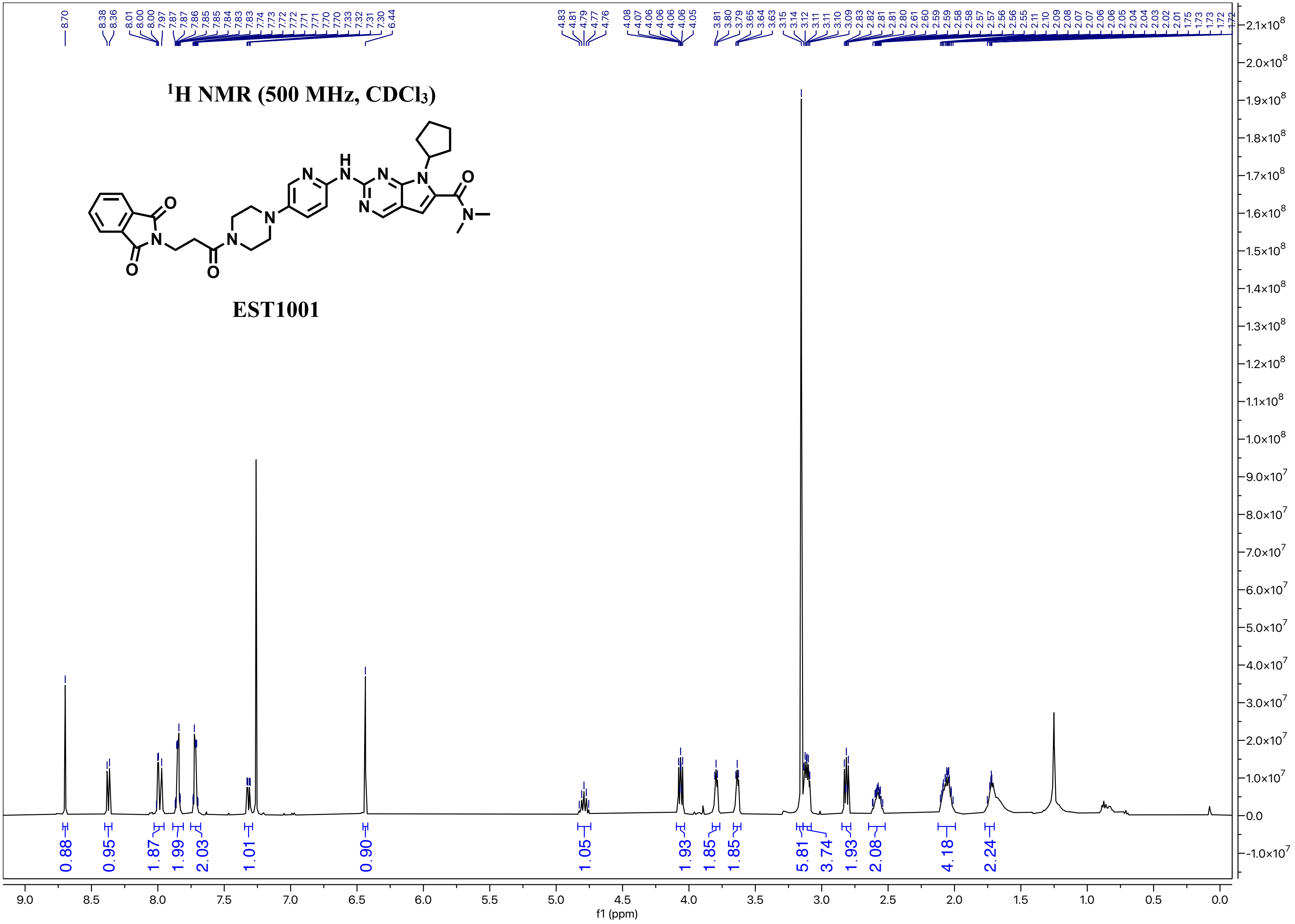

^{1}H NMR (500 MHz, $CDCl_3$)
EST1001
f1 (ppm)
0.88
0.95
1.87
1.99
2.03
1.01
0.90
1.05
1.93
1.85
1.85
5.81
3.74
1.93
2.08
4.18
2.24

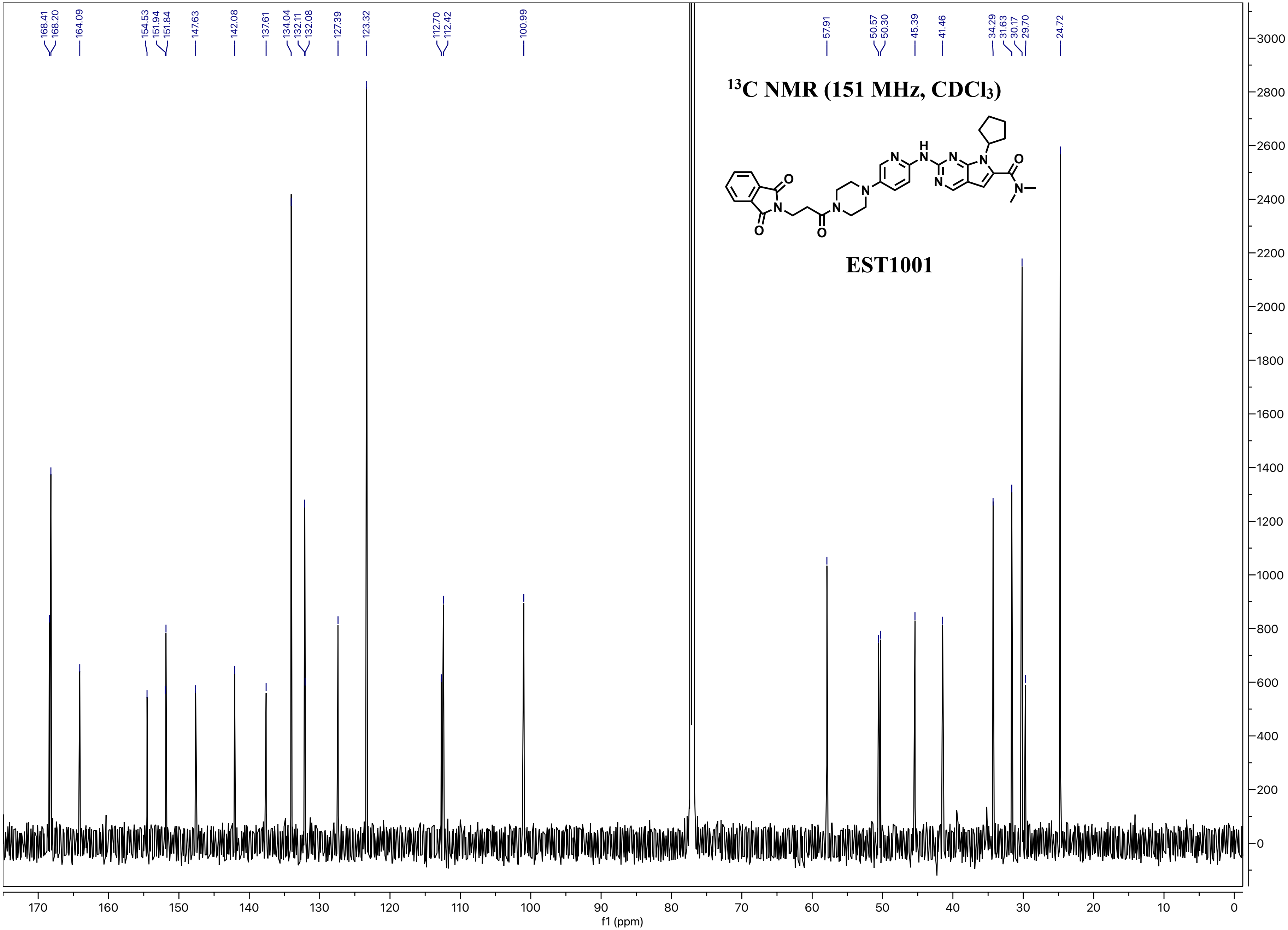

168.41
168.20
164.09
154.53
151.94
151.84
147.63
142.08
137.61
134.04
132.11
132.08
127.39
123.32
112.70
112.42
100.99
57.91
50.57
50.30
45.39
41.46
34.29
31.63
30.17
29.70
24.72
13C NMR (151 MHz, CDCl3)
EST1001
f1 (ppm)

HPLC=0.1%TFA in H2O/ACN with 1.2mL/min

EM23001594423 08-Feb-2023 14:20:23 strubhe1-0208A-v04 LCMS-ZQ1 Kinetex column, ZQ1_10m_Acidic

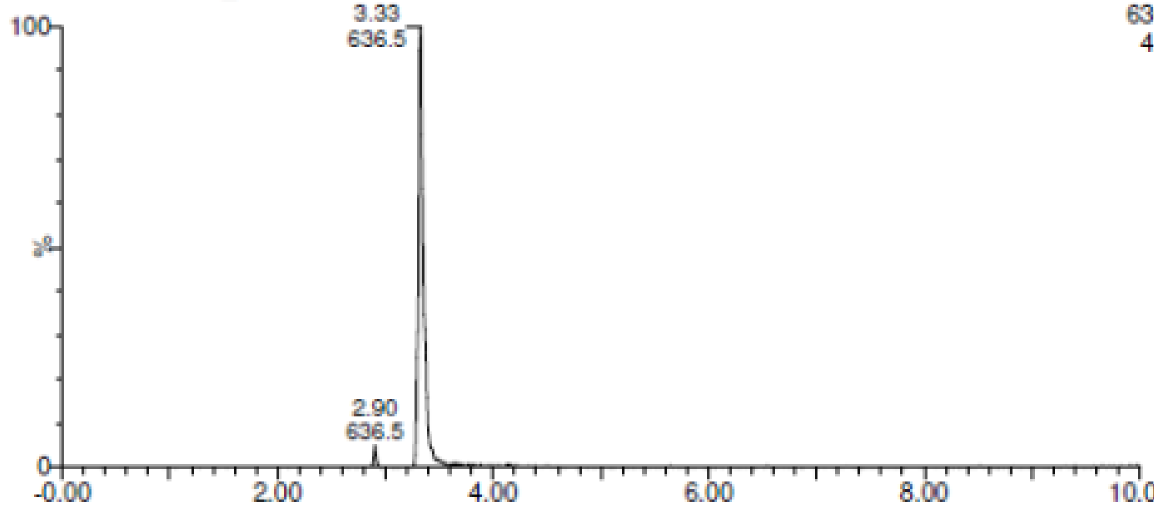

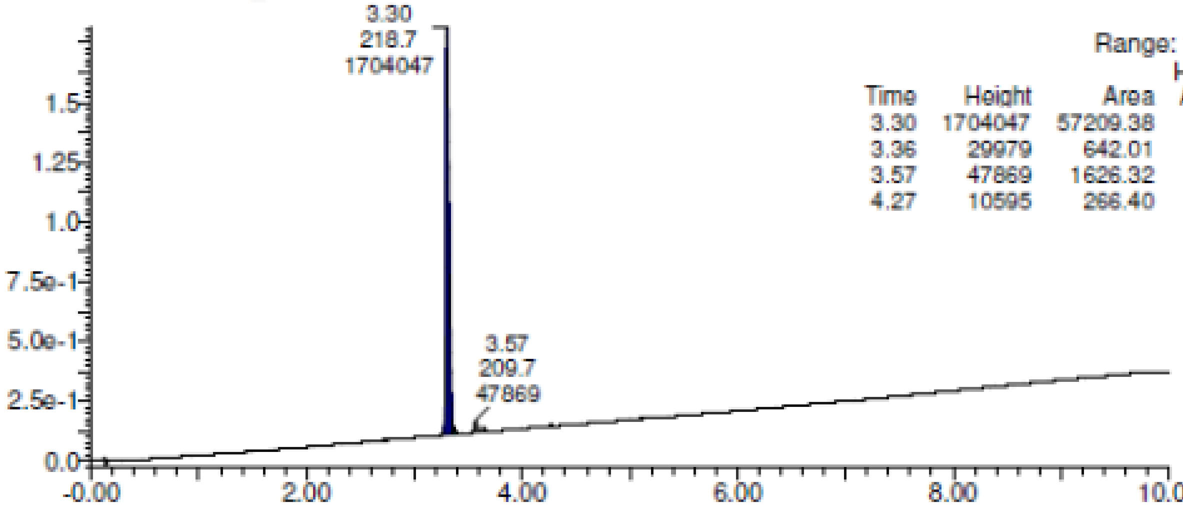

Time	Height	Area	Area%
3.30	1704047	57209.38	95.76
3.36	29979	642.01	1.07
3.57	47869	1626.32	2.72
4.27	10595	266.40	0.45

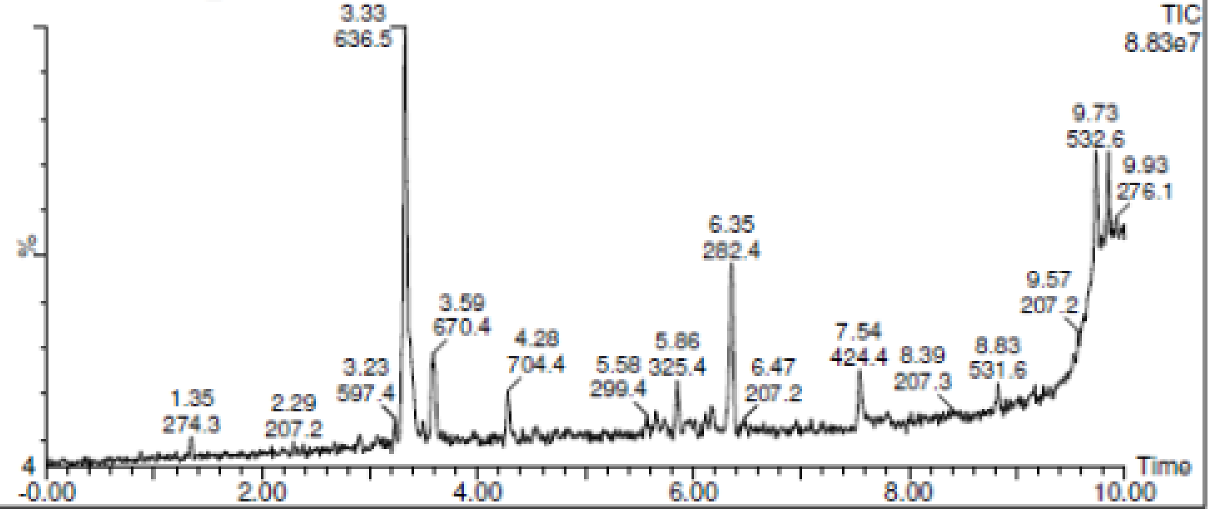

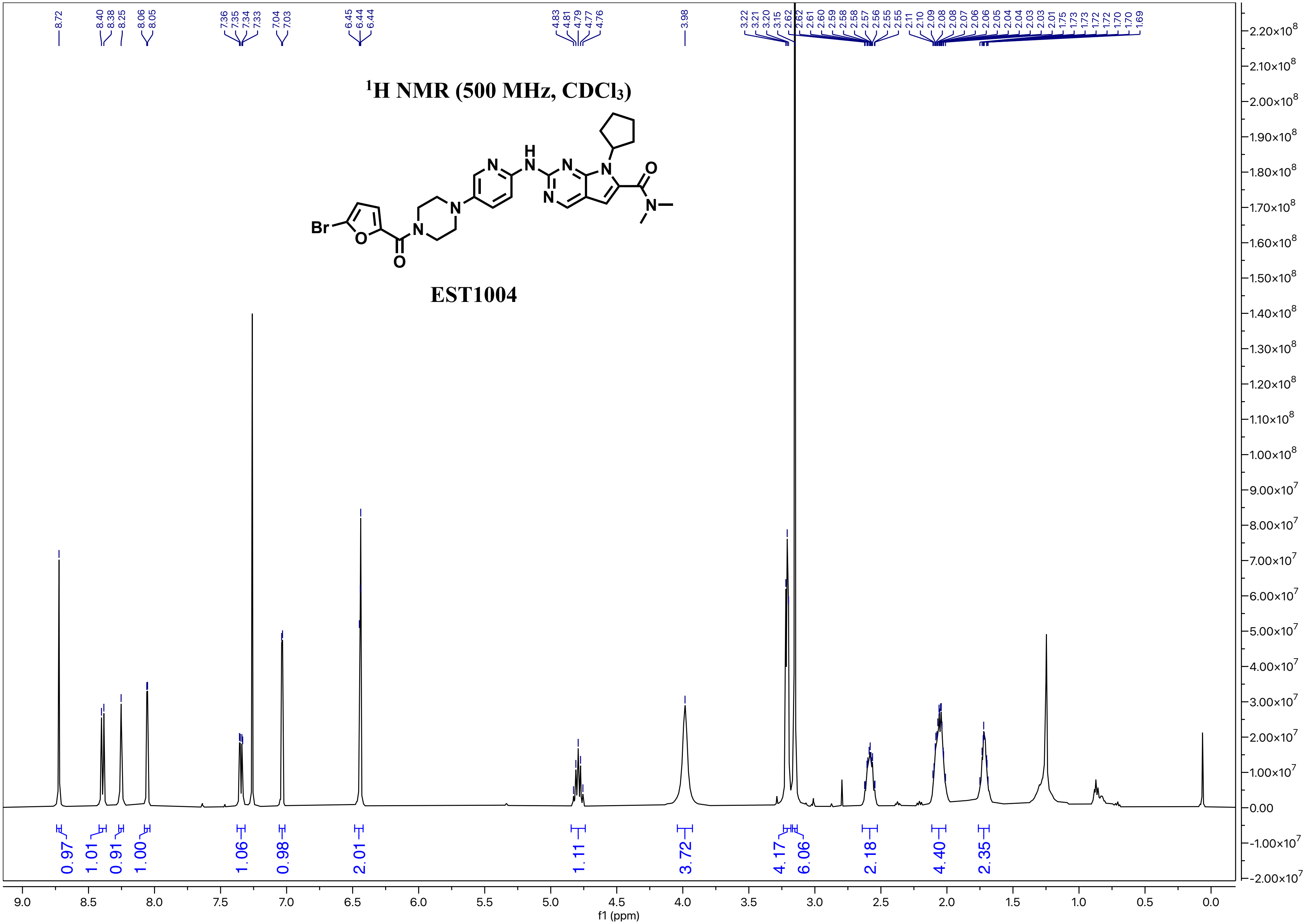
1H NMR (500 MHz, CDCl3)
Br
O
N
N
N
H
N
N
N
N
O
N
EST1004
f1 (ppm)

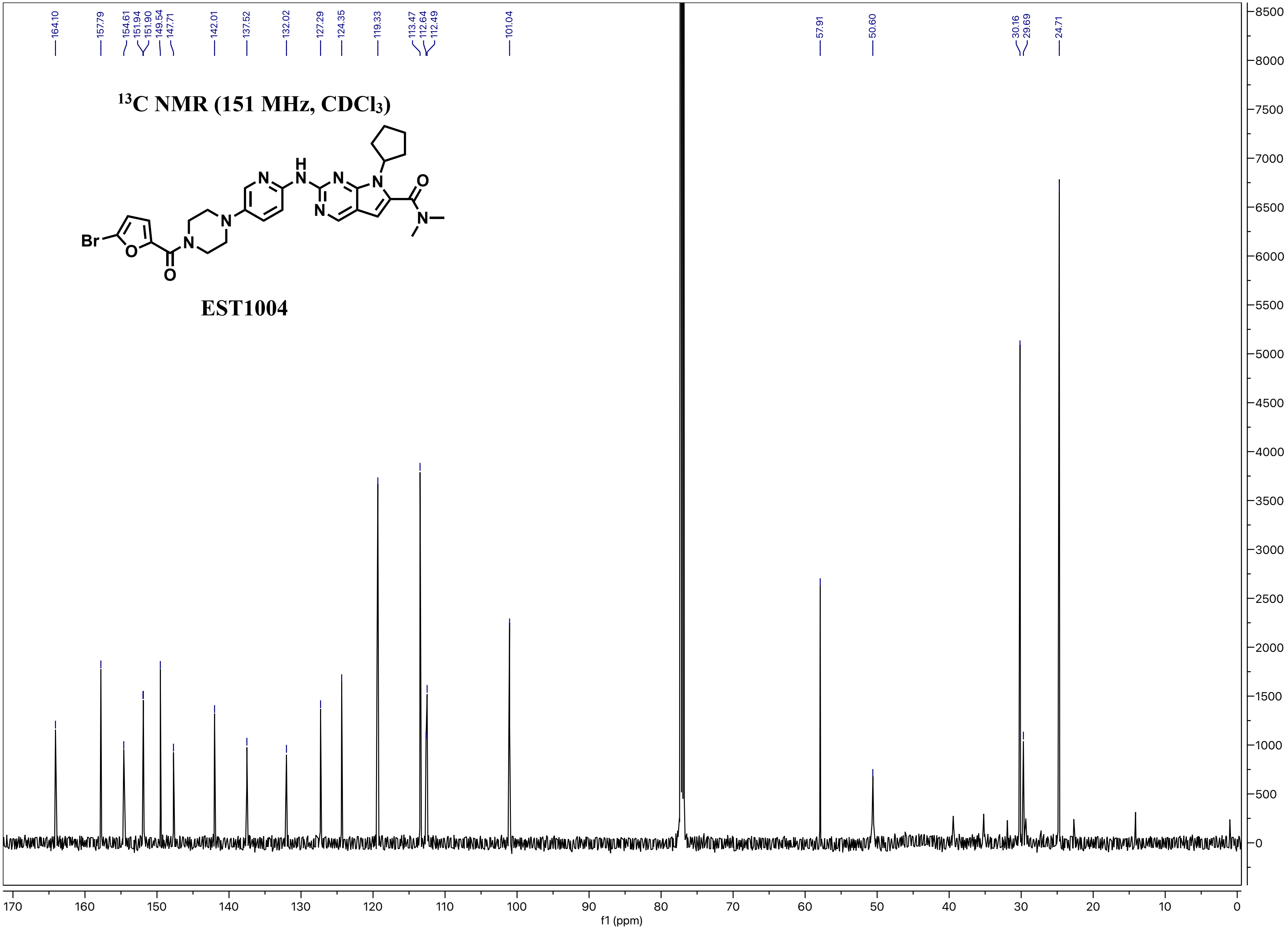
164.10
157.79
154.61
151.94
151.90
149.54
147.71
142.01
137.52
132.02
127.29
124.35
119.33
113.47
112.64
112.49
101.04
57.91
50.60
30.16
29.69
24.71
13C NMR (151 MHz, CDCl3)
EST1004
f1 (ppm)

HPLC=0.1%TFA in H2O/ACN with 1.2mL/min

EM23001594424 | **08-Feb-2023 14:31:26 strubhe1-0208A-v05** | **LCMS-ZQ1 Kinetex column, ZQ1_10m_Acidic**

HESSEMA4-101-EXP220-S01_QC

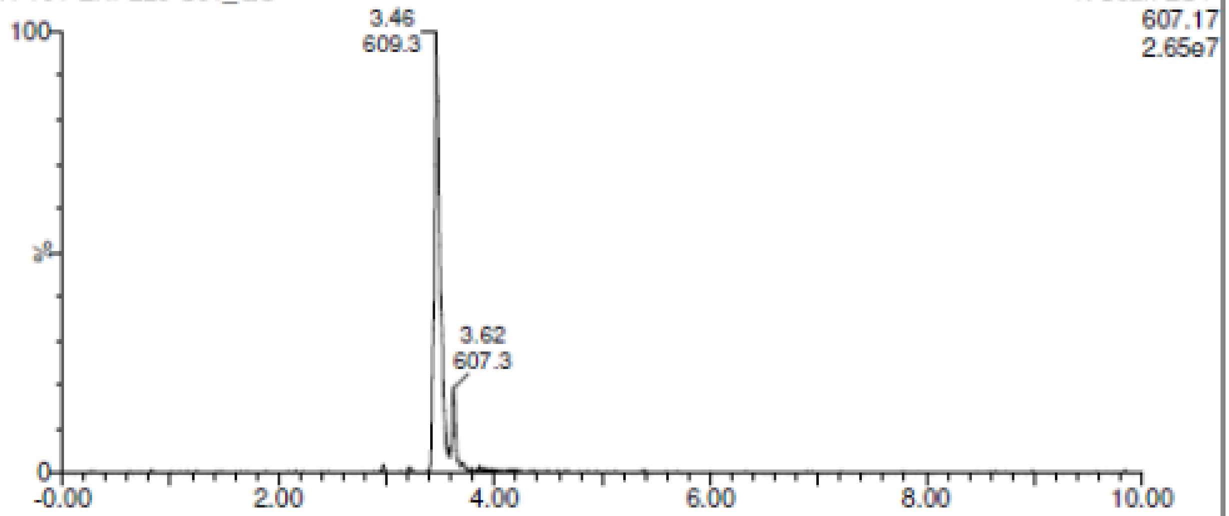

HESSEMA4-101-EXP220-S01_QC

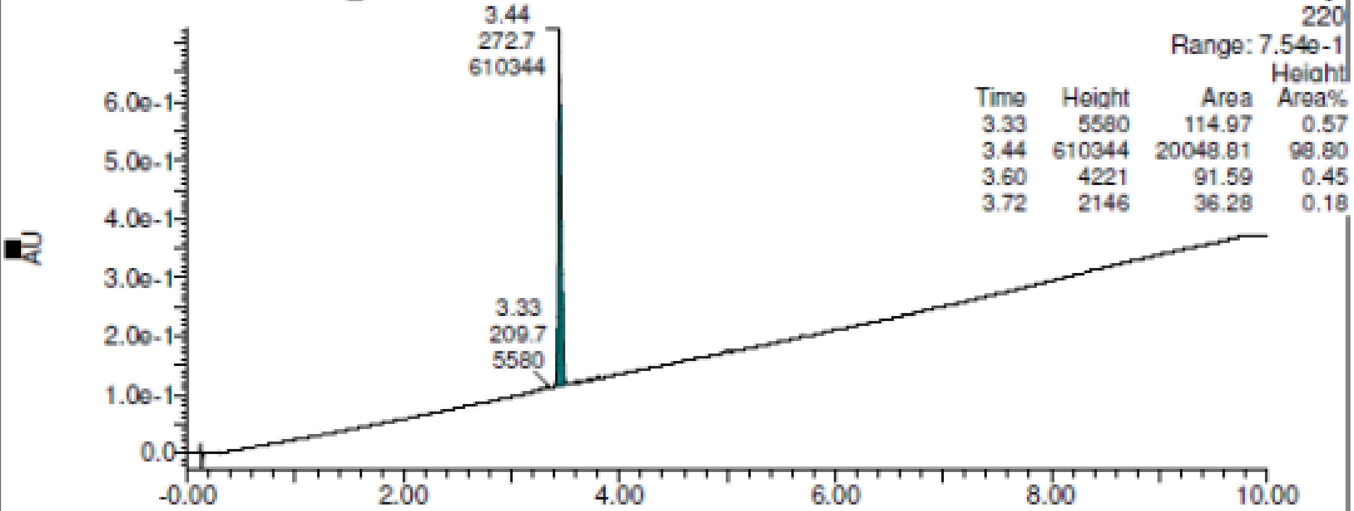

Time	Height	Area	Height Area%
3.33	5580	114.97	0.57
3.44	610344	20048.81	98.80
3.60	4221	91.59	0.45
3.72	2146	36.28	0.18

HESSEMA4-101-EXP220-S01_QC

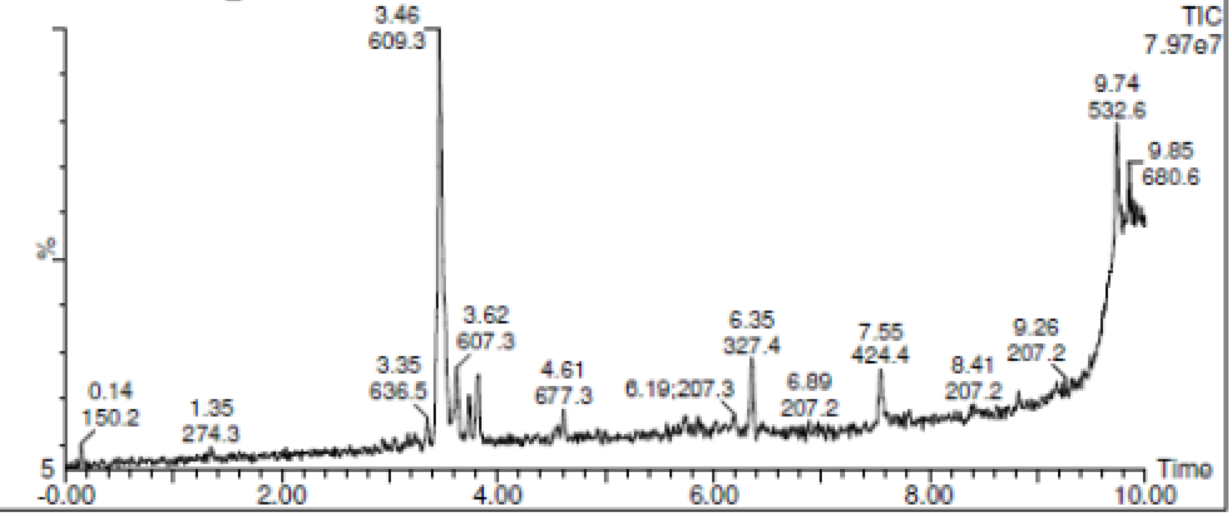

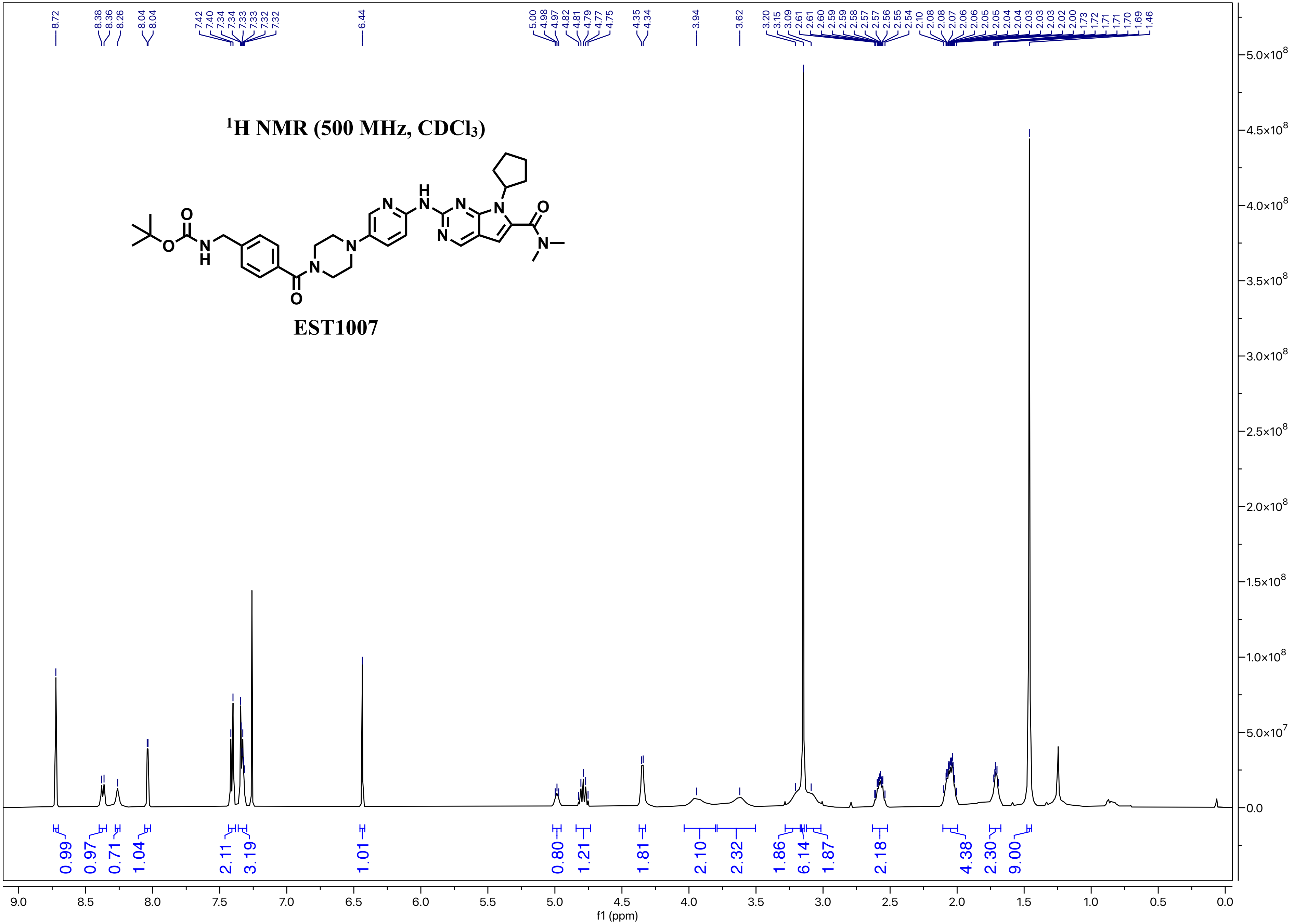

1H NMR (500 MHz, CDCl3)
EST1007
f1 (ppm)

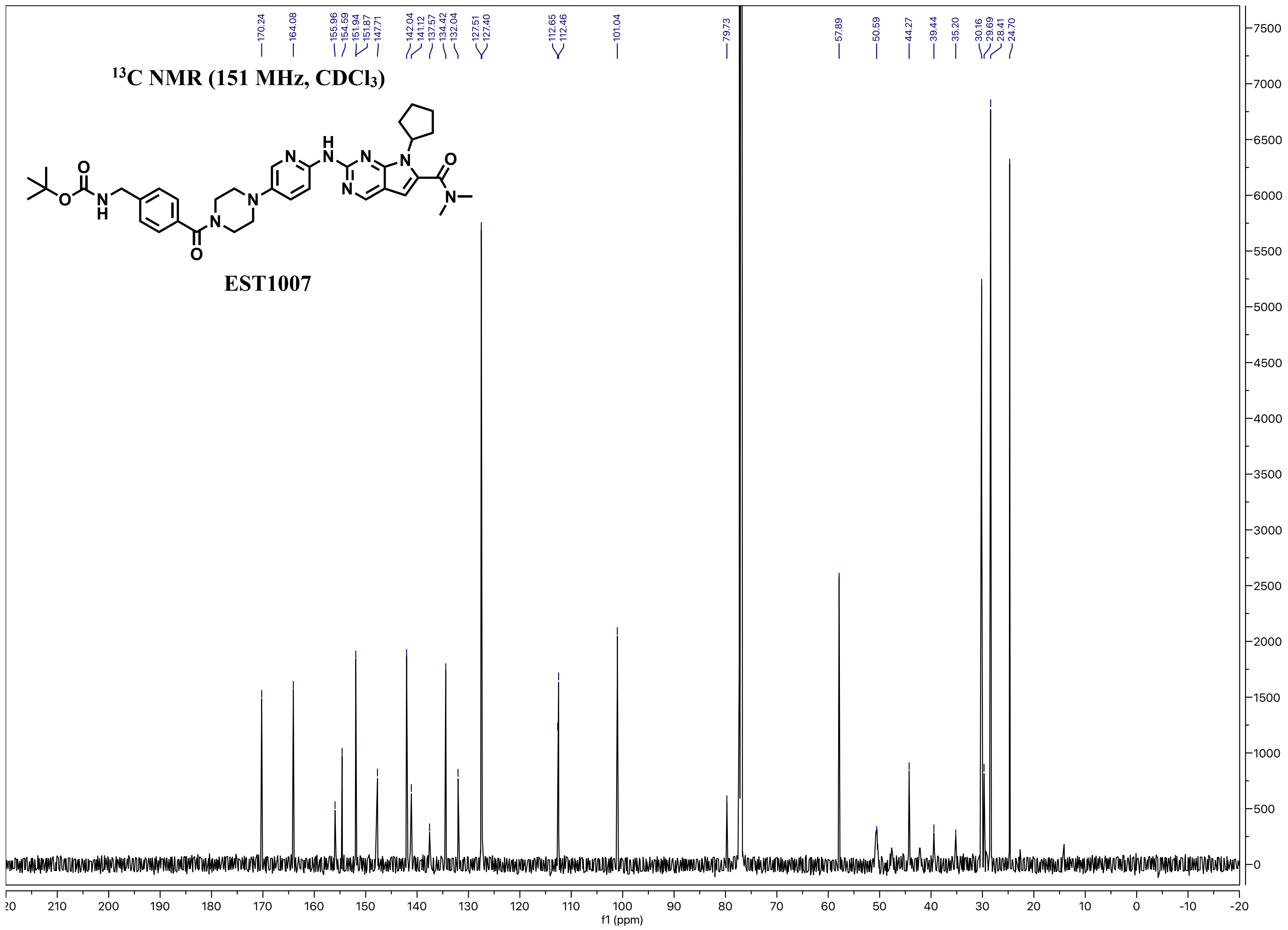

13C NMR (151 MHz, CDCl3)
EST1007
170.24
164.08
155.96
154.59
151.94
151.87
147.71
142.04
141.12
137.57
134.42
132.04
127.51
127.40
112.65
112.46
101.04
79.73
57.89
50.59
44.27
39.44
35.20
30.16
29.69
28.41
24.70
f1 (ppm)

HPLC=0.1%TFA in H2O/ACN with 1.2mL/min

EM23001594425 | 08-Feb-2023 14:42:29 | strubhe1-0208A-v06 | LCMS-ZQ1 Kinetex column, ZQ1_10m_Acidic

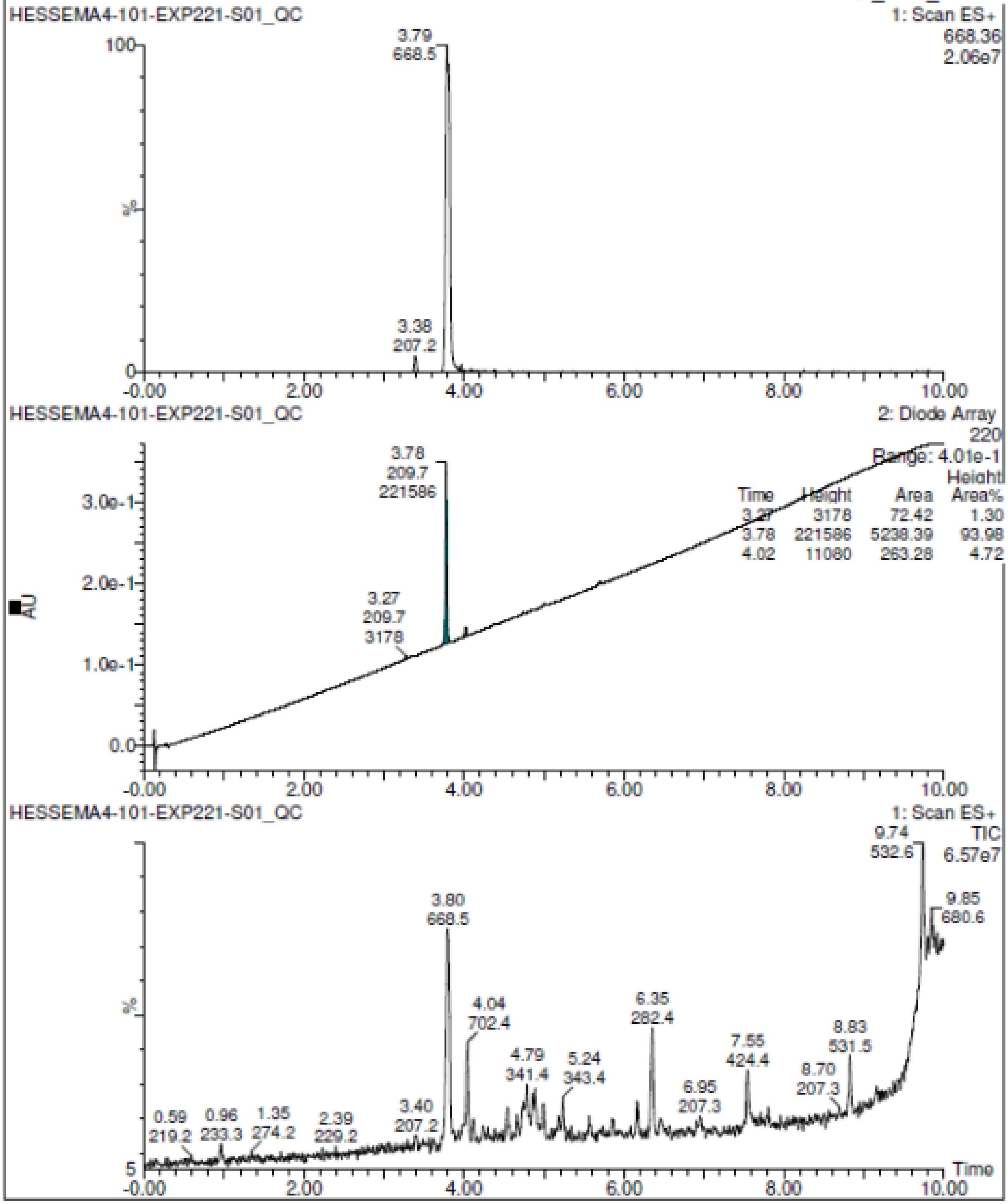

Time	Height	Area	Height Area%
3.27	3178	72.42	1.30
3.78	221586	5238.39	93.98
4.02	11080	263.28	4.72

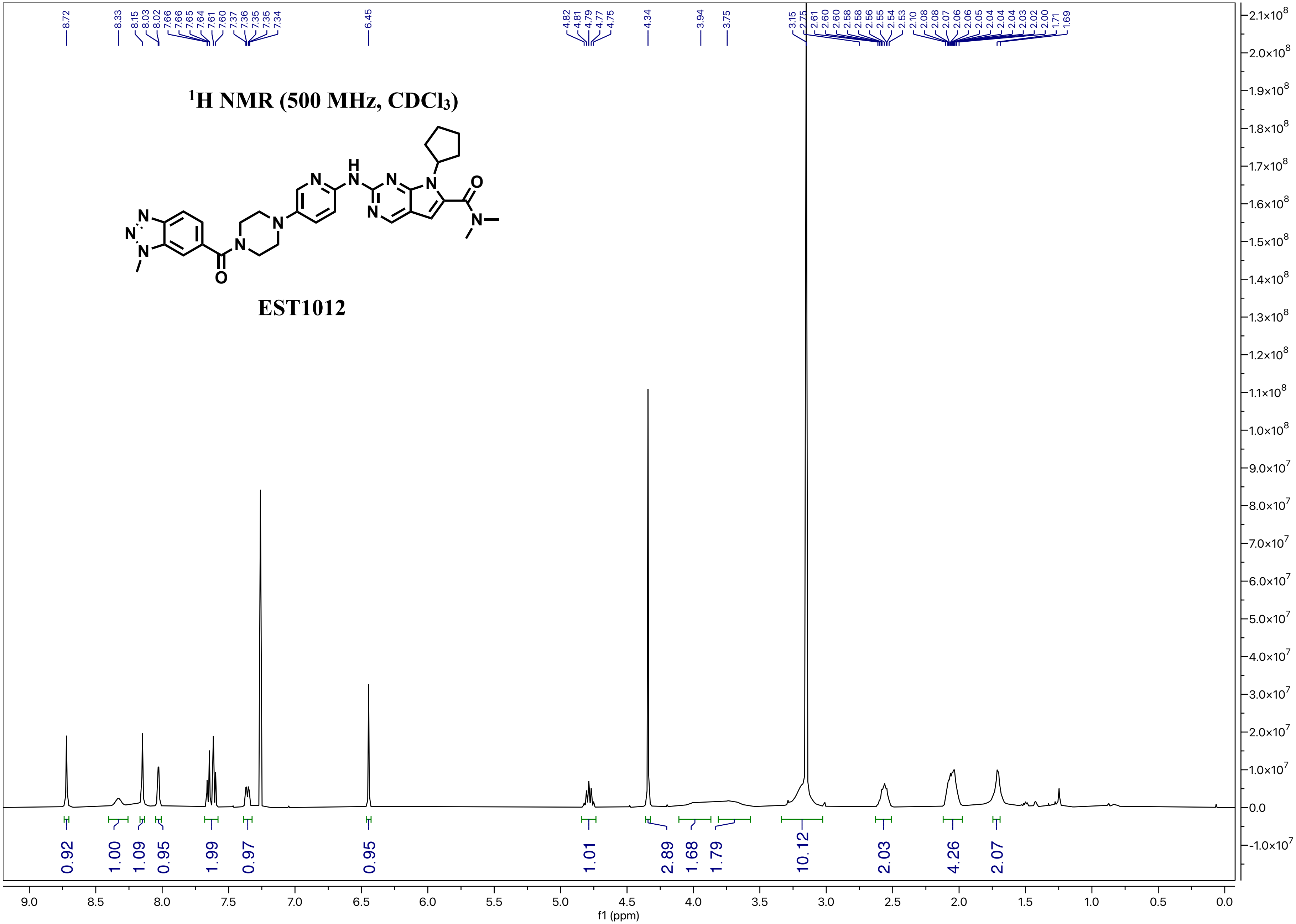

^{1}H NMR (500 MHz, $CDCl_3$)
EST1012
8.72
8.33
8.15
8.03
8.02
7.66
7.66
7.65
7.64
7.61
7.60
7.37
7.36
7.35
7.35
7.34
6.45
4.82
4.81
4.79
4.77
4.75
4.34
3.94
3.75
3.15
2.75
2.61
2.60
2.60
2.58
2.58
2.56
2.55
2.54
2.53
2.10
2.08
2.08
2.07
2.06
2.06
2.05
2.04
2.04
2.04
2.03
2.02
2.00
1.71
1.69
0.92
1.00
1.09
0.95
1.99
0.97
0.95
1.01
2.89
1.68
1.79
10.12
2.03
4.26
2.07
f1 (ppm)

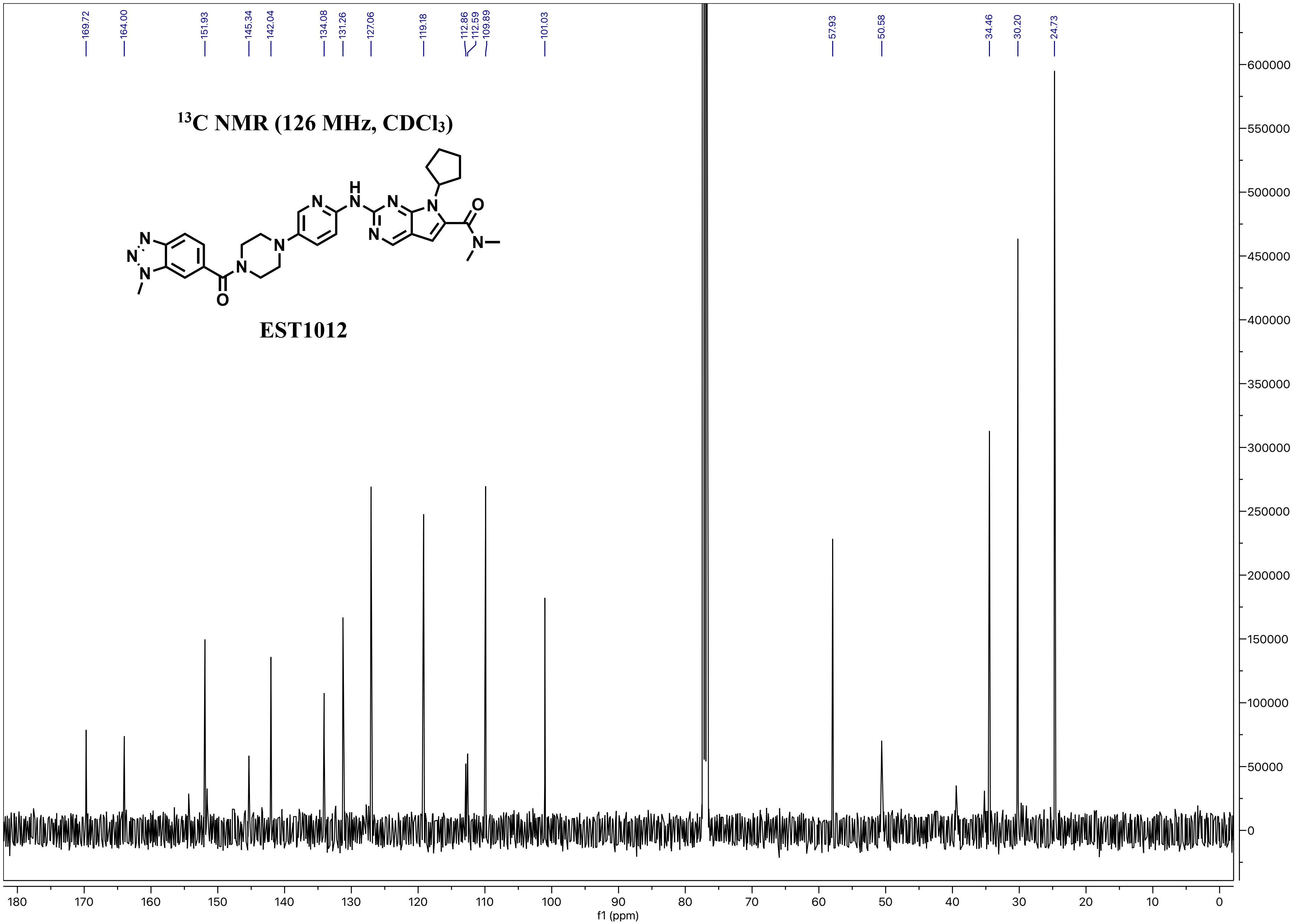
169.72
164.00
151.93
145.34
142.04
134.08
131.26
127.06
119.18
112.86
112.59
109.89
101.03
57.93
50.58
34.46
30.20
24.73
^{13}C NMR (126 MHz, CDCl$_3$)
EST1012
f1 (ppm)

HPLC=0.1%TFA in H2O/ACN with 1.2mL/min

EM23001594427 08-Feb-2023 14:53:33 strubhe1-0208A-v07 LCMS-ZQ1 Kinetex column, ZQ1_10m_Acidic

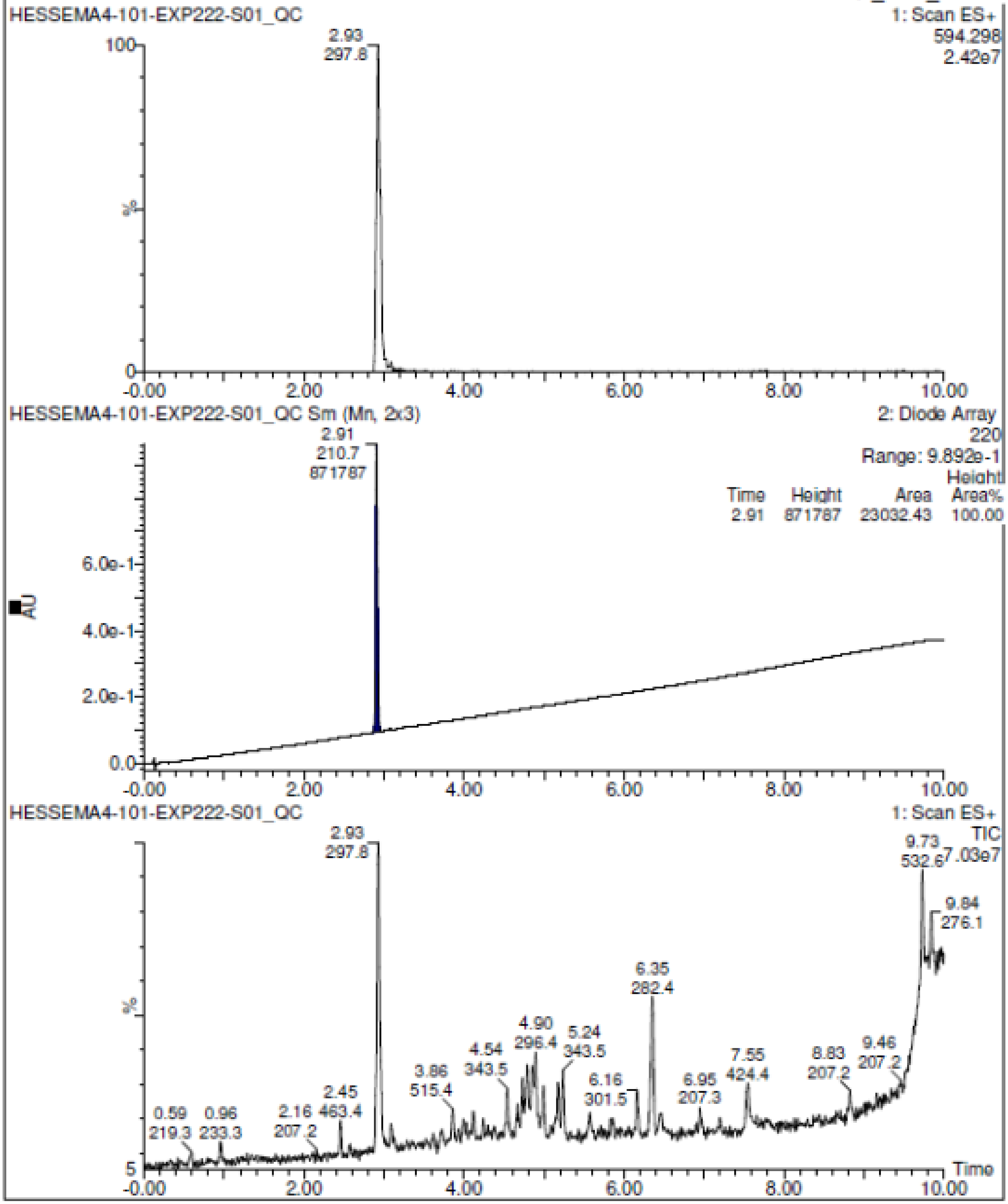

^{1}H NMR (500 MHz, CDCl$_3$)

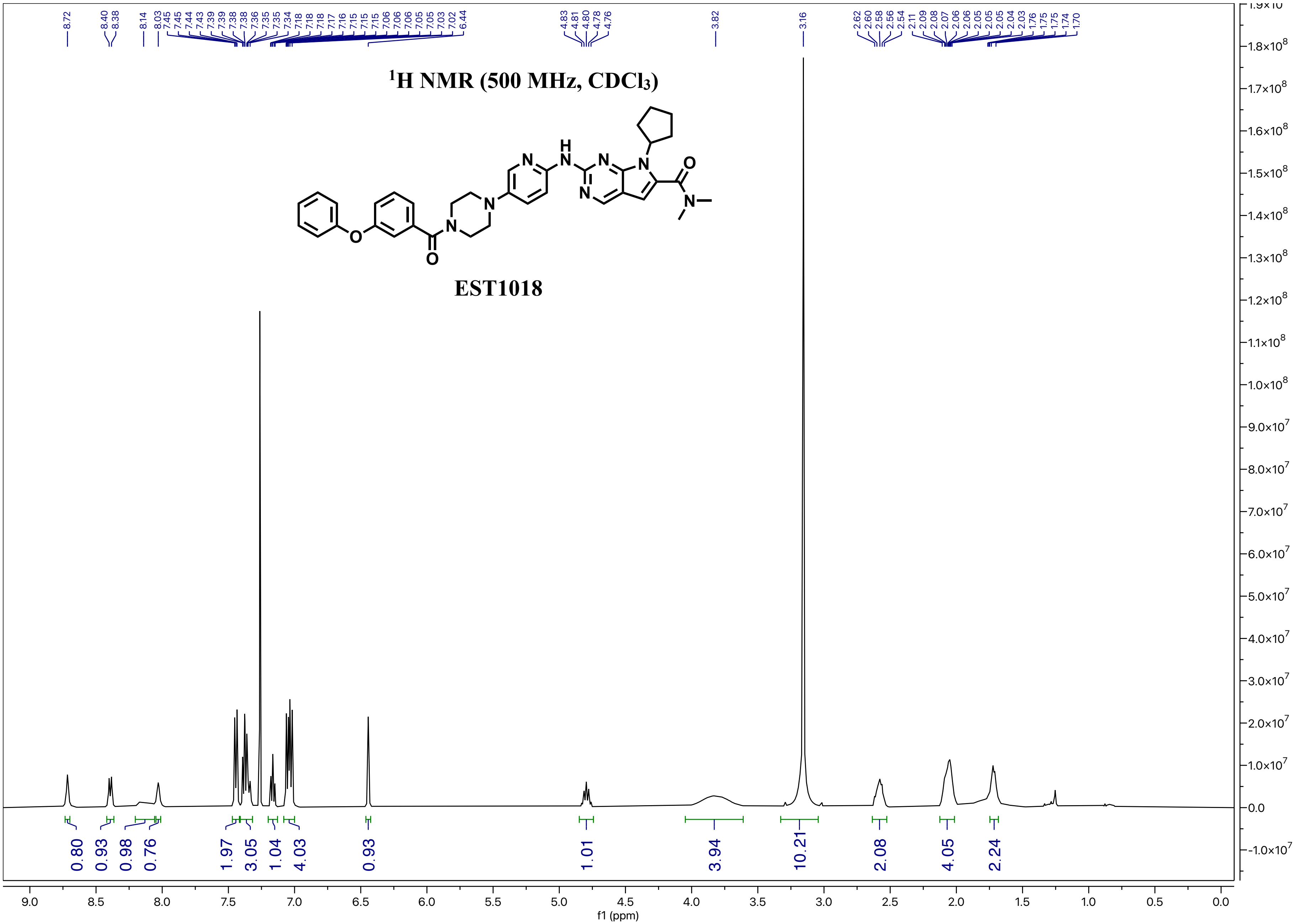

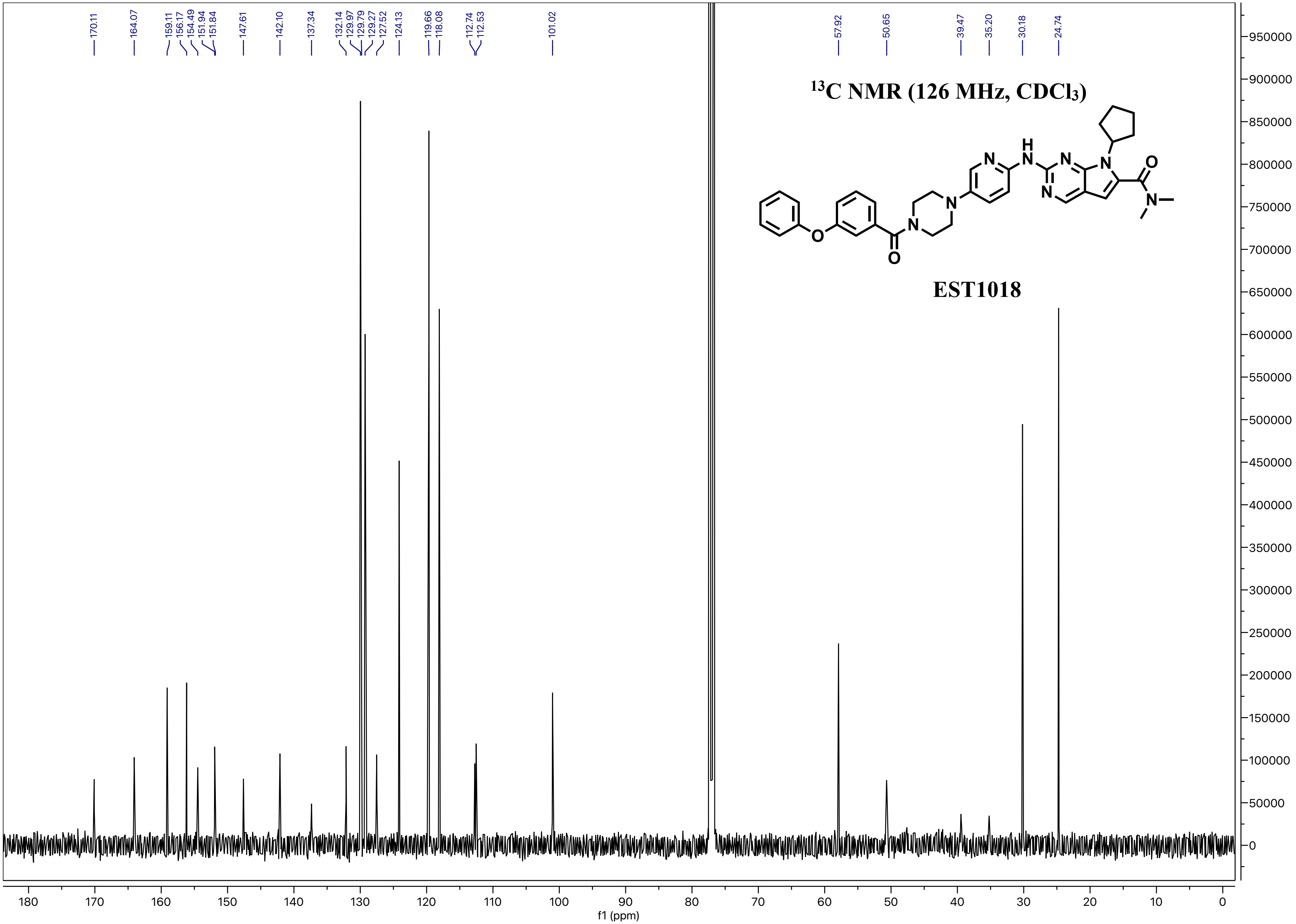

170.11
164.07
159.11
156.17
154.49
151.94
151.84
147.61
142.10
137.34
132.14
129.97
129.79
129.27
127.52
124.13
119.66
118.08
112.74
112.53
101.02
57.92
50.65
39.47
35.20
30.18
24.74
^{13}C NMR (126 MHz, $CDCl_3$)
EST1018
f1 (ppm)

HPLC=0.1%TFA in H2O/ACN with 1.2mL/min

EM23001594428 | 08-Feb-2023 15:04:37 strubhe1-0208A-v08 | LCMS-ZQ1 Kinetex column, ZQ1_10m_Acidic

HESSEMA4-101-EXP223-S01_QC

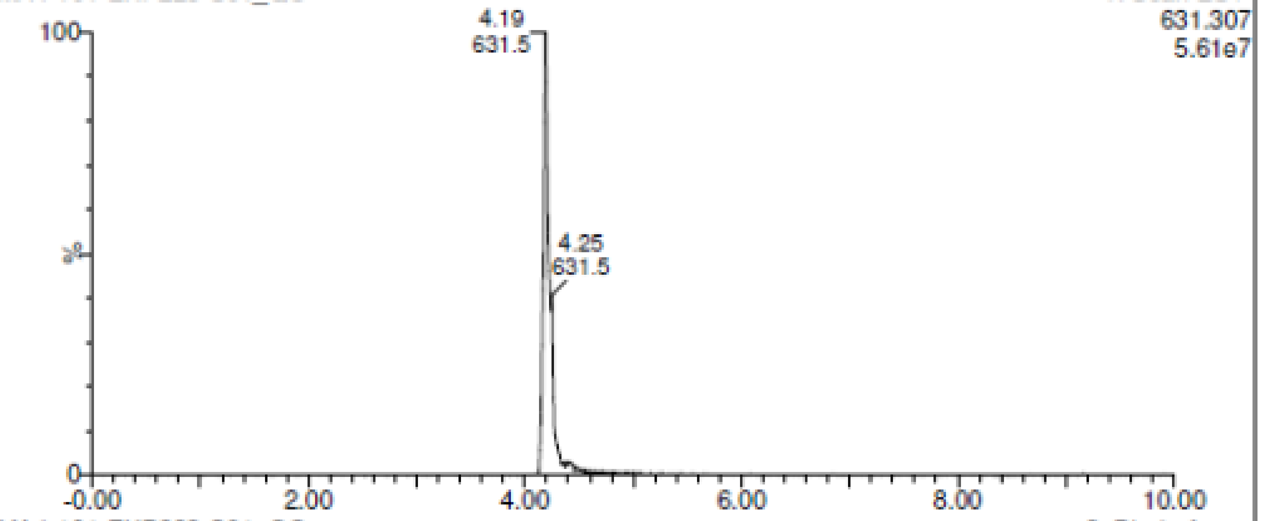

HESSEMA4-101-EXP223-S01_QC

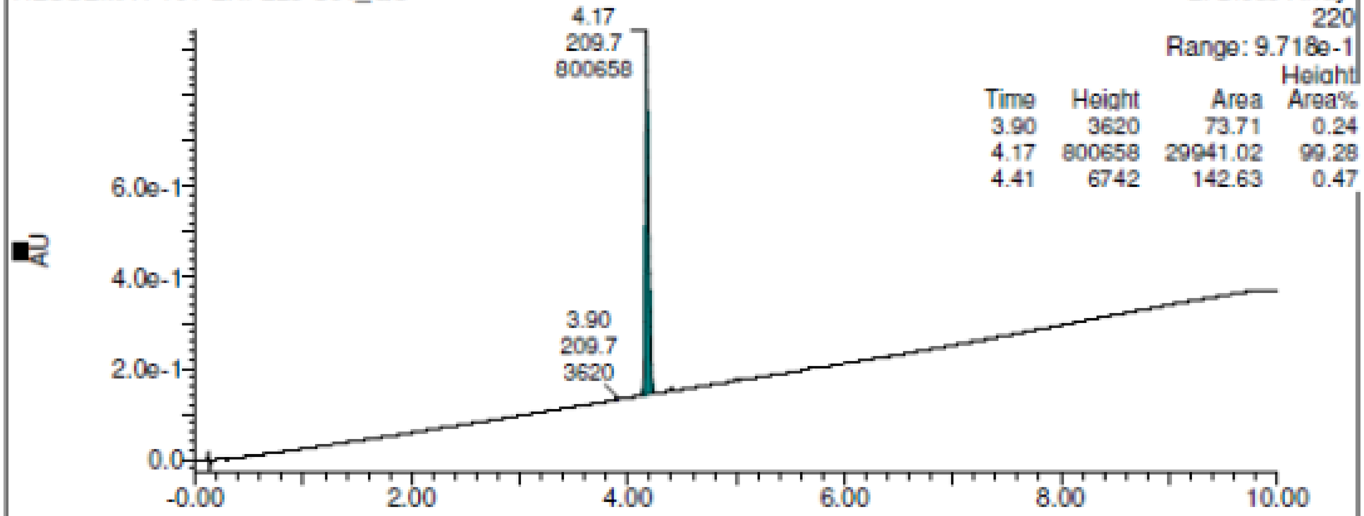

Time	Height	Area	Height Area%
3.90	3620	73.71	0.24
4.17	800658	29941.02	99.28
4.41	6742	142.63	0.47

HESSEMA4-101-EXP223-S01_QC

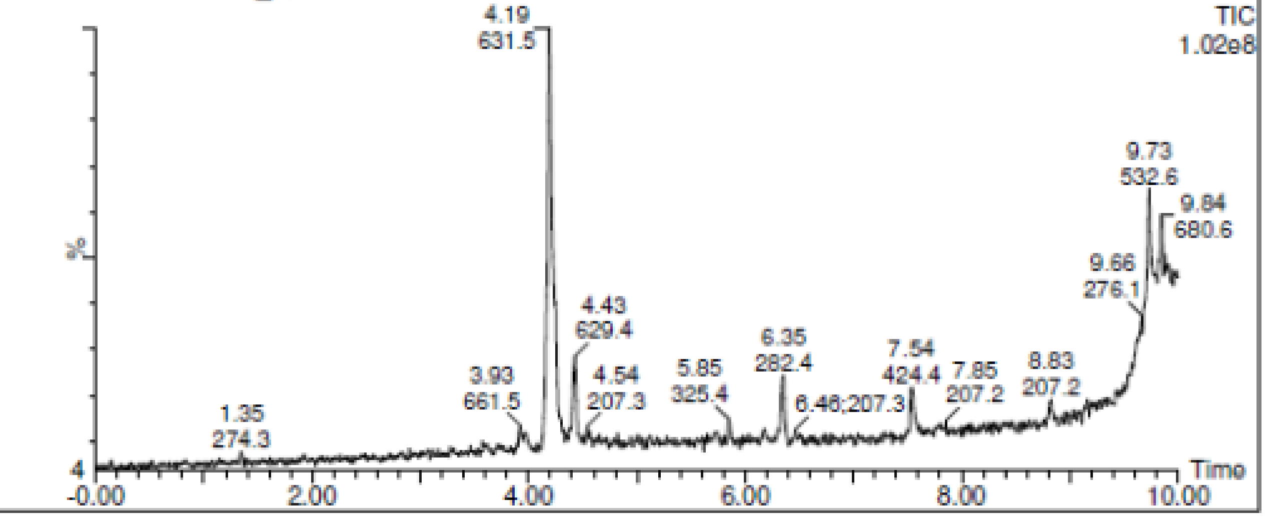

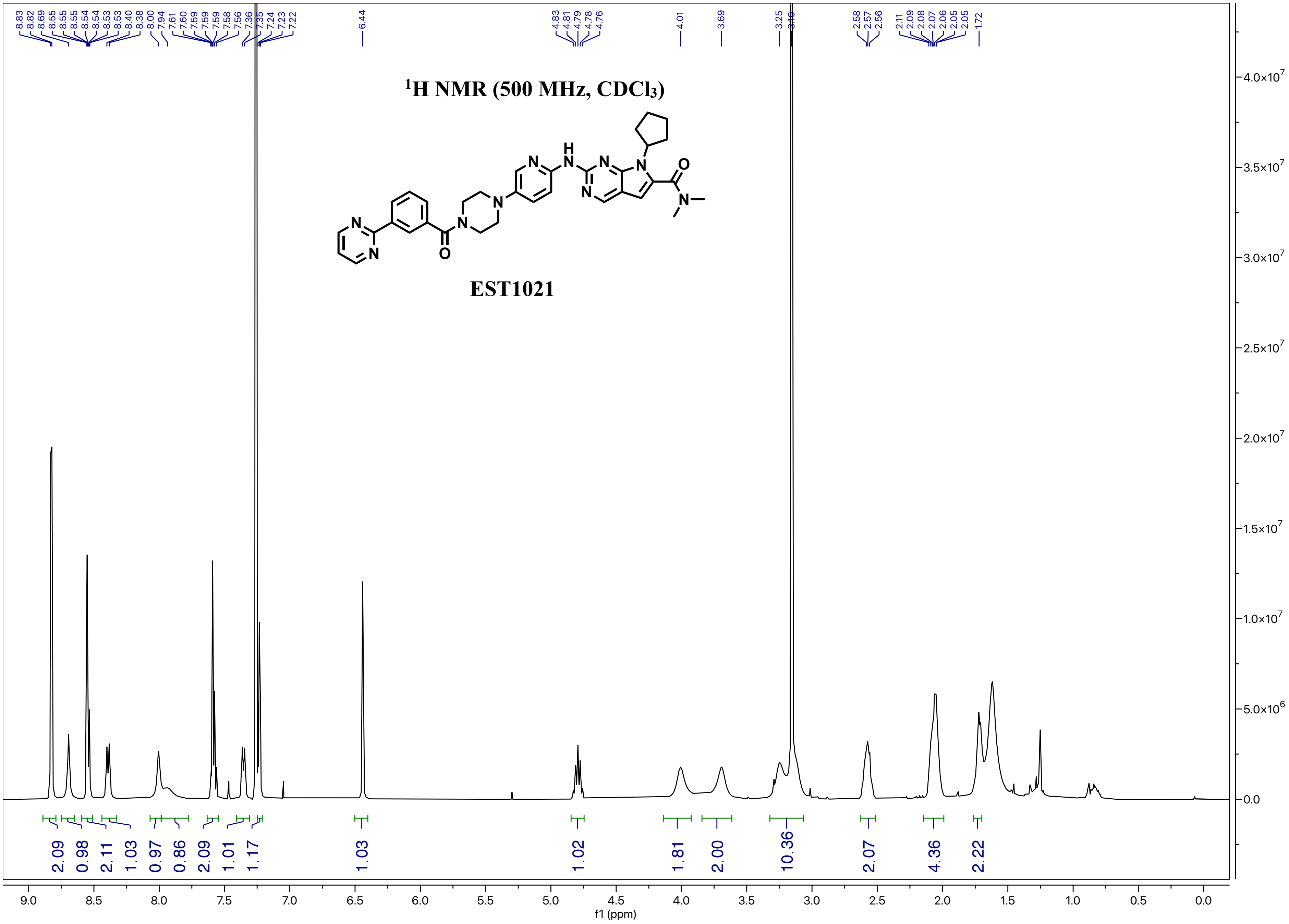

1H NMR (500 MHz, CDCl3)
EST1021
f1 (ppm)

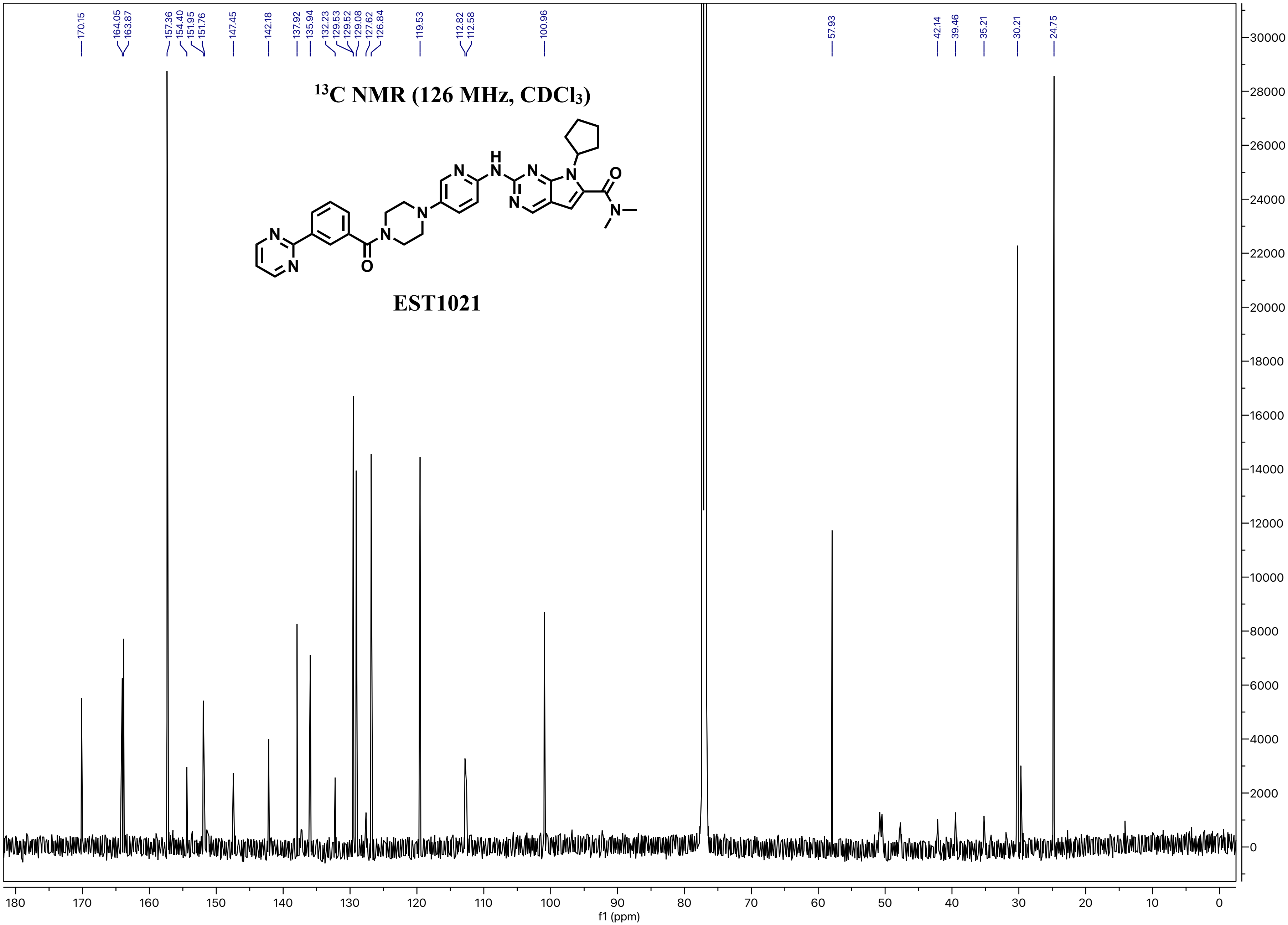

170.15
164.05
163.87
157.36
154.40
151.95
151.76
147.45
142.18
137.92
135.94
132.23
129.53
129.52
129.08
127.62
126.84
119.53
112.82
112.58
100.96
57.93
42.14
39.46
35.21
30.21
24.75
¹³C NMR (126 MHz, CDCl₃)
EST1021
f1 (ppm)

HPLC=0.1%TFA in H2O/ACN with 1.2mL/min

EM23001594429 | 08-Feb-2023 15:15:41 strubhe1-0208A-v09 | LCMS-ZQ1 Kinetex column, ZQ1_10m_Acidic

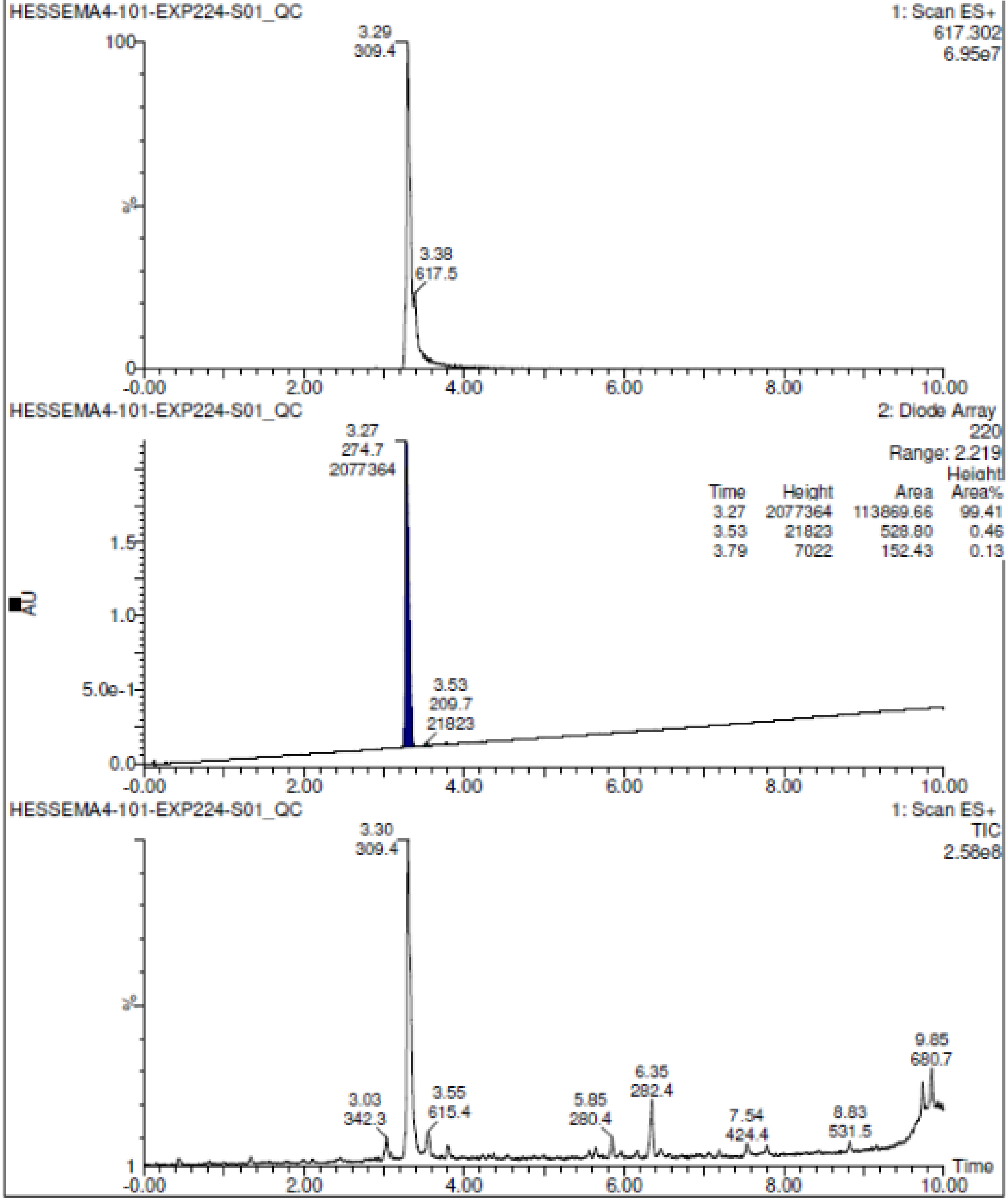

Time	Height	Area	Height Area%
3.27	2077364	113869.66	99.41
3.53	21823	528.80	0.46
3.79	7022	152.43	0.13

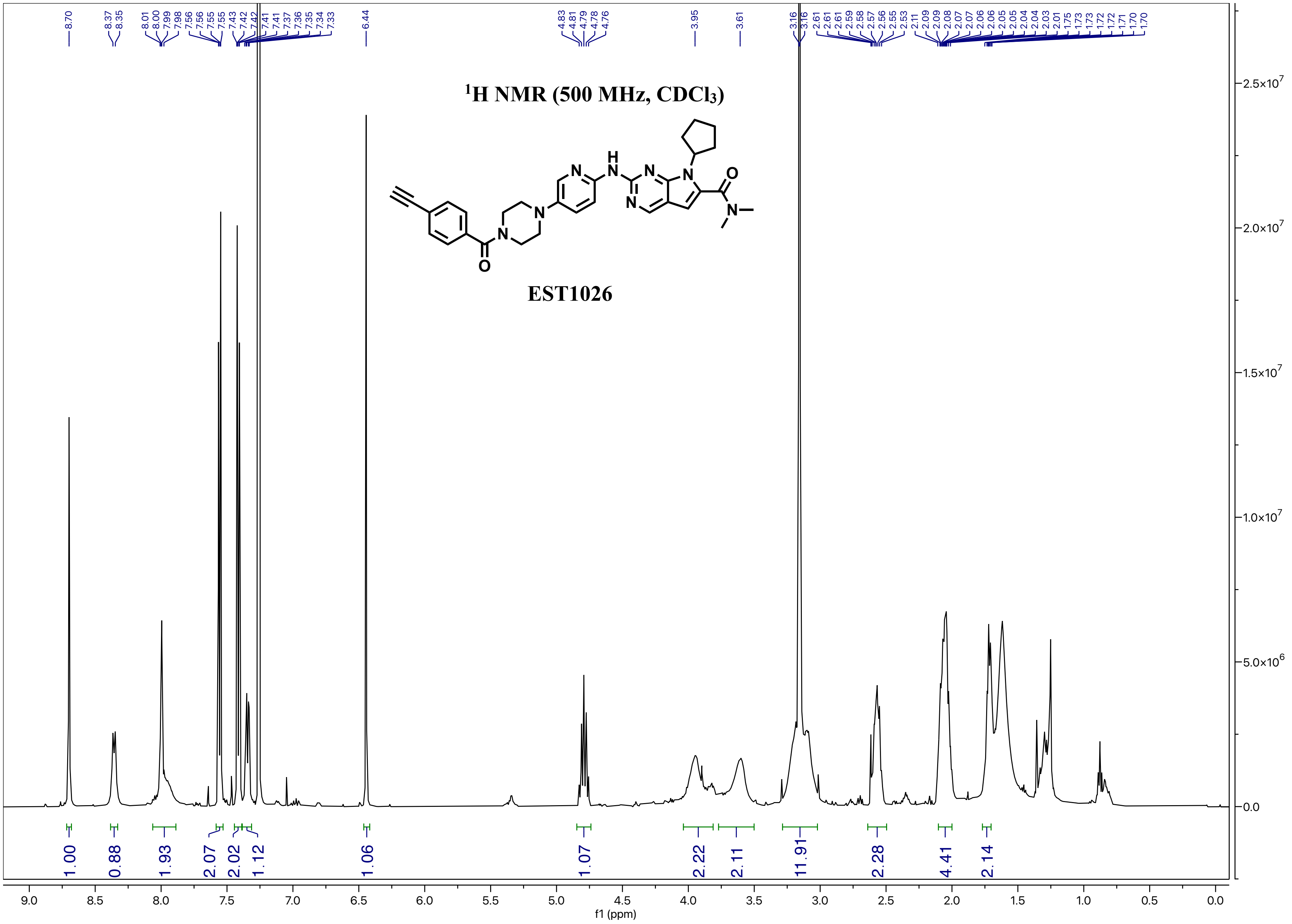
^{1}H NMR (500 MHz, $CDCl_3$)
EST1026
f1 (ppm)

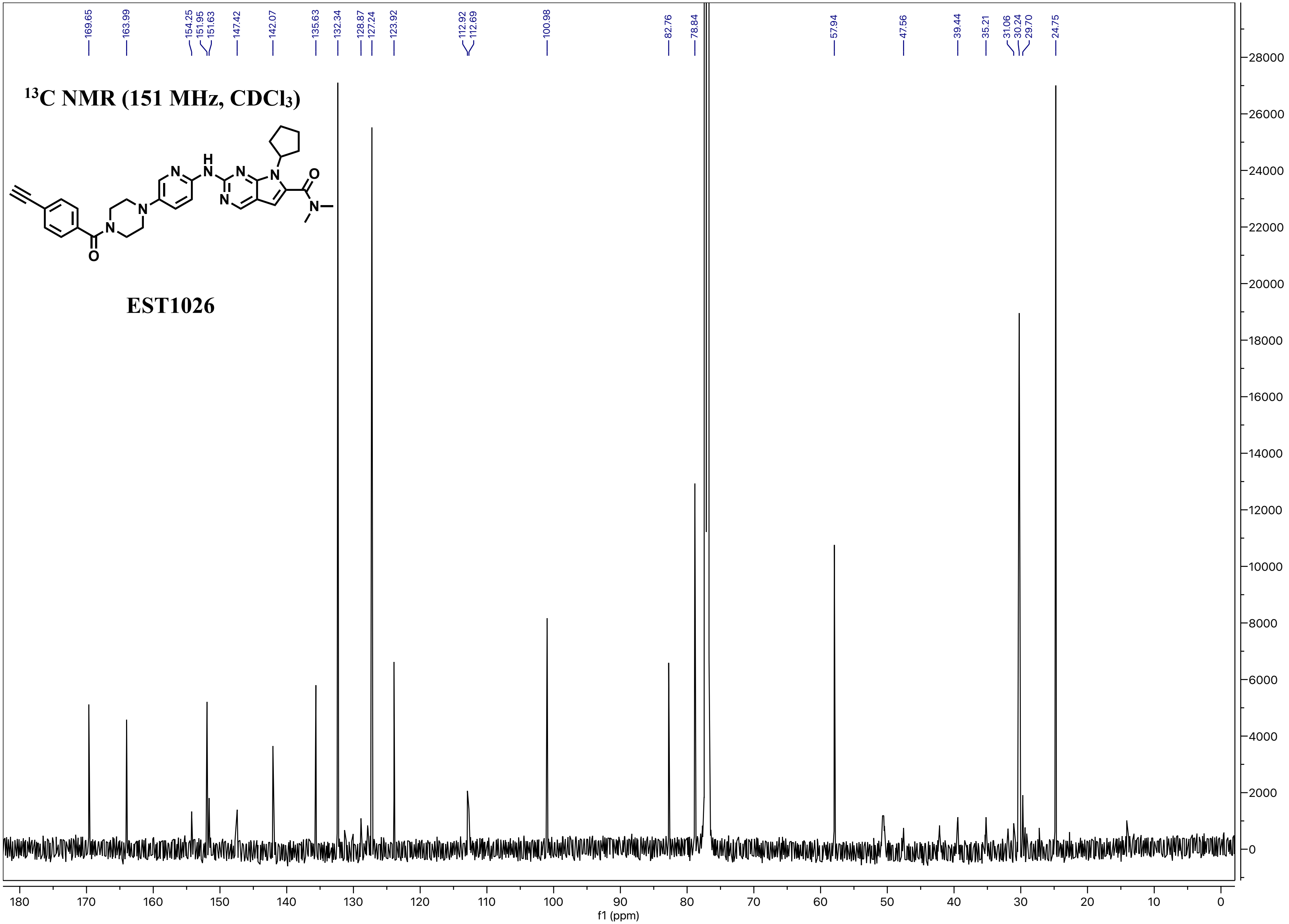

13C NMR (151 MHz, CDCl3)
EST1026
169.65
163.99
154.25
151.95
151.63
147.42
142.07
135.63
132.34
128.87
127.24
123.92
112.92
112.69
100.98
82.76
78.84
57.94
47.56
39.44
35.21
31.06
30.24
29.70
24.75
f1 (ppm)

HPLC=0.1%TFA in H2O/ACN with 1.2mL/min

EM23001594430 | 08-Feb-2023 15:26:43 strubhe1-0208A-v10 | LCMS-ZQ1 Kinetex column, ZQ1_10m_Acidic

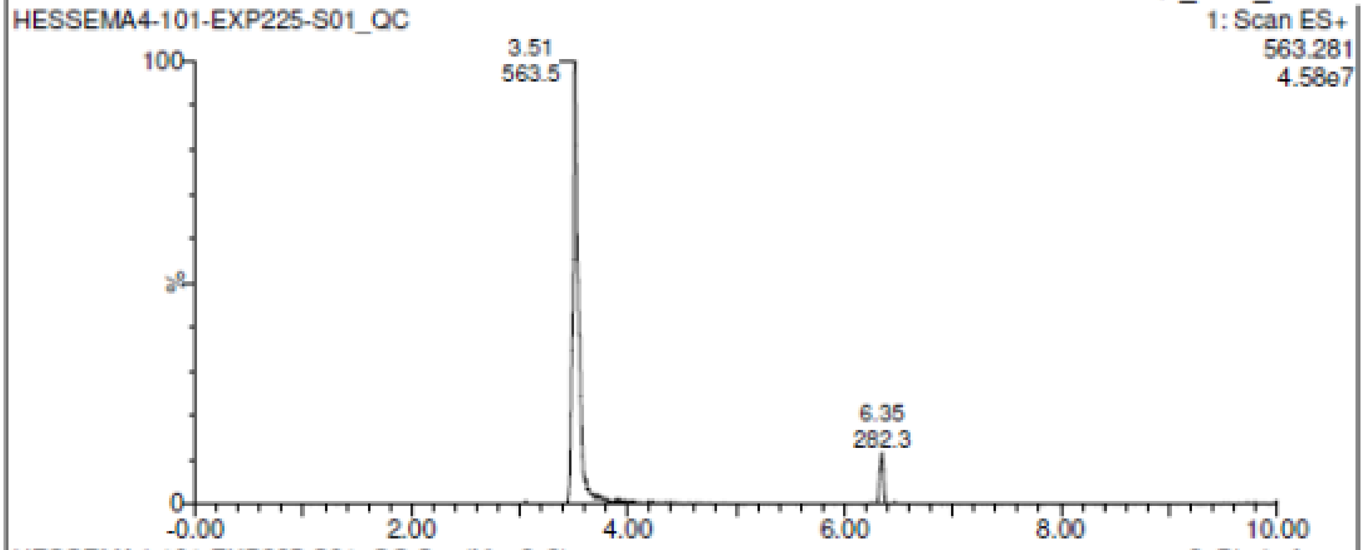

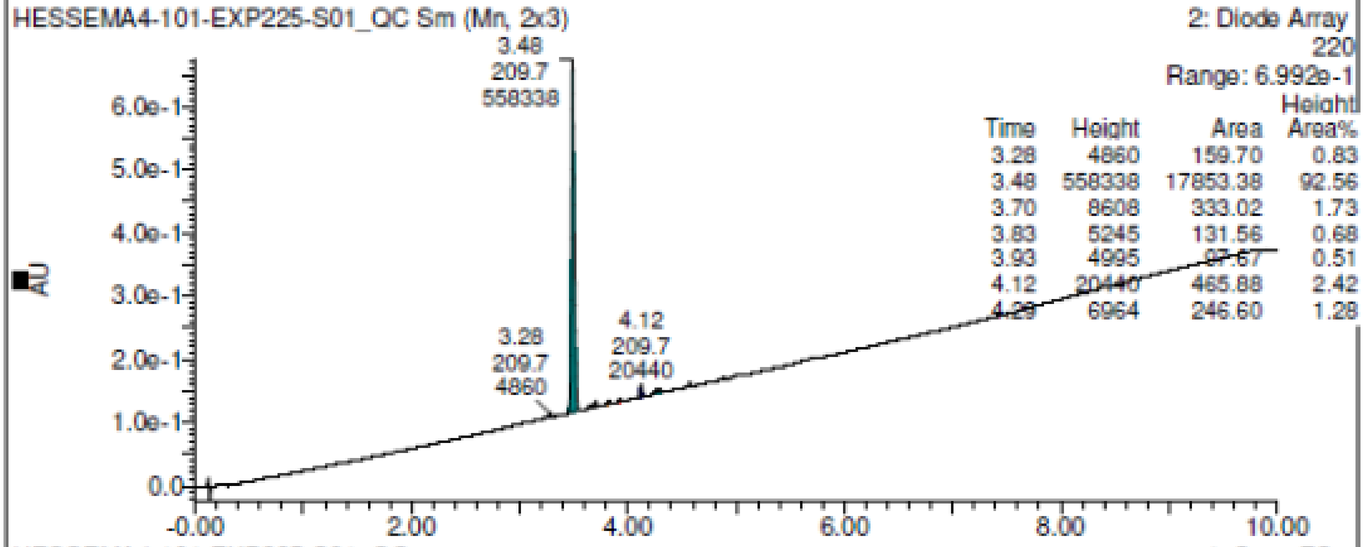

Time	Height	Area	Height Area%
3.28	4860	159.70	0.83
3.48	558338	17853.38	92.56
3.70	8608	333.02	1.73
3.83	5245	131.56	0.68
3.93	4995	97.67	0.51
4.12	20440	465.88	2.42
4.29	6964	246.60	1.28

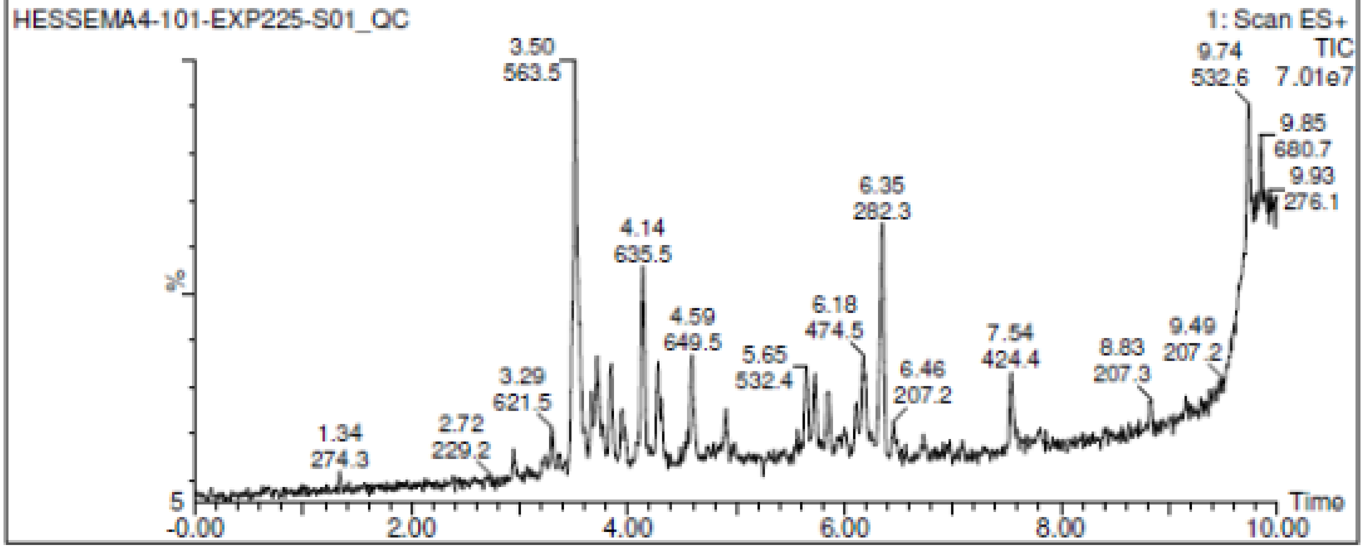

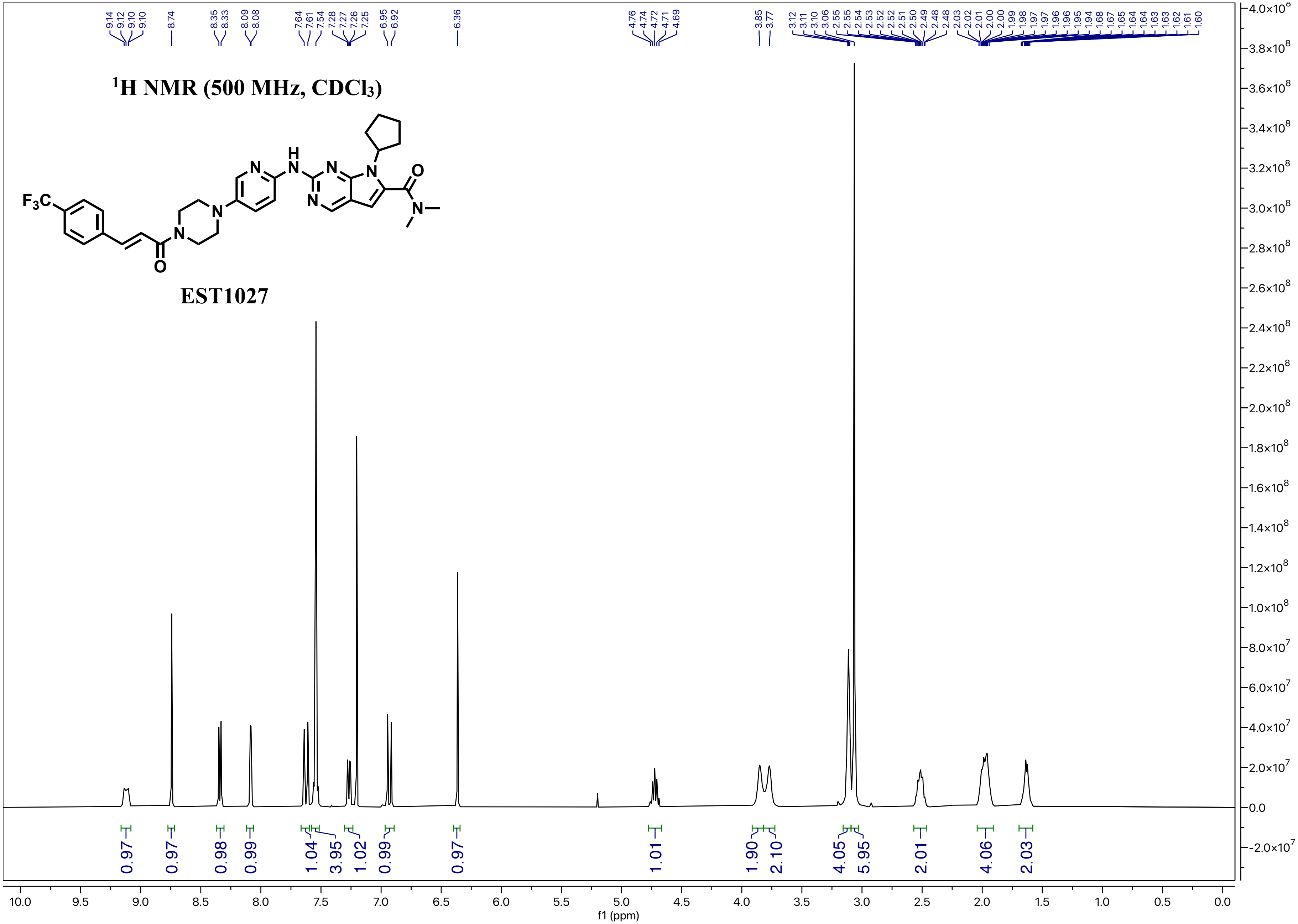

¹H NMR (500 MHz, CDCl₃)
EST1027
F3C
N
H
O
9.14
9.12
9.10
9.10
8.74
8.35
8.33
8.09
8.08
7.64
7.61
7.54
7.28
7.27
7.26
7.25
6.95
6.92
6.36
4.76
4.74
4.72
4.71
4.69
3.85
3.77
0.97
0.97
0.98
0.99
1.04
3.95
1.02
0.99
0.97
1.01
1.90
2.10
4.05
5.95
2.01
4.06
2.03
f1 (ppm)

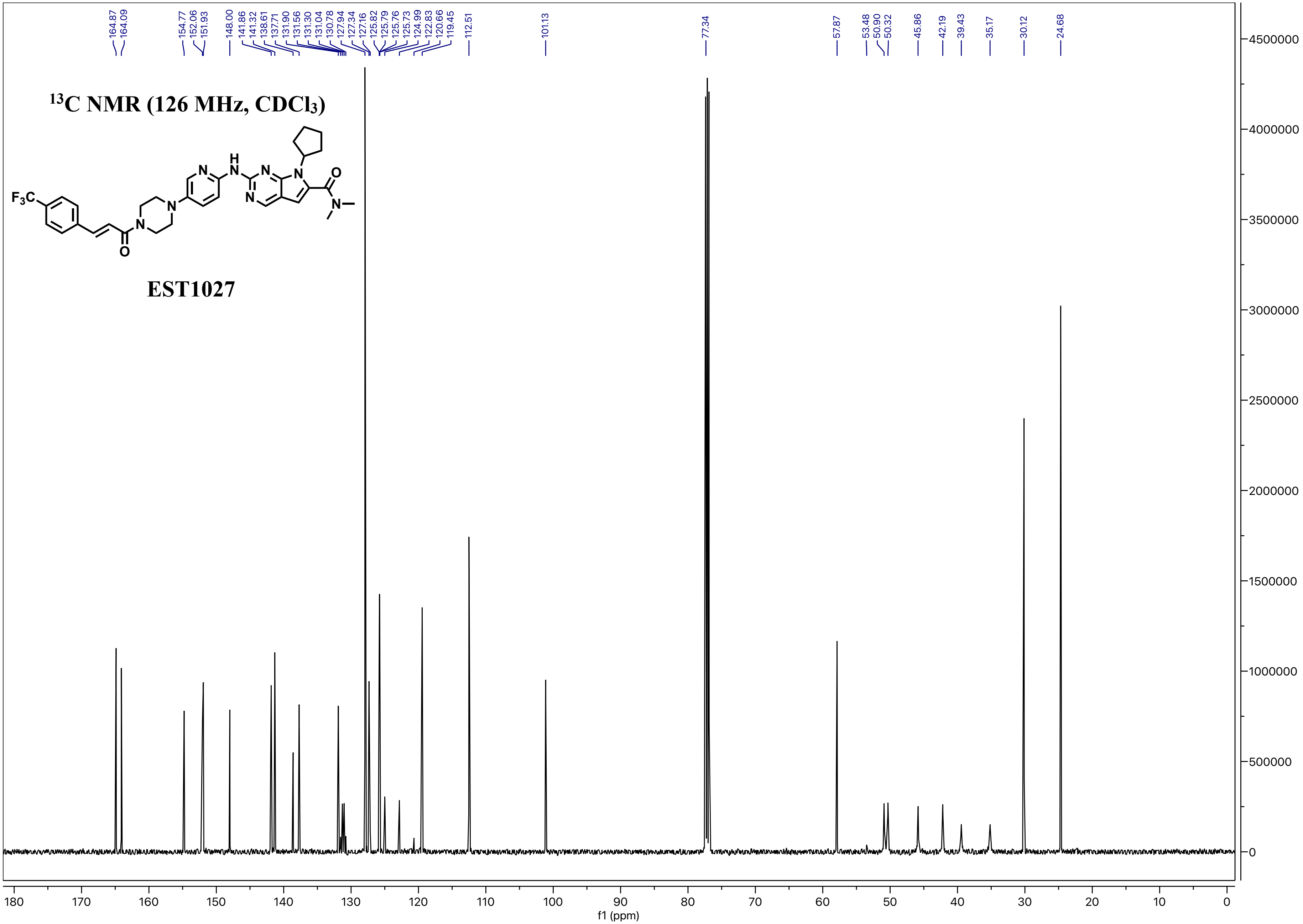

164.87
164.09
154.77
152.06
151.93
148.00
141.86
141.32
138.61
137.71
131.90
131.56
131.30
131.04
130.78
127.94
127.34
127.16
125.82
125.79
125.76
125.73
124.99
122.83
120.66
119.45
112.51
101.13
77.34
57.87
53.48
50.90
50.32
45.86
42.19
39.43
35.17
30.12
24.68
¹³C NMR (126 MHz, CDCl₃)
EST1027
f1 (ppm)

HPLC=0.1%TFA in H2O/ACN with 1.2mL/min

**EM23001594419**	**08-Feb-2023**<br>**13:46:34**<br>**strubhe1-0208A-v01**	**LCMS-ZQ1**<br>**Kinetex column,**<br>**ZQ1_10m_Acidic**

HESSEMA4-101-EXP216-S01_QC

1: Scan ES+
633.284
8.50e7

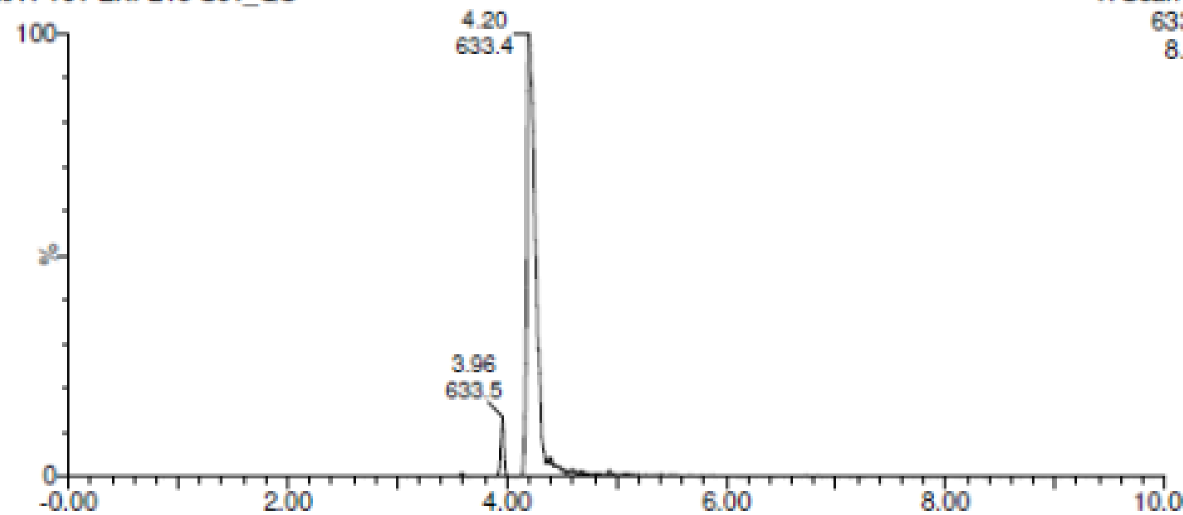

HESSEMA4-101-EXP216-S01_QC Sm (Mn, 2x3)

2: Diode Array
220
Range: 1.474

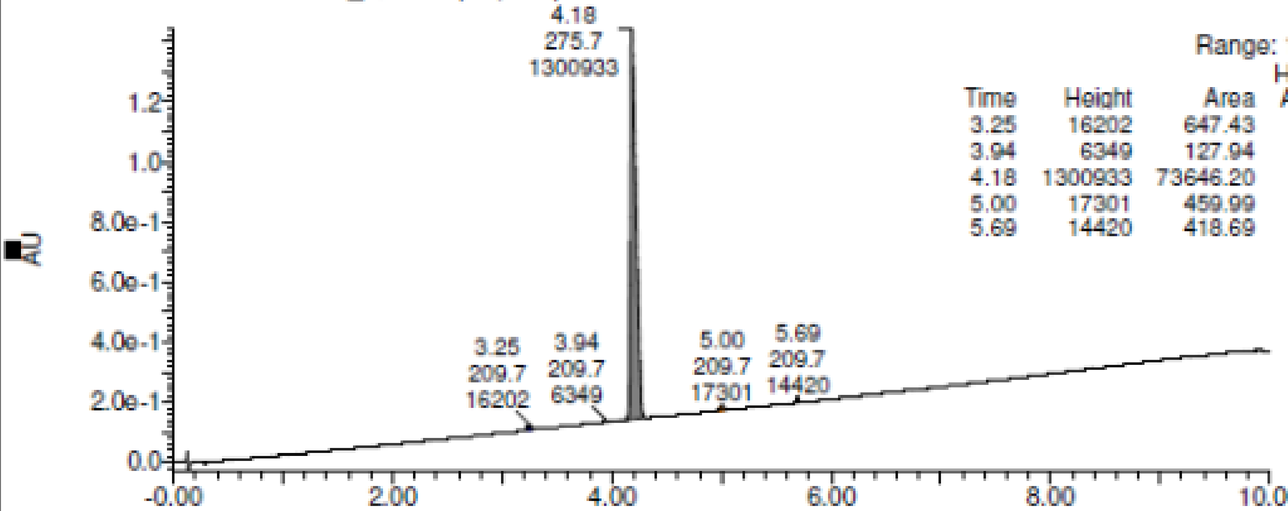

Time	Height	Area	Height Area%
3.25	16202	647.43	0.86
3.94	6349	127.94	0.17
4.18	1300933	73646.20	97.80
5.00	17301	459.99	0.61
5.69	14420	418.69	0.56

HESSEMA4-101-EXP216-S01_QC

1: Scan ES+
TIC
1.42e8

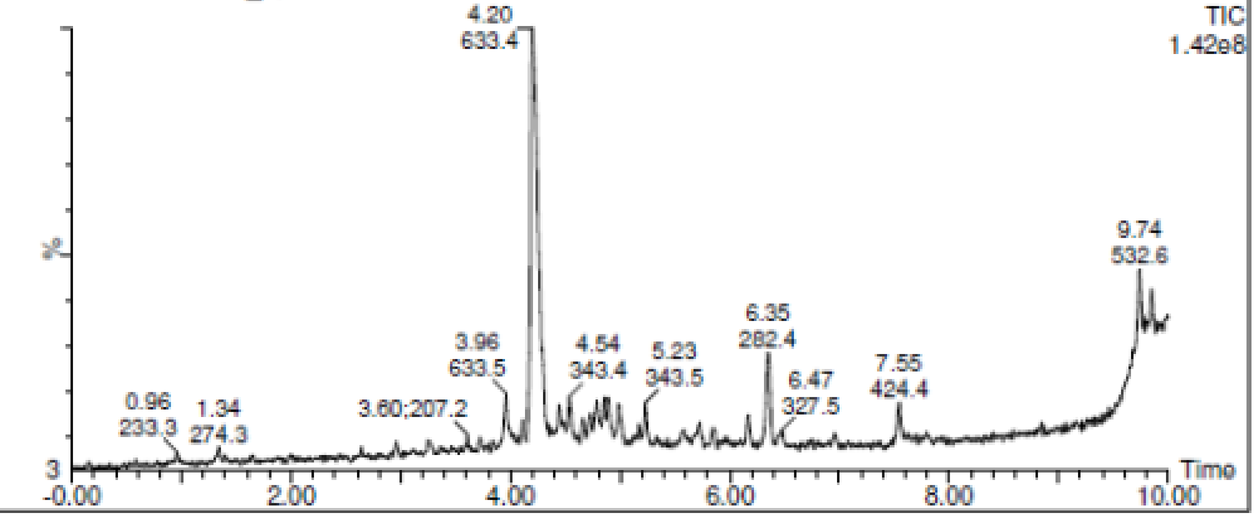

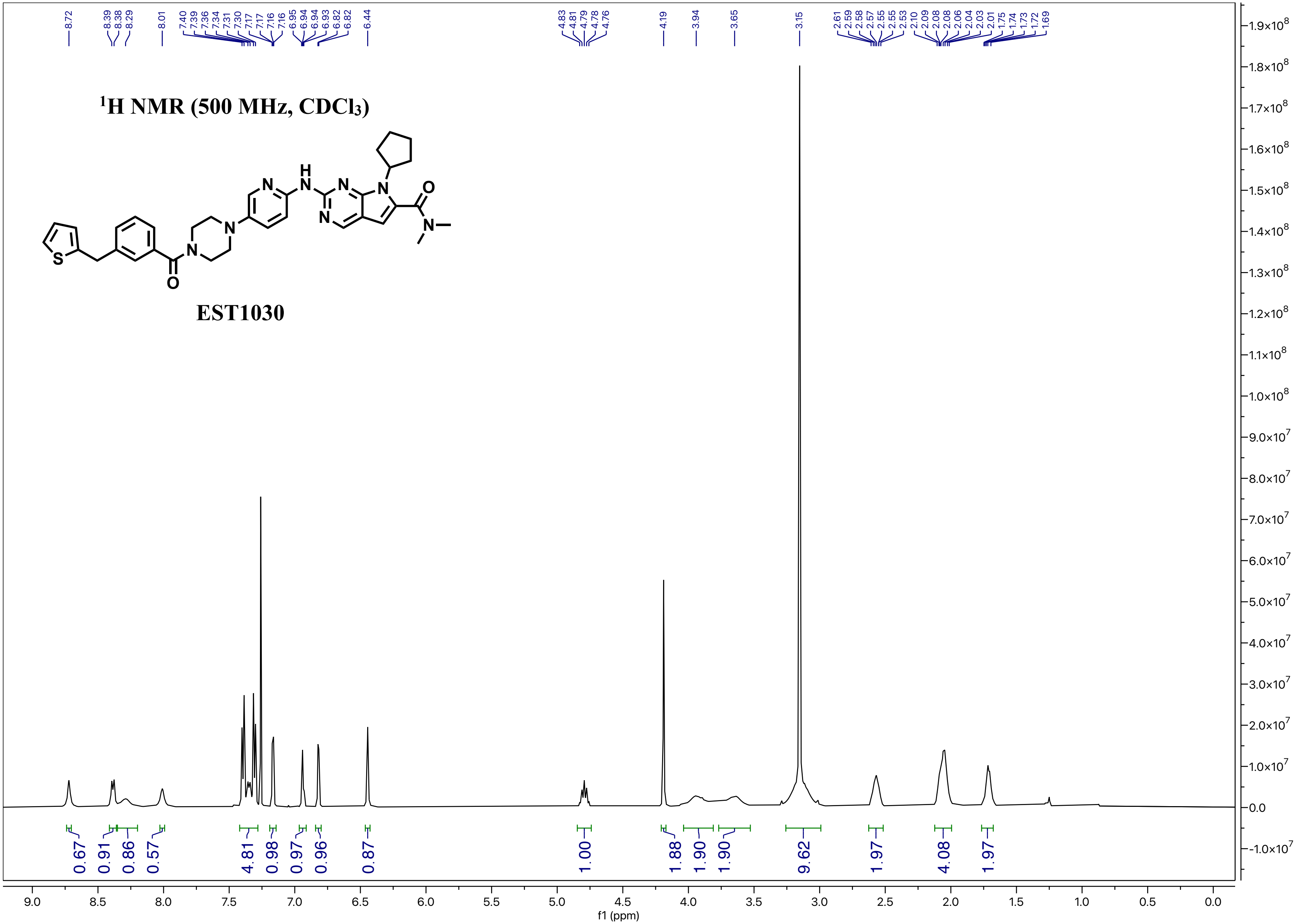

1H NMR (500 MHz, CDCl3)
EST1030
f1 (ppm)

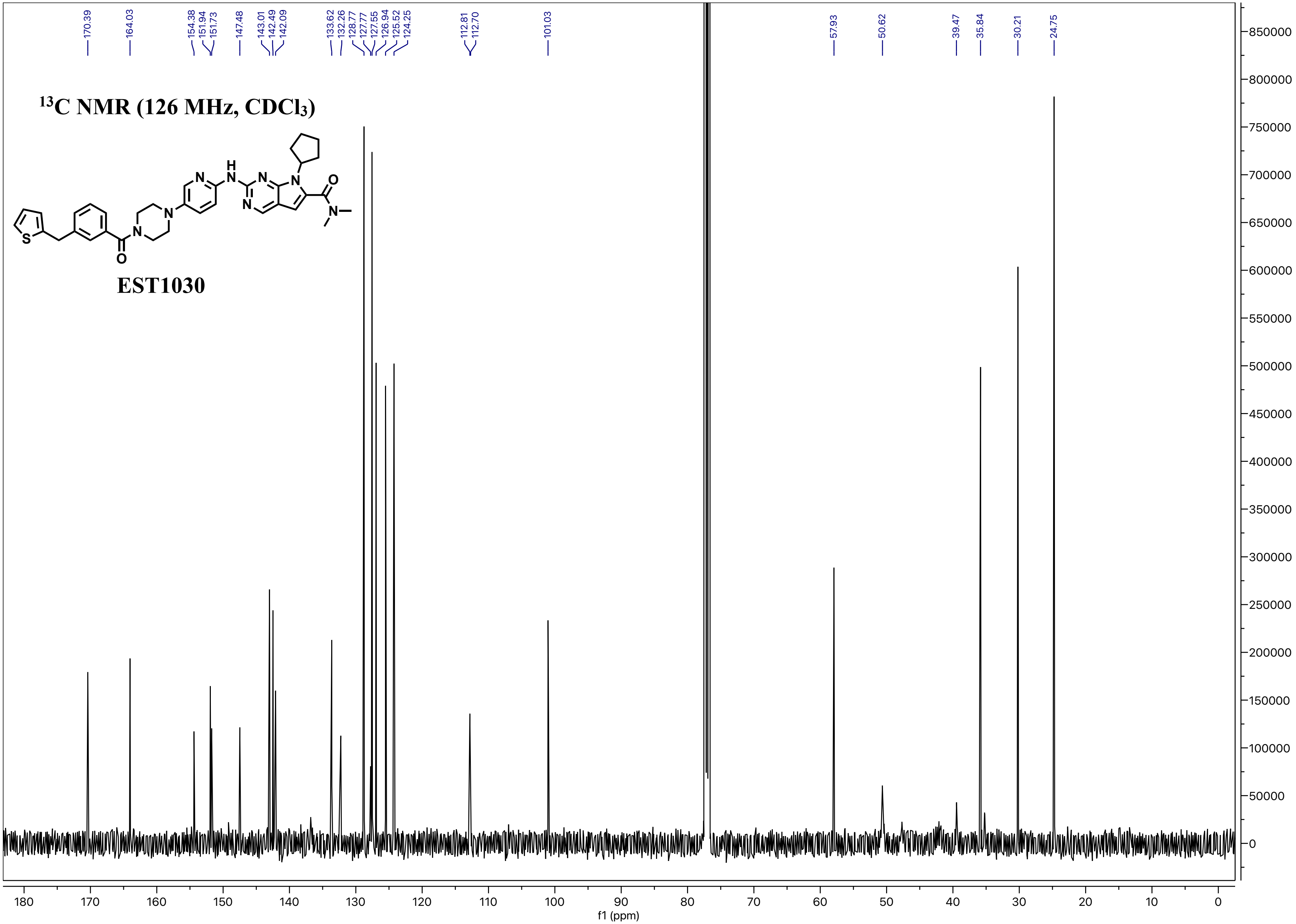

^{13}C NMR (126 MHz, $CDCl_3$)
EST1030
170.39
164.03
154.38
151.94
151.73
147.48
143.01
142.49
142.09
133.62
132.26
128.77
127.77
127.55
126.94
125.52
124.25
112.81
112.70
101.03
57.93
50.62
39.47
35.84
30.21
24.75
f1 (ppm)

HPLC=0.1%TFA in H2O/ACN with 1.2mL/min

EM23001594431 | **08-Feb-2023** | **15:37:45** | **strubhe1-0208A-v11** | **LCMS-ZQ1** | **Kinetex column,** | **ZQ1_10m_Acidic**

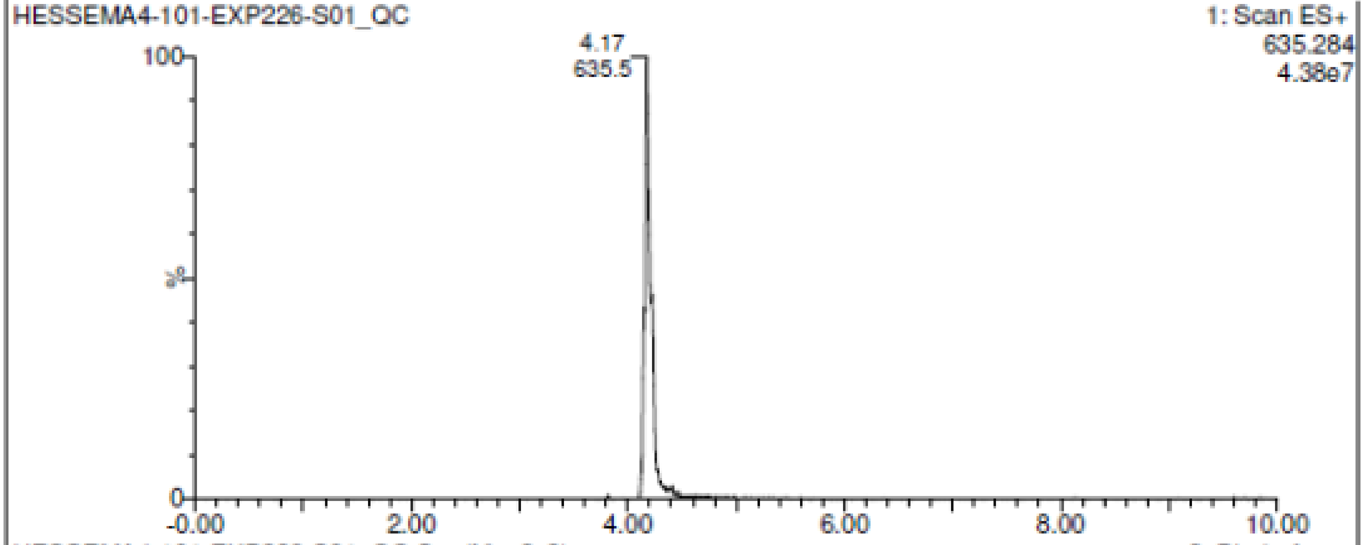

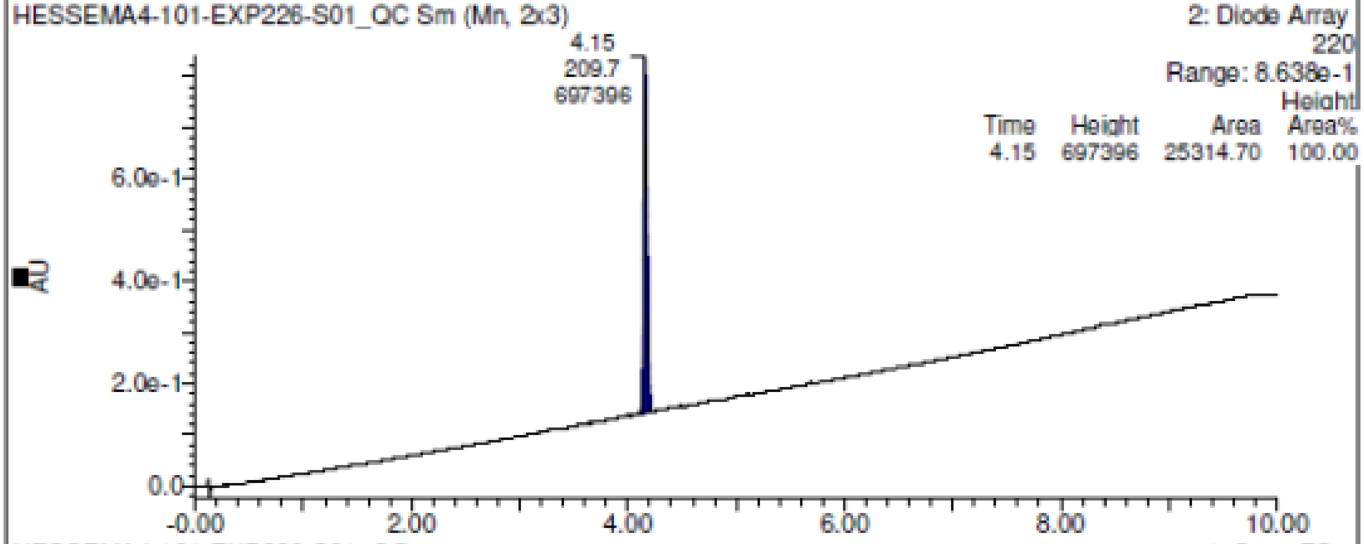

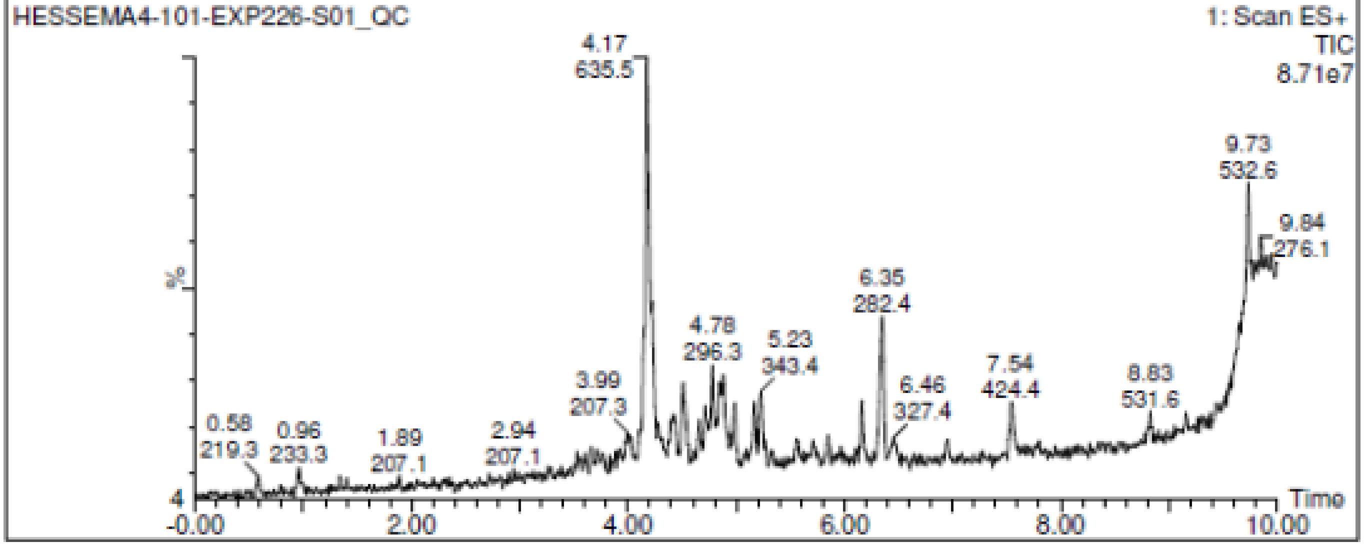

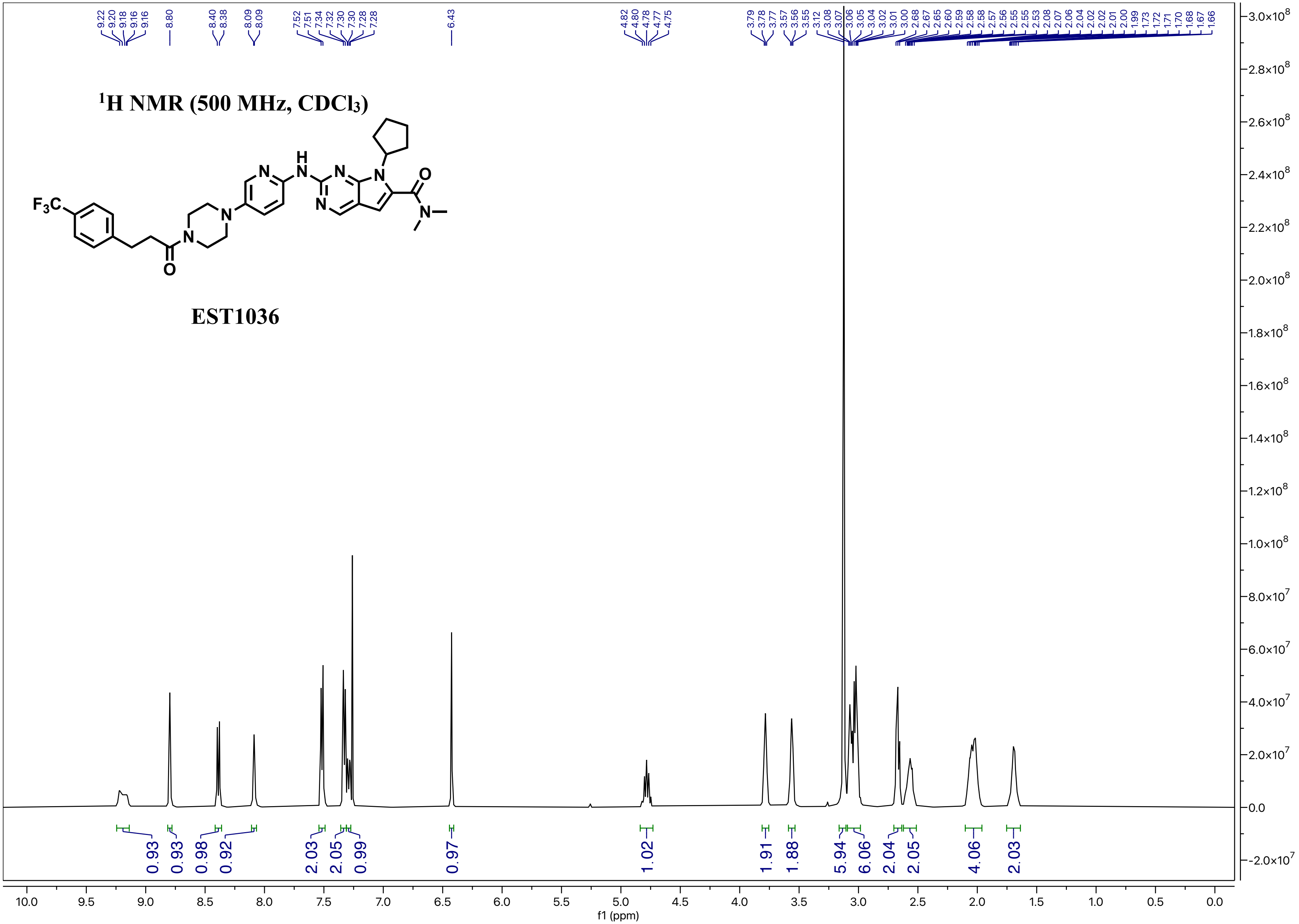

^{1}H NMR (500 MHz, CDCl$_3$)
EST1036
f1 (ppm)

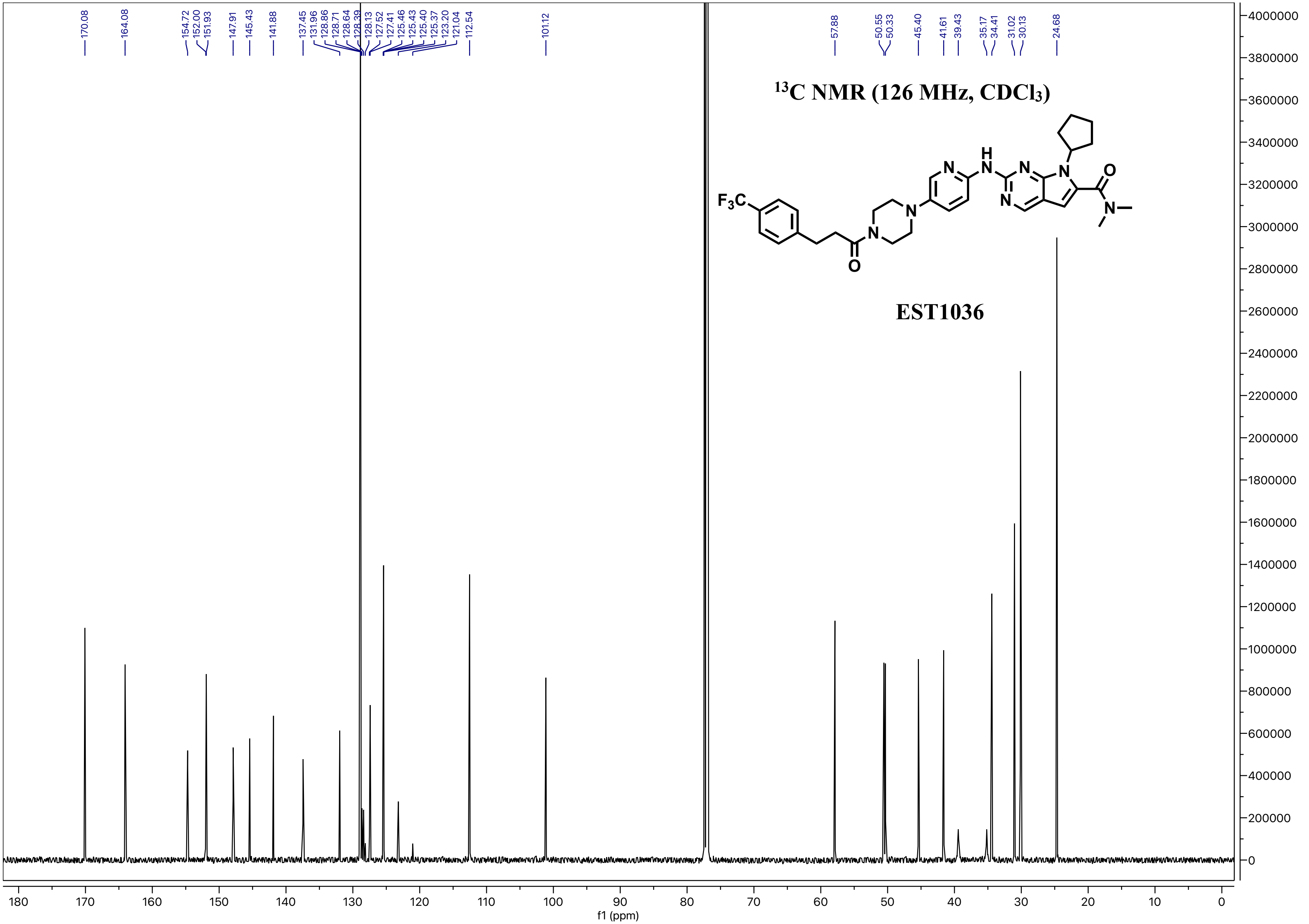

170.08
164.08
154.72
152.00
151.93
147.91
145.43
141.88
137.45
131.96
128.86
128.71
128.64
128.39
128.13
127.52
127.41
125.46
125.43
125.40
125.37
123.20
121.04
112.54
101.12
57.88
50.55
50.33
45.40
41.61
39.43
35.17
34.41
31.02
30.13
24.68
¹³C NMR (126 MHz, CDCl₃)
EST1036
f1 (ppm)

HPLC=0.1%TFA in H2O/ACN with 1.2mL/min

EM23001594432

08-Feb-2023
15:48:47
strubhe1-0208A-v12

LCMS-ZQ1
Kinetex column,
ZQ1_10m_Acidic

HESSEMA4-101-EXP227-S01_QC

1: Scan ES+
635.299
6.91e7

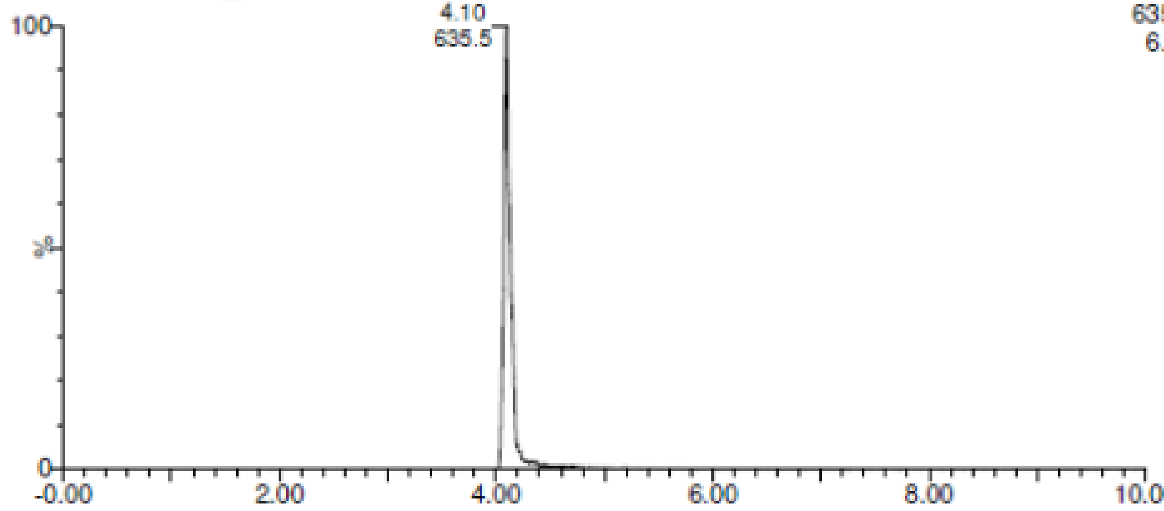

HESSEMA4-101-EXP227-S01_QC Sm (Mn, 2x3)

2: Diode Array
220
Range: 9.194e-1

Time	Height	Area	Height Area%
4.07	756628	31192.64	100.00

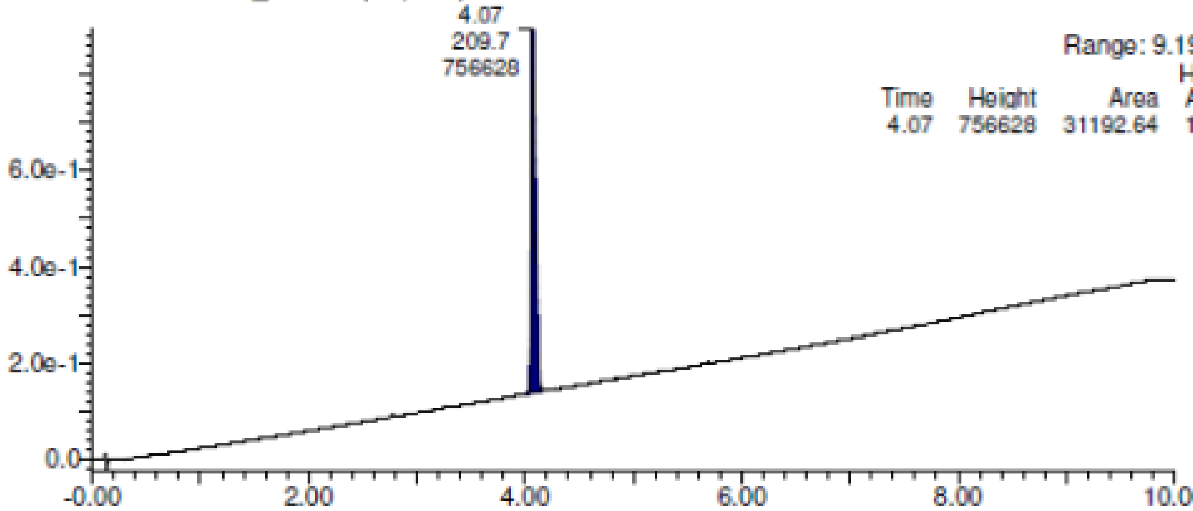

HESSEMA4-101-EXP227-S01_QC

1: Scan ES+
TIC
1.08e8

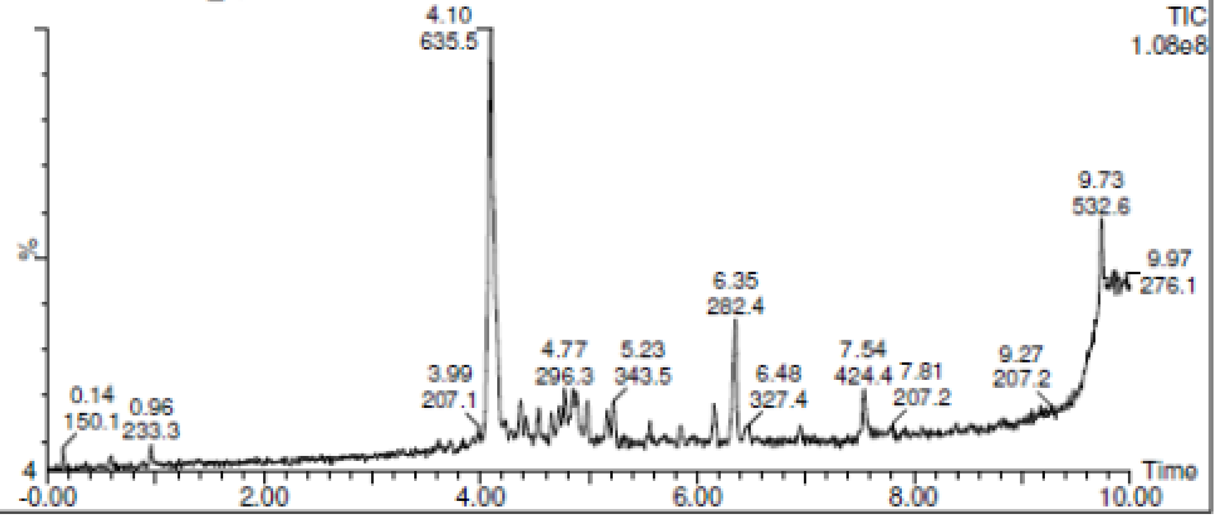

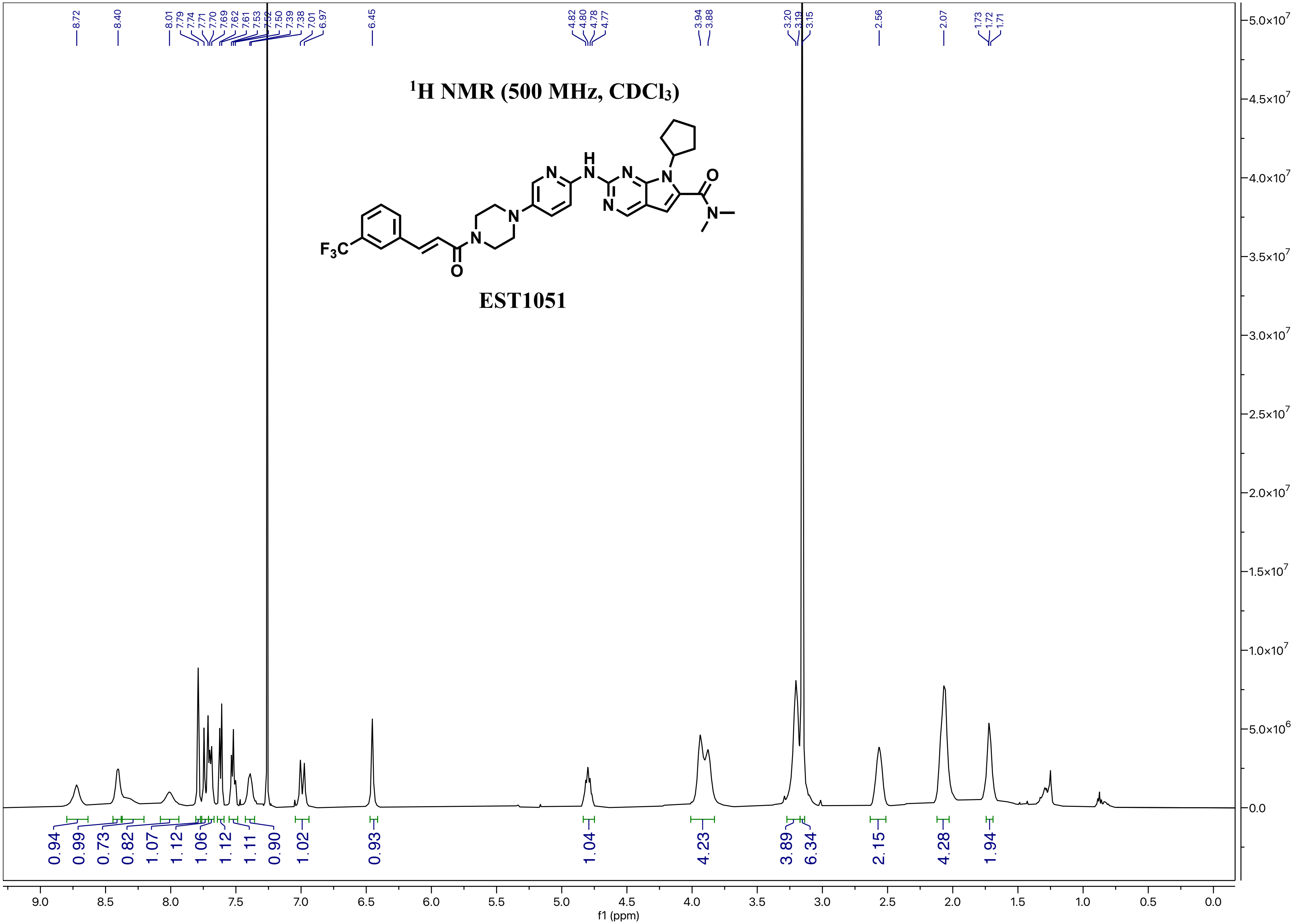

^{1}H NMR (500 MHz, CDCl$_3$)
EST1051
f1 (ppm)

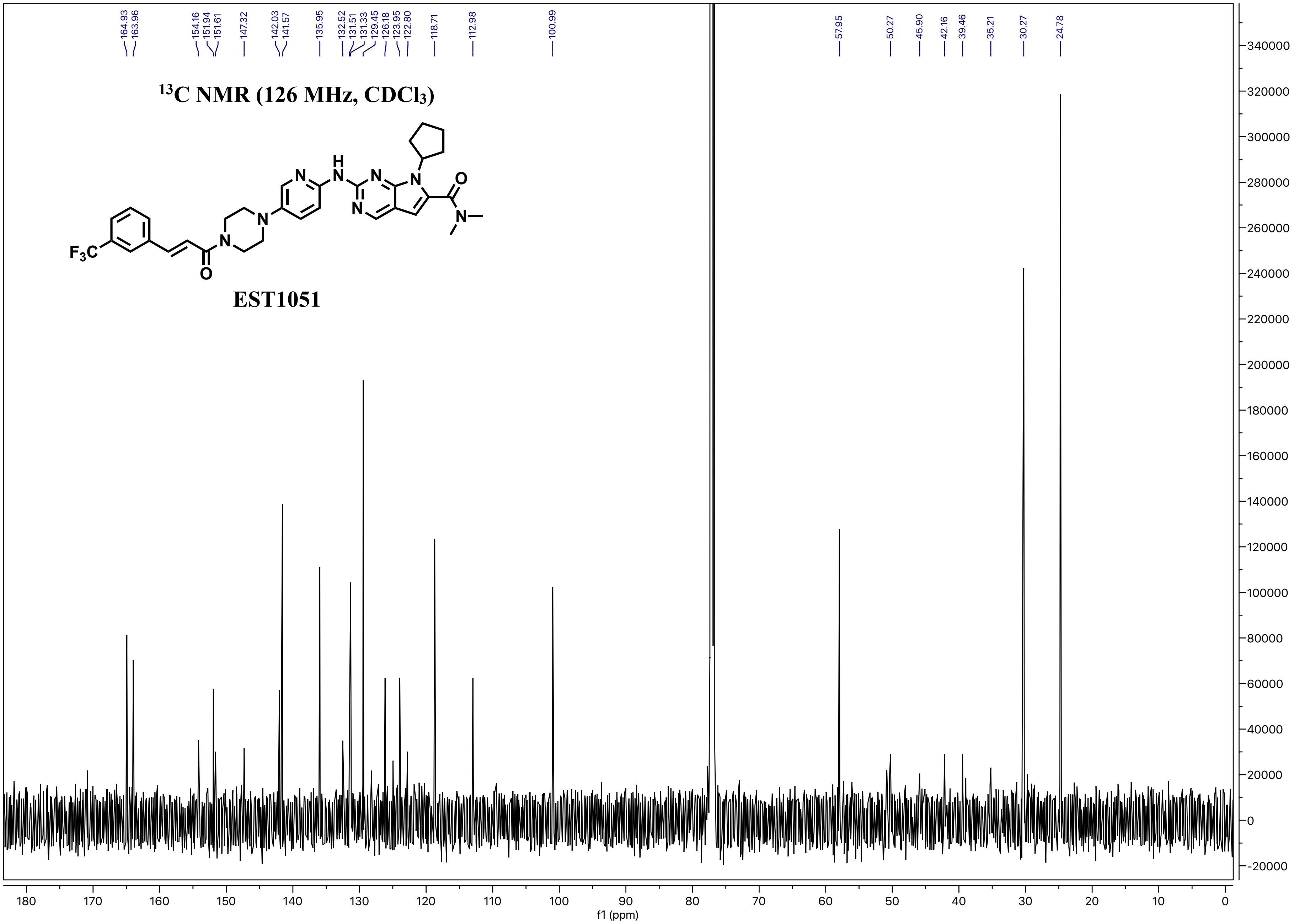
164.93
163.96
154.16
151.94
151.61
147.32
142.03
141.57
135.95
132.52
131.51
131.33
129.45
126.18
123.95
122.80
118.71
112.98
100.99
57.95
50.27
45.90
42.16
39.46
35.21
30.27
24.78
13C NMR (126 MHz, CDCl3)
F3C
EST1051
f1 (ppm)

HPLC=0.1%TFA in H2O/ACN with 1.2mL/min

**EM23001594434**	**08-Feb-2023**<br>**15:59:50**<br>**strubhe1-0208A-v13**	**LCMS-ZQ1**<br>**Kinetex column,**<br>**ZQ1_10m_Acidic**

HESSEMA4-101-EXP228-S01_QC

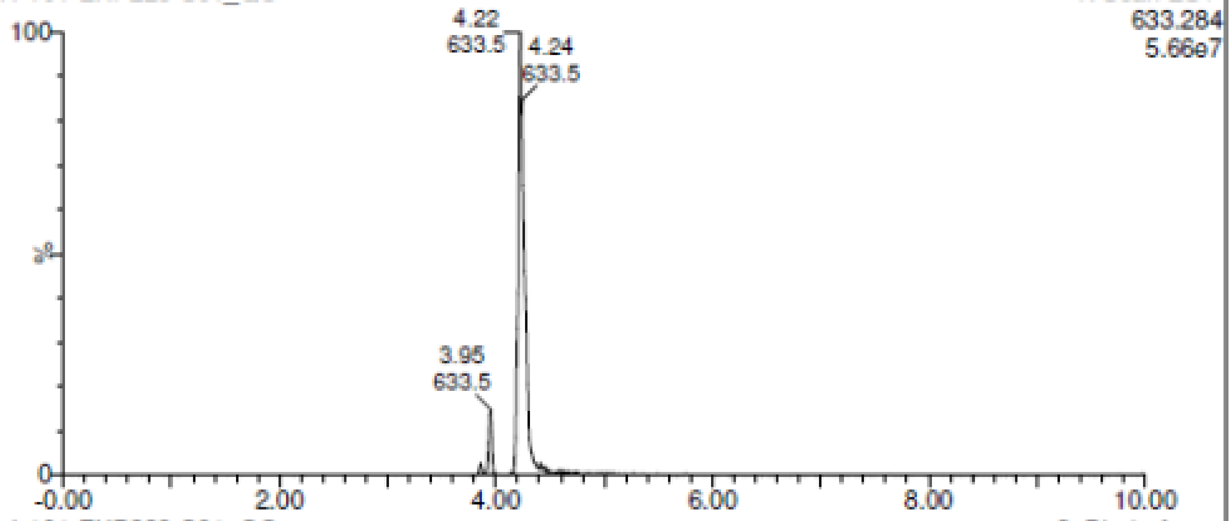

HESSEMA4-101-EXP228-S01_QC

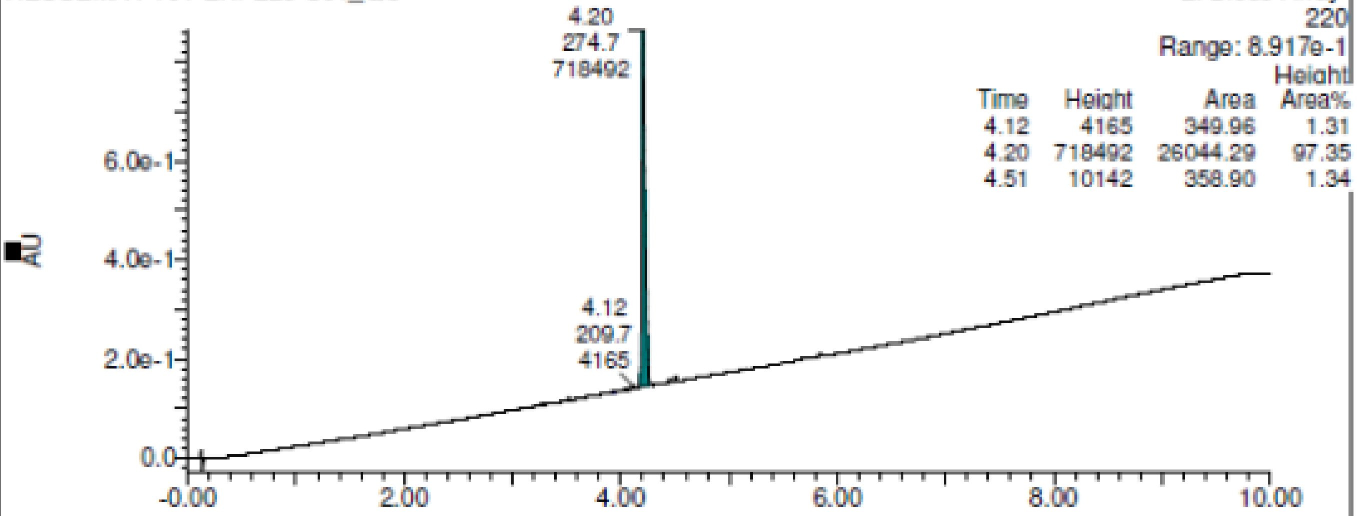

Time	Height	Area	Height Area%
4.12	4165	349.96	1.31
4.20	718492	26044.29	97.35
4.51	10142	358.90	1.34

HESSEMA4-101-EXP228-S01_QC

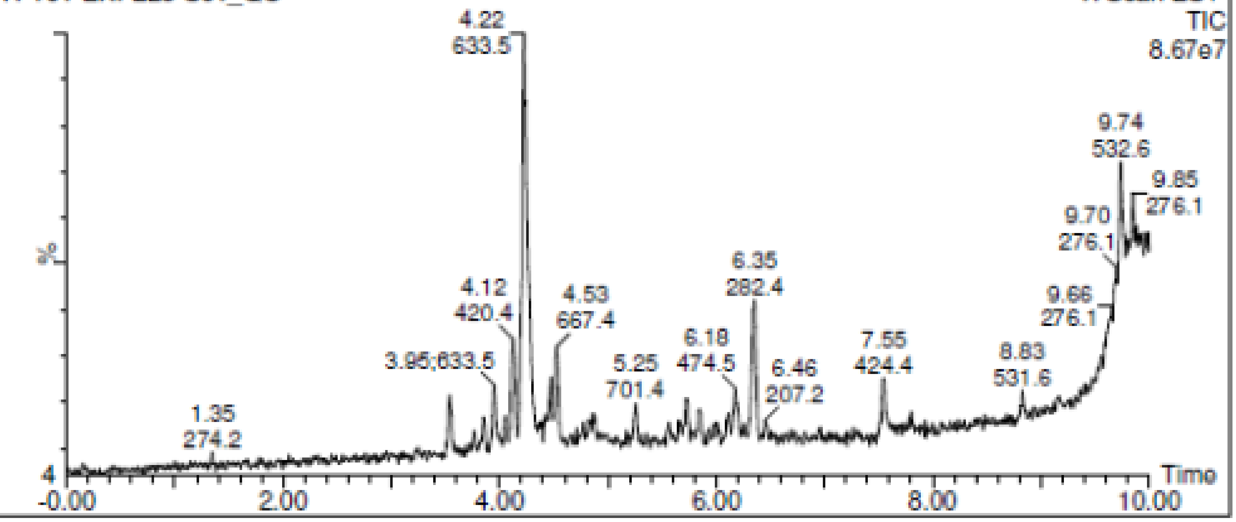

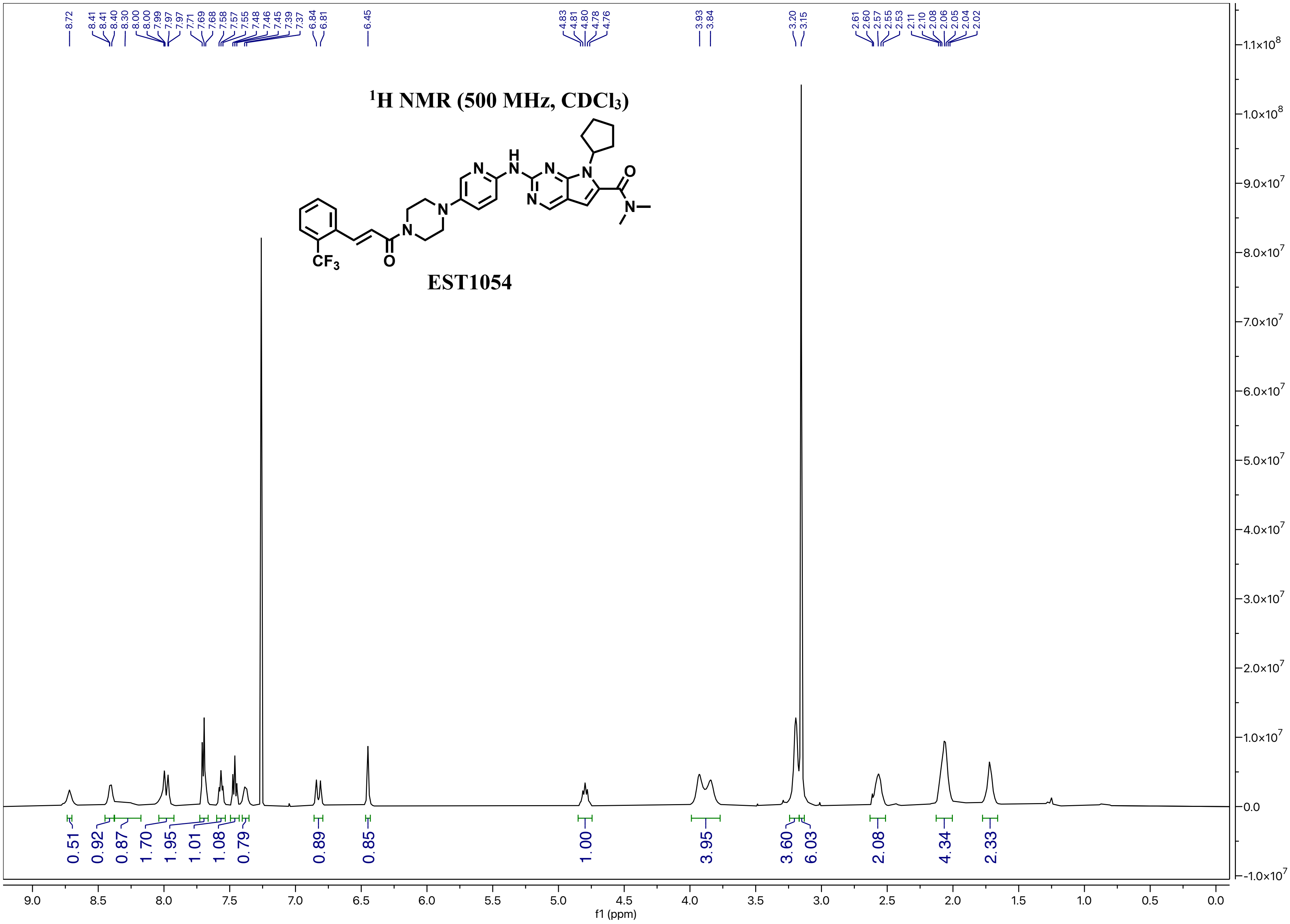
^{1}H NMR (500 MHz, $CDCl_3$)
EST1054
f1 (ppm)

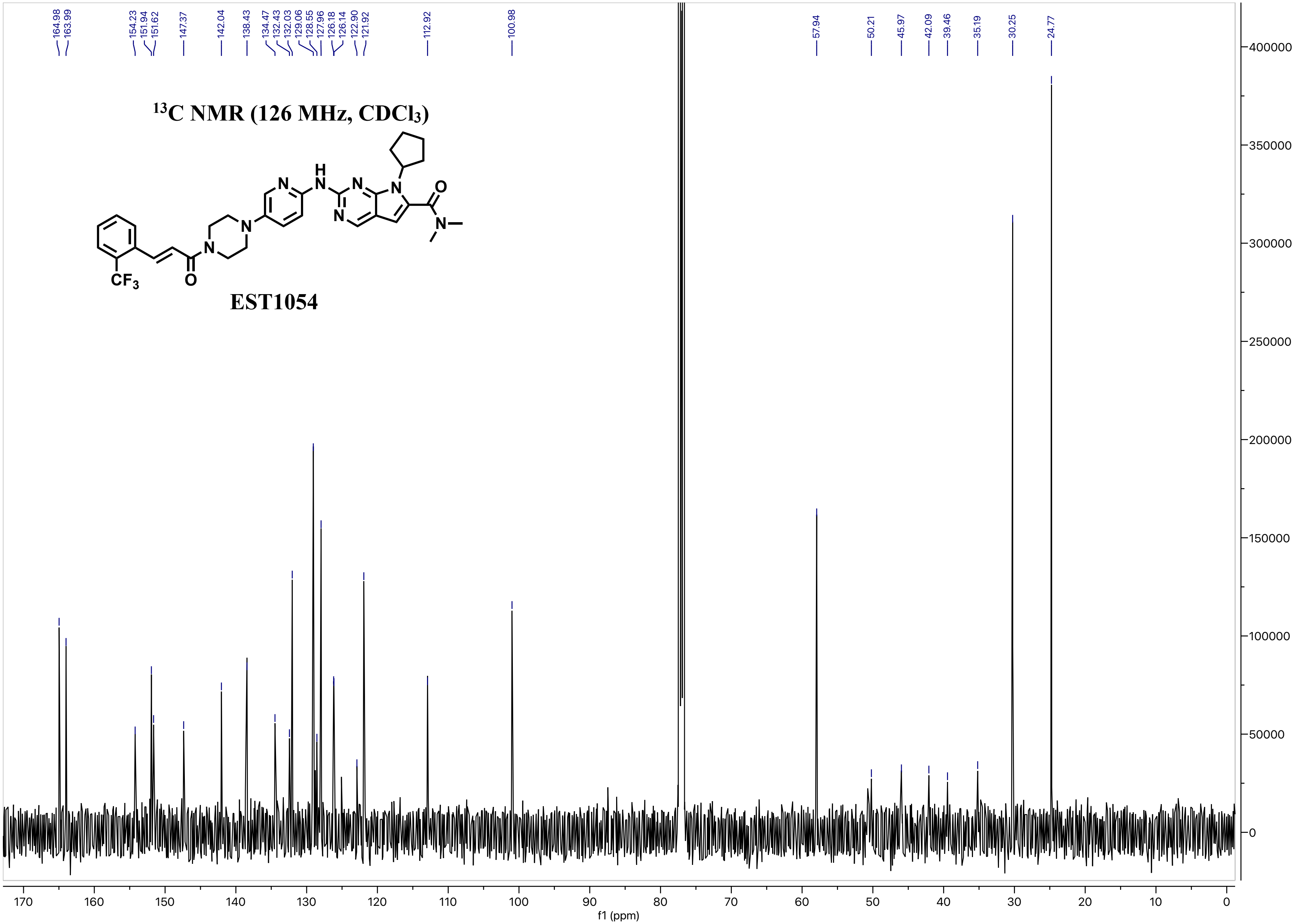
164.98
163.99
154.23
151.94
151.62
147.37
142.04
138.43
134.47
132.43
132.03
129.06
128.55
127.96
126.18
126.14
122.90
121.92
112.92
100.98
57.94
50.21
45.97
42.09
39.46
35.19
30.25
24.77
13C NMR (126 MHz, CDCl3)
EST1054
f1 (ppm)

HPLC=0.1%TFA in H2O/ACN with 1.2mL/min

EM23001594435 | 08-Feb-2023 16:10:53 | strubhe1-0208A-v14 | LCMS-ZQ1 Kinetex column, ZQ1_10m_Acidic

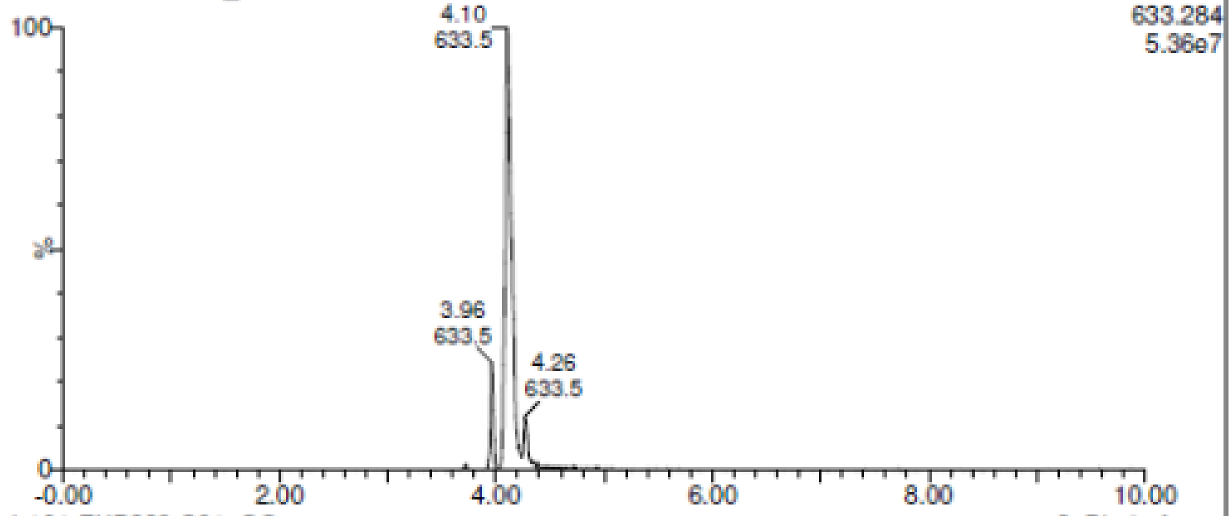

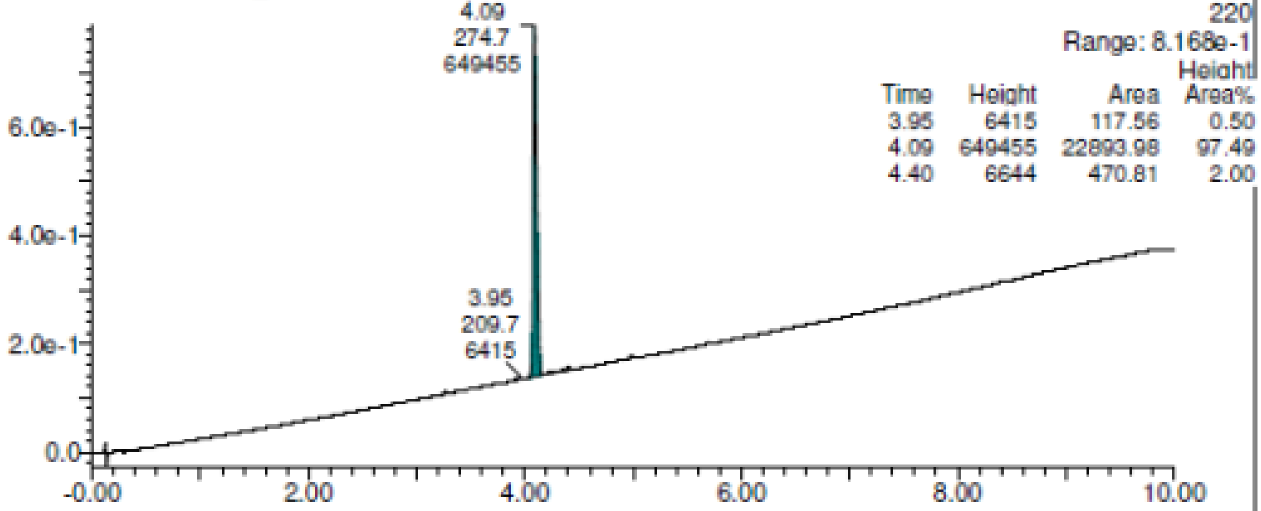

Time	Height	Area	Height Area%
3.95	6415	117.56	0.50
4.09	649455	22893.98	97.49
4.40	6644	470.81	2.00

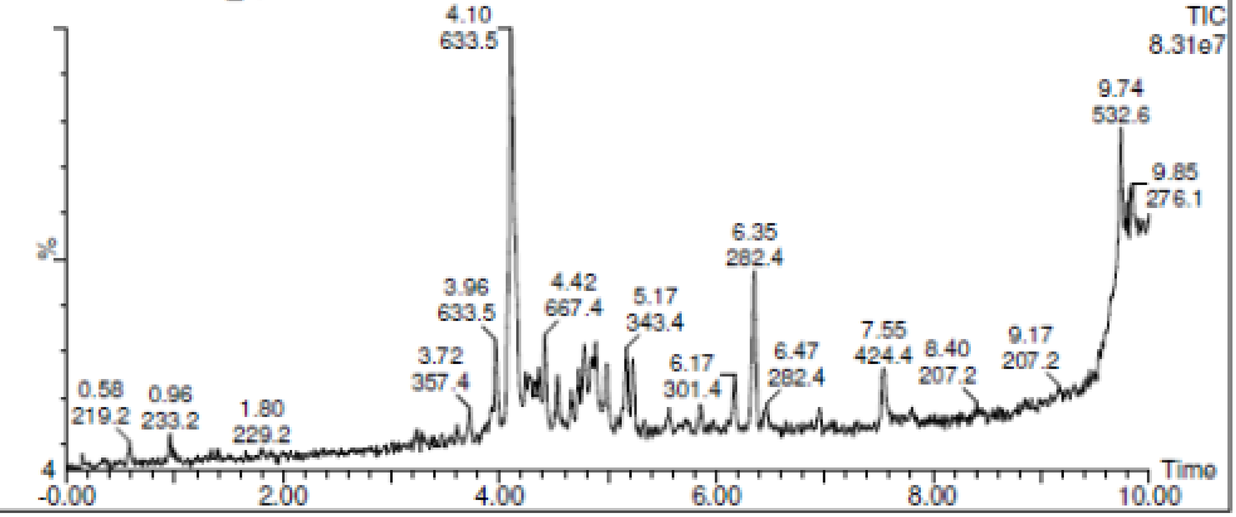

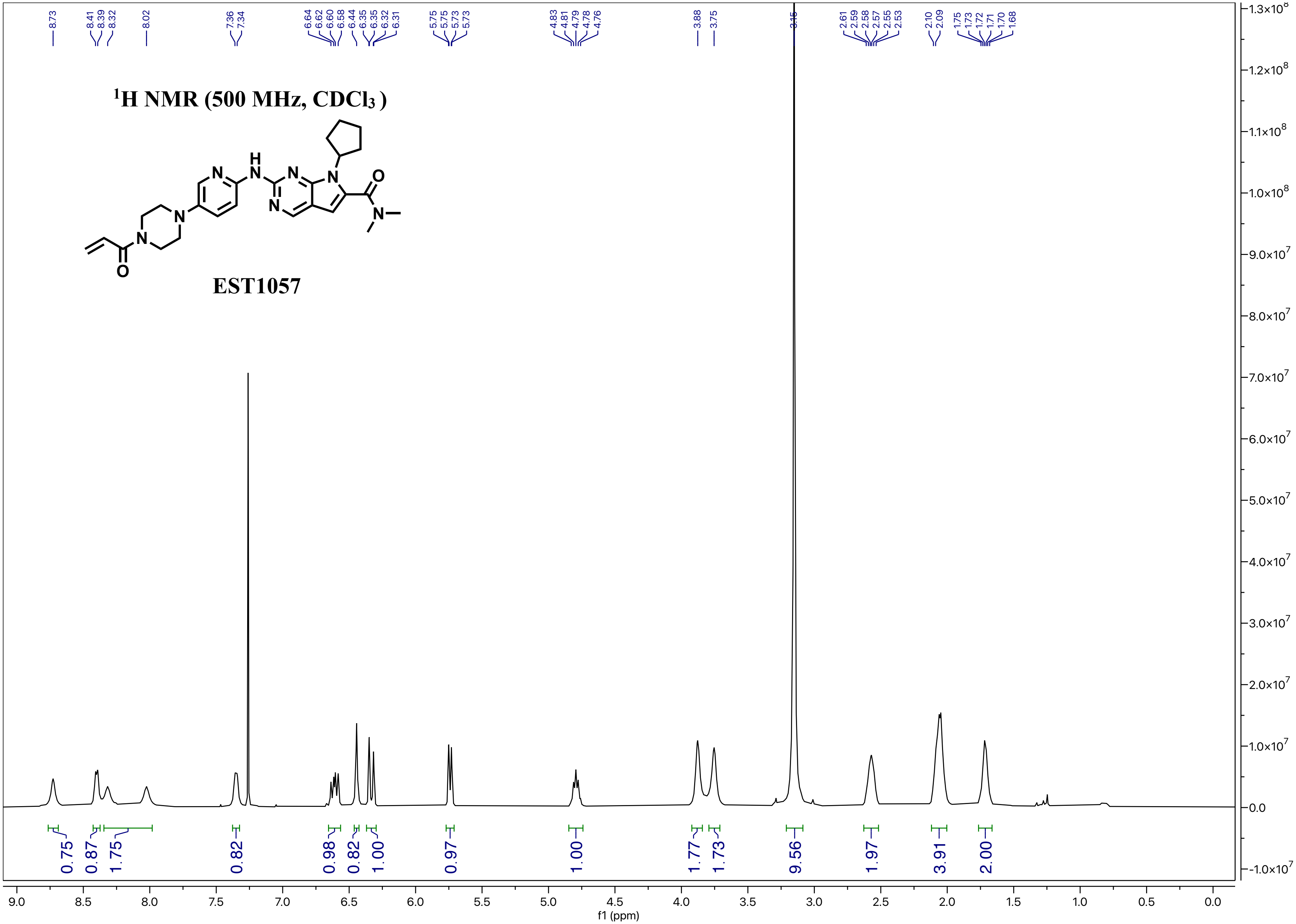
1H NMR (500 MHz, CDCl3)
EST1057
8.73
8.41 8.39 8.32
8.02
7.36 7.34
6.64 6.62 6.60 6.58 6.44 6.35 6.35 6.32 6.31
5.75 5.75 5.73 5.73
4.83 4.81 4.79 4.78 4.76
3.88 3.75
3.15
2.61 2.59 2.58 2.57 2.55 2.53
2.10 2.09
1.75 1.73 1.72 1.71 1.70 1.68
0.75 0.87 1.75 0.82 0.98 0.82 1.00 0.97 1.00 1.77 1.73 9.56 1.97 3.91 2.00
f1 (ppm)

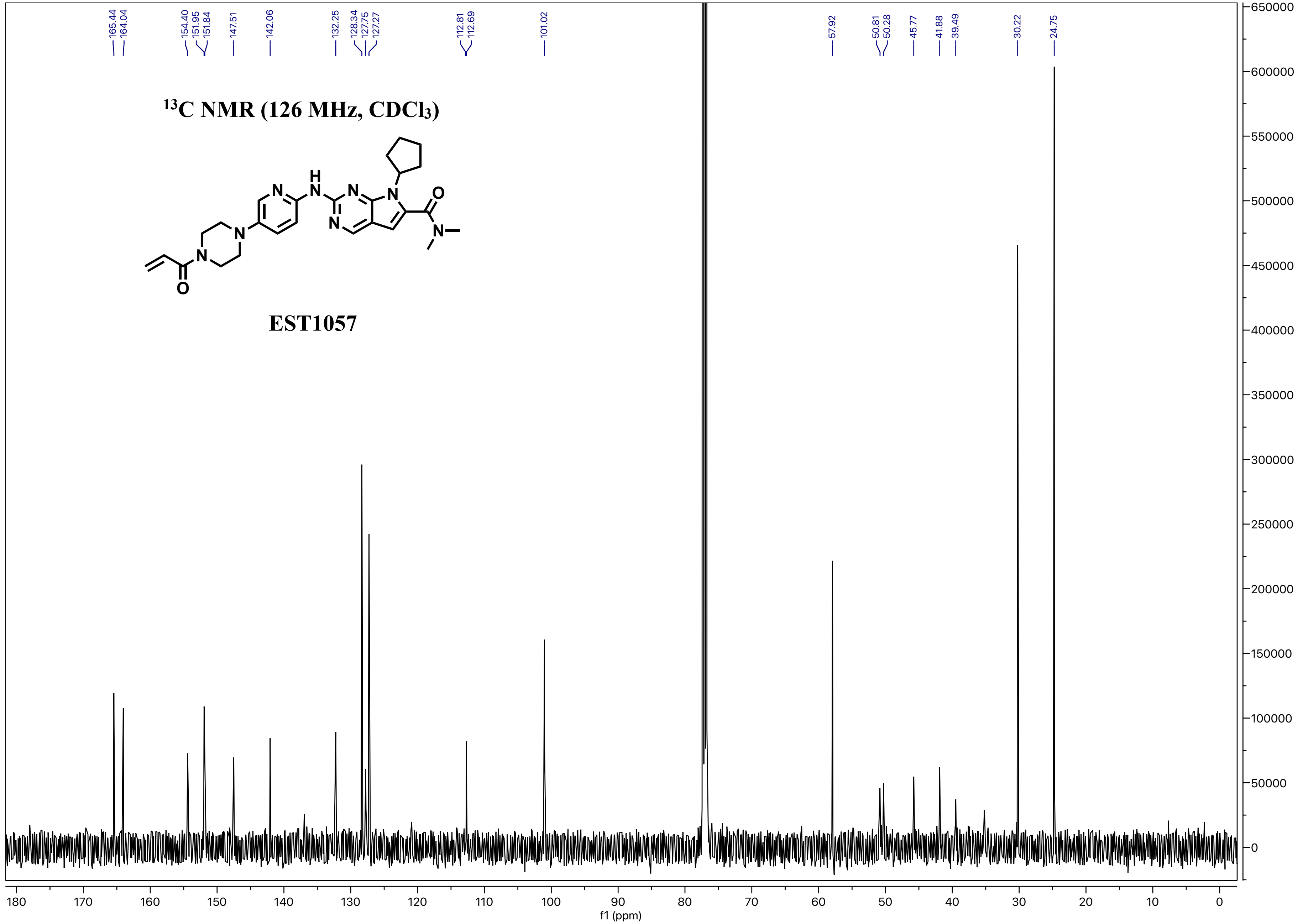

165.44
164.04
154.40
151.95
151.84
147.51
142.06
132.25
128.34
127.75
127.27
112.81
112.69
101.02
57.92
50.81
50.28
45.77
41.88
39.49
30.22
24.75
^{13}C NMR (126 MHz, $CDCl_3$)
EST1057
f1 (ppm)

HPLC=0.1%TFA in H2O/ACN with 1.2mL/min

**EM23001594436**	**08-Feb-2023**	**LCMS-ZQ1**
	**16:21:57**	**Kinetex column,**
	**strubhe1-0208A-v15**	**ZQ1_10m_Acidic**

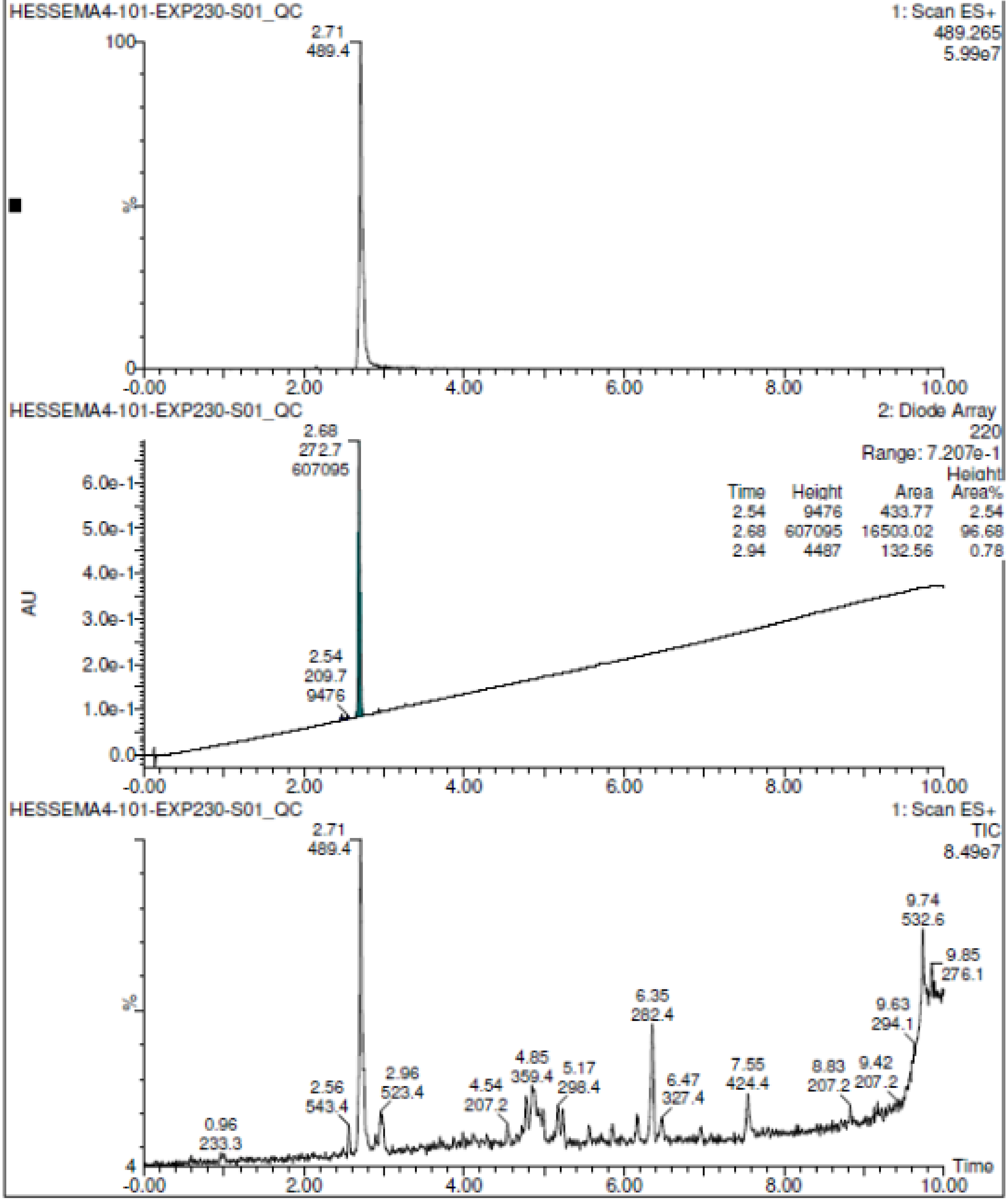

Time	Height	Area	Height Area%
2.54	9476	433.77	2.54
2.68	607095	16503.02	96.68
2.94	4487	132.56	0.78

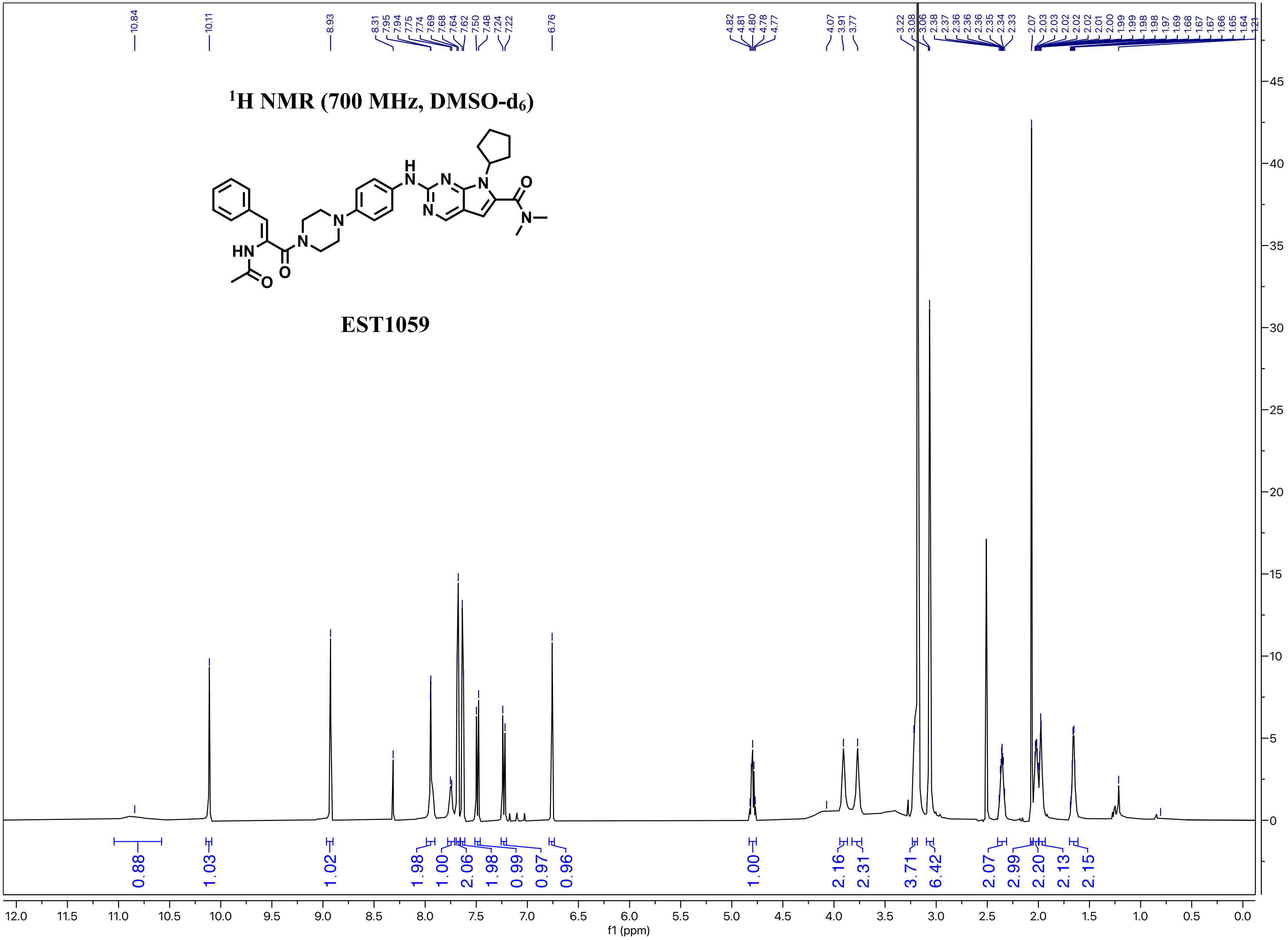

^{1}H NMR (700 MHz, DMSO-d$_6$)
EST1059
f1 (ppm)

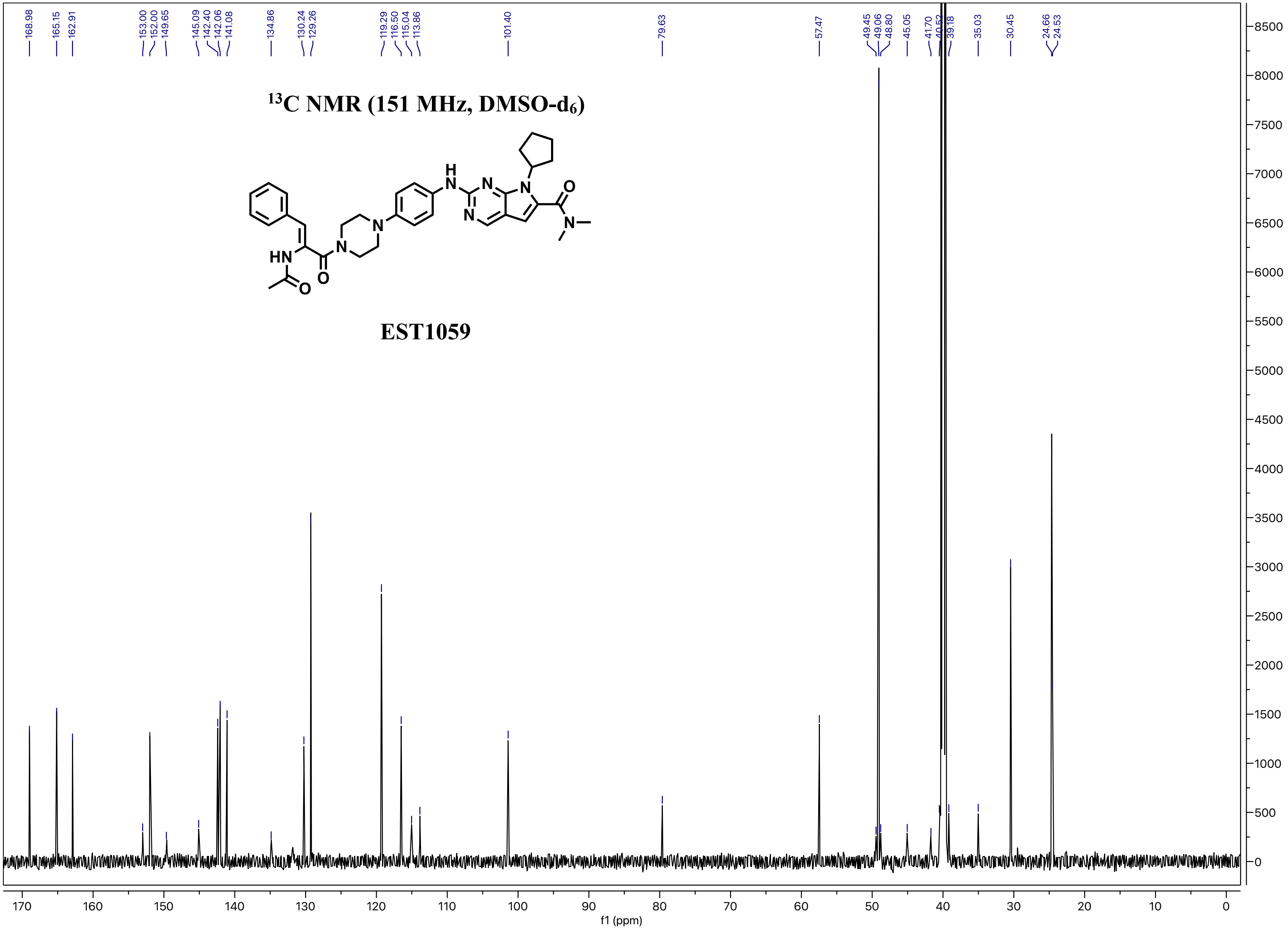
168.98
165.15
162.91
153.00
152.00
149.65
145.09
142.40
142.06
141.08
134.86
130.24
129.26
119.29
116.50
115.04
113.86
101.40
79.63
57.47
49.45
49.06
48.80
45.05
41.70
39.18
35.03
30.45
24.66
24.53
13C NMR (151 MHz, DMSO-d6)
EST1059
f1 (ppm)

HPLC=0.1%TFA in H2O/ACN with 1.2mL/min

EM23001594437

08-Feb-2023
16:33:02
strubhe1-0208A-v16

LCMS-ZQ1
Kinetex column,
ZQ1_10m_Acidic

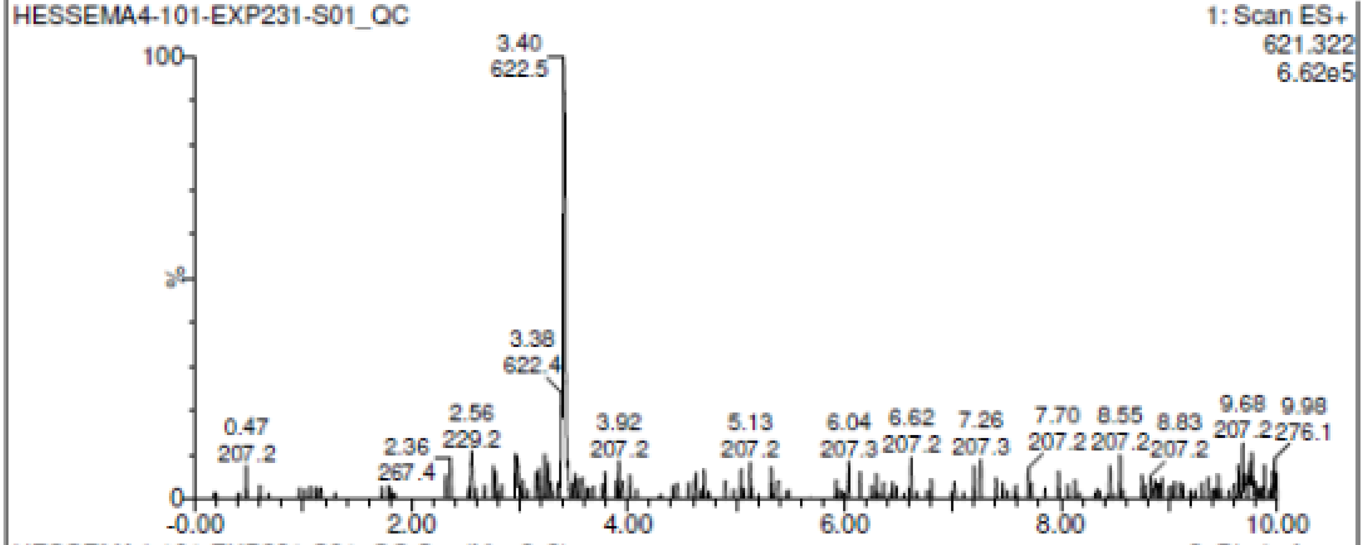

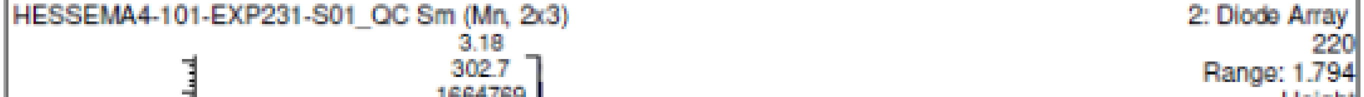

Time	Height	Area	Height Area%
3.18	1664769	67880.83	100.00

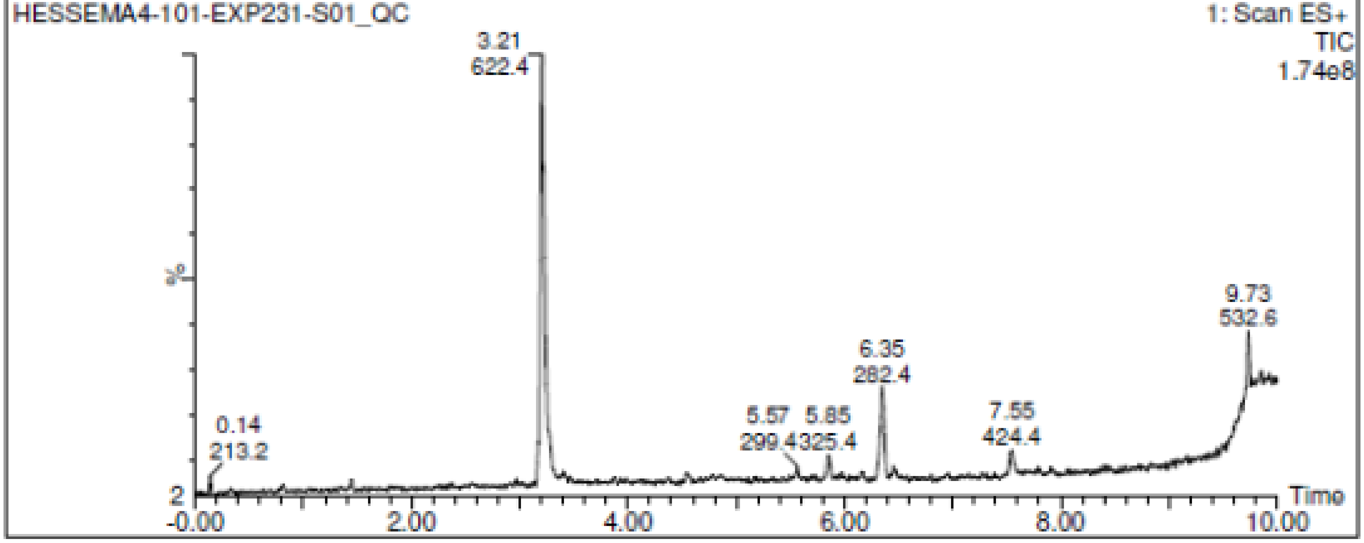

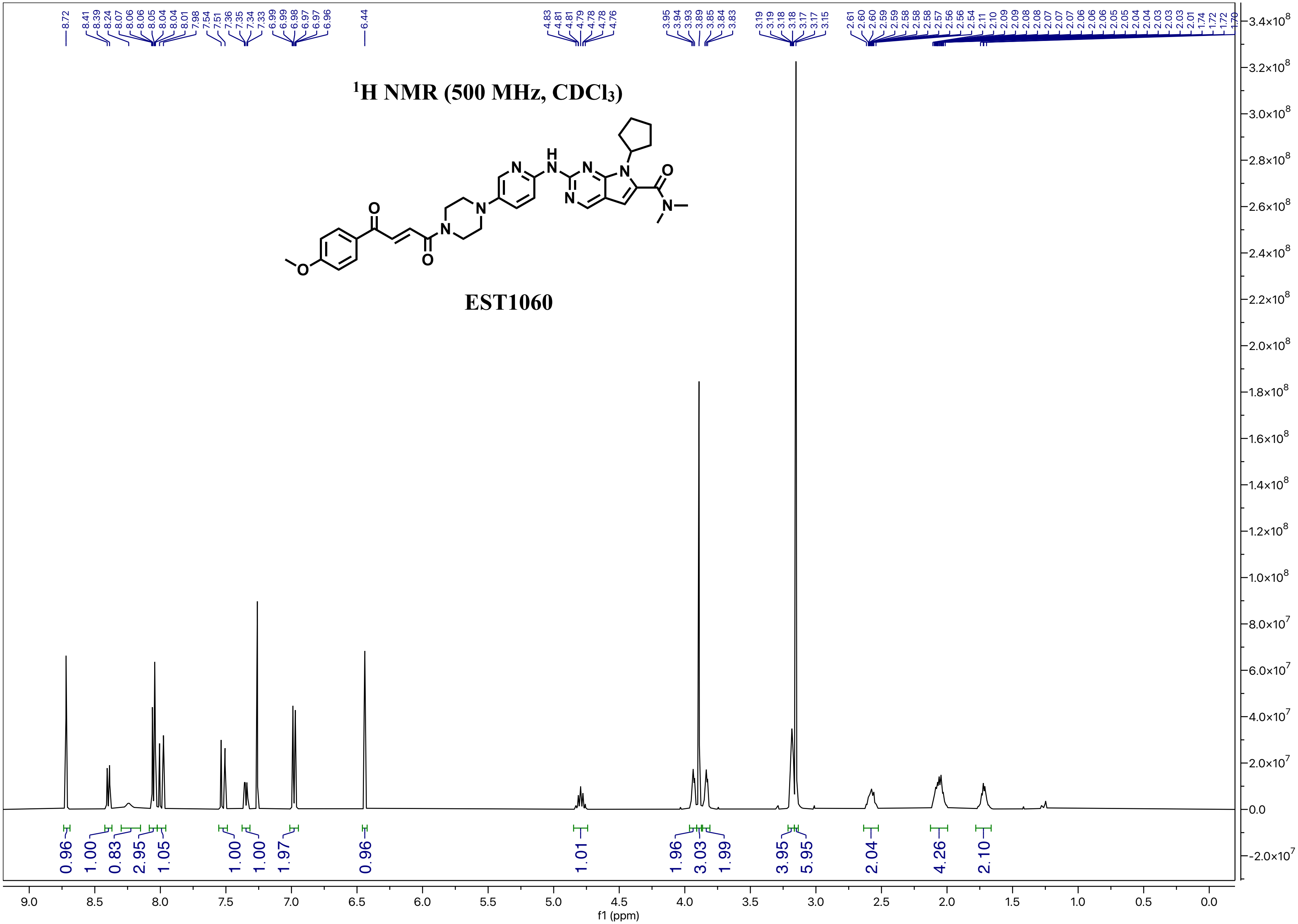

1H NMR (500 MHz, CDCl3)
EST1060
f1 (ppm)

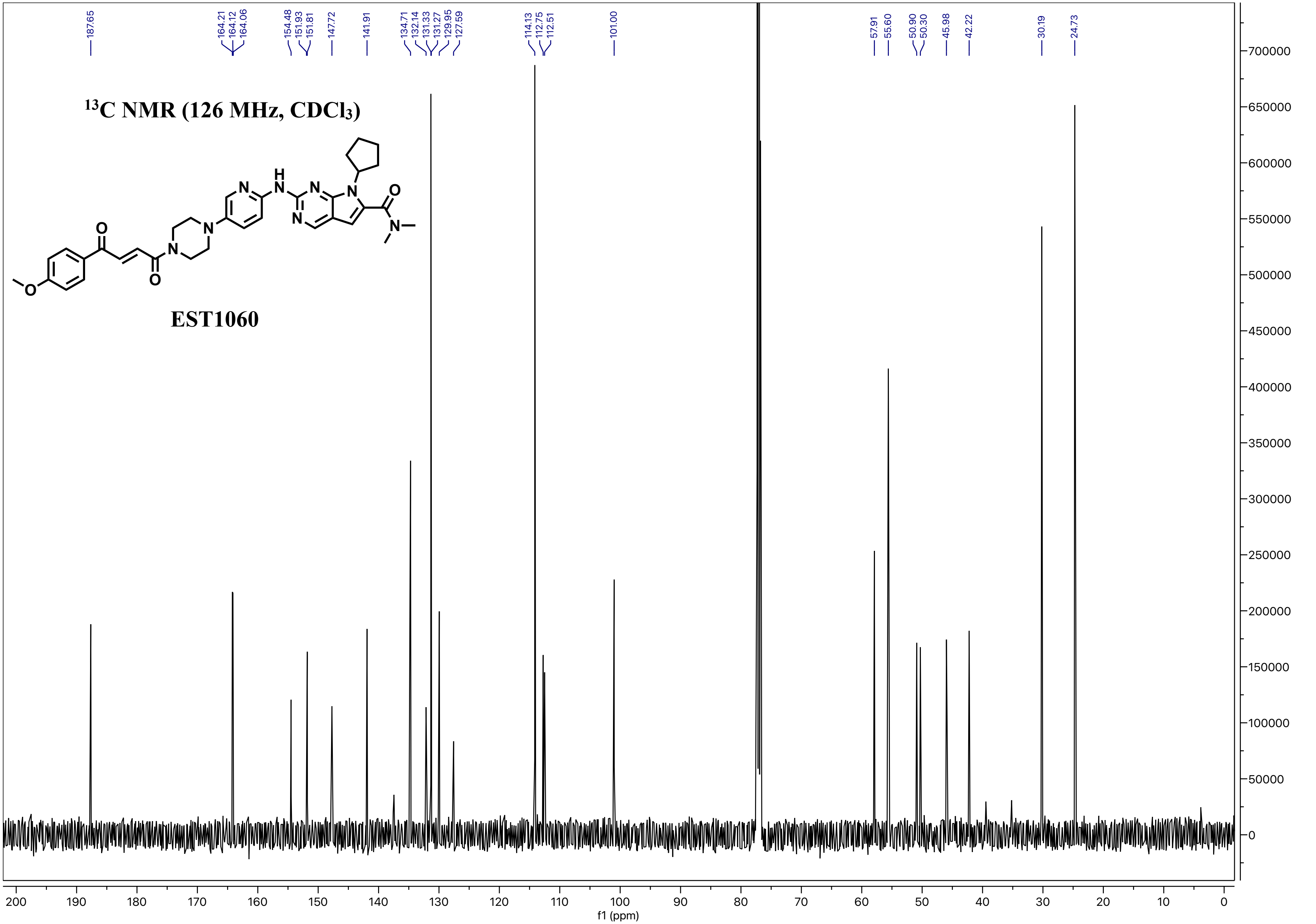

13C NMR (126 MHz, CDCl3)
EST1060
187.65
164.21
164.12
164.06
154.48
151.93
151.81
147.72
141.91
134.71
132.14
131.33
131.27
129.95
127.59
114.13
112.75
112.51
101.00
57.91
55.60
50.90
50.30
45.98
42.22
30.19
24.73
f1 (ppm)

HPLC=0.1%TFA in H2O/ACN with 1.2mL/min

EM23001594421 | 08-Feb-2023 13:58:19 strubhe1-0208A-v02 | LCMS-ZQ1 Kinetex column, ZQ1_10m_Acidic

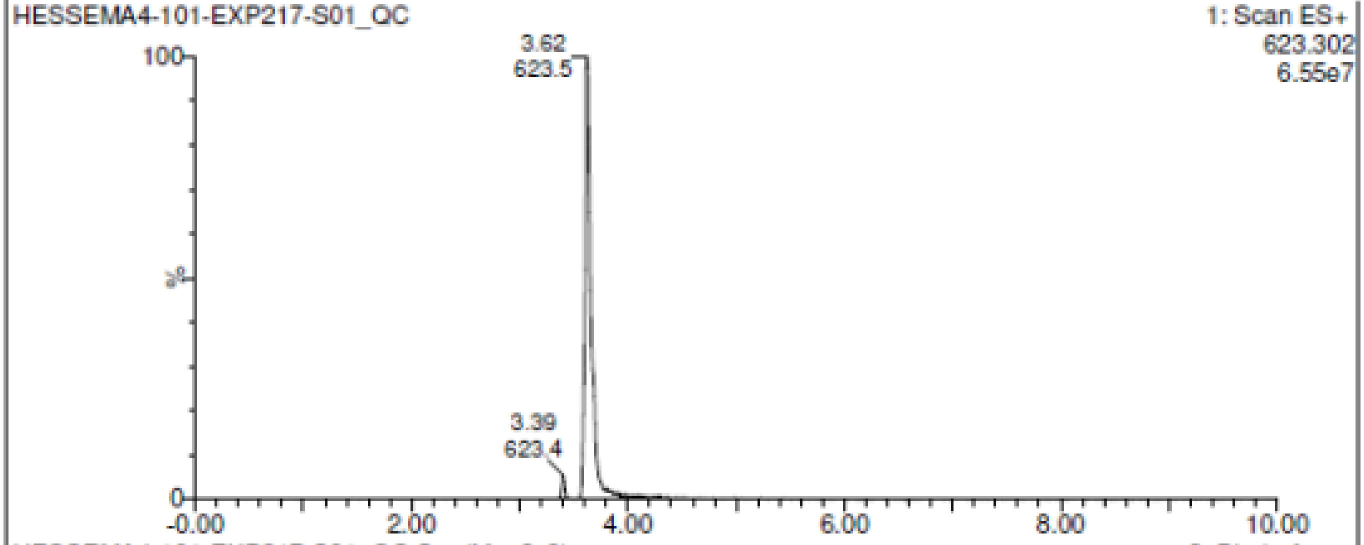

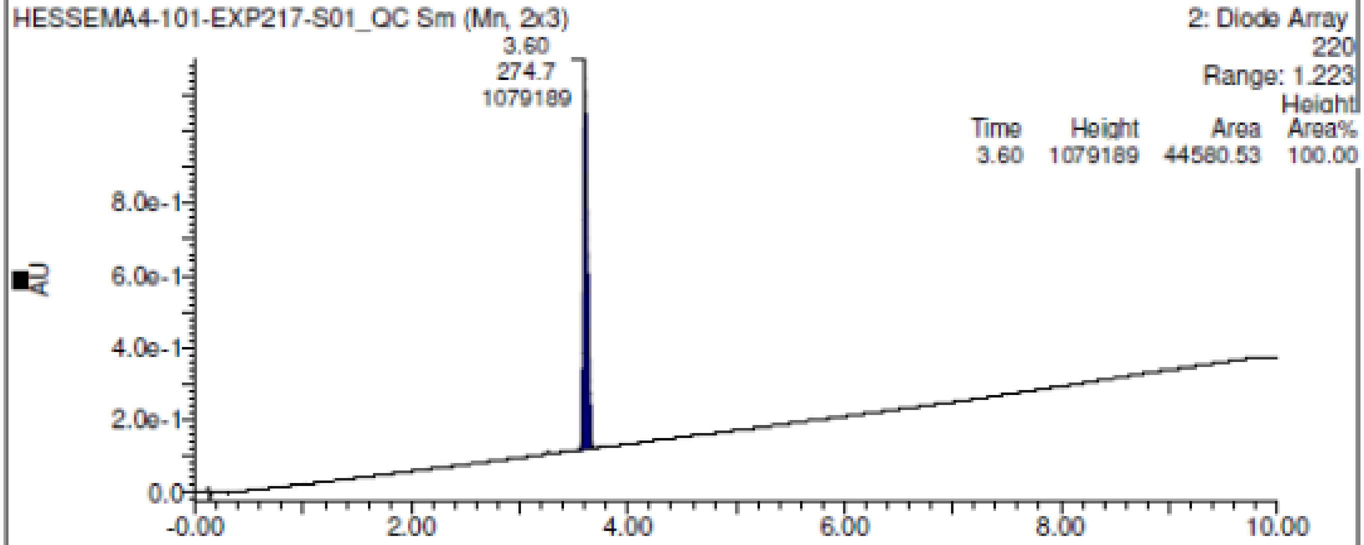

Time	Height	Area	Height Area%
3.60	1079189	44580.53	100.00

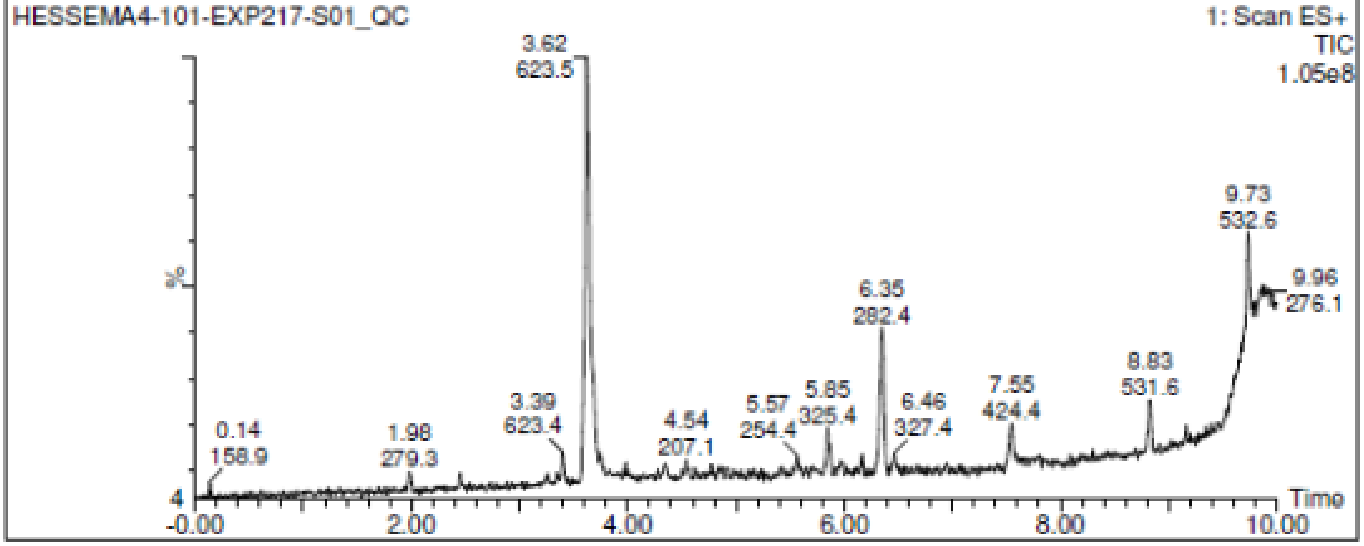

HPLC=0.1%TFA in H2O/ACN with 1.2mL/min

**EM23001594421**	**14-Feb-2023**<br>**13:06:02**<br>**strubhe1-0208A-v02**	**LCMS-ZQ1**<br>**Kinetex column,**<br>**ZQ1_10m_Acidic**

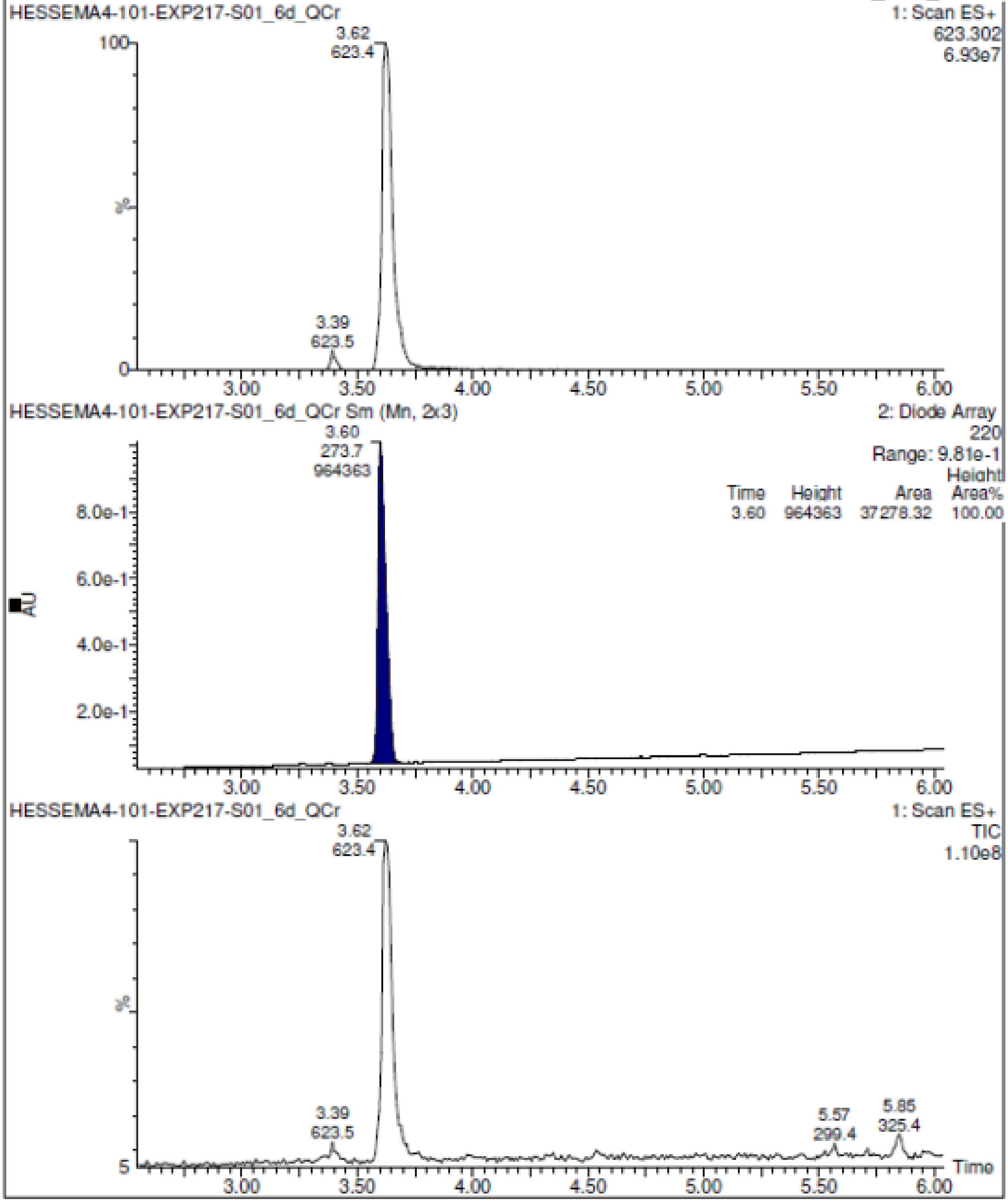

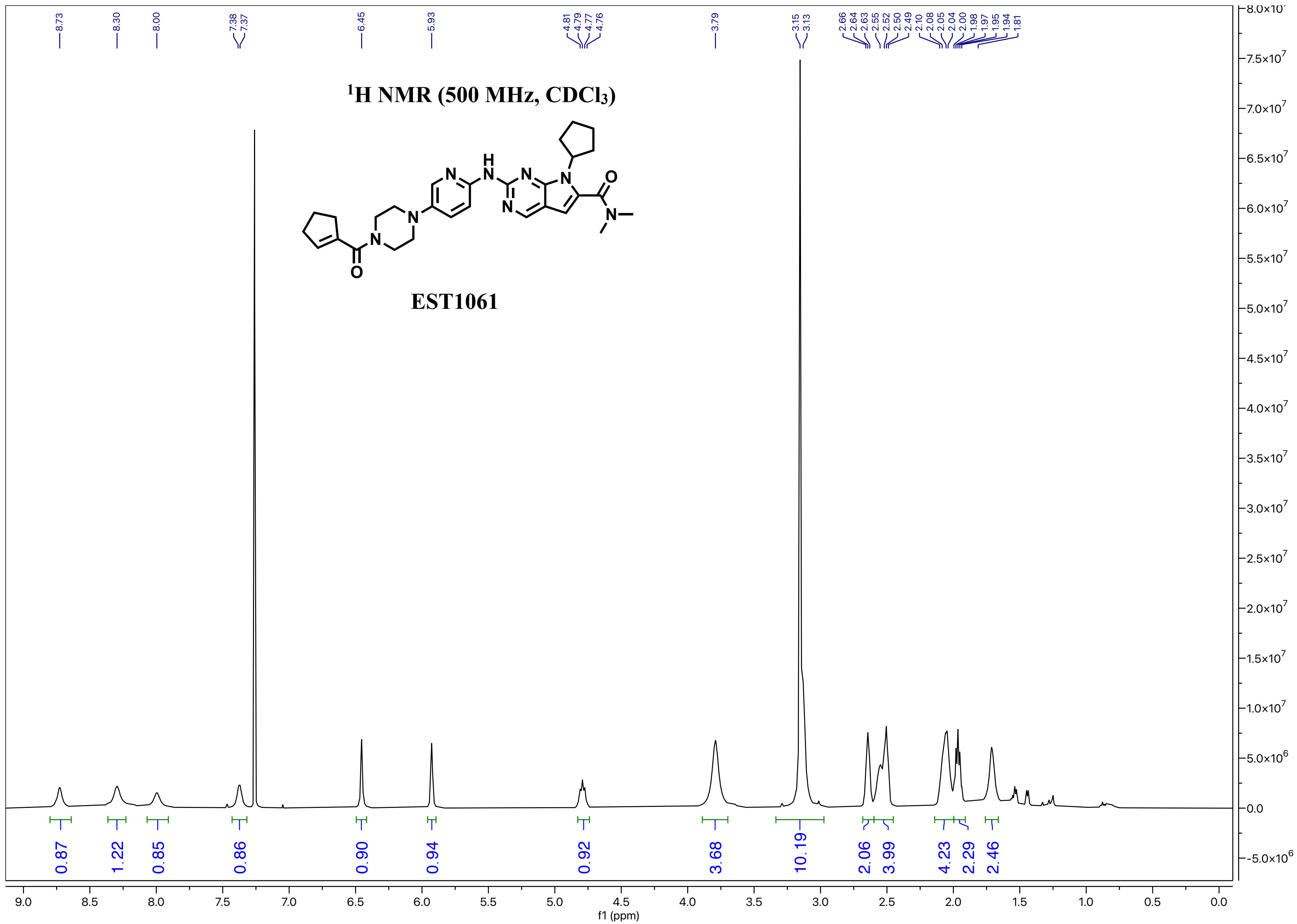

^{1}H NMR (500 MHz, CDCl$_3$)
EST1061
f1 (ppm)

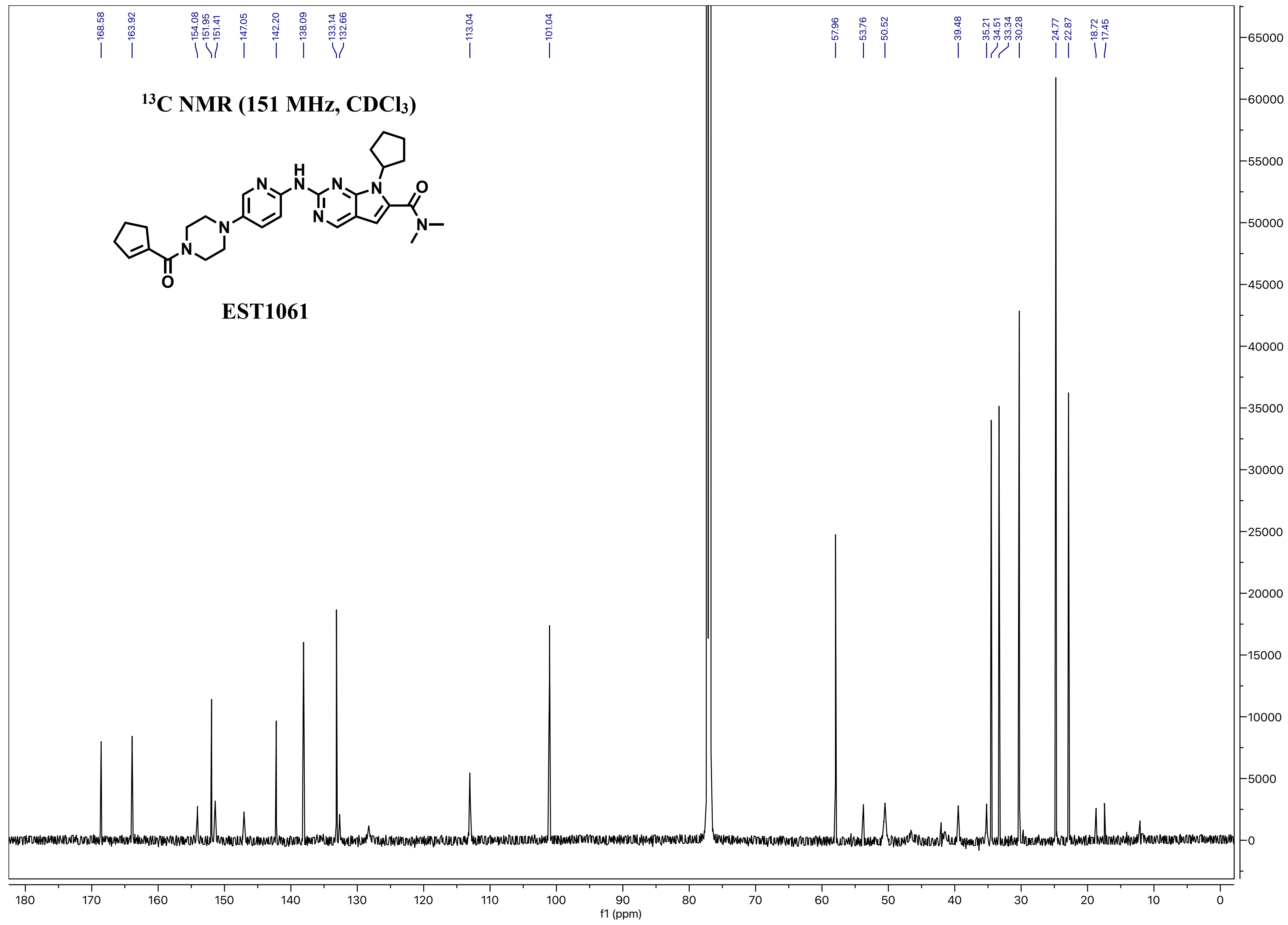
168.58
163.92
154.08
151.95
151.41
147.05
142.20
138.09
133.14
132.66
113.04
101.04
57.96
53.76
50.52
39.48
35.21
34.51
33.34
30.28
24.77
22.87
18.72
17.45
^{13}C NMR (151 MHz, $CDCl_3$)
EST1061
f1 (ppm)

HPLC=0.1%TFA in H2O/ACN with 1.2mL/min

**EM23001594438**	**08-Feb-2023**	**LCMS-ZQ1**
	**16:44:08**	**Kinetex column,**
	**strubhe1-0208A-v17**	**ZQ1_10m_Acidic**

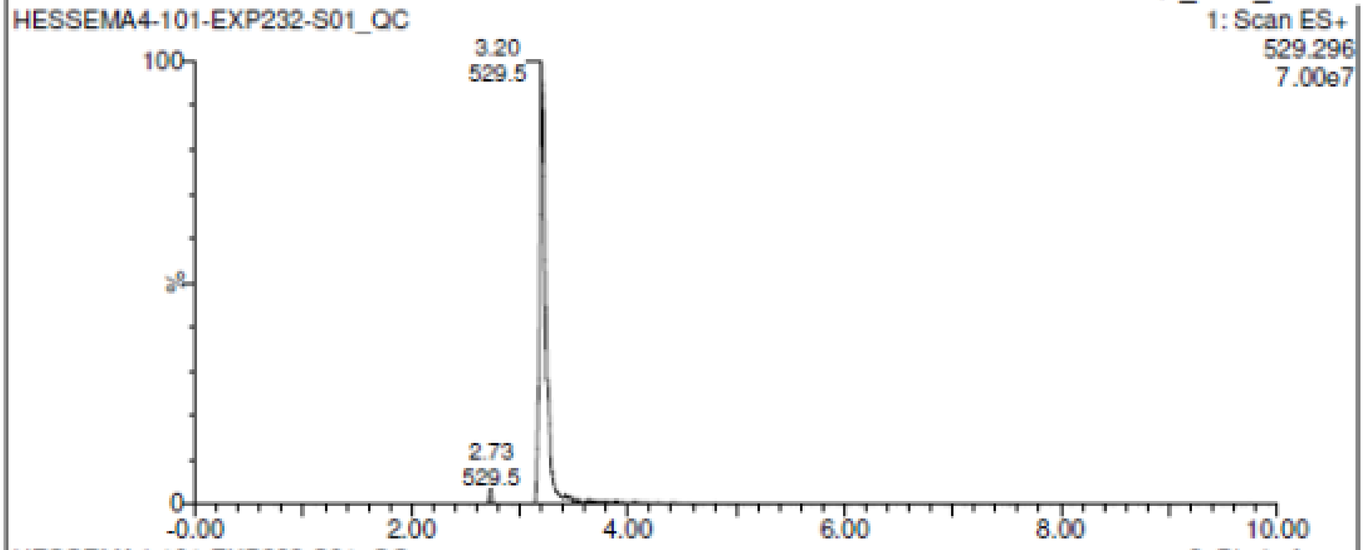

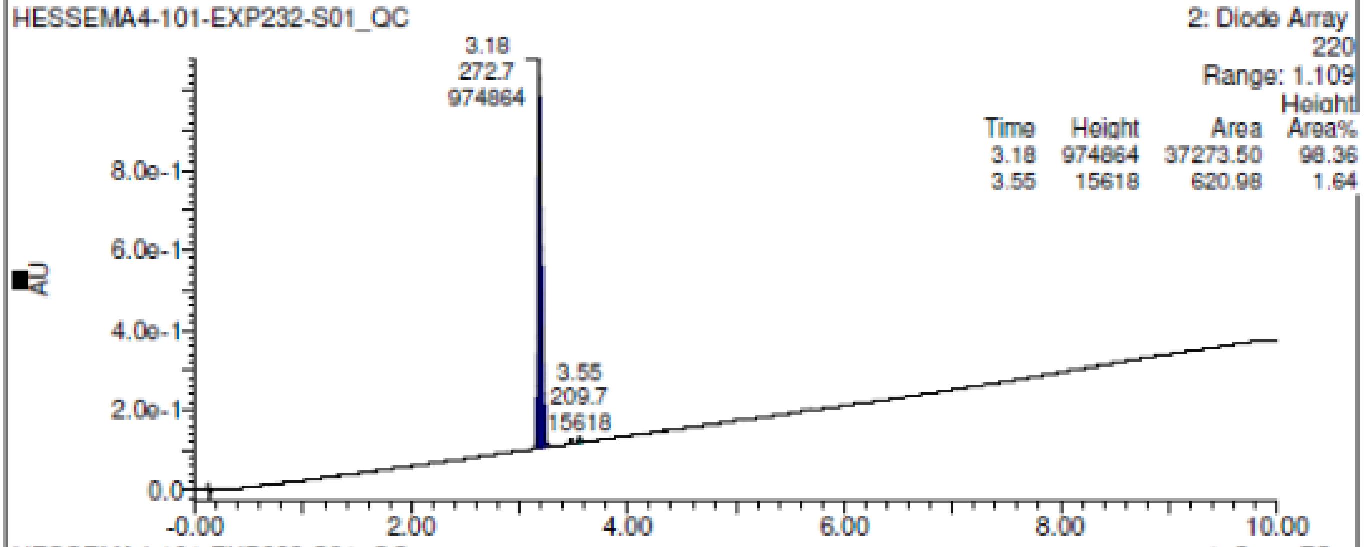

Time	Height	Area	Height Area%
3.18	974864	37273.50	98.36
3.55	15618	620.98	1.64

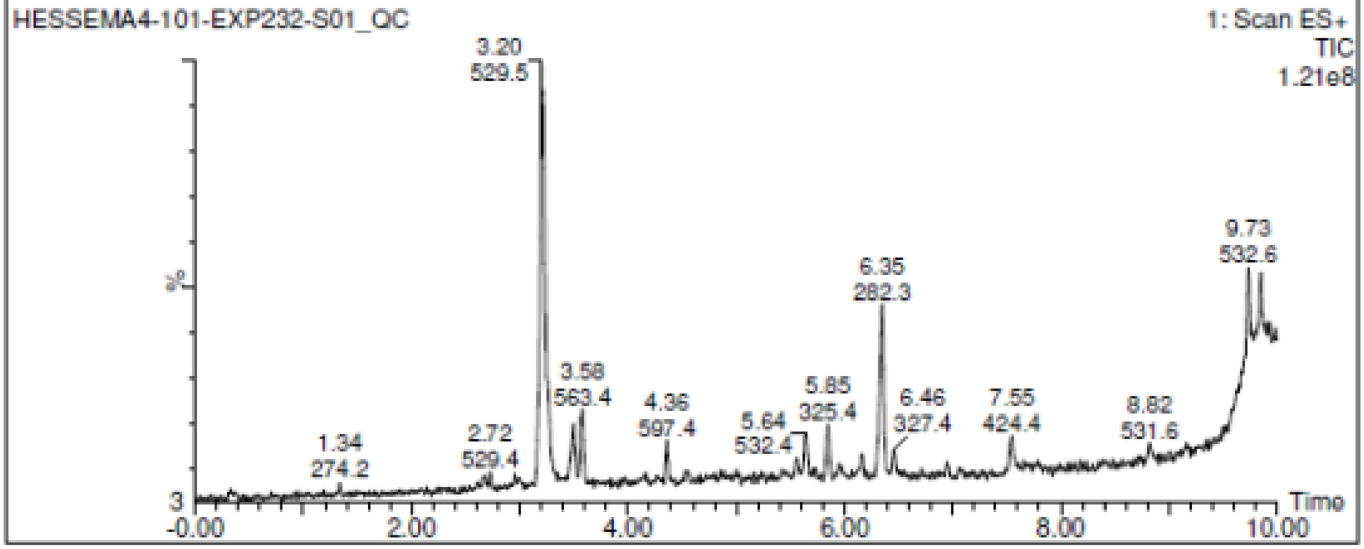

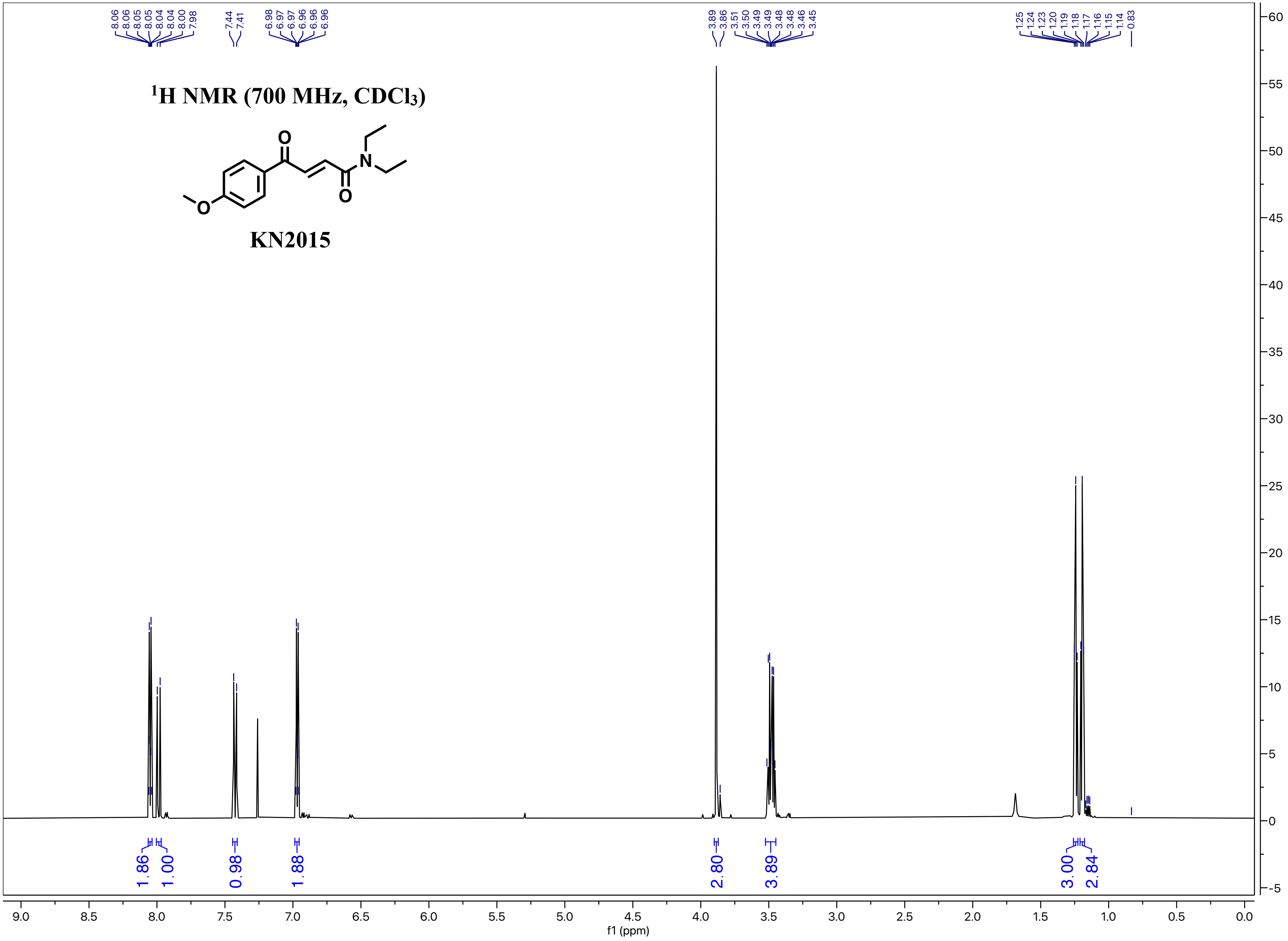

8.06
8.06
8.05
8.05
8.04
8.04
8.00
7.98
7.44
7.41
6.98
6.97
6.97
6.96
6.96
6.96
3.89
3.86
3.51
3.50
3.49
3.49
3.48
3.48
3.46
3.45
1.25
1.24
1.23
1.20
1.19
1.18
1.17
1.16
1.15
1.14
0.83
¹H NMR (700 MHz, CDCl₃)
O
O
N
O
KN2015
1.86
1.00
0.98
1.88
2.80
3.89
3.00
2.84
f1 (ppm)

13C NMR (126 MHz, CDCl3)

KN1025

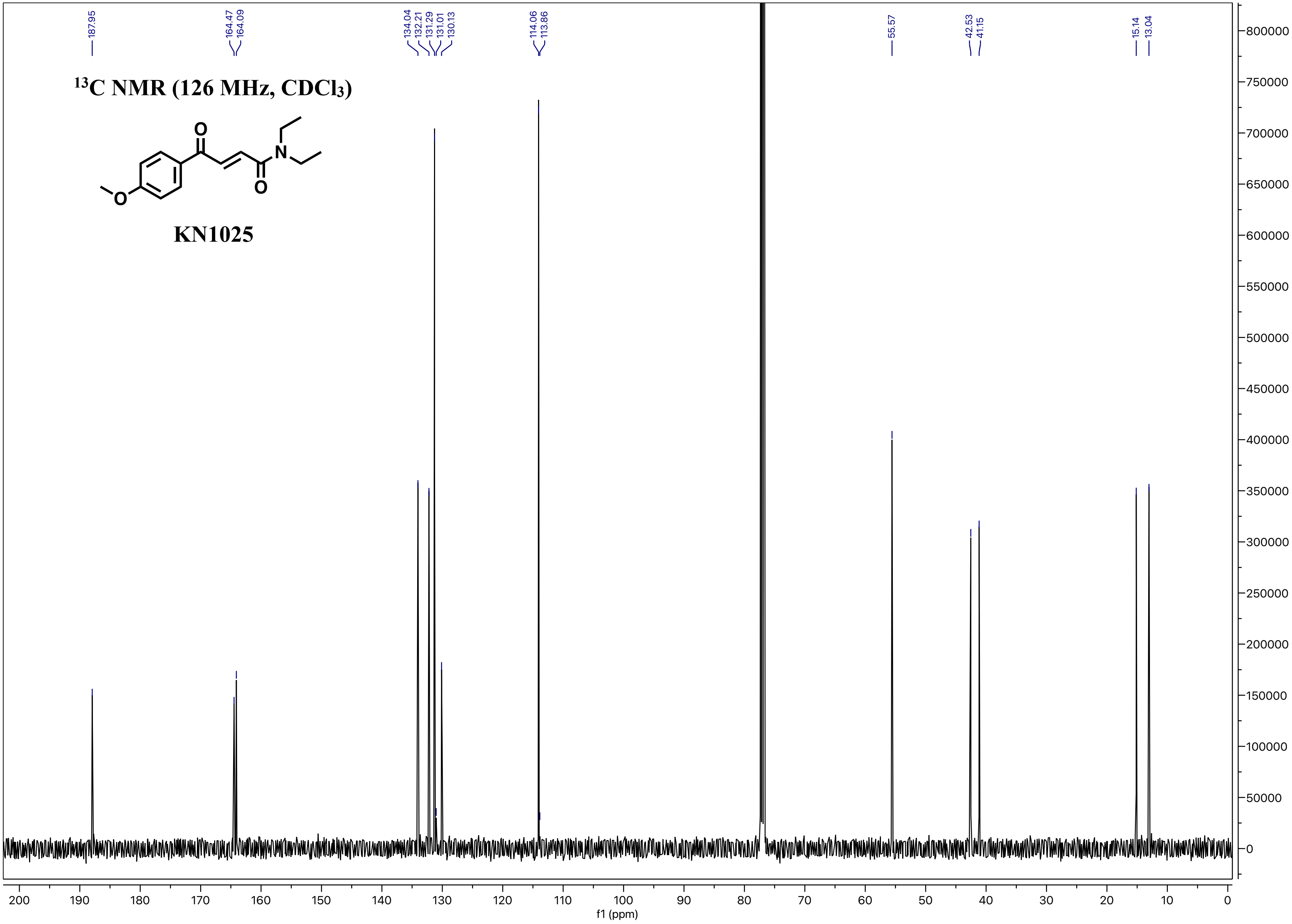

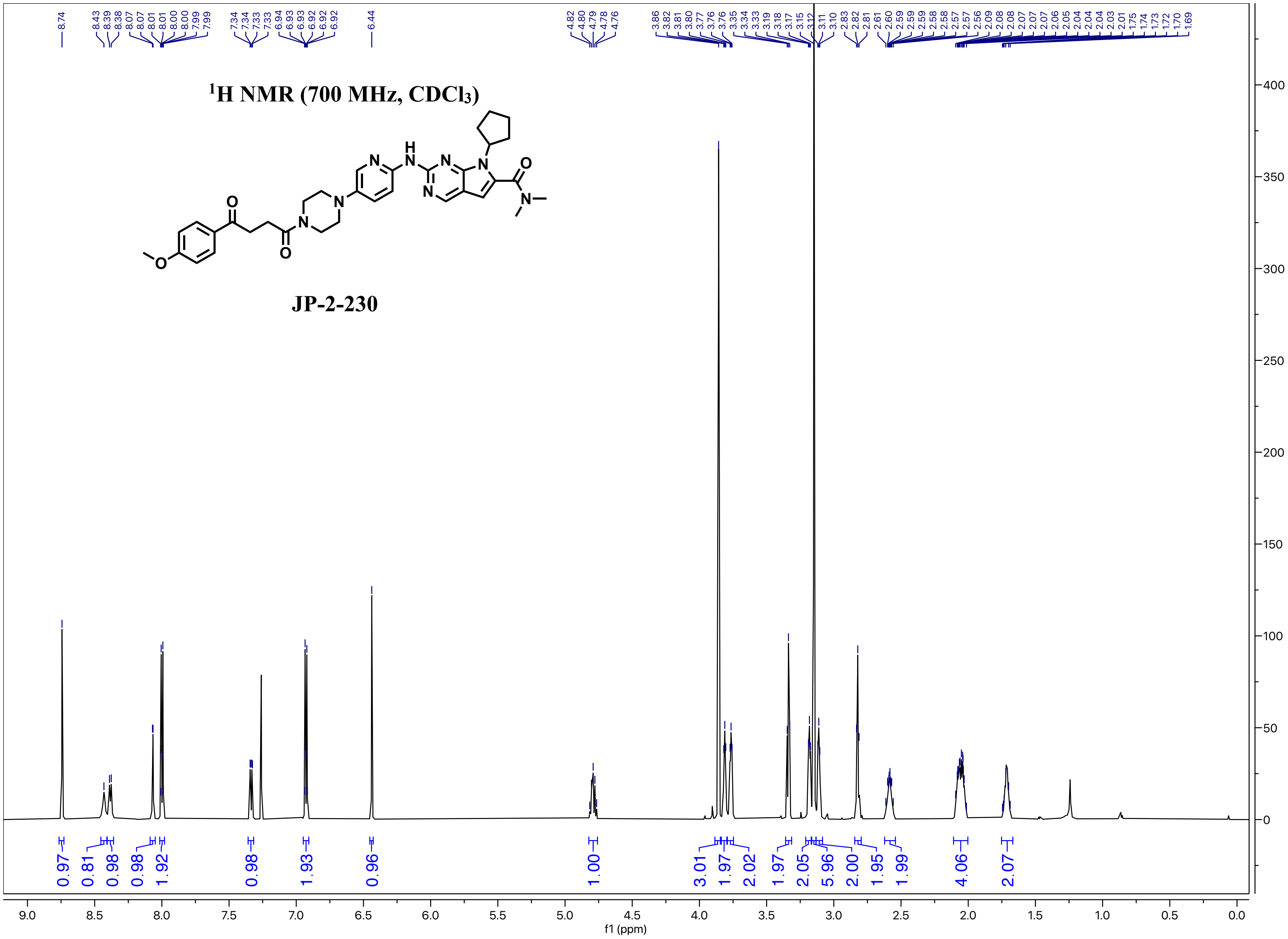

^{1}H NMR (700 MHz, $CDCl_3$)
JP-2-230
f1 (ppm)

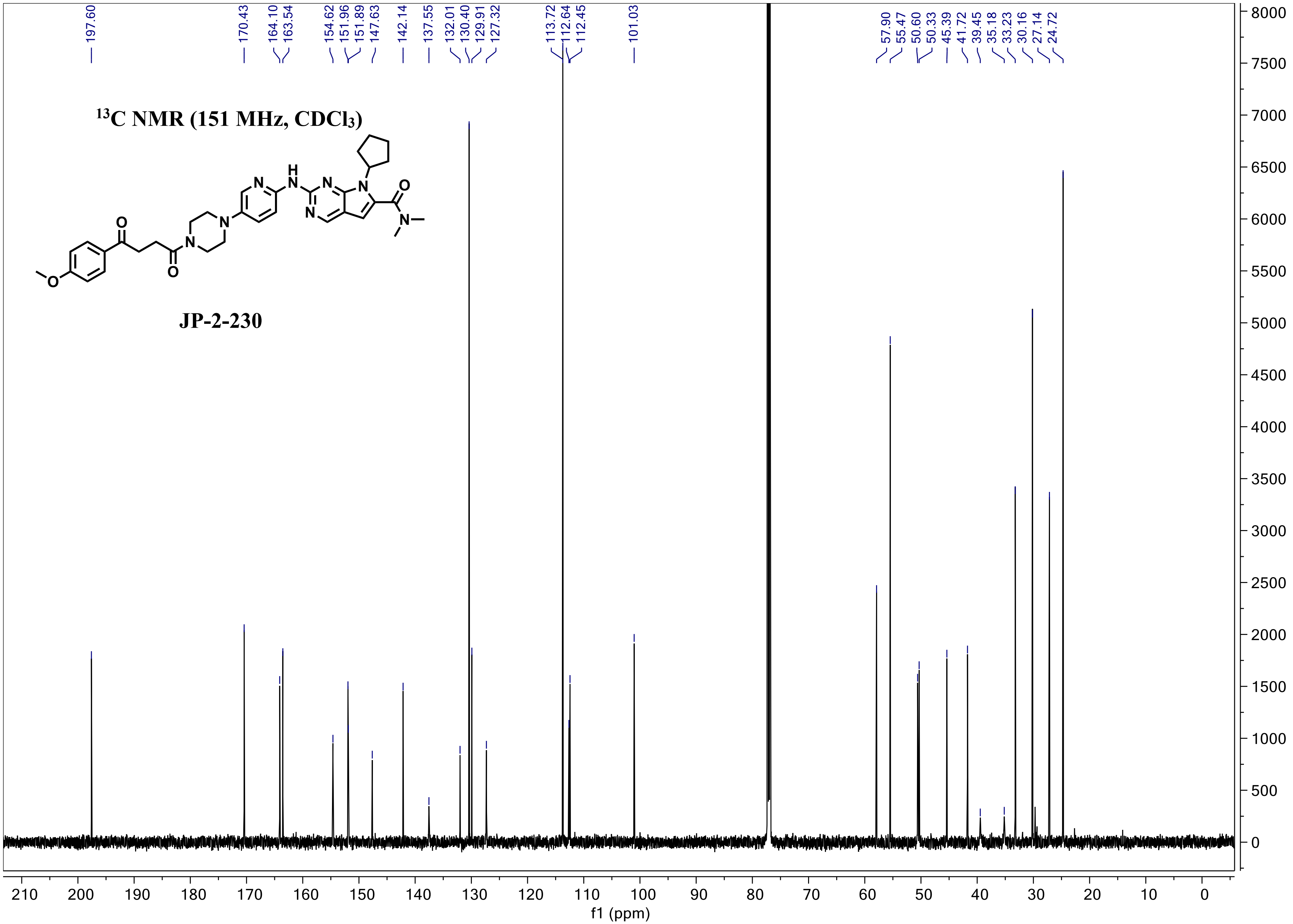

197.60
170.43
164.10
163.54
154.62
151.96
151.89
147.63
142.14
137.55
132.01
130.40
129.91
127.32
113.72
112.64
112.45
101.03
57.90
55.47
50.60
50.33
45.39
41.72
39.45
35.18
33.23
30.16
27.14
24.72
13C NMR (151 MHz, CDCl3)
JP-2-230
f1 (ppm)

HPLC=0.1%TFA in H2O/ACN with 1.2mL/min

EM23001594439 08-Feb-2023 16:55:09 strubhe1-0208A-v18 LCMS-ZQ1 Kinetex column, ZQ1_10m_Acidic

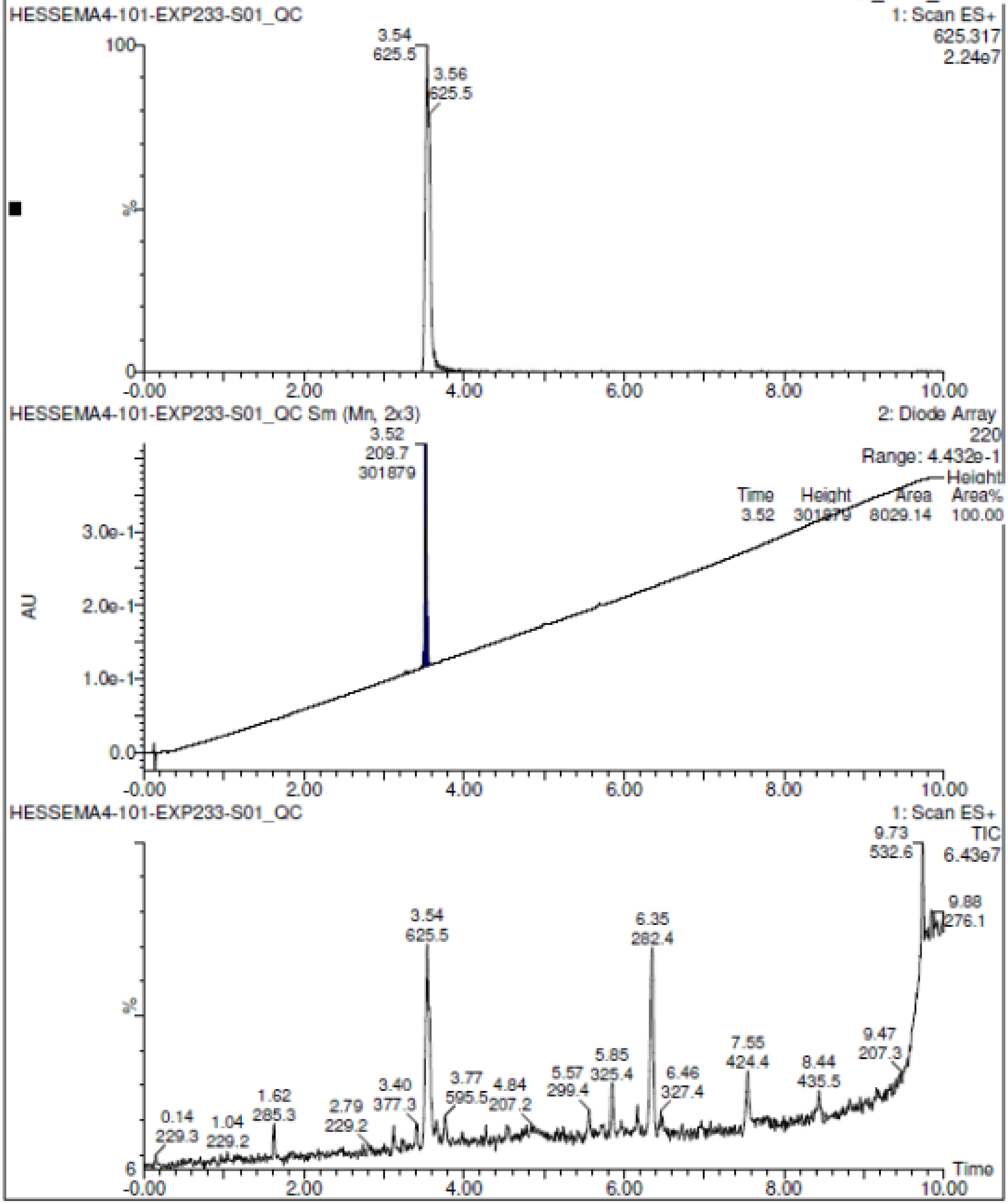

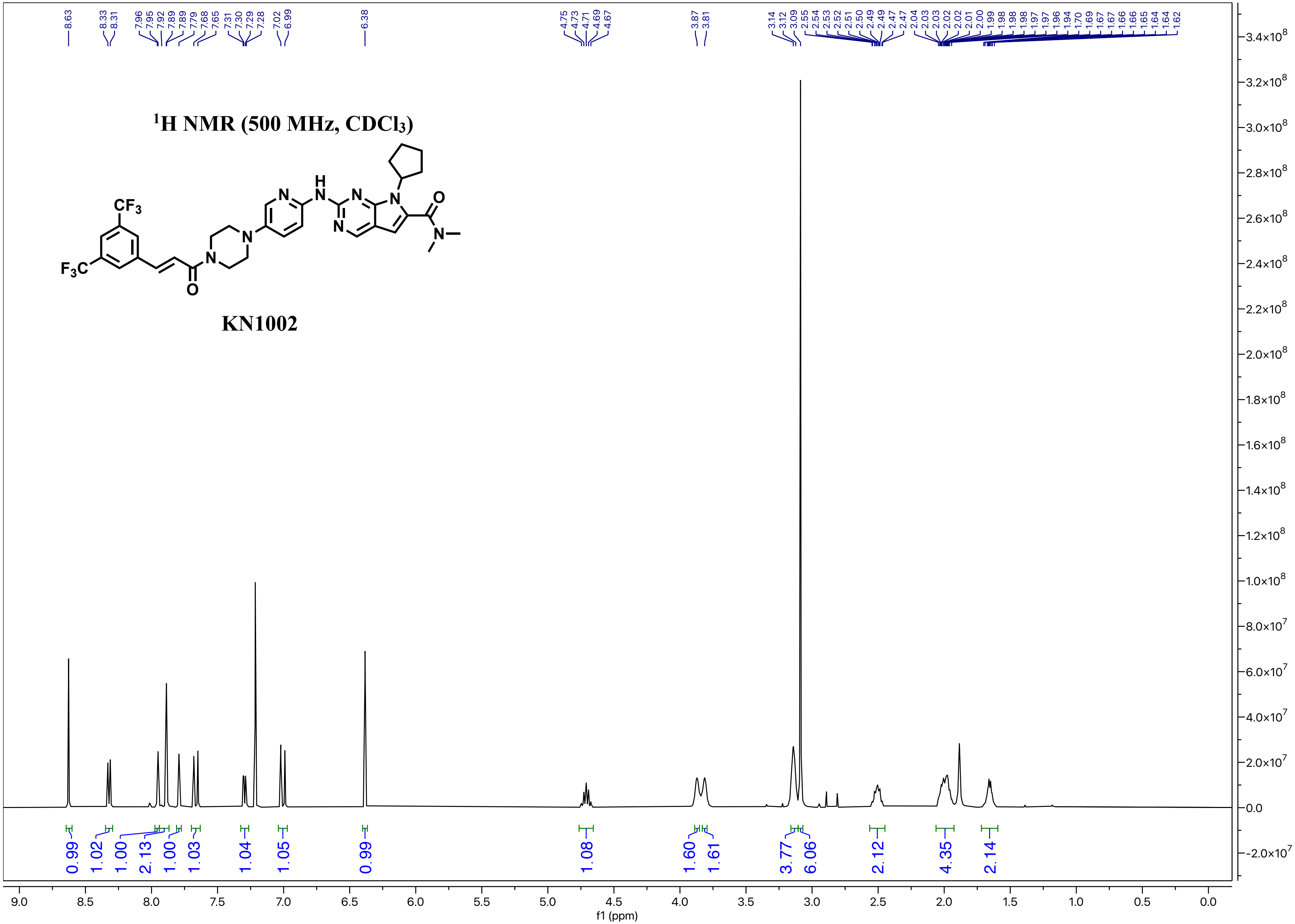

^{1}H NMR (500 MHz, CDCl3)
KN1002
f1 (ppm)

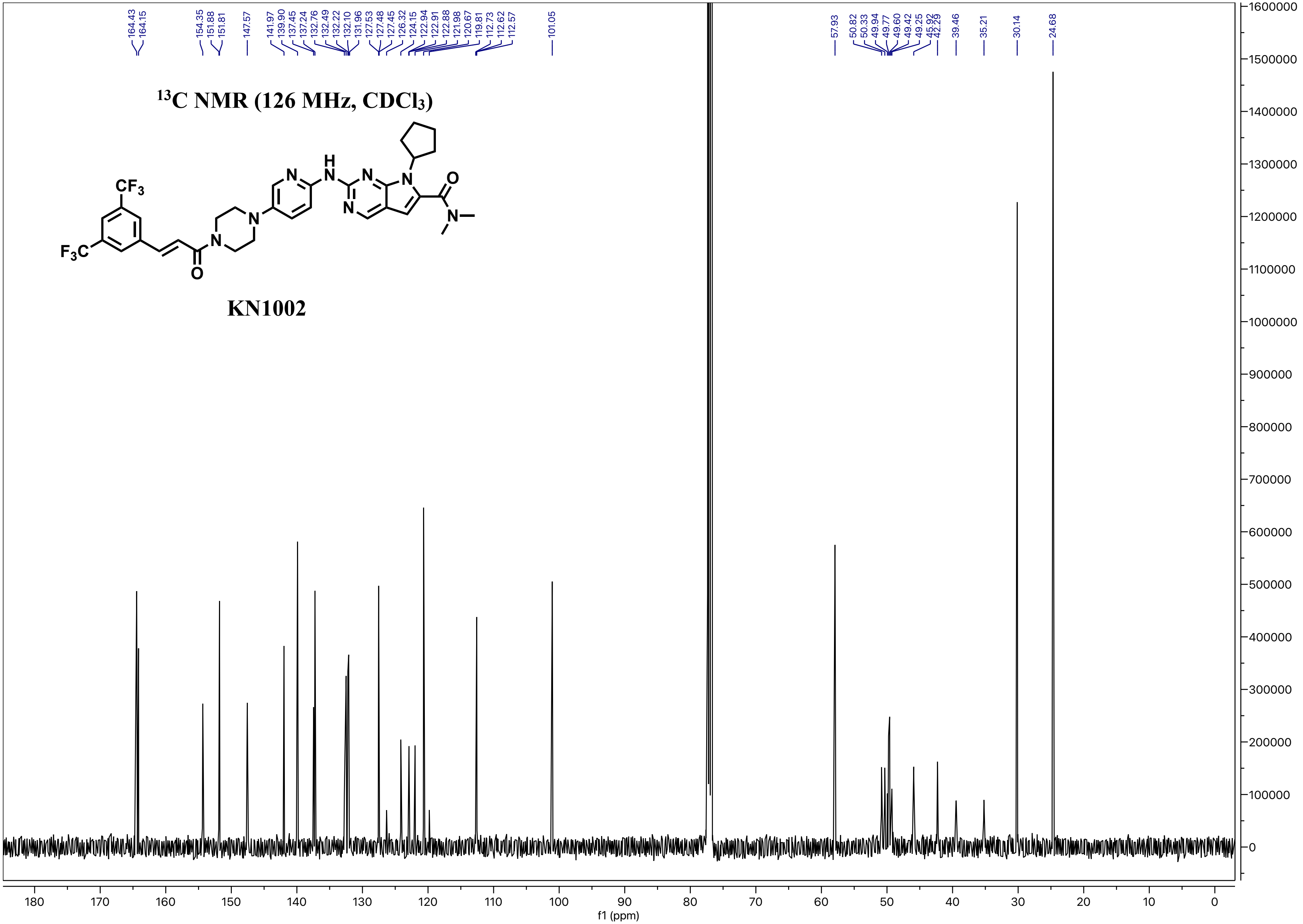

164.43
164.15
154.35
151.88
151.81
147.57
141.97
139.90
137.45
137.24
132.76
132.49
132.22
132.10
131.96
127.53
127.48
127.45
126.32
124.15
122.94
122.91
122.88
121.98
120.67
119.81
112.73
112.62
112.57
101.05
57.93
50.82
50.33
49.94
49.77
49.60
49.42
49.25
45.92
42.29
39.46
35.21
30.14
24.68
^{13}C NMR (126 MHz, CDCl$_3$)
CF$_3$
F$_3$C
KN1002
f1 (ppm)

HPLC=0.1%TFA in H2O/ACN with 1.2mL/min

EM23001594442 | **08-Feb-2023** | **LCMS-ZQ1**

17:06:11 | **Kinetex column,**

strubhe1-0208A-v19 | **ZQ1_10m_Acidic**

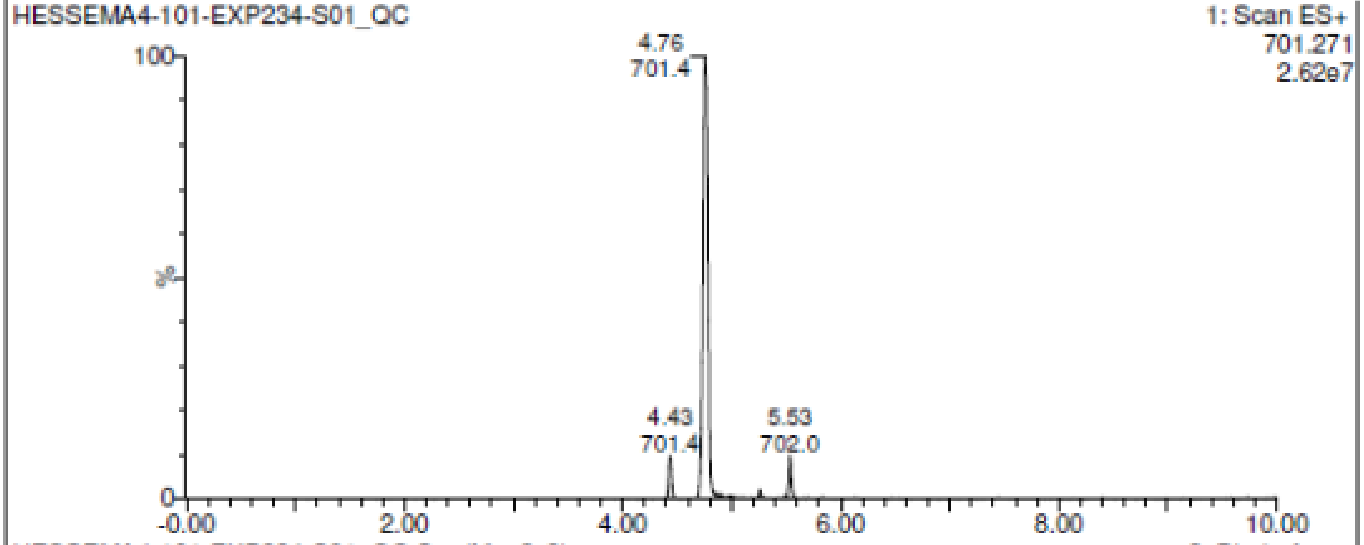

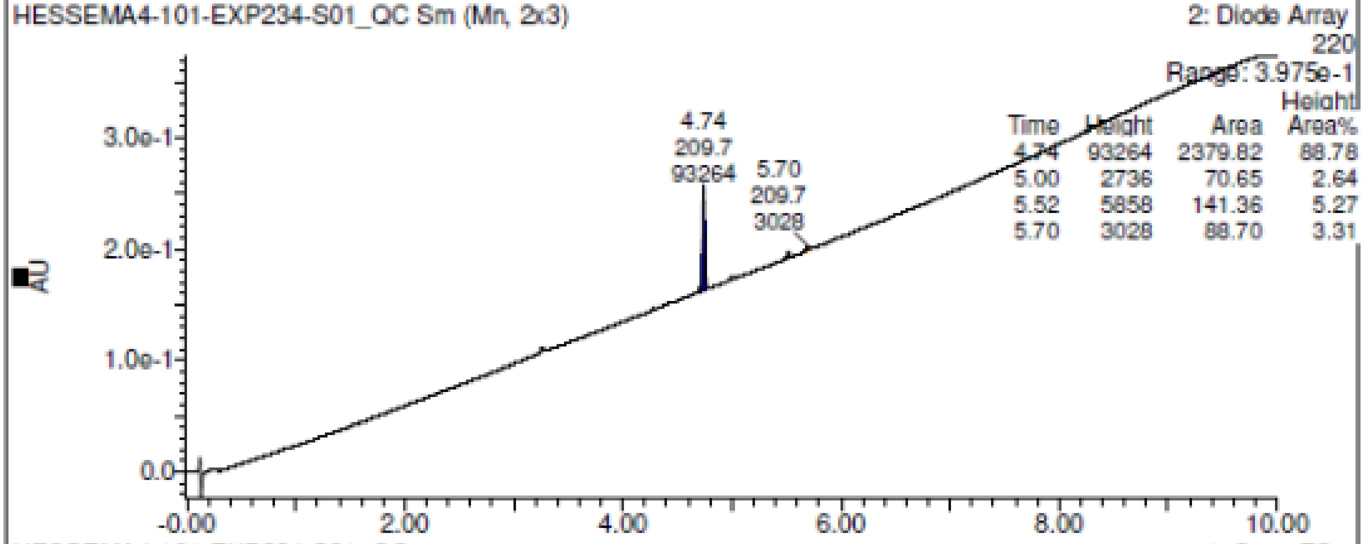

Time	Height	Area	Height Area%
4.74	93264	2379.82	88.78
5.00	2736	70.65	2.64
5.52	5858	141.36	5.27
5.70	3028	88.70	3.31

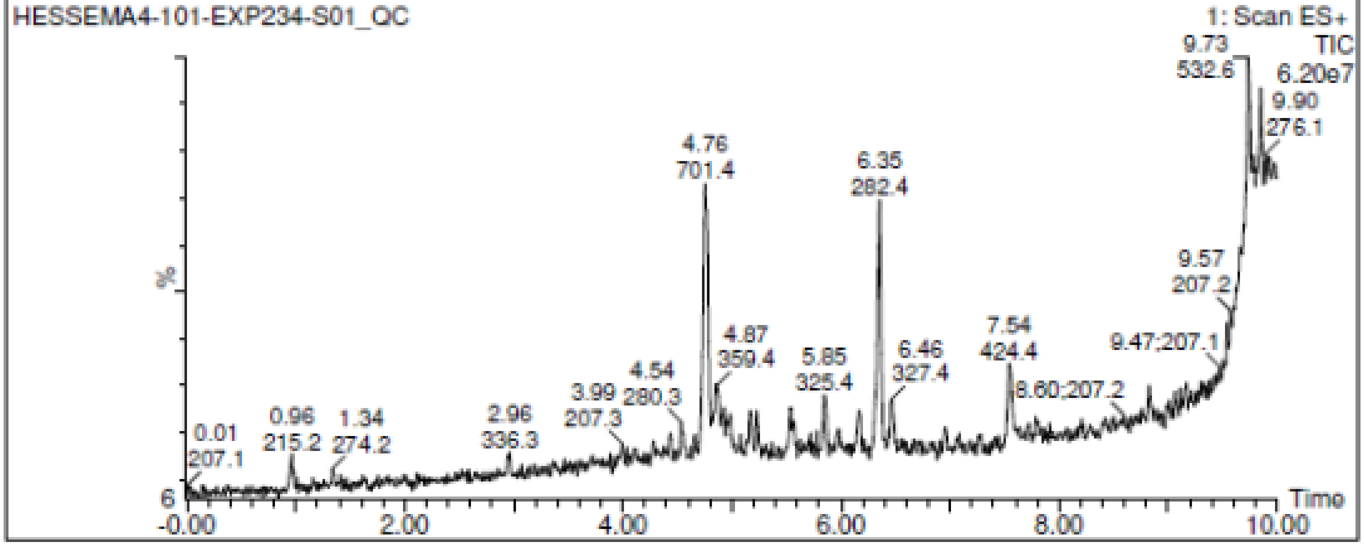

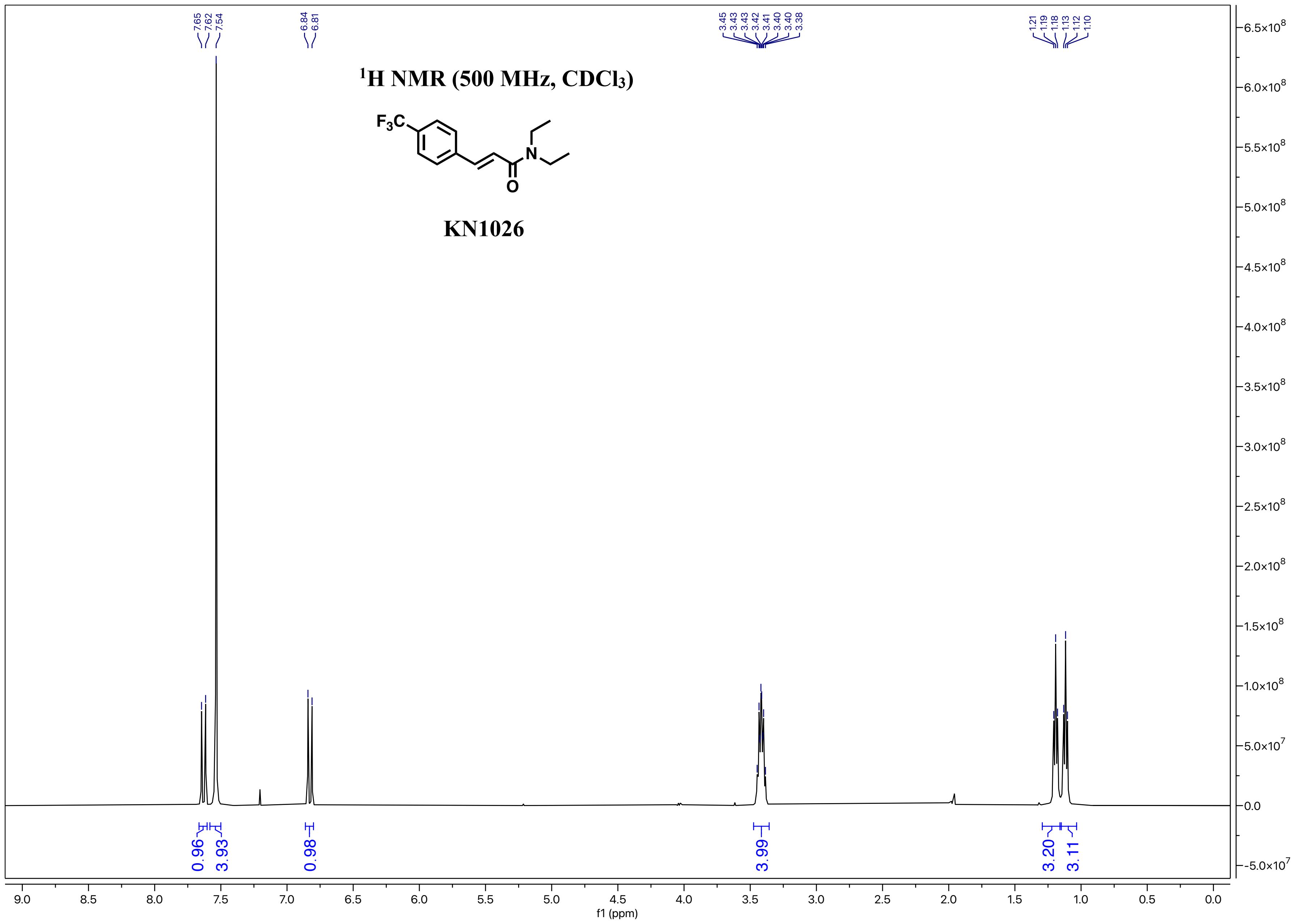

7.65
7.62
7.54
6.84
6.81
3.45
3.43
3.43
3.42
3.41
3.40
3.40
3.38
1.21
1.19
1.18
1.13
1.12
1.10
¹H NMR (500 MHz, CDCl3)
F3C
N
O
KN1026
0.96
3.93
0.98
3.99
3.20
3.11
f1 (ppm)

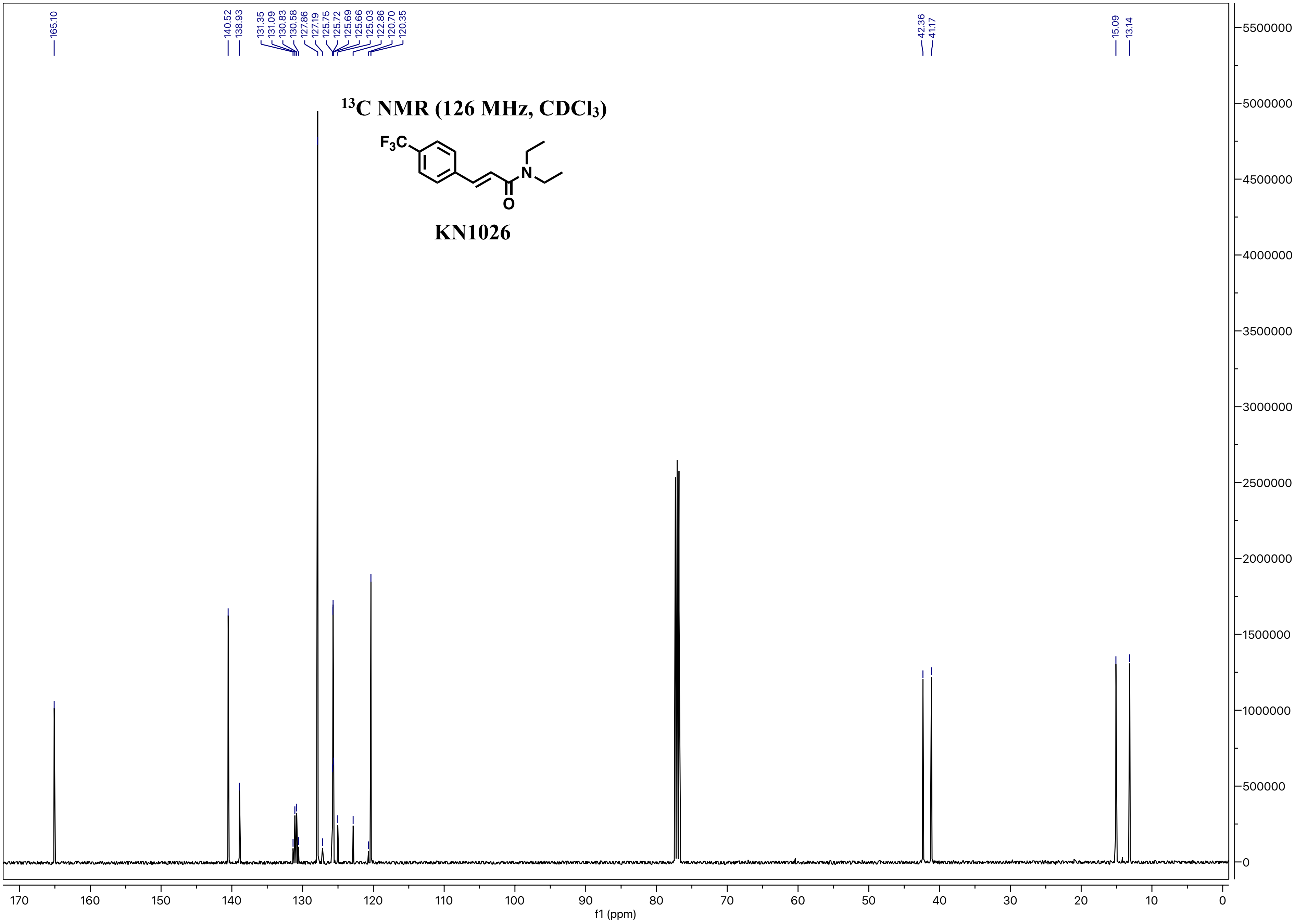
165.10
140.52
138.93
131.35
131.09
130.83
130.58
127.86
127.19
125.75
125.72
125.69
125.66
125.03
122.86
120.70
120.35
42.36
41.17
15.09
13.14
13C NMR (126 MHz, CDCl3)
F3C
N
O
KN1026
5500000
5000000
4500000
4000000
3500000
3000000
2500000
2000000
1500000
1000000
500000
0
170
160
150
140
130
120
110
100
90
80
70
60
50
40
30
20
10
0
f1 (ppm)

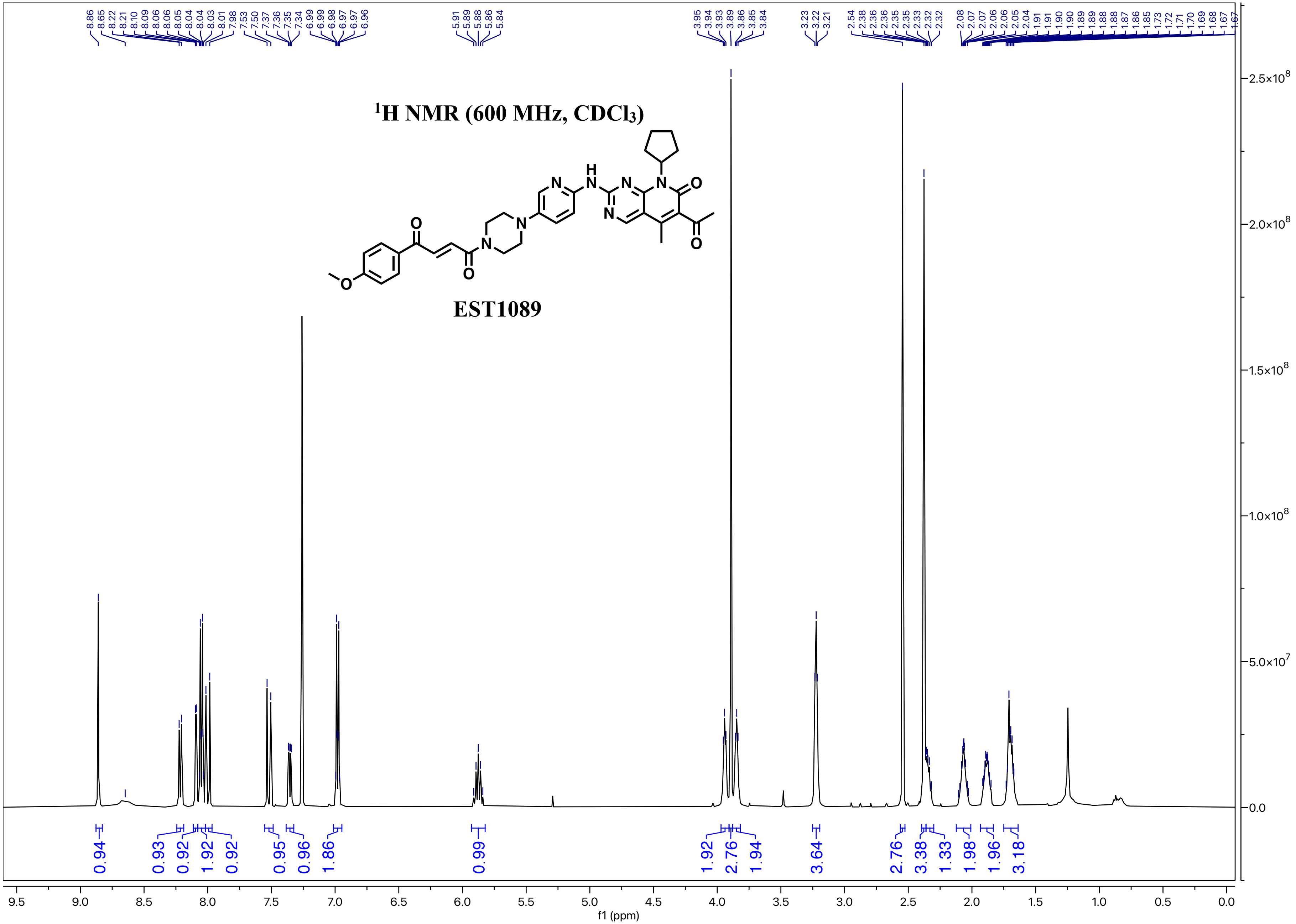

^{1}H NMR (600 MHz, CDCl3)
EST1089
f1 (ppm)

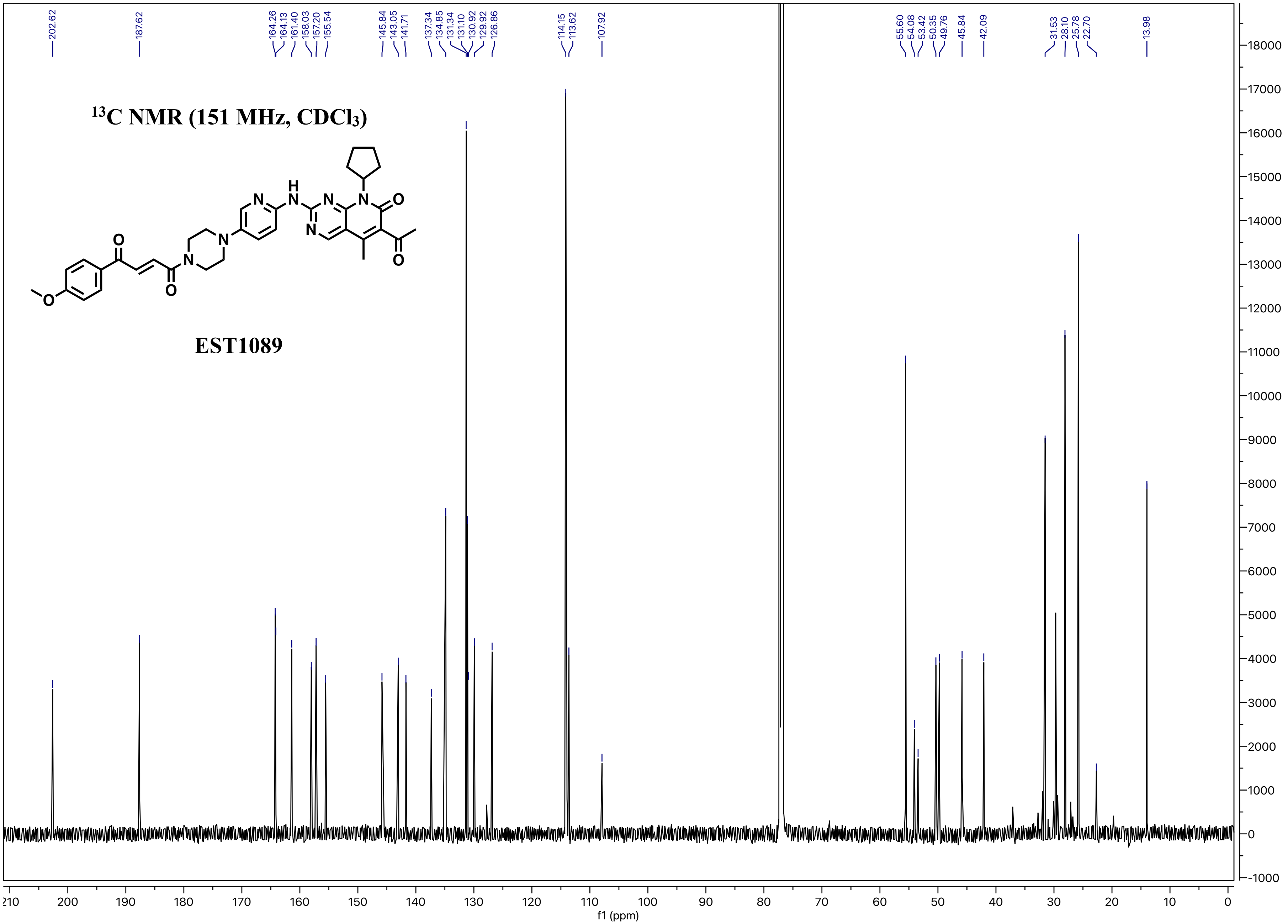

^{13}C NMR (151 MHz, $CDCl_3$)
EST1089
202.62
187.62
164.26
164.13
161.40
158.03
157.20
155.54
145.84
143.05
141.71
137.34
134.85
131.34
131.10
130.92
129.92
126.86
114.15
113.62
107.92
55.60
54.08
53.42
50.35
49.76
45.84
42.09
31.53
28.10
25.78
22.70
13.98
f1 (ppm)

HPLC=0.1%TFA in H2O/ACN with 1.2mL/min

**EM23001590295**	**13-Jan-2023**	**LCMS-ZQ1**
	**16:38:55**	**Kinetex column,**
	**strubhe1-0112A-v09**	**ZQ1_10m_Acidic**

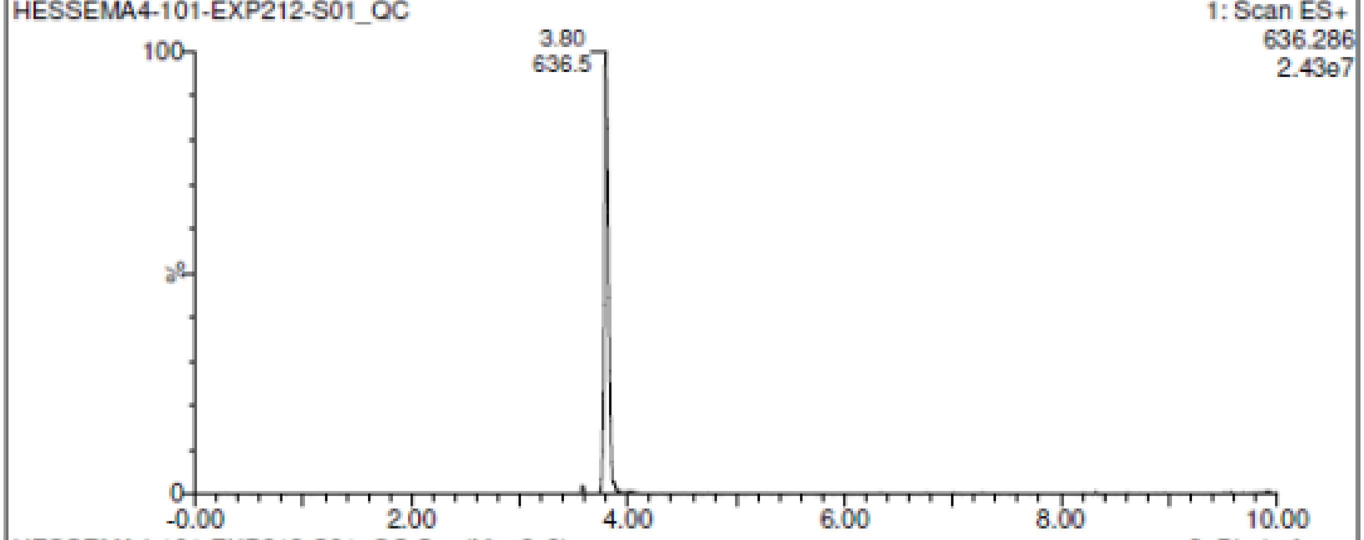

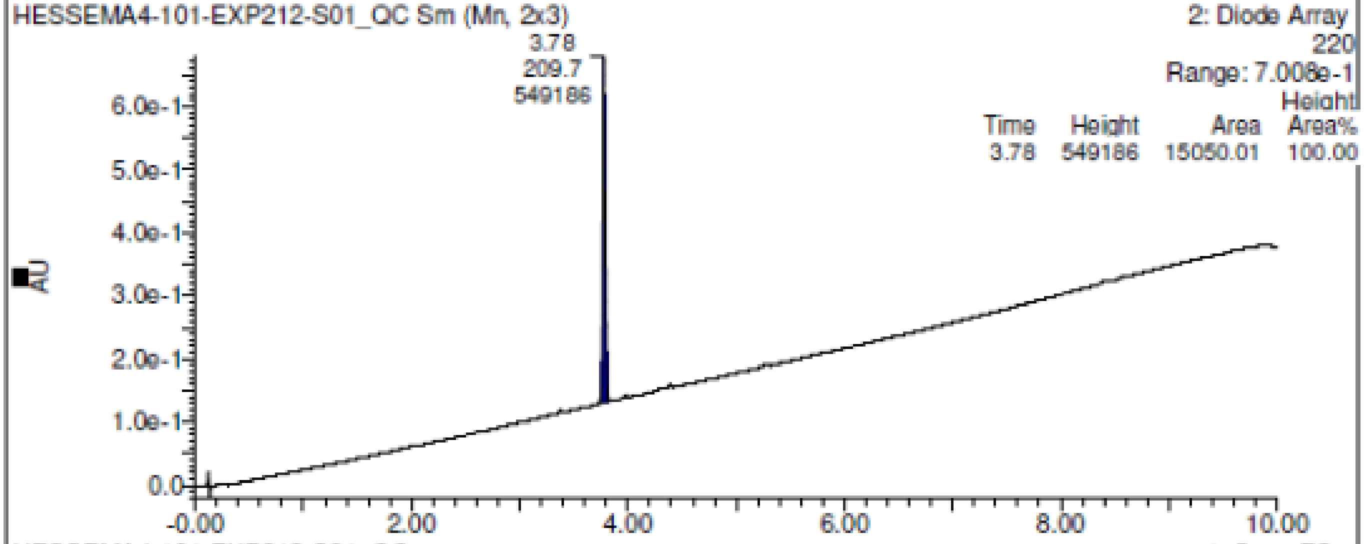

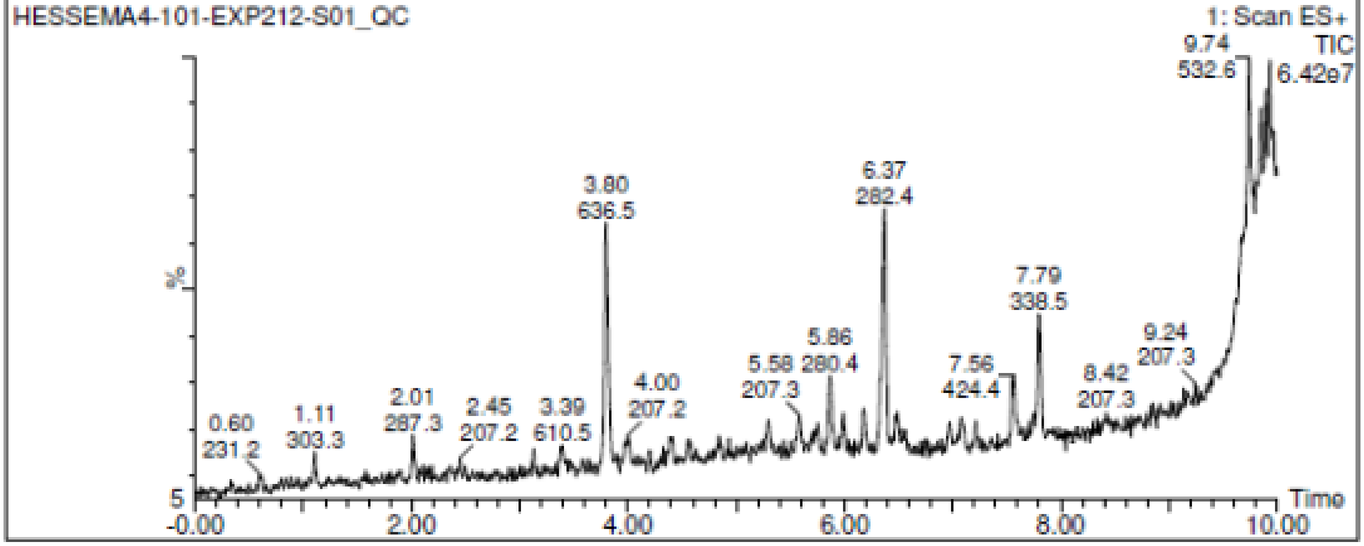

HPLC=0.1%TFA in H2O/ACN with 1.2mL/min

**EM23001590295**	**19-Jan-2023**	**LCMS-ZQ1**
	**17:04:54**	**Kinetex column,**
	**strubhe1-0118A-v09**	**ZQ1_10m_Acidic**

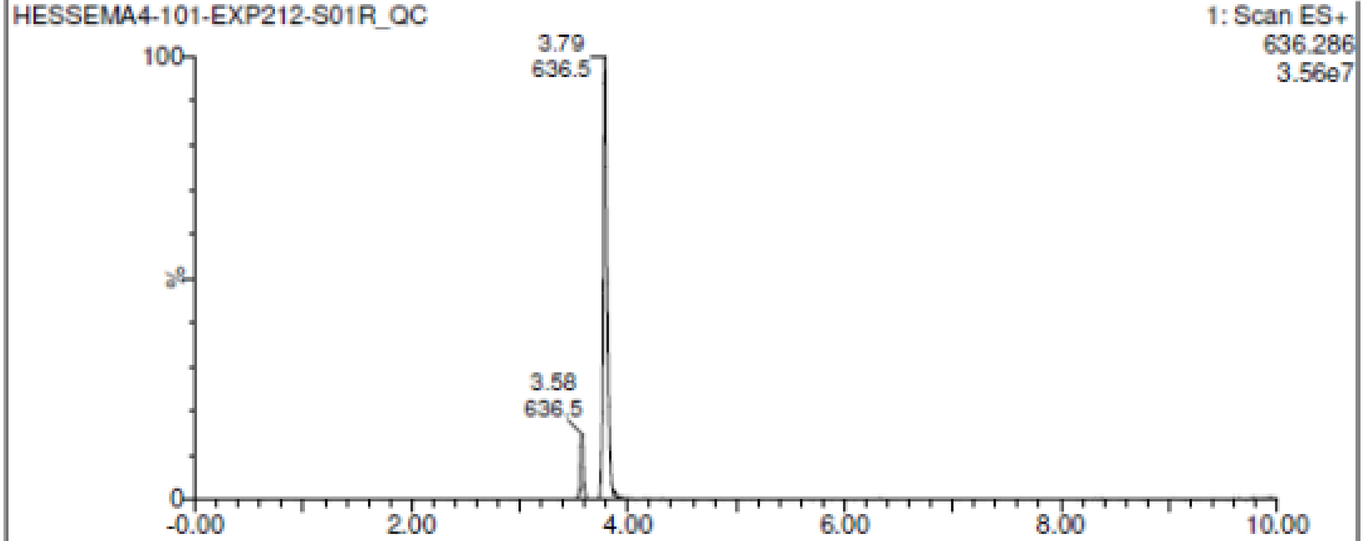

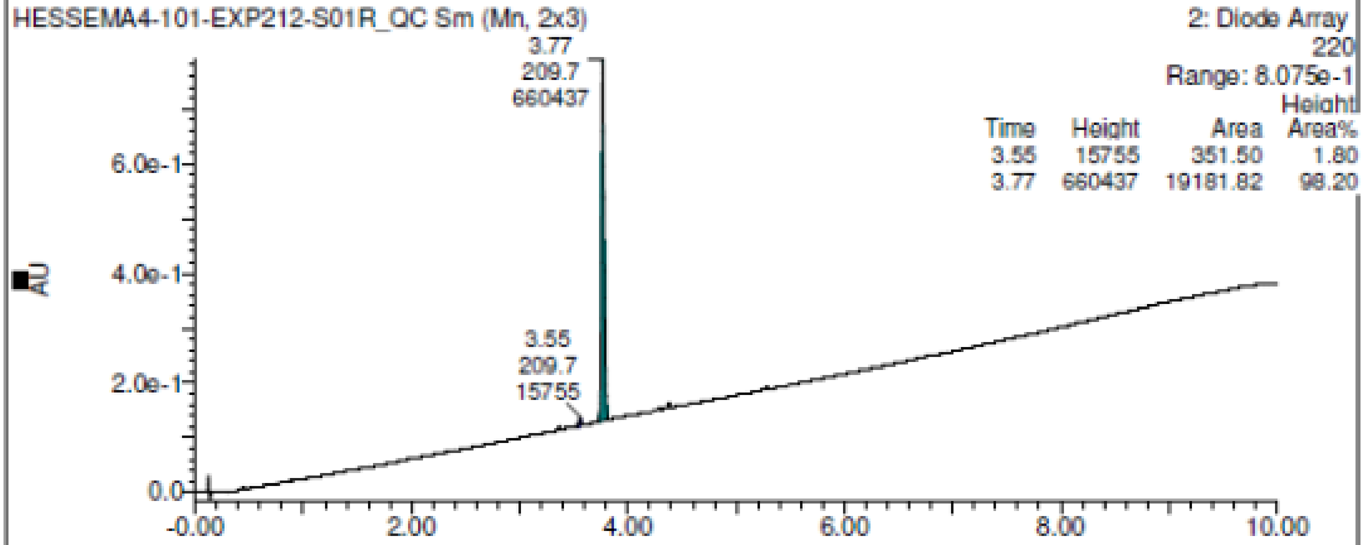

Time	Height	Area	Area%
3.55	15755	351.50	1.80
3.77	660437	19181.82	98.20

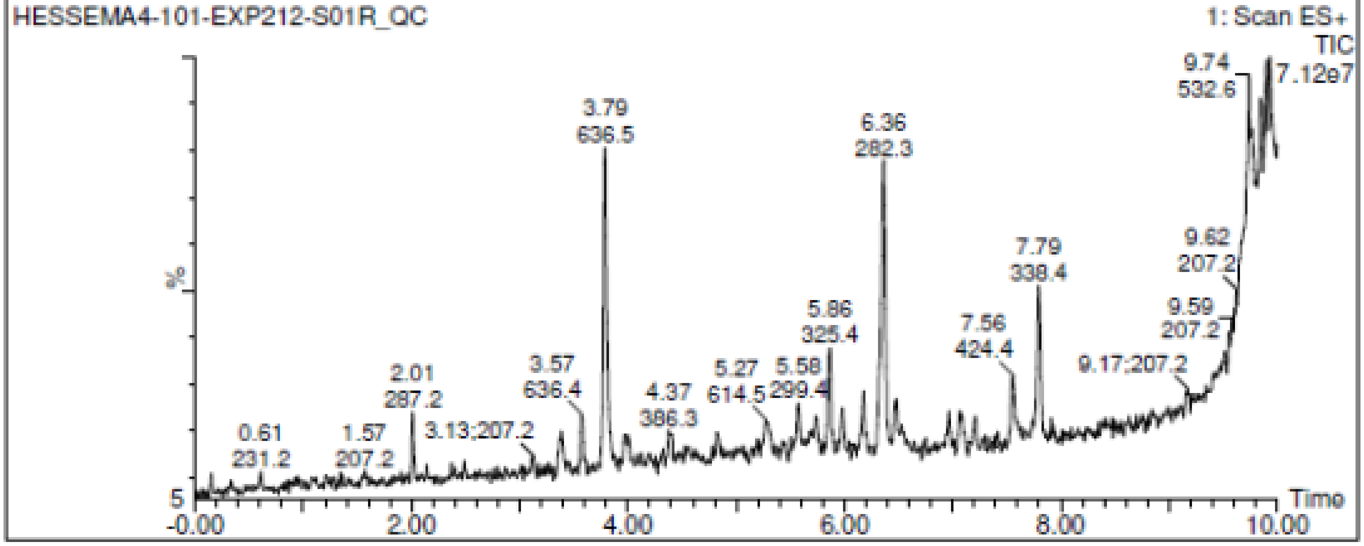

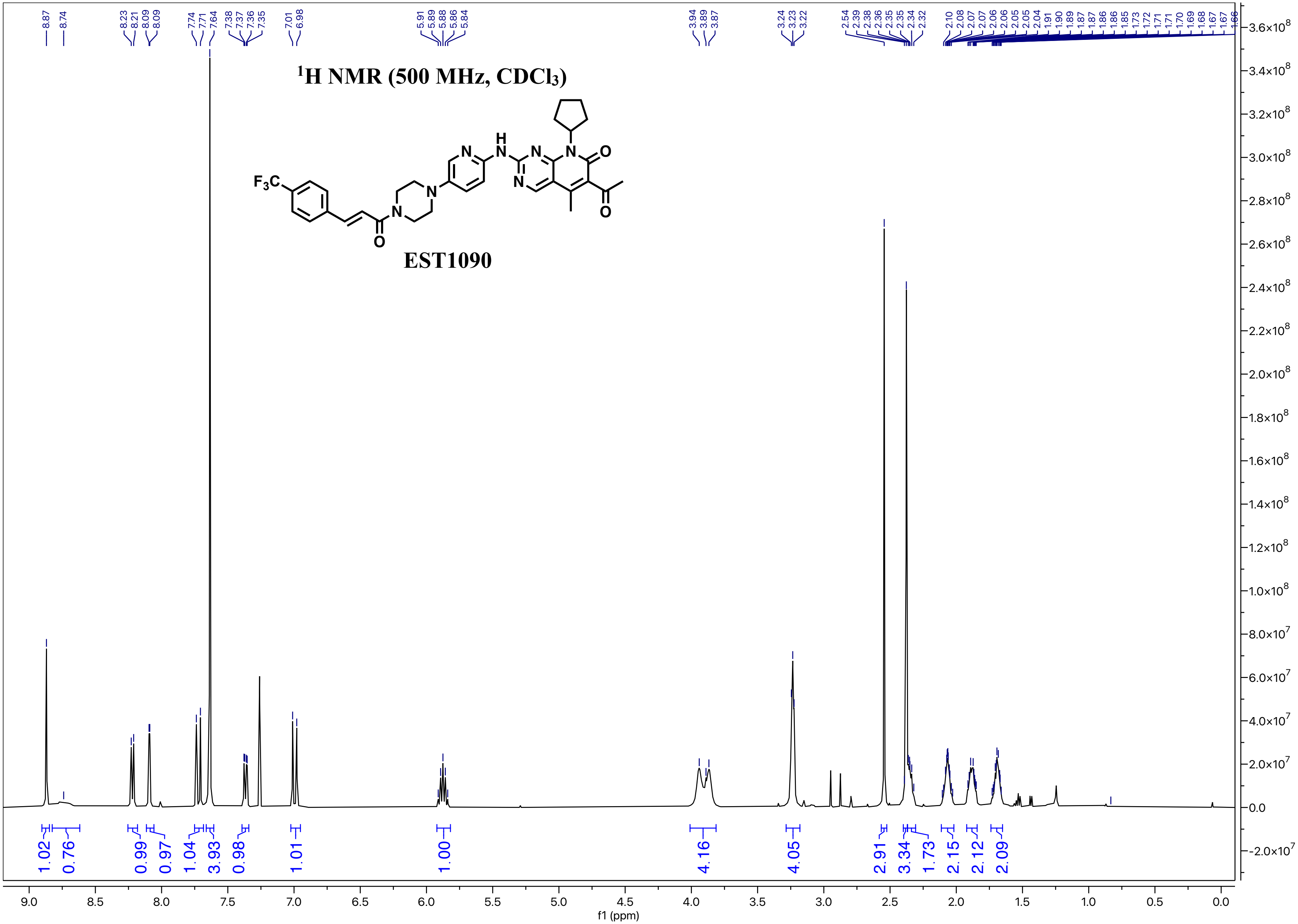

¹H NMR (500 MHz, CDCl3)
EST1090
f1 (ppm)

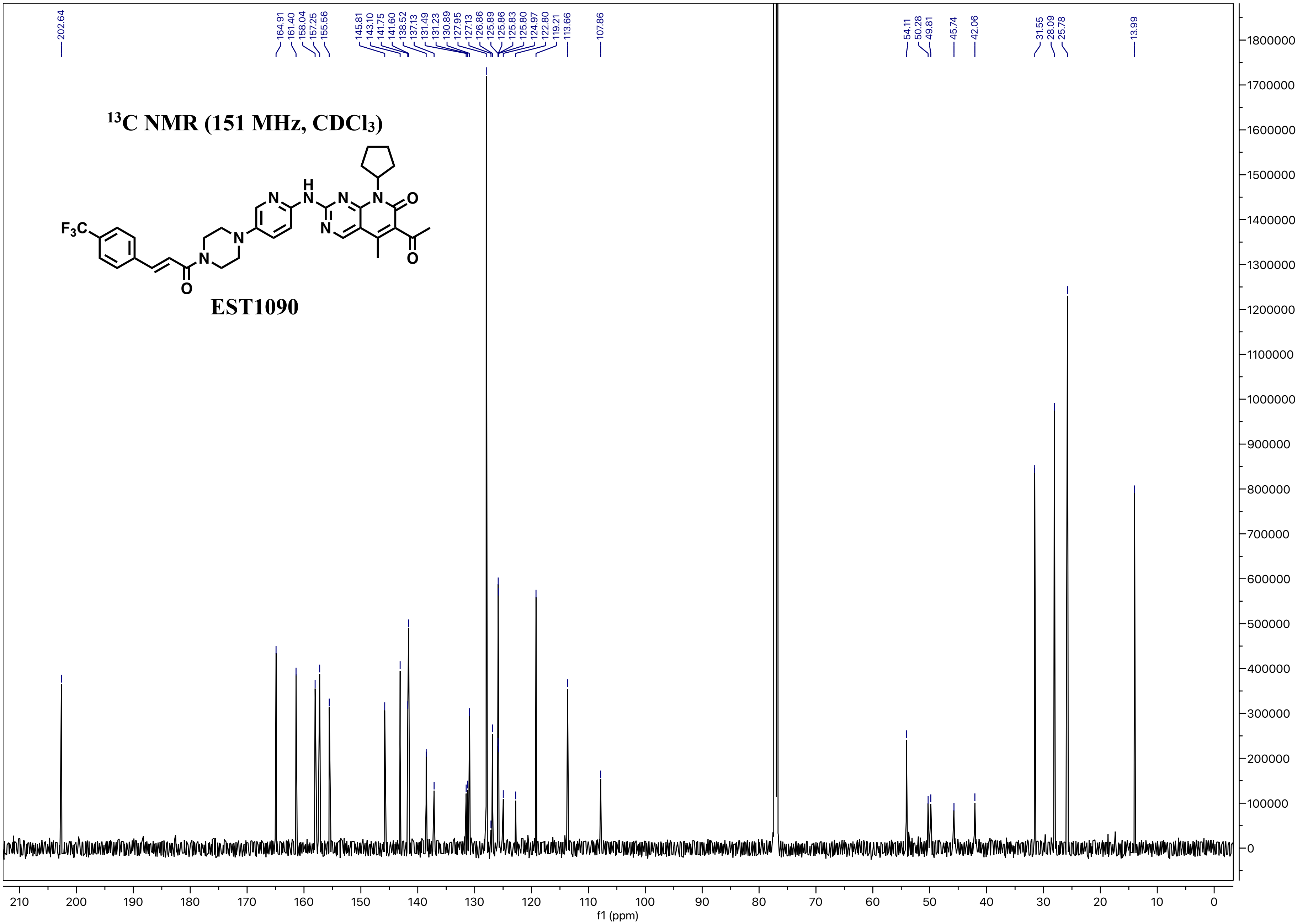

202.64
164.91
161.40
158.04
157.25
155.56
145.81
143.10
141.75
141.60
138.52
137.13
131.49
131.23
130.89
127.95
127.13
126.86
125.89
125.86
125.83
125.80
124.97
122.80
119.21
113.66
107.86
54.11
50.28
49.81
45.74
42.06
31.55
28.09
25.78
13.99
^{13}C NMR (151 MHz, CDCl$_3$)
EST1090
f1 (ppm)

HPLC=0.1%TFA in H2O/ACN with 1.2mL/min

**EM23001594444**	**08-Feb-2023**<br>**17:17:14**<br>**strubhe1-0208A-v20**	**LCMS-ZQ1**<br>**Kinetex column,**<br>**ZQ1_10m_Acidic**

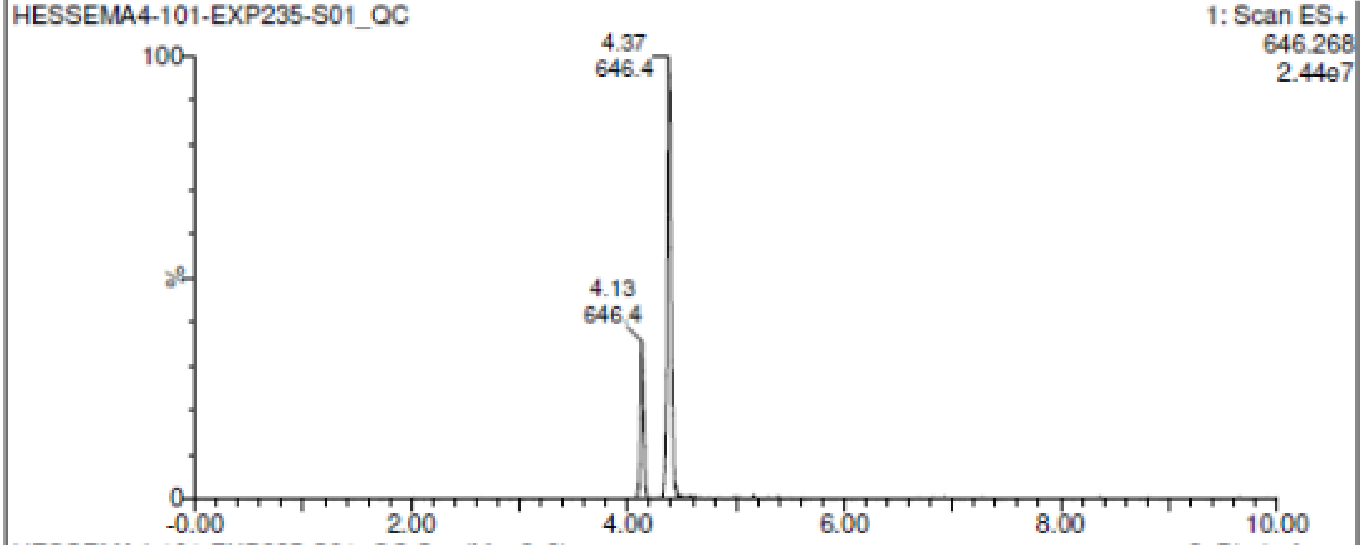

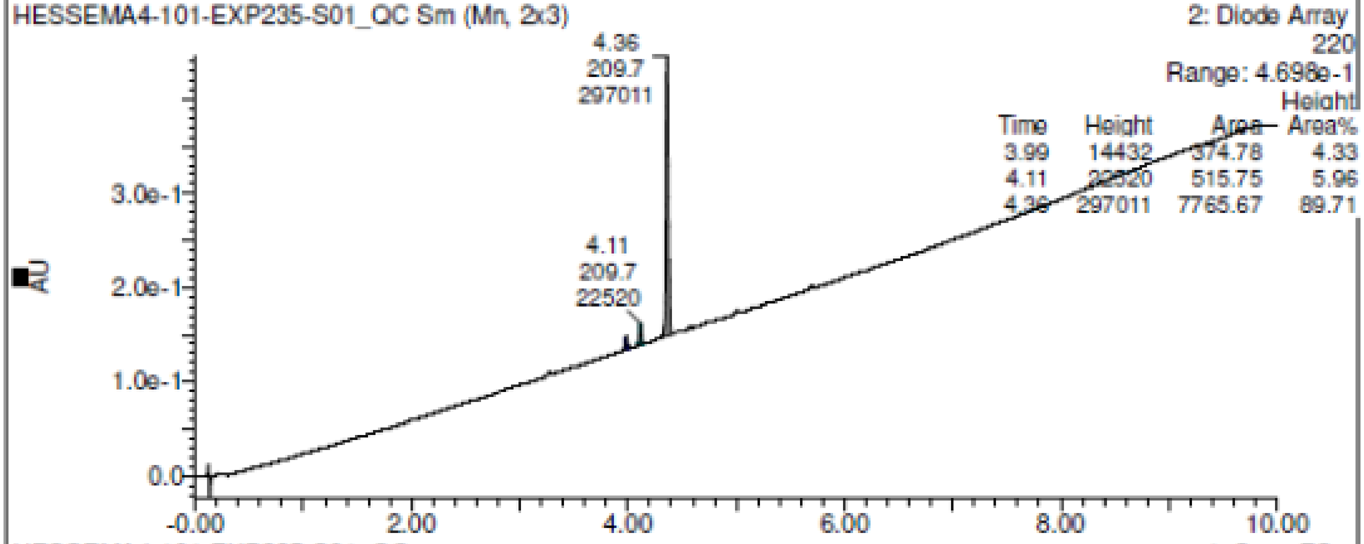

Time	Height	Area	Area%
3.99	14432	374.78	4.33
4.11	22520	515.75	5.96
4.36	297011	7765.67	89.71

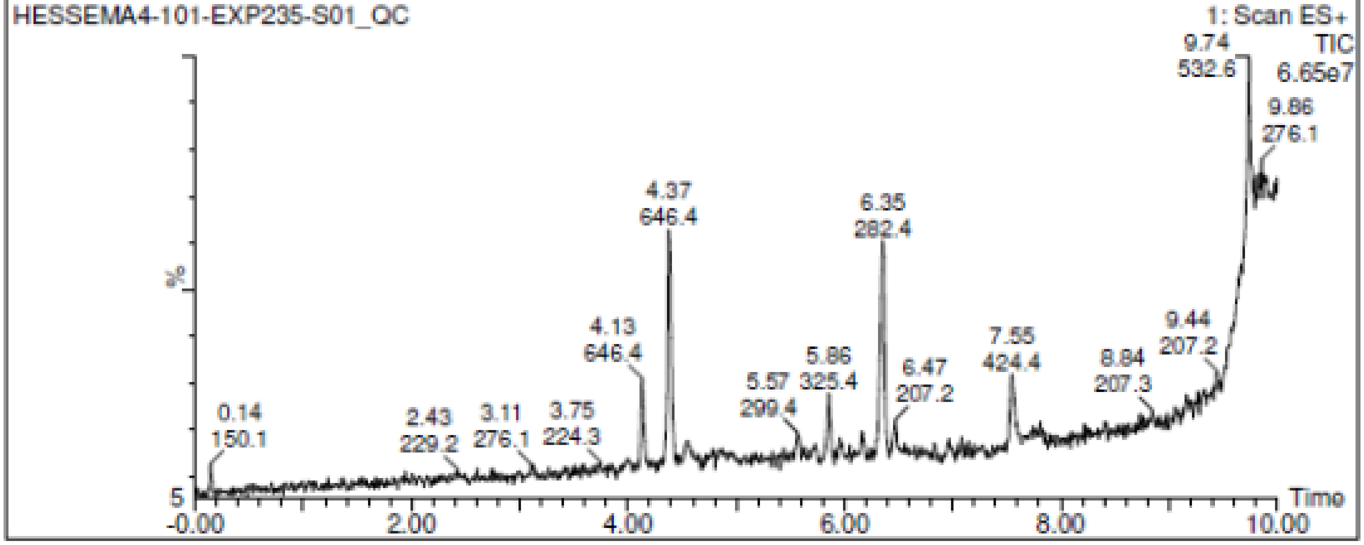

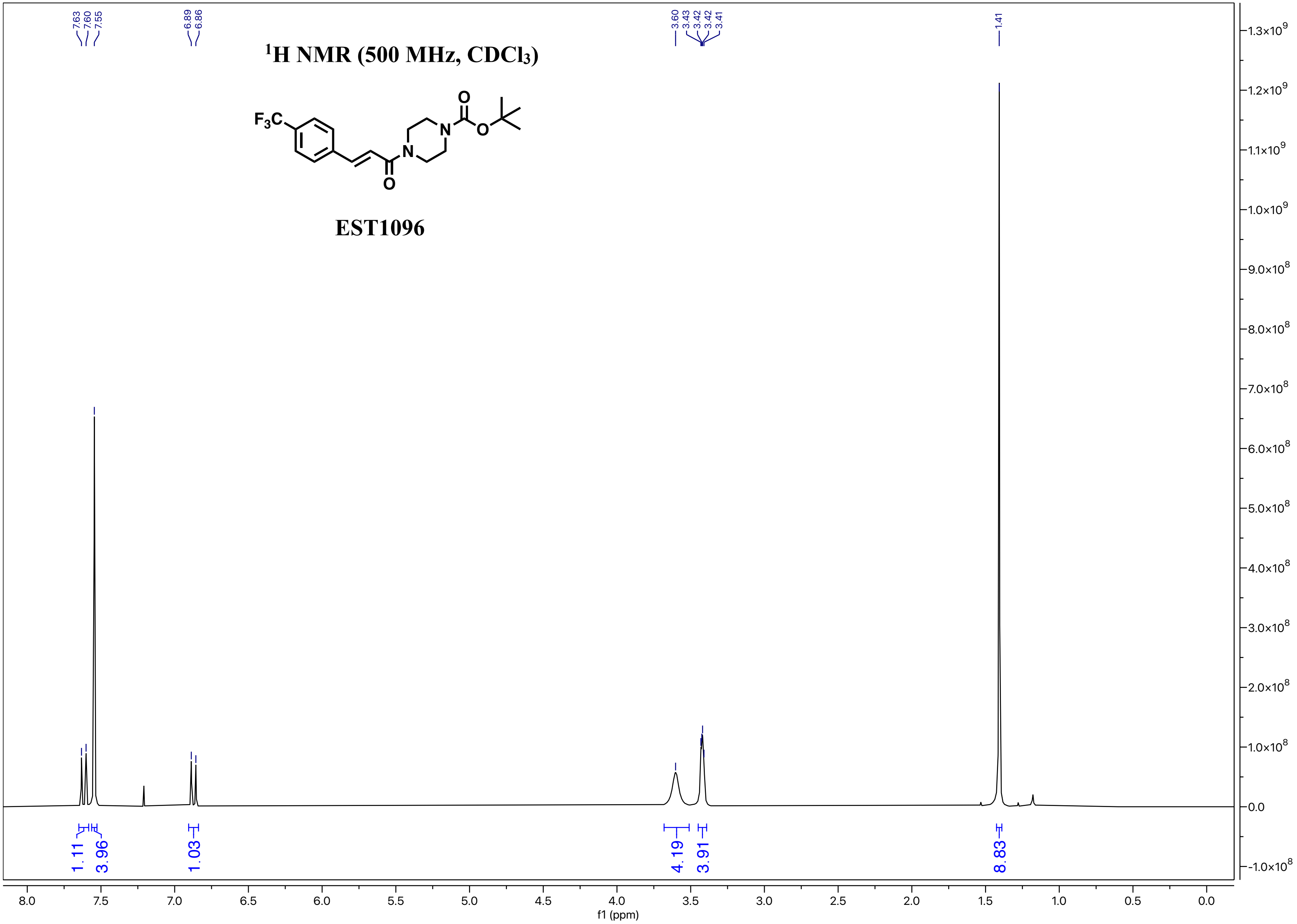

7.63
7.60
7.55
6.89
6.86
3.60
3.43
3.42
3.42
3.41
1.41
¹H NMR (500 MHz, CDCl₃)
F3C
N
N
O
O
O
EST1096
1.11
3.96
1.03
4.19
3.91
8.83
f1 (ppm)

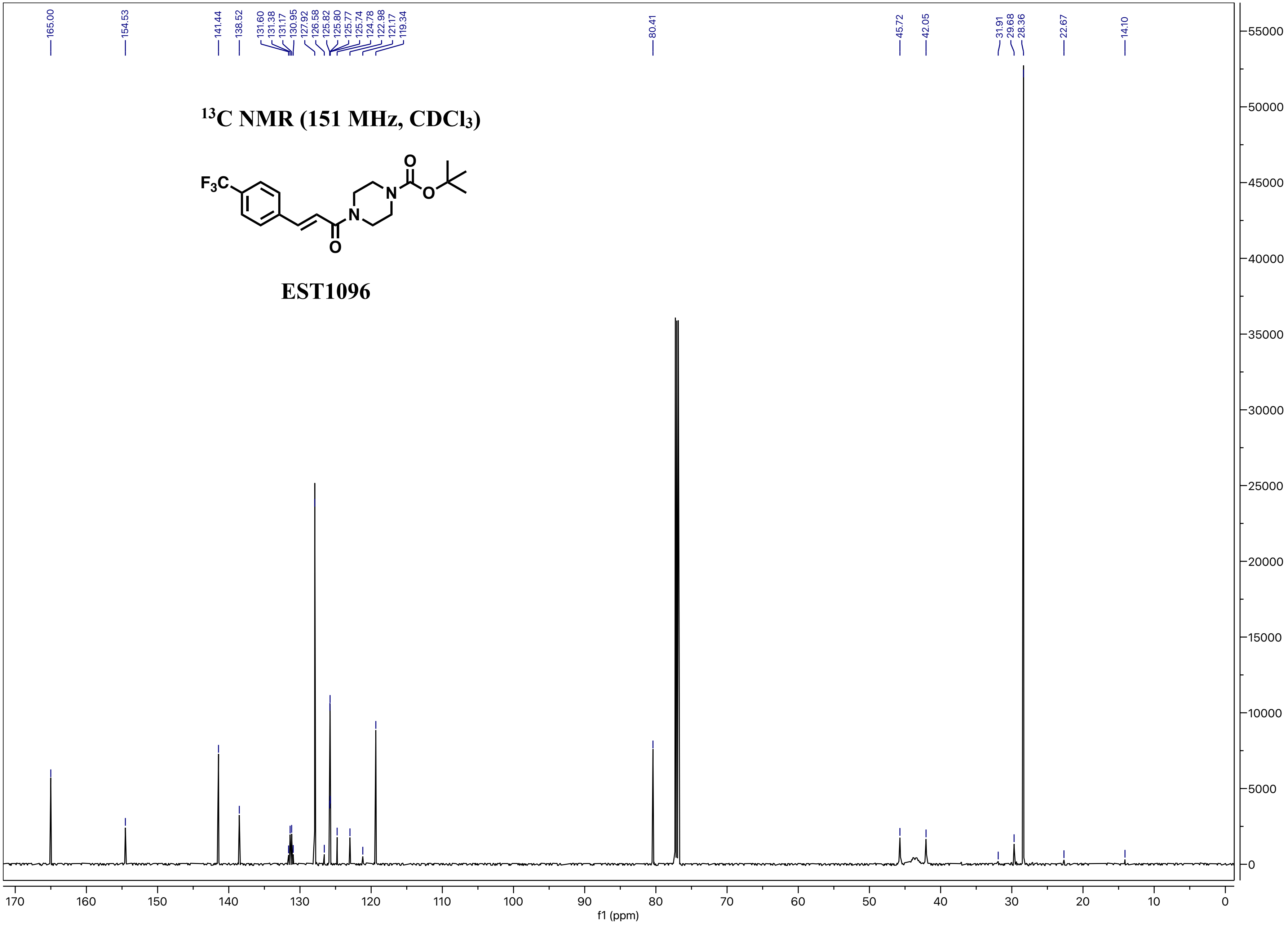
165.00
154.53
141.44
138.52
131.60
131.38
131.17
130.95
127.92
126.58
125.82
125.80
125.77
125.74
124.78
122.98
121.17
119.34
80.41
45.72
42.05
31.91
29.68
28.36
22.67
14.10
^{13}C NMR (151 MHz, $CDCl_3$)
F_3C
O
N
O
N
O
EST1096
f1 (ppm)

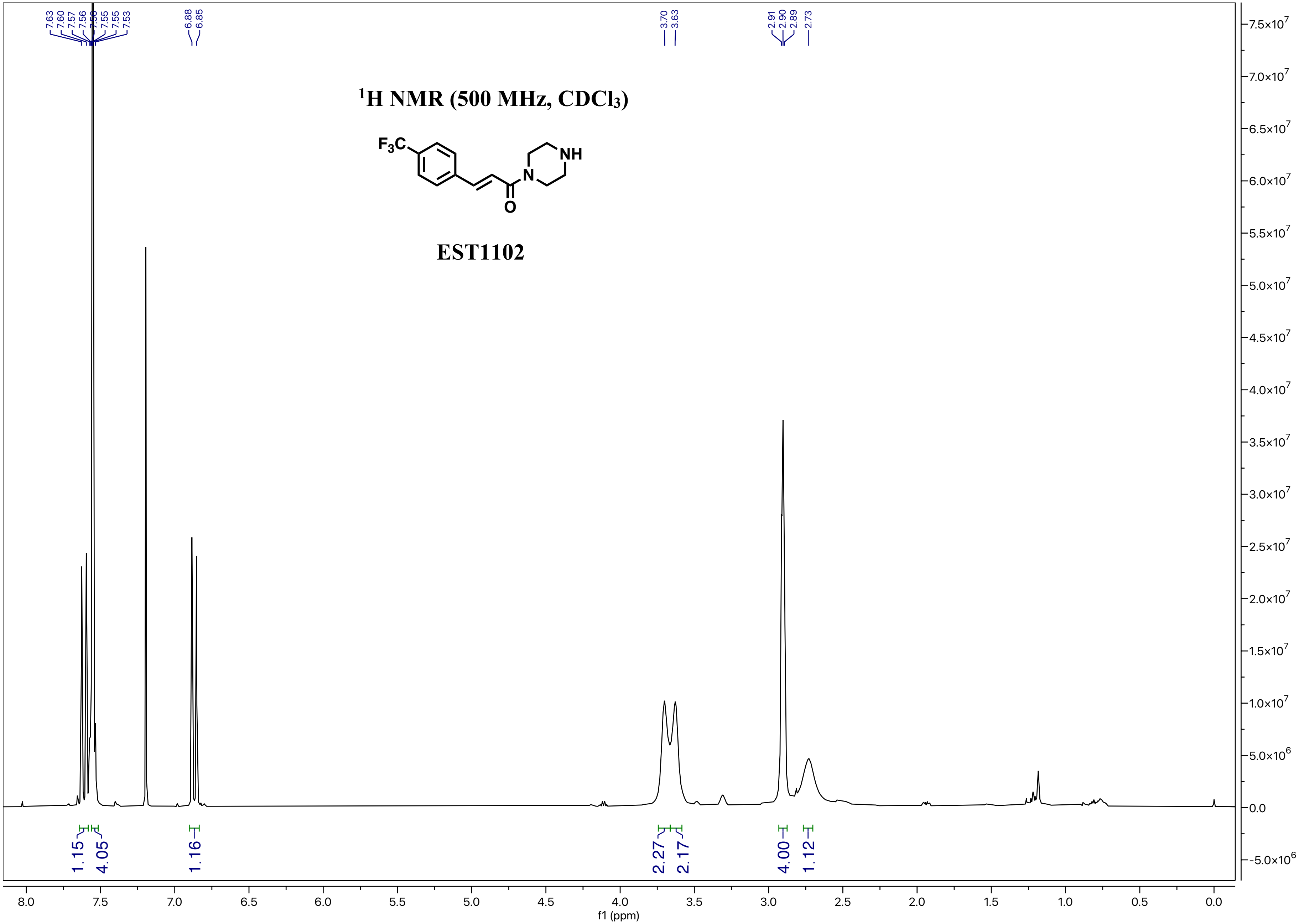

¹H NMR (500 MHz, CDCl₃)
F₃C
NH
N
O
EST1102
7.63
7.60
7.57
7.56
7.56
7.55
7.55
7.53
6.88
6.85
3.70
3.63
2.91
2.90
2.89
2.73
1.15
4.05
1.16
2.27
2.17
4.00
1.12
f1 (ppm)

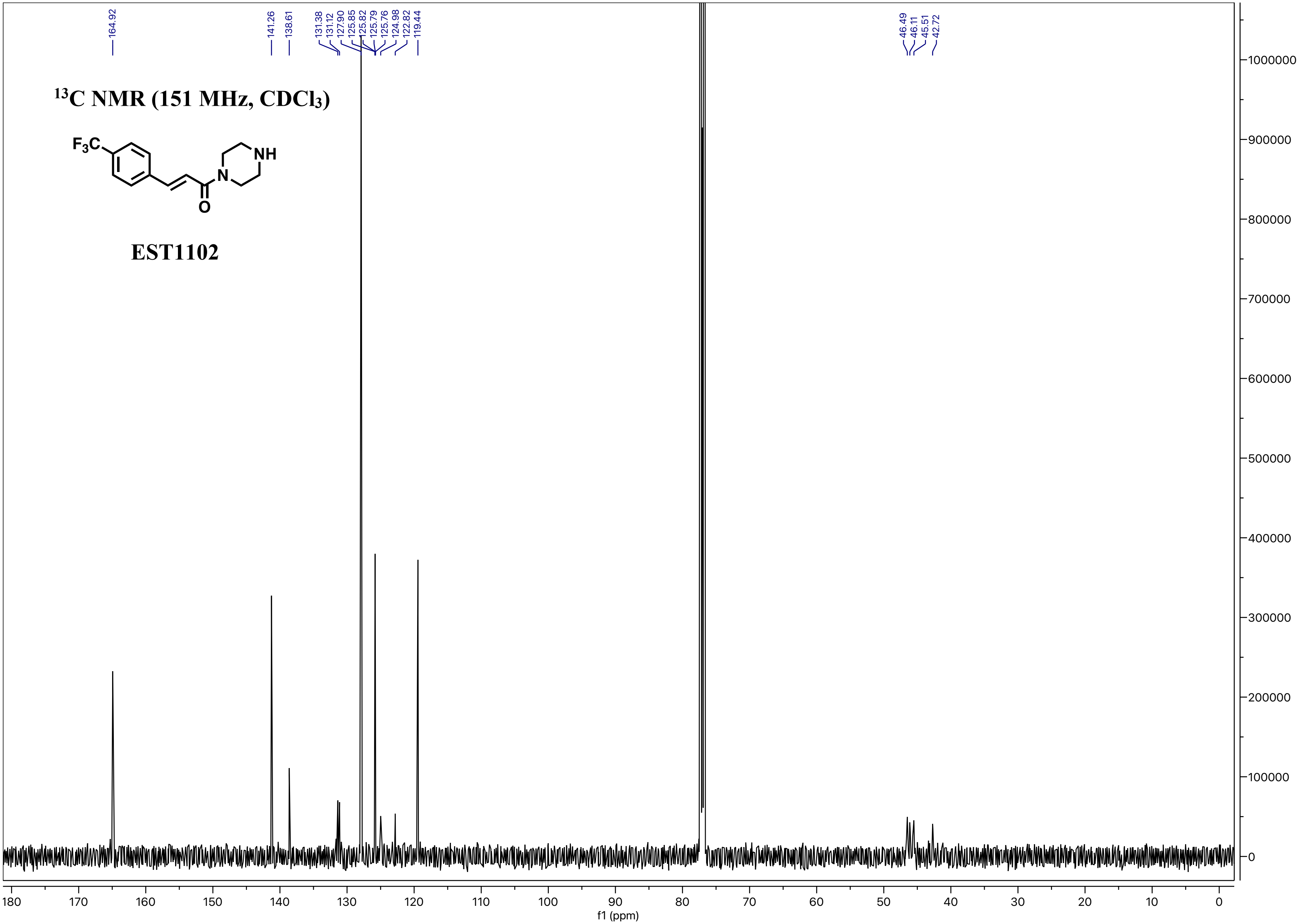

^{13}C NMR (151 MHz, CDCl$_3$)
EST1102
164.92
141.26
138.61
131.38
131.12
127.90
125.85
125.82
125.79
125.76
124.98
122.82
119.44
46.49
46.11
45.51
42.72
f1 (ppm)

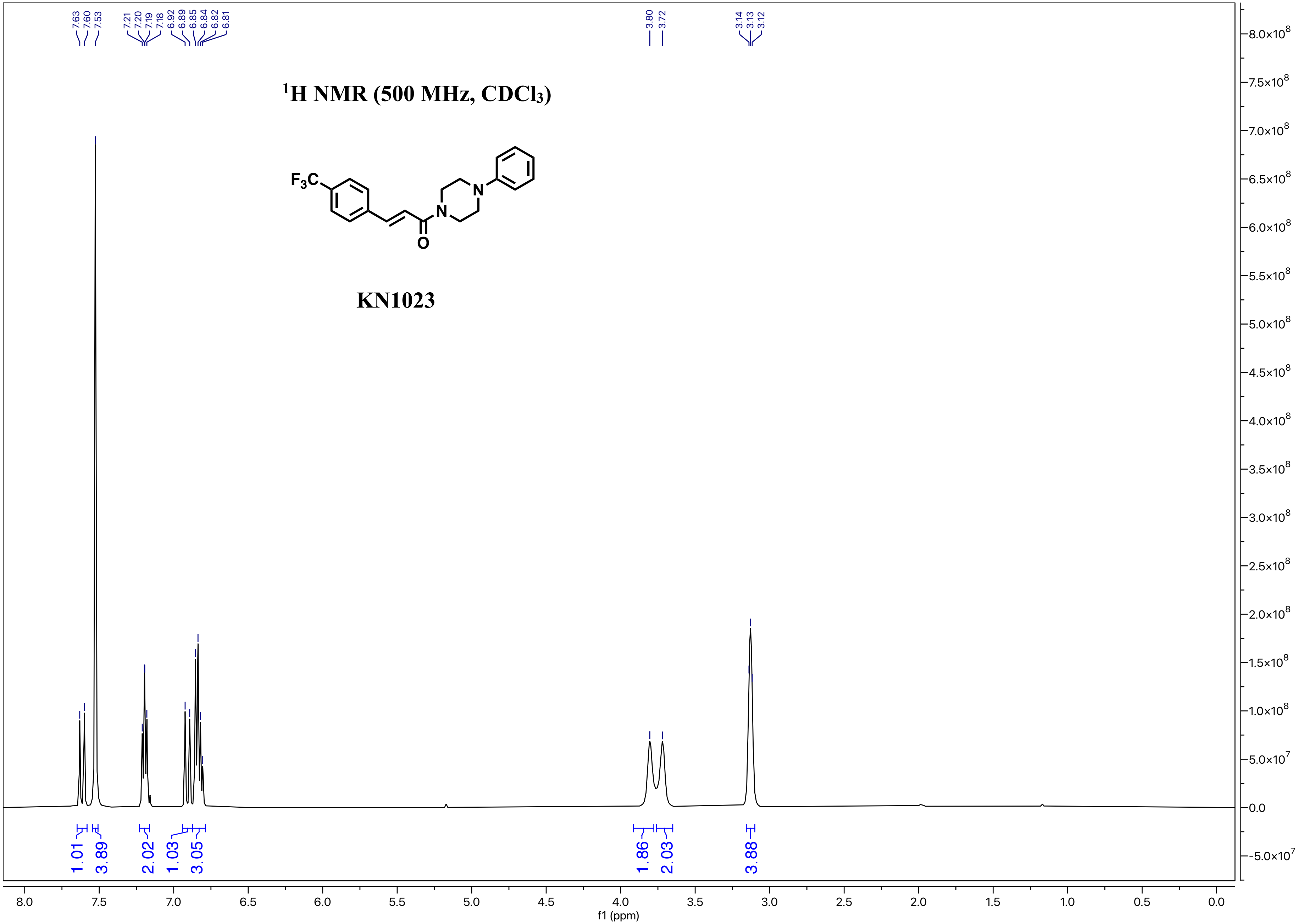

¹H NMR (500 MHz, CDCl3)
KN1023
F3C
N
N
O
7.63
7.60
7.53
7.21
7.20
7.19
7.18
6.92
6.89
6.85
6.84
6.82
6.81
3.80
3.72
3.14
3.13
3.12
1.01
3.89
2.02
1.03
3.05
1.86
2.03
3.88
f1 (ppm)

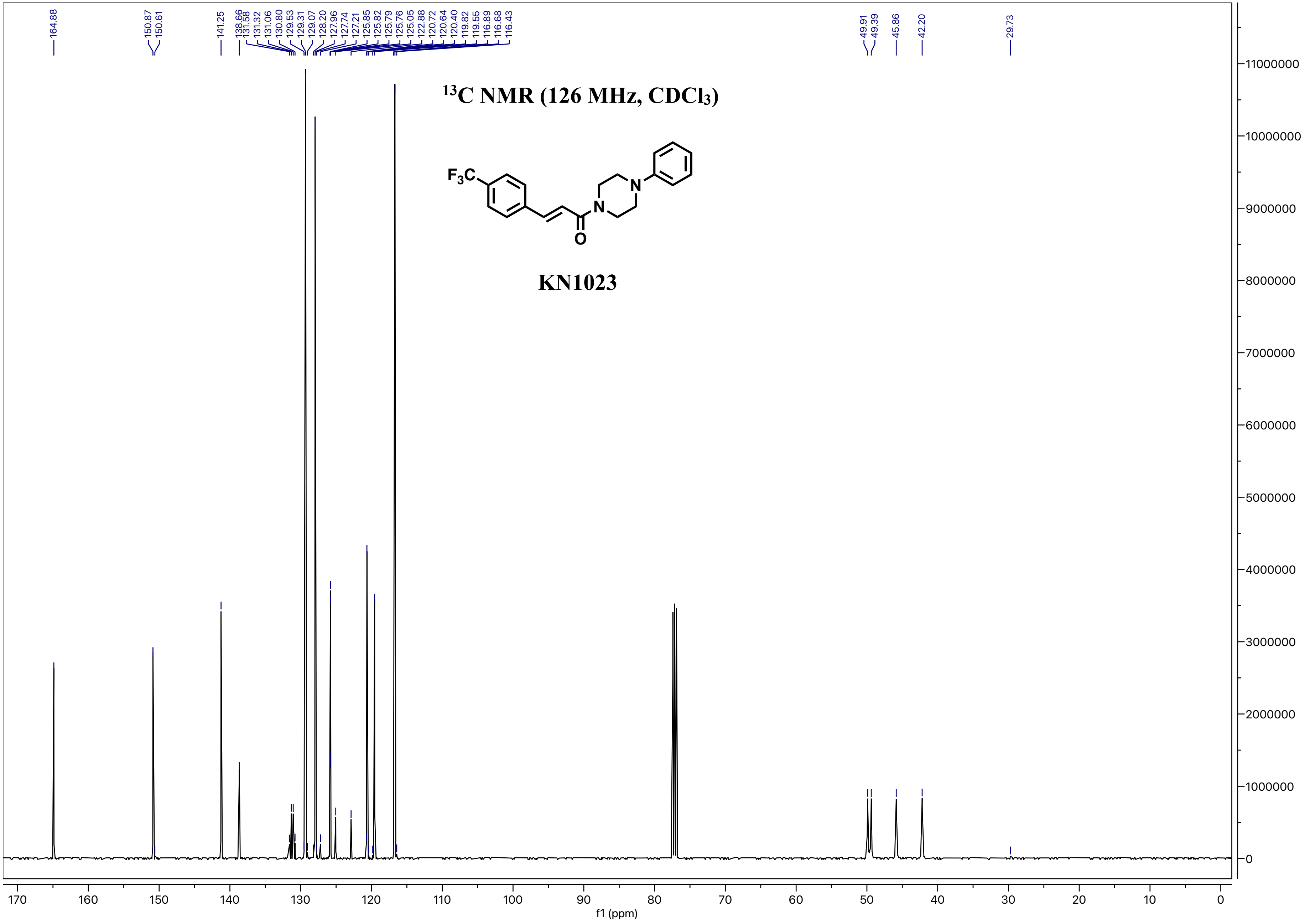

13C NMR (126 MHz, CDCl3)
F3C
N
N
O
KN1023
164.88
150.87
150.61
141.25
138.66
131.58
131.32
131.06
130.80
129.53
129.31
129.07
128.20
127.96
127.74
127.21
125.85
125.82
125.79
125.76
125.05
122.88
120.72
120.64
120.40
119.82
119.55
116.89
116.68
116.43
49.91
49.39
45.86
42.20
29.73
f1 (ppm)

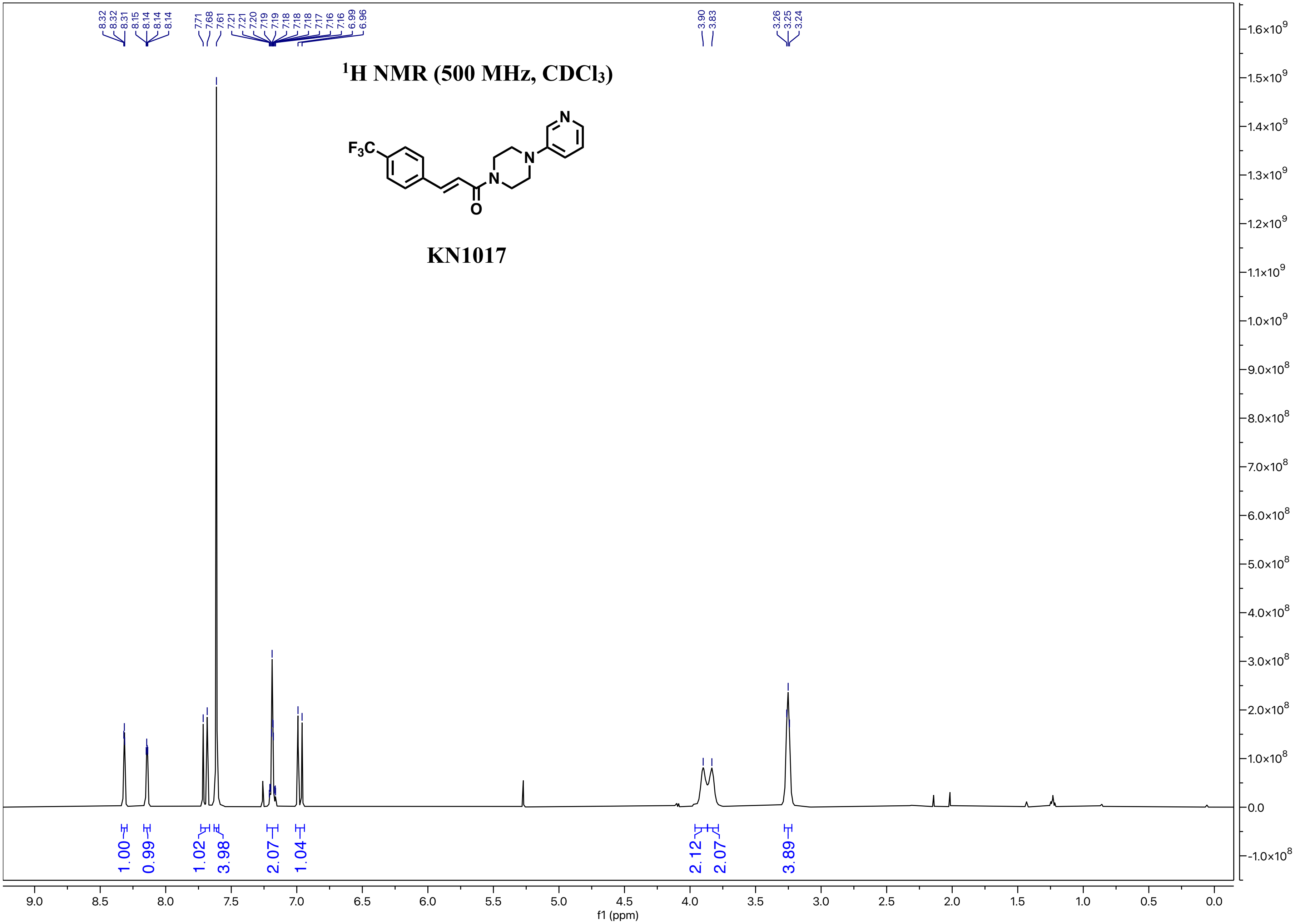

KN1017

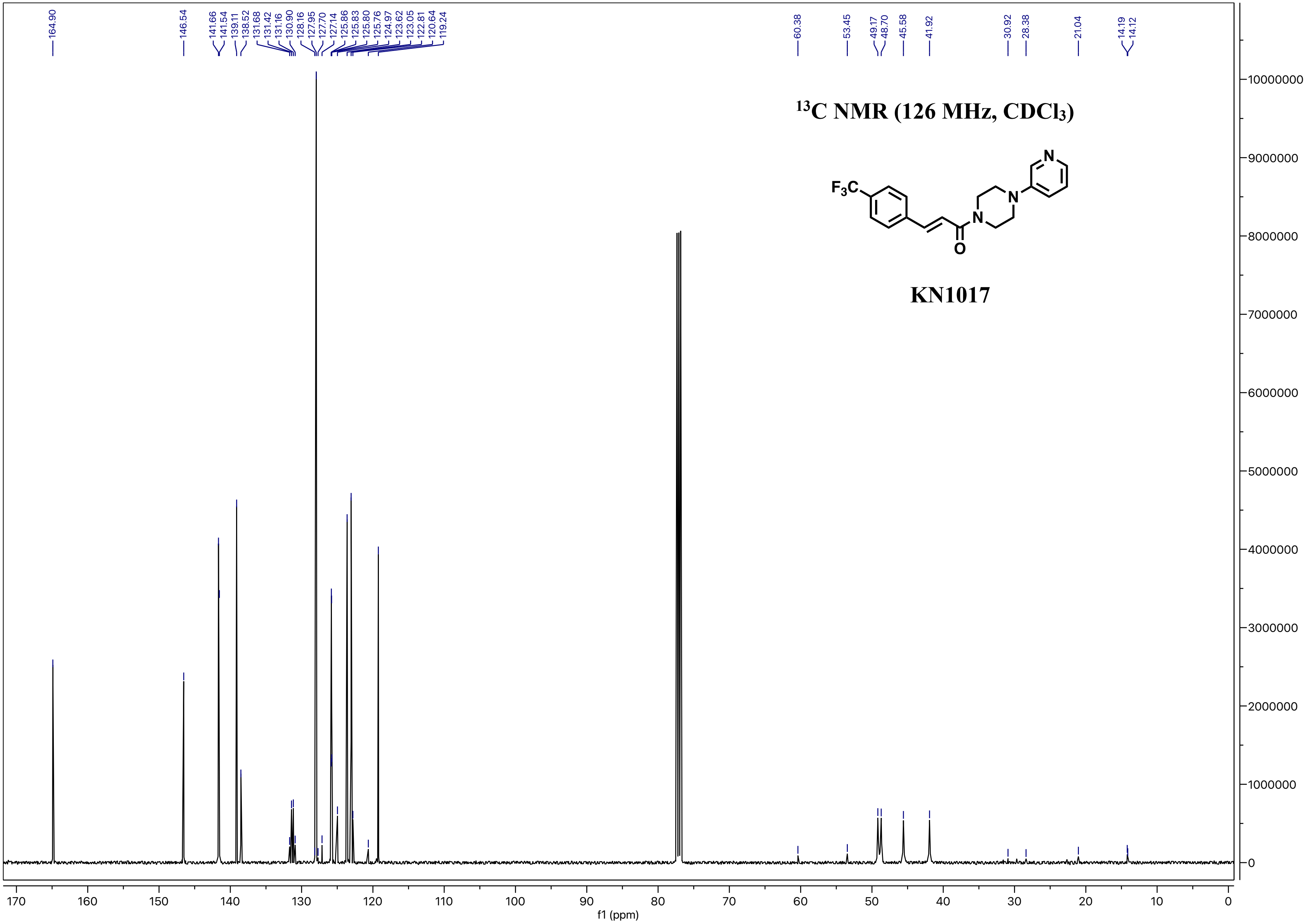

164.90
146.54
141.66
141.54
139.11
138.52
131.68
131.42
131.16
130.90
128.16
127.95
127.70
127.14
125.86
125.83
125.80
125.76
124.97
123.62
123.05
122.81
120.64
119.24
60.38
53.45
49.17
48.70
45.58
41.92
30.92
28.38
21.04
14.19
14.12
13C NMR (126 MHz, CDCl3)
F3C
N
N
N
O
KN1017
f1 (ppm)

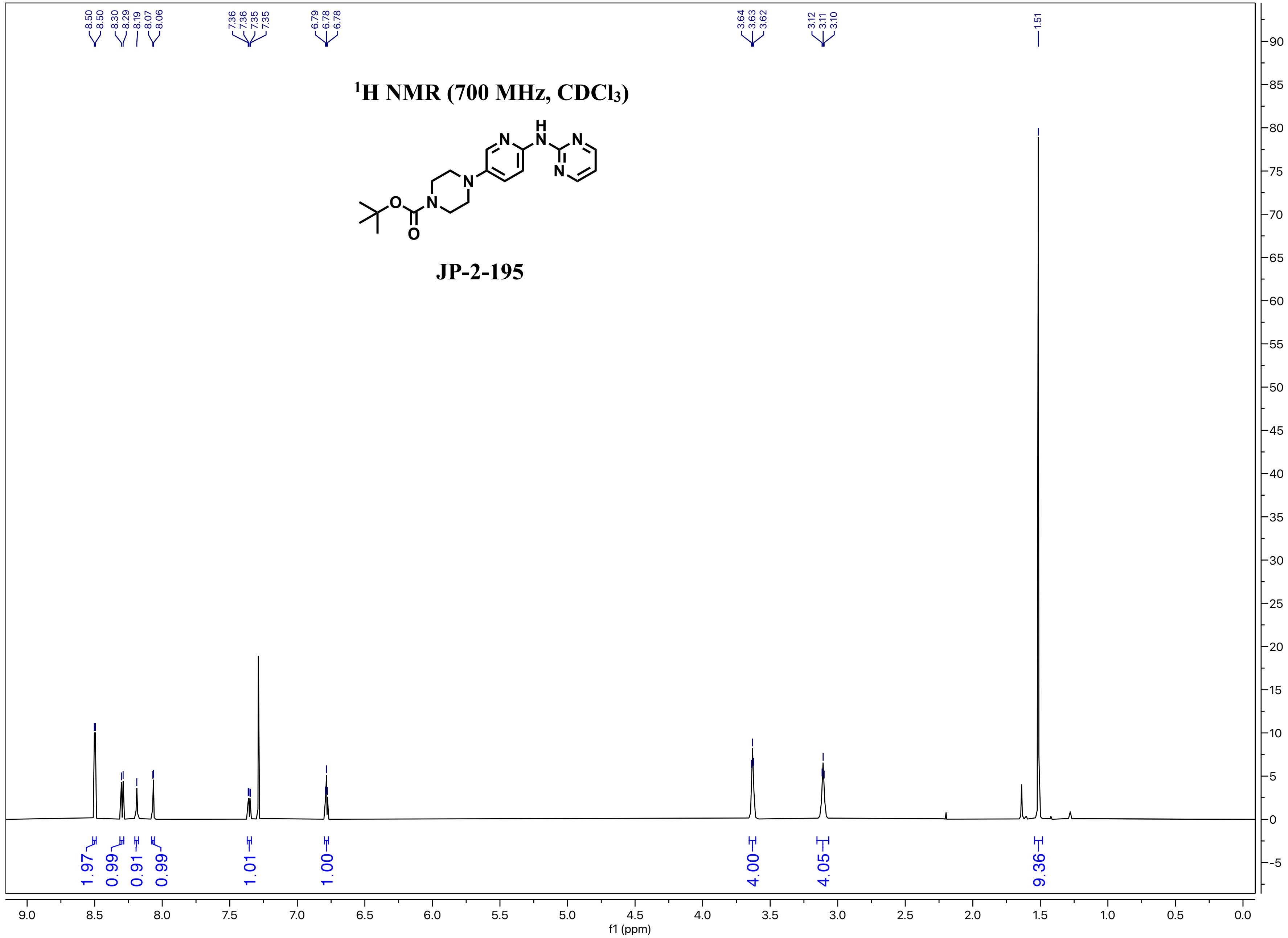
1H NMR (700 MHz, CDCl3)
JP-2-195
8.50
8.50
8.30
8.29
8.19
8.07
8.06
7.36
7.36
7.35
7.35
6.79
6.78
6.78
3.64
3.63
3.62
3.12
3.11
3.10
1.51
1.97
0.99
0.91
0.99
1.01
1.00
4.00
4.05
9.36
f1 (ppm)

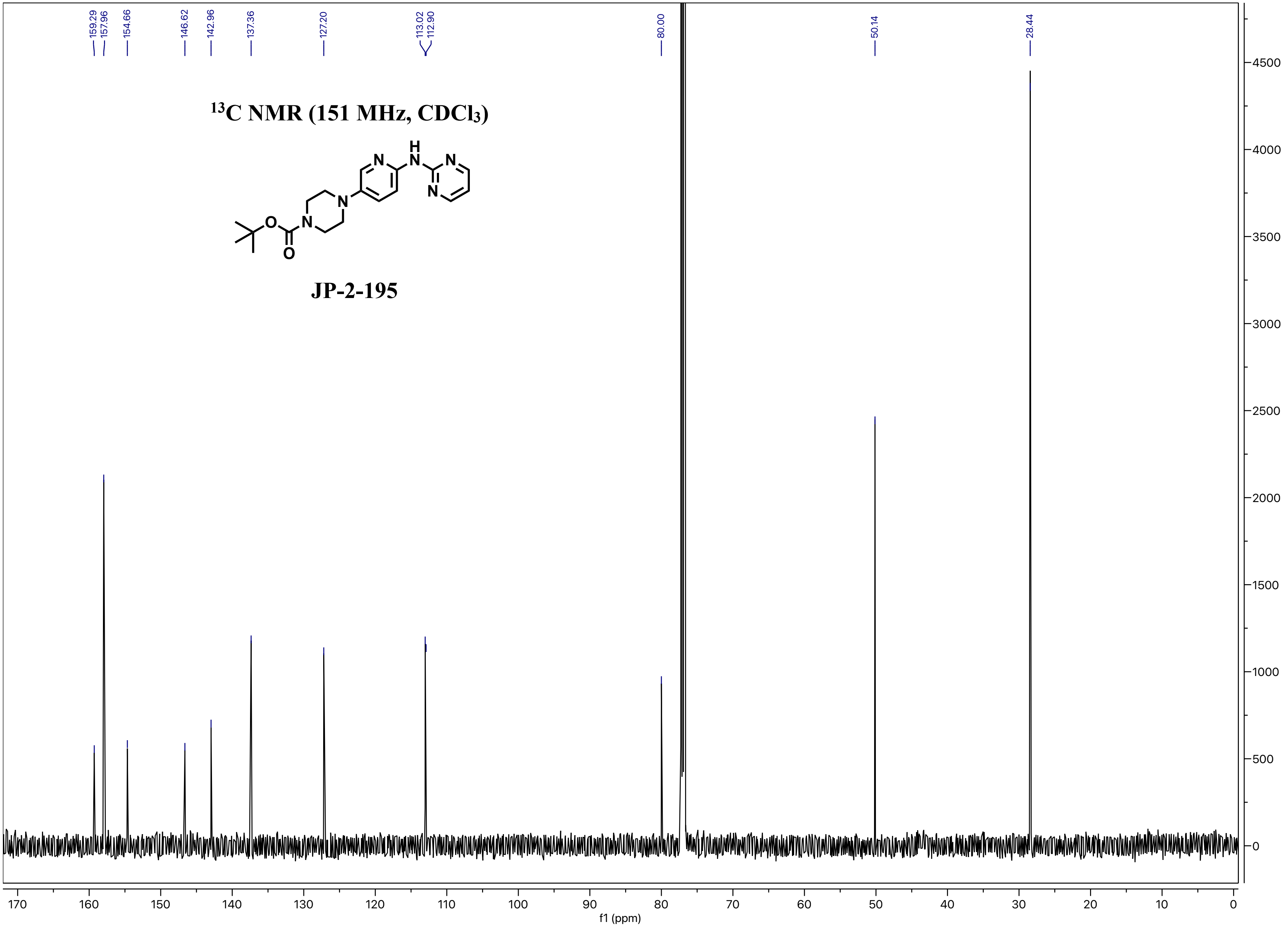
159.29
157.96
154.66
146.62
142.96
137.36
127.20
113.02
112.90
80.00
50.14
28.44
13C NMR (151 MHz, CDCl3)
JP-2-195
4500
4000
3500
3000
2500
2000
1500
1000
500
0
170
160
150
140
130
120
110
100
90
80
70
60
50
40
30
20
10
0
f1 (ppm)

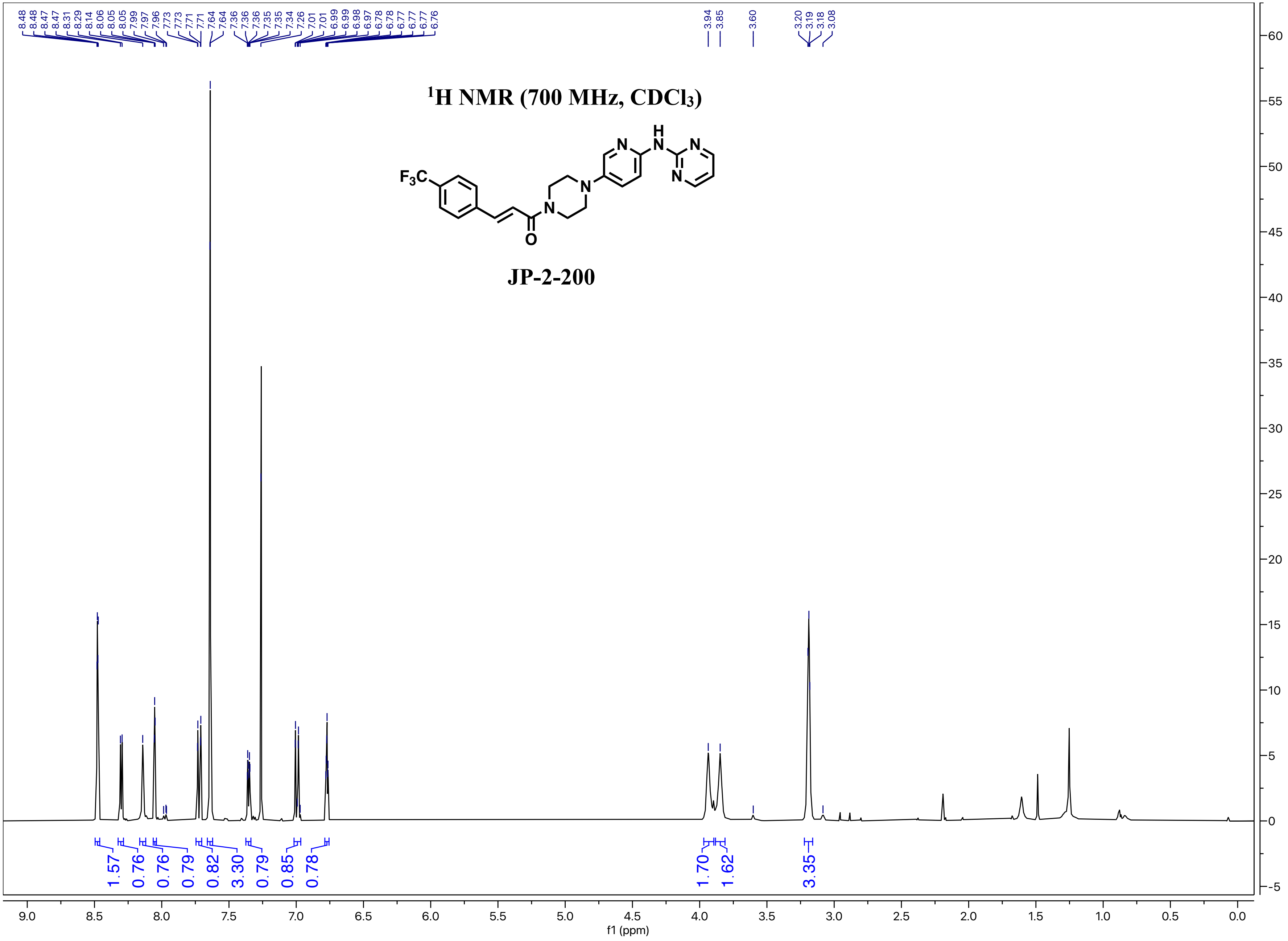

^{1}H NMR (700 MHz, CDCl$_3$)
JP-2-200
F3C
N
H
f1 (ppm)
1.57
0.76
0.76
0.79
0.82
3.30
0.79
0.85
0.78
1.70
1.62
3.35

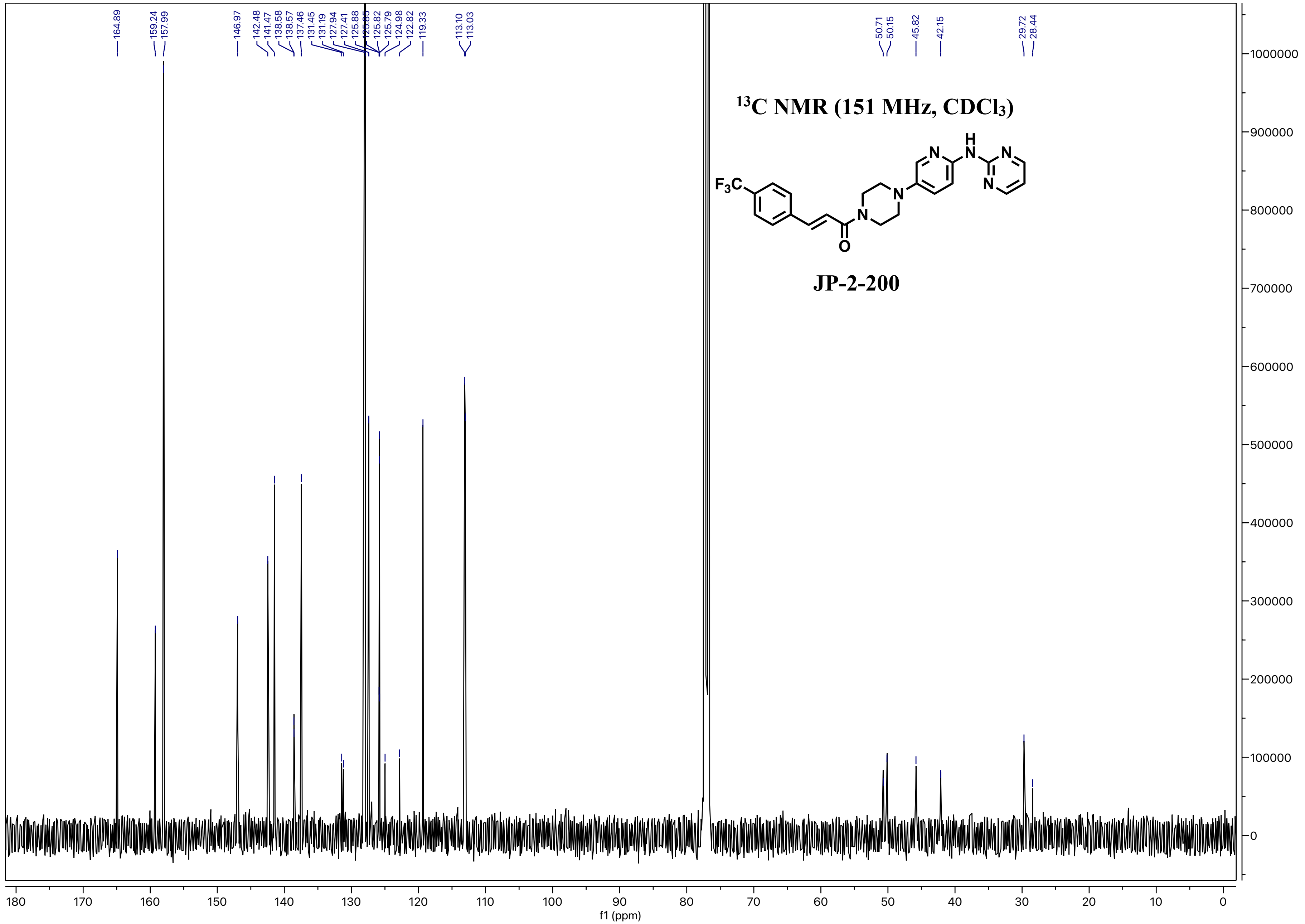
164.89
159.24
157.99
146.97
142.48
141.47
138.58
138.57
137.46
131.45
131.19
127.94
127.41
125.88
125.85
125.82
125.79
124.98
122.82
119.33
113.10
113.03
50.71
50.15
45.82
42.15
29.72
28.44
13C NMR (151 MHz, CDCl3)
F3C
N
H
JP-2-200
1000000
900000
800000
700000
600000
500000
400000
300000
200000
100000
0
180 170 160 150 140 130 120 110 100 90 80 70 60 50 40 30 20 10 0
f1 (ppm)

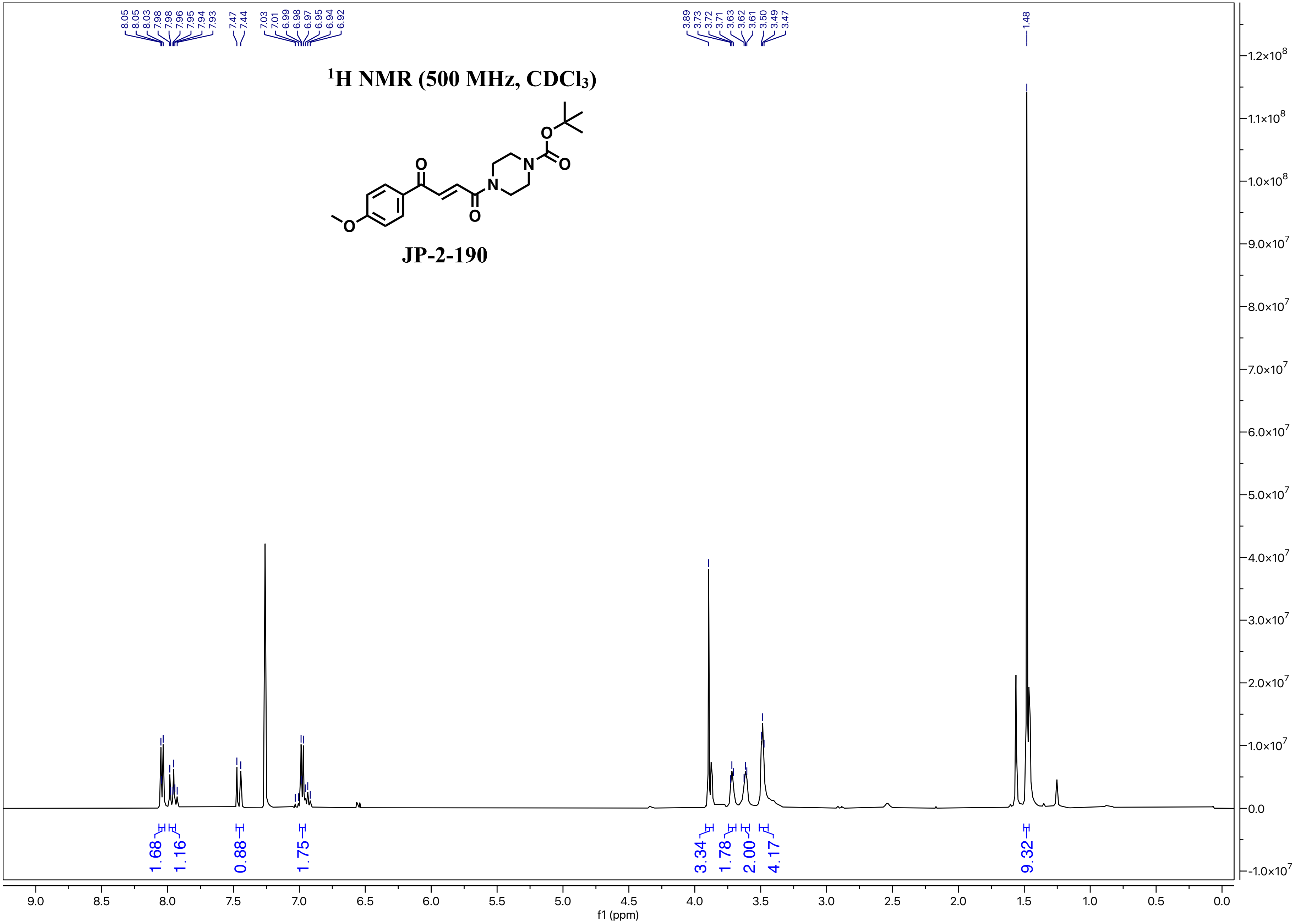

¹H NMR (500 MHz, CDCl₃)
JP-2-190
f1 (ppm)

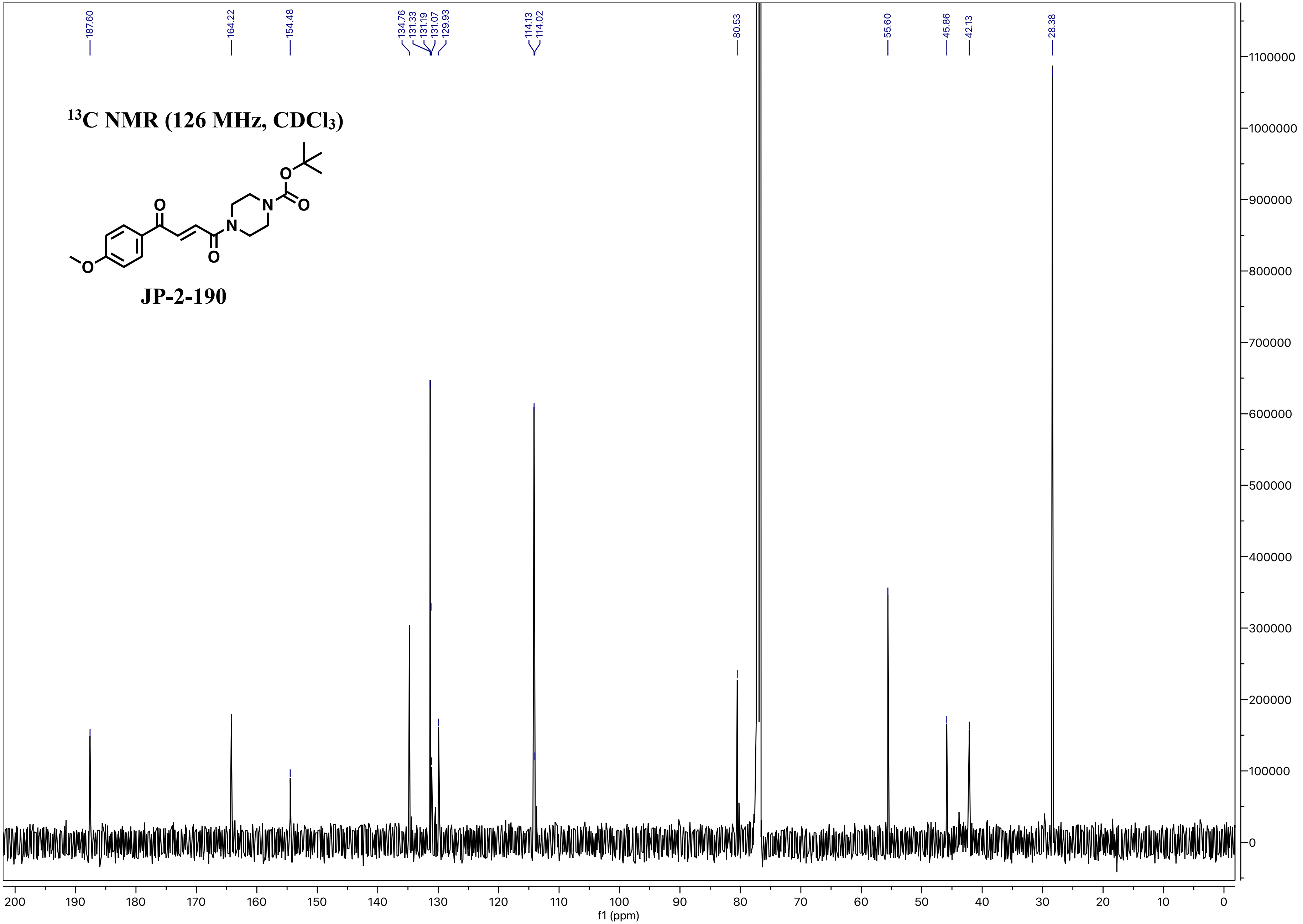

187.60
164.22
154.48
134.76
131.33
131.19
131.07
129.93
114.13
114.02
80.53
55.60
45.86
42.13
28.38
^{13}C NMR (126 MHz, CDCl$_3$)
JP-2-190
f1 (ppm)

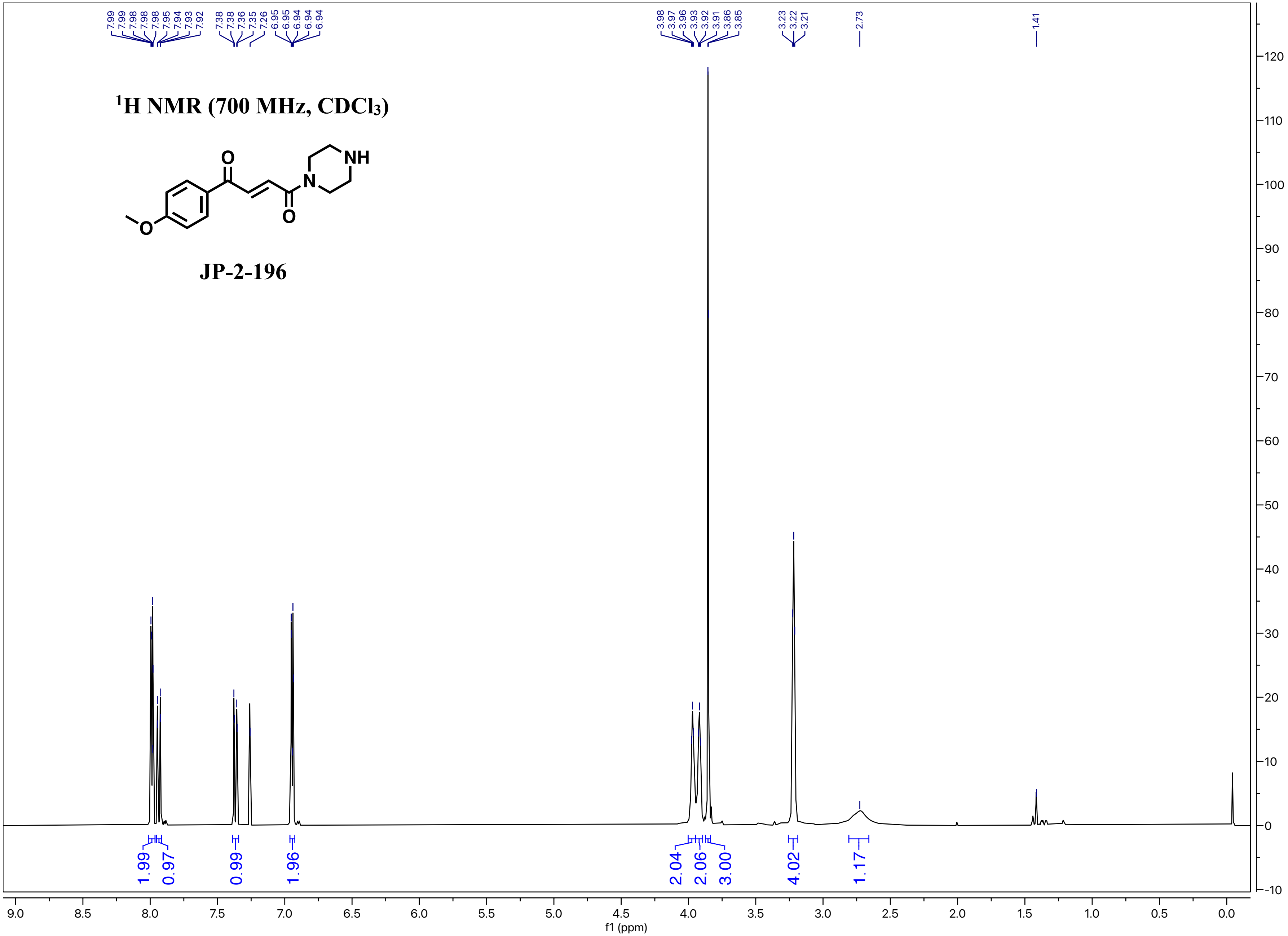

7.99
7.99
7.98
7.98
7.98
7.95
7.94
7.93
7.92
7.38
7.38
7.36
7.35
7.26
6.95
6.95
6.94
6.94
6.94
3.98
3.97
3.96
3.93
3.92
3.91
3.86
3.85
3.23
3.22
3.21
2.73
1.41
^{1}H NMR (700 MHz, CDCl$_3$)
O
NH
N
O
O
JP-2-196
1.99
0.97
0.99
1.96
2.04
2.06
3.00
4.02
1.17
f1 (ppm)

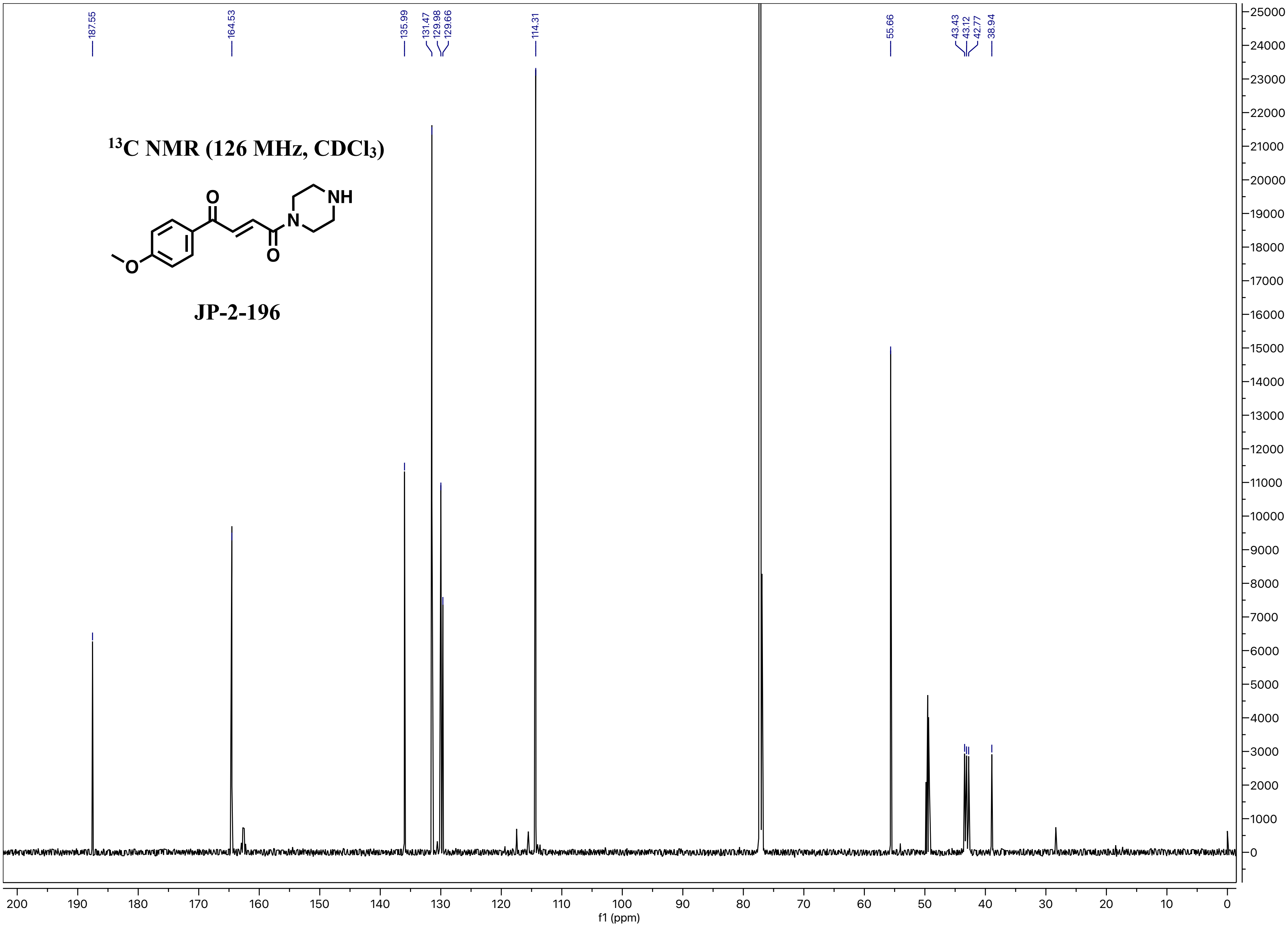

187.55
164.53
135.99
131.47
129.98
129.66
114.31
55.66
43.43
43.12
42.77
38.94
^{13}C NMR (126 MHz, $CDCl_3$)
O
O
N
NH
O
JP-2-196
f1 (ppm)

HPLC=0.1%TFA in H2O/ACN with 1.2mL/min

EM23001594446 | **08-Feb-2023 17:28:17 strubhe1-0208A-v21** | **LCMS-ZQ1 Kinetex column, ZQ1_10m_Acidic**

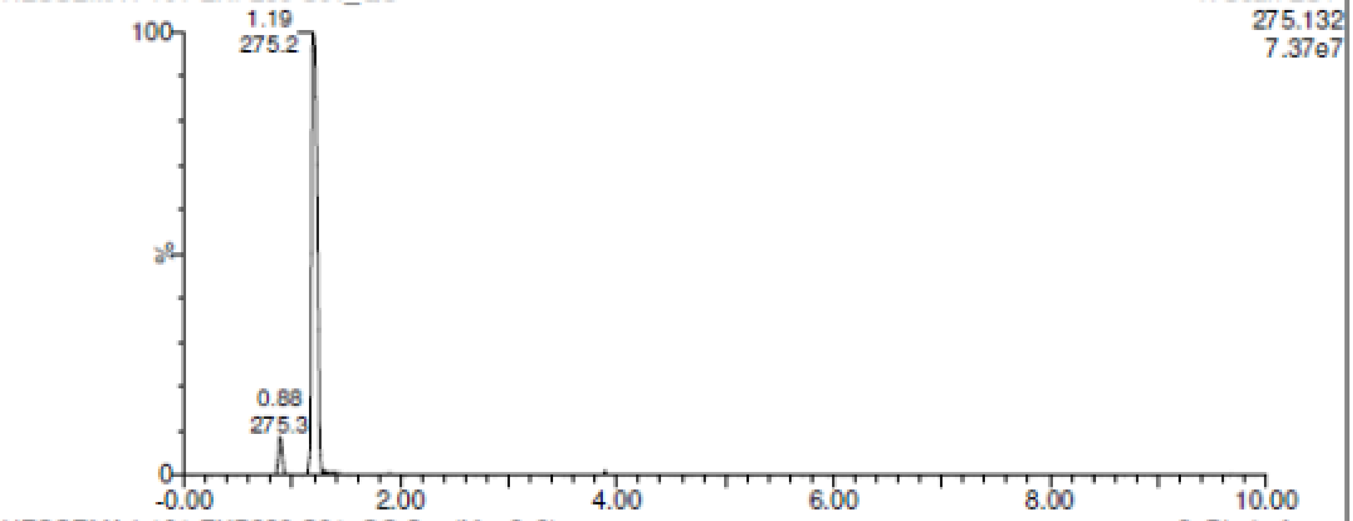

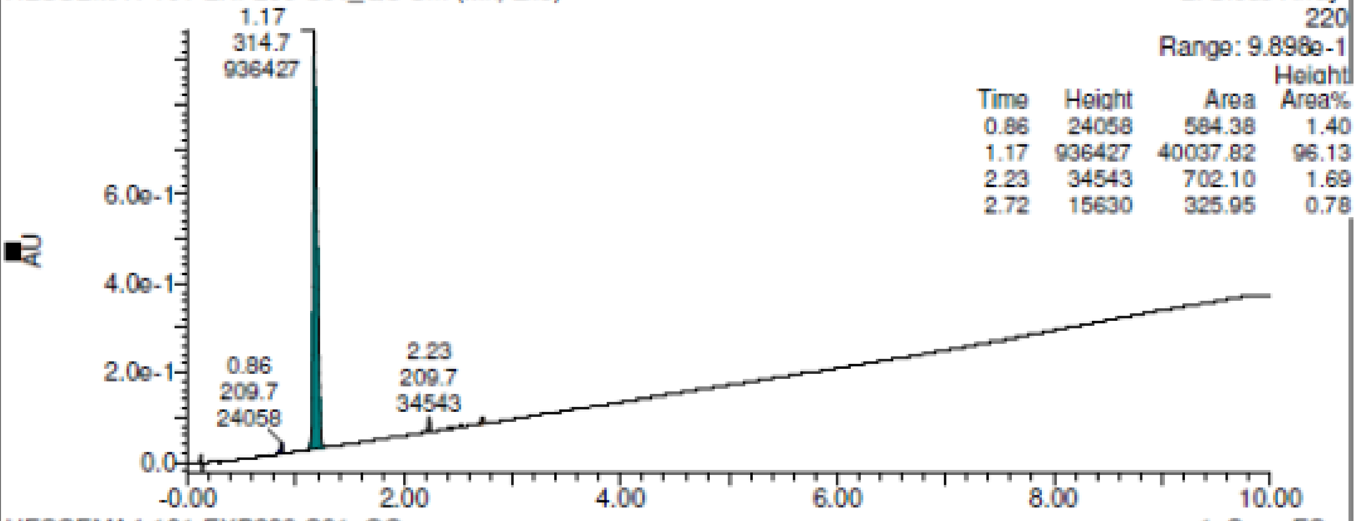

Time	Height	Area	Height Area%
0.86	24058	584.38	1.40
1.17	936427	40037.82	96.13
2.23	34543	702.10	1.69
2.72	15630	325.95	0.78

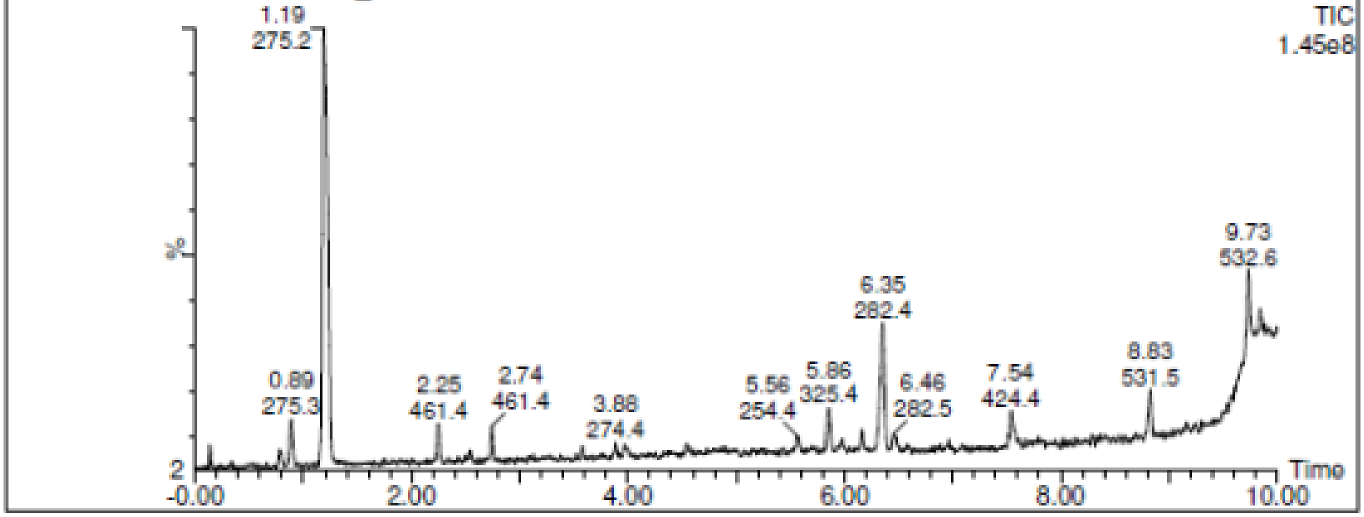

HPLC=0.1%TFA in H2O/ACN with 1.2mL/min

EM23001594446 | **14-Feb-2023 13:28:46 strubhe1-0208A-v21** | **LCMS-ZQ1 Kinetex column, ZQ1_10m_Acidic**

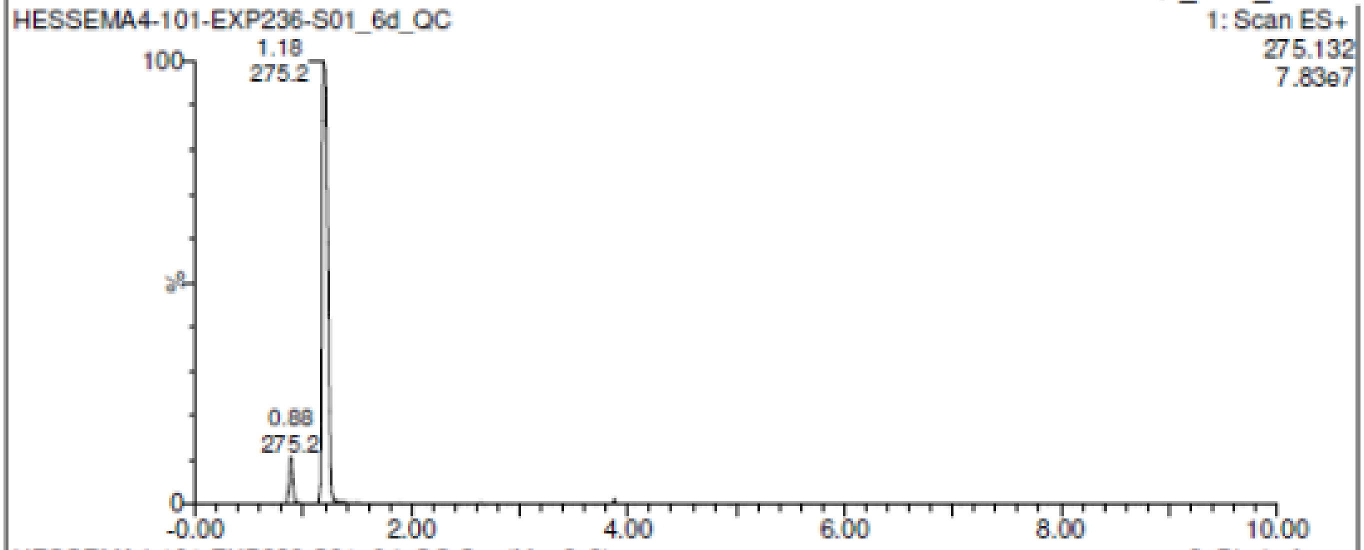

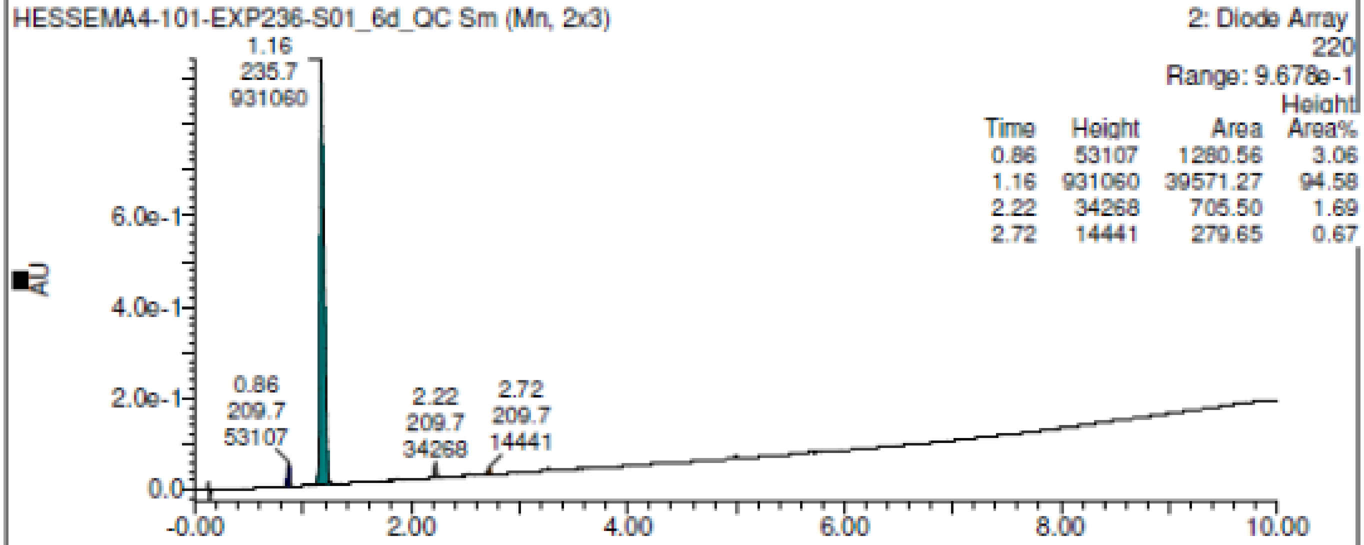

Time	Height	Area	Height Area%
0.86	53107	1280.56	3.06
1.16	931060	39571.27	94.58
2.22	34268	705.50	1.69
2.72	14441	279.65	0.67

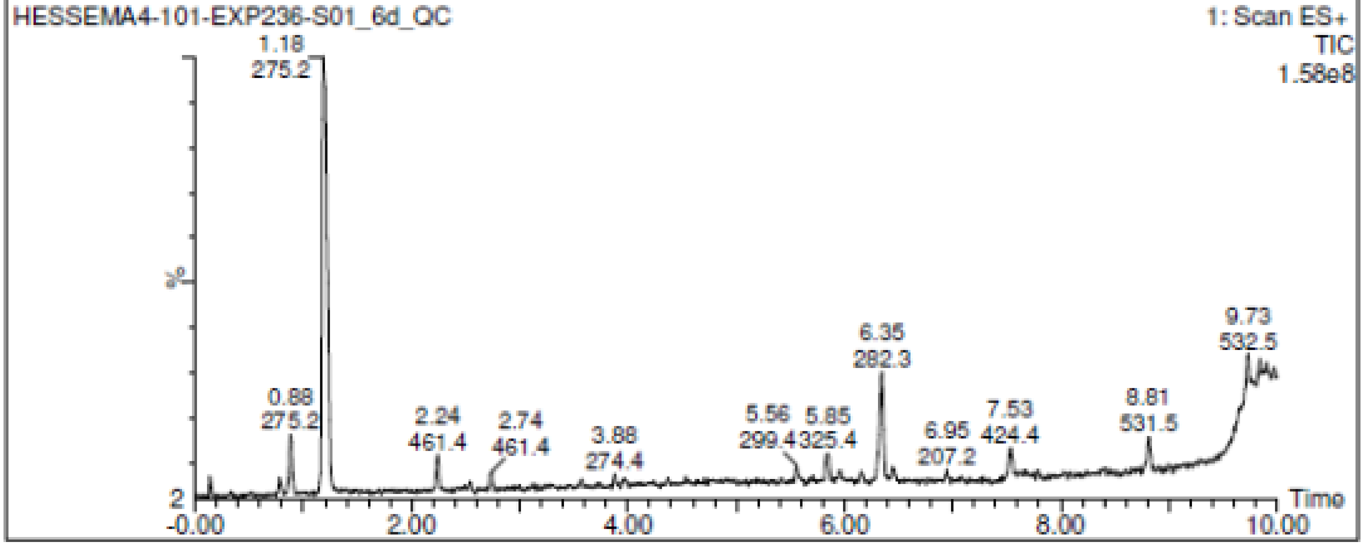

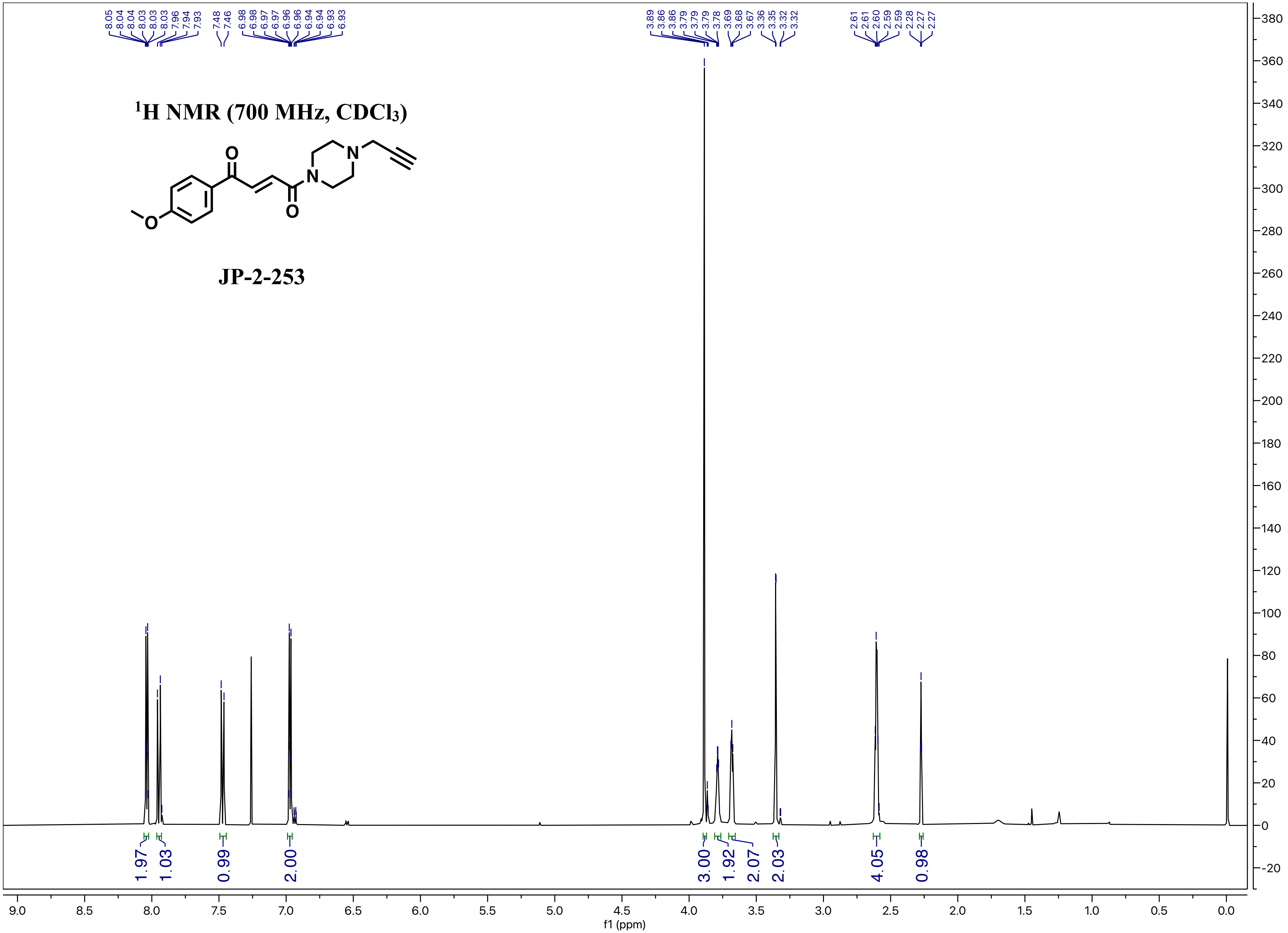

8.05
8.04
8.04
8.03
8.03
8.03
7.96
7.94
7.93
7.48
7.46
6.98
6.98
6.97
6.97
6.96
6.96
6.94
6.94
6.93
6.93
3.89
3.86
3.86
3.79
3.79
3.79
3.78
3.69
3.68
3.67
3.36
3.35
3.32
3.32
2.61
2.61
2.60
2.59
2.59
2.28
2.27
2.27
1H NMR (700 MHz, CDCl3)
O
O
O
N
N
JP-2-253
1.97
1.03
0.99
2.00
3.00
1.92
2.07
2.03
4.05
0.98
f1 (ppm)

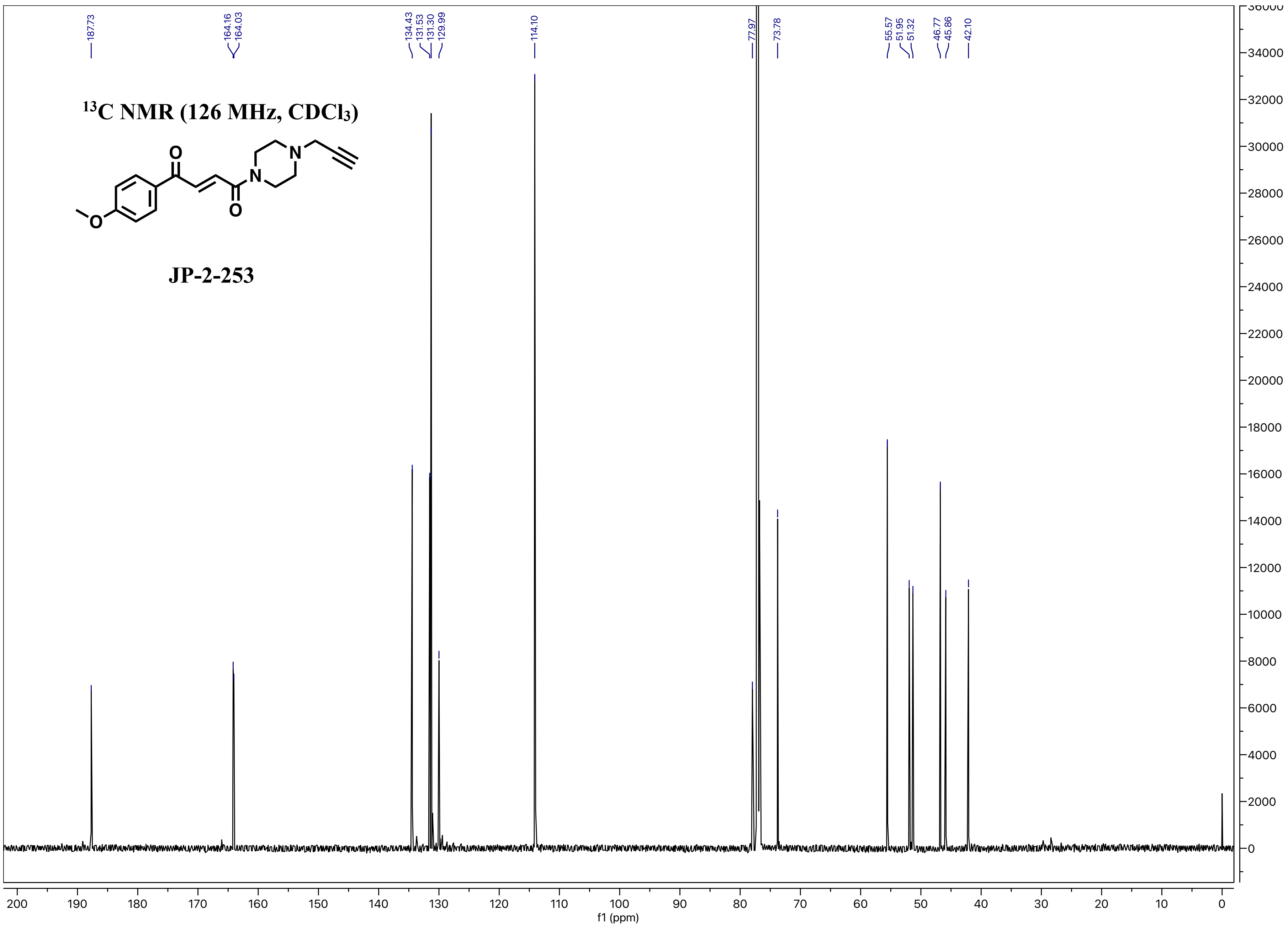
187.73
164.16
164.03
134.43
131.53
131.30
129.99
114.10
77.97
73.78
55.57
51.95
51.32
46.77
45.86
42.10
13C NMR (126 MHz, CDCl3)
JP-2-253
f1 (ppm)

HPLC=0.1%TFA in H2O/ACN with 1.2mL/min

EM23001594453 | 08-Feb-2023 18:01:28 strubhe1-0208A-v24 | LCMS-ZQ1 Kinetex column, ZQ1_10m_Acidic

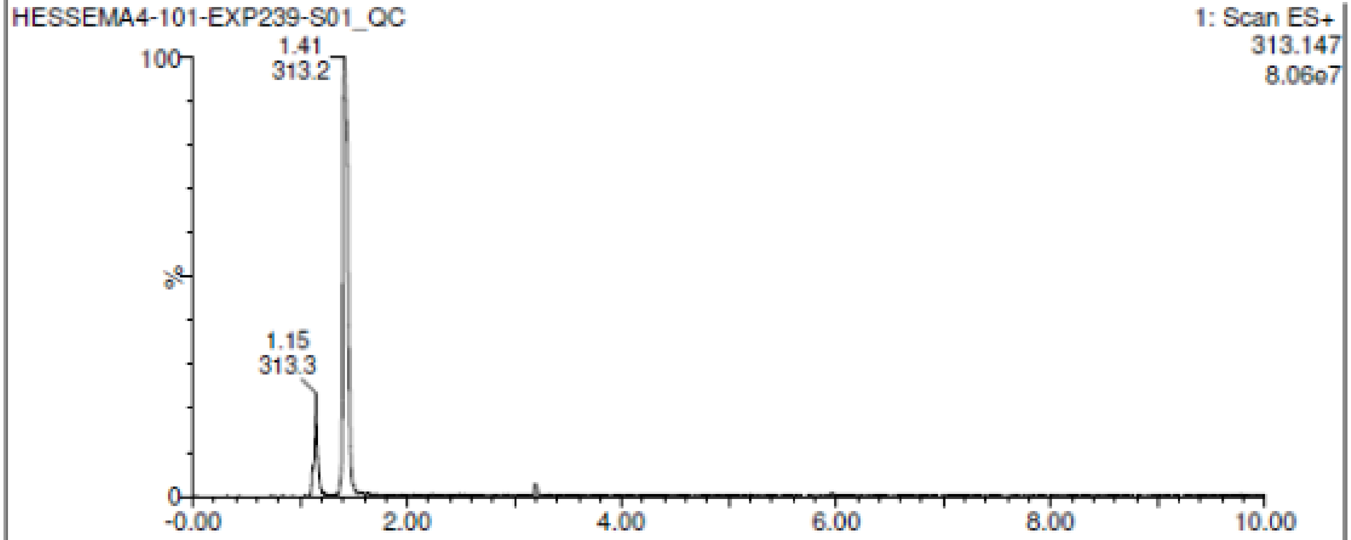

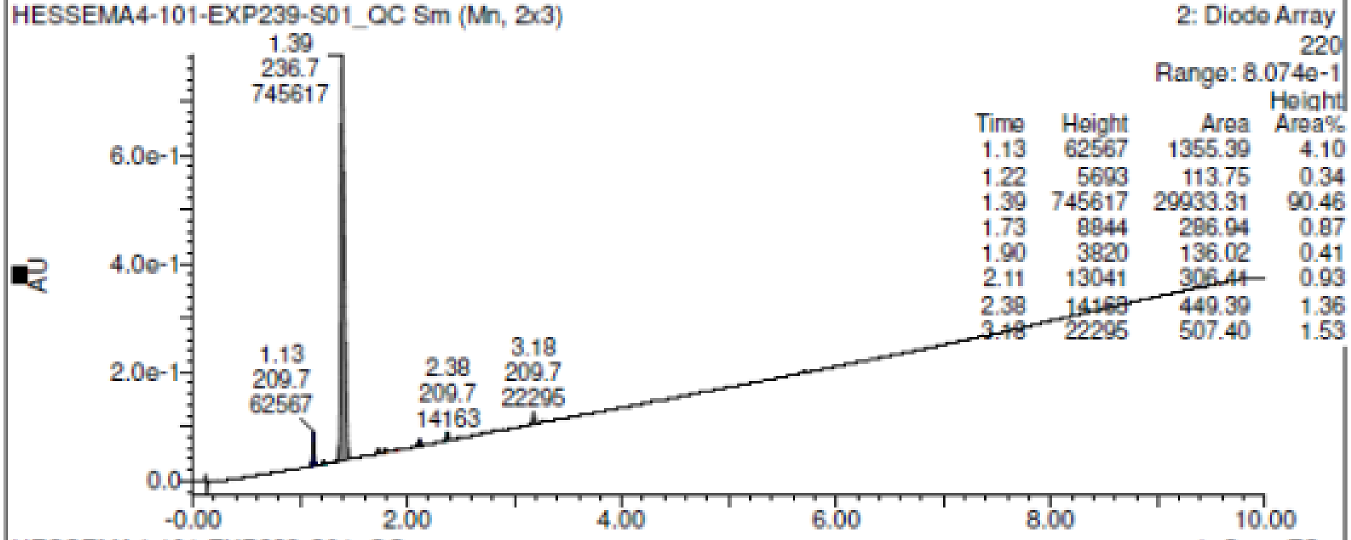

Time	Height	Area	Area%
1.13	62567	1355.39	4.10
1.22	5693	113.75	0.34
1.39	745617	29933.31	90.46
1.73	8844	286.94	0.87
1.90	3820	136.02	0.41
2.11	13041	306.41	0.93
2.38	14163	449.39	1.36
3.18	22295	507.40	1.53

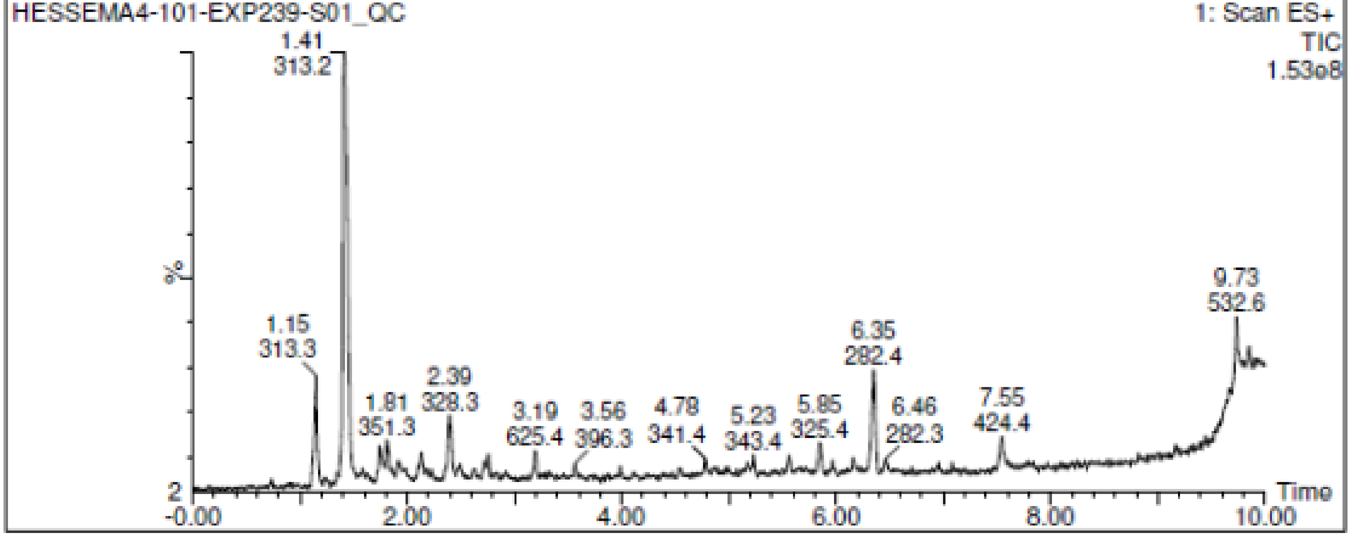

HPLC=0.1%TFA in H2O/ACN with 1.2mL/min

EM23001594453 | 14-Feb-2023 13:51:33 strubhe1-0208A-v24 | LCMS-ZQ1 Kinetex column, ZQ1_10m_Acidic

HESSEMA4-101-EXP239-S01_6d_QC | 1: Scan ES+ 313.147 8.13e7

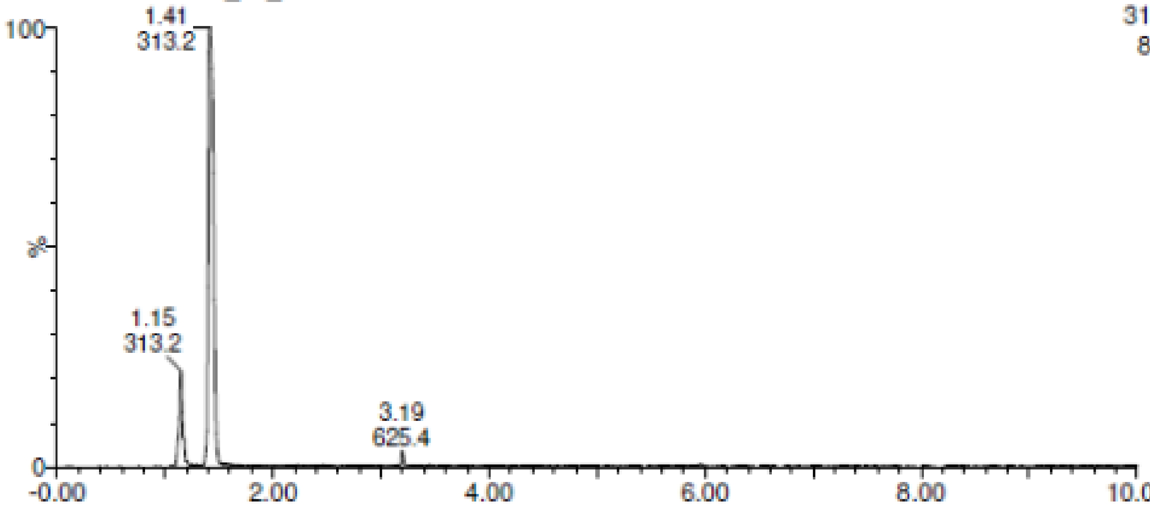

HESSEMA4-101-EXP239-S01_6d_QC Sm (Mn, 2x3) | 2: Diode Array 220 Range: 7.801e-1

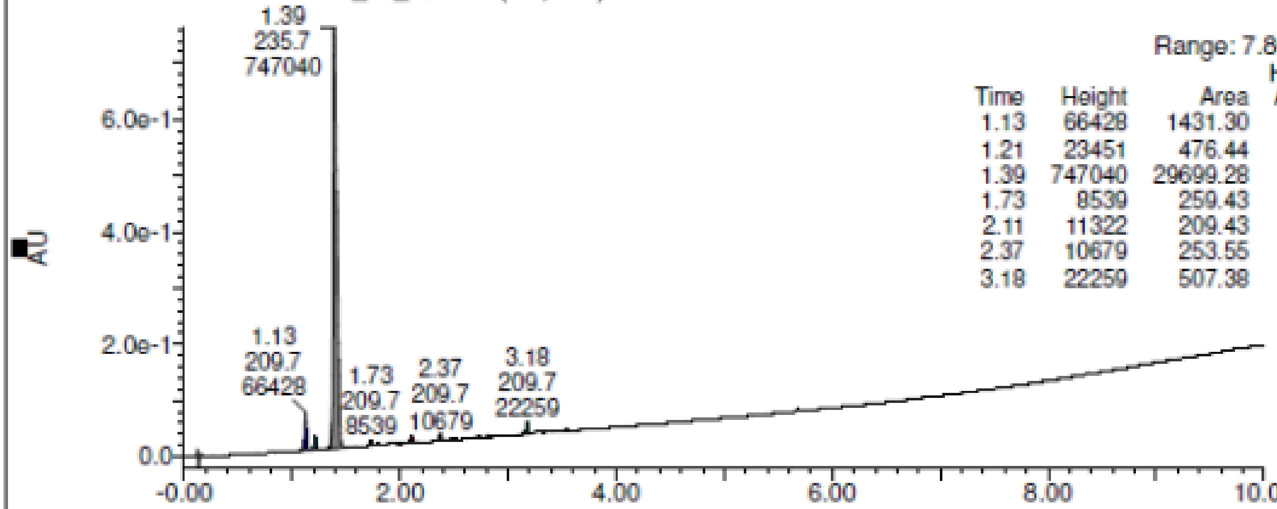

Time	Height	Area	Height Area%
1.13	66428	1431.30	4.36
1.21	23451	476.44	1.45
1.39	747040	29699.28	90.45
1.73	8539	259.43	0.79
2.11	11322	209.43	0.64
2.37	10679	253.55	0.77
3.18	22259	507.38	1.55

HESSEMA4-101-EXP239-S01_6d_QC | 1: Scan ES+ TIC 1.71e8

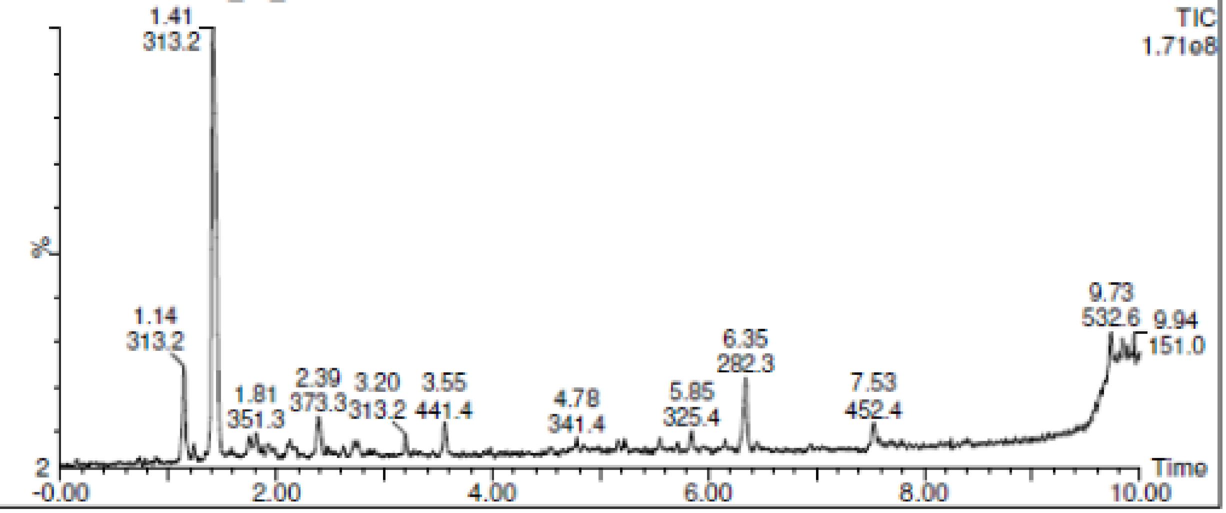

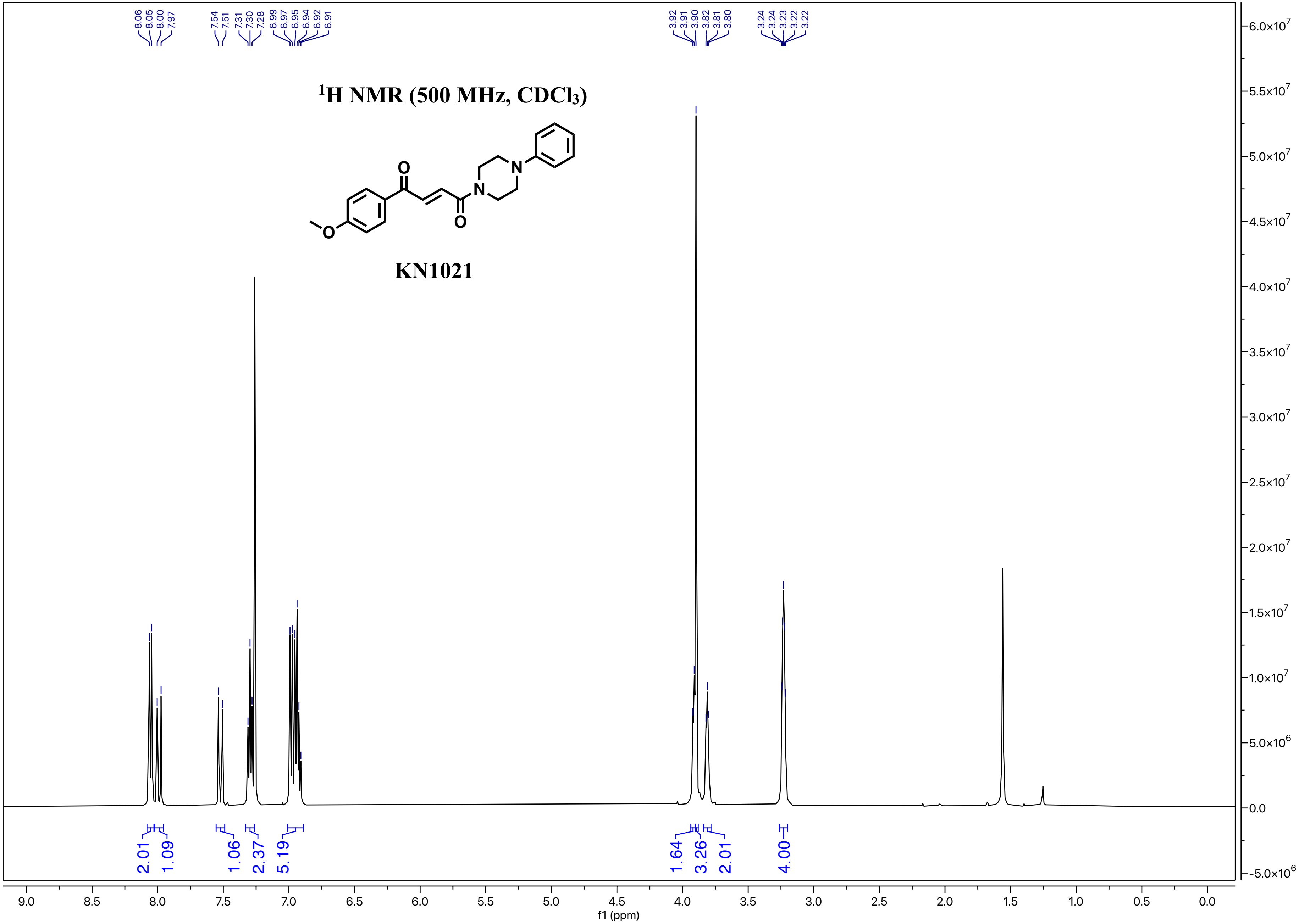

1H NMR (500 MHz, CDCl3)
KN1021
8.06 8.05 8.00 7.97
7.54 7.51 7.31 7.30 7.28 6.99 6.97 6.95 6.94 6.92 6.91
3.92 3.91 3.90 3.82 3.81 3.80
3.24 3.24 3.23 3.22 3.22
2.01 1.09 1.06 2.37 5.19 1.64 3.26 2.01 4.00
f1 (ppm)

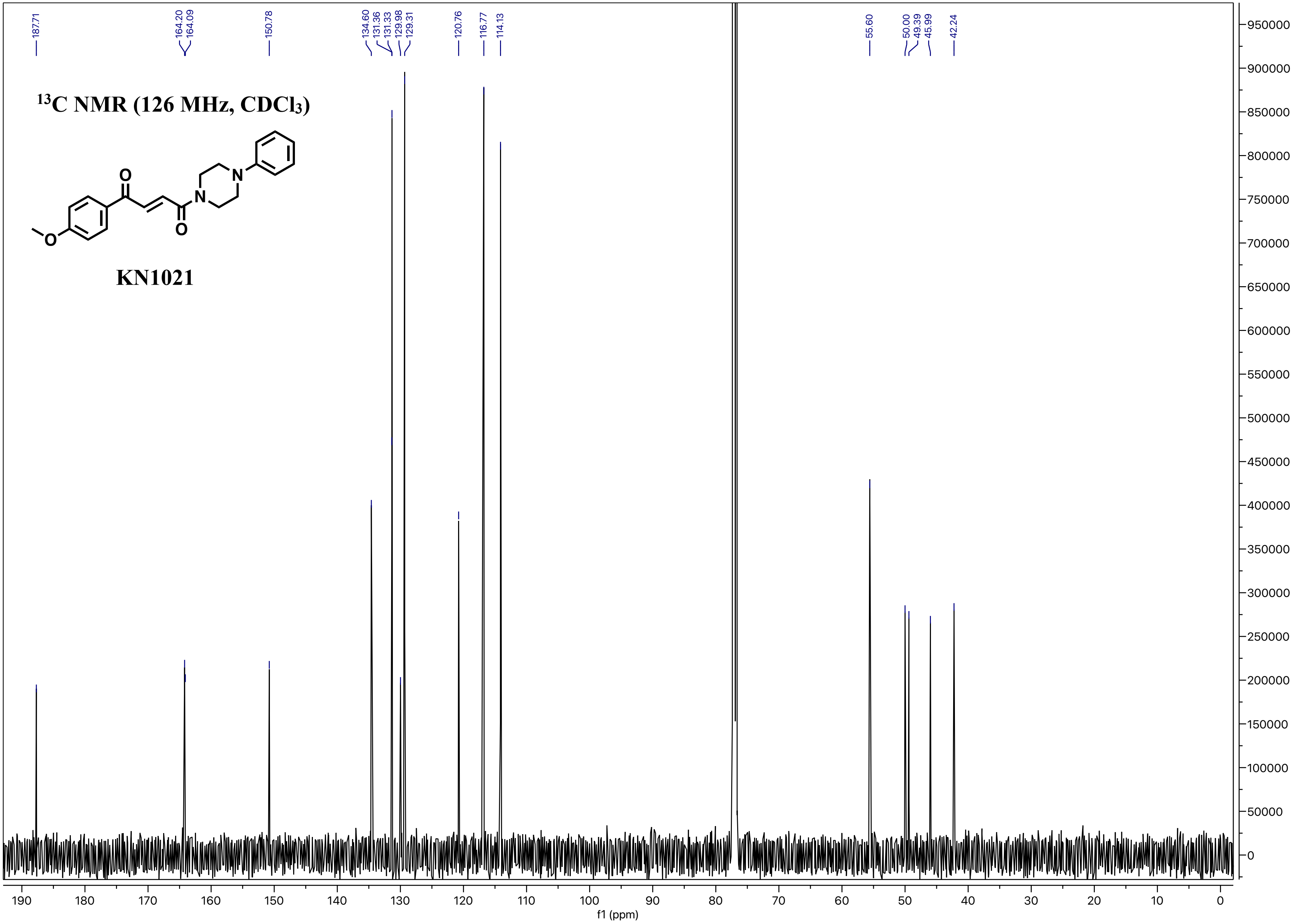

187.71
164.20
164.09
150.78
134.60
131.36
131.33
129.98
129.31
120.76
116.77
114.13
55.60
50.00
49.39
45.99
42.24
^{13}C NMR (126 MHz, CDCl$_3$)
O
O
N
N
O
KN1021
f1 (ppm)

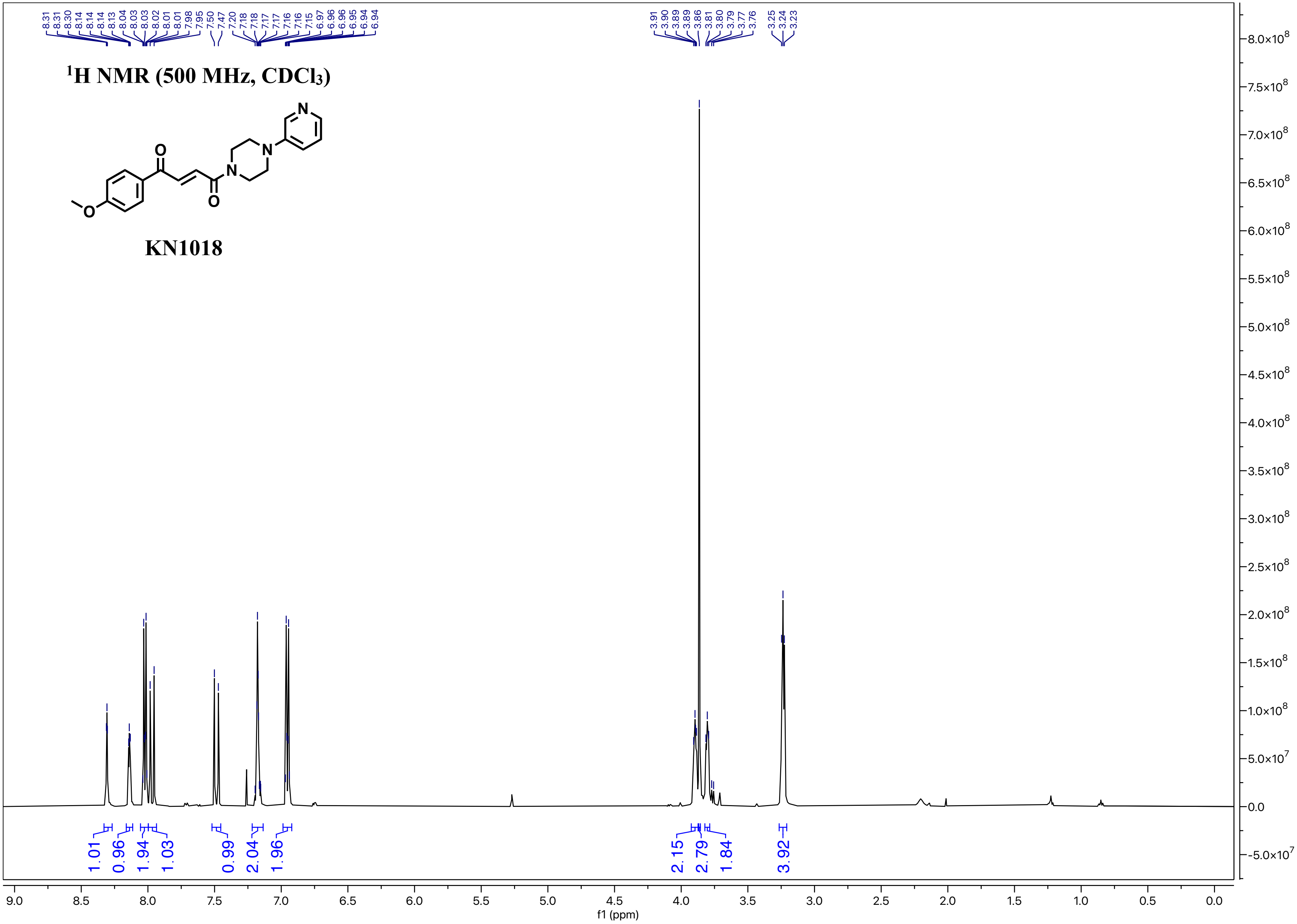

¹H NMR (500 MHz, CDCl3)
KN1018
f1 (ppm)

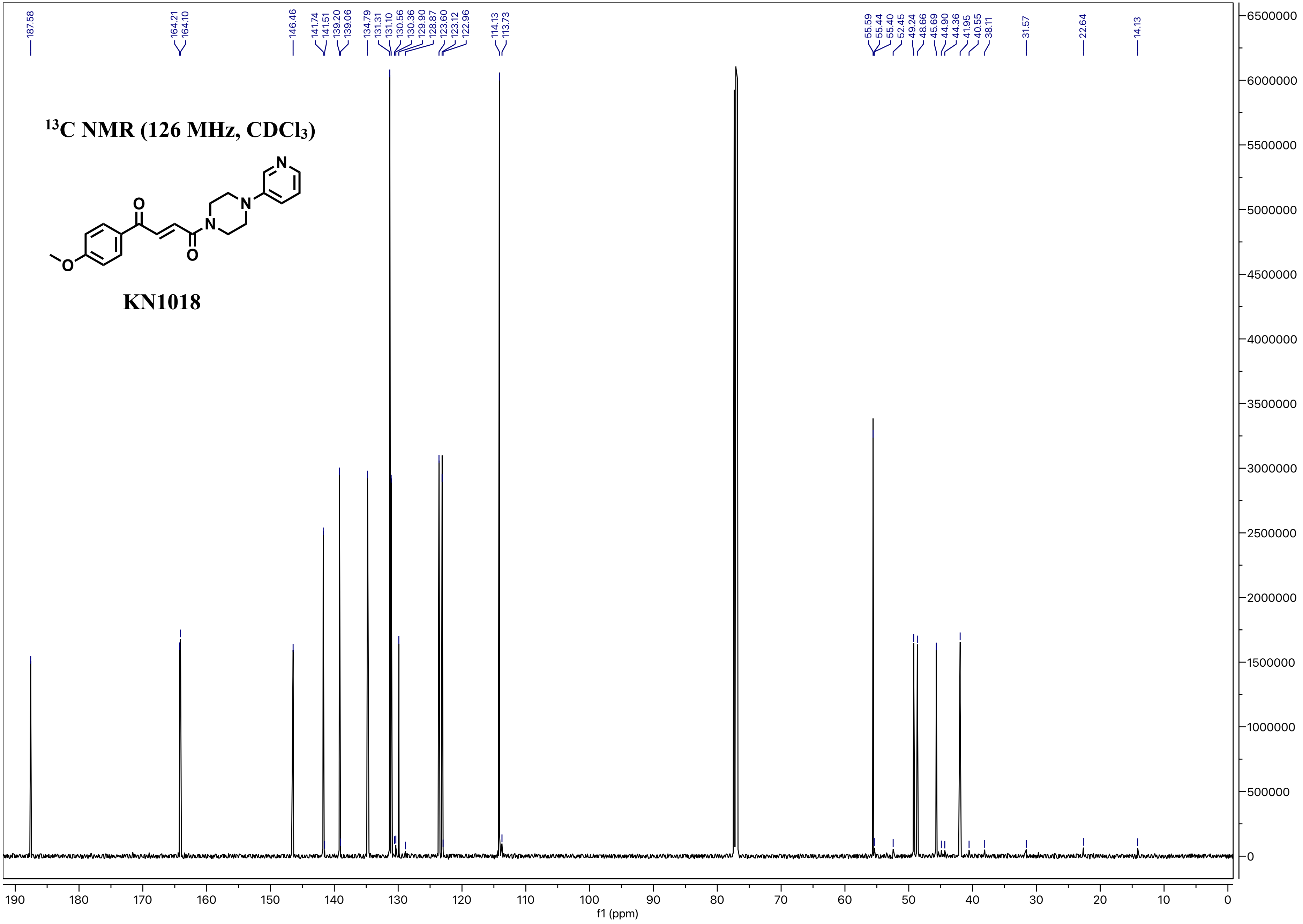

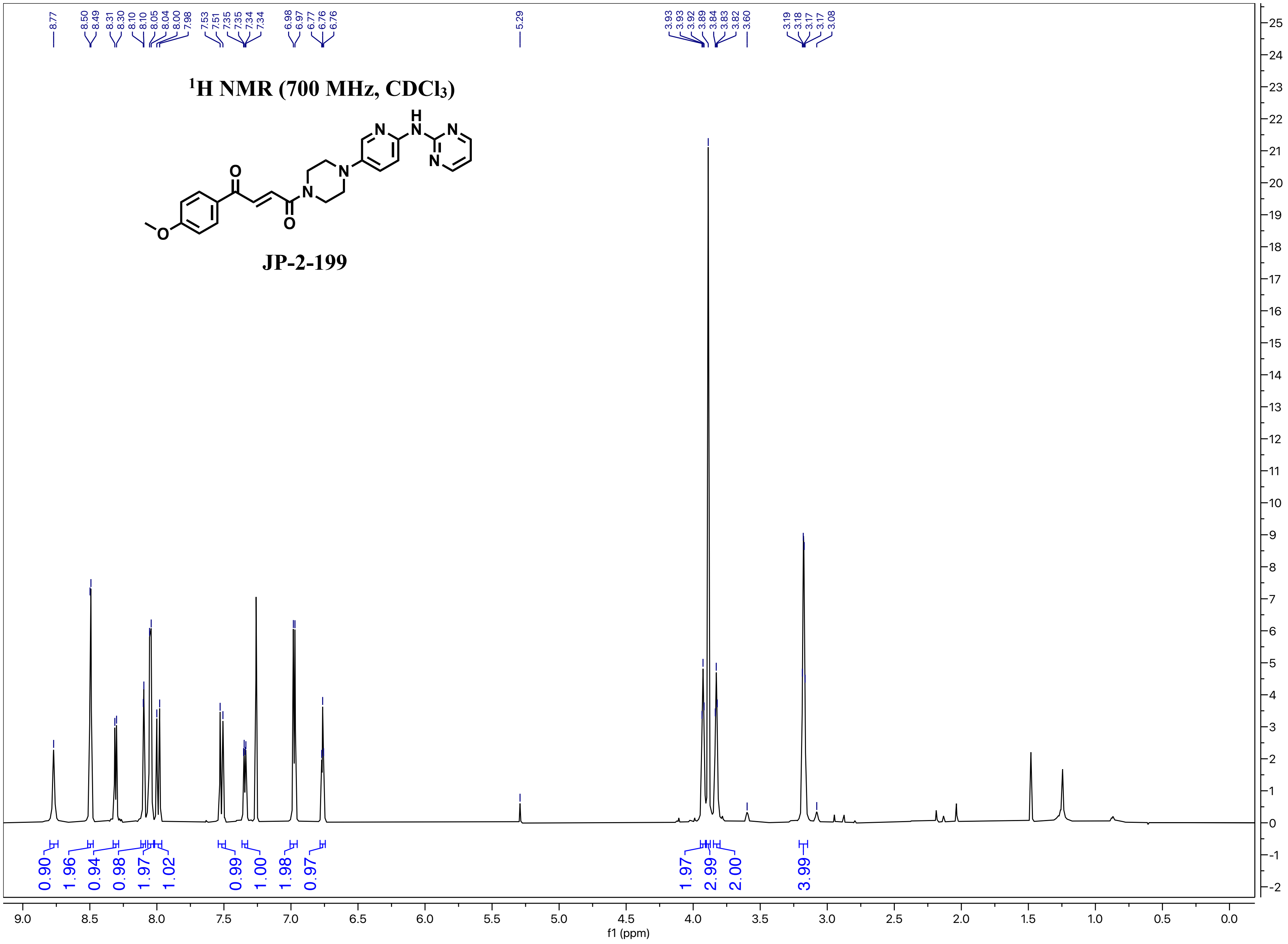

^{1}H NMR (700 MHz, CDCl$_3$)
JP-2-199
8.77 8.50 8.49 8.31 8.30 8.10 8.10 8.05 8.04 8.00 7.98 7.53 7.51 7.35 7.35 7.34 7.34 6.98 6.97 6.77 6.76 6.76
5.29
3.93 3.93 3.92 3.89 3.84 3.83 3.82 3.60
3.19 3.18 3.17 3.17 3.08
0.90 1.96 0.94 0.98 1.97 1.02 0.99 1.00 1.98 0.97
1.97 2.99 2.00
3.99
f1 (ppm)

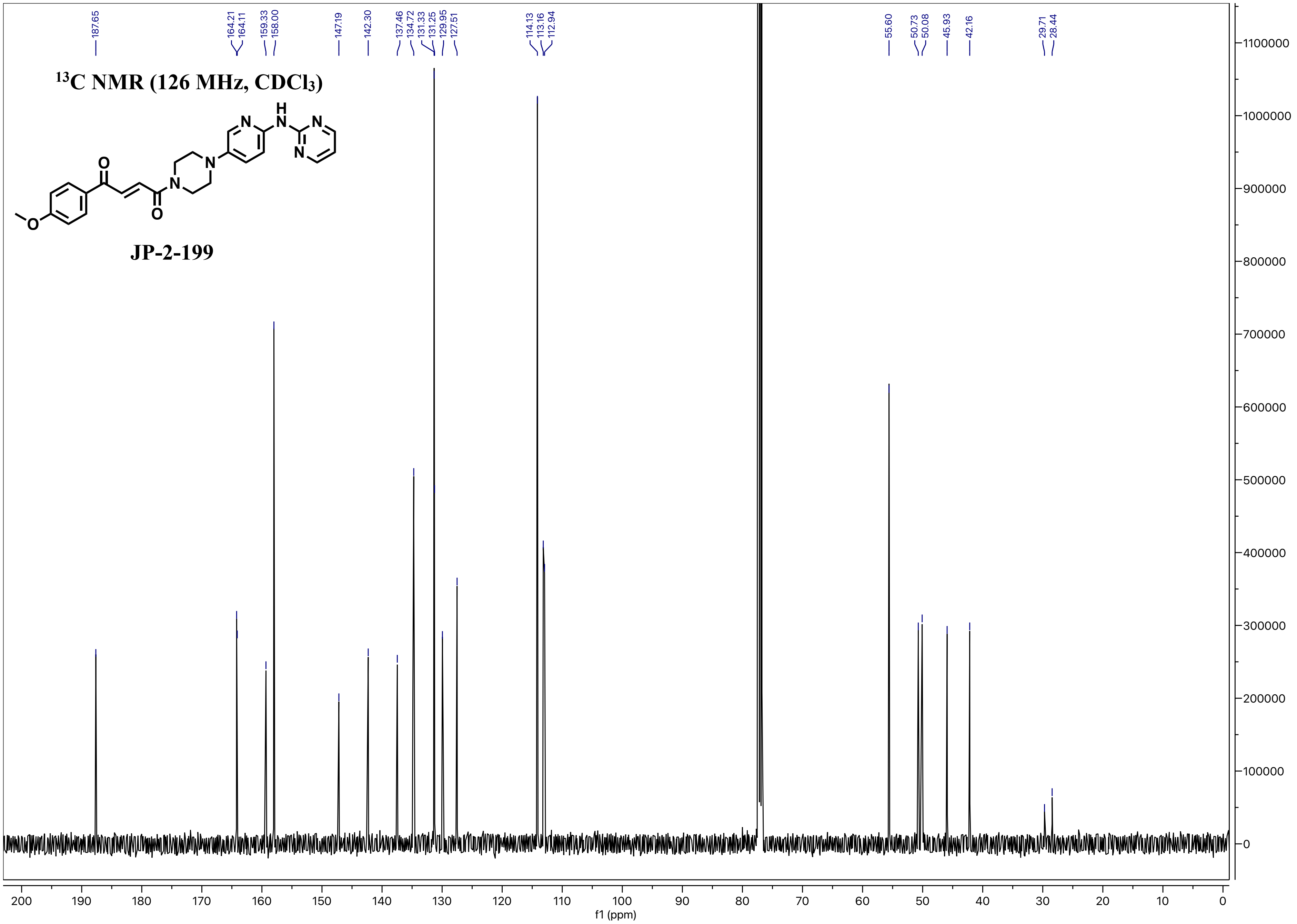

187.65
164.21
164.11
159.33
158.00
147.19
142.30
137.46
134.72
131.33
131.25
129.95
127.51
114.13
113.16
112.94
55.60
50.73
50.08
45.93
42.16
29.71
28.44
^{13}C NMR (126 MHz, $CDCl_3$)
JP-2-199
f1 (ppm)

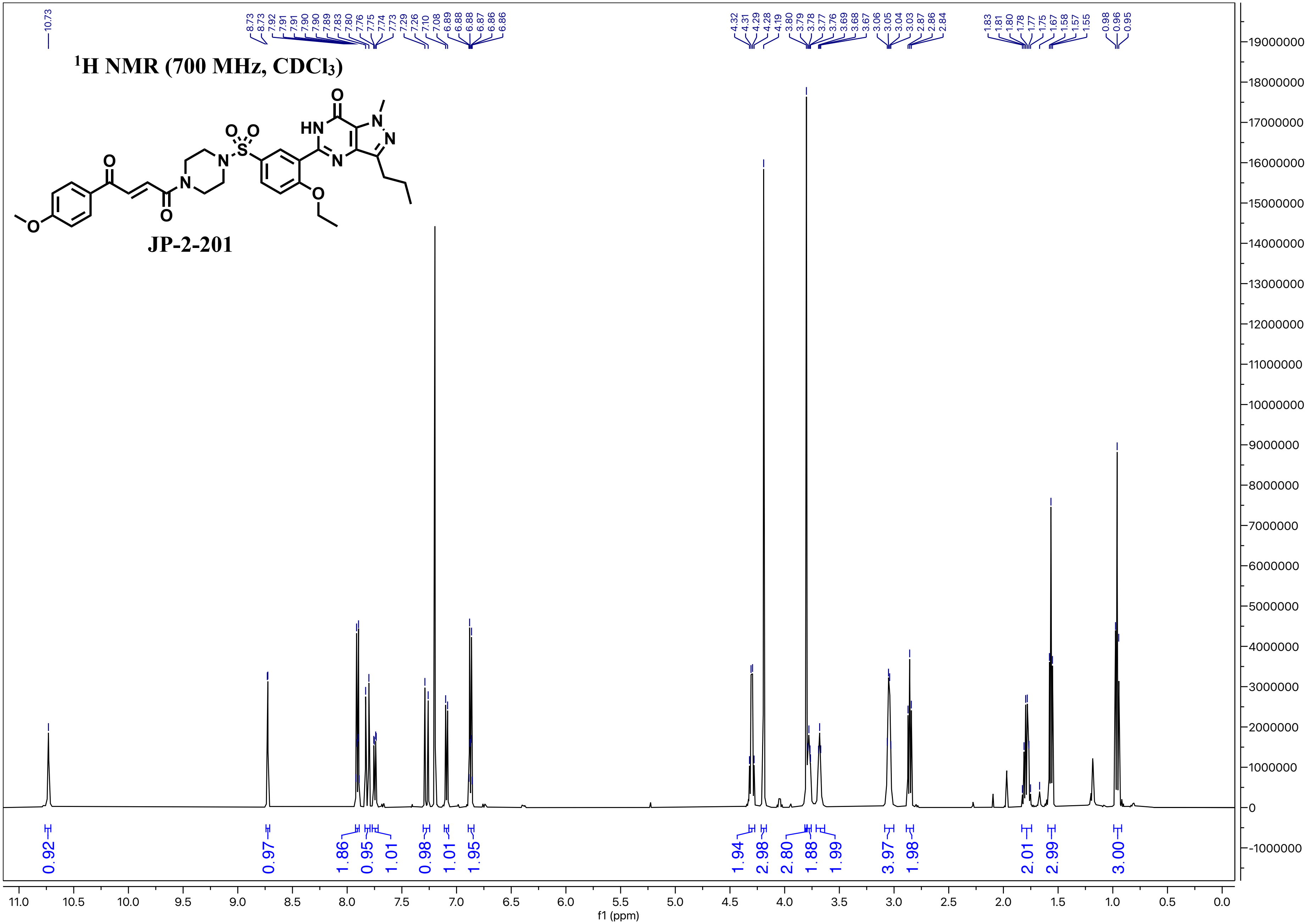

^{1}H NMR (700 MHz, CDCl$_3$)
JP-2-201
f1 (ppm)

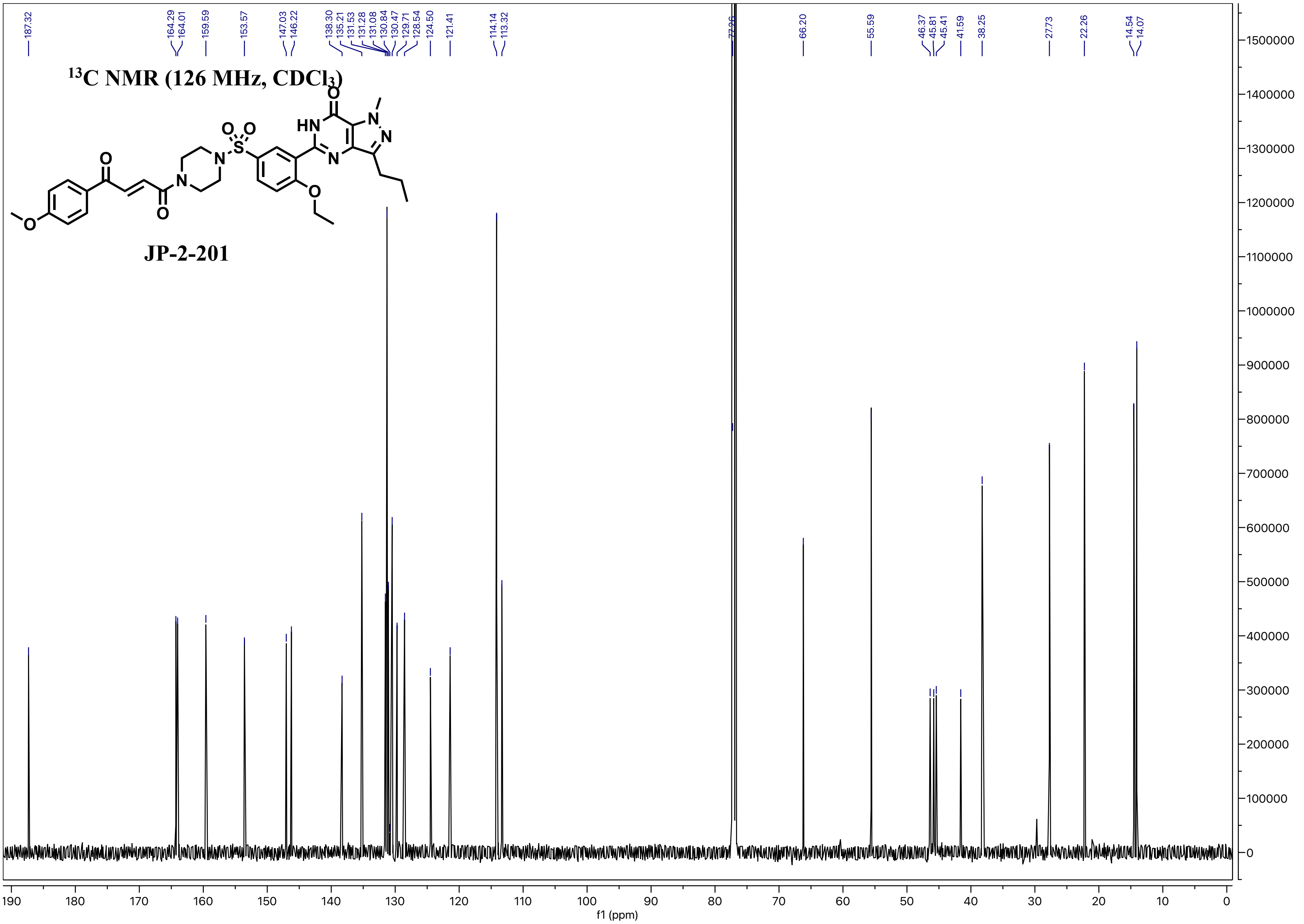

^{13}C NMR (126 MHz, CDCl$_3$)
JP-2-201
187.32
164.29
164.01
159.59
153.57
147.03
146.22
138.30
135.21
131.53
131.28
131.08
130.84
130.47
129.71
128.54
124.50
121.41
114.14
113.32
77.26
66.20
55.59
46.37
45.81
45.41
41.59
38.25
27.73
22.26
14.54
14.07
f1 (ppm)

HPLC=0.1%TFA in H2O/ACN with 1.2mL/min

**EM23001590294**	**13-Jan-2023**	**LCMS-ZQ1**
	**16:27:51**	**Kinetex column,**
	**strubhe1-0112A-v08**	**ZQ1_10m_Acidic**

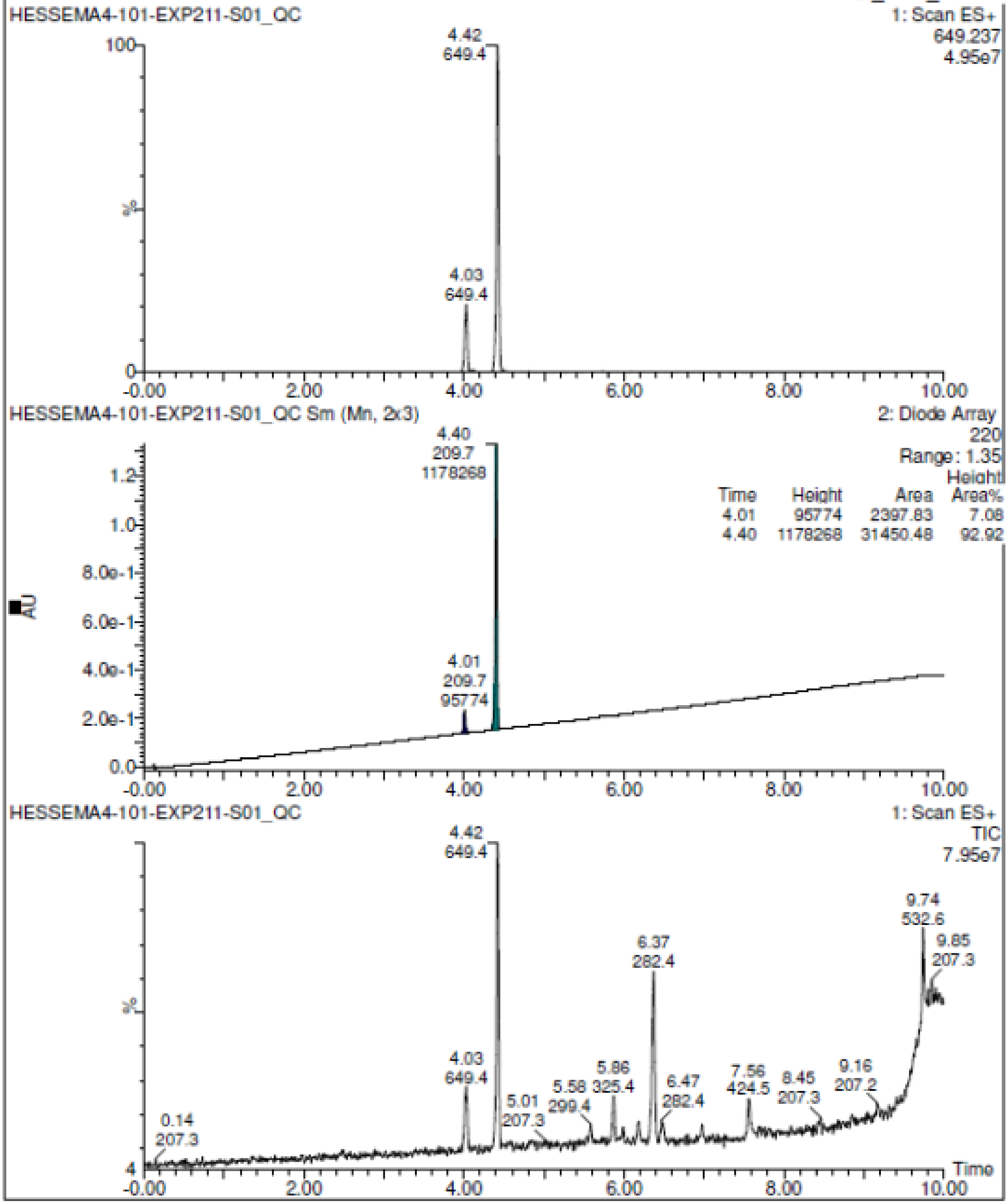

Time	Height	Area	Area%
4.01	95774	2397.83	7.08
4.40	1178268	31450.48	92.92

HPLC=0.1%TFA in H2O/ACN with 1.2mL/min

EM23001590294 | 19-Jan-2023 16:53:50 strubhe1-0118A-v08 | LCMS-ZQ1 Kinetex column, ZQ1_10m_Acidic

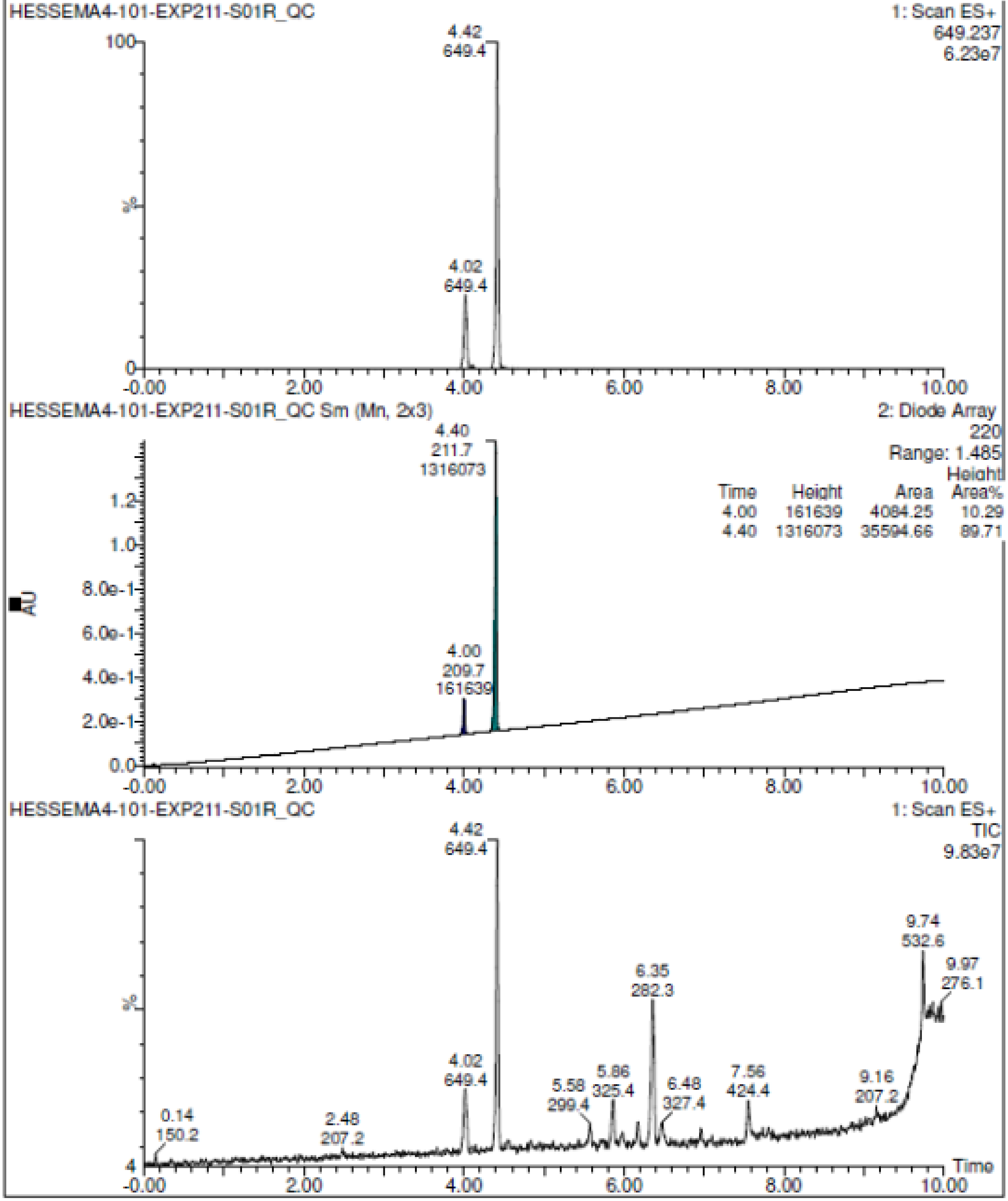

Time	Height	Area	Height Area%
4.00	161639	4084.25	10.29
4.40	1316073	35594.66	89.71

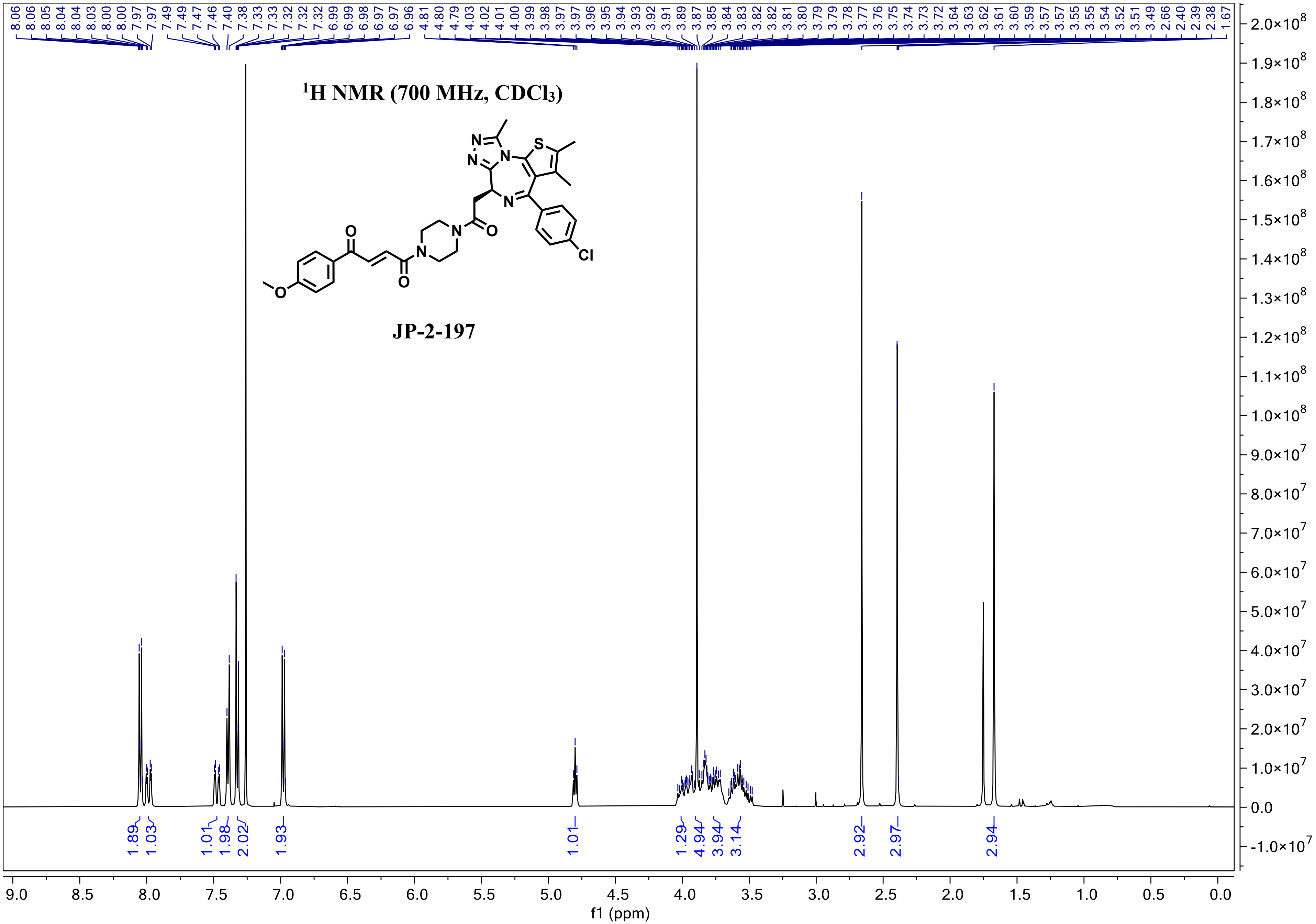

¹H NMR (700 MHz, CDCl3)
JP-2-197
f1 (ppm)

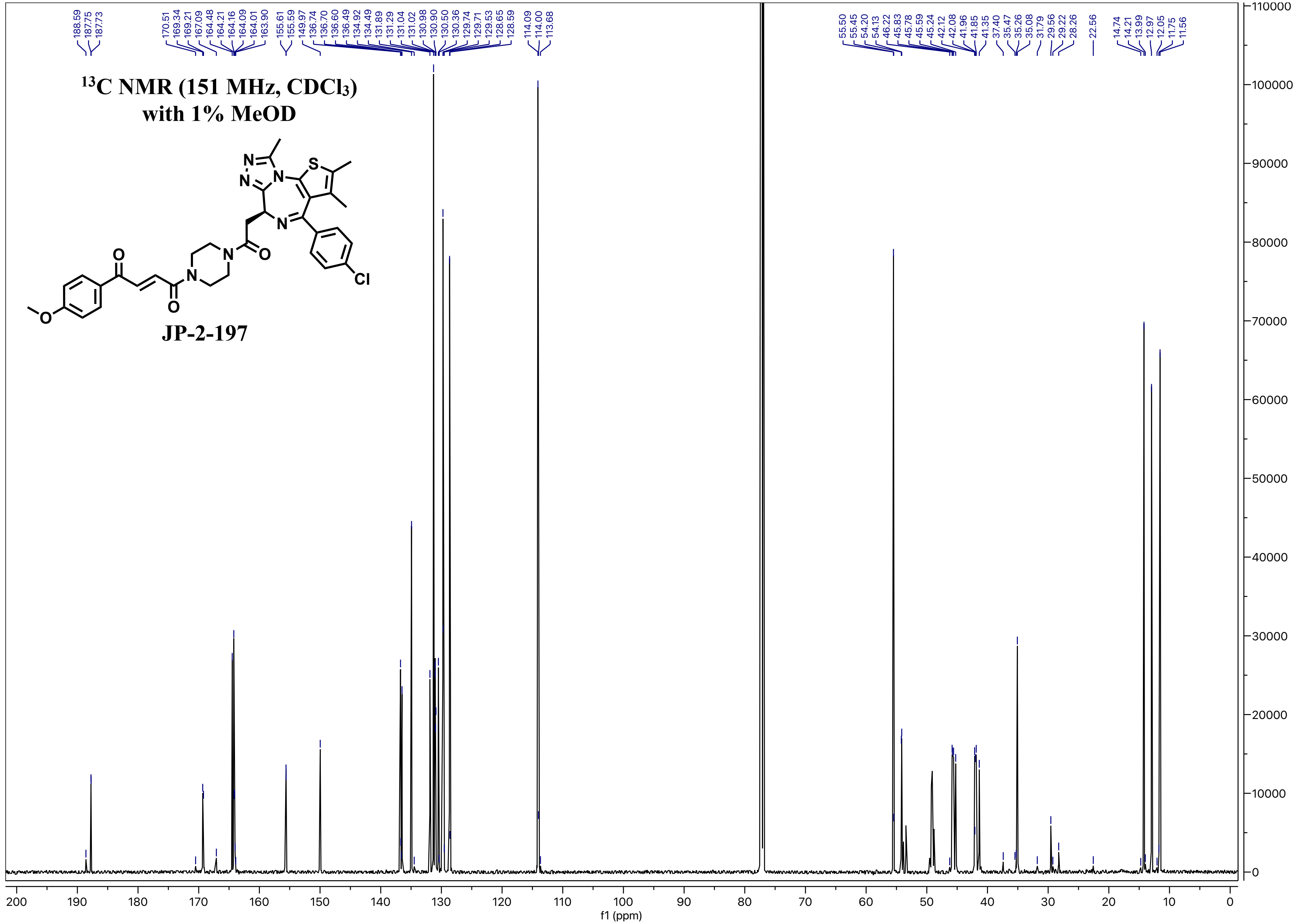

^{13}C NMR (151 MHz, CDCl$_3$)
with 1% MeOD
JP-2-197
f1 (ppm)

HPLC=0.1%TFA in H2O/ACN with 1.2mL/min

EM23001594422 **08-Feb-2023** **14:09:20** **strubhe1-0208A-v03** **LCMS-ZQ1** **Kinetex column, ZQ1_10m_Acidic**

HESSEMA4-101-EXP218-S01_QC

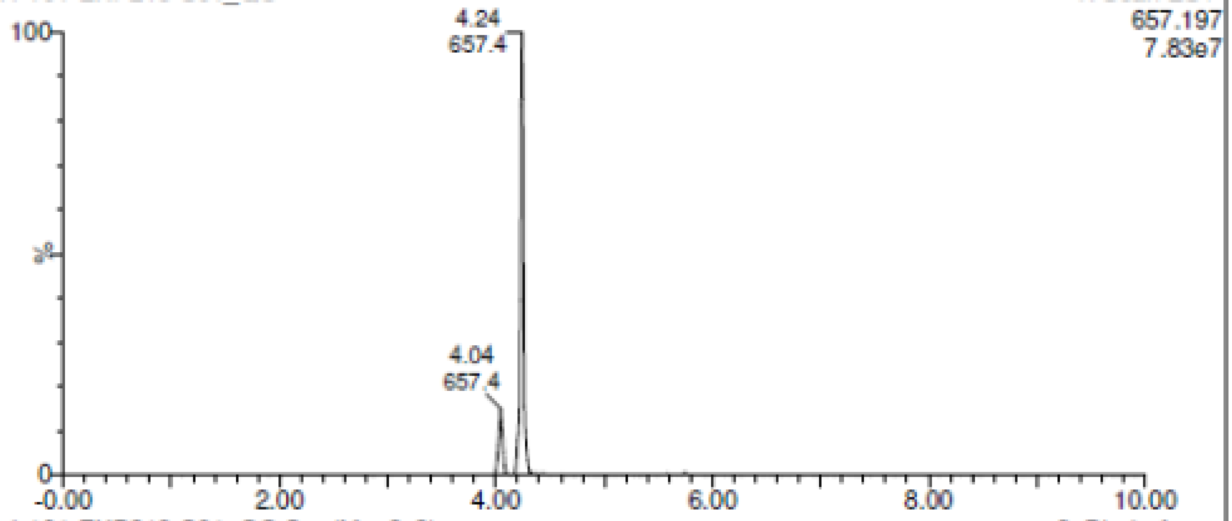

HESSEMA4-101-EXP218-S01_QC Sm (Mn, 2x3)

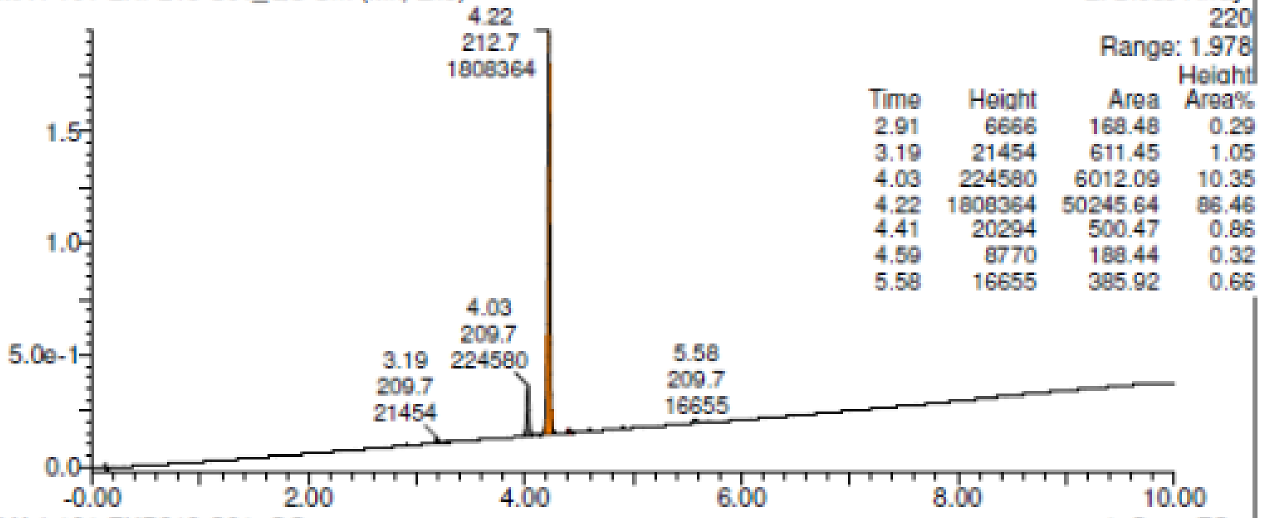

Time	Height	Area	Height Area%
2.91	6666	168.48	0.29
3.19	21454	611.45	1.05
4.03	224580	6012.09	10.35
4.22	1808364	50245.64	86.46
4.41	20294	500.47	0.86
4.59	8770	188.44	0.32
5.58	16655	385.92	0.66

HESSEMA4-101-EXP218-S01_QC

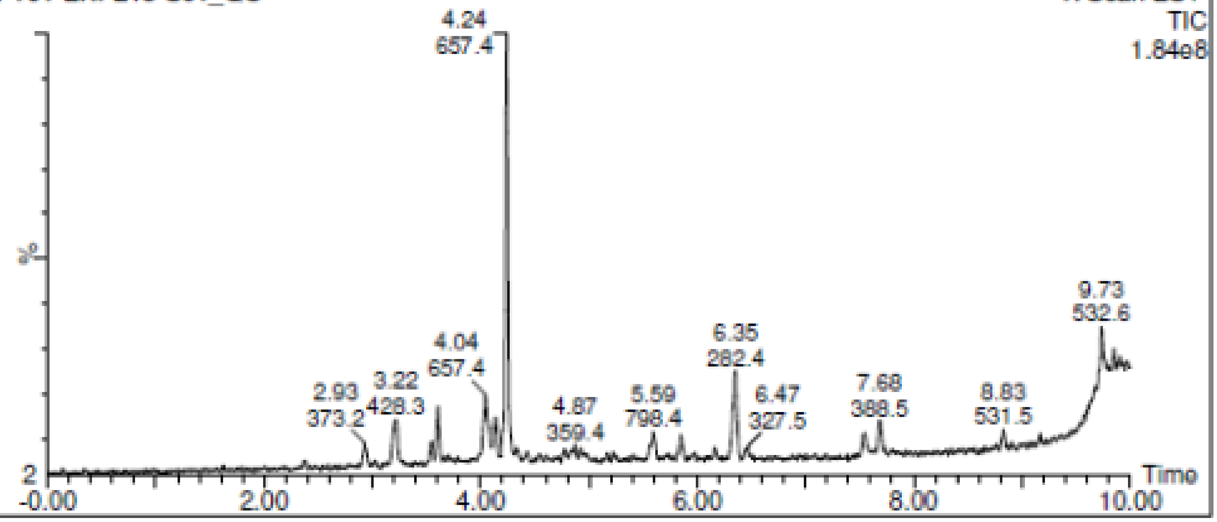

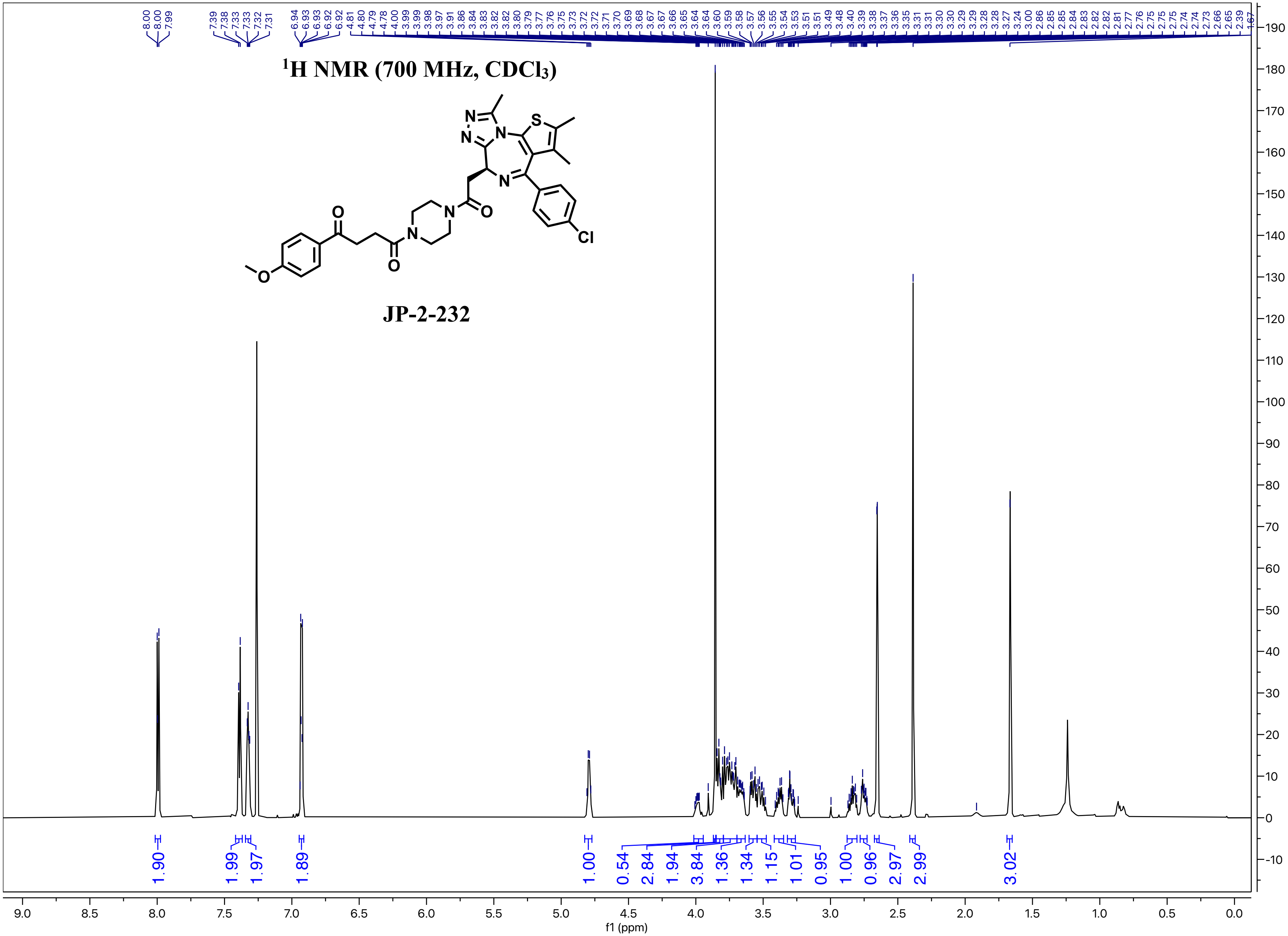

^{1}H NMR (700 MHz, CDCl$_3$)
JP-2-232
8.00 8.00 7.99 7.39 7.38 7.33 7.33 7.32 7.31 6.94 6.93 6.93 6.92 6.92 4.81 4.80 4.79 4.78 4.00 3.99 3.99 3.98 3.97 3.91 3.86 3.84 3.83 3.82 3.82 3.80 3.79 3.77 3.76 3.75 3.73 3.72 3.72 3.71 3.70 3.69 3.68 3.67 3.67 3.66 3.65 3.64 3.64 3.60 3.59 3.58 3.57 3.56 3.55 3.54 3.53 3.51 3.51 3.49 3.48 3.40 3.39 3.38 3.37 3.36 3.35 3.31 3.31 3.30 3.30 3.29 3.29 3.28 3.28 3.27 3.24 3.00 2.86 2.85 2.85 2.84 2.83 2.82 2.82 2.81 2.77 2.76 2.75 2.75 2.75 2.74 2.74 2.73 2.66 2.65 2.39 1.67
1.90 1.99 1.97 1.89 1.00 0.54 2.84 1.94 3.84 1.36 1.34 1.15 1.01 0.95 1.00 0.96 2.97 2.99 3.02
f1 (ppm)

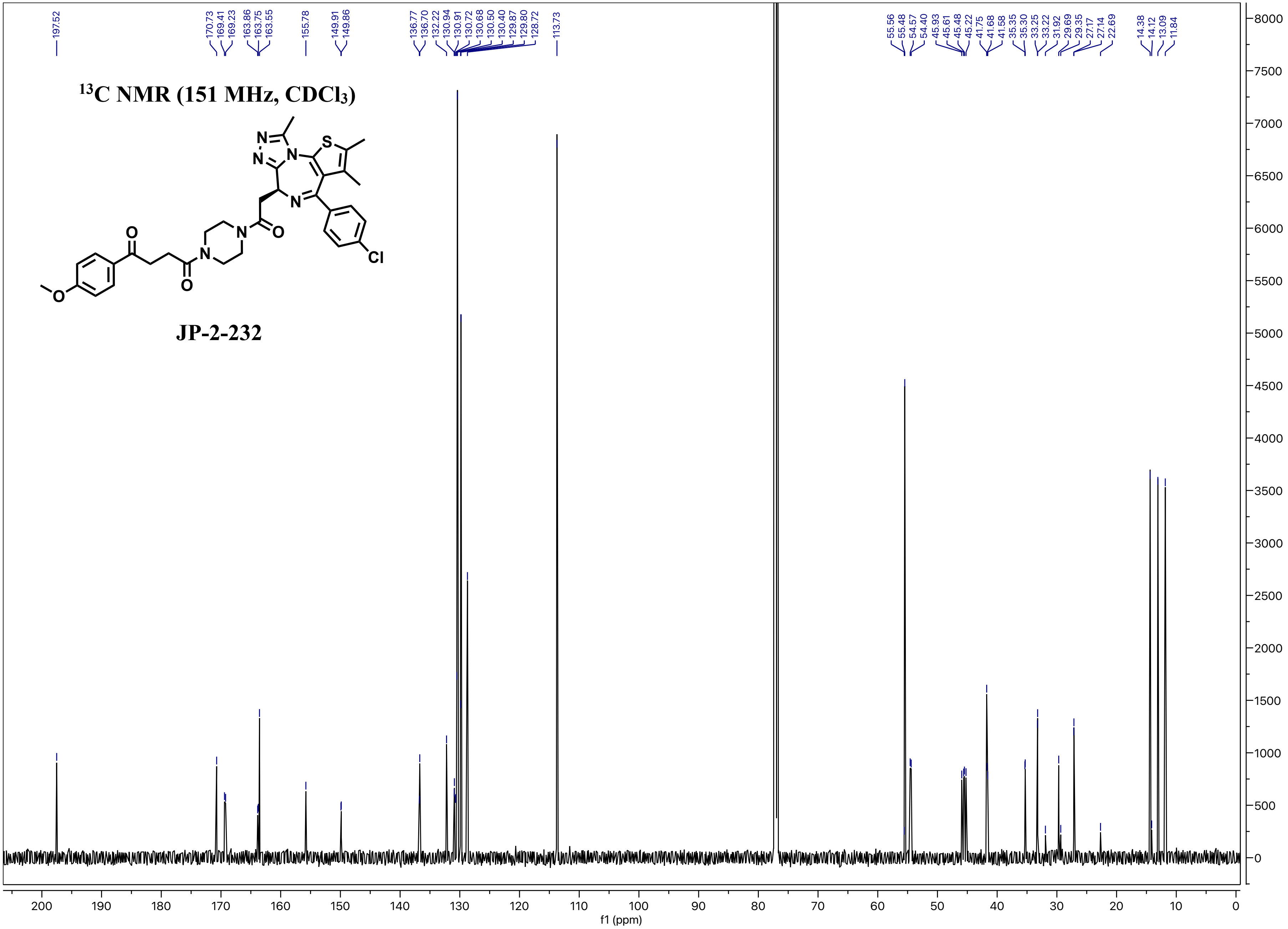
197.52
170.73
169.41
169.23
163.86
163.75
163.55
155.78
149.91
149.86
136.77
136.70
132.22
130.94
130.91
130.72
130.68
130.50
130.40
129.87
129.80
128.72
113.73
55.56
55.48
54.57
54.40
45.93
45.61
45.48
45.22
41.75
41.68
41.58
35.35
35.30
33.25
33.22
31.92
29.69
29.35
27.17
27.14
22.69
14.38
14.12
13.09
11.84
^{13}C NMR (151 MHz, CDCl$_3$)
JP-2-232
f1 (ppm)

HPLC=0.1%TFA in H2O/ACN with 1.2mL/min

EM23001594447	08-Feb-2023<br>17:39:20<br>strubhe1-0208A-v22	LCMS-ZQ1<br>Kinetex column,<br>ZQ1_10m_Acidic

HESSEMA4-101-EXP237-S01_QC

1: Scan ES+
659.213
7.92e7

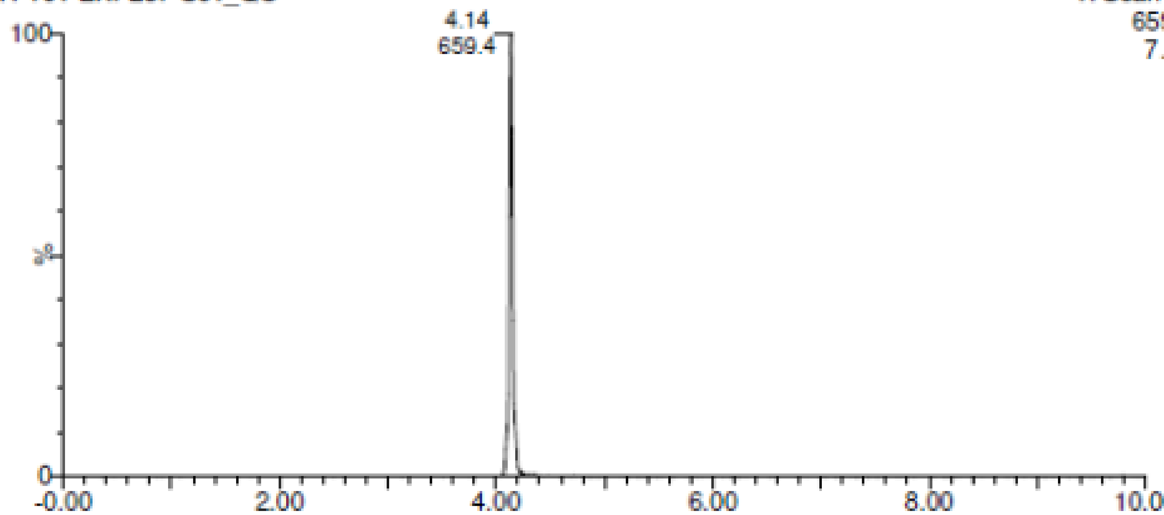

HESSEMA4-101-EXP237-S01_QC Sm (Mn, 2x3)

2: Diode Array
220
Range: 2.241

Time	Height	Area	Height Area%
4.12	2076393	62736.04	100.00

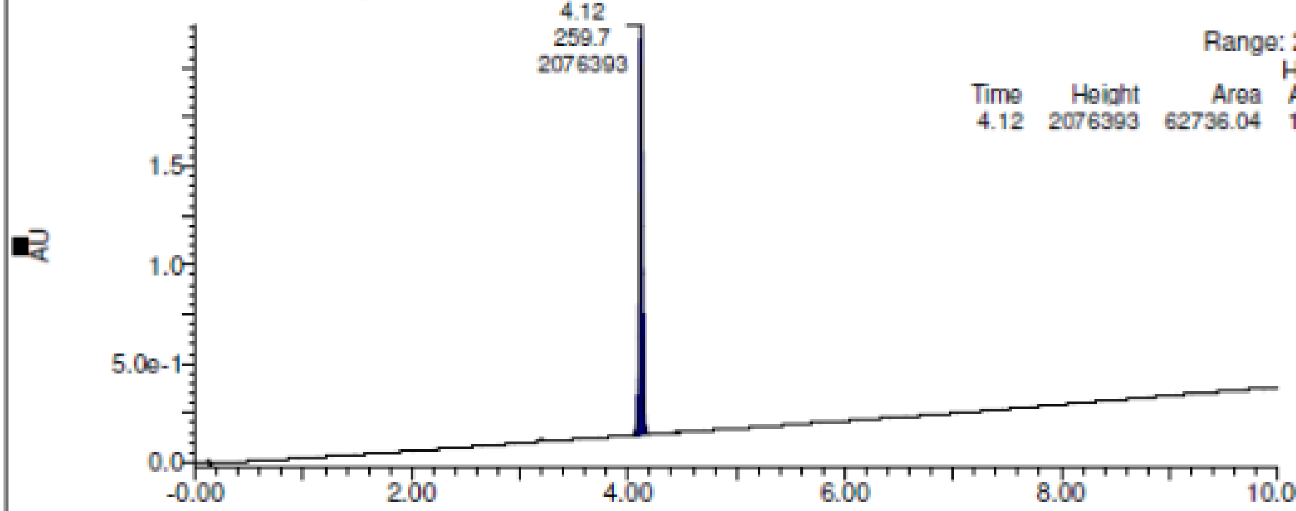

HESSEMA4-101-EXP237-S01_QC

1: Scan ES+
TIC
2.43e8

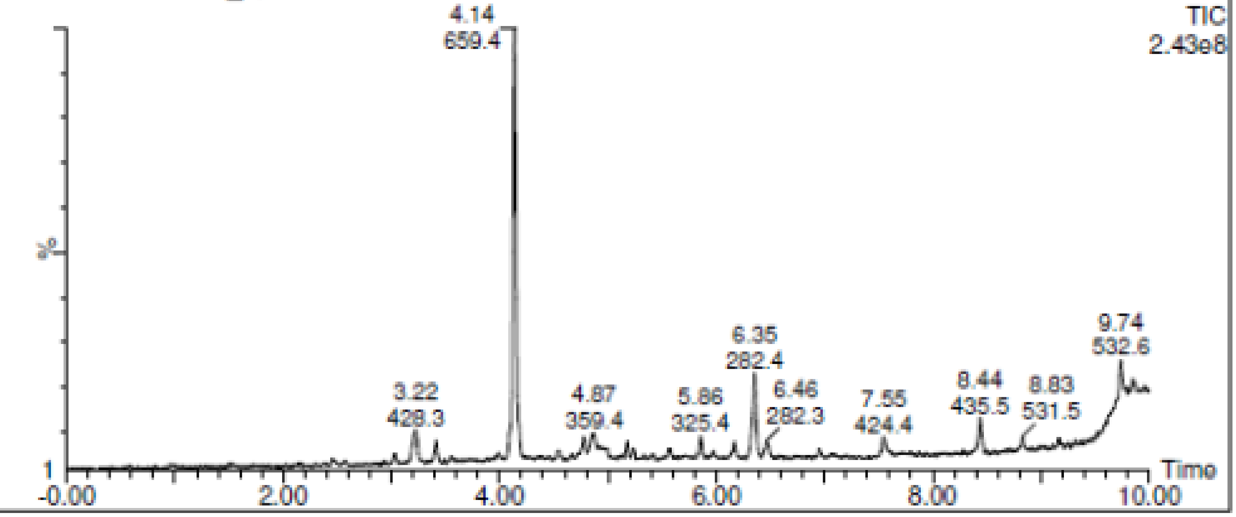

^{1}H NMR (500 MHz, CDCl3)

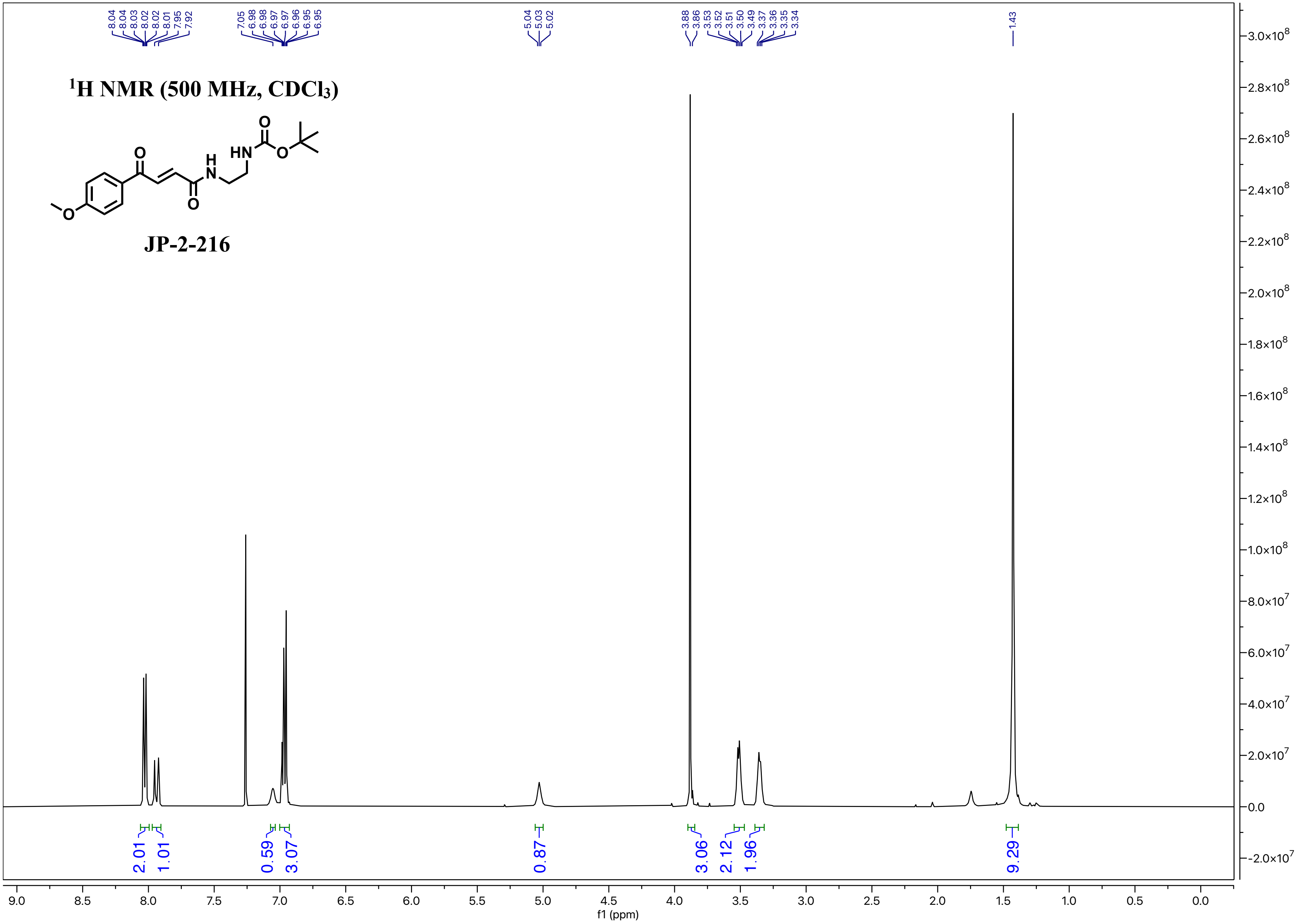

JP-2-216

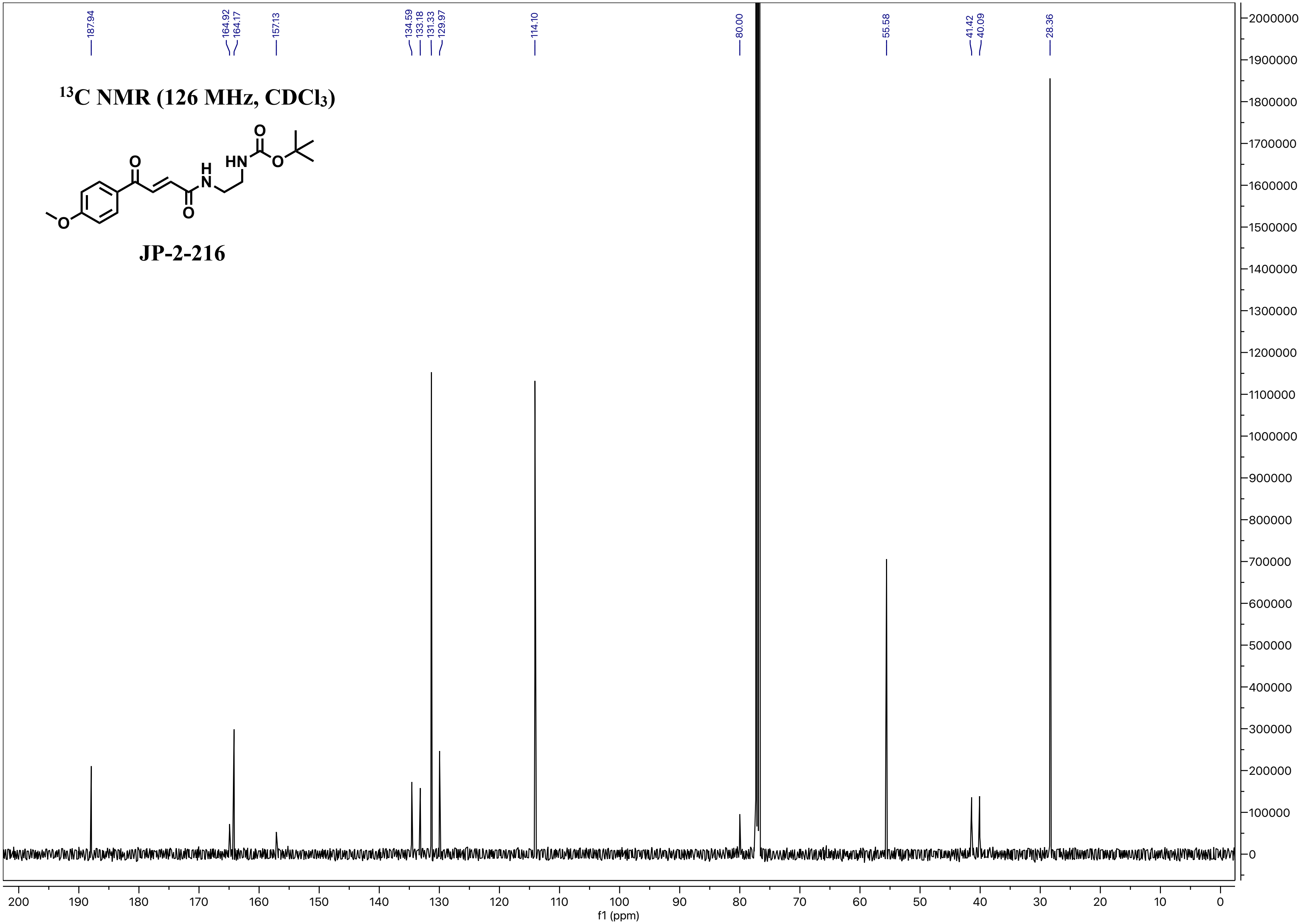

187.94
164.92
164.17
157.13
134.59
133.18
131.33
129.97
114.10
80.00
55.58
41.42
40.09
28.36
13C NMR (126 MHz, CDCl3)
JP-2-216
f1 (ppm)

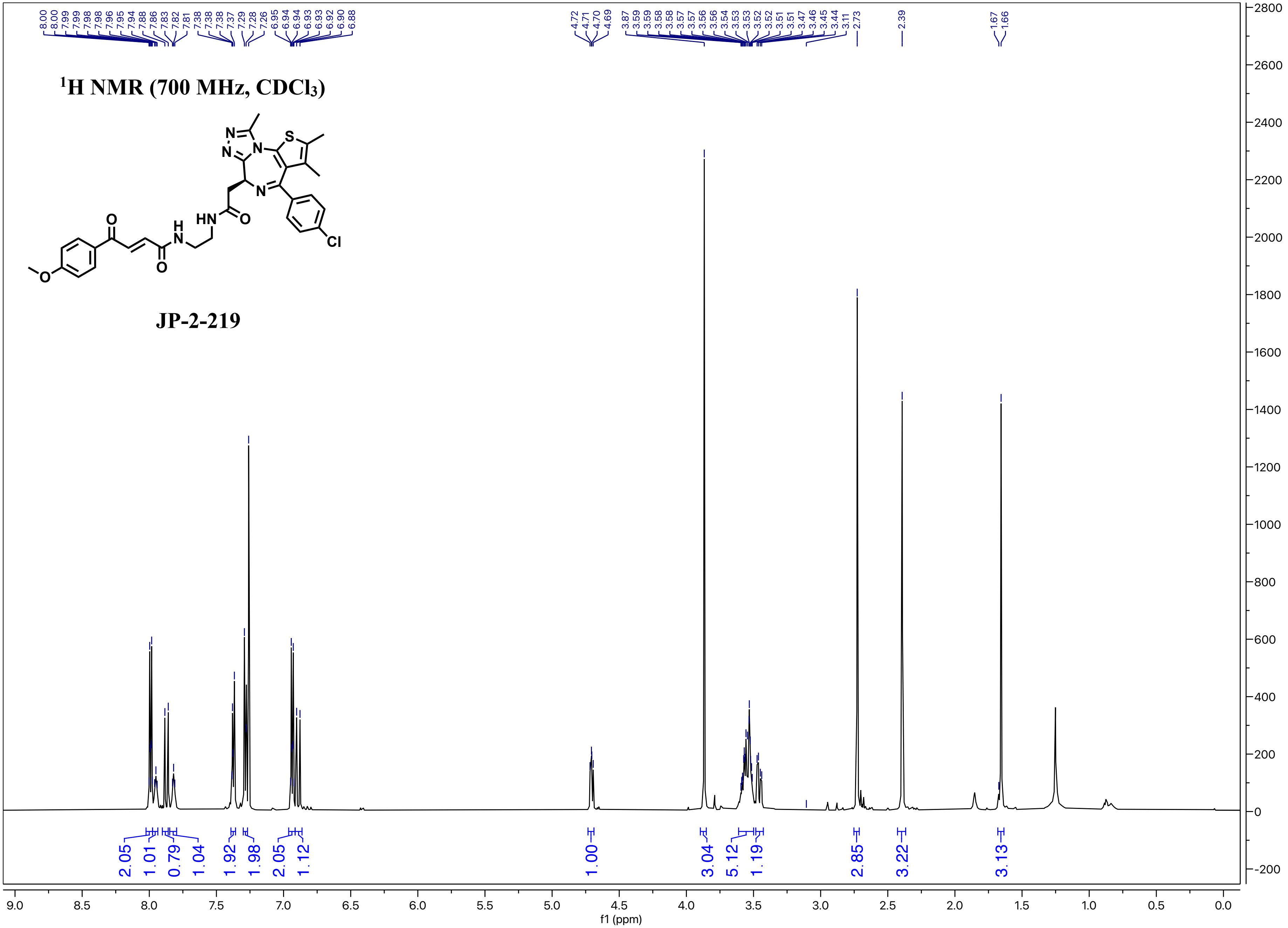

8.00 8.00 7.99 7.98 7.98 7.96 7.95 7.94 7.88 7.86 7.83 7.82 7.81 7.38 7.38 7.38 7.37 7.29 7.28 7.26 6.95 6.94 6.94 6.93 6.93 6.92 6.90 6.88
4.72 4.71 4.70 4.69 3.87 3.59 3.59 3.58 3.58 3.57 3.57 3.56 3.56 3.54 3.53 3.53 3.52 3.52 3.51 3.51 3.47 3.46 3.45 3.44 3.11 2.73 2.39 1.67 1.66
^{1}H NMR (700 MHz, CDCl$_3$)
JP-2-219
2.05 1.01 0.79 1.04 1.92 1.98 2.05 1.12 1.00 3.04 5.12 1.19 2.85 3.22 3.13
f1 (ppm)

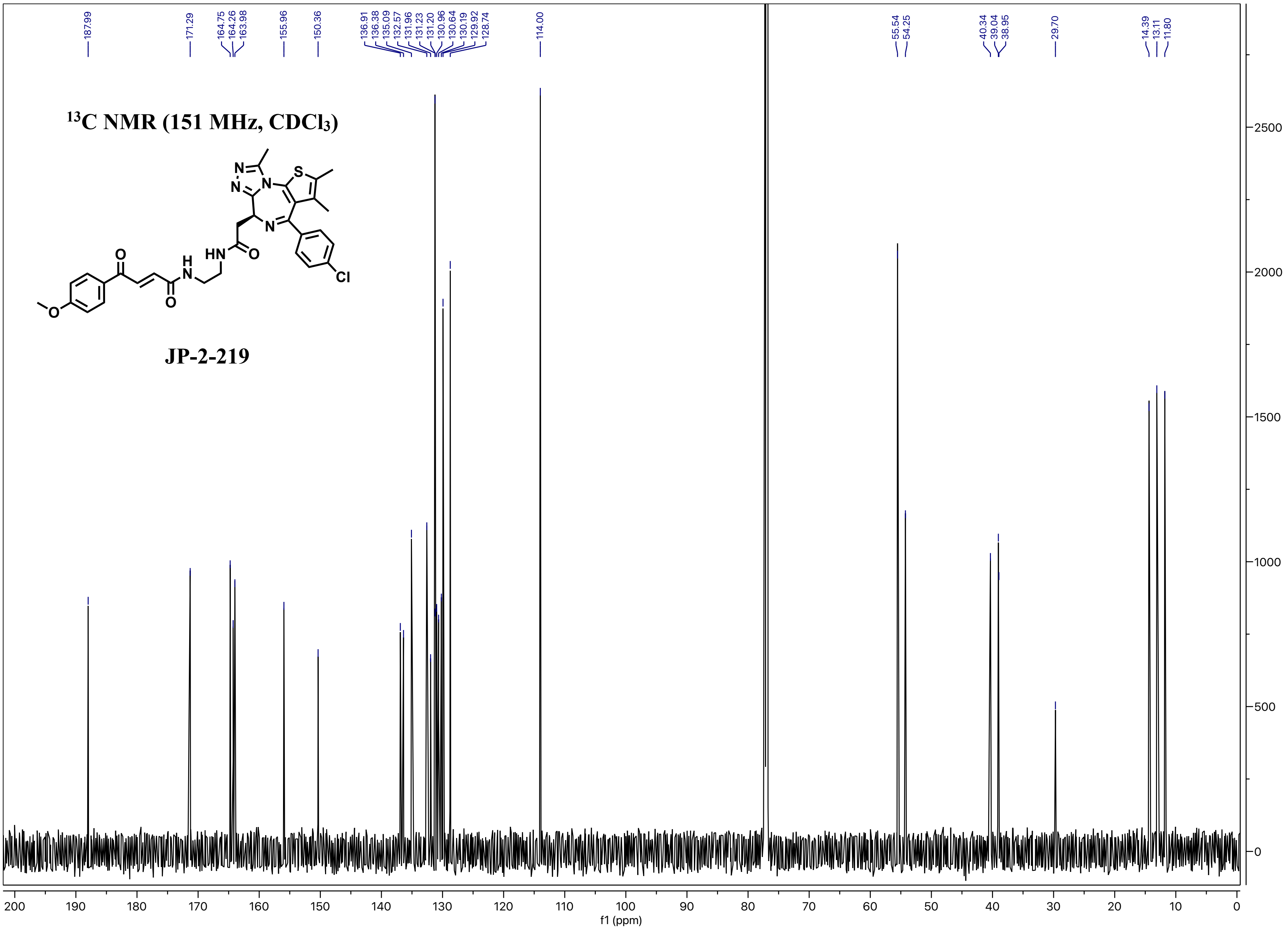

13C NMR (151 MHz, CDCl3)
JP-2-219
187.99
171.29
164.75
164.26
163.98
155.96
150.36
136.91
136.38
135.09
132.57
131.96
131.23
131.20
130.96
130.64
130.19
129.92
128.74
114.00
55.54
54.25
40.34
39.04
38.95
29.70
14.39
13.11
11.80
f1 (ppm)

HPLC=0.1%TFA in H2O/ACN with 1.2mL/min

EM23001594448 | 08-Feb-2023 17:50:24 strubhe1-0208A-v23 | LCMS-ZQ1 Kinetex column, ZQ1_10m_Acidic

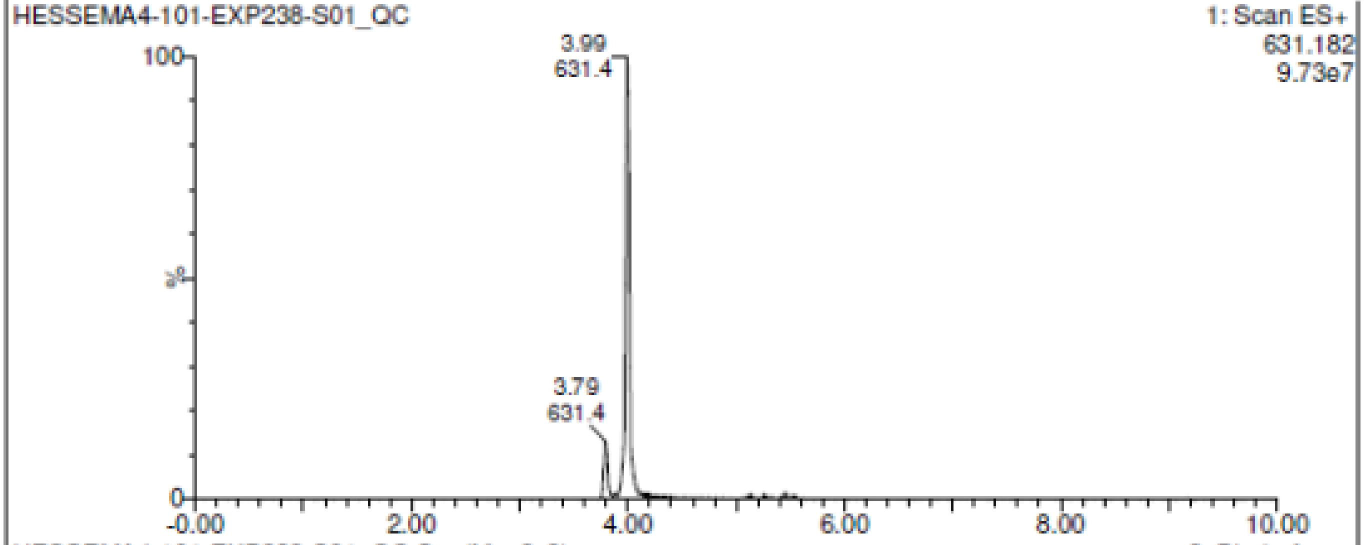

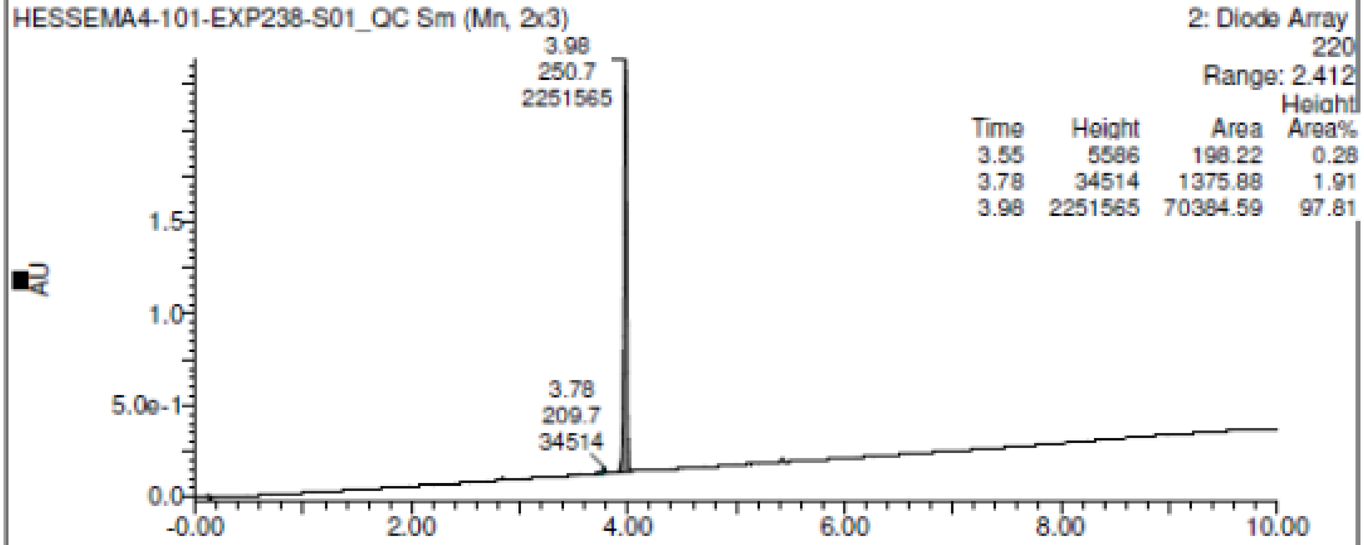

Time	Height	Area	Height Area%
3.55	5586	198.22	0.28
3.78	34514	1375.88	1.91
3.98	2251565	70384.59	97.81

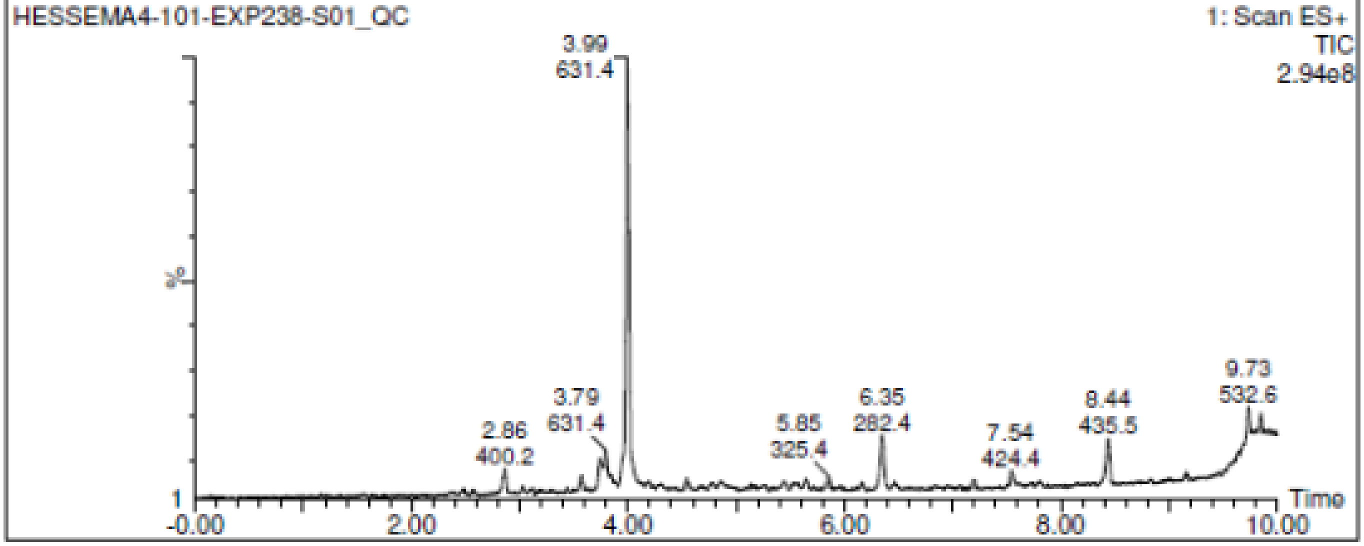

HPLC=0.1%TFA in H2O/ACN with 1.2mL/min

EM23001594448 | 14-Feb-2023 13:40:30 | strubhe1-0208A-v23 | LCMS-ZQ1 Kinetex column, ZQ1_10m_Acidic

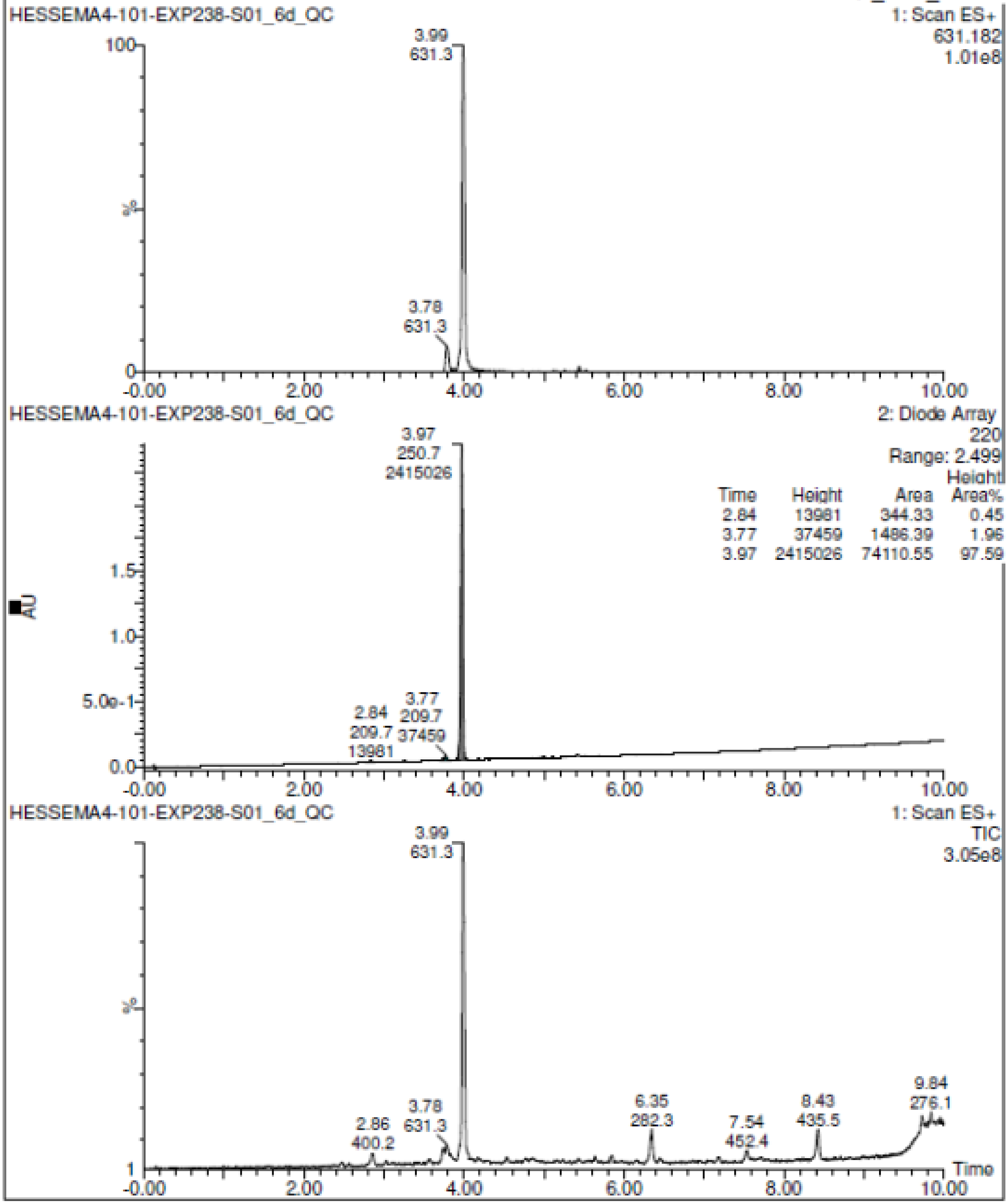

Time	Height	Area	Height Area%
2.84	13981	344.33	0.45
3.77	37459	1486.39	1.96
3.97	2415026	74110.55	97.59

^1H NMR (400 MHz, $CDCl_3$)

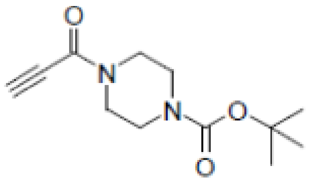

S1

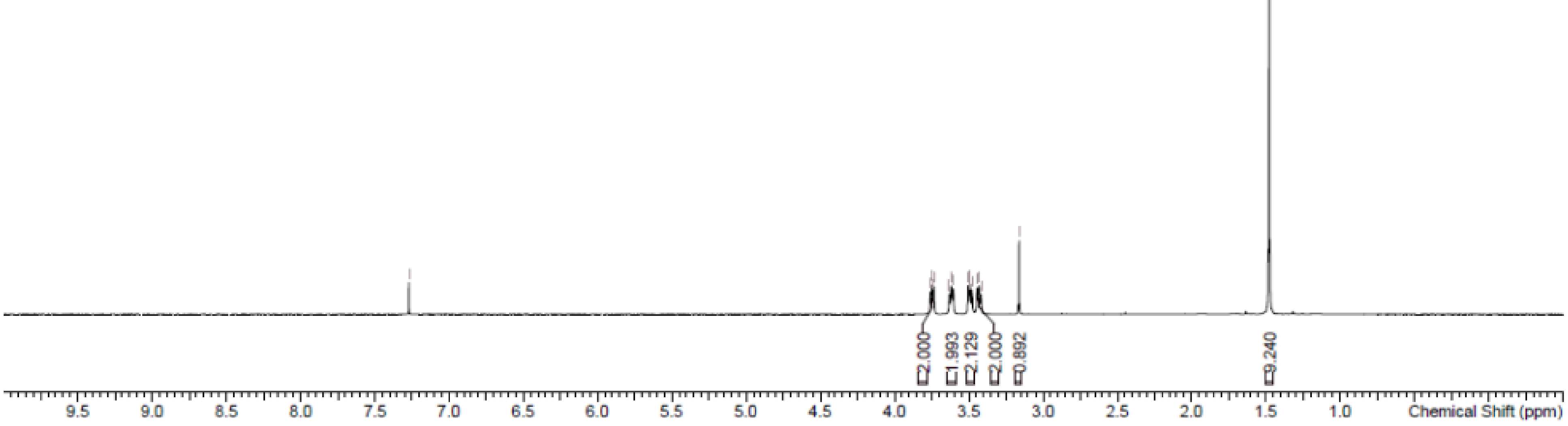

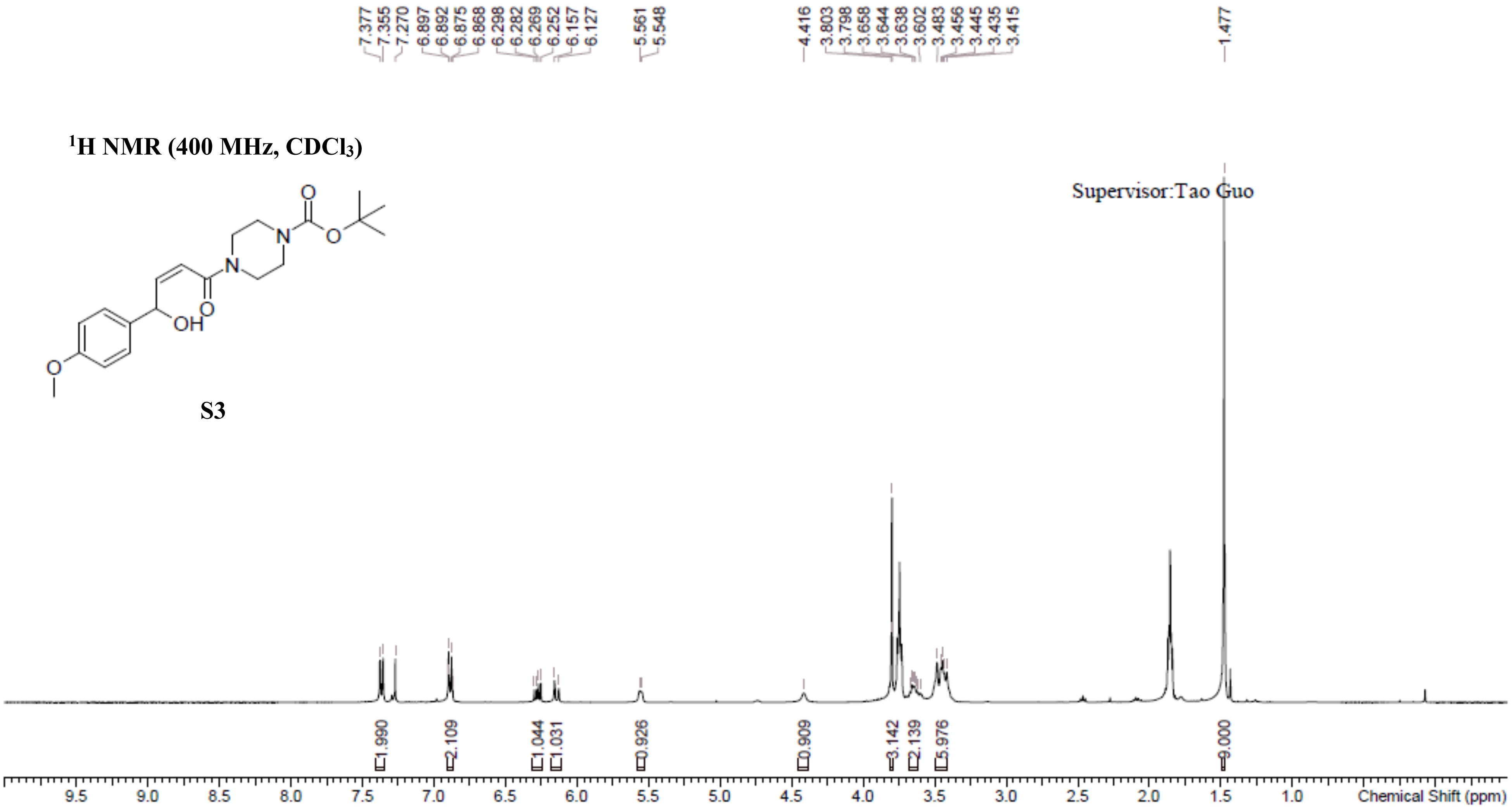

7.377
7.355
7.270
6.897
6.892
6.875
6.868
6.298
6.282
6.269
6.252
6.157
6.127
5.561
5.548
4.416
3.803
3.798
3.658
3.644
3.638
3.602
3.483
3.456
3.445
3.435
3.415
1.477
¹H NMR (400 MHz, CDCl₃)
Supervisor:Tao Guo
S3
1.990
2.109
1.044
1.031
0.926
0.909
3.142
2.139
5.976
9.000
Chemical Shift (ppm)

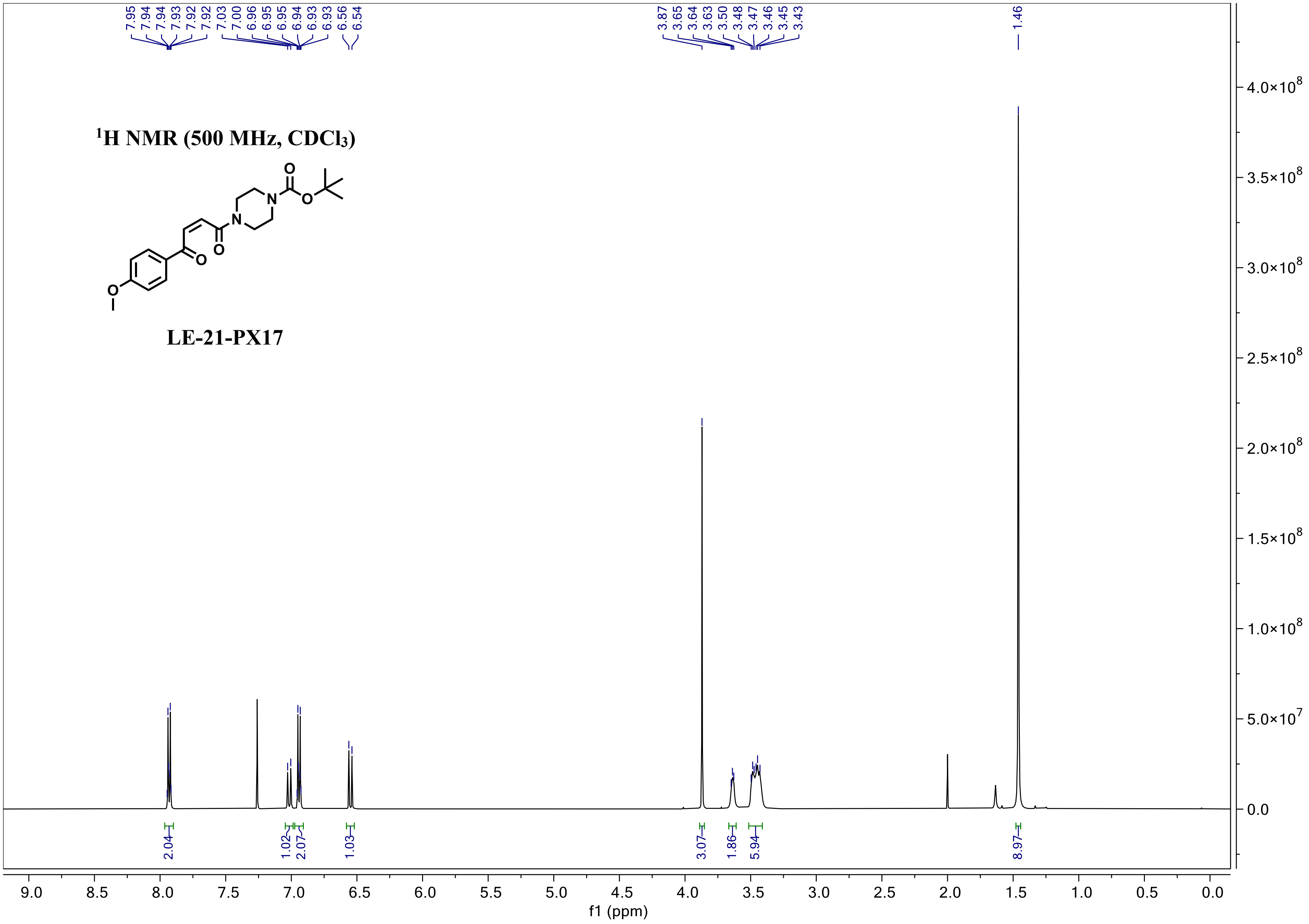
7.95 7.94 7.94 7.93 7.92 7.92 7.03 7.00 6.96 6.95 6.95 6.94 6.93 6.93 6.56 6.54
3.87 3.65 3.64 3.63 3.50 3.48 3.47 3.46 3.45 3.43
1.46
¹H NMR (500 MHz, CDCl₃)
LE-21-PX17
2.04 1.02 2.07 1.03 3.07 1.86 5.94 8.97
f1 (ppm)

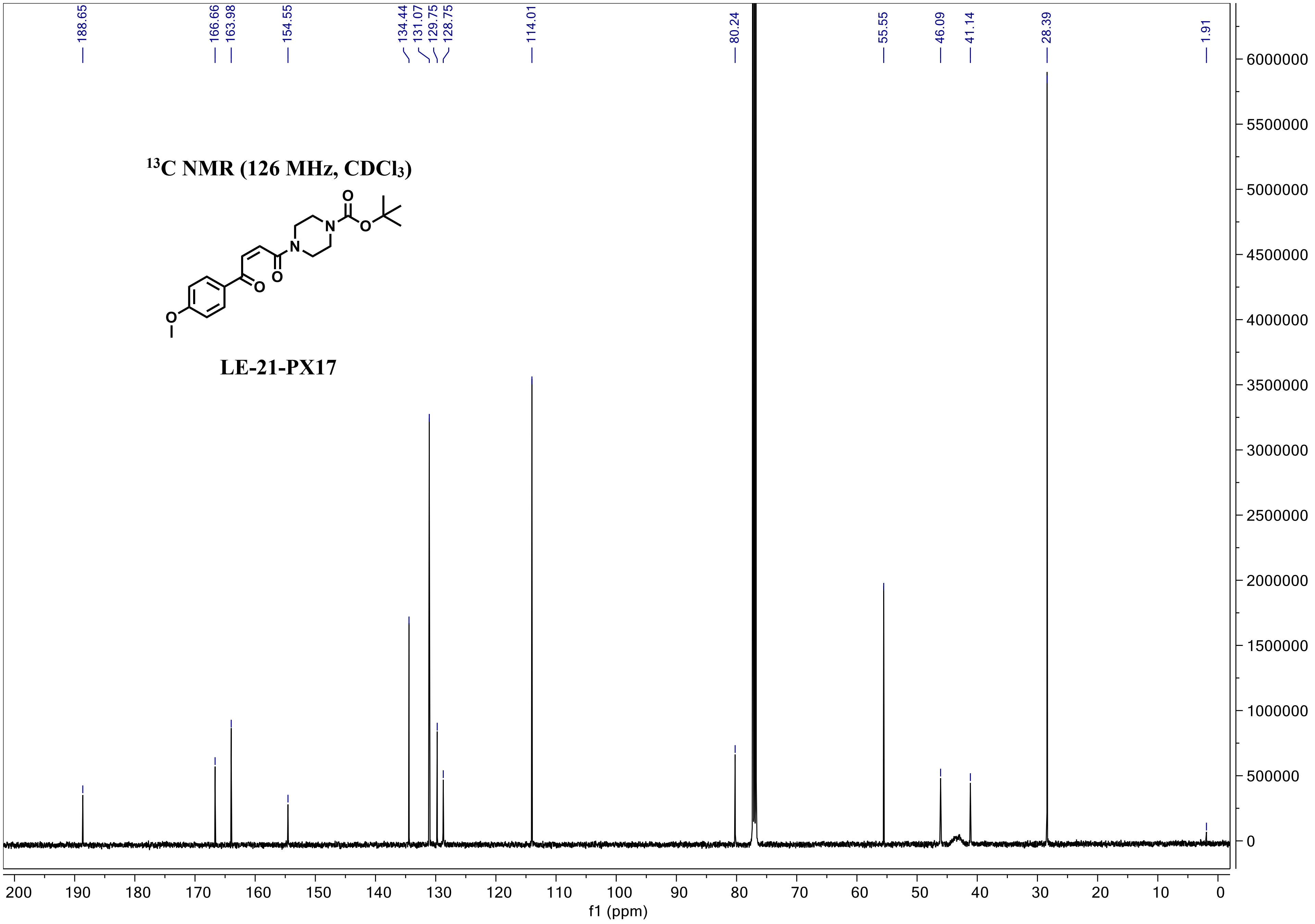

188.65
166.66
163.98
154.55
134.44
131.07
129.75
128.75
114.01
80.24
55.55
46.09
41.14
28.39
1.91
^{13}C NMR (126 MHz, $CDCl_3$)
LE-21-PX17
f1 (ppm)

HPLC=0.1%TFA in H2O/ACN with 1.2mL/min

EM23001597792 | 02-Mar-2023 14:16:04 strubhe1-0302-v03 | LCMS-QDa1 Kinetex column, QDa1_2m_Acidic

PAPATJA1-101-EXP005-S01_Crude

1: Scan ES+
375.184
3.84e6

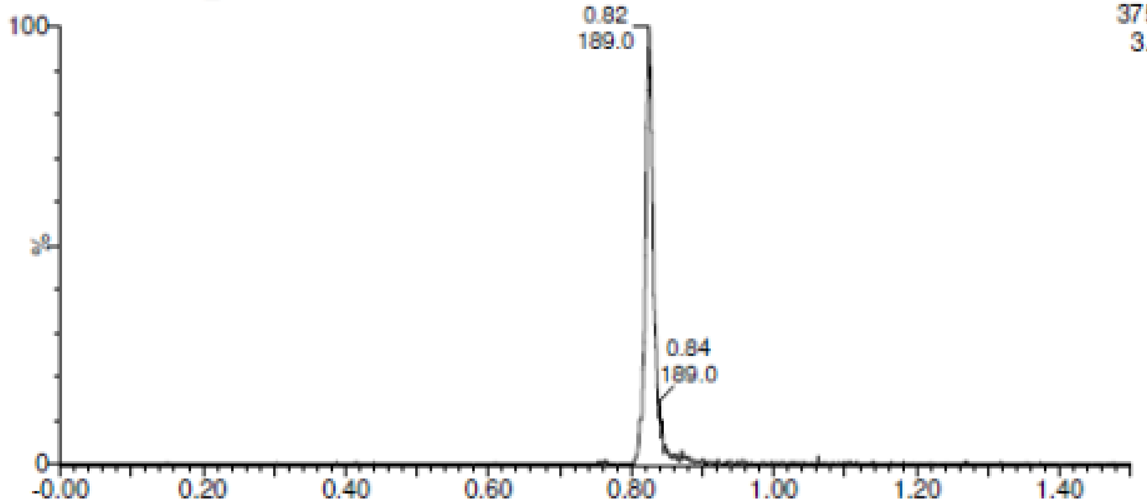

PAPATJA1-101-EXP005-S01_Crude Sm (Mn, 2x3)

2: Diode Array
220
Range: 1.102
Height

Time	Height	Area	Area%
0.6292	3106	39.69	0.28
0.8225	1000911	14135.95	99.06
0.8725	1161	11.73	0.08
1.2167	5729	83.29	0.58

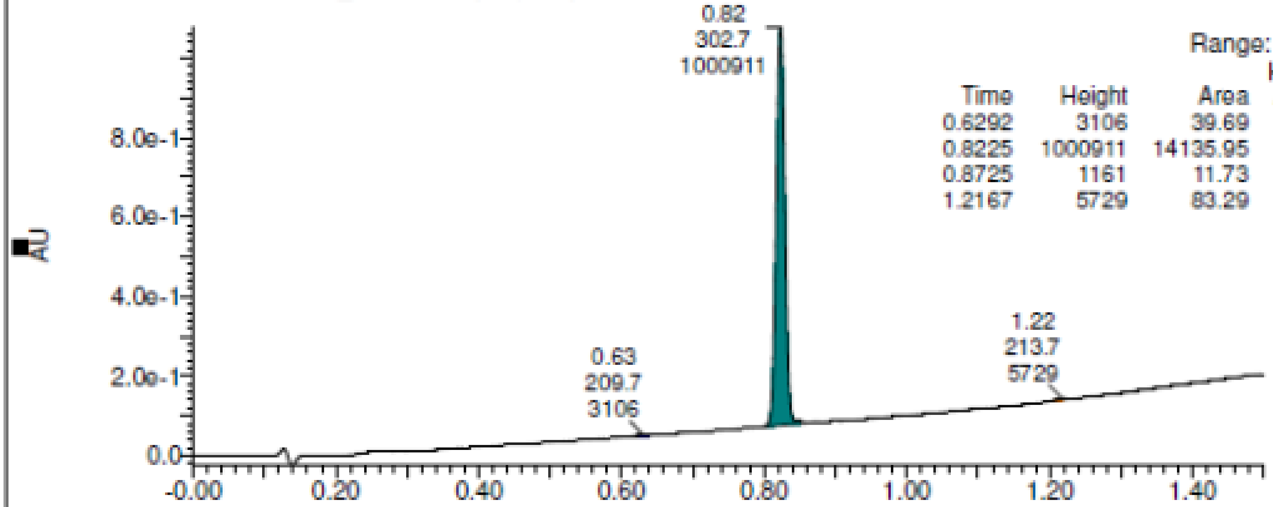

PAPATJA1-101-EXP005-S01_Crude

1: Scan ES+
TIC
1.35e8

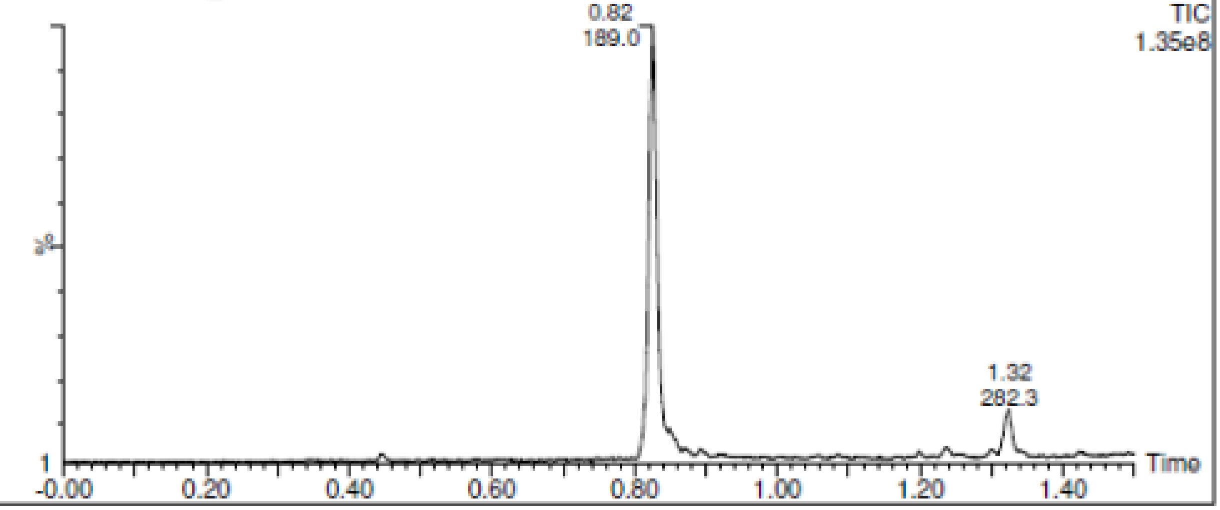

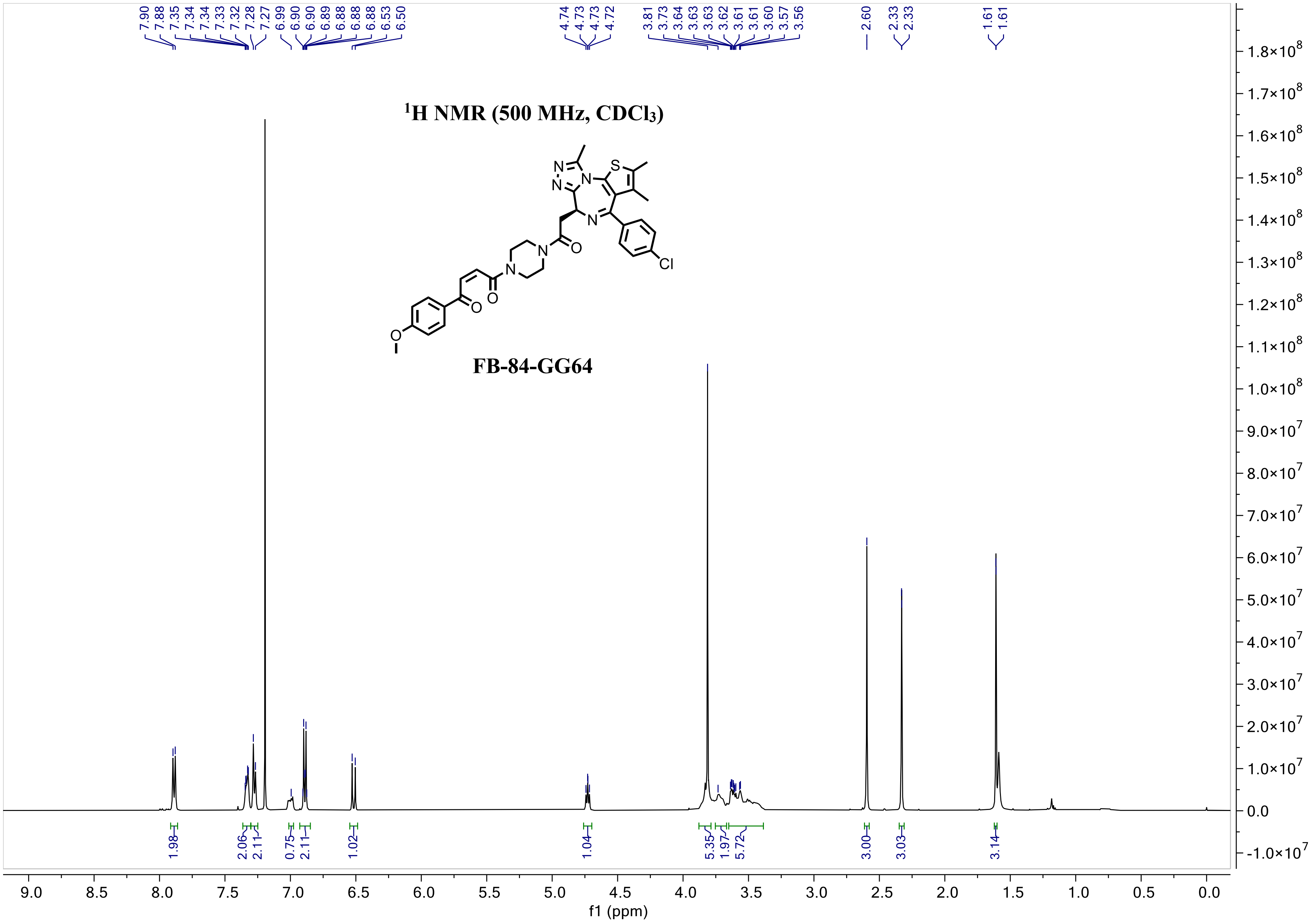

7.90 7.88 7.35 7.34 7.34 7.33 7.32 7.28 7.27 6.99 6.90 6.90 6.89 6.88 6.88 6.88 6.53 6.50
4.74 4.73 4.73 4.72
3.81 3.73 3.64 3.63 3.63 3.62 3.61 3.61 3.60 3.57 3.56
2.60 2.33 2.33
1.61 1.61
1H NMR (500 MHz, CDCl3)
N
N
N
S
N
O
N
N
O
O
O
Cl
FB-84-GG64
1.98 2.06 2.11 0.75 2.11 1.02 1.04 5.35 1.97 5.72 3.00 3.03 3.14
9.0 8.5 8.0 7.5 7.0 6.5 6.0 5.5 5.0 4.5 4.0 3.5 3.0 2.5 2.0 1.5 1.0 0.5 0.0
f1 (ppm)

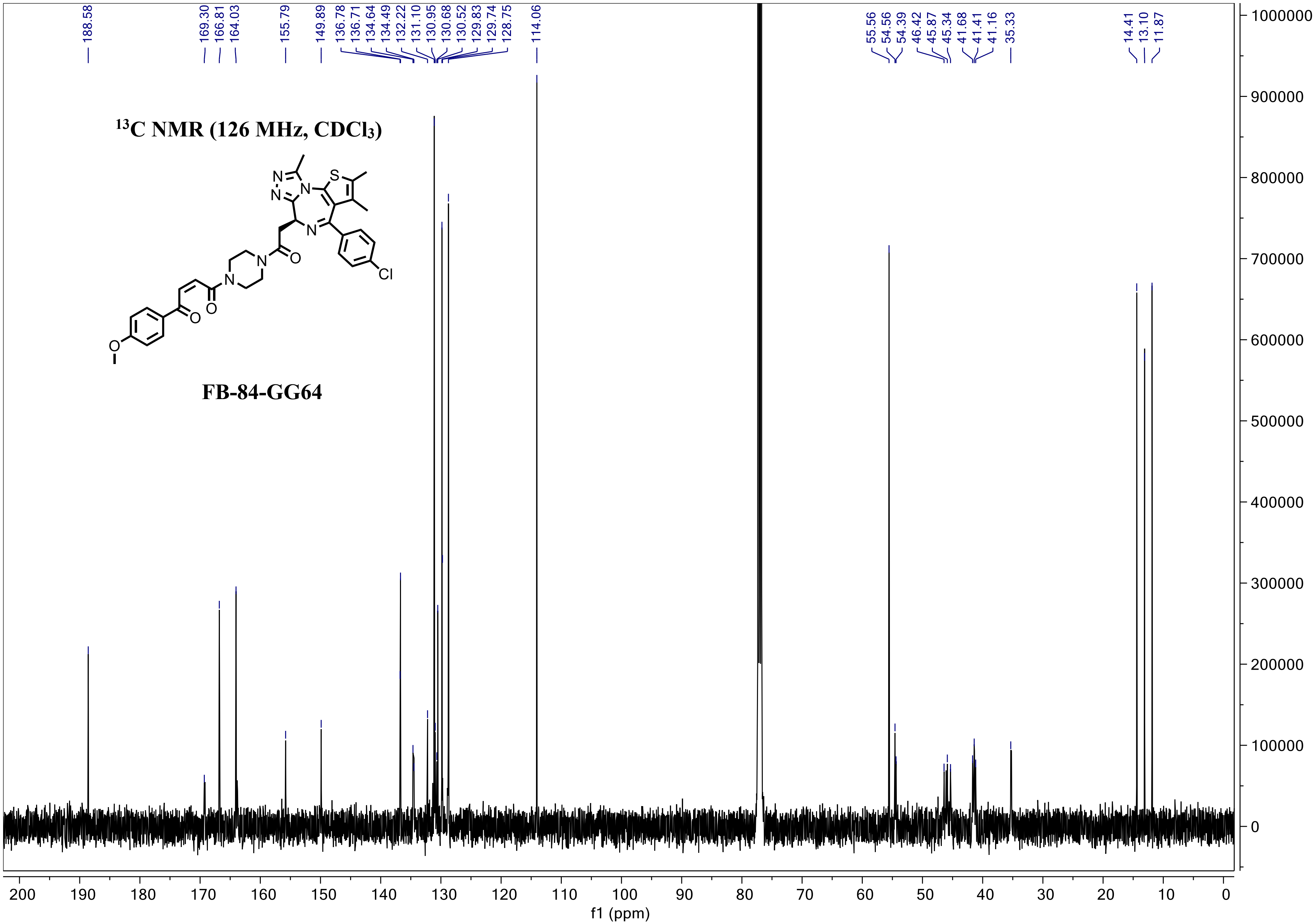

188.58
169.30
166.81
164.03
155.79
149.89
136.78
136.71
134.64
134.49
132.22
131.10
130.95
130.68
130.52
129.83
129.74
128.75
114.06
55.56
54.56
54.39
46.42
45.87
45.34
41.68
41.41
41.16
35.33
14.41
13.10
11.87
13C NMR (126 MHz, CDCl3)
FB-84-GG64
f1 (ppm)

HPLC=0.1%TFA in H2O/ACN with 1.2mL/min

**EM23001597796**	**02-Mar-2023**	**LCMS-QDa1**
	**14:18:33**	**Kinetex column,**
	**strubhe1-0302-v04**	**QDa1_2m_Acidic**

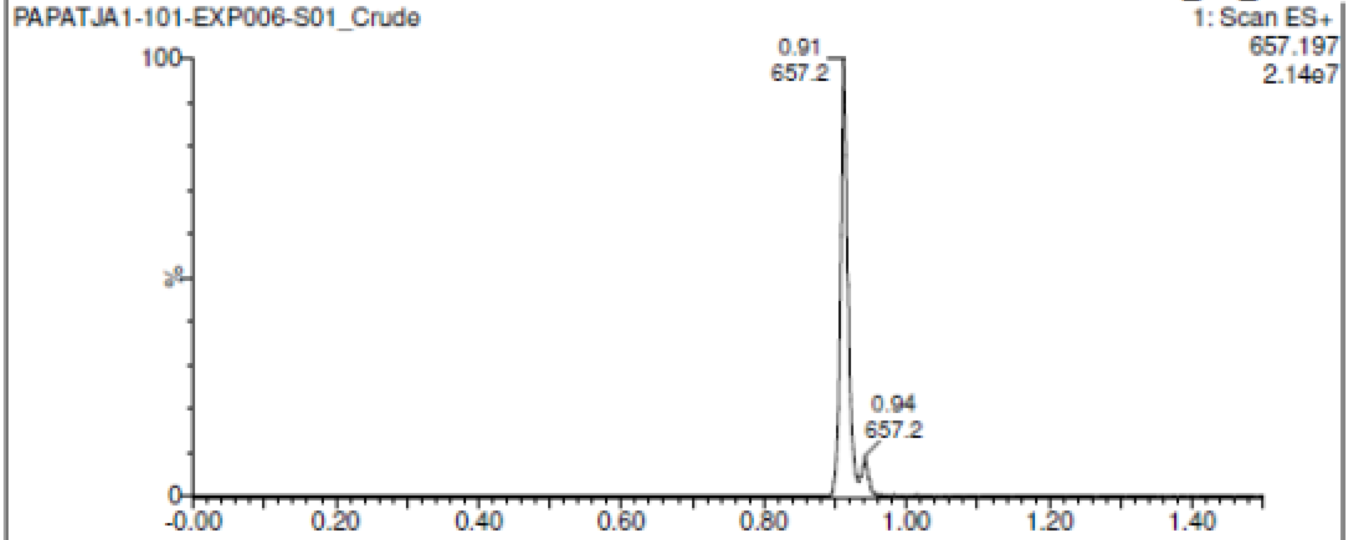

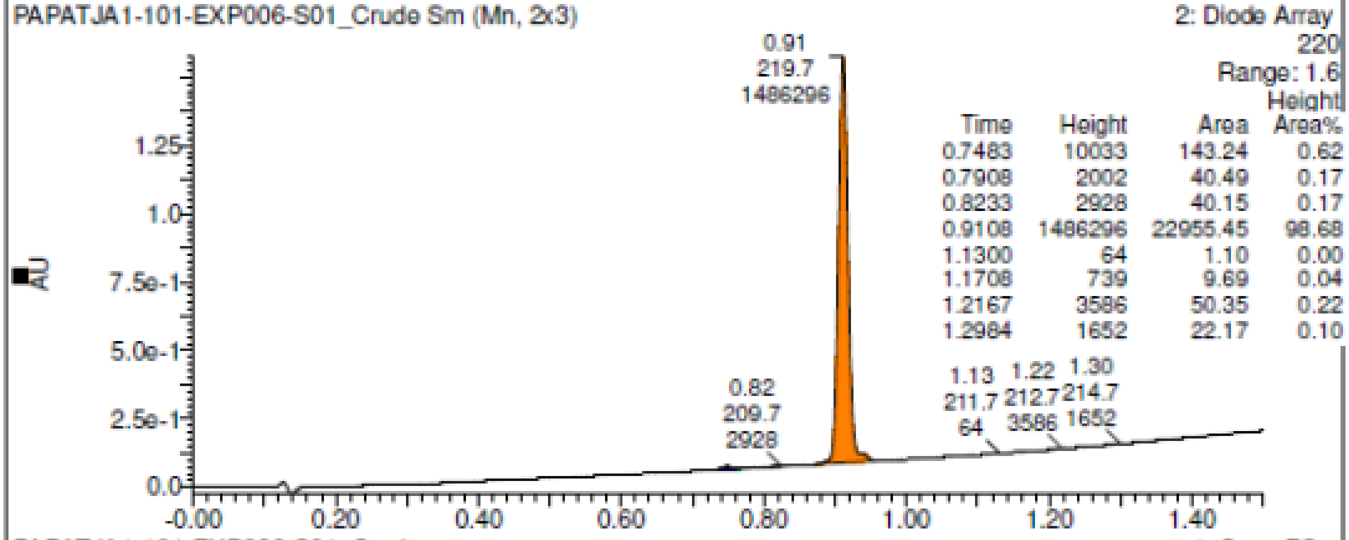

Time	Height	Area	Area%
0.7483	10033	143.24	0.62
0.7908	2002	40.49	0.17
0.8233	2928	40.15	0.17
0.9108	1486296	22955.45	98.68
1.1300	64	1.10	0.00
1.1708	739	9.69	0.04
1.2167	3586	50.35	0.22
1.2984	1652	22.17	0.10

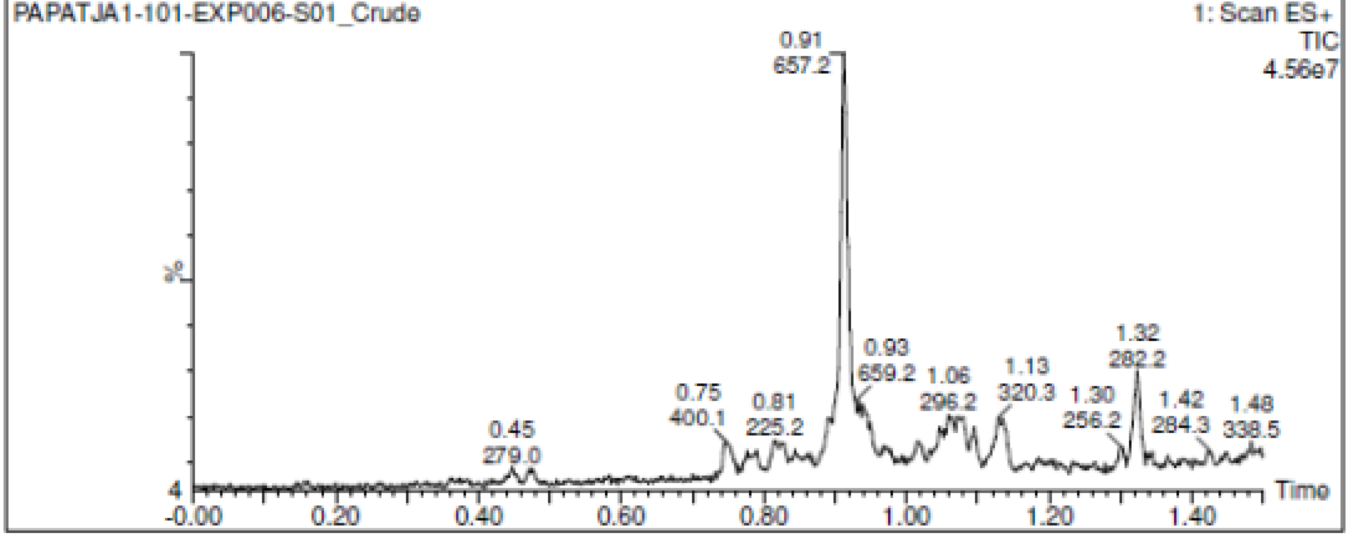

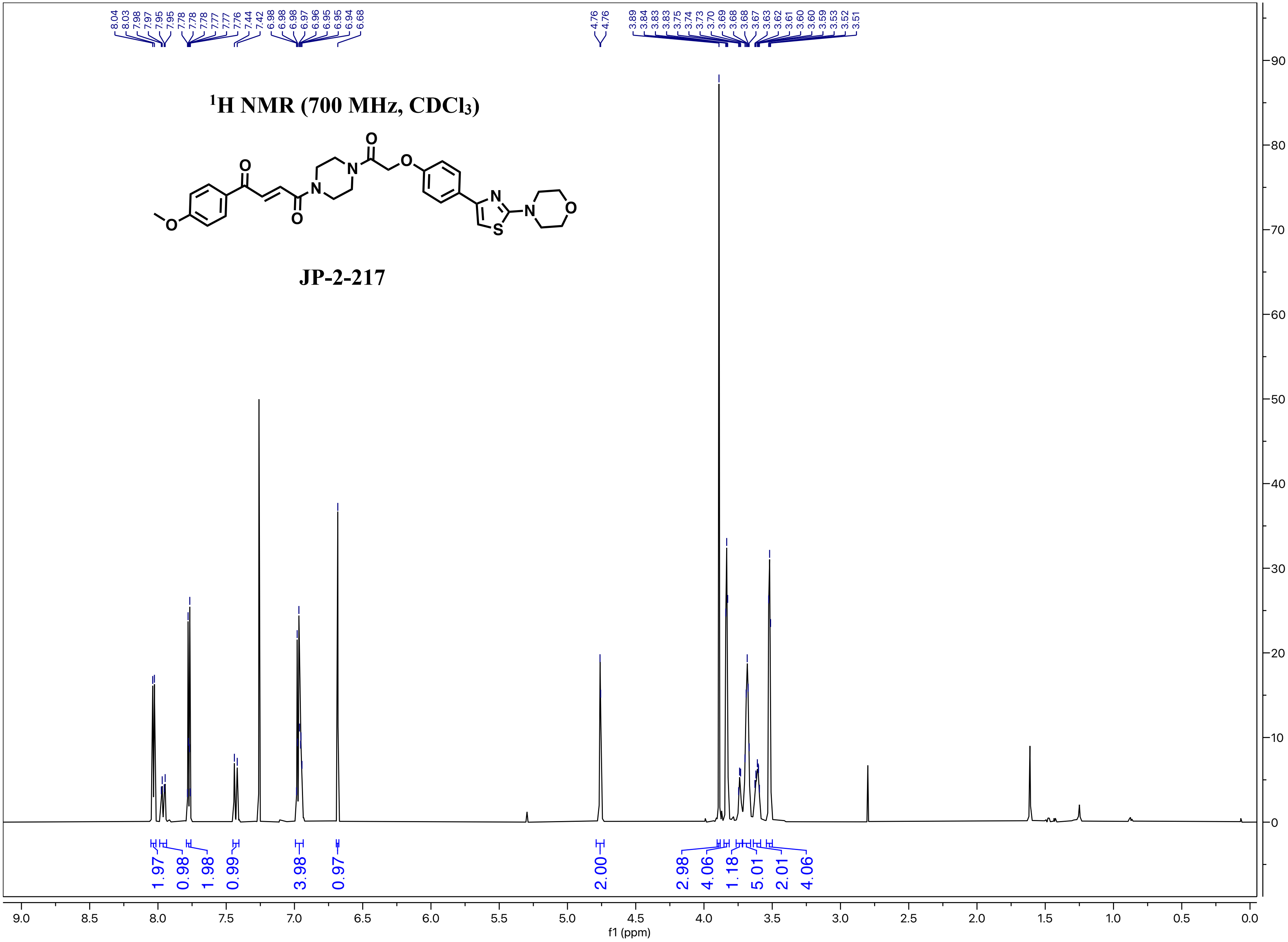

1H NMR (700 MHz, CDCl3)
JP-2-217
8.04 8.03 7.98 7.97 7.95 7.95 7.78 7.78 7.78 7.77 7.77 7.76 7.44 7.42 6.98 6.98 6.97 6.96 6.95 6.95 6.94 6.68
4.76 4.76
3.89 3.84 3.83 3.83 3.75 3.74 3.73 3.70 3.69 3.68 3.68 3.67 3.63 3.62 3.61 3.60 3.60 3.59 3.53 3.52 3.51
1.97 0.98 1.98 0.99 3.98 0.97 2.00 2.98 4.06 1.18 5.01 2.01 4.06
f1 (ppm)

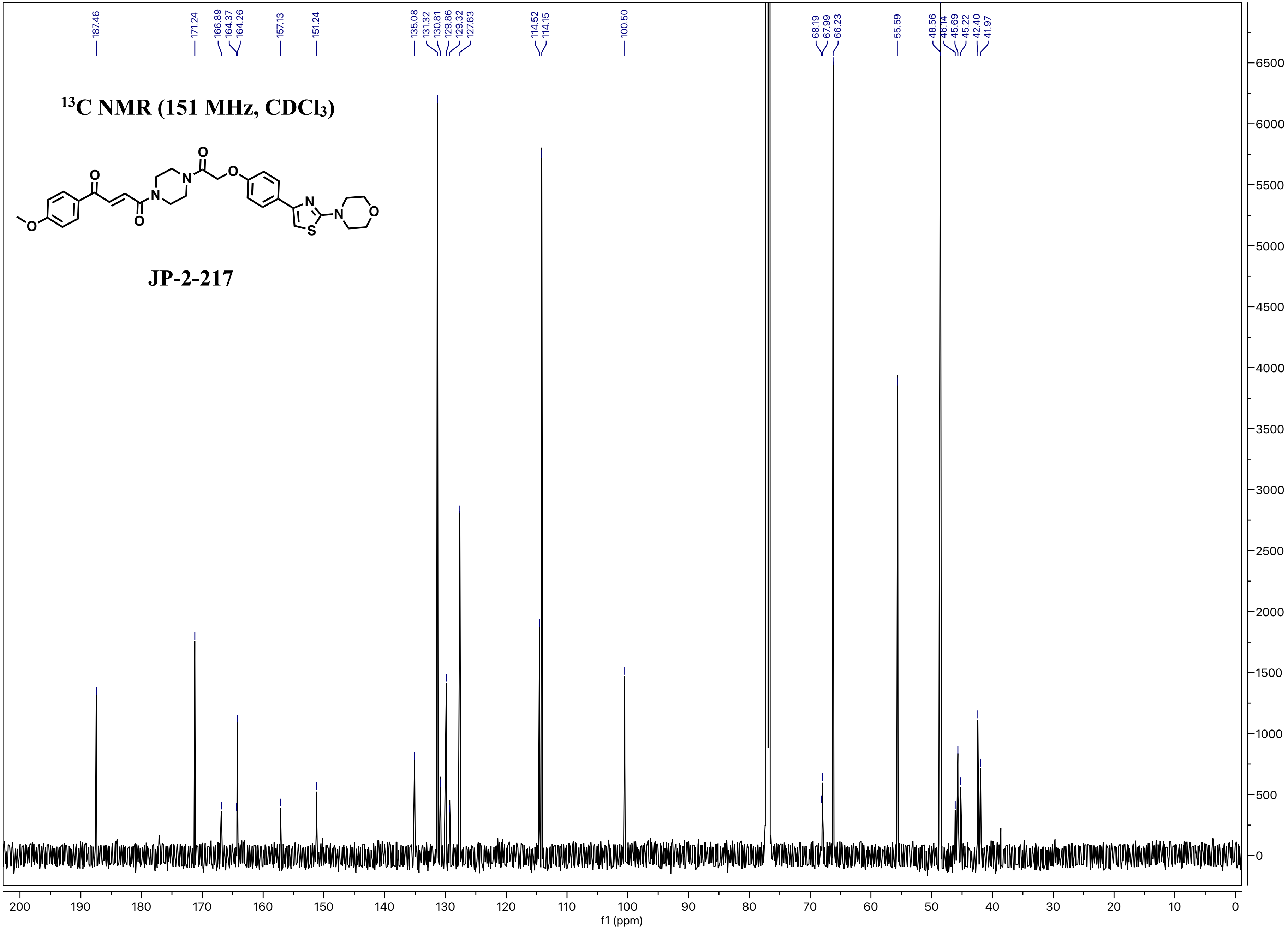

13C NMR (151 MHz, CDCl3)
JP-2-217
187.46
171.24
166.89
164.37
164.26
157.13
151.24
135.08
131.32
130.81
129.86
129.32
127.63
114.52
114.15
100.50
68.19
67.99
66.23
55.59
48.56
46.14
45.69
45.22
42.40
41.97
f1 (ppm)

HPLC=0.1%TFA in H2O/ACN with 1.2mL/min

EM23001590293 | 13-Jan-2023 16:16:48 | strubhe1-0112A-v07 | LCMS-ZQ1 Kinetex column, ZQ1_10m_Acidic

HESSEMA4-101-EXP210-S01_QC — 1: Scan ES+ 577.204 6.61e7

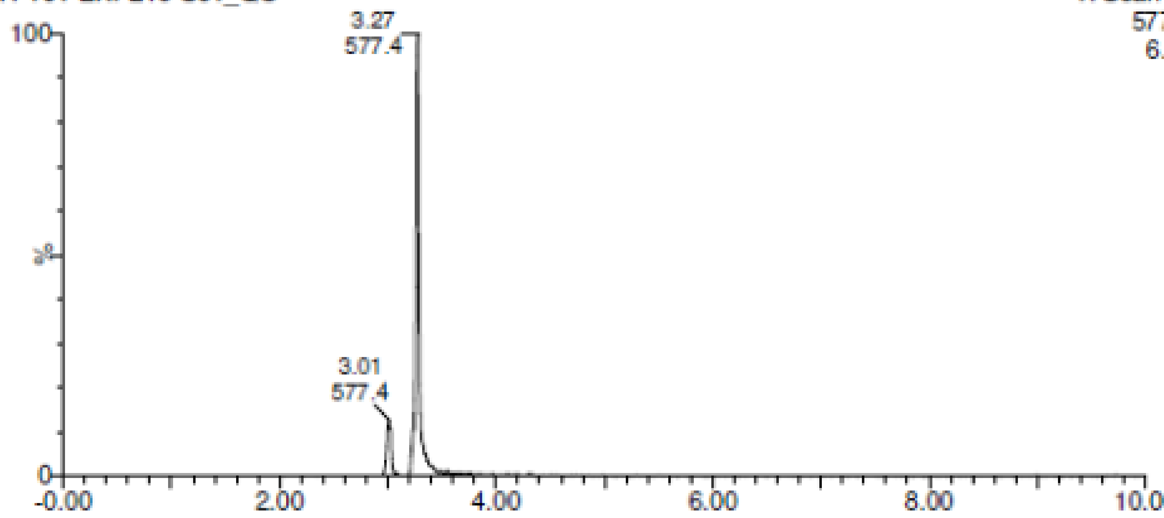

HESSEMA4-101-EXP210-S01_QC Sm (Mn, 2x3) — 2: Diode Array 220 Range: 1.497

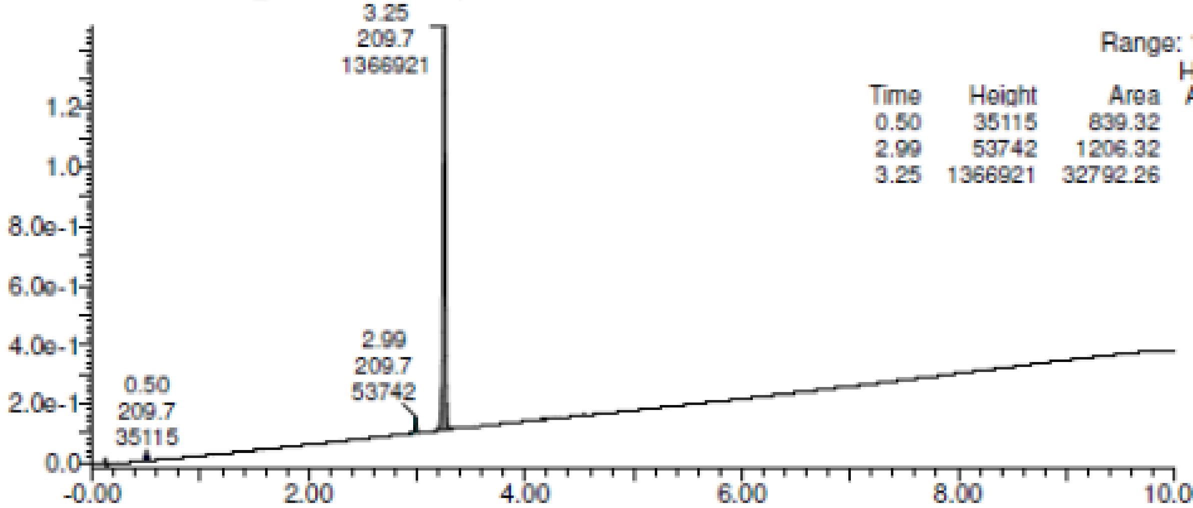

Time	Height	Area	Height Area%
0.50	35115	839.32	2.41
2.99	53742	1206.32	3.46
3.25	1366921	32792.26	94.13

HESSEMA4-101-EXP210-S01_QC — 1: Scan ES+ TIC 1.09e8

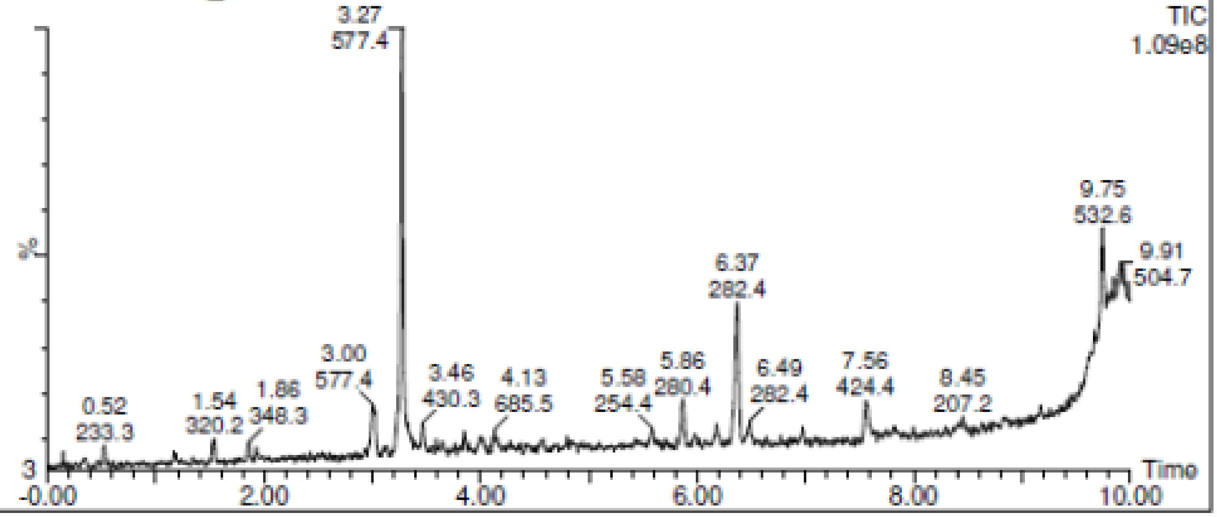

HPLC=0.1%TFA in H2O/ACN with 1.2mL/min

EM23001590293 | **19-Jan-2023 16:42:45 strubhe1-0118A-v07** | **LCMS-ZQ1 Kinetex column, ZQ1_10m_Acidic**

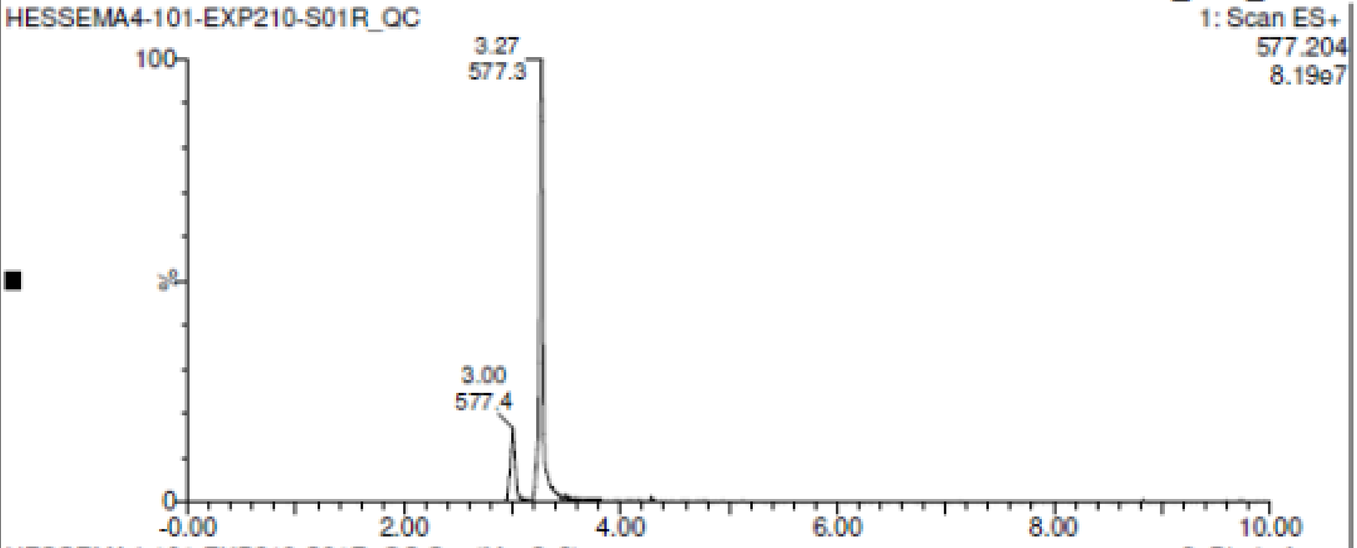

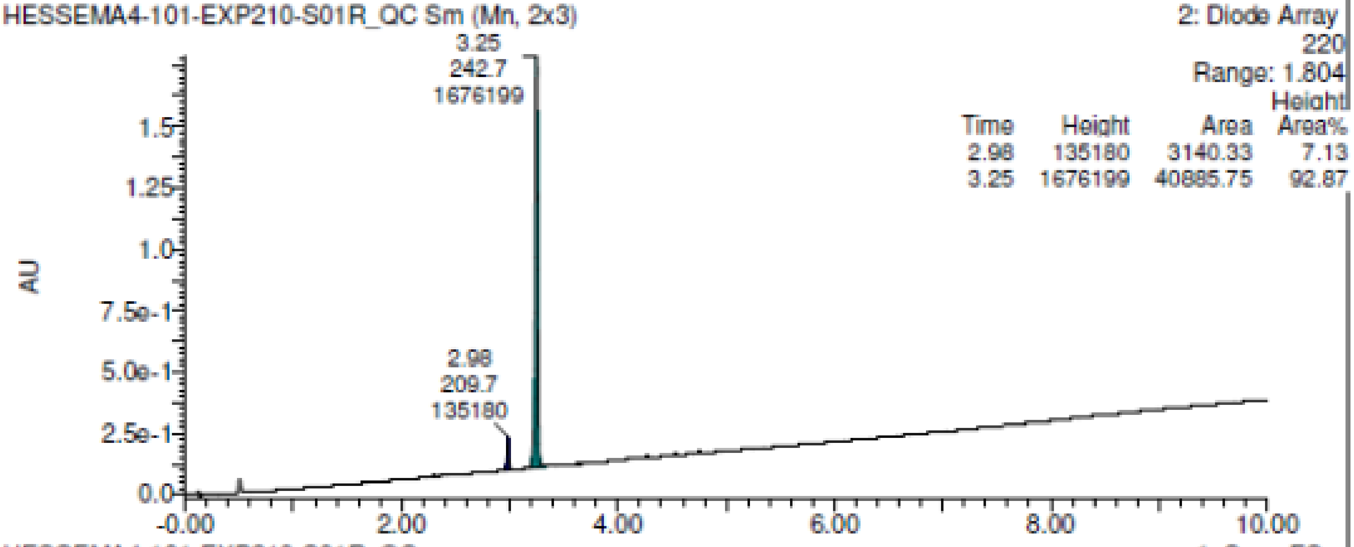

Time	Height	Area	Height Area%
2.98	135180	3140.33	7.13
3.25	1676199	40885.75	92.87

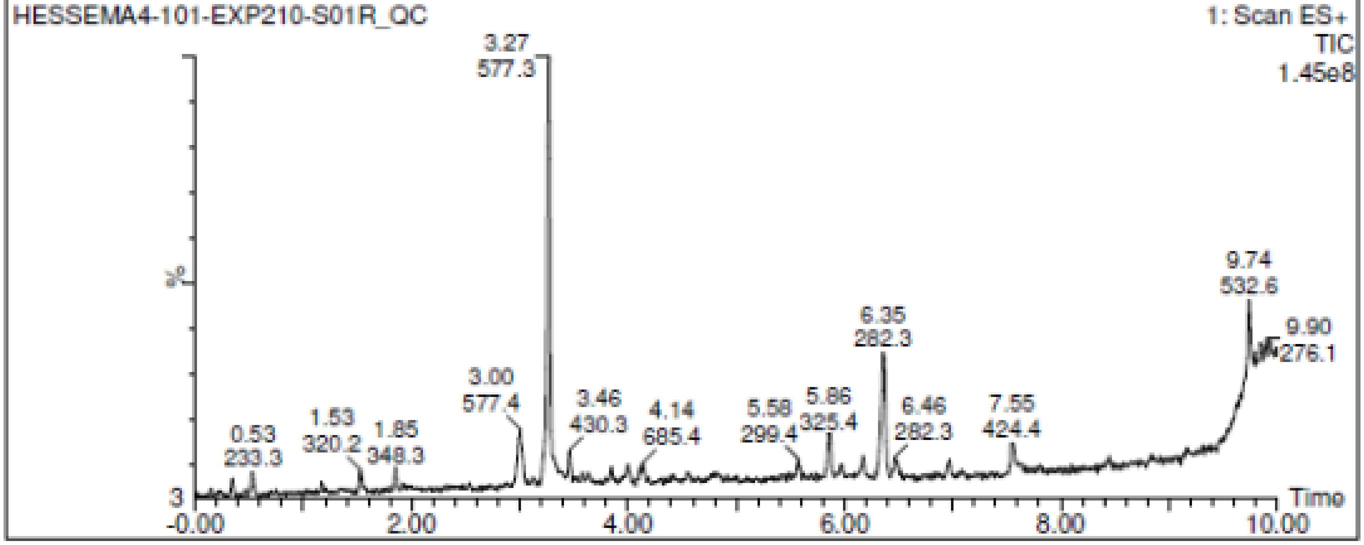

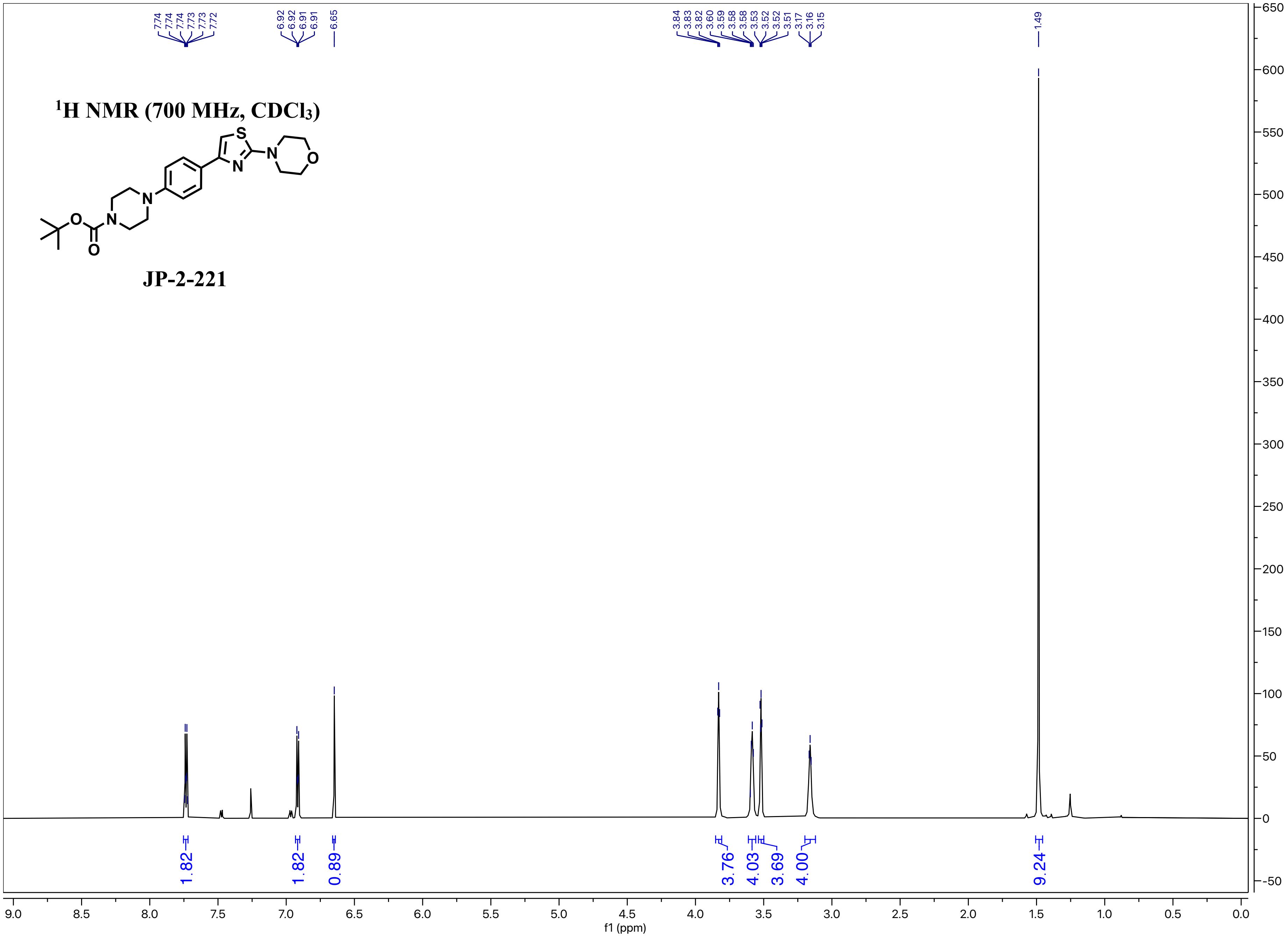

1H NMR (700 MHz, CDCl3)
JP-2-221
7.74
7.74
7.74
7.73
7.73
7.72
6.92
6.92
6.91
6.91
6.65
3.84
3.83
3.82
3.60
3.59
3.58
3.58
3.53
3.52
3.52
3.51
3.17
3.16
3.15
1.49
1.82
1.82
0.89
3.76
4.03
3.69
4.00
9.24
f1 (ppm)

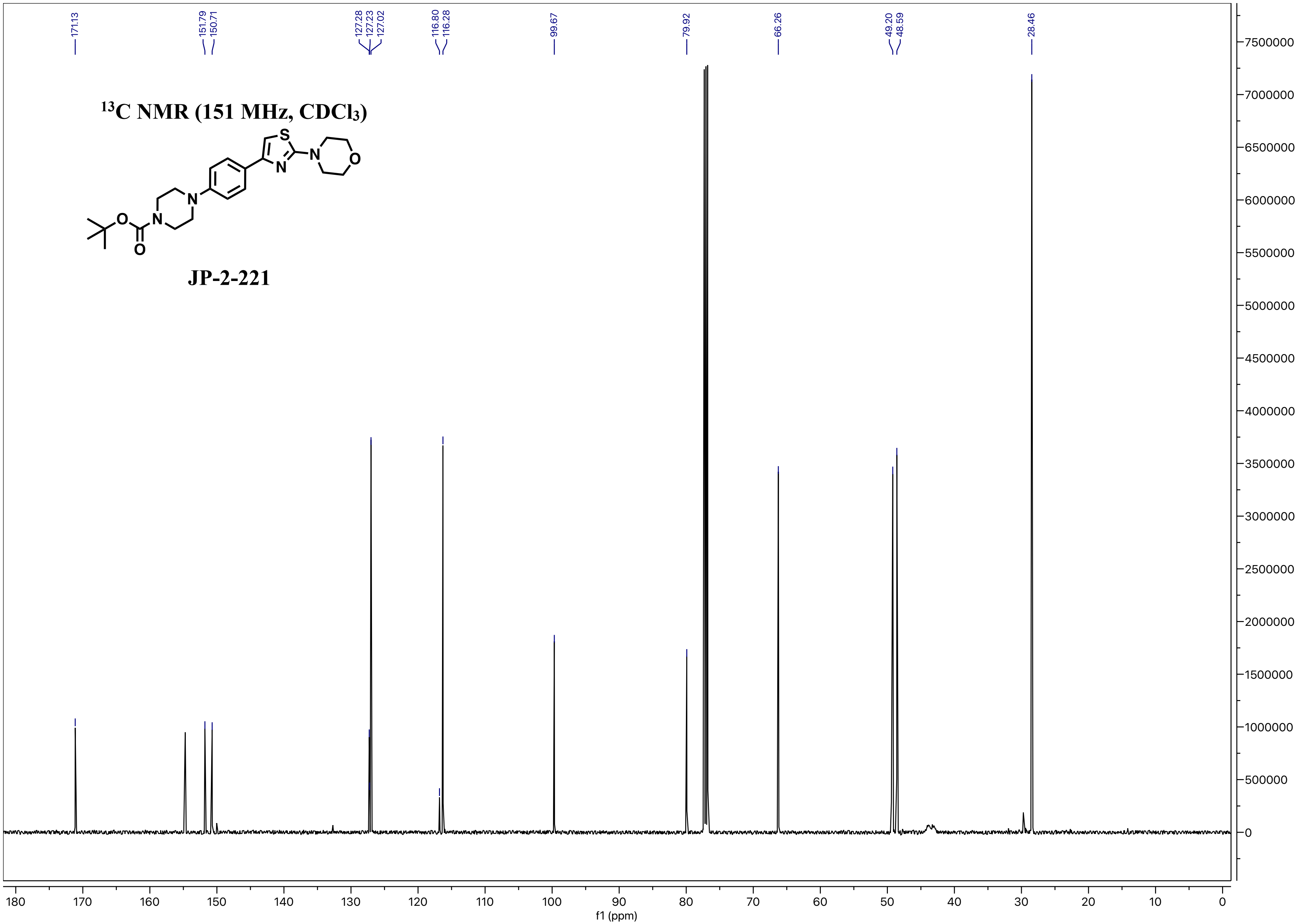

^{13}C NMR (151 MHz, $CDCl_3$)
JP-2-221
S
N
N
O
N
N
O
O
171.13
151.79
150.71
127.28
127.23
127.02
116.80
116.28
99.67
79.92
66.26
49.20
48.59
28.46
f1 (ppm)

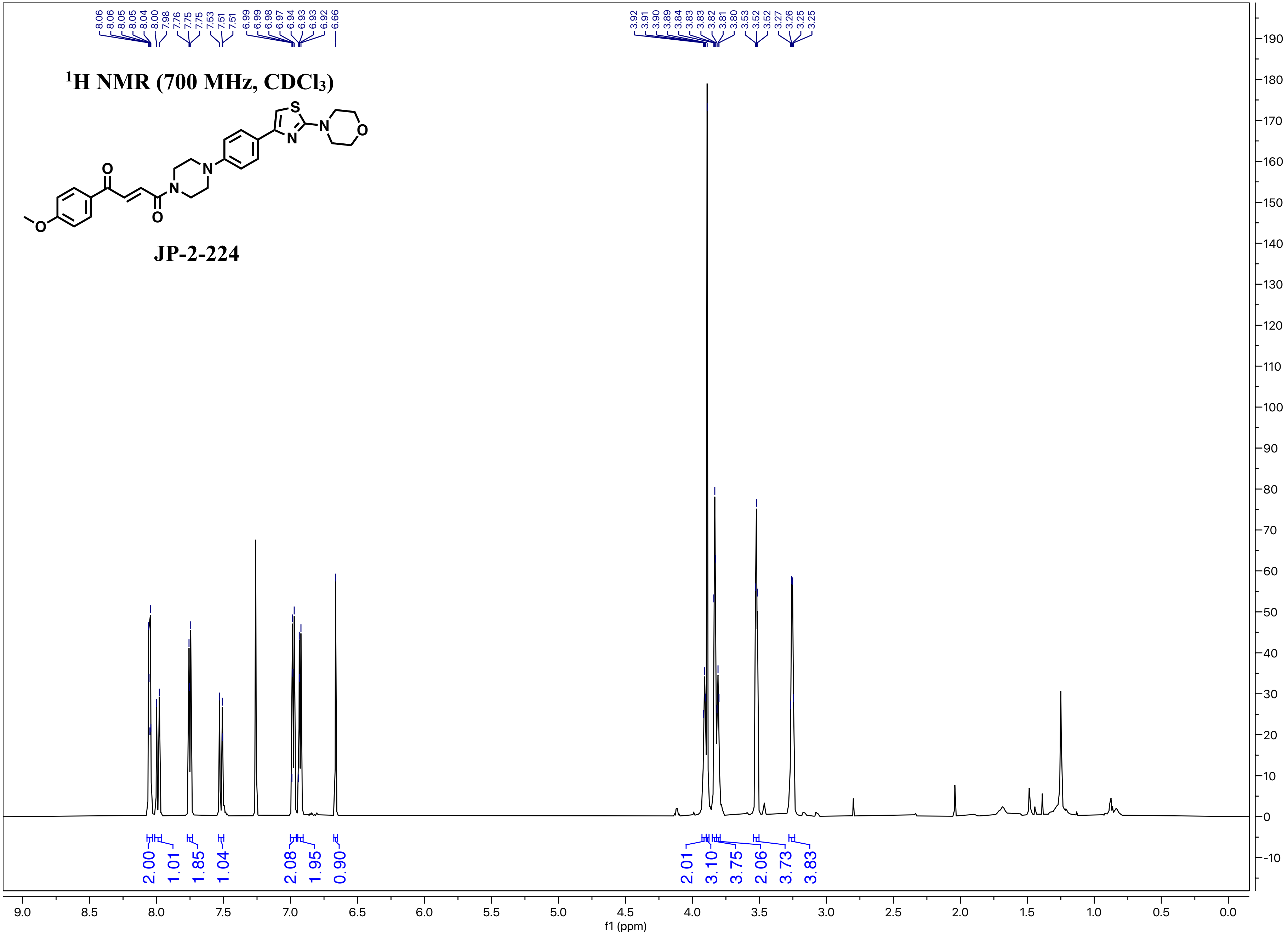

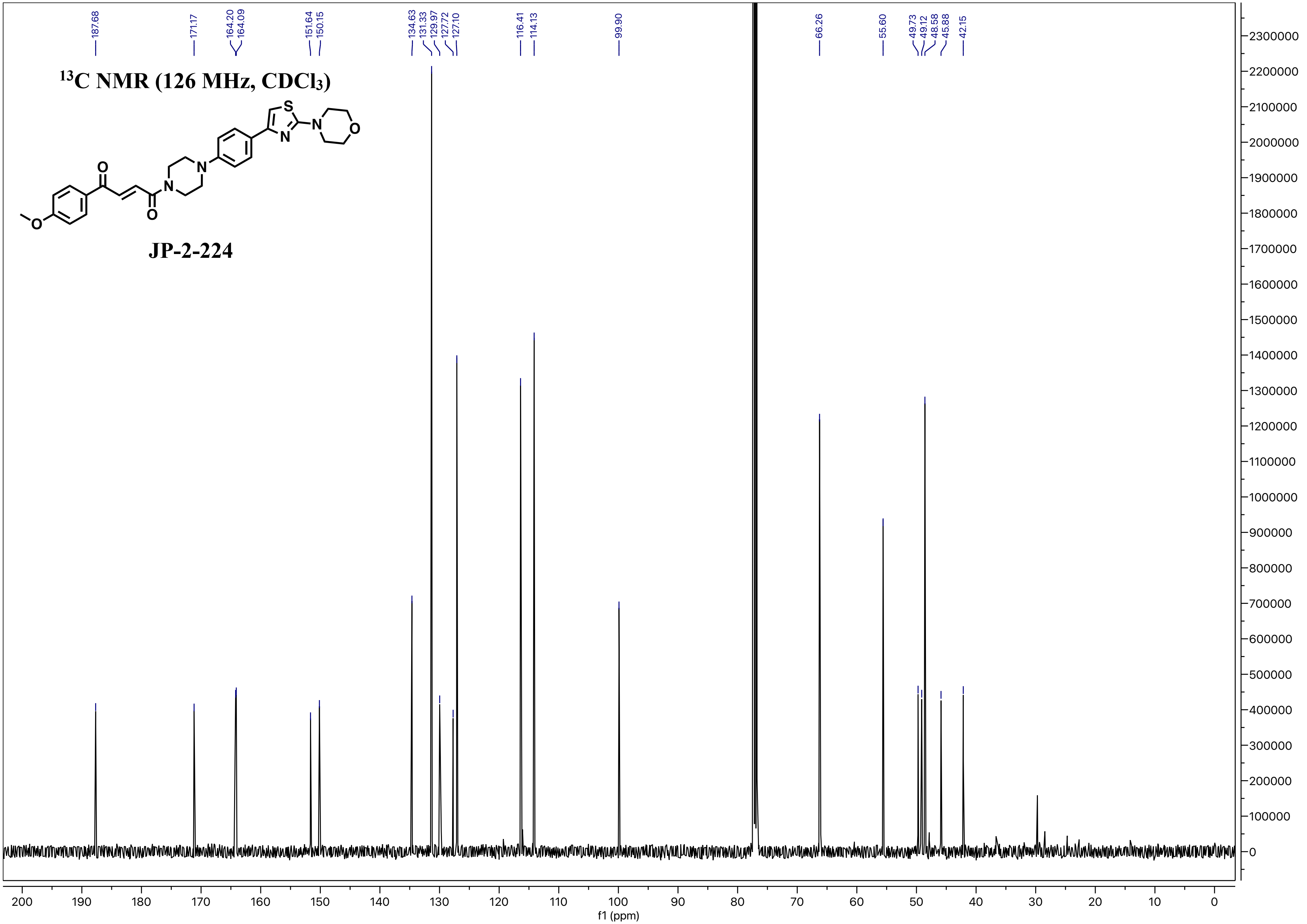

187.68
171.17
164.20
164.09
151.64
150.15
134.63
131.33
129.97
127.72
127.10
116.41
114.13
99.90
66.26
55.60
49.73
49.12
48.58
45.88
42.15
13C NMR (126 MHz, CDCl3)
JP-2-224
f1 (ppm)

HPLC=0.1%TFA in H2O/ACN with 1.2mL/min

EM23001590285 | **13-Jan-2023 15:54:05 strubhe1-0112A-v03** | **LCMS-ZQ1 Kinetex column, ZQ1_10m_Acidic**

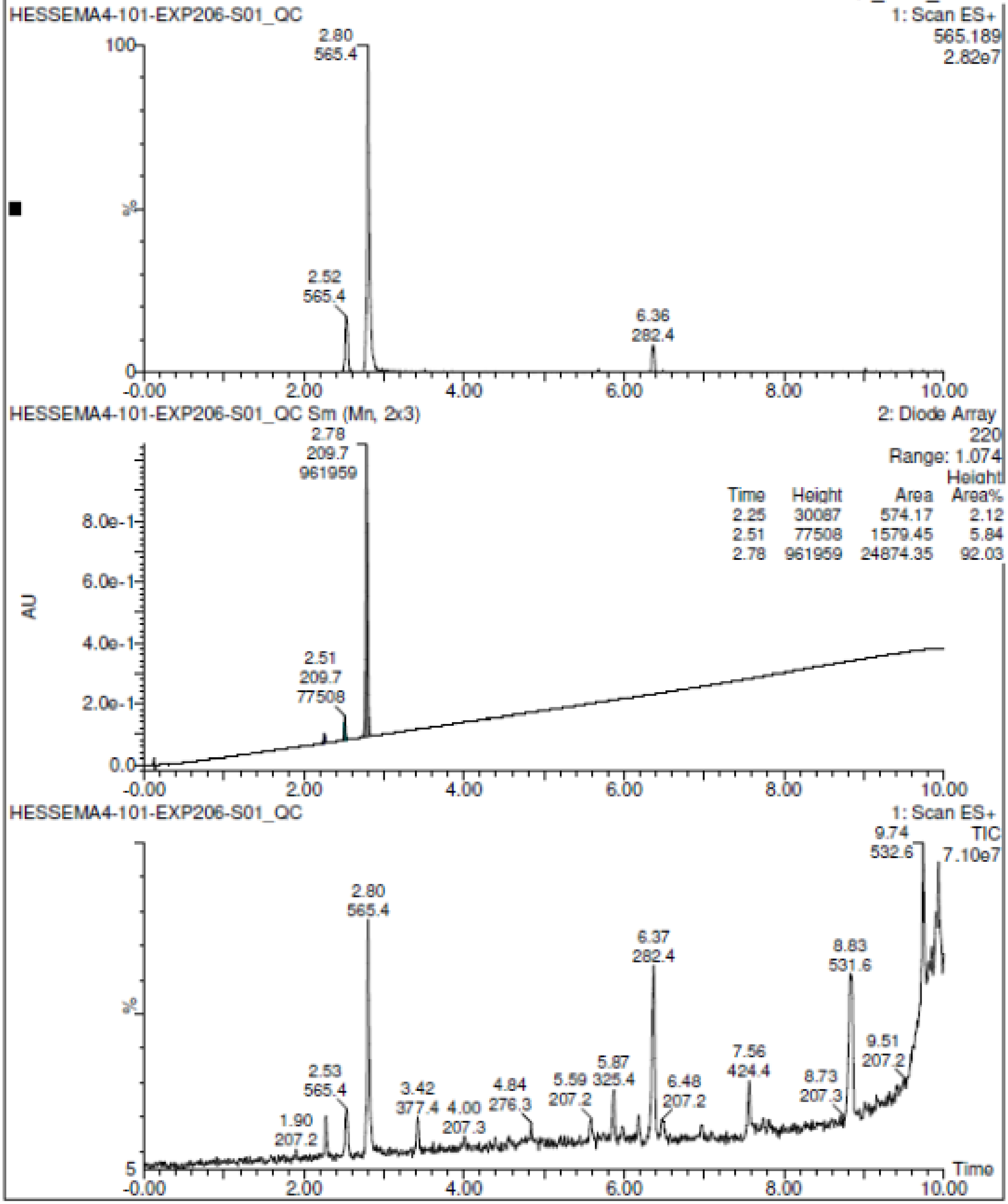

Time	Height	Area	Height Area%
2.25	30087	574.17	2.12
2.51	77508	1579.45	5.84
2.78	961959	24874.35	92.03

HPLC=0.1%TFA in H2O/ACN with 1.2mL/min

**EM23001590291**	**19-Jan-2023**<br>**16:20:41**<br>**strubhe1-0118A-v05**	**LCMS-ZQ1**<br>**Kinetex column,**<br>**ZQ1_10m_Acidic**

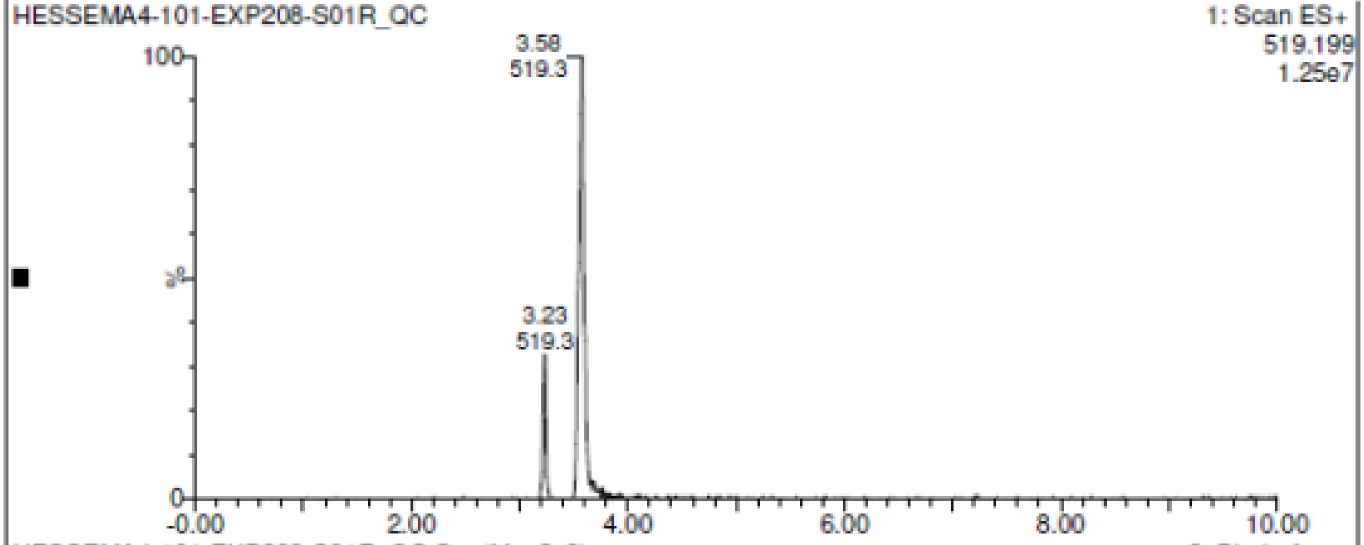

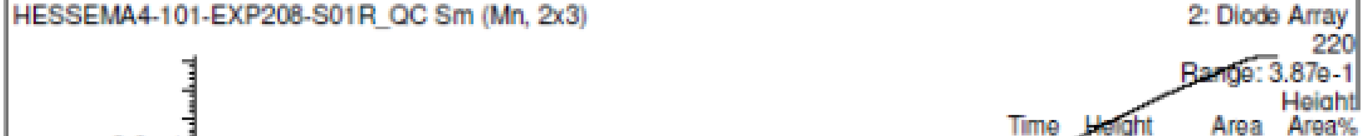

Time	Height	Area	Height Area%
2.63	2232	60.57	2.40
2.78	7688	156.87	6.22
2.81	32347	922.12	36.57
3.21	1759	38.86	1.54
3.55	39191	1021.53	40.51
4.12	1245	28.32	1.12
4.19	2757	87.81	3.48
4.66	6132	158.08	6.27
5.71	1642	47.27	1.87

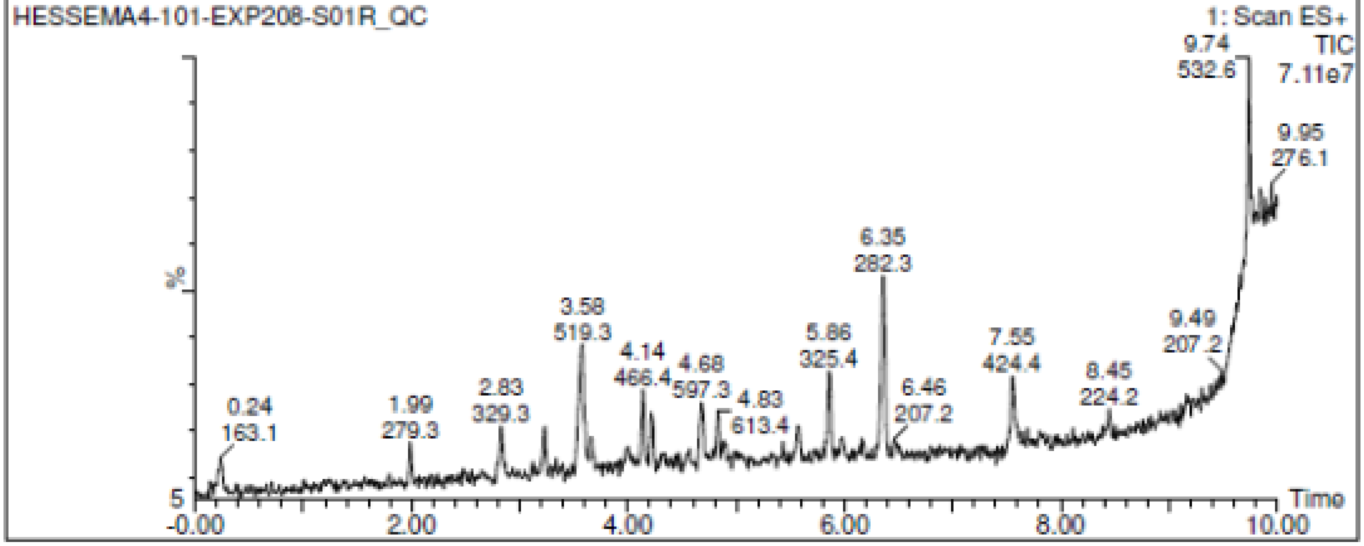

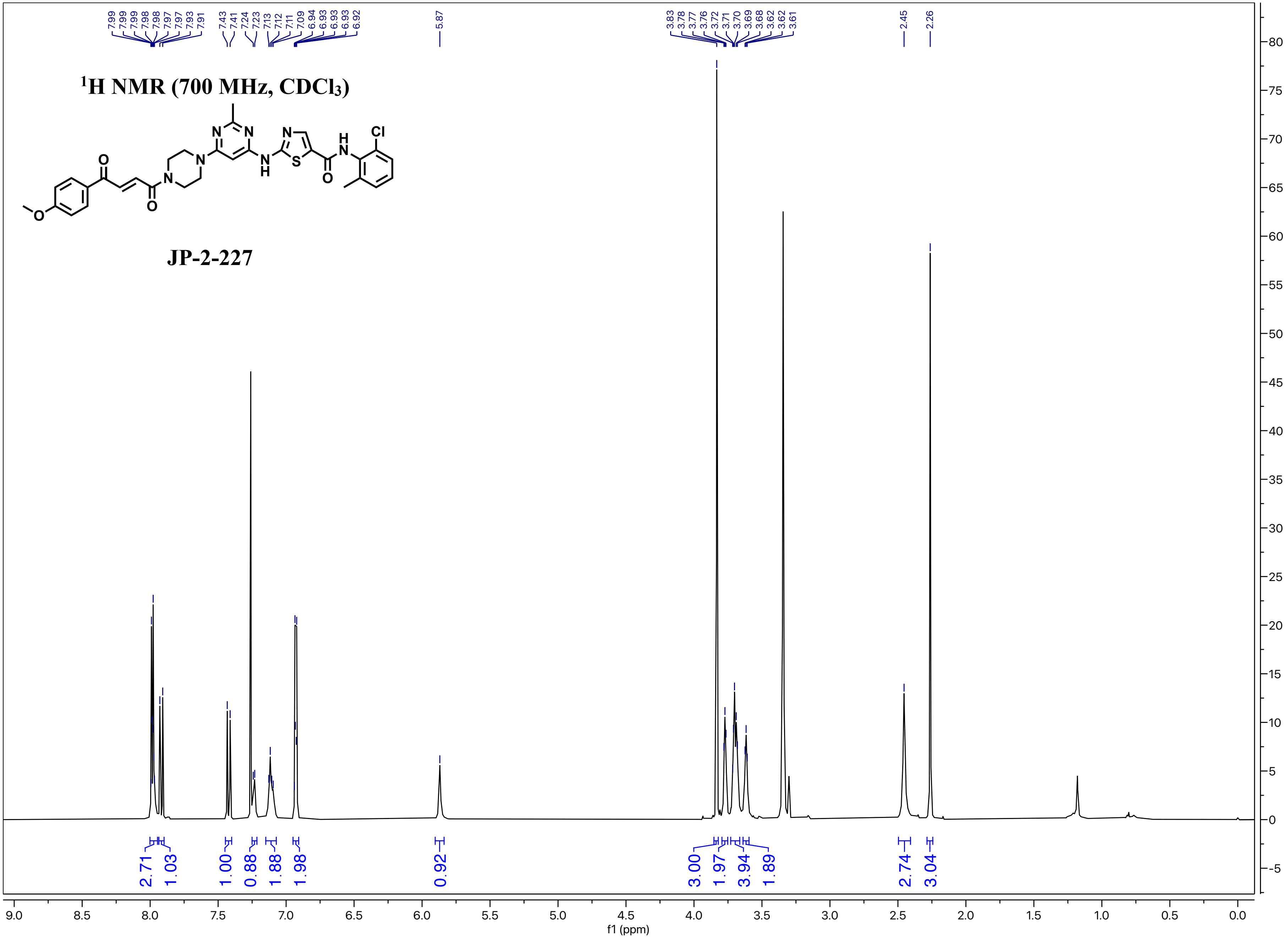

¹H NMR (700 MHz, CDCl₃)
JP-2-227
f1 (ppm)

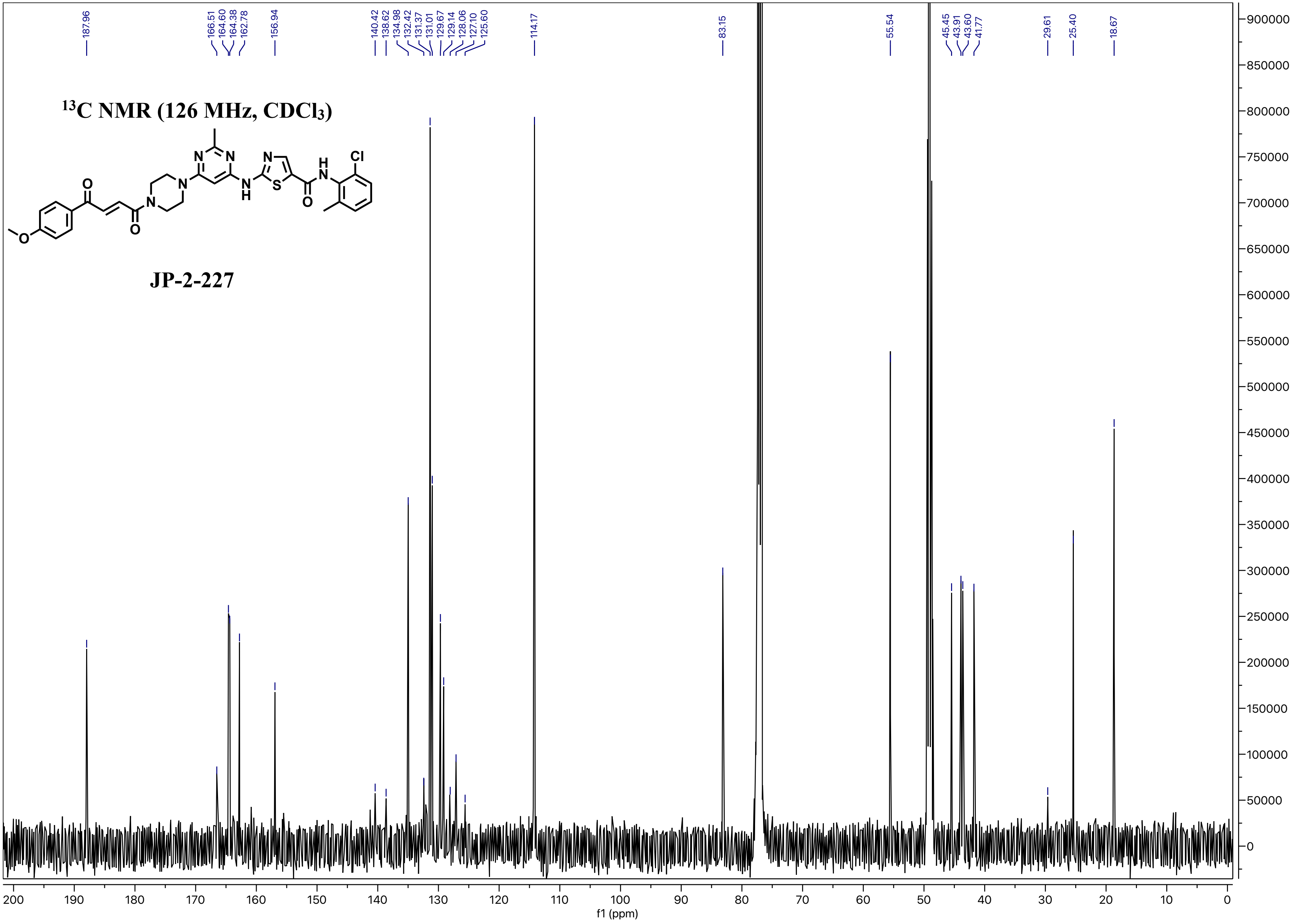

^{13}C NMR (126 MHz, CDCl$_3$)
JP-2-227
187.96
166.51
164.60
164.38
162.78
156.94
140.42
138.62
134.98
132.42
131.37
131.01
129.67
129.14
128.06
127.10
125.60
114.17
83.15
55.54
45.45
43.91
43.60
41.77
29.61
25.40
18.67
f1 (ppm)

HPLC=0.1%TFA in H2O/ACN with 1.2mL/min

EM23001590289

13-Jan-2023
15:30:39
strubhe1-0112A-v04

LCMS-ZQ1
Kinetex column,
ZQ1_10m_Acidic

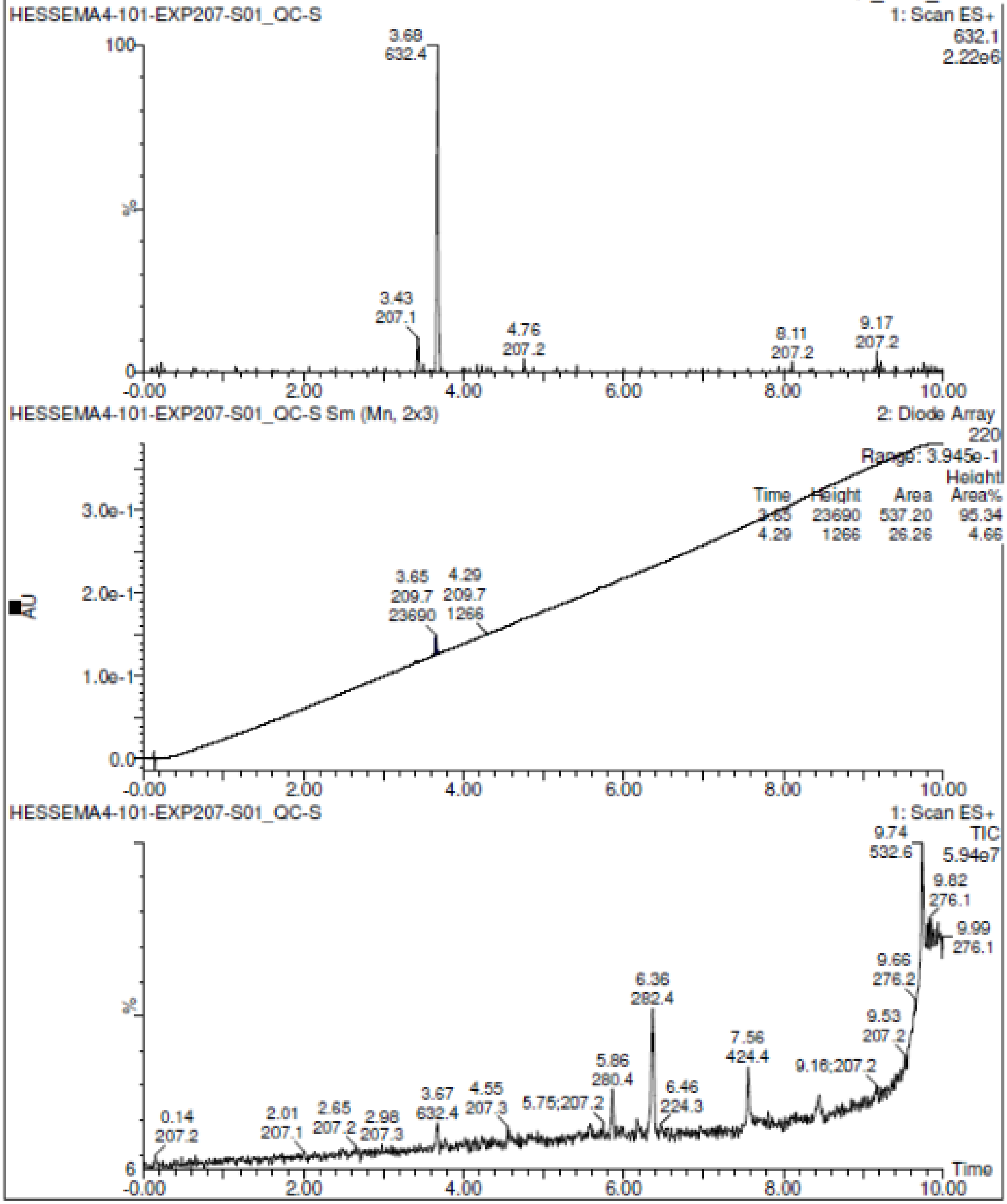

Time	Height	Area	Height Area%
3.65	23690	537.20	95.34
4.29	1266	26.26	4.66

HPLC=0.1%TFA in H2O/ACN with 1.2mL/min

EM23001590289 **19-Jan-2023 16:09:36 strubhe1-0118A-v04** **LCMS-ZQ1 Kinetex column, ZQ1_10m_Acidic**

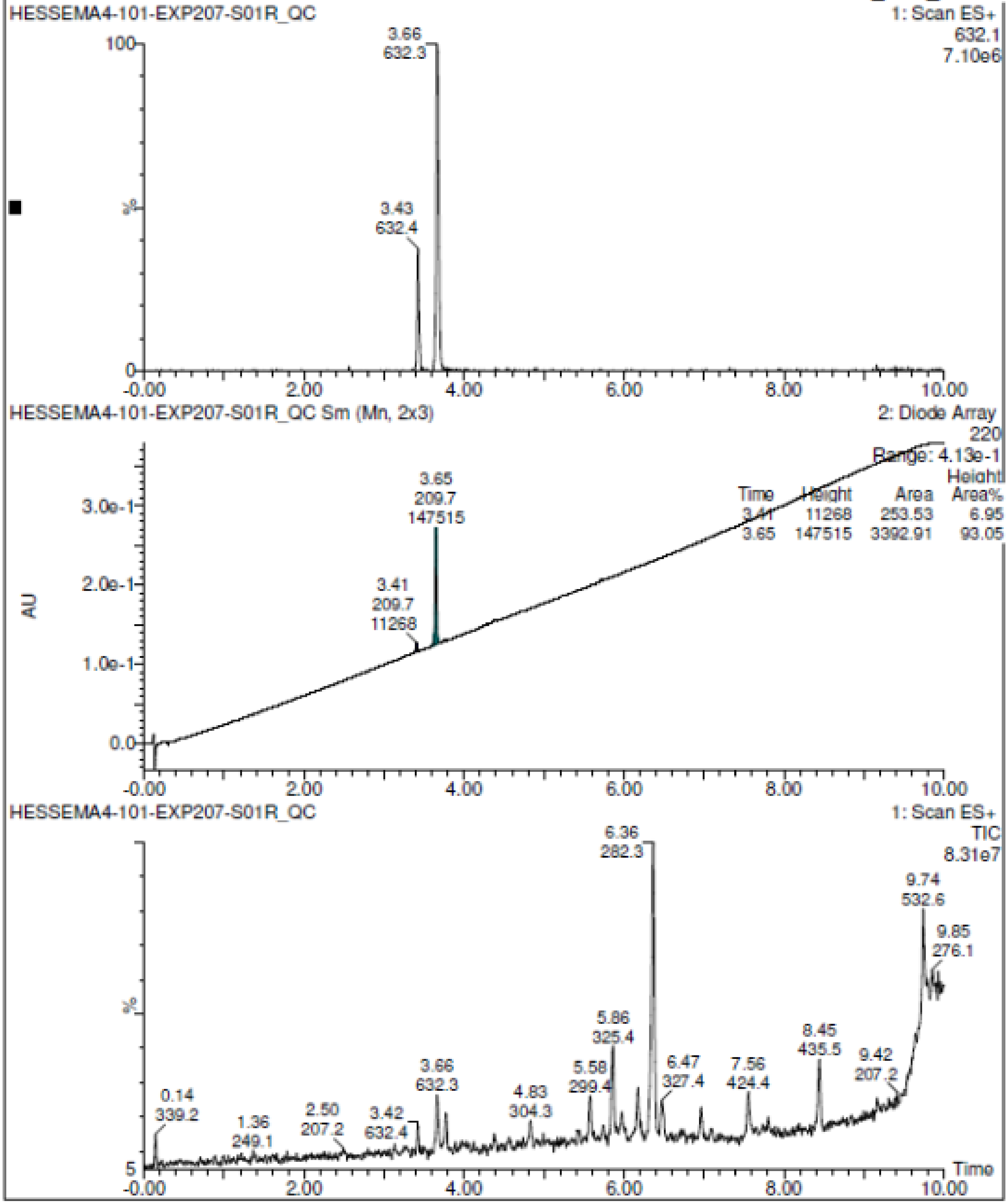

Time	Height	Area	Height Area%
3.41	11268	253.53	6.95
3.65	147515	3392.91	93.05

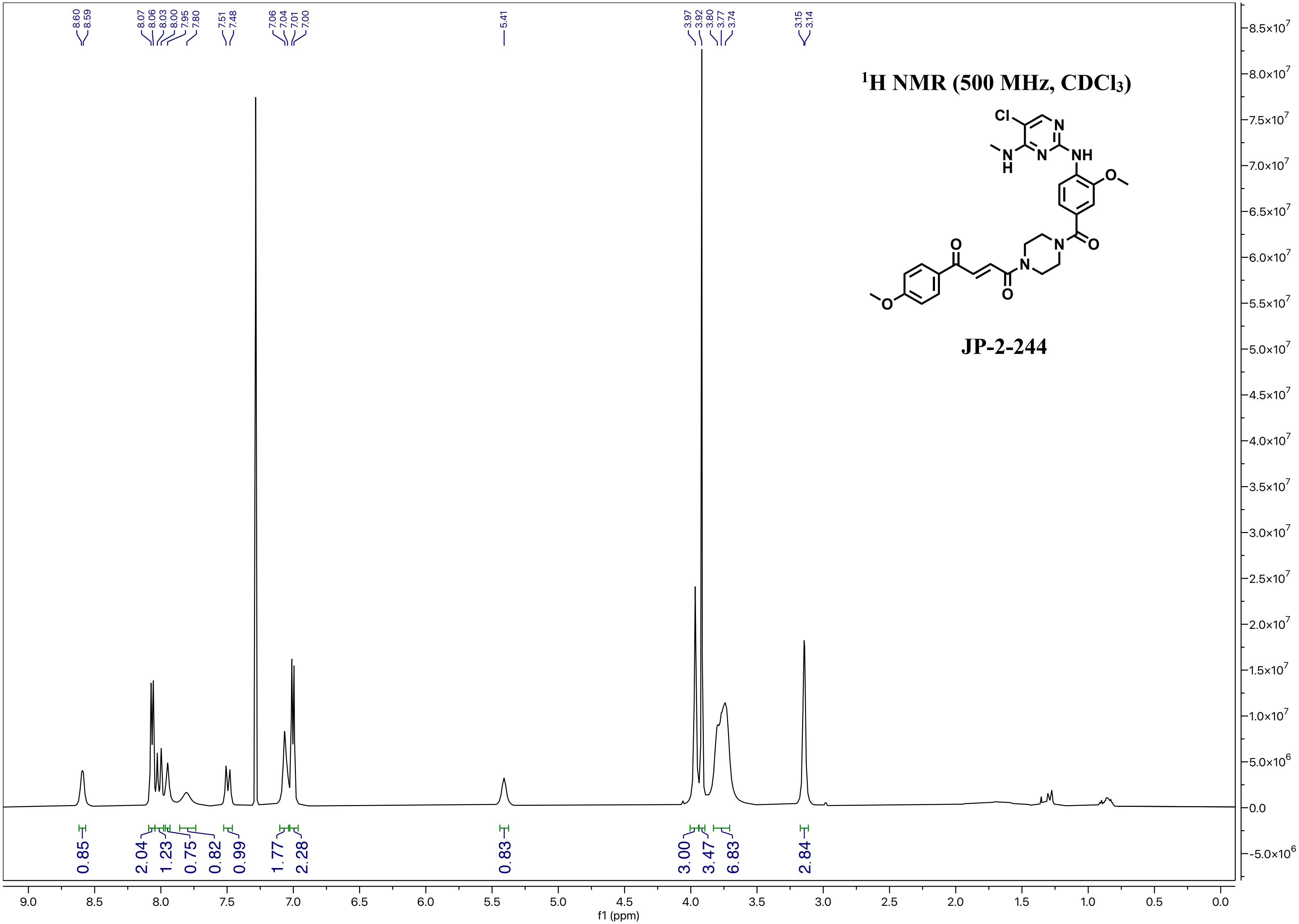

¹H NMR (500 MHz, CDCl3)
Cl
N
NH
N
H
O
O
N
N
O
O
O
JP-2-244
8.60
8.59
8.07
8.06
8.03
8.00
7.95
7.80
7.51
7.48
7.06
7.04
7.01
7.00
5.41
3.97
3.92
3.80
3.77
3.74
3.15
3.14
0.85
2.04
1.23
0.75
0.82
0.99
1.77
2.28
0.83
3.00
3.47
6.83
2.84
f1 (ppm)

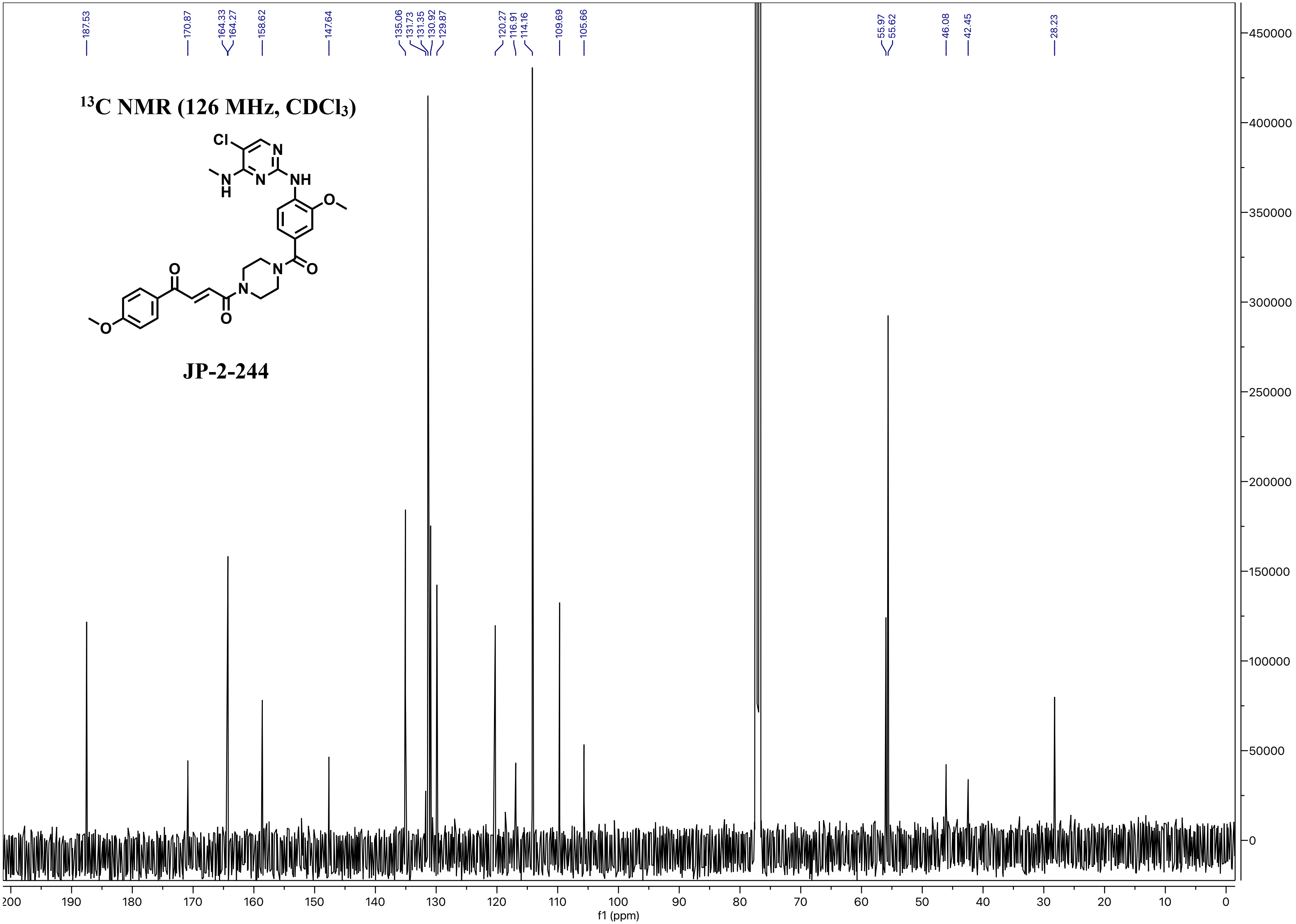

187.53
170.87
164.33
164.27
158.62
147.64
135.06
131.73
131.35
130.92
129.87
120.27
116.91
114.16
109.69
105.66
55.97
55.62
46.08
42.45
28.23
^{13}C NMR (126 MHz, CDCl$_3$)
Cl
N
NH
N
H
O
N
O
O
N
O
O
JP-2-244
450000
400000
350000
300000
250000
200000
150000
100000
50000
0
200
190
180
170
160
150
140
130
120
110
100
90
80
70
60
50
40
30
20
10
0
f1 (ppm)

HPLC=0.1%TFA in H2O/ACN with 1.2mL/min

EM23001590285 | **13-Jan-2023 15:54:05 strubhe1-0112A-v03** | **LCMS-ZQ1 Kinetex column, ZQ1_10m_Acidic**

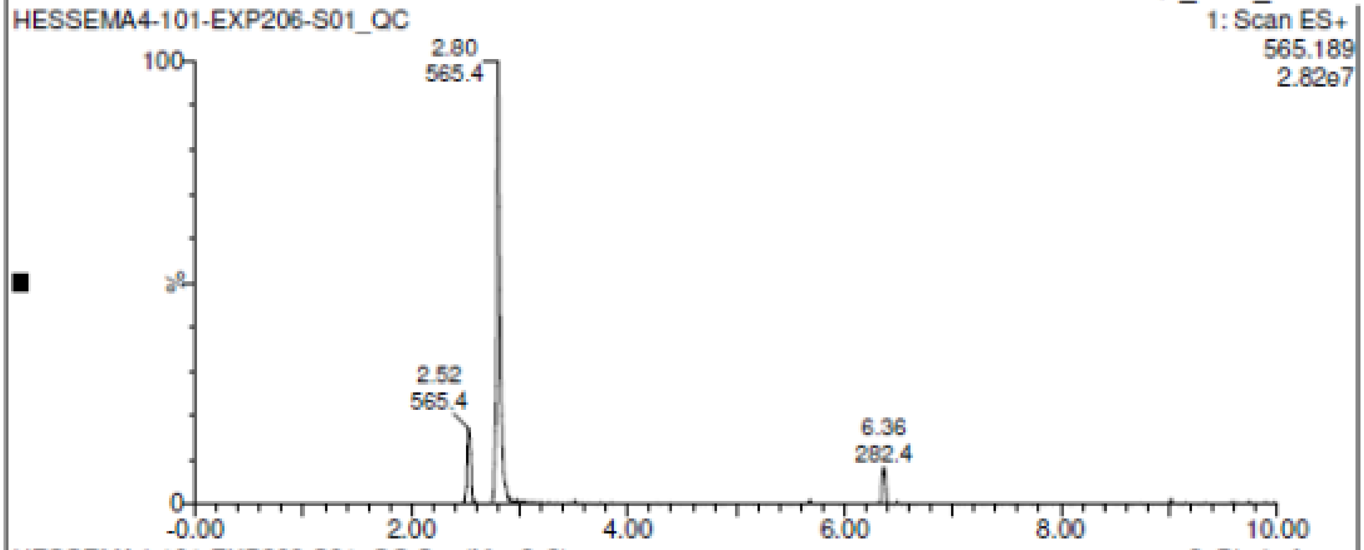

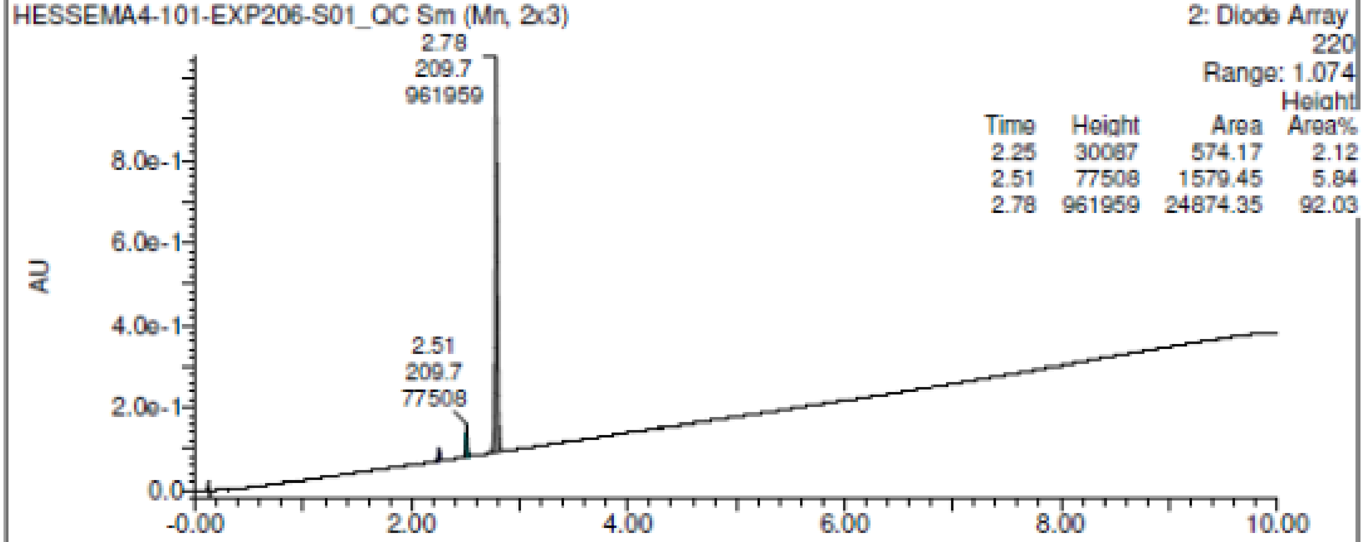

Time	Height	Area	Height Area%
2.25	30087	574.17	2.12
2.51	77508	1579.45	5.84
2.78	961959	24874.35	92.03

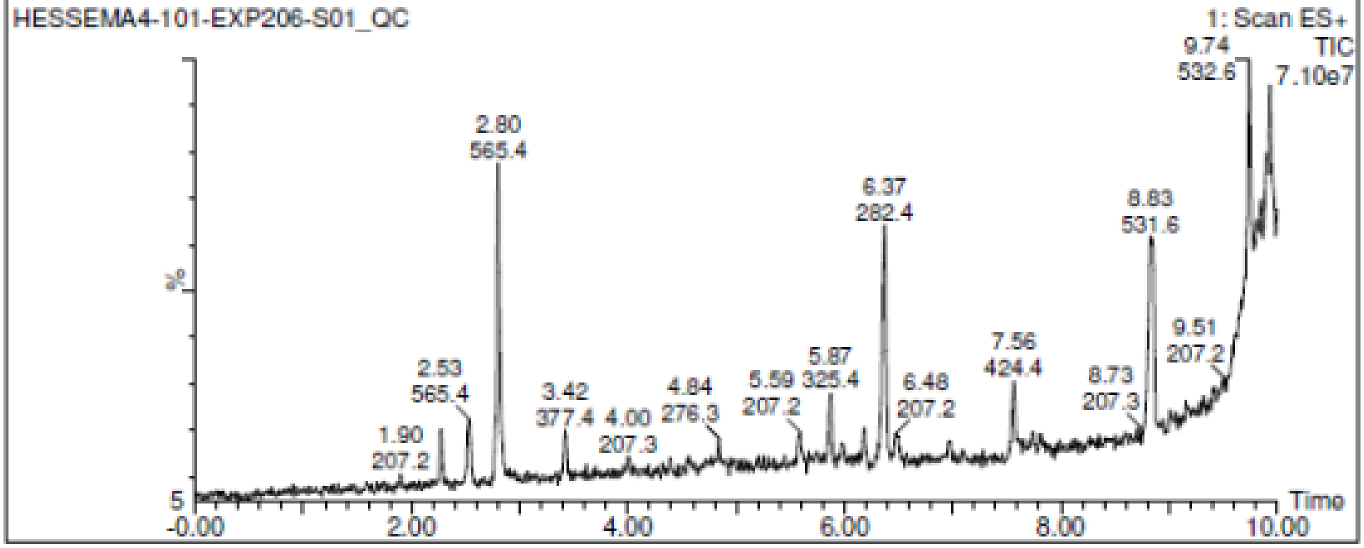

HPLC=0.1%TFA in H2O/ACN with 1.2mL/min

EM23001590285 | 19-Jan-2023 15:58:35 strubhe1-0118A-v03 | LCMS-ZQ1 Kinetex column, ZQ1_10m_Acidic

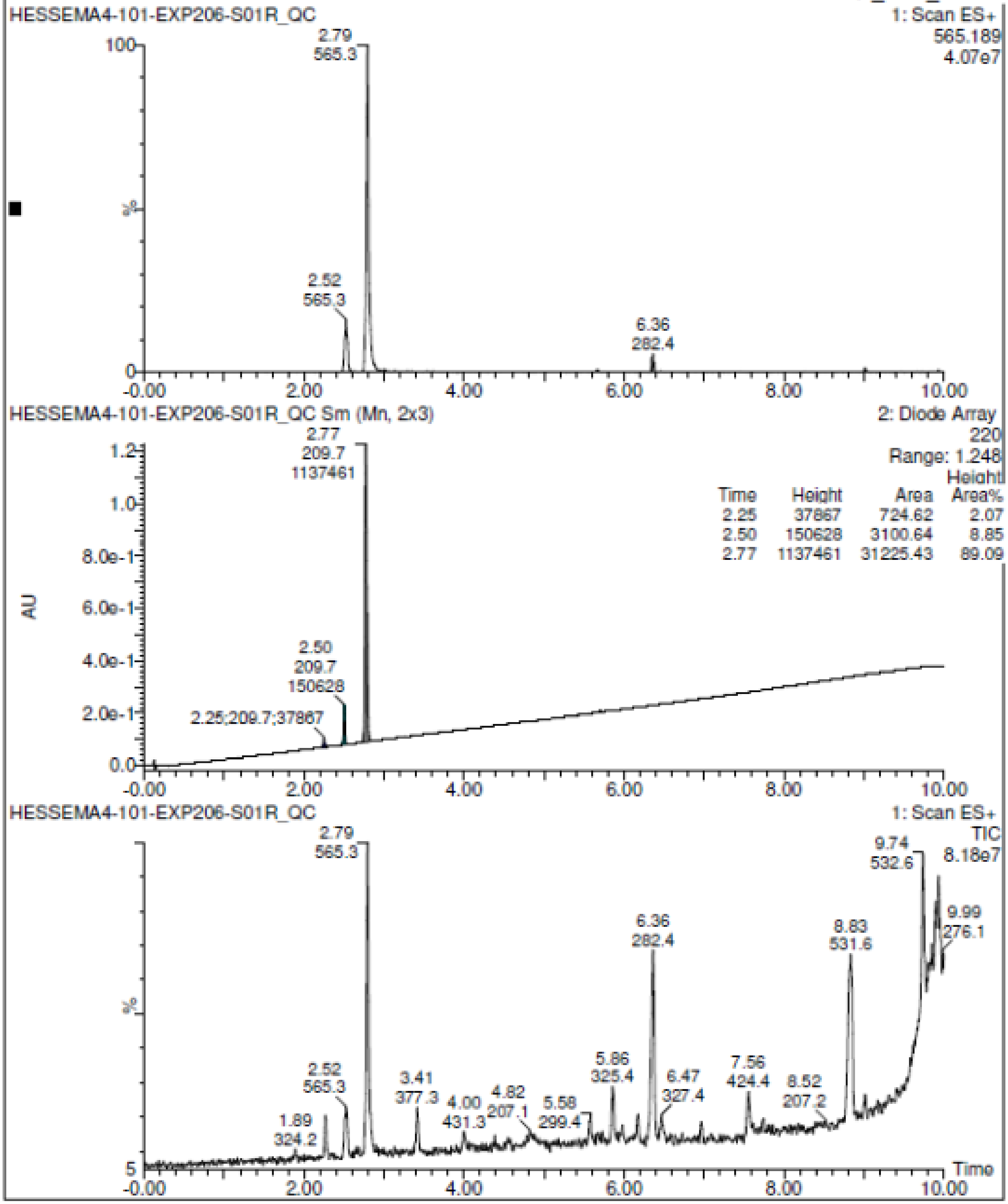

Time	Height	Area	Height Area%
2.25	37867	724.62	2.07
2.50	150628	3100.64	8.85
2.77	1137461	31225.43	89.09

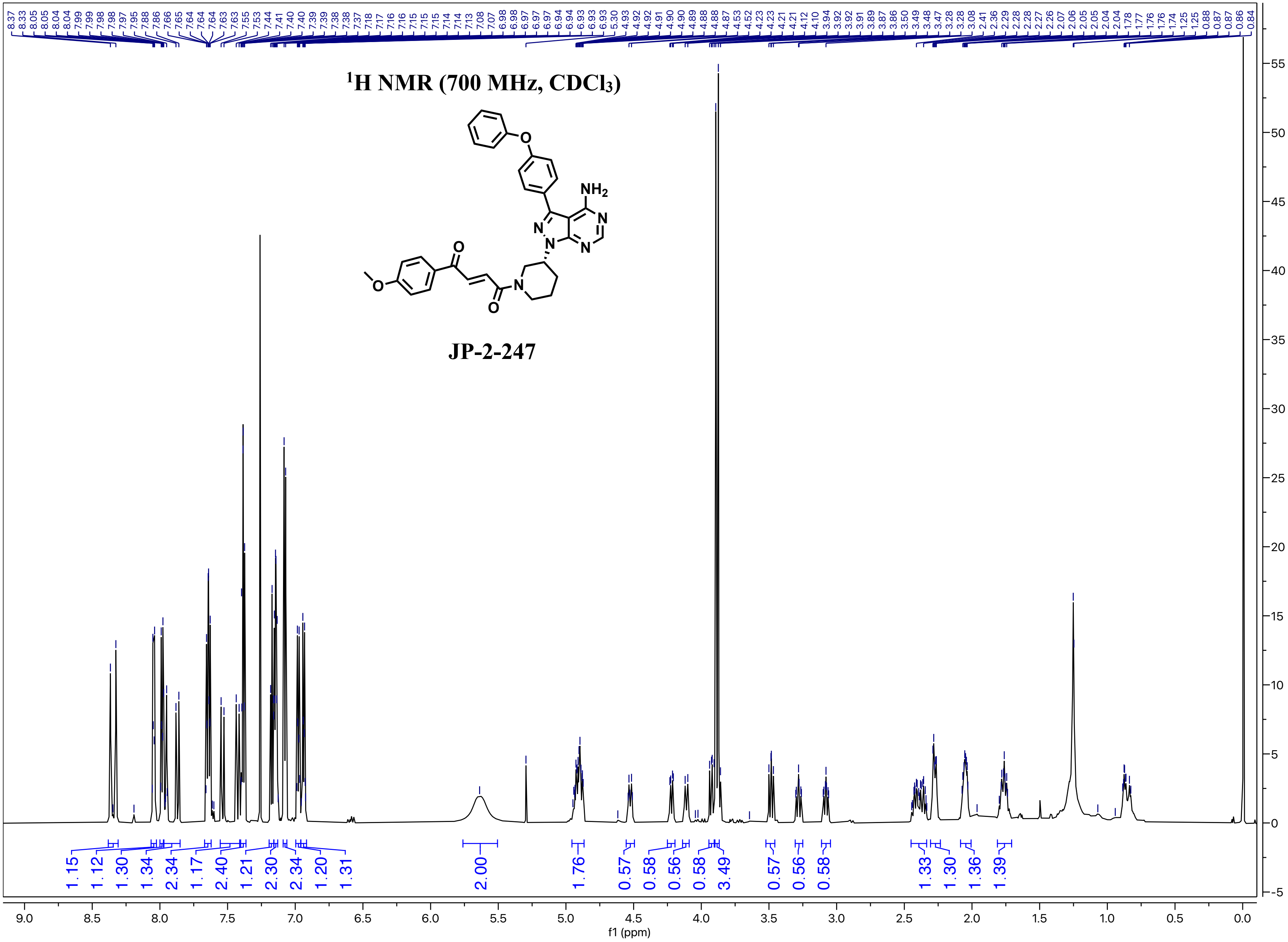

¹H NMR (700 MHz, CDCl3)
NH2
N
O
JP-2-247
f1 (ppm)

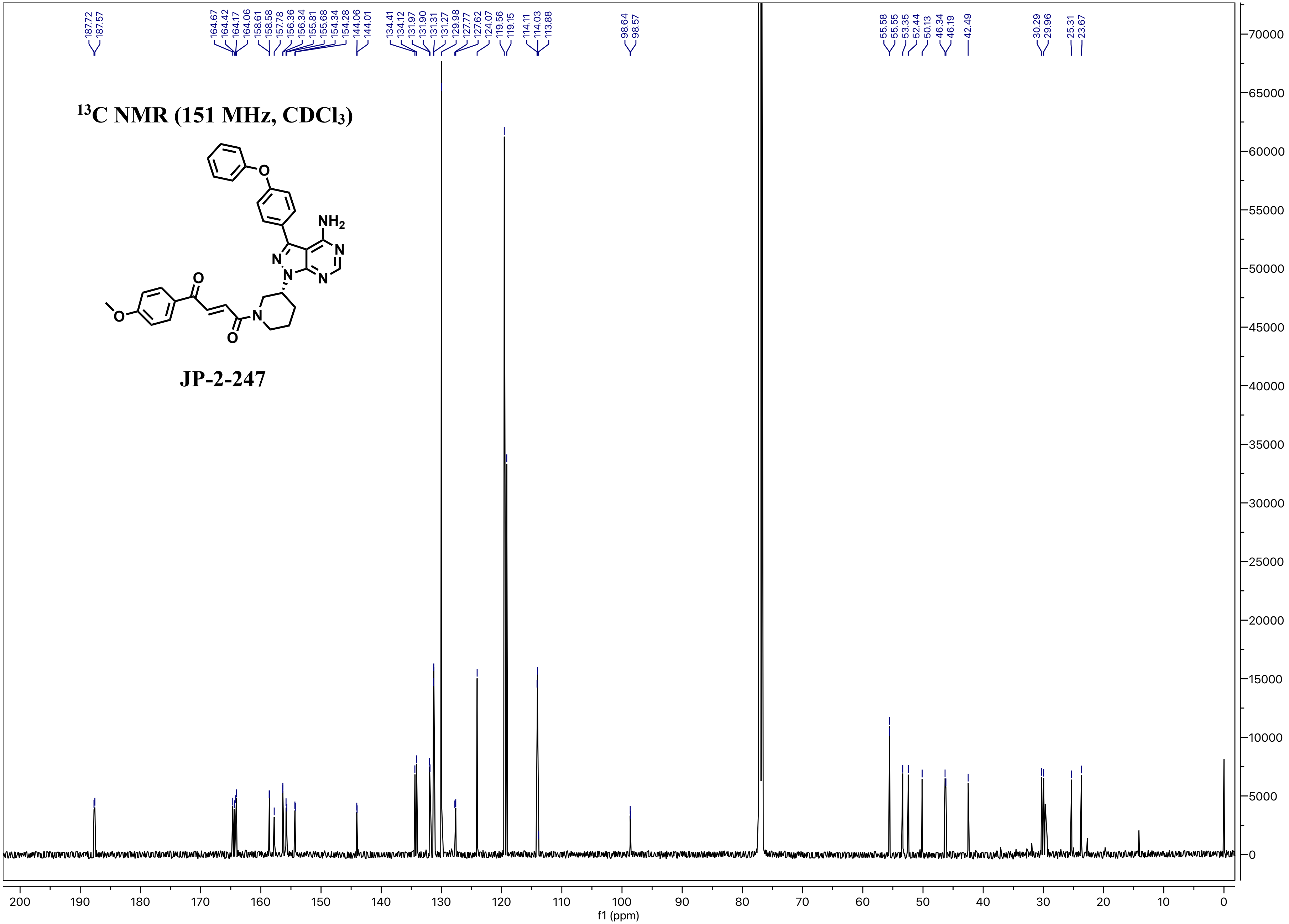

13C NMR (151 MHz, CDCl3)
JP-2-247
187.72
187.57
164.67
164.42
164.17
164.06
158.61
158.58
157.78
156.36
156.34
155.81
155.68
154.34
154.28
144.06
144.01
134.41
134.12
131.97
131.90
131.31
131.27
129.98
127.77
127.62
124.07
119.56
119.15
114.11
114.03
113.88
98.64
98.57
55.58
55.55
53.35
52.44
50.13
46.34
46.19
42.49
30.29
29.96
25.31
23.67
f1 (ppm)

HPLC=0.1%TFA in H2O/ACN with 1.2mL/min

EM23001590284 **13-Jan-2023 14:56:11 strubhe1-0112A-v02** **LCMS-ZQ1 Kinetex column, ZQ1_10m_Acidic**

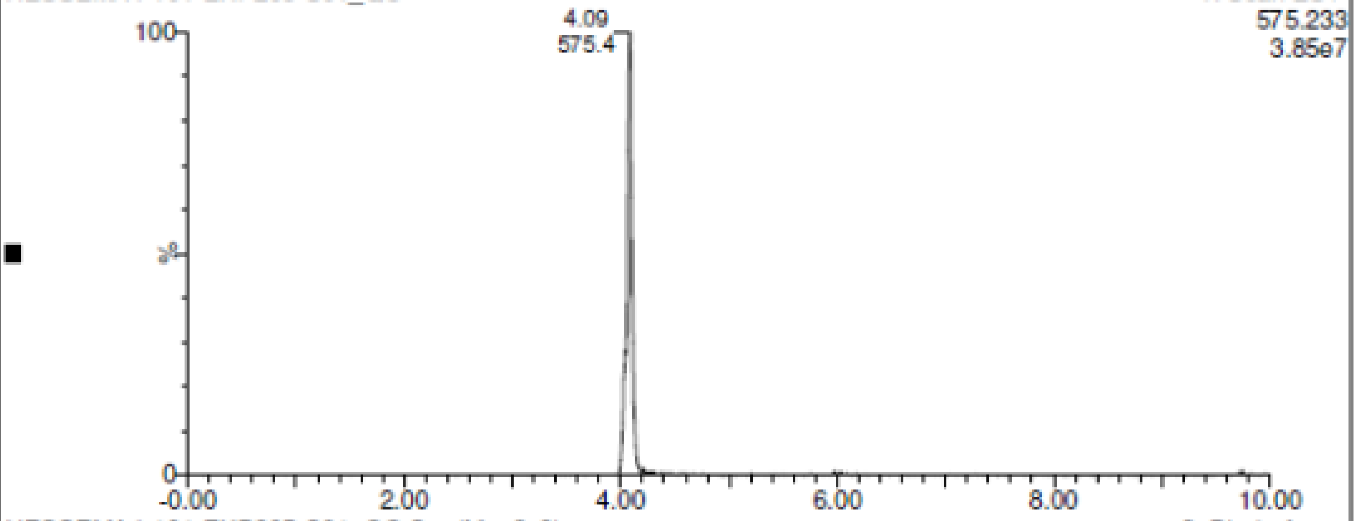

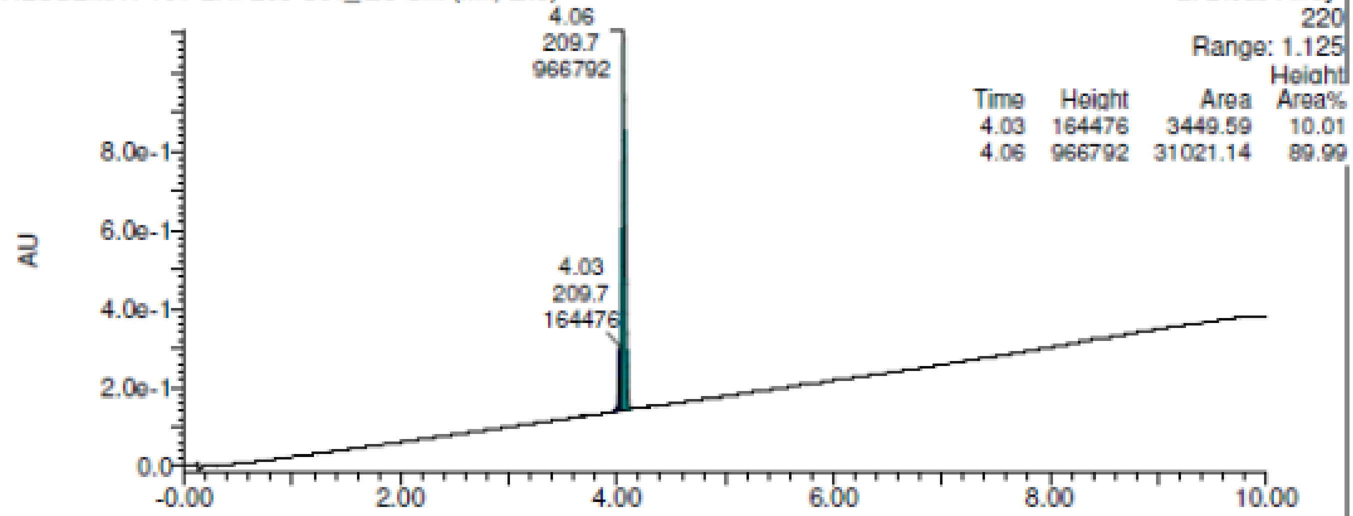

Time	Height	Area	Height Area%
4.03	164476	3449.59	10.01
4.06	966792	31021.14	89.99

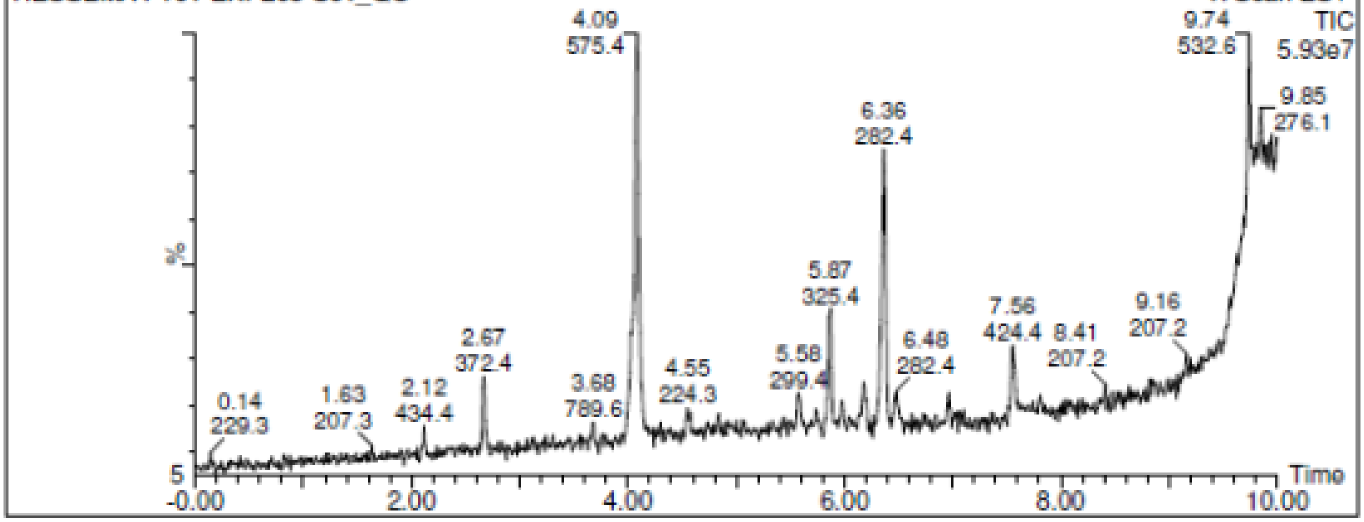

HPLC=0.1%TFA in H2O/ACN with 1.2mL/min

EM23001590284 **19-Jan-2023 15:46:53 strubhe1-0118A-v02** **LCMS-ZQ1 Kinetex column, ZQ1_10m_Acidic**

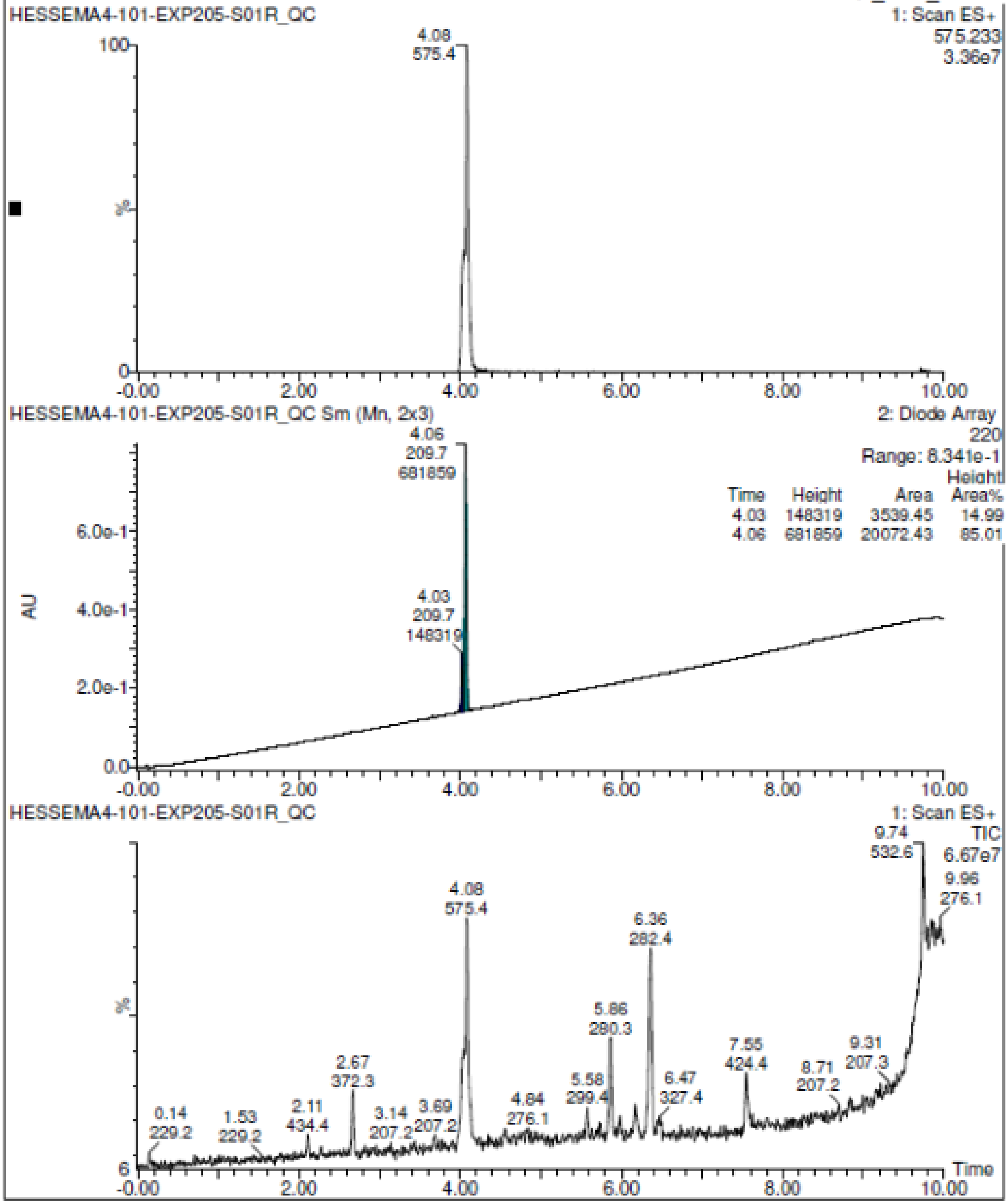

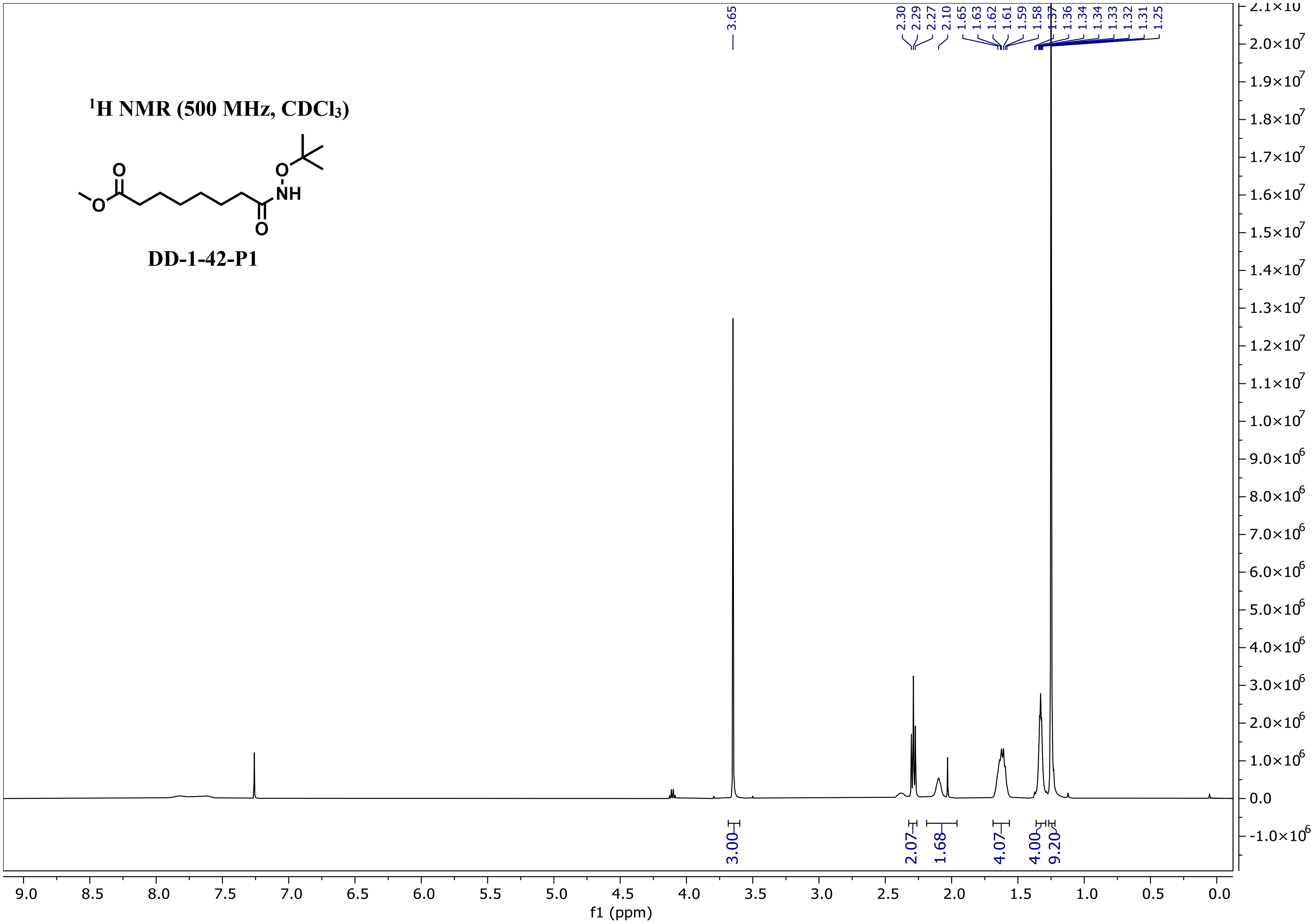

^{1}H NMR (500 MHz, $CDCl_3$)
DD-1-42-P1
3.65
2.30
2.29
2.27
2.10
1.65
1.63
1.62
1.61
1.59
1.58
1.37
1.36
1.34
1.34
1.33
1.32
1.31
1.25
3.00
2.07
1.68
4.07
4.00
9.20
f1 (ppm)

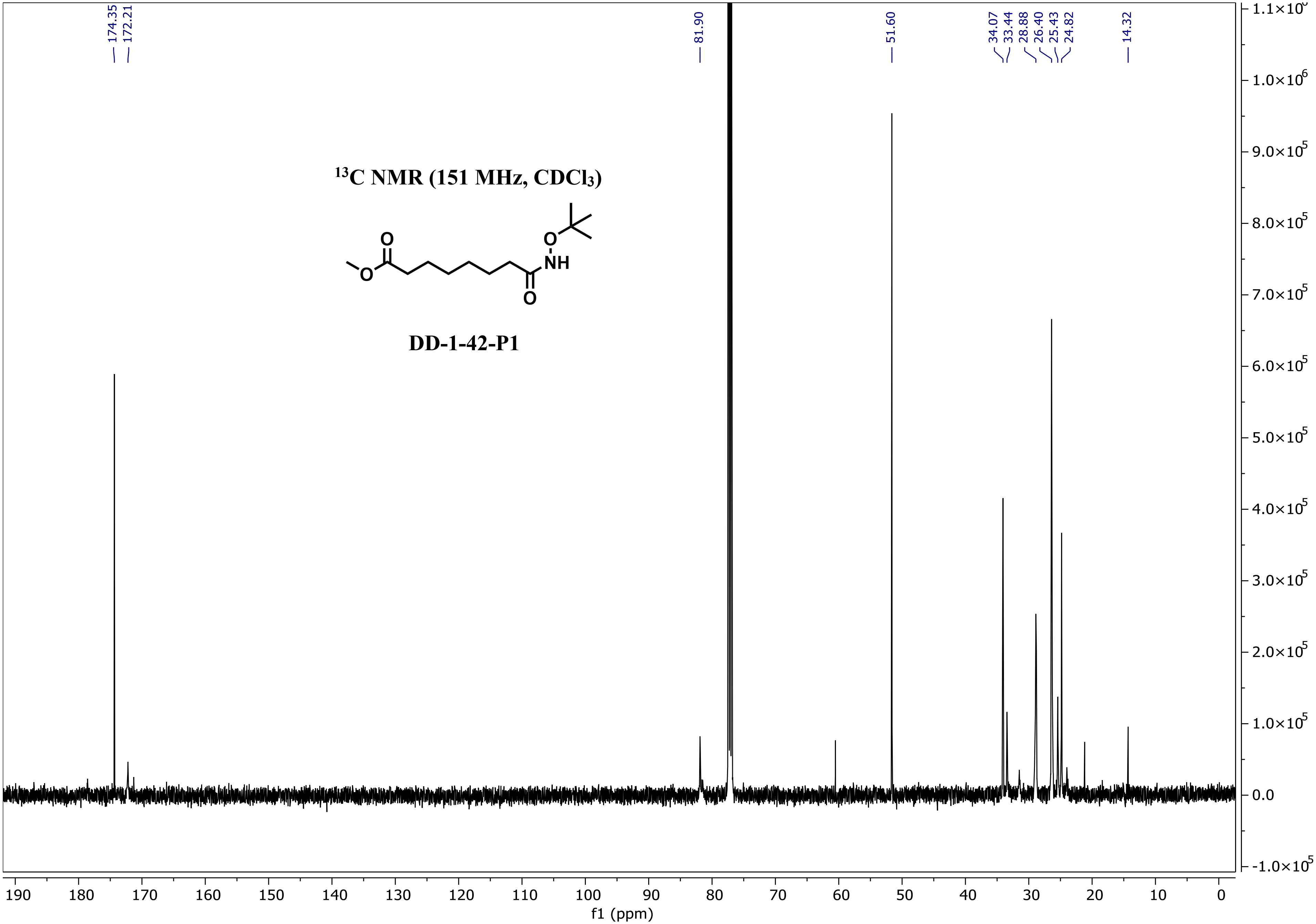

174.35
172.21
81.90
51.60
34.07
33.44
28.88
26.40
25.43
24.82
14.32
¹³C NMR (151 MHz, CDCl₃)
O
O
O
NH
O
O
DD-1-42-P1
1.0×10⁶
9.0×10⁵
8.0×10⁵
7.0×10⁵
6.0×10⁵
5.0×10⁵
4.0×10⁵
3.0×10⁵
2.0×10⁵
1.0×10⁵
0.0
-1.0×10⁵
190 180 170 160 150 140 130 120 110 100 90 80 70 60 50 40 30 20 10 0
f1 (ppm)

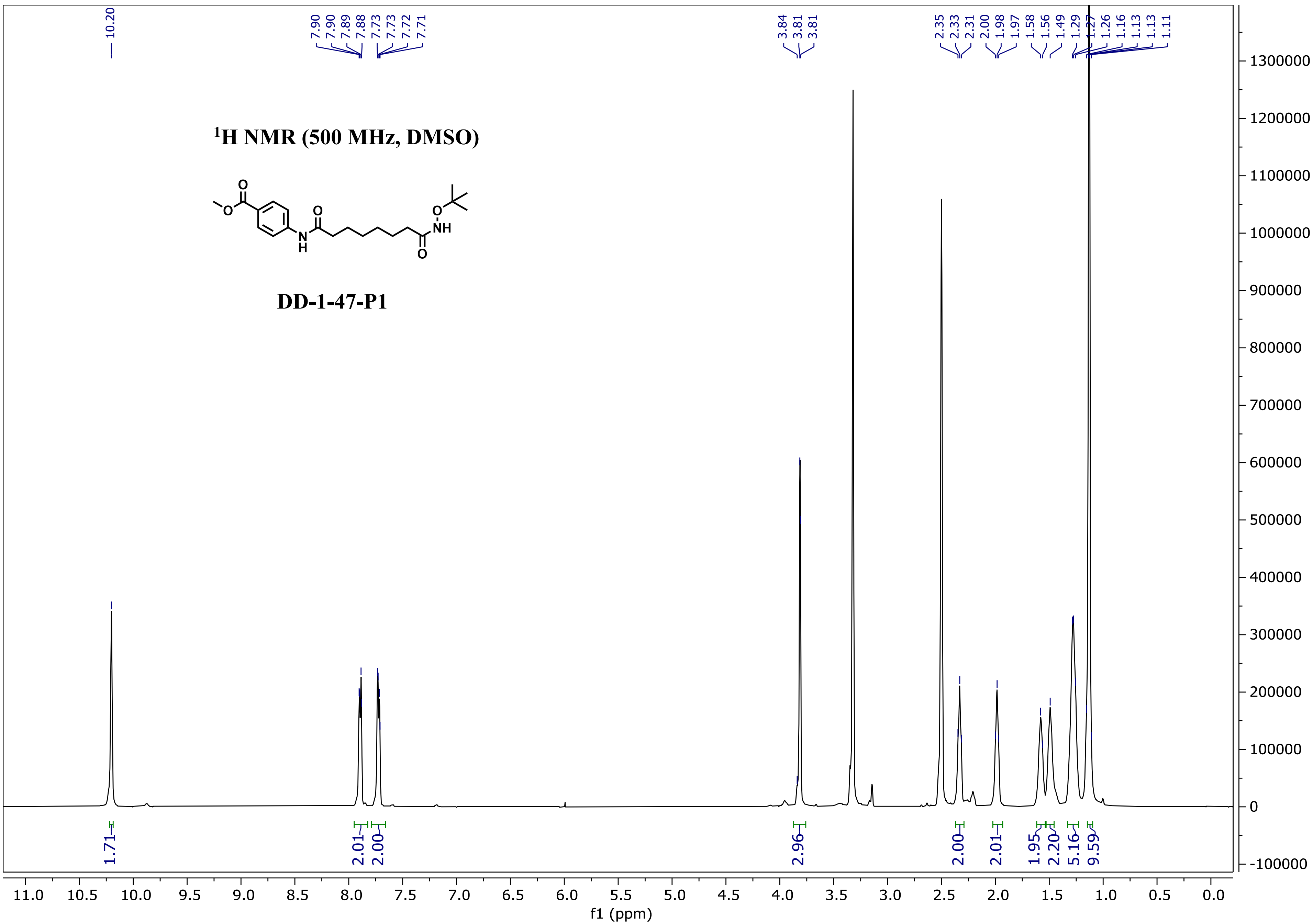
1H NMR (500 MHz, DMSO)
DD-1-47-P1
10.20
7.90 7.90 7.89 7.88 7.73 7.73 7.72 7.71
3.84 3.81 3.81
2.35 2.33 2.31 2.00 1.98 1.97 1.58 1.56 1.49 1.29 1.27 1.26 1.16 1.13 1.13 1.11
1.71 2.01 2.00 2.96 2.00 2.01 1.95 2.20 5.16 9.59
f1 (ppm)

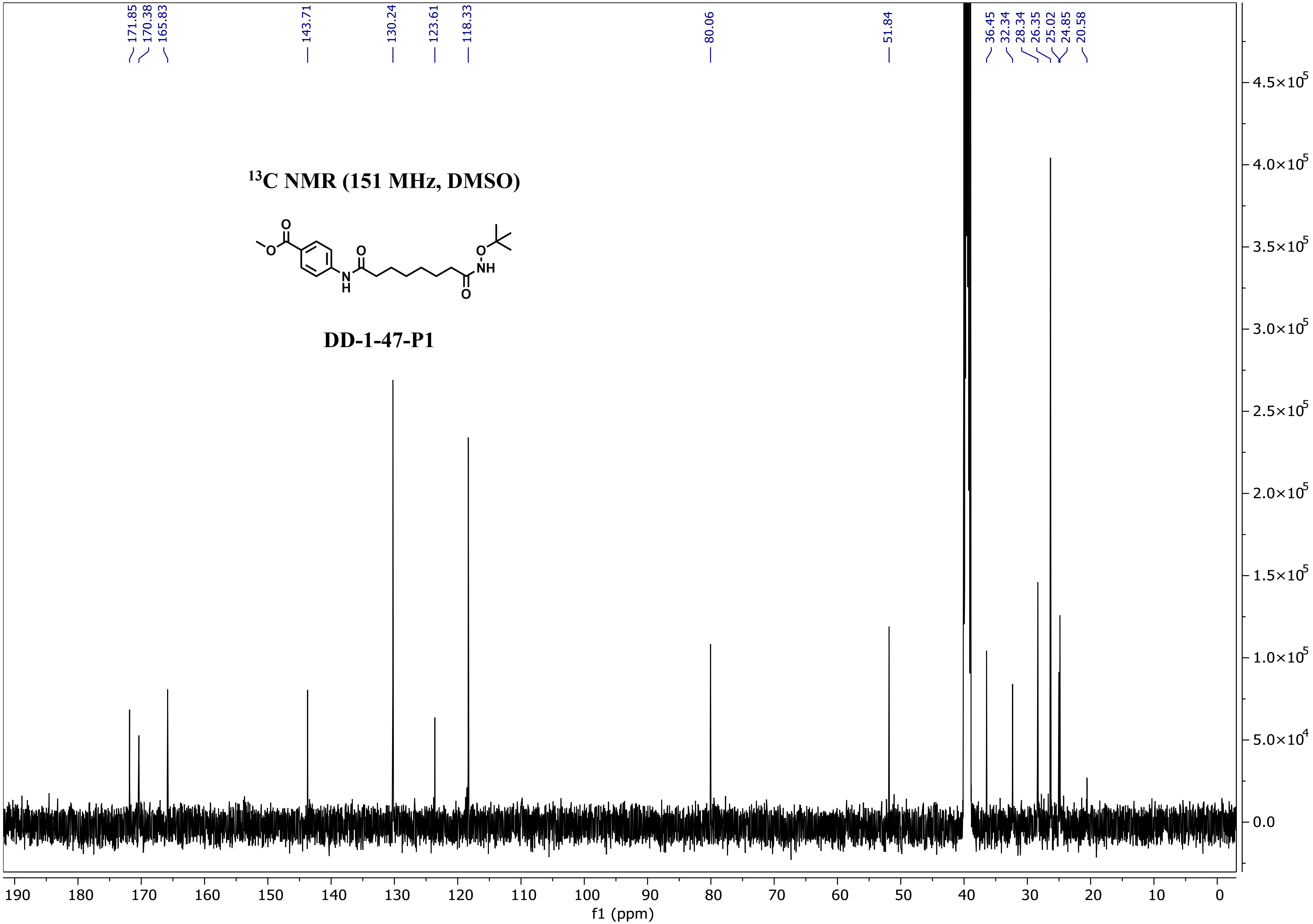

171.85
170.38
165.83
143.71
130.24
123.61
118.33
80.06
51.84
36.45
32.34
28.34
26.35
25.02
24.85
20.58
^{13}C NMR (151 MHz, DMSO)
O
O
O
N
H
O
NH
O
DD-1-47-P1
4.5×10^5
4.0×10^5
3.5×10^5
3.0×10^5
2.5×10^5
2.0×10^5
1.5×10^5
1.0×10^5
5.0×10^4
0.0
190
180
170
160
150
140
130
120
110
100
90
80
70
60
50
40
30
20
10
0
f1 (ppm)

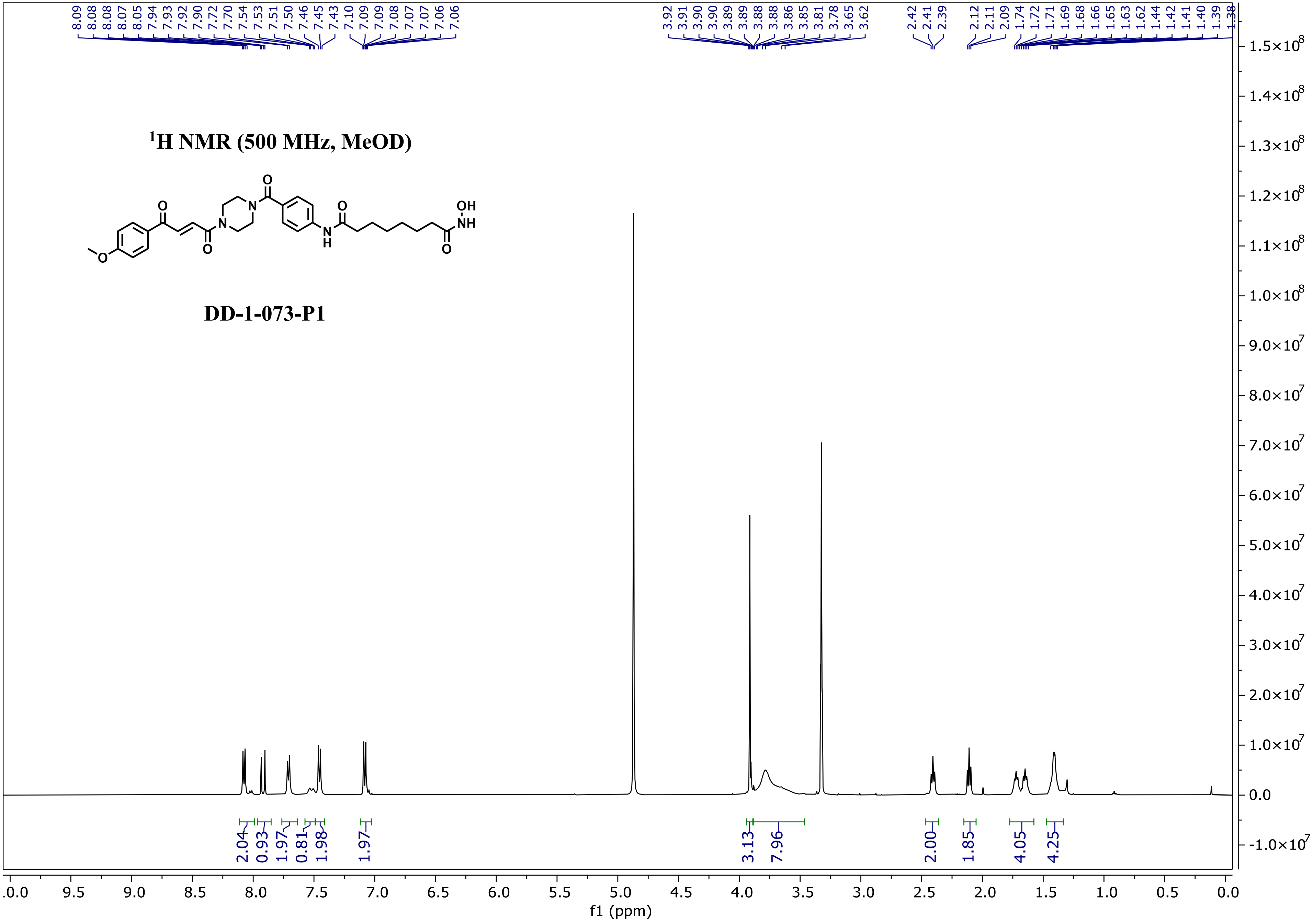

1H NMR (500 MHz, MeOD)
DD-1-073-P1
f1 (ppm)

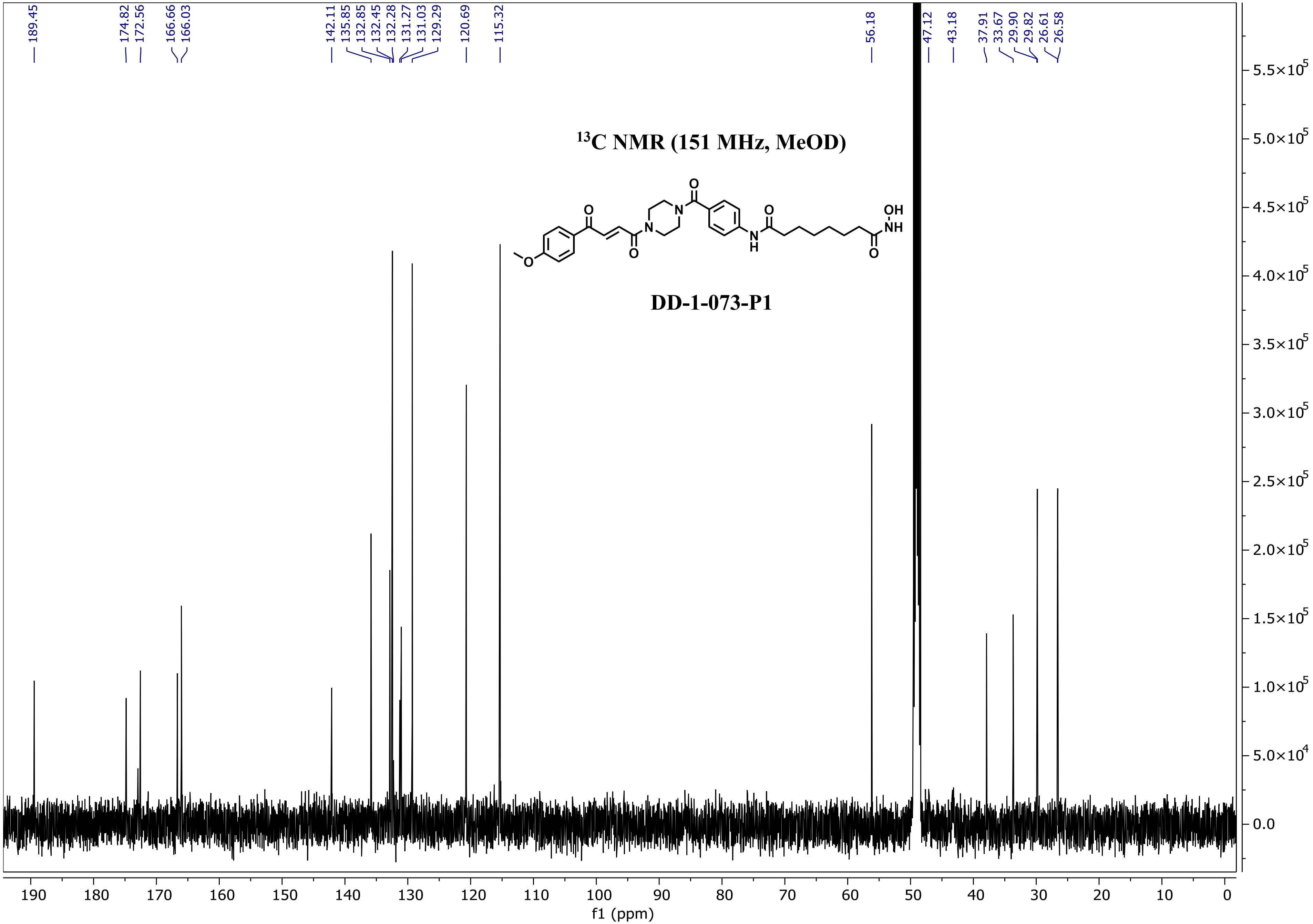
189.45
174.82
172.56
166.66
166.03
142.11
135.85
132.85
132.45
132.28
131.27
131.03
129.29
120.69
115.32
56.18
47.12
43.18
37.91
33.67
29.90
29.82
26.61
26.58
13C NMR (151 MHz, MeOD)
DD-1-073-P1
f1 (ppm)

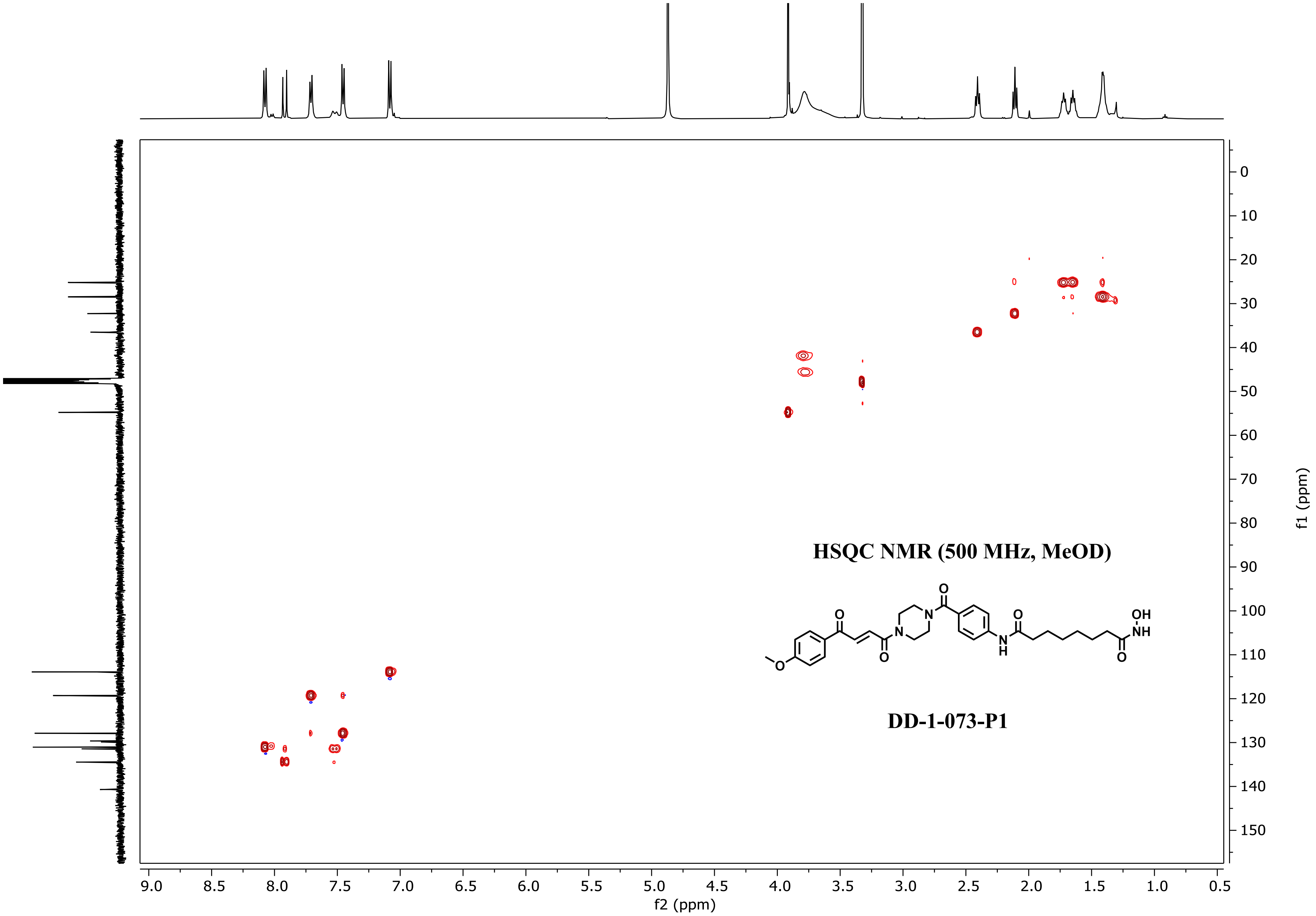

HSQC NMR (500 MHz, MeOD)

DD-1-073-P1

HPLC=0.1%TFA in H2O/ACN with 1.2mL/min

EM23001597802	02-Mar-2023<br>14:21:04<br>strubhe1-0302-v05	LCMS-QDa1<br>Kinetex column,<br>QDa1_2m_Acidic

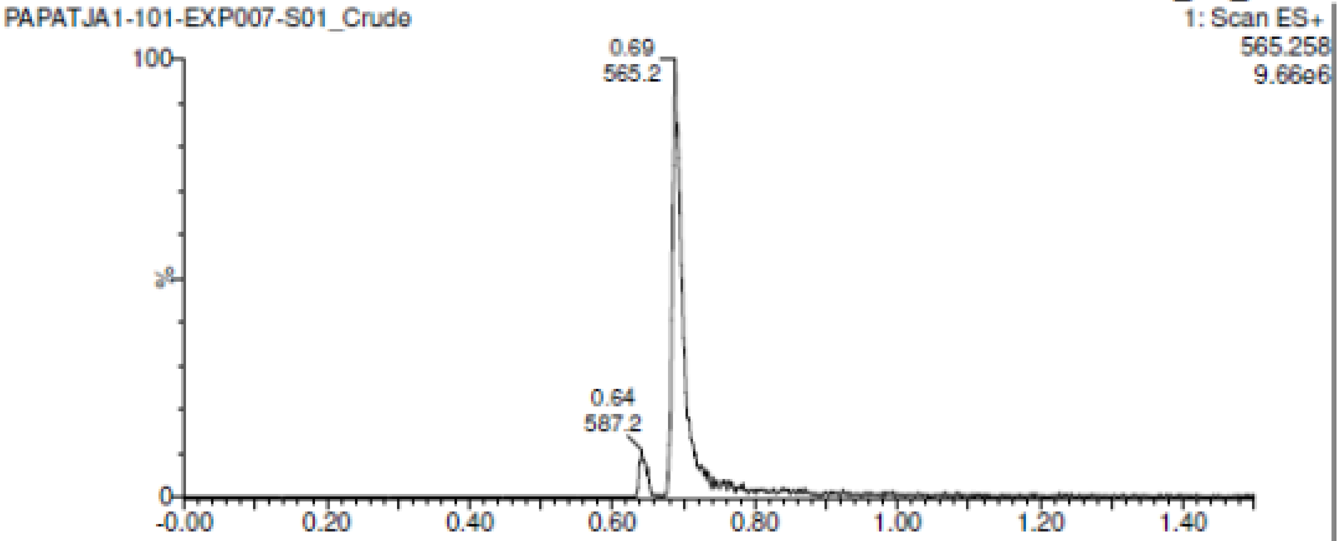

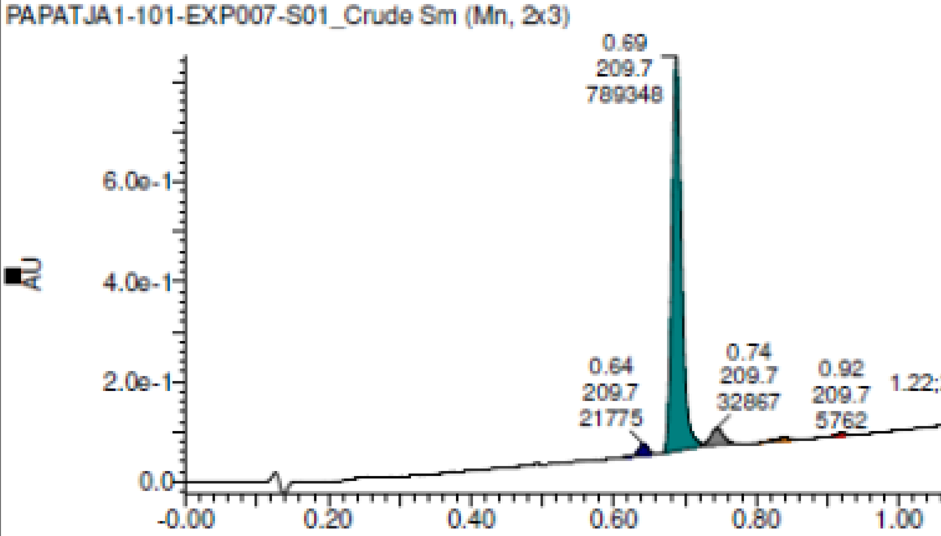

Time	Height	Area	Area%
0.6417	21775	350.51	2.52
0.6875	789348	12416.70	89.41
0.7450	32867	698.93	5.03
0.8383	6367	166.68	1.20
0.9192	5762	84.11	0.61
1.1275	1511	17.87	0.13
1.1692	2792	39.89	0.29
1.2175	3790	53.64	0.39
1.2975	4333	59.11	0.43

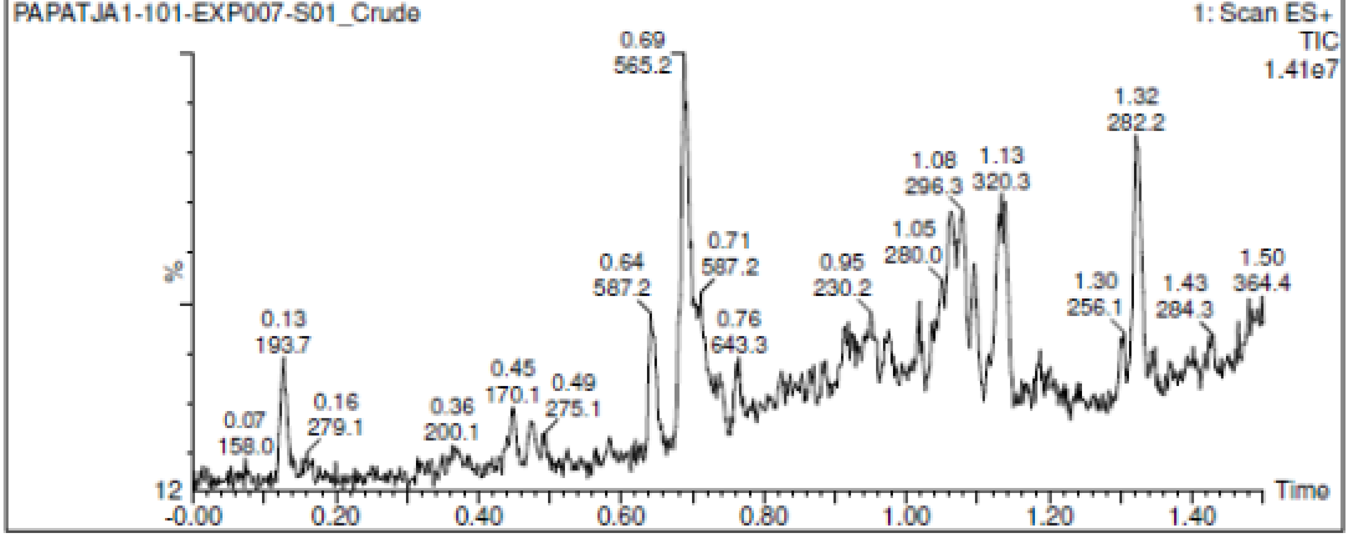

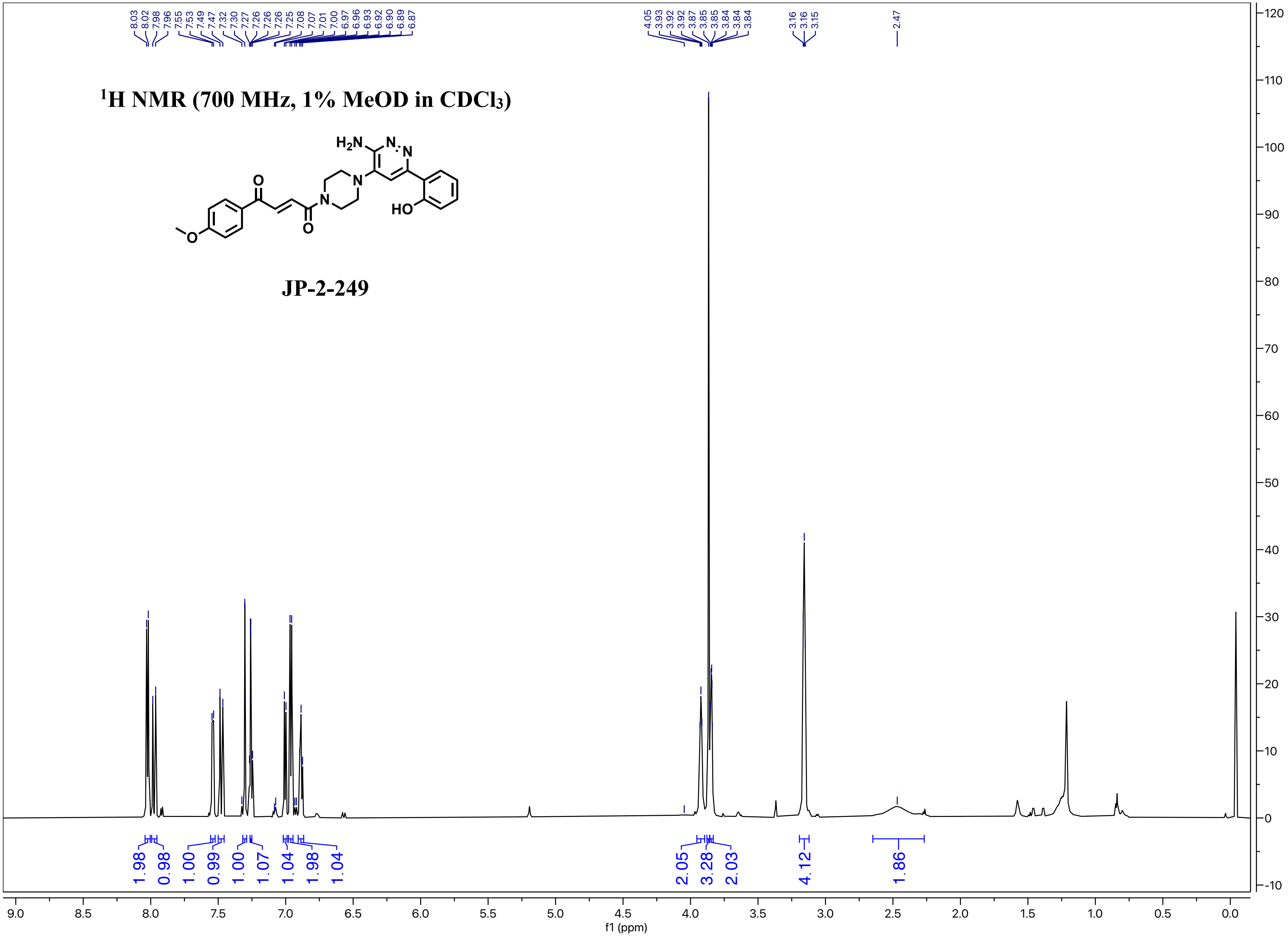
8.03
8.02
7.98
7.96
7.55
7.53
7.49
7.47
7.32
7.30
7.27
7.26
7.26
7.26
7.25
7.08
7.07
7.01
7.00
6.97
6.96
6.93
6.92
6.90
6.89
6.87
4.05
3.93
3.92
3.92
3.87
3.85
3.85
3.84
3.84
3.84
3.16
3.16
3.15
2.47
1H NMR (700 MHz, 1% MeOD in CDCl3)
H2N
N
N
O
N
N
HO
O
O
JP-2-249
1.98
0.98
1.00
0.99
1.00
1.07
1.04
1.98
1.04
2.05
3.28
2.03
4.12
1.86
f1 (ppm)

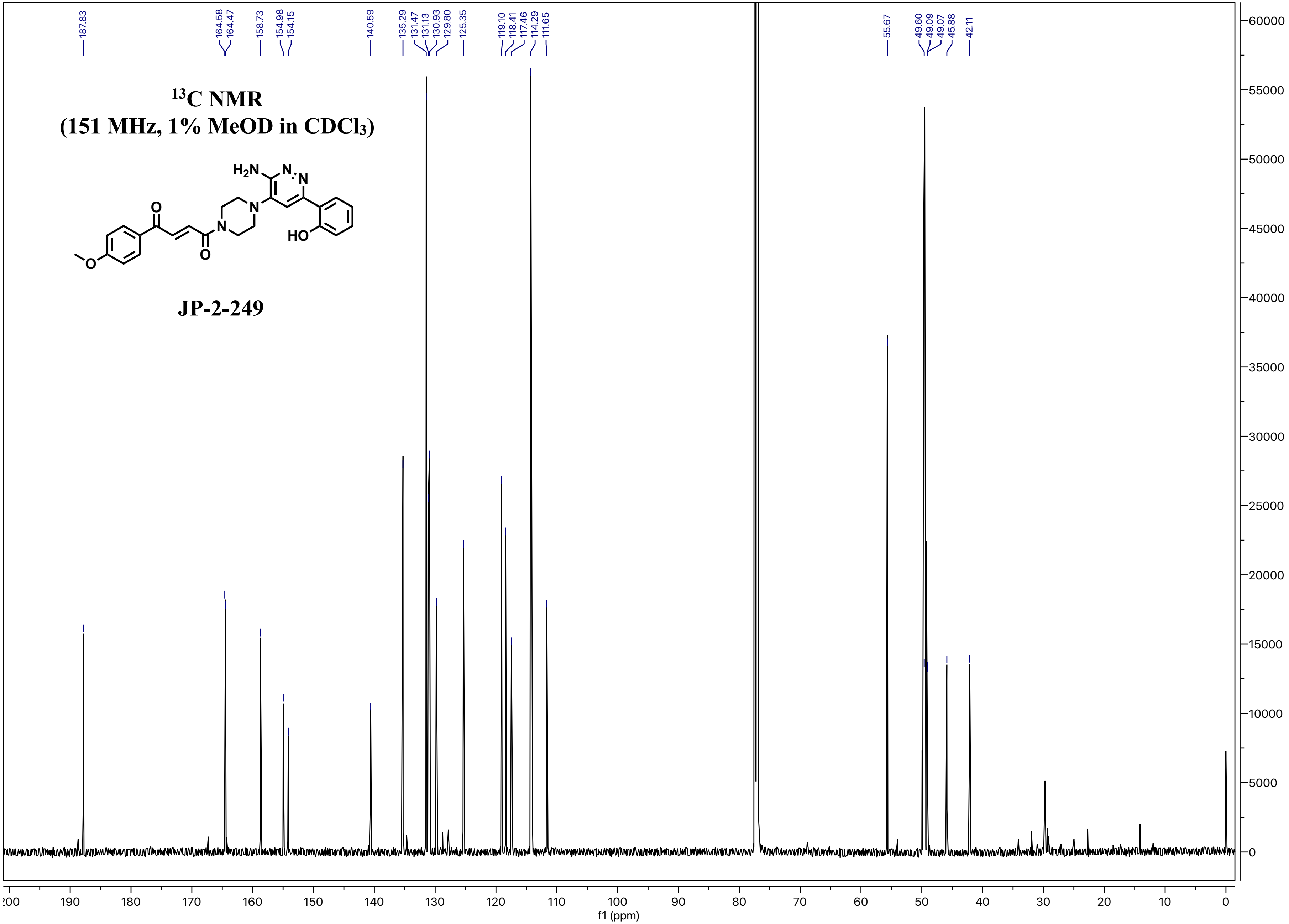

187.83
164.58
164.47
158.73
154.98
154.15
140.59
135.29
131.47
131.13
130.93
129.80
125.35
119.10
118.41
117.46
114.29
111.65
55.67
49.60
49.09
49.07
45.88
42.11
^{13}C NMR
(151 MHz, 1% MeOD in $CDCl_3$)
H_2N
N
N
O
N
N
HO
O
O
JP-2-249
60000
55000
50000
45000
40000
35000
30000
25000
20000
15000
10000
5000
0
200
190
180
170
160
150
140
130
120
110
100
90
80
70
60
50
40
30
20
10
0
f1 (ppm)

HPLC=0.1%TFA in H2O/ACN with 1.2mL/min

EM23001590282 | 13-Jan-2023 14:43:28 strubhe1-0112A-v01 | LCMS-ZQ1 Kinetex column, ZQ1_10m_Acidic

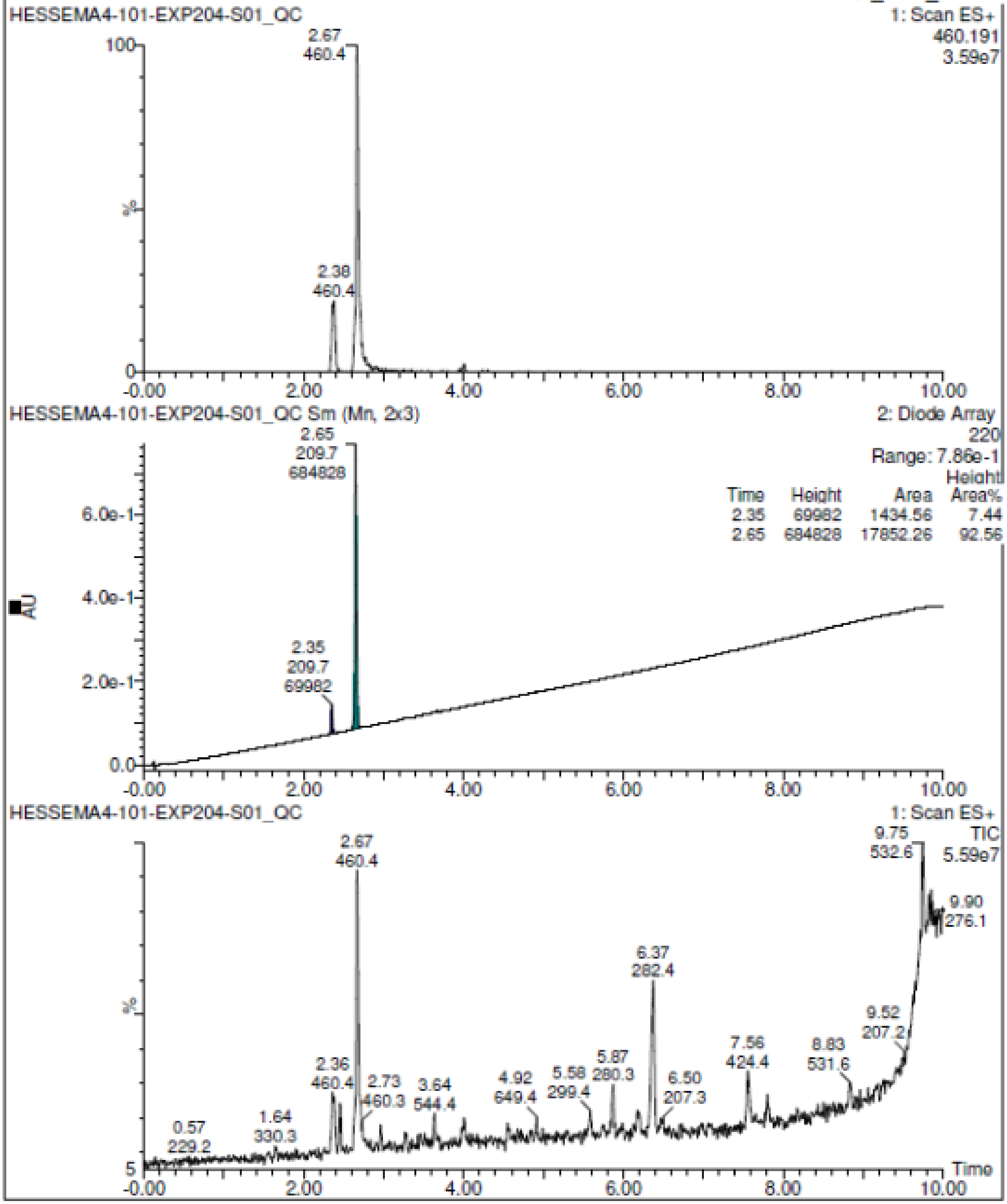

Time	Height	Area	Height Area%
2.35	69982	1434.56	7.44
2.65	684828	17852.26	92.56

HPLC=0.1%TFA in H2O/ACN with 1.2mL/min

EM23001590282

13-Jan-2023
14:43:28
strubhe1-0112A-v01

LCMS-ZQ1
Kinetex column,
ZQ1_10m_Acidic

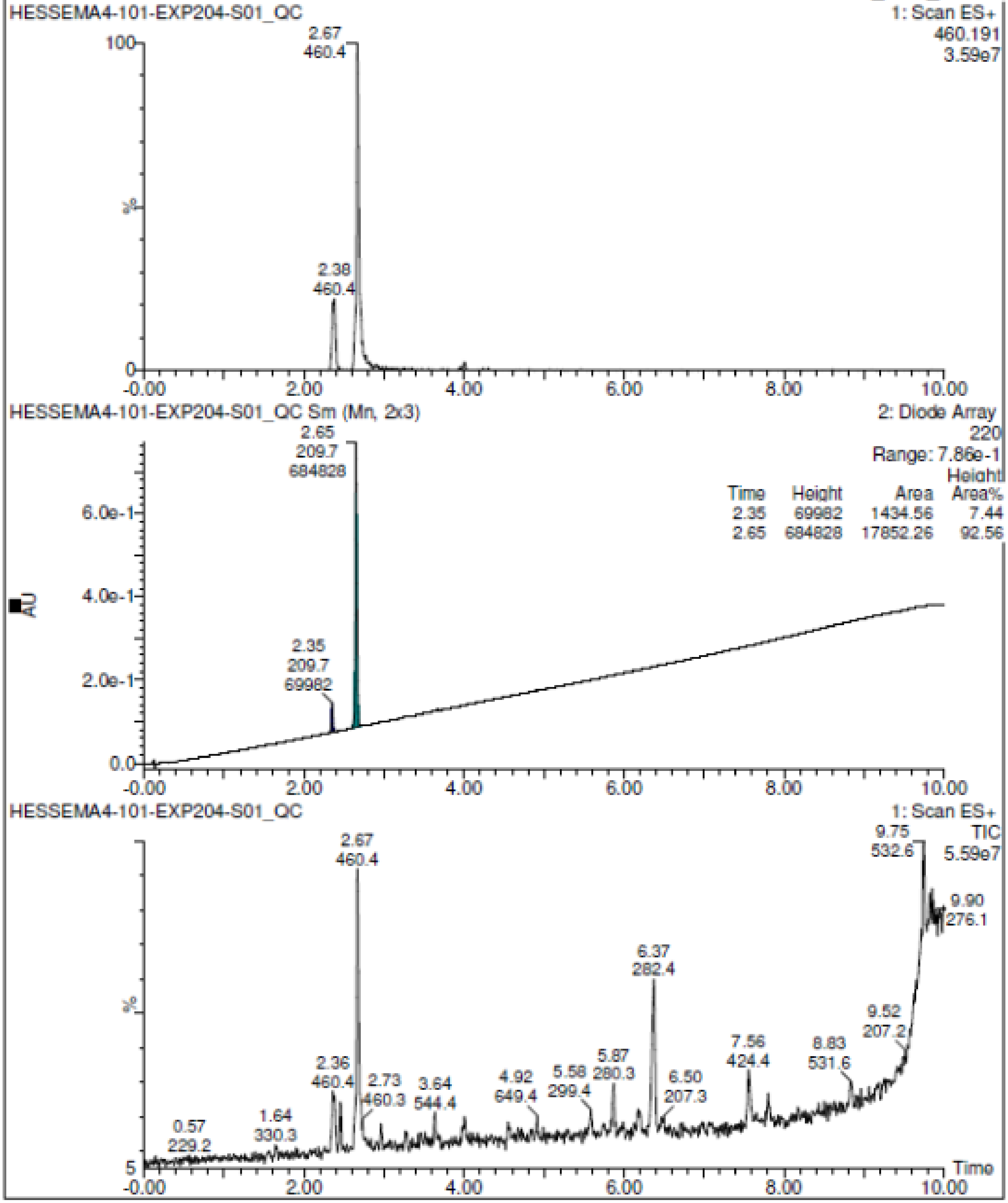

Supplement: Supplementary file 1 — oc2c01317_si_001.pdf [file oc2c01317_si_001.pdf]
